# Defining priority areas for blue whale conservation and investigating overlap with vessel traffic in Chilean Patagonia, using a fast-fitting movement model

**DOI:** 10.1038/s41598-021-82220-5

**Published:** 2021-02-01

**Authors:** Luis Bedriñana-Romano, Rodrigo Hucke-Gaete, Francisco A. Viddi, Devin Johnson, Alexandre N. Zerbini, Juan Morales, Bruce Mate, Daniel M. Palacios

**Affiliations:** 1 Instituto de Ciencias Marinas y Limnológicas, Facultad de Ciencias, Universidad Austral de Chile, Casilla 567, Valdivia, Chile; 2 NGO Centro Ballena Azul, Valdivia, Chile; 3 Marine Mammal Laboratory, Alaska Fisheries Science Center/NOAA, 7600 Sand Point Way NE, Seattle, WA USA; 4 Marine Ecology and Telemetry Research, 2468 Camp McKenzie Tr NW, Seabeck, WA 98380 USA; 5 Cascadia Research Collective, 218 ½ 4th Ave, Olympia, WA 98502 USA; 6 Instituto Aqualie, Av. Dr. Paulo Japiassú Coelho, 714, Sala 206, Juiz de Fora, MG 36033-310 Brazil; 7 Grupo de Ecología Cuantitativa, INIBIOMA-CONICET, Universidad Nacional del Comahue, Bariloche, Argentina; 8 Marine Mammal Institute and Department of Fisheries and Wildlife, Hatfield Marine Science Center, Oregon State University, Newport, OR USA

**Keywords:** Marine biology, Conservation biology, Ecology, Ecological modelling

## Abstract

Defining priority areas and risk evaluation is of utmost relevance for endangered species` conservation. For the blue whale (*Balaenoptera musculus*), we aim to assess environmental habitat selection drivers, priority areas for conservation and overlap with vessel traffic off northern Chilean Patagonia (NCP). For this, we implemented a single-step continuous-time correlated-random-walk model which accommodates observational error and movement parameters variation in relation to oceanographic variables. Spatially explicit predictions of whales’ behavioral responses were combined with density predictions from previous species distribution models (SDM) and vessel tracking data to estimate the relative probability of vessels encountering whales and identifying areas where interaction is likely to occur. These estimations were conducted independently for the aquaculture, transport, artisanal fishery, and industrial fishery fleets operating in NCP. Blue whale movement patterns strongly agreed with SDM results, reinforcing our knowledge regarding oceanographic habitat selection drivers. By combining movement and density modeling approaches we provide a stronger support for purported priority areas for blue whale conservation and how they overlap with the main vessel traffic corridor in the NCP. The aquaculture fleet was one order of magnitude larger than any other fleet, indicating it could play a decisive role in modulating potential negative vessel-whale interactions within NCP.

## Introduction

Animal movement integrates several scales of ecological phenomena, including individual physiological state, locomotive, and navigational capabilities, and how these interact with external (environmental) factors affecting prey distribution. This has been explicitly acknowledged by theoretical approaches that place movement into a wider ecological and evolutionary framework^1–3^. Coupled with this growth in movement ecological theory, the rapid increase in animal tracking technology has allowed researchers to expand the frontiers of the questions that can be answered^4,5^. It is not surprising then, that movement approaches are being increasingly used as an ecological tool for informing conservation and management actions^6–8^. In fulfilling this goal, telemetry data have become particularly useful for oceanic species with wide-ranging life histories, for which other more traditional monitoring approaches are logistically challenging^9^.

For the endangered Eastern South Pacific (ESP) blue whale (*Balaenoptera musculus*) population, northern Chilean Patagonia (NCP) is regarded as its most important summer foraging and nursing ground^10–12^. Previous studies on blue whale occurrence and movement patterns indicated that until the onset of austral autumn/winter migration, blue whales focus most of their activities within these productive coastal waters^12–15^. However, variations in how this population utilizes this region and other areas within the ESP appear to result from changes in prevailing oceanographic conditions^16^.

Species distribution models (SDM) have shown that austral spring chlorophyll-*a* concentration, prior to the whales’ arrival, and thermal fronts are important oceanographic proxies for describing the abundance and distribution patterns of blue whales within the NCP^16^. Krill, the primary prey of blue whales^17^, can take advantage of seasonally enhanced productivity for biomass production, with some time lag linking early life-history stages (*e.g.* larval recruitment) with adult densities^17–21^. Adult krill biomass is subsequently concentrated by thermal fronts into high-density patches which blue whales prey upon^22–25^. This prey aggregation effect driven by thermal fronts could be critical for blue whales, and other large baleen whales, given their energetically costly feeding behavior^26–29^. We hypothesize that both time-lagged distribution of primary productivity and thermal front aggregating effect generates foraging conditions for blue whales within NCP. To further test predictions from this hypothesis, here we propose that individual blue whales modify their behavior within areas of high spring chlorophyll-*a* concentrations and/or thermal front occurrence. As foraging behavior cannot be directly assessed solely by inspecting tracking data, we consider area-restricted search behavior (ARS, lower velocity and less directional persistence) as a proxy for this type of behavior^30,31^.

Potential local threats affecting blue whales in NCP include collisions with vessels due to intense maritime traffic^16,32^, negative interactions with aquaculture and fisheries activities^33–35^, direct and indirect effects from poorly regulated whale-watching operations^36^, and general disturbance from noise and acoustic pollution^37^. As such, identifying priority areas for focusing conservation actions is of utmost relevance considering a population numbering the low hundreds with a very low potential biological removal from anthropogenic origin estimated at 1 individual every 1.8 years^16^ for continued growth.

Vessel collisions with cetaceans have become recognized worldwide as a significant source of anthropogenic mortality and serious injuries^38–41^. Empirical work on this issue has been conducted in a few areas and populations, mostly in the northern Hemisphere^32,39,42,43^, with little effort conducted in South America^32,44^. In most countries, unreported cases, limited monitoring and insufficiently documented incidents have precluded any accurate assessment of the true collision prevalence and trend analyses^32^.

Given the earlier results from SDMs, we considered using telemetry data as a complementary tool for improving our understanding of blue whale habitat selection process^16,17,45^ and investigating overlap with vessel traffic in NCP. In fulfilling these goals, here we provide: i) a novel fast-fitting model application for data gathered from satellite-monitored Argos tags (hereafter Argos tags), ii) model-derived spatial predictions of how whales use the area based on prevailing oceanographic conditions during the tracking period, iii) spatial estimates on the relative probability of encountering blue whales, based on the integration of movement model predictions with those of a previous SDM, and iv) spatial estimates on the relative probability of whales encountering vessels as a measure of risk for four different vessel fleets operating in NCP.

## Methods

### Study area

**Figure 1:**
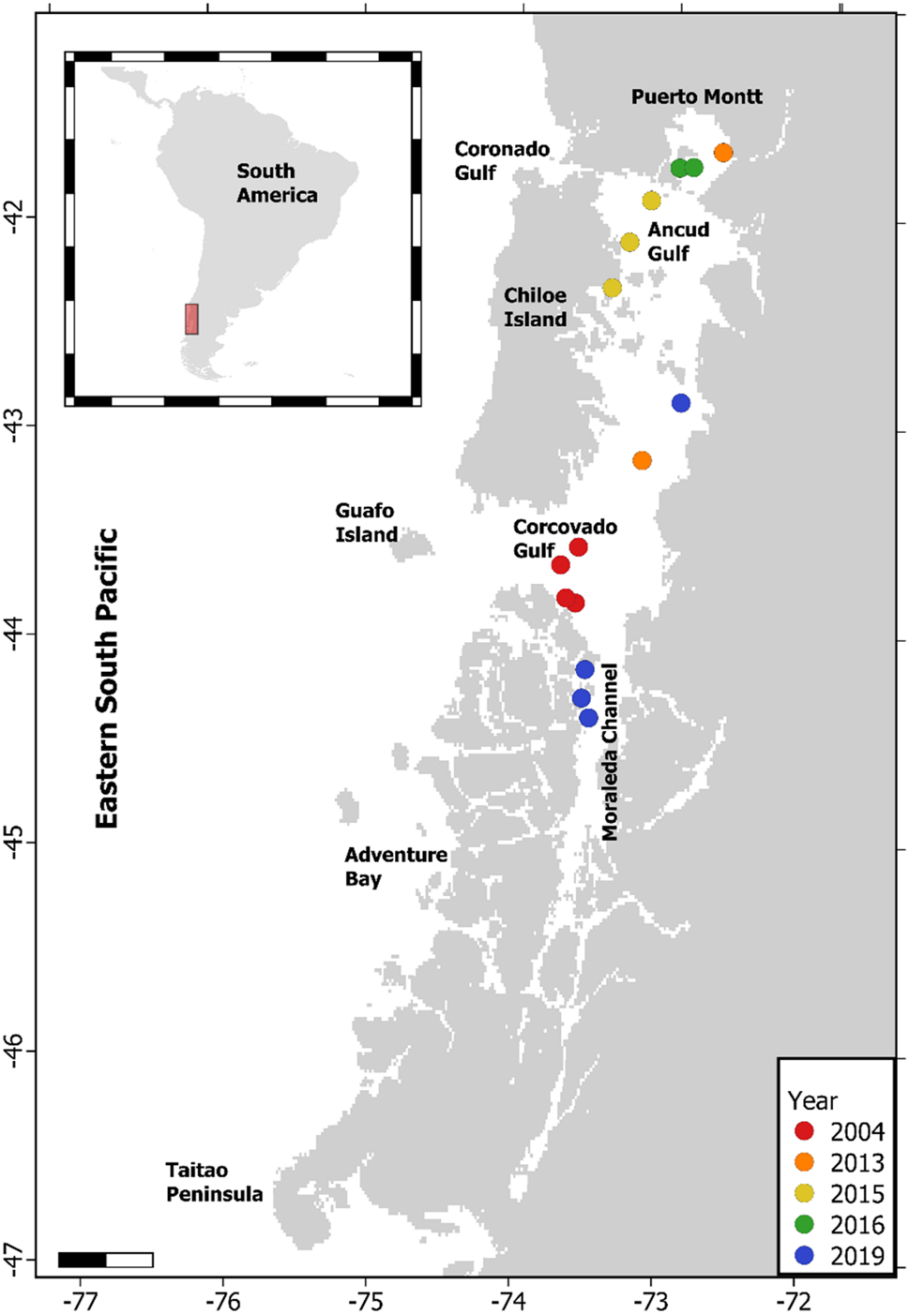
Map of the Chilean Northern Patagonia depicting relevant geographical landmarks, tagging locations and the year of each deployment. Maps were created in R ver. 4.0.2 (https://www.r-project.org) and ensembled in QGIS ver. 3.8.0 (https://www.qgis.org) for final rendering. Maps were created using data on bedrock topography from the National Centers for Environmental Information (https://maps.ngdc.noaa.gov/viewers/grid-extract/index.html). Values above 0 were considered land coverage.

The NCP (41–47°S) is characterized by an intricate array of inner passages, archipelagos, channels, and fjords enclosing roughly 12,000 km of convoluted and protected shoreline (Fig. 1). Primary biological productivity here is modulated by the mixing of sub-Antarctic waters, rich in macro-nutrients, and the abundant input of freshwater (derived from river discharges, heavy precipitation and glacier/snow melt), rich in micro-nutrients, particularly silica^46–48^. Within the NCP, several micro-basins have been described, some of them having particularly high seasonal primary and secondary production^46–50^, providing resources that upper-trophic level species rely on^12,17,50–54^. The area also hosts one of the largest salmon aquaculture industries in the world, among other anthropogenic activities that negatively affects local biodiversity^33,34,55^.

### Tagging and telemetry data

Argos tags were deployed on 15 blue whales during the austral summer and early autumn at their summering grounds off the NCP (Fig. 1), following procedures described elsewhere^14^. Briefly, whales were tagged in waters of Corcovado Gulf during February 2004 (n = 4), and the Chiloe Inner Sea during late March and early April 2013 (n = 2), 2015 (n = 3), 2016 (n = 2) and 2019 (n = 4). Tags were deployed using a custom-modified compressed-air line-thrower (ARTS/RN, Restech Norway^56^) set at pressures ranging between 10 and 14 bar. Several models of custom-designed fully implantable satellite tags were used, including: ST-15 [n = 4], manufactured by Telonics (Mesa, Arizona, USA), SPOT5 [n = 3], SPOT6 [n = 4], and MK10 [n = 4], manufactured by Wildlife Computers (Redmond, Washington, USA).

Raw Argos data included locations within NCP and outside the area after the onset of migratory movement. Because we were concerned with understanding movement patterns within the NCP, we applied a cut-off point of 24 h prior to a clear sign of migration was observed. This subset of the data was filtered using the R package “argosfilter”^57^ removing relocations that comprised velocities exceeding 3 m s^−1^, this upper limit was defined based on previous maximum speed assessments for this population^14^.

### Oceanographic covariates

Chlorophyll-*a* and sea surface temperature (SST) data were extracted using R package “rerddapXtracto”^58^, which accesses the ERDDAP server at the NOAA/SWFSC Environmental Research Division. Chlorophyll-*a* data corresponded to satellite level-3 images from the Moderate Resolution Imaging Spectroradiometer (MODIS) sensor onboard the Aqua satellite (Dataset ID: erdMH1chlamday), corresponding to monthly averages in a grid size of 4.64 × 4.64 km. Distance to areas of high chlorophyll-*a* concentration during spring (DAHCC), defined as the distance to polygons enclosing areas with an average chlorophyll-*a* concentration equal or higher than 5 mg/m^3^ during austral spring months (September, October, November), was the best explanatory variable in a SDM applied to line-transect survey data for blue whales in NCP^16^. Here we used the same procedure to construct this covariate but used the 95^th^ percentile of each year´s concentrations distribution within the study area as the cut-off point for defining areas of high chlorophyll-*a* concentration. This was preferred because whales might select areas with the highest productivity regardless of their absolute values. Maps for DAHCC were created for each year where telemetry data were available, and their values were log transformed to reduce data overdispersion before their use in the models.

For SST, data corresponded to daily averages of level-4 satellite images derived from the Multi-Scale Ultra-High Resolution (MUR) SST Analysis database (Dataset ID: jplMURSST41). MUR-SST maps merge data from different satellites, combined with in-situ measurements, using the Multi-Resolution Variational Analysis statistical interpolation^59^, in a grid size of 0.01 × 0.01 degrees (ca. 1 km^2^). From MUR-SST maps, thermal gradients maps were generated for each day that whale locations were available using the R package “grec” v. 1.3.0^60^ with the Contextual Median Filter algorithm^61^ as the method for calculating gradients. MUR-SST and thermal gradients maps were used to extract the associated covariate values for each whale location.

### Vessel traffic data

To characterize vessel traffic patterns in the area, daily vessel tracking information (time-stamped GPS locations for individualized vessels) was obtained from the Chilean National Fisheries and Aquaculture Service (SERNAPESCA), available at www.sernapesca.cl. This database was released by the Chilean government during 2020 and comprises data involving the industrial and artisanal fisheries, aquaculture, and transport fleets, from March 2019 to present (updated daily). According to Chilean legislation it is mandatory for these fleets to provide tracking information to SERNAPESCA, except for artisanal fishing vessels smaller than 15 m and also for those smaller than 12 m in the case of artisanal purse seiners (www.bcn.cl). Artisanal fishing fleet comprises vessels up to 18 m in length and less than 80 cubic meters of storage capacity; above these metrics fishing vessels are considered part of the industrial fishing fleet. The transport fleet comprises vessels with no size limitations, engaged solely in the transportation of fishery resources. The aquaculture fleet is the most diverse one, considering its different operations (e.g. staff commuting, live and processed resource transportation, and supplies and infrastructure movement) with vessel sizes ranging from 5 to 100 m.

All procedures described next were conducted independently for each fleet during data analyses. We used an 8 × 8 km grid to calculate vessel density (VD_*i*_) for each grid-cell *i*. Vessel data are provided daily, with data gaps occurring for some days. Therefore, VD_*i*_ was calculated by summing the daily number of unique vessels crossing each grid-cell *i* in a month divided by the total number of days with available data (range: 25–31 days). This procedure was conducted for austral summer and austral autumn months (March-June of 2019 and January-June of 2020) and then averaged into a single layer. Potential large differences in traffic patterns between these months were visually inspected through plots, which can be found as Supplementary Figures S1–S4 online. Data from austral winter and austral spring months were not used as most of the blue whale population is absent from the study area during these months^13,14^.

### Modeling approach

Telemetry data analysis has motivated the development and increasing use of various state-space modeling (SSM) approaches, which deal with path reconstruction and complex latent behavioral states^30,31,62,63^. Most practical applications of SSM, however, are computationally intensive and therefore require a long time for fitting them. Recently, SSM has been implemented via Template Model Builder (TMB), a R package that relies on the Laplace approximation combined with automatic differentiation to fast-fit models with latent variables^64–66^. Based on “TMB” tools, we fitted a continuous-time correlated-random-walk model (CTCRW) which estimates two state variables, velocity and true locations from error-prone observed locations, and two parameters, *β* controlling autocorrelation in directionality and velocity and *σ* controlling the overall variability in velocity^62^. Variances for modelling error in locations were derived from the Argos error ellipse^67^. As the error ellipses data were not available for tags deployed in 2004, we calculated the mean error ellipse for all location classes in the newer tags (2013–2019) and assigned these values to the corresponding location classes for tags deployed in 2004.

The original version of this model (with no behavioral variation) was fitted to obtain estimates of the true locations in whale’s paths and used these to extract the corresponding covariate values from DAHCC, SST and thermal gradients rasters. The mean of the covariate values within a 3 km radius from each estimated location was used to partially account for uncertainty in covariate data arising from observation error. This error radius corresponded to twice the known error for Argos location classes 3, 2 and 1^67^. Covariate data were standardized, and missing values were filled with zeros, which correspond to the mean in standardized variables. This only affected 6 whales (ID#s 1,6,7,10,11 and 12), it was restricted to SST and thermal gradient data, and except for one whale never exceeded more than 2.7% of the data (with ID#7 at 10.4% of the data). We modified the original version of the CTCRW by allowing *β*_*t*_and *σ*_*t*_to be random variables that vary in time as a function of environmental covariates.

$${log(\sigma }_{t})\sim \mathrm{Normal}({\mu }_{1,t},{\varepsilon }_{1})$$

$${\mu }_{1,t}=A0+A{X}_{t}$$

$$log({\beta }_{t})\sim \mathrm{Normal}({\mu }_{2,t},{\varepsilon }_{2})$$

$${\mu }_{2,t}=B0+B{X}_{t}$$

where *B0* and *A0* are intercepts, *A* and *B* are vectors of slopes, *X*_*t*_ is the corresponding design matrix holding the standardized covariates, and ε_1_ and ε_2_ correspond to standard deviations. In every case, the estimated standard deviation ε_2_ for *β*_*t*_ was extremely small and presented exceptionally large standard errors; therefore, instead of trying to estimate this parameter, we fixed it at 0.01. In cases where no covariate presented a significant effect on *β*_*t*_ this variable was reduced to a single parameter *β*, which was estimated. Estimated values of *β* larger than 4 produce persistence values lower than 0.05 h, indicating that at very short time differences velocity and location are poorly correlated with previous values. Therefore, in cases where model estimates for *β* were higher than 4 (ID#s 5 and 10) *β* was fixed at 4 indicating overall poorly autocorrelated movement patterns.

Our modelling approach allowed us to quantify the influence of environmental covariates on *β*_*t*_ and *σ*_*t*_ , with higher values of *σ*_*t*_ indicating higher velocities and higher values of *β*_*t*_ indicating lower directional persistence, which might be expressed as *p*_*t*_ = 3/*β*_*t*_ in units of time^62^. As no discrete behavioral states were explicitly included in our model, we defined behavioral states as post hoc categories based on *p*_*t*_ and *σ*_*t*_values and their medians. The expected ARS state (slower and less persistent movement) was defined for locations jointly holding values of *p*_*t*_ and *σ*_*t*_below their medians and the opposite was defined as transit state. The other two logical combinations (high *p*_*t*_ with low *σ*_*t*_ and low *p*_*t*_ with high *σ*_*t*_) were also provided and their interpretation is further discussed below. We also calculated ${\nu }_{t}=\frac{\sqrt{\pi }*{\sigma }_{t} }{\sqrt{{\beta }_{t}}*2}$, which corresponds to long-term velocity^68^. This variable is a function of both *σ*_*t*_and *β*_*t*_(or *p*_*t*_), and hence higher *ν*_*t*_ can be obtained by either increasing *σ*_*t*_or reducing *β*_*t*._ As *ν*_*t*_ is a function of both *σ*_*t*_ and *β*_*t*_, we considered it as a proxy for the ARS-transit continuum, with higher values of *ν*_*t*_representing more transit-like behavior. Expected responses of *ν*_*t*_to covariate variation were inspected through prediction curves.

Finally, model results were used to generate spatial predictions for *ν*_*i*_for each grid-cell *i* using a 1 × 1 km grid. These predictions indicate the expected behavioral responses for whales traversing areas not necessarily visited during the tracking period. Predictive layers were generated for individual whales and averaged across individuals for depicting an overall pattern.

### Integrating movement and species distribution models

Results from a previous SDM were used for assessing spatial overlap between blue whale distribution and marine traffic. Briefly, this model consisted of a Bayesian binomial N-mixture model used to model blue whale groups counts in line-transect data (2009, 2012 and 2014), using distance sampling techniques and oceanographic covariate data^16^. Using an 8 × 8 km grid spatial predictions of blue whale density at each grid-cell *i* (N_*i*_) were generated for eight years (2009–2016) and averaged into a single layer. To integrate outputs from movement models and SDM the relative probability of encountering a whale (RPEW) was calculated as follows

$${RPEW}_{i}=\frac{{N}_{i} \frac{1}{{\nu }_{i}}}{\sum_{i=1}^{n}({N}_{i} \frac{1}{{\nu }_{i}})}.$$

RPEW_*i*_ assumes that the probability of encountering whales increases with predicted density^39,69^. Here we consider behavior might also be part of this function as slow and less persistent movement (ARS) will result in more time spent (1/*ν*_*i*_) allocated to each grid-cell *i* relative to all other grid cells *n*. As N_*i*_, had a spatial resolution of 8 × 8 km, we resampled the *ν*_*i*_ grid to match the coarser grid resolution prior to any calculation, using the mean of aggregated grid-cells.

### Defining spatial overlap with marine traffic

A quantitative measure of risk associated to vessel traffic can be considered as a monotonic function of the number of vessels and the probability of encountering a whale^39,70^. As described above, the relative amount of time allocated to each grid-cell can be obtained from 1/*ν*_*i*_. Therefore, as a measure of risk we calculated the relative probability of vessel encountering whale (RPVEW)^39,69^ by combining N_*i*_, *ν*_*i*_ and VD_*i*_ as follows.

$${RPVEW}_{i}=\frac{{Pw}_{i} {{Pt}_{i} Pv}_{i}}{\sum_{i=1}^{n}{(Pw}_{i} {Pt}_{i}{ Pv}_{i})}$$

where ${Pw}_{i}=\frac{{N}_{i}}{\sum_{i=1}^{n}({N}_{i})}$ corresponds to the probability of observing a whale within each grid-cell *i* relative to all other grid cells *n*, ${Pt}_{i}=\frac{\frac{1}{{\nu }_{i}}}{\sum_{i=1}^{n}(\frac{1}{{\nu }_{i}})}$ corresponds to the time allocated to each grid-cell *i* relative to all other grid cells *n*, and ${Pv}_{i}=\frac{{VD}_{i}}{\sum_{i=1}^{n}{(VD}_{i})}$ corresponds to the observed number of vessels within grid-cell *i* relative to all other grid cells *n*. fleets. Finally, to generate quantitative estimates on the degree of overlap between blue whale distribution and vessel traffic we used the Shoener's D and Warren's I similarity statistics^71^. These statistics range from 0, indicating no overlap, to 1, indicating distributions are identical. To use these statistics, the variables N_*i*_ times 1/*ν*_*i*_ and VD_i_ were rescaled to range between 0 and 1 and inputted to the nicheOverlap function from the R package *dismo*^72,73^. A schematic representation of our workflow can be found as a Supplementary Figure S5 online.

### Statement of approval

The tagging methods employed in this study were approved by the Institutional Animal Care and Use Committee of the National Marine Mammal Laboratory of the Alaska Fisheries Science Center, National Marine Fisheries Service, U.S. National Oceanic and Atmospheric Administration. All methods employed in this study were carried out in accordance with guidelines from Subsecretaría de Pesca y Acuicultura (SUBPESCA), which provided full authorization to undertake this research through resolution #2267 of the Chilean Ministry of Economy and Tourism.

## Results

**Figure 2:**
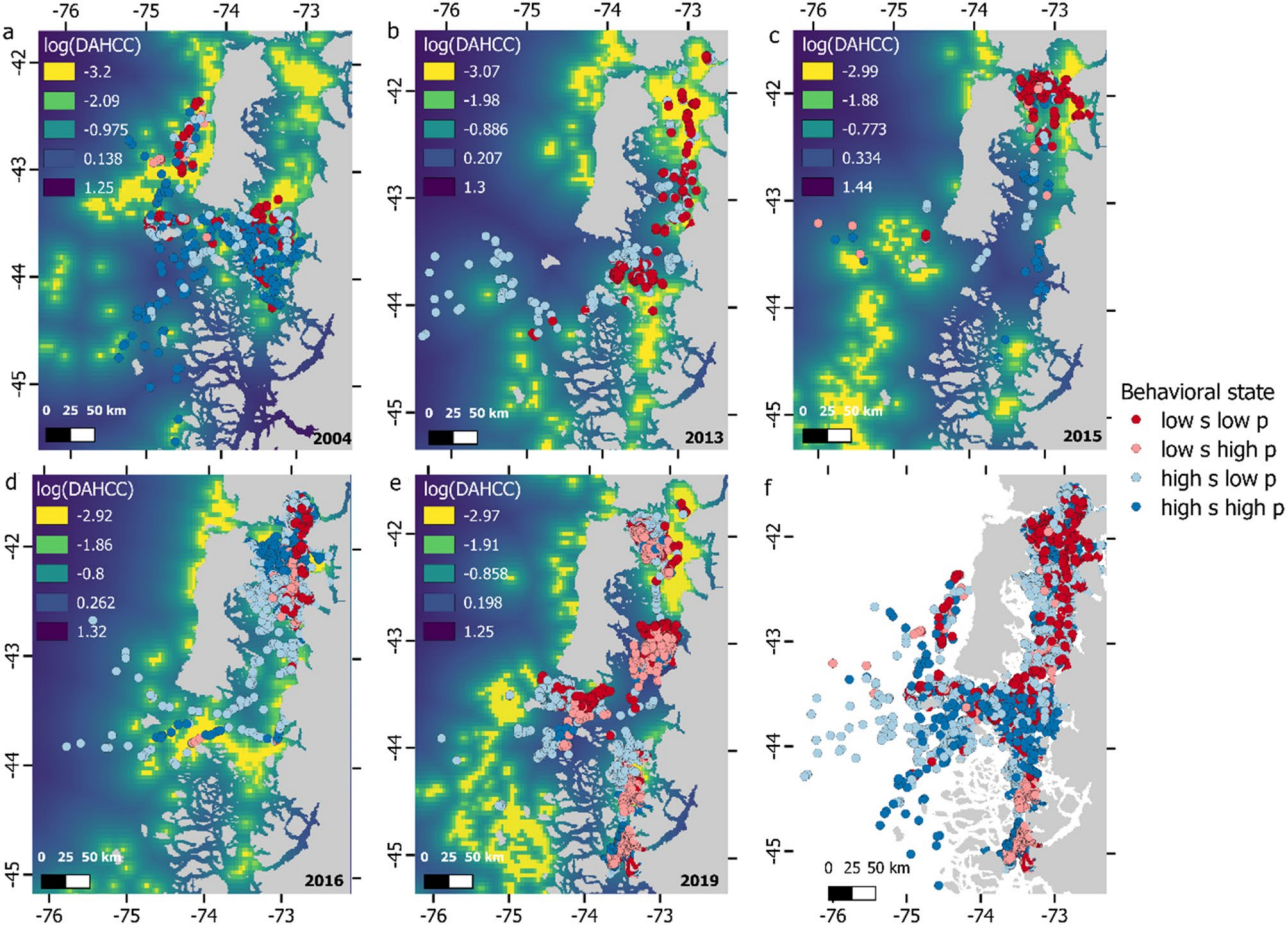
Behavioral variation for tagged whales. Panels (**a**–**e**) summarize results for 2004, 2013, 2015, 2016 and 2019, respectively and panel f combines all tracks. Red to blue four-color ramp indicates the percentile to which each location belongs regarding variation in σ_t_ and 3/β_t_ (persistence). By using the medians, the four possible combinations are presented as a posteriori behavioral state identification. Locations jointly holding values of σ_t_ and 3/β_t_ below their medians across all whales (low s and low p) can be considered ARS behavior, while the opposite (high s and high p) can be considered transit. Blue (far) to yellow (close) color ramp in the background indicates variation in standardized distance to areas of high chlorophyll concentration (DAHCC) in log scale, which was the most consistent covariate shaping blue whale movement patterns in this study. Data layers (including maps) were created in R ver. 4.0.2 (www.r-project.org) and ensembled in QGIS ver. 3.8.0 (www.qgis.org) for final rendering. Maps were created using data on bedrock topography from the National Centers for Environmental Information (https://maps.ngdc.noaa.gov/viewers/grid-extract/index.html). Values above 0 were considered land coverage.

Tracking duration for instrumented whales while within the study area ranged from 8.1 to 105 days (mean = 52.03, sd = 29.3, median = 48.7), yielding tracks that ranged from 49 to 1,728 locations (mean = 460.27, sd = 582.36, median = 140) used for modelling (after filtering, Table 1). In general, whales tended to remain in very localized coastal areas, where high productivity occurs during each austral spring (Fig. 2). No instrumented individuals departed from NCP until the onset of austral autumn–winter months (April-July)^14^. Pearson correlation analyses showed that none of the used covariates were strongly correlated (*r* < 0.5, *p* < 0.01). Except for one instrumented whale (ID#12), all animals showed a significant positive correlation between *σ*_*t*_ and DAHCC, six animals showed a significant negative correlation between *σ*_*t*_ and thermal gradients (Table 1). These results imply a clear pattern of whales reducing their velocities near areas that were highly productive during spring each year and/or where higher thermal gradients occur. The relationship with SST was less clear as three individuals showed a significant negative correlation and five a significant positive one (Table 1).

**Table 1:** Parameters estimations for each individual whale (log scale). Individual ID, tag deploying date, number of available locations (locs) and tracking days are provided for each whale. Missing values for parameters estimating variation in log(β) represent the cases where this was considered as a single parameter instead of a random variable. For each covariate estimates, standard errors (SE), and *p* values are provided for each parameter.

ID	Date	locs	Tracking days	log(σ)											log(sd)	
				Intercept		DAHCC			SST			Thermal gradient				
				Estimate	SE	Estimate	SE	*p* value	Estimate	SE	*p* value	Estimate	SE	*p* value	Estimate	SE
1	2004-02-13	128	75	12.64	0.16	**1.76**	**0.11**	** < 0.001**	** − 1.42**	**0.3**	** < 0.001**	0.12	0.14	0.39	− 9.51	1010
2	2004-02-19	119	62.7	9.6	0.24	**0.79**	**0.17**	** < 0.001**	− 0.09	0.15	0.5	− 0.08	0.12	0.5	− 1.24	0.07
3	2004-02-12	110	36	9.09	0.16	**0.43**	**0.13**	** < 0.001**	** − 0.22**	**0.08**	** < 0.01**	− 0.05	0.11	0.6	− 1.45	0.11
4	2004-02-18	140	47.6	8.74	0.17	**0.38**	**0.1**	** < 0.001**	** − 0.27**	**0.09**	** < 0.01**	− 0.01	0.08	0.95	− 1.12	0.05
5	2013-04-01	304	45.1	8.86	0.08	**0.29**	**0.06**	** < 0.001**	0.1	0.06	0.08	0.08	0.06	0.23	− 0.93	0.02
6	2013-04-23	68	8.1	12.9	0.43	**2.2**	**0.3**	** < 0.001**	0.45	0.24	0.06	0.53	0.42	0.2	− 1.8	0.34
7	2015-04-17	314	48.7	8.7	0.12	**0.61**	**0.08**	** < 0.001**	− 0.04	0.06	0.46	− 0.8	0.06	0.19	− 1.17	0.03
8	2015-04-13	130	21.6	12.35	0.3	**2.97**	**0.18**	** < 0.001**	**1.69**	**0.18**	** < 0.001**	− **0.44**	**0.13**	** < 0.001**	− 1.6	0.12
9	2015-04-09	49	17.1	9.5	0.28	**0.56**	**0.21**	** < 0.01**	**0.3**	**0.13**	** < 0.05**	− **1.28**	**0.41**	** < 0.01**	− 8.5	173
10	2016-04-04	129	20.1	10.61	0.23	**1.16**	**0.10**	** < 0.001**	− 0.16	0.1	0.11	0.01	0.1	0.9	− 1.15	0.06
11	2016-04-05	1710	105.2	8.85	0.07	**0.21**	**0.03**	** < 0.001**	**0.09**	**0.03**	** < 0.01**	− **0.09**	**0.03**	** < 0.05**	− 1.62	0.02
12	2019-02-03	649	100	8.19	0.05	0.05	0.11	0.6	**0.26**	**0.04**	** < 0.001**	− **0.11**	**0.05**	** < 0.05**	− 1.8	0.06
13	2019-02-06	1122	71.4	8.3	0.07	**0.19**	**0.04**	** < 0.001**	**0.31**	**0.03**	** < 0.001**	− **0.08**	**0.03**	** < 0.05**	− 10.1	291
14	2019-04-27	204	49.1	8.04	0.07	**0.71**	**0.30**	** < 0.05**	− 0.02	0.07	0.72	0.06	0.07	0.4	− 1.02	0.04
15	2019-02-09	1728	72.7	9.04	0.08	**0.38**	**0.03**	** < 0.001**	− 0.02	0.03	0.50	− **0.08**	**0.03**	** < 0.01**	− 1.76	0.03

ID	Date	locs	Tracking days	log(β)												
				Intercept		DAHCC			SST			Thermal gradient				
				Estimate	SE	Estimate	SE	*p* value	Estimate	SE	*p* value	Estimate	SE	*p* value		
1	2004-02-13	128	75	− 1.23	0.6	** − 0.53**	**0.19**	** < 0.01**	**6.79**	**0.43**	** < 0.001**	**0.79**	**0.3**	** < 0.01**		
2	2004-02-19	119	62.7	1.86	2.28	− 0.79	1.13	0.49	**3.22**	**1.25**	** < 0.01**	0.08	0.58	0.9		
3	2004-02-12	110	36	0.82	0.98	0.22	0.46	0.64	− 0.11	0.49	0.83	1.005	0.63	0.09		
4	2004-02-18	140	47.6	0.64	0.99	− **1.29**	**0.42**	** < 0.01**	**1.31**	**0.56**	** < 0.05**	− 0.09	0.3	0.76		
5	2013-04-01	304	45.1	4	**–**	**–**	**–**	**–**	**–**	**–**	**–**	**–**	**–**	**–**		
6	2013-04-23	68	8.1	3.5	1.12	**–**	**–**	**–**	**–**	**–**	**–**	**–**	**–**	**–**		
7	2015-04-17	314	48.7	3.34	0.74	0.08	0.32	0.8	**1.46**	**0.33**	** < 0.001**	− 0.25	0.24	0.31		
8	2015-04-13	130	21.6	3.36	1.12	− **1.00**	**0.31**	** < 0.001**	− **3.33**	**0.39**	** < 0.001**	**4.86**	**0.48**	** < 0.001**		
9	2015-04-09	49	17.1	− 1.39	0.37	0.36	0.35	0.3	**0.93**	**0.17**	** < 0.001**	− 0.36	0.38	0.35		
10	2016-04-04	129	20.1	4	**–**	**–**	**–**	**–**	**–**	**–**	**–**	**–**	**–**	**–**		
11	2016-04-05	1710	105.2	1.88	0.17	**0.49**	**0.07**	** < 0.001**	− **0.37**	**0.13**	** < 0.01**	− 0.15	0.12	0.21		
12	2019-02-03	649	100	1.31	0.25	− 1.54	1.37	0.26	0.51	0.33	0.13	− 0.09	0.36	0.8		
13	2019-02-06	1122	71.4	0.84	0.29	0.22	0.13	0.10	− 0.17	0.11	0.10	** − 0.20**	**0.09**	** < 0.05**		
14	2019-04-27	204	49.1	3.57	0.9	− 5.7	3.58	0.11	0.54	0.65	0.41	0.85	0.68	0.21		
15	2019-02-09	1728	72.7	2.04	0.2	**0.4**	**0.08**	** < 0.001**	0.05	0.15	0.74	− 0.15	0.1	0.13		

Bold value indicates statistically significance *p* < 0.05

Regarding correlations between *β*_*t*_ and environmental covariates, it was expected that whenever significant, they would present the opposite sign of those that were significant regarding *σ*_*t*_, rendering a continuum between ARS and transit behavior. This was the case for three individuals with respect to DAHCC (ID#s1, 4 and 8), four individuals with respect to SST (ID#s 1, 4, 8 and 11) and one individual with respect to thermal gradients (ID#8, Table 1). Interestingly, two individuals showed the same signal in their correlation between DAHCC and *β*_*t*_, as well as, between DAHCC and *σ*_*t*_ (ID# 11 and 15). The same occurred for one individual regarding SST (ID#9) and one individual regarding thermal gradients (ID#13, Table 1).

Post hoc definition of behavioral states showed the expected occurrence of both transit and ARS behavior. However, it also showed the occurrence of intermediate behavioral states at locations associated with low speed and high persistence and vice versa (Fig. 2). These types of intermediate behaviors were more predominant in individuals tagged in 2016 and 2019.

**Figure 3:**
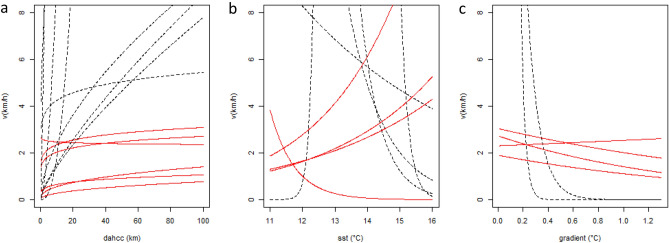
Prediction curves indicate expected variation in long-term velocity (*ν*_*t*_) in relation to environmental covariates, (**a**) distance to areas of high chlorophyll concentration (DAHCC) in log scale, (**b**) sea surface temperature (SST) and c) thermal gradients. Red lines indicate predictions for whales exhibiting more than 200 locations (ID#s 5, 7, 11, 12, 13, 14 and 15) and black lines correspond to those with less locations available.

**Figure 4:**
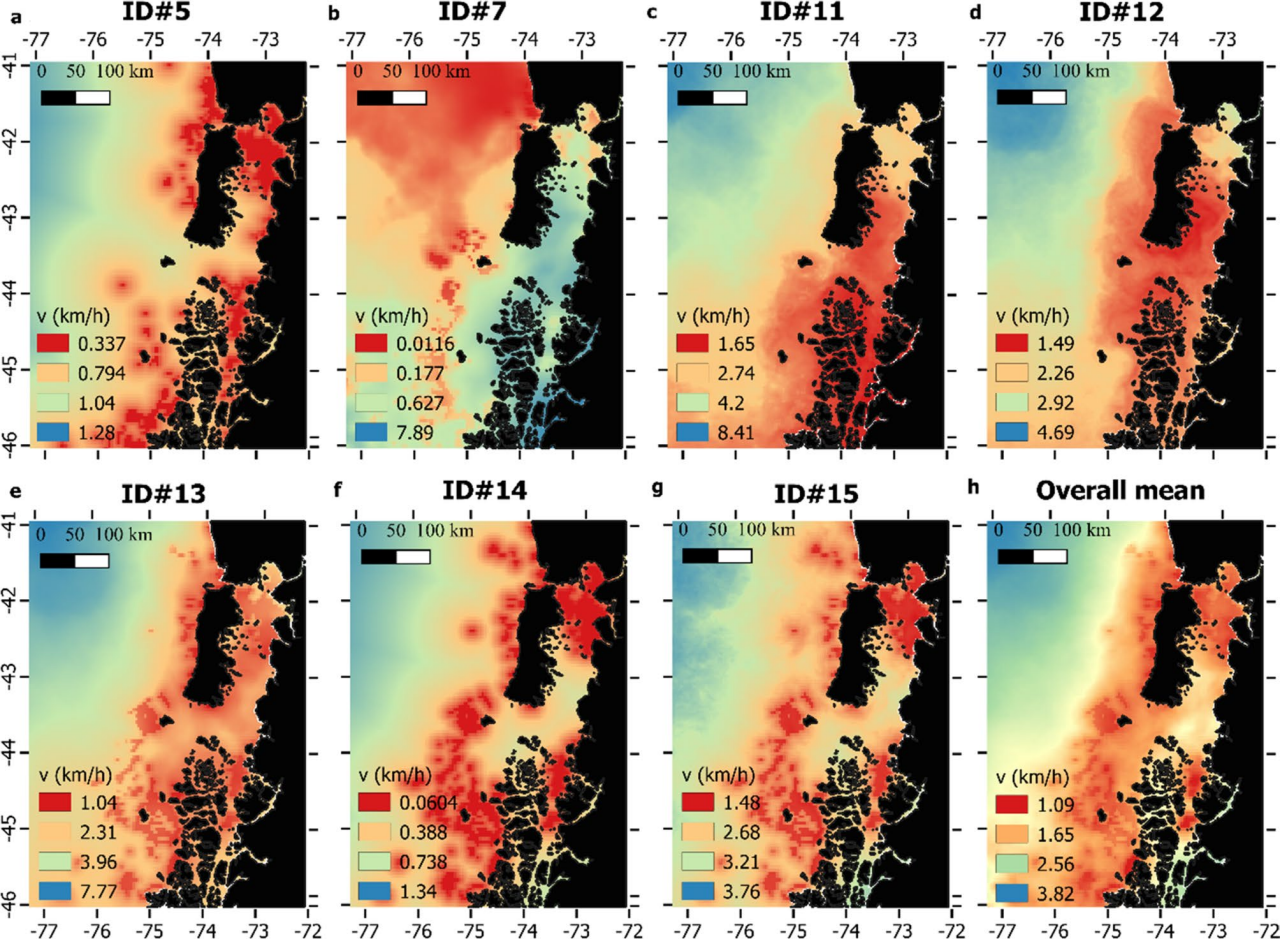
Spatial predictions of expected long-term velocity (*ν*_*t*_) responses in the entire study area, for every instrumented whale with more than 200 locations (panels **a**–**g**). The bottom right panel (h) shows the overall mean for all seven individuals. Data layers (including maps) were created in R ver. 4.0.2 (www.r-project.org) and ensembled in QGIS ver. 3.8.0 (www.qgis.org) for final rendering. Maps were created using data on bedrock topography from the National Centers for Environmental Information (https://maps.ngdc.noaa.gov/viewers/grid-extract/index.html). Values above 0 were considered land coverage.

**Figure 5:**
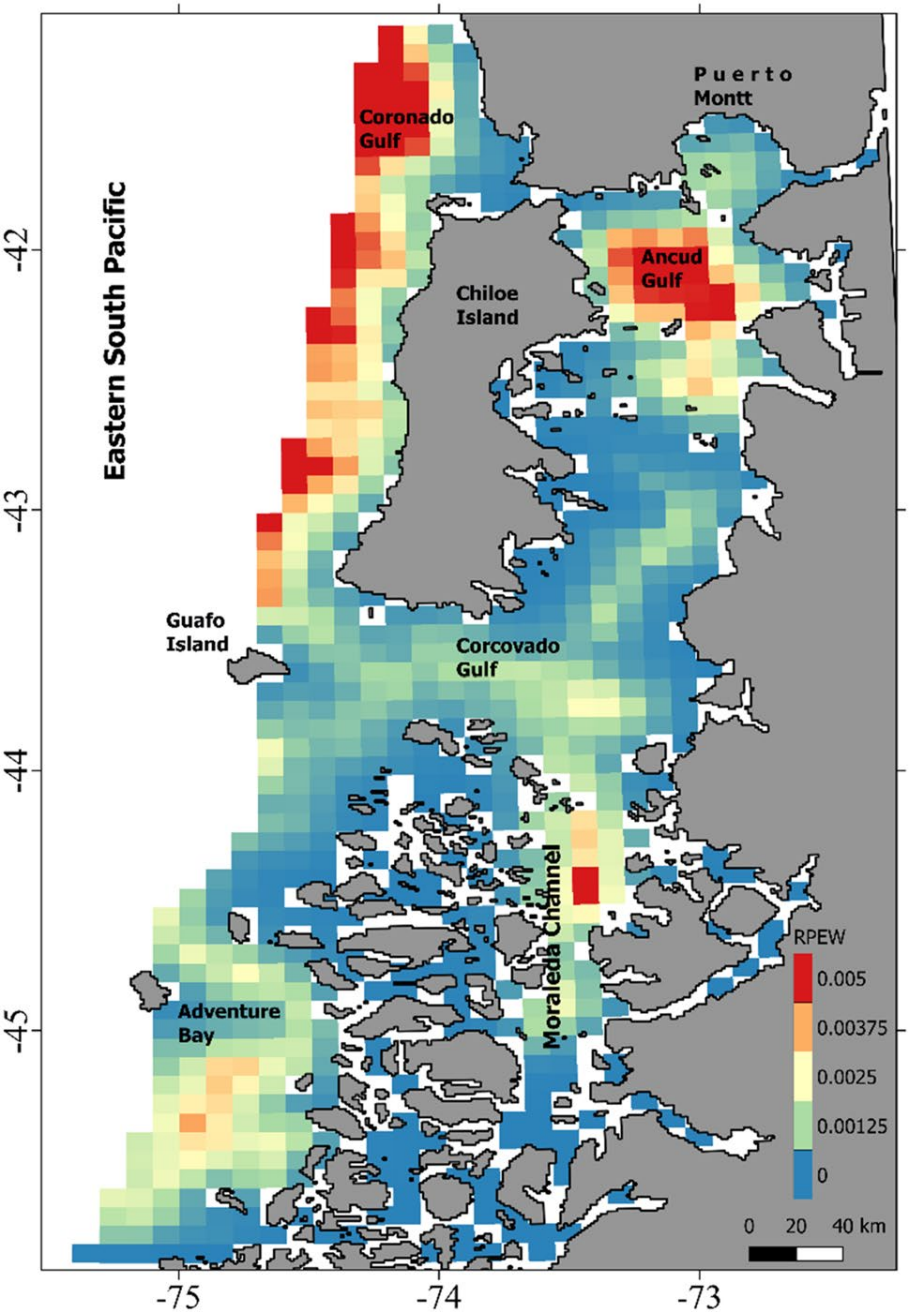
Relative probability of encountering a blue whale (RPEW). This integrates the output of the movements and species distribution models for areas within 25 km from shore. Data layers (including the map) were created in R ver. 4.0.2 (www.r-project.org) and ensembled in QGIS ver. 3.8.0 (www.qgis.org) for final rendering. Map was created using data on bedrock topography from the National Centers for Environmental Information (https://maps.ngdc.noaa.gov/viewers/grid-extract/index.html). Values above 0 were considered land coverage.

Prediction curves for *ν*_*t*_ based on covariate variation provided unrealistic predictions for individuals for which a relatively small number of locations were available (< 200 locations, Fig. 3). For this reason, we only generated spatial predictions of *ν*_*t*_ (Fig. 4) for individuals having tracks with more than 200 locations (ID#s 5, 7, 11, 12, 13, 14 and 15). Interindividual variation was observed regarding absolute values for *ν*_*t*_, indicating that some whales moved, in general, faster and in a more persistent manner (Fig. 4 b,c,e) than others, and also in terms of where their lowest values (ARS behavior) were expected. Despite this individual variation, some areas were consistently depicted as having the lowest values for *ν*_*t*_, which are highlighted when the spatial predictions for these seven whales were averaged into an overall mean (Fig. 4h). Spatial predictions on RPEW highlighted areas of aggregation for blue whales in NCP, mainly located in the western part of Chiloe Island, Ancud Gulf, Adventure Bay and northern Moraleda Channel (Fig. 5).

**Figure 6:**
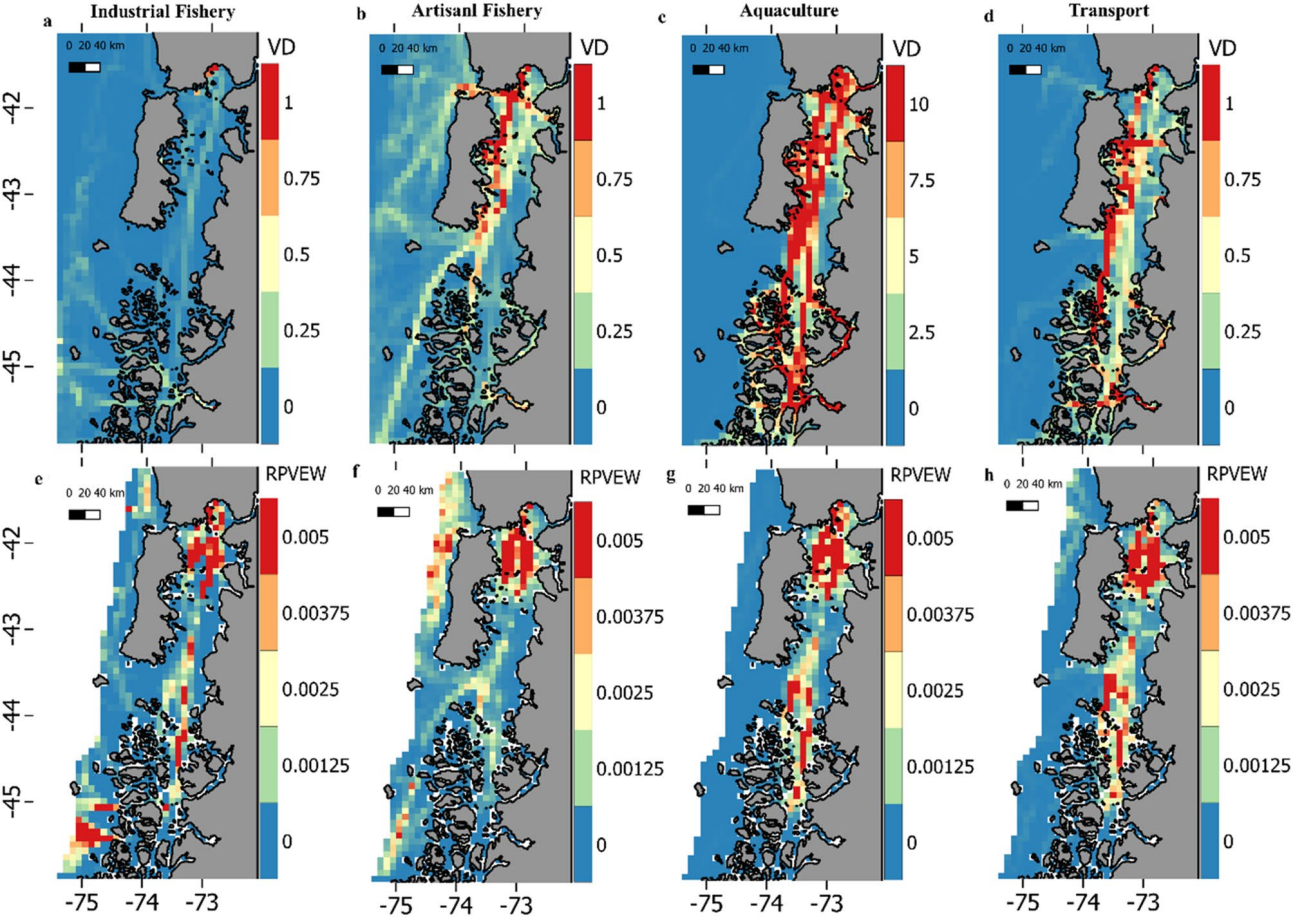
Top panels show vessel density (VD) as the mean number of vessels visiting each 8 × 8 km grid-cell per day, for the industrial fishery (**a**), artisanal fishery (**b**), aquaculture (**c**) and transport (**d**) fleets. Note the large difference in color bar increments for the aquaculture fleet. Bottom panels show the relative probability of vessel encountering whale (RPVEW) for the industrial fishery (**e**), artisanal fishery (**f**), aquaculture (**g**) and transport (**h**) fleets. The data of the different fleets are provided by the Chilean national services of fisheries and aquaculture, (SERNAPESCA) and are freely available at www.sernapesca.cl. Data layers (including maps) were created in R ver. 4.0.2 (www.r-project.org) and ensembled in QGIS ver. 3.8.0 (www.qgis.org) for final rendering. Maps were created using data on bedrock topography from the National Centers for Environmental Information (https://maps.ngdc.noaa.gov/viewers/grid-extract/index.html). Values above 0 were considered land coverage.

VD absolute values were highest for the aquaculture fleet (range:0–78.4) followed by artisanal fishery (0–13.9), transport (range: 0–8) and industrial fishery (range: 0–1.9) fleets. The number of active vessels per day was highest for the aquaculture fleet (range: 602–729), followed by the artisanal fishery (range: 37–76), transport (range: 6–57) and industrial fishery (range: 1–13) fleets. Although the four fleets studied here showed spatial variation on RPVEW, all of them coincided in a high probability of whales interacting with vessels throughout the Chiloe inner sea (Fig. 6). Among the four fleets studied the artisanal fishing fleet showed the highest overlap with blue whale distribution patterns (D = 0.34; I = 0.64). The industrial fishery (D = 0.28; I = 0.48), aquaculture (D = 0.24; I = 0.46) and transport (D = 0.23; I = 0.45) fleets showed similar lower overlap (Fig. 6).

## Discussion

### Blue whale habitat selection and priority areas for conservation

Understanding the environmental drivers of blue whale habitat selection^16,17^ is paramount for defining priority areas for its conservation and developing recommendations for marine spatial planning^11,74^. In pursuing this goal, our setting combined previous SDM fit to line-transect data with a movement model fit to telemetry data in a complementary manner. Telemetry data supports the spatial pertinence of previously defined areas for assessing blue whale abundance and distribution patterns through ship-borne surveys. Although, some whales performed brief excursions to adjacent offshore waters, they tended to remain within the NCP coastal areas during most of the tracking time, which in two cases extended for up to 3 months (Table 1). Potential caveats to this approach include tagging location bias (*i.e.* only performed in coastal waters, Fig. 1) and sampling size, which should be overcome through the ongoing tagging program.

Previous SDM^16^ showed that spring productivity and, secondarily, thermal fronts were important covariates for predicting blue whale densities. Results here show that the same covariates selected by SDM are important for understanding blue whale’s movement patterns. As with the aforementioned SDM, DAHCC was the most prevalent covariate retained in our models, which combined with thermal gradients, displayed an unequivocal pattern in their correlation with *σ*_*t*_. This is, whales tended to reduce their velocity near areas of high primary productivity that had occurred during austral spring and where strong thermal gradients take place (Table 1, Fig. 3a). As with many other large whale species, worldwide abundance and distribution patterns of blue whales have been linked to predictable highly and seasonally productive waters associated to high chlorophyll-*a*, among other proxies for enhanced productivity^19,20,24,75–78^. Nevertheless, as blue whales feed almost exclusively on krill, temporal lags are expected to occur between seasonally high primary productivity, euphausiids early life-history stage processes (*e.g.* larval recruitment), the peak in adult euphausiid densities and the peak in whale abundance^17,20,78^. Refining our understanding of how temporal lags relate chlorophyll-*a* to euphausiid spatial patterns and then to blue whale distribution remains a pending task^79,80^, especially considering that euphausiid spatial ecology in the NCP is poorly understood^49,81^.

Although spring chlorophyll-*a* appears to be a suitable general proxy for blue whale prey availability in the NCP, whales are expected to respond in a much more complex manner to environmental heterogeneity. Previously, blue whale density in the NCP was found to be higher near areas of thermal front recurrence^16^. By using telemetry data, we were able to refine the assessment scale and test whether blue whales responded to daily changes in thermal gradients. Despite the relatively coarse resolution of Argos data, we were able to find evidence for behavioral response in six whales while traversing thermal gradients of less than 1 °C (Fig. 3c). This may even represent an underestimation given the reported response of blue whales to gradients as low as 0.03 °C^82^. Thus, our results provide additional support on the relevance of coarse to meso-scale thermal gradients when shaping marine predator distribution^16,23,82,83^. The underlying mechanism for this pattern, however, is not clear, as thermal fronts might be responsible for increasing prey availability by boosting local productivity and/or by aggregating prey patches^22–25,83,84^. Within the NCP, both processes are likely to be tightly coupled. The influence of fresh waters rich in silicic acid, among other nutrients, from high river discharges due to glacier melt and heavy rain, fertilize the photic zone by mixing with macronutrient-loaded oceanic deep water^46,49,81,85,86^. This large fresh water input in conjunction with higher irradiance reaching the surface during spring and summer, wind stress, tide and complex bottom topography promotes alternating processes of vertical and horizontal stratification/mixing of the water column, enhancing primary production as well as plankton aggregation^87–89^. In this context, areas selected by blue whales in the NCP might not just be of high biological productivity, but where frontal dynamics lead to highly concentrated prey patches.

SST presented an equivocal pattern regarding blue whale movement patterns, suggesting a preference for colder waters in four individuals and the opposite in four other individuals (Table 1, Fig. 3b). This might be a temporal issue if whales in some years/seasons found their prey in colder/warmer waters. For instance, Ancud Gulf tends to present higher temperatures during spring and summer than the Corcovado Gulf as the latter represents the main entrance path for sub-superficial oceanic colder waters into the Chiloe inner sea. Alternatively, the lack of a clear trend in observed blue whale movement patterns regarding SST might be the result of a preference for intermediate temperatures that linear predictors failed to detect^76^.

Blue whales appear to respond to dynamic water-column processes by performing continuous behavioral changes without necessarily departing from relatively discrete areas (*e.g.* Ancud Gulf and Moraleda Channel, Fig. 2). For instance, whales ID#11, ID#13 and ID#15 presented a higher probability of reducing their velocity nearby areas of high productivity and strong thermal gradients, a higher probability of increasing persistence nearby areas of high productivity (for whales ID#11 and ID#15, Table 1), and all three spent from one to 3 months within specific micro-basins (Ancud Gulf and Moraleda Channel). This suggests that both transit-like and ARS behaviors co-occur spatially, temporarily oscillating with the suitability of foraging conditions.

Higher blue whale densities observed in the same areas where tagged individuals presented ARS behavior in a previous study^16^ could have been attributed to multiple individuals entering and leaving these areas. However, the results presented here show that instrumented blue whales concentrate in relatively discrete areas for extended periods of time (up to 3 months) searching for and exploiting available resources. The limited movement elicited by blue whales might be regarded as an indicator of low interspecific competition, considering that their population abundance is still estimated to be considerably below pre-whaling levels^16,90,91^. Other mechanisms like dominance^92^ and predator avoidance^93^, have been purported to explain limited animal movement. Thus, other factors should be considered in the future for understanding other dimensions of blue whales’ habitat selection process, as well as temporal variations on it.

Independently, both SDM and movement models predictions, highlighted similar areas of aggregation for blue whales in NCP based on observed oceanographic conditions (see Supplementary Fig. S5 online). These are clearly delimited by our RPEW map and considered Ancud Gulf, the Western coast of Chiloe Island, Corcovado Gulf / Moraleda Channel (CGMC), and Adventure Bay (Figs. 1 and 5). As previous SDMs were restricted to areas within 25 km from shore, some offshore areas visited by blue whales were not considered during RPEW computation. However, as the overall tendency to remain in coastal waters by instrumented whales was clear (Fig. 2f), we consider RPEW to be adequate.

### Quantifying overlap with vessel traffic

For Chile, detailed and freely available vessel traffic data as those used here are limited to recent years (2019–2020), precluding long term assessments on vessel traffic spatiotemporal variation^95^. Although limited to 10 months of data, results showed little intra-fleet variation for the transport and aquaculture vessel activities, as well as, for those occurring in the inner sea for both fishing fleets (see Supplementary Figs. S1–S4 online). This was expected as transport and logistic support operations from aquaculture operations are less variable than the shifting resource-tracking operations of fishing vessels. In addition, the inner waters concentrate obligated marine corridors for entering/leaving the area which are used similarly regardless of vessel type. Henceforth, our estimates are expected to adequately reflect general vessel traffic patterns for each fleet but inspecting possible temporal variation in these patterns should be pursued in the future.

The four different vessel fleets considered here elicited differences in VD values and their spatial use of the study area (Fig. 6). While artisanal and industrial fishing fleets utilize inner waters to the east and open waters to the west of the study area, aquaculture and transport fleets are mainly constrained to inner waters (Fig. 6). According to Chilean legislation, the artisanal fishing fleet is restricted to operate within 5 nm (9.3 km) from the coast in open and inner waters while the industrial fishing operations are to be performed beyond this area to the West. This might explain the artisanal fishing fleet´s high score on the similarity statistics, indicating the largest degree of overlap with blue whale coastal distribution. In other words, this fleet distributes the RPVEW more homogeneously matching blue whale distribution, while other fleets concentrate only at specific areas (lower degree of overlap). In comparison with results presented here, a study using the same overlap statistics, showed a higher degree of overlap between vessels and three species of cetaceans in the Mediterranean Sea^73^. This was expected as the Mediterranean Sea is a high intensity vessel traffic area^96^. However, most of the marine traffic recorded in that study (73.3%) corresponded to small sailing boats, suggesting low probabilities of lethal ship-strikes in general but pinpointing that shipping routes (where larger vessels navigate) might pose higher risk. This brings forward the fact that spatial overlap is just one of the factors affecting collision risk and its outcome, with vessel density, speed and size also contributing to it^39,40,97^. Although the industrial fishing fleet presents a lower degree of spatial overlap with blue whales and the lowest number of operating vessels, industrial vessels might yield a higher probability of lethal interactions if they occur, due to larger vessel size. This fleet also presented a particular pattern of high RPVEW values off Adventure Bay (Fig. 6).

With up to 729 active vessels operating per day (83% of the total) and up to 78 vessels per day crossing a single grid-cell (VD), aquaculture fleet corresponds to the largest and most densely distributed fleet in the NCP. Hence, while RPVEW predictions highlights the specific areas where interactions are more likely to occur for each vessel fleet, in absolute terms, it is possible that the aquaculture fleet represents the major driver of negative vessel-whale interactions in NCP.

When considering results from all fleets together it is clear that the inner waters largely concentrate higher VD and high RPVEW values for all fleets (Fig. 6). This area holds the largest number of human settlements in the NCP and the main port pertaining to the regional capital, Puerto Montt, raising concerns for potential collisions, behavioral disturbance and/or heavy noise exposure^38,94,98–101^ for blue whales there. Although, no systematic monitoring or registering protocol exists in this region, local authorities’ statements and the local press have documented at least three large whale mortality events linked to vessel collisions in the NCP (two blue whales and one sei whale), with two occurring nearby Puerto Montt and the other one at CGMC (Fig. 5). The ability of blue whales to avoid approaching vessels appears to be limited to relatively slow descents/ascents, with no horizontal movements away from a vessel^102,103^, therefore, collision events might pose significant threats to survival and recovery^97^ for this endangered population. As inner waters of NCP might be considered, at the time, the spot of higher relative and absolute probabilities of negative interactions between blue whales and vessels, management actions are urgently needed to be implemented. For now, the most effective way to reduce collision risk is to keep whales and vessels apart, either in space or time, and where/when this is not possible, other measures (such as speed regulation) can be sought and applied singularly or in combination, considering variations in vessel activity and whale´s distribution^40,102,104^, as data become available. In addition, it is important to acknowledge that all analyses performed here were restricted to vessels carrying transponders and legally mandated to submit position data. Therefore, several vessels types operating in the area that could contribute to collision risk (*e.g.* international cargo and tankers, cruiseliners, as well as artisanal, recreational and military vessels) are currently unaccounted for.

Because widely migratory species, such as the blue whale, do not recognize political boundaries, it is of great importance to identify the location of corridors and critical areas where they perform their vital activities (i.e., feed, migrate, breed, calve) to provide baseline information for their conservation. Efforts must be implemented at the local, national and international scales if success is to be reached, as ESP blue whale population recovery might be jeopardized by the loss of even a few individuals a year^16^ after being severely depleted by the whaling industry during the 20th Century.

### Modelling approach

One of the main differences between our modelling approach and previously published SSMs is in that behavioral variation that arises from the dependence on time-varying parameters (*σ*_*t*_ and *βt*) rather than switches in discrete pre-determined behavioral states^30,31,65,107^. While the latter approach allows formal prediction, testing on the spatio-temporal occurrence of known behavioral modes (*e.g.* areas where ARS is likely to occur), time-varying approaches permit investigating variation in movement patterns that cannot, or are not desired to be, categorized a priori^65,107,108^. This poses a significant advantage in cases where animal movement fails to conform to the usual transit/ARS binary view. For instance, a previous work^14^ fitted a switching SSM to most of the data we analyzed here and found that transit states were very rare within the NCP. In agreement with this, our results show that *ca.* 75% of all whale estimated locations presented persistence values lower than 1.6 h, which is consistent with the biological expectation of whales primarily engaged in foraging related activities within NCP^12^. In this scenario, attempting to explore the effect of environmental variables on switching probability between ARS and transit states^76^ would have been difficult, as very few locations and their associated covariates would have been available for the transit state. By exploring changes in movement parameters, we can assess how animals’ velocity and/or persistence respond to environmental covariates without the need of further assumptions. Following the transit/ARS rationale of conventional switching SSMs, one would expect that if a covariate is correlated with *σ*_*t*_ it also would be with *βt,* but with an opposite sign. That is, at certain covariate values an animal’s velocity and persistence are likely to decrease indicating ARS behavior, as was the case for several individuals and variables (Table 1). However, this does not need to always be the case, as shown by whales ID#11 and ID#15, which reduced their velocity near areas of high productivity in conjunction with increased persistence (Table 1). In general, this might occur because both transit and ARS behavior co-occur in similar areas with respect to DAHCC but differ in other variables (SST and thermal gradients). Nonetheless, alternative explanations for other behaviors, apart from transit/ARS, might arise. For instance, short-lived chasing bursts (escorting-like behavior) has been described for the NCP^109^ , which are expected to present high velocities but not necessarily high persistence. On the other hand, slow persistent behavior, mostly present in whales tagged in years with the highest data transmission throughput (2016–2019, Fig. 2d–e, Table 1), might be explained by the ratio of the location error relative to the scale of movement. Thus, if short time periods separate two or more locations with limited movement, high persistence might arise from negligible variation in both speed and location, as observation error increases disproportionately relative to the scale of the movement process.

Overall, our modelling approach accounted for observational error and allowed for the incorporation of environmental covariates to inform movement parameters without the need for regularization of location data into fixed time intervals^30,65^, all in one single step. By fitting the model through the R package “TMB” analysis took an average of 60.5 s to run (range: 2.6–310.6, processor: Intel Core i7-7700HQ at 2.8 GHz, RAM: 32 GB) which is a significant advantage when processing large amounts of data.

## Conclusions

Blue whale movement patterns agree with previous studies on their distribution, highlighting the importance of coastal waters and reinforcing our knowledge about primary production and thermal fronts as important environmental drivers for this species´ habitat selection process in the NCP. Considering defined priority areas for blue whale conservation in the area, those located at inner waters concentrated the highest probabilities of whales interacting with vessels. Among the studied vessel fleets, the unparalleled size of the aquaculture fleet indicates this could play a decisive role in modulating potential negative vessel-whale interactions within NCP. The results of this study clearly pinpoint specific areas where management actions are urgently needed, especially considering the undetermined number of vessels strikes and levels of noise exposure in the region. This information should be considered by Governmental and International organizations to inform, design, and rapidly implement mitigation action using existing national and international conservation instruments.

## Supplementary Information

Supplementary Information

## Data Availability

C +  + /TMB code for fitting the model (CTCRW_matrix_cov.cpp), raw telemetry data and accompanying covariate data are available as Supplementary Information.
