# Supplementary material for: Defining priority areas for blue whale conservation and investigating overlap with vessel traffic in Chilean Patagonia, using a fast-fitting movement model
Source: Sci Rep. 2021 Feb 1;11:2709. doi: 10.1038/s41598-021-82220-5 (PMC7851173; doi:10.1038/s41598-021-82220-5)
Supplement: Supplementary file 1 — Supplementary Information [file 41598_2021_82220_MOESM1_ESM.pdf]

Supplementary file

Defining priority areas for blue whale conservation and investigating overlap with vessel traffic in Chilean Patagonia, using a fast-fitting movement model

*Luis Bedriñana-Romano[1,2], *Rodrigo Hucke-Gaete[1,2], Francisco A. Viddi[1,2], Devin Johnson[3], Alexandre N. Zerbini[3,4,5,6], Juan Morales[7], Bruce Mate[8] & Daniel M. Palacios[8]

[1]Instituto de Ciencias Marinas y Limnológicas, Facultad de Ciencias, Universidad Austral de Chile, Casilla 567, Valdivia, Chile
[2] NGO Centro Ballena Azul, Valdivia, Chile
[3]Marine Mammal Laboratory Alaska Fisheries Science Center/NOAA 7600 Sand Point Way NE, Seattle, WA, USA
[4]Marine Ecology and Telemetry Research, 2468 Camp McKenzie Tr NW, Seabeck, WA, 98380, USA
[5]Cascadia Research Collective, 218 ½ 4th Ave, Olympia, WA, 98502, USA
[6]Instituto Aqualie, Av. Dr. Paulo Japiassú Coelho, 714, Sala 206, 36033-310, Juiz de Fora, MG, Brazil.
[7]Grupo de Ecología Cuantitativa, INIBIOMA-CONICET, Universidad Nacional del Comahue, Bariloche, Argentina
[8] Marine Mammal Institute and Department of Fisheries and Wildlife, Oregon State University, Hatfield Marine Science Center, Newport, Oregon, United States of America.
*Corresponding author: rhucke@uach.cl
*Corresponding author: luis.bedrinana.romano@gmail.com

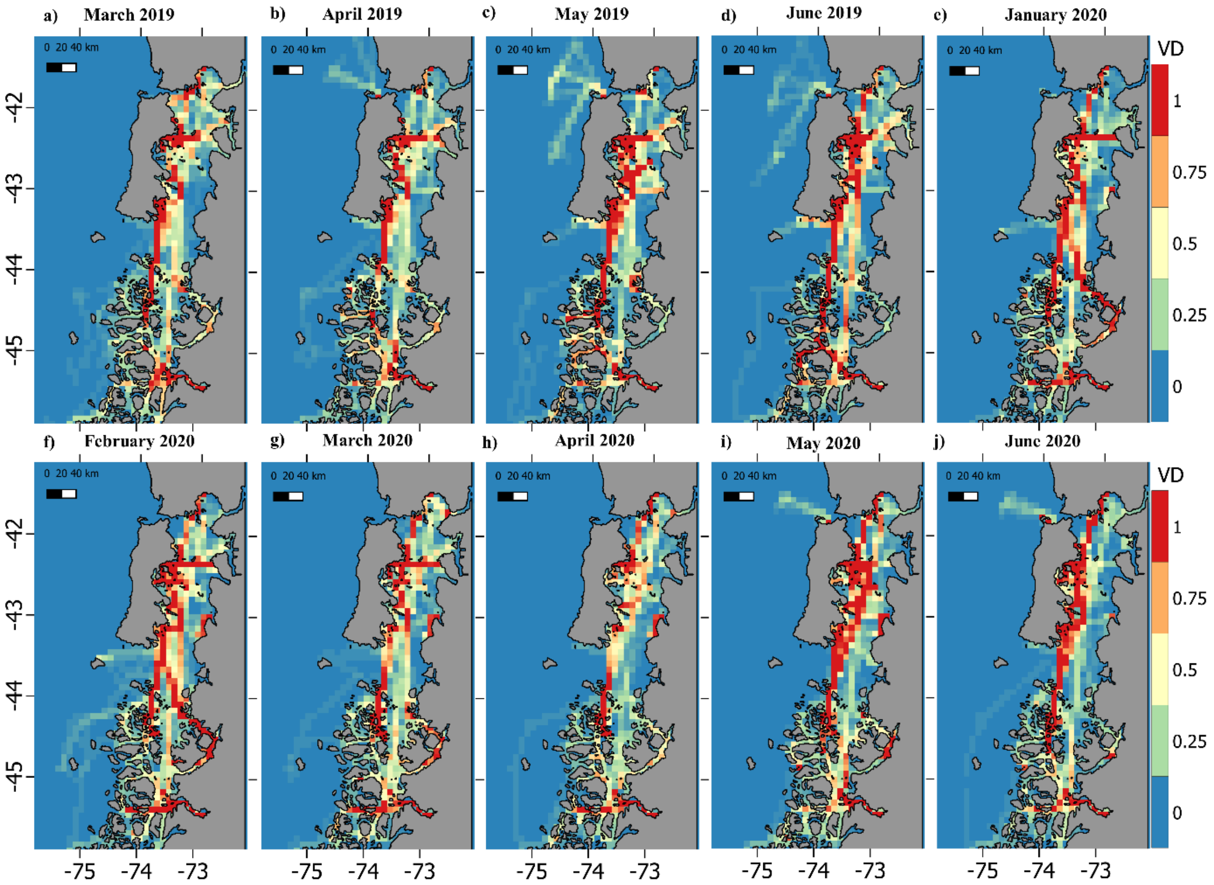

Supplementary Figure S1. Monthly vessel density (VD) data used for analyses for the transport fleet. VD is expressed as the mean number of vessels visiting each 8 x 8 km grid-cell per day. The data considered information from March to June of 2019 (a-d) and from January to June of 2020 (e-j). These months were selected considering blue whale presence in the area prior to winter migration. Data layers (including maps) were created in R ver. 4.0.2 (www.r-project.org) and ensembled in QGIS ver. 3.8.0 (www.qgis.org) for final rendering. Maps were created using data on bedrock topography from the National Centers for Environmental Information (https://maps.ngdc.noaa.gov/viewers/grid-extract/index.html). Values above 0 were considered land coverage.

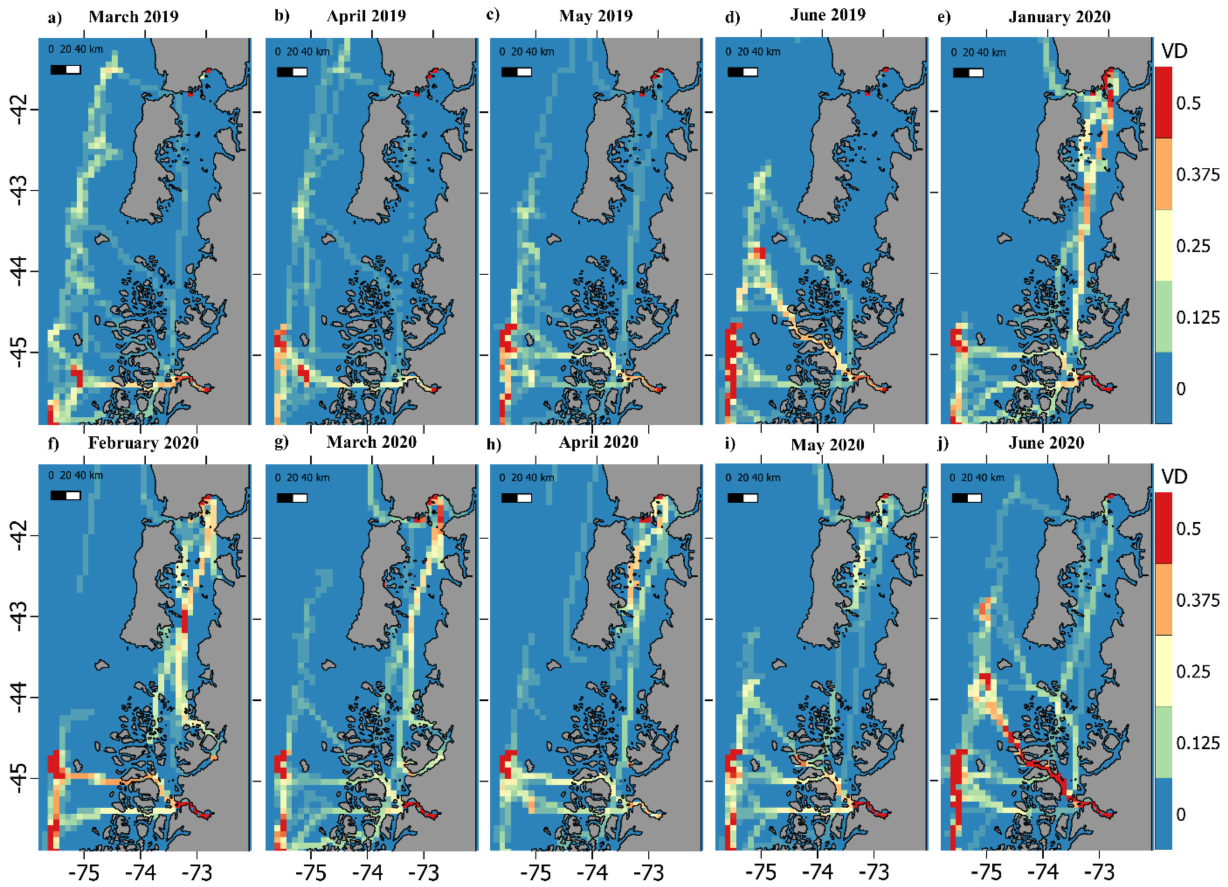

Supplementary Figure S2. Monthly vessel density (VD) data used for analyses for the industrial fishery fleet. VD is expressed as the mean number of vessels visiting each 8 x 8 km grid-cell per day. The data considered information from March to June of 2019 (a-d) and from January to June of 2020 (e-j). These months were selected considering blue whale presence in the area prior to winter migration. Data layers (including maps) were created in R ver. 4.0.2 (www.r-project.org) and ensembled in QGIS ver. 3.8.0 (www.qgis.org) for final rendering. Maps were created using data on bedrock topography from the National Centers for Environmental Information (https://maps.ngdc.noaa.gov/viewers/grid-extract/index.html). Values above 0 were considered land coverage.

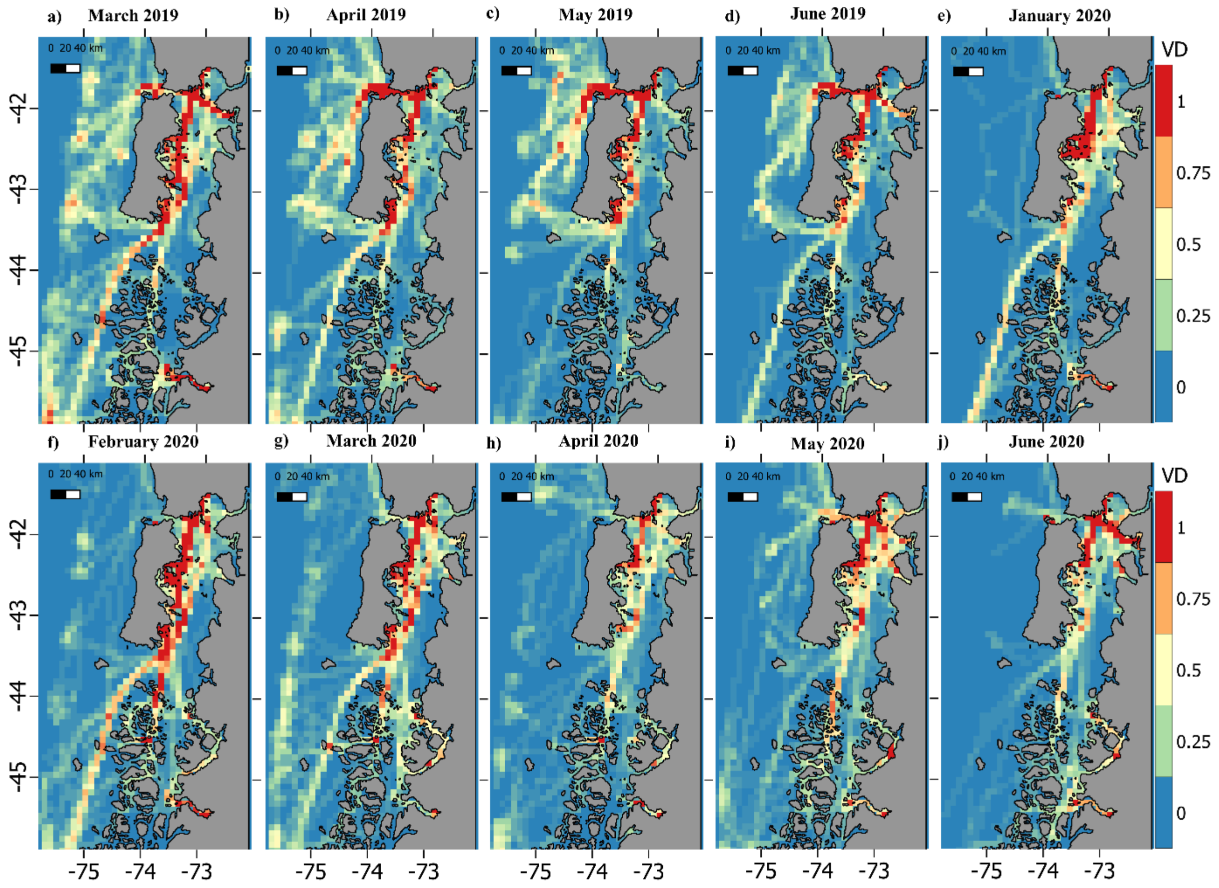

Supplementary Figure S3. Monthly vessel density (VD) data used for analyses for the artisanal fishery fleet. VD is expressed as the mean number of vessels visiting each 8 x 8 km grid-cell per day. The data considered information from March to June of 2019 (a-d) and from January to June of 2020 (e-j). These months were selected considering blue whale presence in the area prior to winter migration. Data layers (including maps) were created in R ver. 4.0.2 (www.r-project.org) and ensembled in QGIS ver. 3.8.0 (www.qgis.org) for final rendering. Maps were created using data on bedrock topography from the National Centers for Environmental Information (https://maps.ngdc.noaa.gov/viewers/grid-extract/index.html). Values above 0 were considered land coverage.

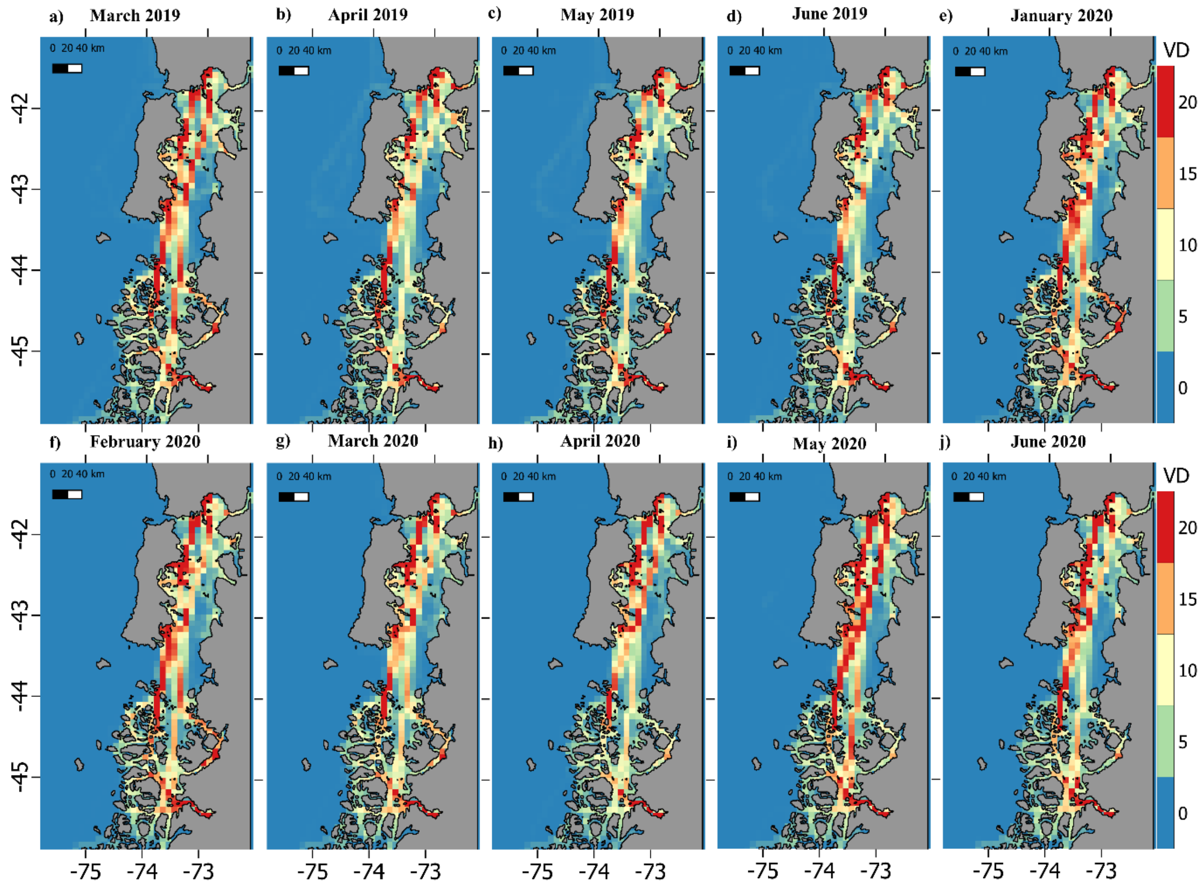

Supplementary Figure S4. Monthly vessel density (VD) data used for analyses for the aquaculture fleet. VD is expressed as the mean number of vessels visiting each 8 x 8 km grid-cell per day. The data considered information from March to June of 2019 (a-d) and from January to June of 2020 (e-j). These months were selected considering blue whale presence in the area prior to winter migration. Data layers (including maps) were created in R ver. 4.0.2 (www.r-project.org) and ensembled in QGIS ver. 3.8.0 (www.qgis.org) for final rendering. Maps were created using data on bedrock topography from the National Centers for Environmental Information (https://maps.ngdc.noaa.gov/viewers/grid-extract/index.html). Values above 0 were considered land coverage.

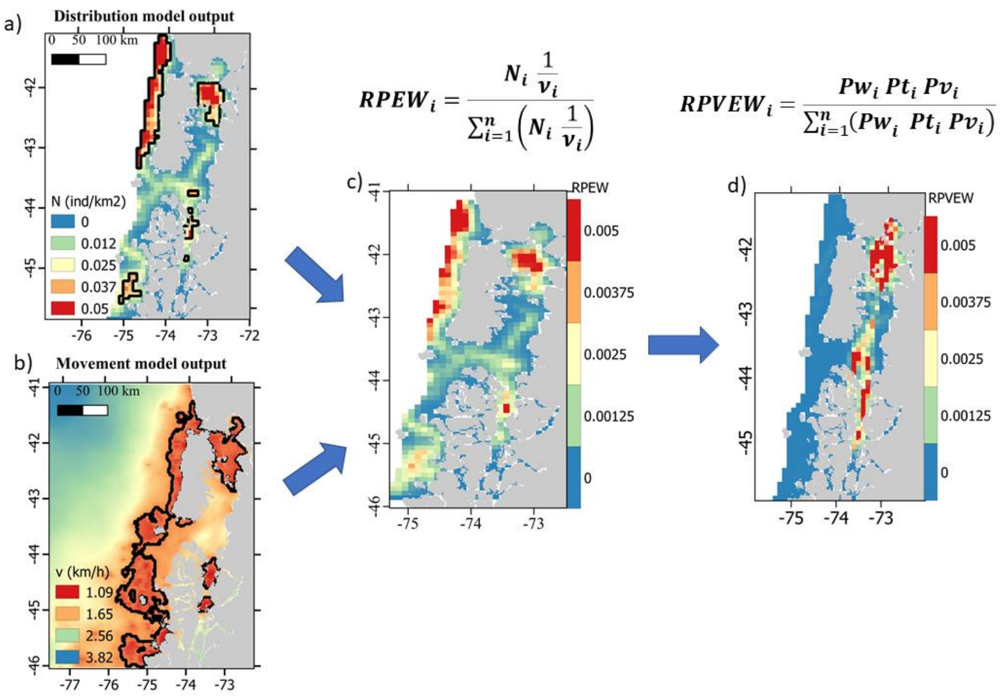

Supplementary Figure S5. Schematic summarizing methods used for assessing the relative probability of whales encountering vessels. Panels a and b show the output of distribution and movement models, respectively. Polygons delimited by black lines show the highest 20% of expected densities (a) and lowest long-term velocities (b). These polygons are larger for the movement model output because it is based on a larger study area incorporating more offshore waters. Expected densities (N) and time (1/v) allocated to each grid-cell are used for calculating the relative probability of encountering whales (RPEW) showed in panel c. *Pw, Pt and Pv* correspond to the expected relative density, relative time and relative vessel density for each grid-cell, which are combined for calculating the relative probability of whales encountering vessels (RPVEW) showed in panel d. Data layers (including maps) were created in R ver. 4.0.2 (www.r-project.org) and ensembled in QGIS ver. 3.8.0 (www.qgis.org) for final rendering. Maps were created using data on bedrock topography from the National Centers for Environmental Information (https://maps.ngdc.noaa.gov/viewers/grid-extract/index.html). Values above 0 were considered land coverage.

Movement model

TMB/C++ code for fitting the movement model described in the main text. A .cpp file of this model is also provided with the supplementary files, which is ready to use with R.

```
#include <TMB.hpp>


template<class Type>

Type objective_function<Type>::operator() ()

{
```

```
// Data//
DATA_VECTOR(x);
DATA_VECTOR(y);
DATA_VECTOR(delta);
DATA_VECTOR(errx);
DATA_VECTOR(erry);
DATA_SCALAR(sd1);
DATA_SCALAR(sd2);
DATA_SCALAR(sd_beta);
DATA_VECTOR(ahcc);
DATA_VECTOR(sst);
DATA_VECTOR(grad);

//Parameters//

PARAMETER_MATRIX(alpha1); // rbind(u1,V1), i.e., 2xN matrix where alpha1[,1] = c(u1[1], V1[1])
PARAMETER_MATRIX(alpha2); // rbind(u2,V2)
PARAMETER_VECTOR(log_sigma);
PARAMETER_VECTOR(log_beta);
PARAMETER(B0);
PARAMETER(A0);
PARAMETER(A1);
PARAMETER(A2);
PARAMETER(A3);
PARAMETER(B1);
PARAMETER(B2);
PARAMETER(B3);
PARAMETER(log_sd_sigma);
//PARAMETER(log_sd_beta);
```

```
  //Transform Paameters//
  Type sd_sigma = exp(log_sd_sigma);
  //Type sd_beta = exp(log_sd_beta);




  // define bivariate state vectors, matrices, etc...
  matrix<Type> T(2,2); T.fill(0); T(0,0) = 1.0;
  matrix<Type> Q(2,2); Q.fill(0);
  matrix<Type> mean_alpha1(2,1);
  matrix<Type> mean_alpha2(2,1);

  Type nll = Type(0);
  // Initial conditions
  Q(0,0) = sd1*sd1; // here I interpreted sd1 to be the sd for initial loc and sd2 to be the sd for initial
velocity
  Q(1,1) = sd2*sd2;
  nll += density::MVNORM(Q)(alpha1.col(0)); // This implies the initial mean is zero, I might change
this.
  nll += density::MVNORM(Q)(alpha2.col(0)); // MVNORM_t returns -log mvn density

  vector<Type> sigma=exp(log_sigma);
  vector<Type> beta=exp(log_beta);
  //Process model
  for( int i=1;i < alpha1.cols();i++) {

    nll -= dnorm(log_beta(i-1), B0 + B1 *ahcc(i-1)+ B2*sst(i-1) + B3*grad(i-1), sd_beta, true);
    nll -= dnorm(log_sigma(i-1), A0 + A1 *ahcc(i-1)+ A2*sst(i-1) + A3*grad(i-1), sd_sigma, true);

    T(0,1) = (1-exp(-beta(i-1)*delta(i-1)))/beta(i-1);
    T(1,1) = exp(-beta(i-1)*delta(i-1));
```

```
    Q(0,0) = (sigma(i-1)*sigma(i-1))*(delta(i-1)-(2/beta(i-1))*(1-exp(-beta(i-1)*delta(i-1)))+(1/(2*beta(i-
1)))*(1-exp(-2*beta(i-1)*delta(i-1))));
    Q(0,1) = 0.5*(sigma(i-1)*sigma(i-1))*(1 - 2*exp(-beta(i-1)*delta(i-1)) + exp(-2*beta(i-1)*delta(i-1)));
    Q(1,0) = Q(0,1);
    Q(1,1) = 0.5*(sigma(i-1)*sigma(i-1))*beta(i-1)*(1-exp(-2*beta(i-1)*delta(i-1)));

    mean_alpha1 = T * alpha1.col(i-1);
    mean_alpha2 = T * alpha2.col(i-1);
    nll += density::MVNORM(Q)(alpha1.col(i)-mean_alpha1);
    nll += density::MVNORM(Q)(alpha2.col(i)-mean_alpha2);
  }
  //Observation model
  for( int i=0;i < x.size();i++) {

    nll-=dnorm(x(i),alpha1(0,i),errx(i),true);
    nll-=dnorm(y(i),alpha2(0,i),erry(i),true);
  }

  ADREPORT(alpha1);
  ADREPORT(alpha2);
  ADREPORT(log_sigma);
  ADREPORT(log_beta);
  ADREPORT(B0);
  ADREPORT(A0);
  ADREPORT(A1);
  ADREPORT(A2);
  ADREPORT(A3);
  ADREPORT(B1);
  ADREPORT(B2);
  ADREPORT(B3);
```

```
ADREPORT(sd_sigma);
//ADREPORT(sd_beta);
return nll;
```

Raw data

Id=Unique number identifying specific tagged whale, date=date and time for each location, lc=Argos locations class, v1 and v2 =Variances for modelling error in locations derived from the Argos error ellipse, long=longitude, lat=latitude, delta= time difference between adjacent locations, grad, ahcc and sst correspond to standardized environmental covariates.

id	date	lc	v1	v2	long	lat	delta	grad	ahcc	sst
822	2/14/2004 12:13	A	1846137731	80281855.35	-73.352	-44.023	47170	6.3470419	-0.674402574	-0.037849549
822	2/16/2004 23:41	2	863491.9002	117318.8964	-73.423	-43.818	214047	-0.153070501	-0.65944062	1.743408246
822	2/17/2004 12:45	1	19838678.47	1565421.652	-73.421	-43.863	47059	-0.102508691	-0.762496887	1.750580318
822	2/17/2004 20:49	0	4697718710	205165923.2	-73.447	-43.94	29058	-0.124894357	-1.468477498	1.658633548
822	2/17/2004 23:16	2	863491.9002	117318.8964	-73.533	-43.907	8815	-0.080468227	-1.479281264	1.531677446
822	2/18/2004 12:15	A	1846137731	80281855.35	-73.277	-44.066	46755	-0.084884064	-1.611766755	1.536791773
822	2/18/2004 20:39	A	1846137731	80281855.35	-73.437	-43.918	30237	-0.099059392	-1.593429334	1.55735543
822	2/19/2004 12:20	A	1846137731	80281855.35	-73.442	-43.872	56457	-0.497377238	-1.49802235	1.606941331
822	2/19/2004 20:26	B	1445289616	162773088	-73.571	-43.927	29141	-0.39095108	-2.414489334	1.591380008
822	2/20/2004 12:07	A	1846137731	80281855.35	-73.362	-44.037	56444	1.466122164	-2.371403731	1.002190701
822	2/20/2004 20:16	1	19838678.47	1565421.652	-73.378	-44.068	29390	1.466122164	-2.361317687	1.002190701

822	2/21/2004 9:37	B	1445289616	162773088	-73.252	-44.169	48062	1.771800354	-2.361317687	1.057787659
822	2/21/2004 20:06	1	19838678.47	1565421.652	-73.611	-43.838	37732	1.834997154	-0.766302252	1.074858632
822	2/21/2004 23:19	0	4697718710	205165923.2	-73.479	-43.863	11533	0.044537606	-0.953048735	1.220987354
822	2/22/2004 20:00	Z	343535924.1	50175612.8	-73.131	-44.003	74471	4.173427613	-0.888974111	1.370577103
822	2/23/2004 12:00	B	1445289616	162773088	-73.444	-43.739	57601	1.232408211	-0.764537706	1.543113269
822	2/24/2004 23:55	B	1445289616	162773088	-73.401	-44.062	129337	-0.468737341	-1.326459507	1.454221094
822	2/25/2004 12:44	A	1846137731	80281855.35	-73.463	-44.017	46119	-0.468737341	-1.326459507	1.454221094
822	2/25/2004 23:30	1	19838678.47	1565421.652	-73.621	-43.938	38762	0.848966386	-1.326459507	1.647929295
822	2/26/2004 9:22	B	1445289616	162773088	-73.83	-43.893	35546	0.798883315	-1.329297869	1.638987921
822	2/26/2004 12:26	B	1445289616	162773088	-73.462	-44.005	11040	-0.07467215	-2.749278275	1.656001339
822	2/26/2004 23:04	A	1846137731	80281855.35	-73.564	-43.943	38234	-0.335400275	-2.749278275	1.635734689
822	2/27/2004 12:17	A	1846137731	80281855.35	-73.325	-44.085	47584	-0.335400275	-2.749278275	1.635734689
822	2/28/2004 12:03	B	1445289616	162773088	-73.233	-44.068	85546	-0.168644695	-2.728055343	1.625209949
822	2/28/2004 20:28	A	1846137731	80281855.35	-73.448	-43.958	30325	-0.322886701	-1.568561824	1.597340701
822	2/29/2004 20:16	A	1846137731	80281855.35	-73.439	-43.904	85708	-0.307648547	-1.540272584	1.479179404
822	3/1/2004 20:05	A	1846137731	80281855.35	-73.5	-44.069	85690	-0.637819327	-2.078181771	0.99316467

822	3/1/2004 23:08	B	1445289616	162773088	-73.453	-44.047	10989	-0.51696615	-2.078181771	0.709693299
822	3/4/2004 9:46	B	1445289616	162773088	-73.401	-43.821	211066	-0.488076664	-2.078181771	0.702500984
822	3/4/2004 12:43	A	1846137731	80281855.35	-73.335	-44.073	10669	-0.497446977	-0.821012891	0.745111315
822	3/5/2004 12:38	A	1846137731	80281855.35	-72.9	-44.108	86101	-0.481920181	-0.586894966	0.494713771
822	3/5/2004 23:14	A	1846137731	80281855.35	-73.329	-44.27	38138	-0.586681142	-2.730788511	0.281198959
822	3/6/2004 9:00	B	1445289616	162773088	-73.416	-44.331	35177	-0.587826201	-2.560197733	0.280569736
822	3/6/2004 12:16	B	1445289616	162773088	-73.391	-44.313	11725	-0.586264269	-2.741115046	0.281820184
822	3/6/2004 20:48	0	4697718710	205165923.2	-73.341	-44.305	30766	-0.584705003	-2.83277466	0.282811483
822	3/7/2004 20:36	B	1445289616	162773088	-73.35	-44.175	85637	0.441775216	-2.776083371	-0.588073193
822	3/8/2004 23:45	1	19838678.47	1565421.652	-73.316	-44.129	97746	2.083071326	-0.984314859	-0.366544762
822	3/9/2004 20:13	0	4697718710	205165923.2	-73.528	-43.934	73687	1.562126931	-2.246936263	-0.418175777
822	3/9/2004 23:21	2	863491.9002	117318.8964	-73.544	-43.96	11287	0.816826392	-2.277457785	-0.302617796
822	3/10/2004 20:03	B	1445289616	162773088	-73.379	-44.239	74516	0.878951785	-2.320469456	-0.312047586
822	3/12/2004 9:54	B	1445289616	162773088	-73.318	-44.112	136232	1.581345042	-2.366264897	-0.117190655
822	3/12/2004 12:40	A	1846137731	80281855.35	-73.337	-43.975	10001	0.266872767	-2.267290523	-0.006814429
822	3/12/2004 23:47	1	19838678.47	1565421.652	-73.132	-43.858	40018	-0.107659935	-2.668453428	-0.313737192

822	3/13/2004 12:28	1	19838678.47	1565421.652	-73.217	-43.646	45677	0.510961465	-2.286810282	-0.679094349
822	3/13/2004 23:25	1	19838678.47	1565421.652	-73.215	-43.781	43001	-0.445182557	-2.307327789	0.231253843
822	3/14/2004 12:25	B	1445289616	162773088	-73.162	-43.902	46802	-0.443020029	-2.307327789	0.231713041
822	3/14/2004 12:41	B	1445289616	162773088	-73.207	-43.902	964	-0.440580753	-2.366266935	0.232576535
822	3/14/2004 20:56	B	1445289616	162773088	-73.068	-43.435	29700	-0.45866444	-1.31530814	-0.181383953
822	3/14/2004 23:01	1	19838678.47	1565421.652	-73.159	-43.474	7503	-0.453156572	-1.950844295	-0.125186202
822	3/15/2004 12:02	B	1445289616	162773088	-73.188	-43.515	46816	-0.452991263	-2.419223125	-0.110113075
822	3/15/2004 20:45	1	19838678.47	1565421.652	-73.133	-43.574	31404	-0.344980214	-1.654923926	-0.123106804
822	3/17/2004 9:39	B	1445289616	162773088	-73.472	-44.019	132856	-0.452642413	-2.633610387	-0.205502246
822	3/17/2004 20:23	2	863491.9002	117318.8964	-73.15	-43.669	38655	-0.042354249	-2.440619871	0.780010861
822	3/17/2004 23:32	1	19838678.47	1565421.652	-73.14	-43.682	11293	-0.042175283	-2.479920972	0.781025073
822	3/18/2004 9:00	B	1445289616	162773088	-72.571	-43.767	34118	-0.042175283	-2.479920972	0.781025073
822	3/18/2004 20:13	1	19838678.47	1565421.652	-73.204	-43.469	40332	-0.112472907	-1.632068031	0.898269992
822	3/18/2004 23:08	0	4697718710	205165923.2	-73.16	-43.5	10542	-0.109886647	-1.61992581	0.897953132
822	3/19/2004 12:49	0	4697718710	205165923.2	-73.159	-43.626	49271	-0.072660932	-1.088892026	0.86261224
822	3/19/2004 20:01	0	4697718710	205165923.2	-73.191	-43.65	25918	-0.165260539	-1.087697936	0.941060103

822	3/19/2004 23:31	B	1445289616	162773088	-73.237	-43.723	12560	-0.165260539	-1.087697936	0.941060103
822	3/20/2004 9:52	B	1445289616	162773088	-73.009	-43.839	37249	-0.165260539	-1.087697936	0.941060103
822	3/20/2004 20:24	B	1445289616	162773088	-73.147	-43.597	37971	-0.302171742	-1.499375888	0.893346995
822	3/21/2004 12:27	0	4697718710	205165923.2	-73.188	-43.511	57759	-0.312981876	-1.639723145	0.899890897
822	3/21/2004 23:37	1	19838678.47	1565421.652	-73.317	-43.279	40224	-0.4911191	-3.213029789	0.710724627
822	3/22/2004 9:19	A	1846137731	80281855.35	-73.348	-43.032	34882	-0.4911191	-3.213029789	0.710724627
822	3/22/2004 23:13	B	1445289616	162773088	-73.244	-43.361	50054	-0.404902784	-1.525842721	0.511446559
822	3/23/2004 8:57	B	1445289616	162773088	-73.528	-43.386	35022	-0.39961337	-1.259373068	0.497143173
822	3/23/2004 12:00	B	1445289616	162773088	-72.947	-43.566	11024	-0.39961337	-1.259373068	0.497143173
822	3/23/2004 20:53	A	1846137731	80281855.35	-73.48	-43.871	31932	-0.359612832	-1.689802836	0.328113525
822	3/24/2004 20:44	1	19838678.47	1565421.652	-73.513	-43.809	85907	-0.293600123	-0.627584287	0.127473804
822	3/25/2004 9:50	1	19838678.47	1565421.652	-73.54	-43.871	47150	-0.283862786	-1.012339112	0.079458765
822	3/25/2004 23:42	1	19838678.47	1565421.652	-73.707	-43.834	49932	-0.251213625	-0.526154772	-0.567212014
822	3/26/2004 12:48	B	1445289616	162773088	-73.76	-43.712	47107	-0.304002546	-0.682924501	-0.554912777
822	3/26/2004 20:22	1	19838678.47	1565421.652	-73.463	-43.646	27271	-0.204941255	-0.75595696	-0.48496271
822	3/26/2004 23:20	0	4697718710	205165923.2	-73.469	-43.652	10694	-0.216532427	-0.761592893	-0.529992104

822	3/27/2004 12:48	0	4697718710	205165923.2	-73.16	-43.953	48440	-0.435223472	-1.294014465	-0.661738497
822	3/27/2004 20:10	0	4697718710	205165923.2	-73.108	-43.831	26550	-0.770391162	-2.753898636	-0.671687178
822	3/27/2004 23:00	B	1445289616	162773088	-73.056	-43.818	10190	-0.769034547	-3.01367288	-0.672298584
822	3/27/2004 23:30	B	1445289616	162773088	-73.101	-43.84	1800	-0.768418444	-3.033936723	-0.672411603
822	3/28/2004 12:37	B	1445289616	162773088	-73.085	-43.796	47214	-0.762443548	-2.199634237	-0.67369948
822	3/28/2004 19:59	3	102706.2314	10029.44029	-73.057	-43.7	26499	-0.758622492	-1.703400548	-0.673512307
822	3/29/2004 9:52	B	1445289616	162773088	-73.138	-43.826	50005	-0.683537817	-1.335344003	-0.67416535
822	3/29/2004 23:52	0	4697718710	205165923.2	-72.963	-43.558	50377	-0.688657039	-1.259035865	-0.707610792
822	3/30/2004 12:15	B	1445289616	162773088	-73.107	-43.629	44595	-0.647170047	-0.858276869	-0.680480234
822	3/30/2004 23:27	3	102706.2314	10029.44029	-73.061	-43.804	40317	-0.709044361	-1.869916891	-0.752823248
822	3/31/2004 22:59	0	4697718710	205165923.2	-73.189	-43.601	84707	-0.563836309	-1.206251551	-0.93159927
822	4/1/2004 12:02	B	1445289616	162773088	-73.472	-43.872	47016	-0.526839581	-0.732353402	-0.921679215
822	4/1/2004 20:33	B	1445289616	162773088	-73.443	-43.868	30671	-0.12800731	-0.729556636	-0.248229904
822	4/2/2004 20:42	B	1445289616	162773088	-73.463	-43.799	86907	-0.061476147	-0.659575461	-0.41459069
822	4/2/2004 23:55	Z	343535924.1	50175612.8	-73.46	-43.841	11617	-0.062554049	-0.682628709	-0.42077514
822	4/3/2004 9:35	0	4697718710	205165923.2	-73.595	-43.744	34792	-0.052459596	-0.593360527	-0.38310467

822	4/3/2004 20:28	Z	343535924.1	50175612.8	-73.408	-44.013	39179	-0.107224782	-2.607105812	-0.720580389
822	4/3/2004 23:30	0	4697718710	205165923.2	-73.573	-44.021	10907	-0.078913425	-1.1835671	-0.635064595
822	4/4/2004 20:21	B	1445289616	162773088	-73.465	-43.623	75084	-0.149751419	-0.930877799	-0.55369289
822	4/4/2004 23:06	2	863491.9002	117318.8964	-73.49	-43.621	9901	-0.150614004	-0.947076779	-0.552000502
822	4/5/2004 20:32	B	1445289616	162773088	-73.353	-43.945	77110	-0.578316366	-0.998087296	-1.05802998
822	4/6/2004 23:59	B	1445289616	162773088	-73.073	-43.977	98826	-0.572624585	-1.699355583	-0.944499072
822	4/7/2004 9:41	A	1846137731	80281855.35	-73.499	-44.125	34945	-0.583618572	-1.634509147	-0.954100345
822	4/8/2004 9:17	B	1445289616	162773088	-73.46	-44.103	84925	-0.422910055	-1.229770197	-0.76820854
822	4/10/2004 9:37	A	1846137731	80281855.35	-73.332	-44.06	174042	-0.38420711	-0.940985111	-0.561836404
822	4/11/2004 20:39	A	1846137731	80281855.35	-73.43	-44.352	126073	-0.581439216	-1.622513753	-1.344347016
822	4/12/2004 12:48	0	4697718710	205165923.2	-73.391	-44.193	58177	-0.573794197	-1.682503073	-1.34121368
822	4/12/2004 23:15	0	4697718710	205165923.2	-73.207	-44.265	37601	-0.570076986	-1.737337246	-1.347779202
822	4/13/2004 20:19	0	4697718710	205165923.2	-73.272	-44.165	75830	-0.574558899	-1.600218309	-1.264178953
822	4/14/2004 9:38	Z	343535924.1	50175612.8	-73.303	-44.16	47952	-0.571009203	-1.572172223	-1.262623353
822	4/14/2004 20:09	B	1445289616	162773088	-73.09	-44.206	37882	-0.571569166	-1.553095289	-1.205698148
822	4/15/2004 19:59	Z	343535924.1	50175612.8	-73.536	-44.221	85768	-0.57335974	-1.642002158	-1.210807168

822	4/16/2004 9:28	0	4697718710	205165923.2	-73.413	-44.233	48556	-0.511195838	-1.667500309	-1.023177658
822	4/16/2004 23:24	B	1445289616	162773088	-73.463	-44.201	50152	-0.468258579	-1.502399713	-0.999297219
822	4/17/2004 20:40	0	4697718710	205165923.2	-73.432	-44.148	76568	-0.472495667	-1.430218289	-0.949430154
822	4/18/2004 12:53	A	1846137731	80281855.35	-73.155	-44.254	58390	-0.473237122	-1.24921807	-0.954298517
822	4/19/2004 9:13	B	1445289616	162773088	-73.382	-44.338	73220	-0.197805487	-1.361146102	-0.943636509
822	4/19/2004 20:51	B	1445289616	162773088	-73.328	-43.78	41852	-0.232983326	-1.755651072	-1.327732034
822	4/20/2004 9:38	B	1445289616	162773088	-73.462	-43.797	45992	-0.318985471	-0.754358478	-1.240116831
822	4/20/2004 12:48	B	1445289616	162773088	-73.377	-43.895	11424	-0.335572763	-0.617633853	-1.217964195
822	4/21/2004 9:14	0	4697718710	205165923.2	-74.095	-43.586	73569	-0.610769178	-0.55146001	-1.456653093
822	4/21/2004 12:35	B	1445289616	162773088	-74.206	-43.596	12031	-0.456811081	-0.578293132	-1.454674539
822	4/21/2004 20:29	B	1445289616	162773088	-74.324	-43.506	28466	3.167895139	-1.50595244	-1.021783149
822	4/22/2004 12:23	1	19838678.47	1565421.652	-74.443	-43.63	57255	2.32438274	-0.680385328	-1.521175181
822	4/22/2004 20:14	2	863491.9002	117318.8964	-74.646	-43.831	28244	1.454373681	0.091994658	-1.28132604
822	4/23/2004 12:11	Z	343535924.1	50175612.8	-74.462	-44.023	57433	1.687235382	0.265494604	-1.621029719
822	4/23/2004 20:04	A	1846137731	80281855.35	-74.422	-43.8	28397	1.818334051	0.175583775	-1.358628626
822	4/24/2004 9:38	B	1445289616	162773088	-74.96	-43.77	48818	-0.357203356	-0.14978441	-1.525182401

822	4/24/2004 23:33	A	1846137731	80281855.35	-75.039	-43.64	50119	0.662095534	-0.410442162	-1.352922288
822	4/25/2004 23:06	B	1445289616	162773088	-74.872	-42.975	84747	-0.287780555	-1.06501909	-1.080187869
822	4/26/2004 23:50	0	4697718710	205165923.2	-74.539	-42.869	89056	-0.35149277	-2.992387863	-0.901884304
822	4/28/2004 23:41	1	19838678.47	1565421.652	-74.445	-42.469	172276	-0.519540661	-1.546030004	-0.479020443
828	2/20/2004 9:49	1	19838678.47	1565421.652	-73.871	-43.596	48081	-0.309775561	-0.745792991	0.894934151
828	2/20/2004 12:07	1	19838678.47	1565421.652	-73.804	-43.621	8267	-0.10621833	-0.662753058	0.86174584
828	2/20/2004 23:45	2	863491.9002	117318.8964	-73.852	-43.562	41928	-0.670120698	-0.625331279	0.825454701
828	2/21/2004 9:39	B	1445289616	162773088	-73.643	-43.628	35600	-0.625689509	-0.785634064	0.840280183
828	2/21/2004 12:42	B	1445289616	162773088	-73.792	-43.559	10990	-0.721701798	-0.77688408	0.835466289
828	2/21/2004 20:08	0	4697718710	205165923.2	-73.884	-43.554	26768	-0.777552329	-0.962012369	0.82862989
828	2/21/2004 23:20	2	863491.9002	117318.8964	-74	-43.524	11511	-0.757033508	-1.108398457	0.940864828
828	2/22/2004 9:04	1	19838678.47	1565421.652	-74.015	-43.519	35070	-0.749654449	-1.179315628	0.940906291
828	2/22/2004 23:02	B	1445289616	162773088	-74.198	-43.439	50259	-0.864464473	-2.011280448	1.179206659
828	2/24/2004 9:56	1	19838678.47	1565421.652	-74.061	-43.487	125631	-0.778607787	-1.64930725	1.433891755
828	2/24/2004 12:58	0	4697718710	205165923.2	-74.064	-43.588	10914	-0.781421779	-1.417055245	1.432442814
828	2/24/2004 23:54	B	1445289616	162773088	-73.878	-43.543	39372	-0.699456467	-1.148096018	1.634003598

828	2/25/2004 9:32	0	4697718710	205165923.2	-74.465	-43.459	34711	-0.628629392	-0.665072628	1.704370516
828	2/25/2004 23:25	B	1445289616	162773088	-74.321	-43.06	49932	-0.135245559	-3.213029789	1.924845057
828	2/26/2004 12:30	A	1846137731	80281855.35	-74.494	-43.028	47117	-0.225547624	-3.213029789	1.914938137
828	2/26/2004 20:49	A	1846137731	80281855.35	-74.523	-42.972	29930	-0.243031703	-3.213029789	1.912605791
828	2/26/2004 23:04	1	19838678.47	1565421.652	-74.526	-42.961	8124	-0.60686294	-3.213029789	1.88438179
828	2/27/2004 12:06	1	19838678.47	1565421.652	-74.572	-42.956	46919	-0.685426399	-3.213029789	1.888608845
828	2/27/2004 12:16	2	863491.9002	117318.8964	-74.585	-42.965	582	-0.685426399	-3.213029789	1.888608845
828	2/28/2004 20:27	2	863491.9002	117318.8964	-74.544	-43.036	115854	-0.487396432	-3.213029789	1.785187239
828	2/28/2004 23:56	0	4697718710	205165923.2	-74.575	-43.022	12533	-0.723286489	-3.213029789	1.569939465
828	2/29/2004 9:42	B	1445289616	162773088	-74.841	-42.843	35165	-0.745264435	-3.213029789	1.563130964
828	2/29/2004 20:14	B	1445289616	162773088	-74.579	-42.747	37922	-0.688544588	-1.372338005	1.517471317
828	3/1/2004 20:04	0	4697718710	205165923.2	-74.315	-42.374	85791	-0.709222027	-1.176171403	1.317026475
828	3/2/2004 12:08	B	1445289616	162773088	-73.933	-42.596	57867	-0.529717807	-1.27235768	1.115756502
828	3/3/2004 12:54	1	19838678.47	1565421.652	-74.305	-42.374	89151	-0.664105685	-2.373181768	1.295980976
828	3/4/2004 9:45	A	1846137731	80281855.35	-74.269	-42.36	75094	-0.727898973	-2.049233467	1.083998142
828	3/5/2004 9:20	B	1445289616	162773088	-74.444	-42.251	84896	1.042094258	-1.763148864	0.609853735

828	3/5/2004 12:30	B	1445289616	162773088	-74.417	-42.381	11347	1.753168608	-2.105545703	0.701003735
828	3/5/2004 23:16	0	4697718710	205165923.2	-74.421	-42.444	38790	0.472206064	-1.180783853	0.393852903
828	3/6/2004 12:21	B	1445289616	162773088	-74.61	-42.669	47071	1.395305115	-1.133604409	0.495131311
828	3/7/2004 20:38	0	4697718710	205165923.2	-74.483	-42.677	116273	3.912535791	-1.356026504	0.156077528
828	3/8/2004 9:49	2	863491.9002	117318.8964	-74.586	-42.782	47427	0.885540553	-1.427175207	0.559128473
828	3/8/2004 20:26	1	19838678.47	1565421.652	-74.497	-42.681	38218	0.928674674	-1.367871679	0.920854354
828	3/8/2004 23:43	1	19838678.47	1565421.652	-74.48	-42.672	11838	3.00527258	-1.188647094	0.998123868
828	3/9/2004 9:58	B	1445289616	162773088	-74.5	-42.671	36863	2.701564848	-1.108561301	1.11661029
828	3/9/2004 23:20	1	19838678.47	1565421.652	-74.446	-42.626	48121	0.921779757	-1.059569279	1.217388836
828	3/12/2004 23:48	0	4697718710	205165923.2	-74.538	-42.76	260886	0.055845648	-1.520091498	0.797556178
828	3/13/2004 9:29	0	4697718710	205165923.2	-74.679	-42.656	34887	0.136334866	-1.548675333	0.807977362
828	3/13/2004 12:27	B	1445289616	162773088	-74.605	-42.731	10651	0.136334866	-1.543944942	0.807977362
828	3/13/2004 23:27	1	19838678.47	1565421.652	-74.521	-42.686	43202	-0.507362136	-1.578877261	0.762315414
828	3/15/2004 12:02	B	1445289616	162773088	-74.812	-42.766	131717	0.463416668	-1.174584039	0.166671158
828	3/15/2004 20:50	0	4697718710	205165923.2	-74.685	-43.307	31665	-0.079623675	-0.85619027	-0.194817713
828	3/16/2004 9:59	B	1445289616	162773088	-74.695	-43.476	47343	0.040403682	-0.592627718	-0.381693369

828	3/16/2004 20:37	A	1846137731	80281855.35	-74.266	-43.701	38298	-0.631538134	-0.541644229	-0.455145745
828	3/16/2004 23:58	B	1445289616	162773088	-74.465	-43.779	12048	-0.547011255	-0.389836873	-0.432837937
828	3/17/2004 20:22	0	4697718710	205165923.2	-74.221	-43.761	73444	-0.541535346	-0.500179934	-0.132089504
828	3/17/2004 23:30	2	863491.9002	117318.8964	-74.263	-43.778	11263	-0.523864912	-0.52494594	-0.133420804
828	3/18/2004 20:12	B	1445289616	162773088	-74.319	-43.811	74533	-0.505083211	-0.377164038	0.049332322
828	3/18/2004 23:07	A	1846137731	80281855.35	-74.34	-43.877	10517	-0.517058178	-0.330940257	0.033280161
828	3/19/2004 12:13	0	4697718710	205165923.2	-74.598	-43.794	47167	-0.519187152	-0.249653069	0.050975987
828	3/19/2004 20:03	0	4697718710	205165923.2	-74.483	-43.796	28171	-0.52653	-0.228175755	0.2104512
828	3/21/2004 9:26	B	1445289616	162773088	-74.561	-43.888	134612	-0.367876125	-0.205976577	0.333576028
828	3/21/2004 12:28	A	1846137731	80281855.35	-74.488	-43.876	10889	-0.354349862	-0.274492574	0.347662009
828	3/21/2004 23:35	B	1445289616	162773088	-74.215	-43.751	40042	-0.213339855	-0.970611519	0.492581342
828	3/22/2004 12:14	A	1846137731	80281855.35	-74.367	-43.826	45515	-0.237837175	-1.74440345	0.469461559
828	3/22/2004 23:15	A	1846137731	80281855.35	-74.254	-43.631	39660	-0.476105572	-0.630957069	0.415480254
828	3/23/2004 12:19	B	1445289616	162773088	-74.085	-43.62	47029	-0.51277815	-0.971765544	0.43542692
828	3/23/2004 20:53	A	1846137731	80281855.35	-74.062	-43.76	30878	-0.511203073	-0.852364755	0.397197454
828	3/24/2004 20:49	Z	343535924.1	50175612.8	-72.956	-43.345	86151	-0.55155869	-2.278368223	0.384267175

828	3/25/2004 9:46	A	1846137731	80281855.35	-73.088	-43.515	46633	-0.550235894	-1.049149354	0.316254444
828	3/25/2004 20:30	B	1445289616	162773088	-73.202	-43.682	38620	-0.560349249	-1.960682549	-0.733412229
828	3/25/2004 23:43	A	1846137731	80281855.35	-73.074	-43.66	11579	-0.561358217	-1.980348049	-0.735564145
828	3/26/2004 9:27	B	1445289616	162773088	-73.046	-43.582	35051	-0.562840918	-2.236455198	-0.742758729
828	3/26/2004 12:42	B	1445289616	162773088	-73.093	-43.606	11721	-0.564276238	-2.497205553	-0.749020906
828	3/26/2004 20:19	Z	343535924.1	50175612.8	-73.107	-43.652	27395	-0.497617075	-2.943516708	-0.619118569
828	3/26/2004 23:18	1	19838678.47	1565421.652	-73.191	-43.711	10731	-0.497393236	-3.129144509	-0.637235449
828	3/27/2004 12:50	0	4697718710	205165923.2	-73.417	-43.726	48712	-0.489318319	-3.115163629	-0.63949091
828	3/27/2004 20:09	1	19838678.47	1565421.652	-73.554	-43.803	26352	-0.66502474	-0.60115402	-0.69737763
828	3/28/2004 12:37	A	1846137731	80281855.35	-73.448	-43.827	59314	-0.677684274	-0.661497546	-0.70764877
828	3/28/2004 20:02	0	4697718710	205165923.2	-73.571	-43.779	26685	-0.746141992	-0.587985967	-0.667674473
828	3/29/2004 9:54	A	1846137731	80281855.35	-73.605	-43.801	49892	-0.752307323	-0.581573585	-0.670490815
828	3/29/2004 23:49	0	4697718710	205165923.2	-73.382	-43.772	50128	-0.725760016	-0.574360934	-0.684831616
828	3/30/2004 9:28	Z	343535924.1	50175612.8	-73.512	-43.756	34710	-0.721067695	-0.604155182	-0.688581523
828	3/30/2004 12:11	0	4697718710	205165923.2	-73.511	-43.71	9822	-0.715663104	-0.681389207	-0.694507825
828	3/30/2004 23:23	3	102706.2314	10029.44029	-73.354	-43.712	40272	-0.74025902	-1.166518385	-0.789694945

828	3/31/2004 9:10	B	1445289616	162773088	-73.428	-43.334	35246	-0.699867711	-1.182327504	-0.778420546
828	3/31/2004 12:55	A	1846137731	80281855.35	-73.05	-43.63	13509	-0.699867711	-1.181373863	-0.778420546
828	3/31/2004 20:57	0	4697718710	205165923.2	-73.129	-43.643	28889	-0.663485789	-1.421070564	-0.925253306
828	4/1/2004 12:36	0	4697718710	205165923.2	-73.544	-43.441	56344	-0.662167735	-3.006674726	-0.896956769
828	4/2/2004 9:33	0	4697718710	205165923.2	-73.768	-43.227	75439	-0.26310692	-2.04198434	0.194108094
828	4/2/2004 20:39	B	1445289616	162773088	-72.972	-43.197	39982	-0.173092617	-2.446982222	-0.038998267
828	4/3/2004 9:39	A	1846137731	80281855.35	-73.185	-43.698	46749	-0.19278163	-0.838464555	-0.278274093
828	4/3/2004 20:32	0	4697718710	205165923.2	-73.719	-43.731	39221	-0.194614157	-0.782180033	-0.437434389
828	4/3/2004 23:44	B	1445289616	162773088	-73.406	-43.849	11479	-0.200039692	-0.40957076	-0.561138061
828	4/4/2004 12:54	B	1445289616	162773088	-74.446	-43.704	47398	-0.164094072	-0.697375939	-0.611742145
828	4/4/2004 20:18	A	1846137731	80281855.35	-73.604	-43.584	26667	-0.46804044	-0.82994097	-0.731572934
828	4/4/2004 23:08	0	4697718710	205165923.2	-73.641	-43.608	10170	-0.468737334	-0.910330996	-0.740391293
828	4/4/2004 23:29	A	1846137731	80281855.35	-73.64	-43.64	1294	-0.469029346	-0.920289741	-0.743607599
828	4/5/2004 12:09	0	4697718710	205165923.2	-73.463	-43.633	45593	-0.492835647	-1.747302015	-0.826576797
828	4/5/2004 20:14	B	1445289616	162773088	-74.548	-43.609	29080	0.557125013	-1.018785334	-0.528735452
828	4/6/2004 12:29	0	4697718710	205165923.2	-74.874	-43.734	58520	1.720927898	-0.531807093	-1.105904897

828	4/7/2004 23:36	1	19838678.47	1565421.652	-74.858	-43.729	126393	-0.32642077	0.017842612	-1.114471052
828	4/8/2004 9:19	A	1846137731	80281855.35	-73.793	-43.559	34986	3.528826659	-0.672418162	-0.395175494
828	4/9/2004 20:40	A	1846137731	80281855.35	-73.176	-43.839	127301	-0.279630928	-0.428161542	-0.380571632
828	4/10/2004 20:57	A	1846137731	80281855.35	-74.839	-43.806	87415	-0.703370599	-0.311413951	-0.696613387
828	4/11/2004 9:52	B	1445289616	162773088	-74.541	-43.933	46466	-0.35668542	0.014189297	-0.798637445
828	4/12/2004 20:33	B	1445289616	162773088	-75.007	-43.303	124888	2.46123264	-1.363558064	-1.122690546
828	4/12/2004 23:18	A	1846137731	80281855.35	-74.851	-43.31	9911	2.442356295	-1.361728714	-1.129058076
828	4/13/2004 12:21	0	4697718710	205165923.2	-74.858	-43.274	46937	2.607524697	-1.253459128	-1.169058841
828	4/13/2004 20:19	A	1846137731	80281855.35	-74.849	-43.351	28713	0.162450297	-1.240217458	-1.480977585
828	4/14/2004 9:38	B	1445289616	162773088	-75.014	-43.286	47924	1.209115647	-1.219274827	-1.370118302
828	4/14/2004 20:07	A	1846137731	80281855.35	-74.897	-43.184	37773	0.336652184	-1.226352892	-1.389986287
828	4/15/2004 12:09	B	1445289616	162773088	-74.483	-43.283	57706	0.331195108	-1.100862699	-1.364013924
828	4/15/2004 23:46	B	1445289616	162773088	-74.739	-43.151	41796	0.089418106	-1.098933129	-1.549853632
828	4/16/2004 9:29	0	4697718710	205165923.2	-74.808	-43.028	34985	0.141721667	-1.086273901	-1.54769256
828	4/17/2004 9:09	A	1846137731	80281855.35	-74.862	-43.184	85178	-0.338691615	-1.077133893	-1.473724125
828	4/17/2004 12:22	B	1445289616	162773088	-74.487	-43.297	11629	-0.477306135	-1.080836017	-1.485544239

828	4/17/2004 20:42	A	1846137731	80281855.35	-74.625	-43.281	29979	0.144306954	-1.049636519	-1.411842227
828	4/18/2004 9:42	A	1846137731	80281855.35	-74.836	-43.137	46806	2.037583175	-2.510925194	-1.138845807
828	4/19/2004 10:00	0	4697718710	205165923.2	-74.728	-42.888	87460	-0.391467672	-3.183176089	-0.684087704
828	4/19/2004 20:50	A	1846137731	80281855.35	-74.448	-42.796	39011	0.509062045	-1.622332054	-1.128376425
828	4/19/2004 23:56	0	4697718710	205165923.2	-74.324	-42.8	11146	0.669991022	-1.585863491	-1.124573315
828	4/20/2004 9:37	B	1445289616	162773088	-74.616	-42.921	34849	0.994998835	-2.490464186	-1.147986283
828	4/21/2004 9:11	B	1445289616	162773088	-75.005	-43.046	84844	1.392757047	-1.37236716	-1.092984969
828	4/21/2004 23:06	B	1445289616	162773088	-74.846	-42.896	50147	0.959906008	-1.119627161	-1.170267323
828	4/22/2004 9:41	A	1846137731	80281855.35	-75.01	-42.822	38080	0.828956973	-0.86733036	-1.097567811
828	4/22/2004 12:21	1	19838678.47	1565421.652	-75.233	-42.73	9569	2.187190644	-0.501936643	-1.019694828
832	2/13/2004 9:17	1	19838678.47	1565421.652	-73.781	-43.828	34926	1.310833453	-0.577161922	-0.722613376
832	2/13/2004 12:37	A	1846137731	80281855.35	-73.545	-44.133	12013	2.269901278	-0.746113976	-1.750096396
832	2/13/2004 23:10	2	863491.9002	117318.8964	-73.716	-43.839	38002	1.400109083	-0.518128323	-0.36008373
832	2/14/2004 9:12	0	4697718710	205165923.2	-73.463	-43.842	36073	2.700935528	-0.757979082	-0.25638795
832	2/14/2004 12:14	1	19838678.47	1565421.652	-73.533	-43.832	10919	3.884997719	-0.747073659	-0.403096331
832	2/16/2004 9:45	1	19838678.47	1565421.652	-73.364	-43.889	163888	0.157505245	-0.763050051	1.545907835

832	2/16/2004 12:52	Z	343535924.1	50175612.8	-72.994	-44.124	11238	-0.021753636	-1.187655014	1.636951299
832	2/16/2004 20:57	0	4697718710	205165923.2	-73.788	-43.392	29101	-0.523486766	-1.290744272	1.282509327
832	2/16/2004 23:39	2	863491.9002	117318.8964	-73.706	-43.516	9708	-0.512244926	-0.98104399	1.032105605
832	2/17/2004 9:22	1	19838678.47	1565421.652	-73.701	-43.591	34946	-0.50836496	-0.963382134	1.071270677
832	2/17/2004 12:45	1	19838678.47	1565421.652	-73.6	-43.701	12200	-0.489685462	-0.537114419	1.164081434
832	2/17/2004 20:52	1	19838678.47	1565421.652	-73.349	-43.56	29216	-0.50769667	-1.259242683	1.305968704
832	2/17/2004 23:15	2	863491.9002	117318.8964	-73.51	-43.536	8613	-0.438547779	-2.138995873	1.076043134
832	2/18/2004 9:15	B	1445289616	162773088	-73.612	-43.759	35987	-0.410281058	-0.846180642	1.010296975
832	2/18/2004 12:18	1	19838678.47	1565421.652	-73.637	-43.713	10997	-0.396407039	-0.453120231	0.967015153
832	2/18/2004 12:33	1	19838678.47	1565421.652	-73.623	-43.723	889	-0.395016825	-0.434134834	0.964662343
832	2/18/2004 20:40	2	863491.9002	117318.8964	-73.618	-43.699	29216	-0.400395216	-0.515816401	0.967877938
832	2/18/2004 23:12	B	1445289616	162773088	-73.768	-43.69	9077	-0.440804007	-0.489275315	1.089652686
832	2/19/2004 12:14	Z	343535924.1	50175612.8	-73.739	-43.784	46974	-0.410577182	-0.402726951	1.033998218
832	2/19/2004 20:29	1	19838678.47	1565421.652	-73.687	-43.718	29663	-0.415086545	-0.439729686	1.021136172
832	2/20/2004 9:49	1	19838678.47	1565421.652	-73.776	-43.694	48038	0.005938444	-0.562765267	0.135557935
832	2/20/2004 12:07	A	1846137731	80281855.35	-73.752	-43.684	8254	-0.713108124	-0.552179802	0.127295797

832	2/20/2004 20:18	2	863491.9002	117318.8964	-73.447	-43.54	29469	-0.790733157	-1.464640917	0.163569874
832	2/20/2004 23:45	2	863491.9002	117318.8964	-73.636	-43.569	12416	-0.945358281	-1.380762209	0.102442297
832	2/21/2004 9:30	B	1445289616	162773088	-73.602	-43.574	35118	-0.726008946	-0.662279428	0.093379597
832	2/21/2004 12:44	1	19838678.47	1565421.652	-73.783	-43.634	11626	-0.540552229	-0.573087914	0.08283271
832	2/21/2004 20:06	0	4697718710	205165923.2	-73.439	-43.606	26495	-0.839708139	-0.796339629	0.093433067
832	2/21/2004 23:22	2	863491.9002	117318.8964	-73.641	-43.63	11778	-0.568072525	-0.875611399	0.392380683
832	2/22/2004 9:06	A	1846137731	80281855.35	-73.614	-43.661	35022	-0.555617302	-0.795798448	0.398750803
832	2/22/2004 12:25	0	4697718710	205165923.2	-73.697	-43.584	11951	-0.581558789	-0.836966872	0.385407962
832	2/23/2004 12:02	0	4697718710	205165923.2	-73.673	-43.683	85040	-0.500317263	-0.70885677	0.707518073
832	2/24/2004 9:54	3	102706.2314	10029.44029	-73.757	-43.515	78716	-0.900379242	-0.703876839	0.866555755
832	2/24/2004 12:56	0	4697718710	205165923.2	-73.859	-43.494	10908	-0.913669535	-0.570656503	0.859481407
832	2/24/2004 23:50	1	19838678.47	1565421.652	-73.986	-43.443	39263	-0.849001319	-1.51937956	1.082878378
832	2/25/2004 9:33	2	863491.9002	117318.8964	-74.088	-43.49	34953	-0.799562535	-1.352233516	1.080404564
832	2/25/2004 12:42	3	102706.2314	10029.44029	-74.169	-43.475	11326	-0.751662299	-0.962362693	1.10154508
832	2/25/2004 12:54	2	863491.9002	117318.8964	-74.182	-43.475	728	-0.748235119	-0.95290775	1.103354038
832	2/25/2004 23:28	1	19838678.47	1565421.652	-74.301	-43.467	38041	-0.705538038	-1.159164252	1.217874226

832	2/26/2004 9:11	1	19838678.47	1565421.652	-74.2	-43.484	35008	-0.716700572	-1.174115885	1.195222187
832	2/26/2004 12:30	1	19838678.47	1565421.652	-74.306	-43.459	11912	-0.706585862	-1.054278115	1.214702722
832	2/26/2004 20:50	1	19838678.47	1565421.652	-74.153	-43.418	30012	-0.722059551	-0.868514591	1.196978045
832	2/26/2004 23:04	1	19838678.47	1565421.652	-74.163	-43.493	8062	-0.779628323	-0.975460803	1.152234032
832	2/27/2004 12:08	A	1846137731	80281855.35	-73.835	-43.534	47026	-0.812716945	-0.942955177	1.122610327
832	2/27/2004 20:40	0	4697718710	205165923.2	-74.203	-43.609	30723	-0.849621737	-0.708434106	1.100078777
832	2/28/2004 12:07	Z	343535924.1	50175612.8	-74.235	-43.982	55611	-0.922452232	-0.386446573	1.024775173
832	2/28/2004 20:28	1	19838678.47	1565421.652	-74.306	-43.986	30043	-0.900787759	-0.267063072	1.013200613
832	2/28/2004 23:56	0	4697718710	205165923.2	-74.252	-43.918	12471	-0.855577323	-0.127167997	0.843307657
832	2/29/2004 9:40	0	4697718710	205165923.2	-74.849	-44.276	35082	-0.863830517	-0.041450982	0.766751329
832	2/29/2004 13:00	B	1445289616	162773088	-74.858	-44.27	12003	-0.96002978	-0.101576521	0.762385027
832	2/29/2004 20:17	1	19838678.47	1565421.652	-75.064	-44.345	26201	-0.824930541	-0.115890852	0.718564896
832	2/29/2004 23:32	1	19838678.47	1565421.652	-75.122	-44.38	11693	-0.226876221	-0.227605902	0.264893736
832	3/1/2004 9:16	0	4697718710	205165923.2	-75.106	-44.322	35047	-0.293157142	-0.223016825	0.331946303
832	3/1/2004 12:36	1	19838678.47	1565421.652	-75.12	-44.318	11981	-0.195836885	-0.207807696	0.355963509
832	3/1/2004 20:03	1	19838678.47	1565421.652	-74.953	-44.282	26847	0.361570367	-0.039280085	0.464549798

832	3/1/2004 23:08	1	19838678.47	1565421.652	-74.925	-44.38	11090	-0.619148515	-0.275905723	-0.723496018
832	3/2/2004 12:13	B	1445289616	162773088	-75.017	-44.297	47079	0.560228749	-0.117481713	-0.618380771
832	3/2/2004 23:46	1	19838678.47	1565421.652	-74.932	-44.327	41625	0.037214102	-0.155547848	-0.668652494
832	3/3/2004 12:54	1	19838678.47	1565421.652	-74.989	-44.403	47233	-0.473590335	-0.217139138	-0.73332688
832	3/3/2004 23:35	A	1846137731	80281855.35	-75.23	-44.563	38492	0.070049071	-0.907244919	-0.656430317
832	3/4/2004 9:49	B	1445289616	162773088	-75.319	-44.63	36814	-0.516325203	-1.345912951	-0.662066767
832	3/4/2004 12:41	0	4697718710	205165923.2	-75.343	-44.597	10317	-0.593680941	-1.320925924	-0.664425067
832	3/4/2004 23:39	1	19838678.47	1565421.652	-75.382	-44.765	39516	0.115440195	-1.109153263	-0.928876622
832	3/5/2004 9:20	B	1445289616	162773088	-75.217	-44.526	34867	-0.281009271	-0.269250944	-0.594164634
832	3/5/2004 12:30	1	19838678.47	1565421.652	-74.831	-44.717	11395	-0.551030913	-0.33881756	-0.643575933
832	3/5/2004 20:57	1	19838678.47	1565421.652	-74.538	-44.862	30425	-0.770239345	0.024675614	-0.682284739
832	3/5/2004 23:13	0	4697718710	205165923.2	-74.596	-44.811	8138	-0.747601168	0.02072565	-0.620331683
832	3/6/2004 12:18	1	19838678.47	1565421.652	-74.964	-45.038	47079	-0.611319263	0.205952973	-0.779769723
832	3/6/2004 20:48	1	19838678.47	1565421.652	-74.604	-45.547	30635	-0.847398805	-1.982620362	-0.904525633
832	3/7/2004 12:04	B	1445289616	162773088	-74.402	-45.407	54947	0.133670436	-1.658059487	-1.804939506
832	3/7/2004 20:38	0	4697718710	205165923.2	-74.555	-44.816	30840	0.671057058	0.162973379	-2.300796179

832	3/8/2004 9:48	2	863491.9002	117318.8964	-74.805	-44.157	47397	-0.068174038	0.121688781	-1.745241662
832	3/8/2004 20:23	1	19838678.47	1565421.652	-74.556	-44.065	38086	1.684768286	0.162433431	-1.61359693
832	3/8/2004 23:45	1	19838678.47	1565421.652	-74.509	-44.11	12127	1.069184963	0.10629754	-1.948935697
832	3/9/2004 9:27	0	4697718710	205165923.2	-74.403	-44.116	34937	-0.480702227	-0.549939834	-2.330205079
832	3/9/2004 20:14	A	1846137731	80281855.35	-73.973	-43.625	38829	2.76210335	-0.651466067	-2.098961464
832	3/9/2004 23:20	2	863491.9002	117318.8964	-73.758	-43.694	11142	2.55465296	-0.57294365	-2.220391362
832	3/10/2004 12:24	0	4697718710	205165923.2	-73.24	-43.465	47043	1.019411494	-1.042436284	-2.620170924
832	3/10/2004 20:07	Z	343535924.1	50175612.8	-73.558	-43.27	27789	0.5237686	-0.999253205	-2.348045301
832	3/10/2004 23:42	B	1445289616	162773088	-73.702	-43.557	12868	0.022022334	-0.735212647	-2.524893207
832	3/11/2004 12:52	0	4697718710	205165923.2	-74.697	-43.374	47442	-0.291191061	-0.525721582	-1.140620876
832	3/11/2004 20:47	0	4697718710	205165923.2	-74.457	-43.1	28484	3.18560488	-2.433987024	0.009502736
832	3/11/2004 23:34	B	1445289616	162773088	-74.447	-43.068	10006	0.796969236	-2.855522268	-0.05942835
832	3/12/2004 9:53	1	19838678.47	1565421.652	-74.457	-42.77	37143	1.280635974	-1.132470935	0.153711873
832	3/12/2004 12:43	1	19838678.47	1565421.652	-74.423	-42.766	10214	1.512325868	-1.095703353	0.163964823
832	3/12/2004 23:20	B	1445289616	162773088	-74.526	-42.774	38194	-0.239071965	-1.352311763	-0.027774851
832	3/12/2004 23:51	0	4697718710	205165923.2	-74.514	-42.76	1853	-0.229314908	-1.429456795	-0.049083322

832	3/13/2004 9:33	2	863491.9002	117318.8964	-74.523	-42.945	34964	0.139703284	-3.20668341	-0.19207974
832	3/13/2004 12:29	2	863491.9002	117318.8964	-74.512	-42.889	10531	-0.115624952	-2.305302821	-0.106535169
832	3/13/2004 23:28	1	19838678.47	1565421.652	-74.538	-42.741	43148	-0.543410124	-2.166820582	0.052563321
832	3/14/2004 12:45	0	4697718710	205165923.2	-74.28	-42.812	47838	-0.598422413	-1.18629165	0.019587569
832	3/14/2004 20:56	A	1846137731	80281855.35	-74.24	-42.626	29459	-0.200066439	-1.869170341	-0.411920085
832	3/14/2004 23:05	A	1846137731	80281855.35	-74.31	-42.55	7703	-0.220644433	-1.779588037	-0.441315081
832	3/15/2004 12:05	1	19838678.47	1565421.652	-74.289	-42.56	46796	-0.366643666	-2.308890293	-0.539844759
832	3/15/2004 20:47	1	19838678.47	1565421.652	-74.244	-42.569	31317	0.31869999	-2.86539442	-0.376352566
832	3/16/2004 10:00	0	4697718710	205165923.2	-74.439	-42.783	47608	0.117595891	-3.012351232	-0.346811666
832	3/16/2004 20:37	1	19838678.47	1565421.652	-74.493	-42.821	38225	2.196770053	-1.373705018	-0.439784549
832	3/16/2004 23:55	1	19838678.47	1565421.652	-74.518	-42.82	11865	1.495351618	-1.309408722	-0.601703493
832	3/17/2004 9:37	1	19838678.47	1565421.652	-74.69	-42.704	34939	2.262179888	-1.569697174	0.187616395
832	3/17/2004 20:26	2	863491.9002	117318.8964	-74.202	-42.519	38926	0.512976304	-2.320077259	-0.138096414
832	3/17/2004 23:31	1	19838678.47	1565421.652	-74.325	-42.495	11128	0.145579089	-2.164097336	-0.167576423
832	3/17/2004 23:56	0	4697718710	205165923.2	-74.275	-42.458	1492	0.316143781	-2.069961193	-0.164033374
832	3/18/2004 9:14	1	19838678.47	1565421.652	-74.365	-42.712	33472	-0.290775089	-1.015856552	0.073445234

832	3/18/2004 12:32	0	4697718710	205165923.2	-74.488	-42.861	11868	1.142860825	-1.396465301	-0.074980872
832	3/18/2004 12:55	B	1445289616	162773088	-74.555	-42.885	1405	1.211244983	-1.430430103	-0.091190497
832	3/18/2004 20:11	1	19838678.47	1565421.652	-74.476	-42.851	26157	-0.245881474	-1.670050815	0.135342055
832	3/18/2004 23:08	1	19838678.47	1565421.652	-74.55	-42.812	10592	-0.363803421	-1.325839278	0.096027592
832	3/19/2004 12:53	1	19838678.47	1565421.652	-74.592	-42.829	49490	-0.092823624	-1.063877655	0.121027976
832	3/19/2004 20:53	A	1846137731	80281855.35	-74.643	-42.924	28790	-0.388105333	-2.062898597	0.291908638
832	3/19/2004 23:30	B	1445289616	162773088	-74.585	-42.909	9465	-0.273783157	-2.22188739	0.300892373
3040	2/19/2004 12:14	A	1846137731	80281855.35	-73.425	-43.782	46817	-0.31416261	-0.698233393	1.529909102
3040	2/19/2004 20:30	1	19838678.47	1565421.652	-73.537	-43.647	29742	-0.293457019	-0.833880191	1.576081976
3040	2/20/2004 9:51	1	19838678.47	1565421.652	-73.643	-43.617	48044	-0.616540116	-0.952600219	0.6595289
3040	2/20/2004 12:06	0	4697718710	205165923.2	-73.705	-43.573	8091	-0.618866299	-1.296147999	0.660310435
3040	2/20/2004 20:18	2	863491.9002	117318.8964	-73.534	-43.52	29535	-0.543275559	-2.81508259	0.677926003
3040	2/20/2004 23:46	1	19838678.47	1565421.652	-73.657	-43.574	12481	-0.60433773	-1.19739881	0.640256616
3040	2/21/2004 9:27	0	4697718710	205165923.2	-73.529	-43.608	34874	-0.562114063	-1.142973618	0.658869093
3040	2/21/2004 12:48	A	1846137731	80281855.35	-73.473	-43.645	12075	-0.58379067	-0.776810902	0.671840011
3040	2/21/2004 20:06	1	19838678.47	1565421.652	-73.455	-43.428	26281	-0.511493867	-2.394263809	0.696478897

3040	2/21/2004 23:20	1	19838678.47	1565421.652	-73.557	-43.465	11605	-0.5380523	-2.095356335	0.83445277
3040	2/22/2004 9:05	A	1846137731	80281855.35	-73.432	-43.564	35135	-0.496516651	-1.375300355	0.876519061
3040	2/22/2004 12:23	2	863491.9002	117318.8964	-73.616	-43.641	11864	-0.35033376	-0.845583704	0.904548129
3040	2/22/2004 19:59	B	1445289616	162773088	-73.156	-43.734	27366	-0.478870622	-3.152491693	0.897354254
3040	2/23/2004 12:02	0	4697718710	205165923.2	-73.796	-43.642	57765	-0.388288487	-0.562940031	1.131925054
3040	2/24/2004 9:54	1	19838678.47	1565421.652	-73.527	-43.569	78735	-0.52692942	-1.69031 46	1.357030498
3040	2/24/2004 12:54	1	19838678.47	1565421.652	-73.551	-43.647	10764	-0.486617205	-0.835832309	1.367256394
3040	2/24/2004 23:50	2	863491.9002	117318.8964	-73.528	-43.628	39401	-0.536499975	-0.961569589	1.496871344
3040	2/25/2004 9:33	1	19838678.47	1565421.652	-73.52	-43.572	34947	-0.527693413	-1.56895999	1.509209925
3040	2/25/2004 12:43	3	102706.2314	10029.44029	-73.575	-43.634	11405	-0.547918858	-0.931248944	1.484548123
3040	2/25/2004 12:51	1	19838678.47	1565421.652	-73.573	-43.636	512	-0.548018721	-0.914839574	1.48430592
3040	2/25/2004 23:26	2	863491.9002	117318.8964	-73.518	-43.715	38079	-0.488200227	-0.489409543	1.449040318
3040	2/26/2004 9:20	2	863491.9002	117318.8964	-73.395	-43.724	35641	-0.442114899	-0.866992137	1.504361457
3040	2/26/2004 12:28	A	1846137731	80281855.35	-73.516	-43.745	11296	-0.502971615	-0.450996549	1.447348975
3040	2/26/2004 20:51	2	863491.9002	117318.8964	-73.711	-43.701	30167	-0.536573553	-0.480497245	1.416539092
3040	2/26/2004 23:03	1	19838678.47	1565421.652	-73.636	-43.72	7917	-0.555143624	-0.426179727	1.452920121

3040	2/27/2004 12:06	0	4697718710	205165923.2	-73.701	-43.714	46943	-0.557237479	-0.454561538	1.450582881
3040	2/27/2004 12:18	1	19838678.47	1565421.652	-73.679	-43.705	773	-0.557237479	-0.459337648	1.450582881
3040	2/27/2004 20:38	1	19838678.47	1565421.652	-73.678	-43.695	29989	-0.545803479	-0.491749371	1.453030133
3040	2/28/2004 12:07	0	4697718710	205165923.2	-73.684	-43.63	55698	-0.558558213	-0.748429281	1.479443948
3040	2/28/2004 20:28	1	19838678.47	1565421.652	-73.737	-43.71	30096	-0.60537869	-0.52419688	1.463772232
3040	2/28/2004 23:56	0	4697718710	205165923.2	-73.694	-43.732	12468	-0.590901786	-0.526455949	1.270229579
3040	2/29/2004 9:40	1	19838678.47	1565421.652	-73.732	-43.548	35037	-0.562833067	-0.823469702	1.285826163
3040	2/29/2004 12:59	B	1445289616	162773088	-73.308	-43.529	11937	-0.497512541	-1.710784516	1.396875814
3040	2/29/2004 20:16	1	19838678.47	1565421.652	-73.612	-43.412	26232	-0.531438788	-1.624532284	1.33439262
3040	2/29/2004 23:33	1	19838678.47	1565421.652	-73.602	-43.386	11831	-0.549597725	-2.008806825	1.060979982
3040	3/1/2004 9:16	1	19838678.47	1565421.652	-73.555	-43.528	34981	-0.551287476	-3.044708428	1.033927505
3040	3/1/2004 12:35	1	19838678.47	1565421.652	-73.48	-43.597	11945	-0.549376489	-1.10999343	1.02502557
3040	3/1/2004 20:04	0	4697718710	205165923.2	-73.511	-43.409	26946	-0.545710881	-2.835082628	1.068448698
3040	3/1/2004 23:08	0	4697718710	205165923.2	-73.497	-43.392	11034	-0.467723599	-3.049697197	0.813979677
3040	3/2/2004 8:59	0	4697718710	205165923.2	-73.446	-43.418	35443	-0.465086077	-2.318477165	0.817614195
3040	3/2/2004 12:10	0	4697718710	205165923.2	-73.479	-43.38	11483	-0.464975255	-2.931957537	0.825026326

3040	3/3/2004 12:53	0	4697718710	205165923.2	-73.685	-43.504	88964	-0.401810683	-1.184299948	0.757536942
3040	3/3/2004 20:43	B	1445289616	162773088	-73.573	-43.43	28214	-0.386514684	-1.932210462	0.840937492
3040	3/3/2004 23:34	A	1846137731	80281855.35	-73.533	-43.429	10222	-0.398558929	-2.193649087	0.751011261
3040	3/4/2004 9:44	1	19838678.47	1565421.652	-73.792	-43.545	36636	-0.454216798	-0.585191587	0.590486945
3040	3/4/2004 12:41	1	19838678.47	1565421.652	-73.808	-43.638	10594	-0.464853755	-0.638756626	0.554372334
3040	3/4/2004 23:40	1	19838678.47	1565421.652	-73.704	-43.681	39552	-0.430572079	-0.525379897	0.227422934
3040	3/5/2004 12:30	0	4697718710	205165923.2	-73.944	-43.613	46174	-0.444301253	-1.095294479	0.117953199
3040	3/5/2004 20:58	0	4697718710	205165923.2	-73.75	-43.525	30505	-0.399509135	-0.861542954	0.249809181
3040	3/5/2004 23:14	2	863491.9002	117318.8964	-73.654	-43.56	8152	-0.421458015	-1.292512655	0.171047714
3040	3/6/2004 12:15	2	863491.9002	117318.8964	-73.713	-43.526	46880	-0.418471029	-0.924632544	0.14846077
3040	3/6/2004 20:49	0	4697718710	205165923.2	-73.611	-43.528	30815	-0.406831428	-2.267639925	0.205903206
3040	3/7/2004 12:04	0	4697718710	205165923.2	-73.338	-43.297	54885	0.164495658	-3.182672316	-1.727117826
3040	3/7/2004 20:37	2	863491.9002	117318.8964	-73.462	-43.329	30826	-0.1155309	-3.20668341	-1.837338962
3040	3/7/2004 20:47	2	863491.9002	117318.8964	-73.454	-43.346	585	-0.03110564	-3.20668341	-1.813874099
3040	3/8/2004 9:51	1	19838678.47	1565421.652	-73.646	-43.54	47059	-0.152292845	-1.472194102	-1.841084533
3040	3/8/2004 20:28	A	1846137731	80281855.35	-73.509	-43.434	38218	-0.279773724	-2.221018884	-1.824423235

3040	3/8/2004 23:43	1	19838678.47	1565421.652	-73.569	-43.582	11700	0.452565575	-1.553584359	-1.390132885
3040	3/9/2004 9:26	2	863491.9002	117318.8964	-73.641	-43.642	34962	0.185178775	-0.801692001	-1.477889295
3040	3/9/2004 12:46	B	1445289616	162773088	-73.451	-43.664	11972	0.760056676	-0.69771949	-1.212760441
3040	3/9/2004 20:15	A	1846137731	80281855.35	-73.516	-43.438	26952	0.250489618	-2.213738555	-1.465425575
3040	3/9/2004 23:21	1	19838678.47	1565421.652	-73.5	-43.497	11158	0.284033651	-2.013072932	-1.167867737
3040	3/10/2004 9:01	B	1445289616	162773088	-73.527	-43.387	34790	1.78026423	-3.20668341	-1.546727521
3040	3/10/2004 12:35	B	1445289616	162773088	-73.5	-43.431	12881	0.849550578	-2.445312385	-1.283228182
3040	3/10/2004 20:06	0	4697718710	205165923.2	-73.48	-43.297	27020	1.996674329	-2.851029226	-1.243186984
3040	3/10/2004 23:45	A	1846137731	80281855.35	-73.314	-43.252	13187	0.366204515	-3.20668341	-1.193794768
3040	3/11/2004 12:50	B	1445289616	162773088	-73.399	-43.267	47068	0.293598206	-2.836408194	-1.203863231
3040	3/11/2004 20:48	B	1445289616	162773088	-73.426	-43.354	28692	0.700306947	-3.20668341	-1.297531489
3040	3/11/2004 23:32	A	1846137731	80281855.35	-73.449	-43.428	9828	0.144660735	-2.38011078	-1.28079325
3040	3/12/2004 9:55	B	1445289616	162773088	-73.676	-43.538	37410	-0.269311226	-1.177377248	-1.623321268
3040	3/12/2004 12:41	2	863491.9002	117318.8964	-73.702	-43.622	9961	0.687969774	-0.738559038	-1.582628255
3040	3/12/2004 20:25	B	1445289616	162773088	-73.684	-43.714	27788	2.511310284	-0.440967849	-1.442823185
3040	3/12/2004 23:51	0	4697718710	205165923.2	-73.698	-43.773	12360	3.925832278	-0.409935286	-0.85748808

3040	3/13/2004 9:32	1	19838678.47	1565421.652	-73.609	-43.659	34861	0.753547895	-0.731299373	-1.678179235
3040	3/13/2004 12:29	1	19838678.47	1565421.652	-73.6	-43.727	10623	2.644749669	-0.414275601	-1.491673482
3040	3/13/2004 12:50	B	1445289616	162773088	-73.578	-43.716	1289	2.524918171	-0.41027297	-1.49462425
3040	3/13/2004 23:27	0	4697718710	205165923.2	-73.43	-43.757	41809	-0.534416076	-0.647213539	0.01585887
3040	3/14/2004 12:18	0	4697718710	205165923.2	-73.632	-43.683	46251	-0.525924218	-0.646763098	-0.006277351
3040	3/14/2004 12:46	B	1445289616	162773088	-73.548	-43.671	1670	-0.526713569	-0.667097899	-0.009163743
3040	3/14/2004 20:55	0	4697718710	205165923.2	-73.991	-43.704	29356	-0.411355487	-1.909987614	-0.544569197
3040	3/15/2004 12:07	0	4697718710	205165923.2	-73.696	-43.506	54708	-0.442146257	-0.854197957	-0.408106949
3040	3/15/2004 20:45	1	19838678.47	1565421.652	-73.939	-43.569	31074	-0.275007465	-0.892880078	-0.527156332
3040	3/16/2004 10:01	A	1846137731	80281855.35	-73.987	-43.481	47757	-0.461150906	-1.483228042	-0.439175423
3040	3/16/2004 20:35	0	4697718710	205165923.2	-74.389	-43.427	38069	0.007555637	-0.826111261	-0.292078532
3040	3/16/2004 23:55	0	4697718710	205165923.2	-74.291	-43.551	11977	-0.135148125	-1.980037822	-0.516073152
3040	3/17/2004 9:39	B	1445289616	162773088	-74.885	-43.37	35052	-0.032403895	-1.94345393	-0.301486576
3040	3/17/2004 20:25	1	19838678.47	1565421.652	-74.728	-43.5	38742	-0.213918838	-0.590418758	-0.39111264
3040	3/17/2004 23:30	1	19838678.47	1565421.652	-74.777	-43.489	11150	-0.119525276	-0.733824971	-0.37141388
3040	3/17/2004 23:57	A	1846137731	80281855.35	-74.758	-43.487	1572	-0.100730023	-0.755741192	-0.365507542

3040	3/18/2004 9:05	0	4697718710	205165923.2	-74.965	-43.549	32894	-0.055863836	-0.570617844	-0.425358819
3040	3/18/2004 12:36	A	1846137731	80281855.35	-74.784	-43.641	12700	-0.54599373	-0.164486022	-0.489772182
3040	3/18/2004 20:12	0	4697718710	205165923.2	-74.919	-43.57	27308	-0.382636936	-0.47813098	-0.013032149
3040	3/18/2004 23:08	0	4697718710	205165923.2	-74.916	-43.575	10585	-0.36900016	-0.498749867	-0.007560601
3040	3/19/2004 12:53	2	863491.9002	117318.8964	-74.975	-43.528	49498	-0.22389479	-0.790406645	0.055296805
3040	3/19/2004 20:05	0	4697718710	205165923.2	-74.92	-43.603	25912	-0.278836244	-0.390846702	0.149140586
3040	3/20/2004 12:39	1	19838678.47	1565421.652	-74.957	-43.585	59679	-0.228453943	-0.471401439	0.175218717
3040	3/20/2004 20:28	A	1846137731	80281855.35	-74.881	-43.491	28081	-0.353084349	-0.787844539	0.319987927
3040	3/20/2004 23:57	Z	343535924.1	50175612.8	-74.797	-43.589	12591	-0.47364822	-0.312464547	0.264075796
3040	3/21/2004 9:45	2	863491.9002	117318.8964	-74.947	-43.591	35237	-0.389090532	-0.445617991	0.252959416
3040	3/21/2004 12:27	1	19838678.47	1565421.652	-74.896	-43.616	9732	-0.422436529	-0.318648337	0.242585759
3040	3/21/2004 23:38	0	4697718710	205165923.2	-74.858	-43.71	40245	-0.347750719	-0.03100206	0.132765027
3040	3/22/2004 9:19	A	1846137731	80281855.35	-74.887	-43.585	34866	-0.27548875	-0.412941614	0.268029295
3040	3/22/2004 12:14	0	4697718710	205165923.2	-74.929	-43.661	10518	-0.148544845	-0.200767351	0.156637802
3040	3/22/2004 23:12	0	4697718710	205165923.2	-74.898	-43.492	39503	-0.120905086	-0.834841516	0.318310122
3040	3/23/2004 12:39	A	1846137731	80281855.35	-74.736	-43.734	48404	-0.358911961	0.04820906	0.137868856

3040	3/23/2004 20:57	A	1846137731	80281855.35	-74.599	-43.418	29861	0.365769995	-0.480803149	0.807324791
3040	3/24/2004 20:43	2	863491.9002	117318.8964	-74.569	-43.474	85592	0.752953599	-0.433763127	0.627840777
3040	3/25/2004 9:29	B	1445289616	162773088	-74.604	-43.478	45956	0.701102414	-0.461507927	0.601219766
3040	3/25/2004 9:49	2	863491.9002	117318.8964	-74.554	-43.487	1176	0.687851759	-0.478895994	0.592726669
3040	3/25/2004 20:32	1	19838678.47	1565421.652	-74.469	-43.465	38603	5.780609863	-0.712887447	-0.038892915
3040	3/25/2004 23:44	1	19838678.47	1565421.652	-74.466	-43.466	11515	5.784933845	-0.724301648	-0.0522691
3040	3/26/2004 9:08	1	19838678.47	1565421.652	-74.966	-43.47	33833	1.207692015	-1.265144106	0.049898582
3040	3/26/2004 9:24	0	4697718710	205165923.2	-74.975	-43.431	962	1.264988024	-1.338457418	0.053253101
3040	3/26/2004 12:45	A	1846137731	80281855.35	-74.699	-43.397	12053	0.811825316	-1.357373311	-0.023712622
3040	3/26/2004 20:23	1	19838678.47	1565421.652	-74.989	-43.489	27453	0.414859849	-1.100829212	-0.307859729
3040	3/26/2004 23:20	A	1846137731	80281855.35	-74.936	-43.463	10674	0.332634003	-1.261042365	-0.256144533
3040	3/27/2004 12:20	0	4697718710	205165923.2	-74.742	-43.473	46790	0.218833693	-0.719469004	-0.343035351
3040	3/27/2004 12:52	0	4697718710	205165923.2	-74.827	-43.546	1932	0.10793686	-0.57168321	-0.413109924
3040	3/27/2004 20:12	1	19838678.47	1565421.652	-74.95	-43.434	26370	-0.466087258	-1.567553762	-0.415130802
3040	3/28/2004 12:42	0	4697718710	205165923.2	-74.905	-43.469	59391	-0.401010266	-0.91281856	-0.440832522
3040	3/28/2004 20:03	0	4697718710	205165923.2	-74.237	-43.516	26489	0.050299063	-2.417036742	-0.176514013

3040	3/29/2004 9:54	2	863491.9002	117318.8964	-74.718	-43.478	49872	-0.21677085	-0.672250514	-0.221067182
3040	3/29/2004 12:25	0	4697718710	205165923.2	-74.764	-43.491	9050	-0.276378064	-0.722752192	-0.271724349
3040	3/29/2004 23:49	1	19838678.47	1565421.652	-74.835	-43.46	41023	-0.390861922	-0.967166235	-0.315417439
3040	3/30/2004 9:30	1	19838678.47	1565421.652	-74.85	-43.456	34881	-0.425150825	-1.007988703	-0.318785559
3040	3/30/2004 12:13	B	1445289616	162773088	-75.125	-43.247	9746	0.495400084	-3.166924859	-0.199664526
3040	3/30/2004 23:26	A	1846137731	80281855.35	-74.781	-43.425	40365	-0.304410294	-1.255198897	-0.396521491
3040	3/31/2004 9:08	0	4697718710	205165923.2	-74.44	-43.313	34939	-0.519567551	-0.834834817	-0.216268504
3040	3/31/2004 23:03	A	1846137731	80281855.35	-74.072	-43.671	50128	-0.255805786	-1.18168581	-0.820695349
3040	4/1/2004 10:03	A	1846137731	80281855.35	-74.839	-43.521	39564	-0.394392588	-0.592746789	-0.713816104
3040	4/1/2004 20:57	Z	343535924.1	50175612.8	-74.37	-43.784	39254	-0.123028685	-0.251470472	-0.641838951
3040	4/2/2004 20:43	2	863491.9002	117318.8964	-74.473	-44.138	85560	-0.351232071	0.07010569	-1.381792426
3040	4/2/2004 23:53	0	4697718710	205165923.2	-74.555	-44.172	11403	-0.420069013	0.094563386	-1.410874223
3040	4/3/2004 9:10	0	4697718710	205165923.2	-74.503	-44.221	33441	-0.471401645	0.008511078	-1.437841307
3040	4/3/2004 20:30	1	19838678.47	1565421.652	-74.54	-44.114	40771	-0.294390609	0.146670721	-1.472399214
3040	4/3/2004 23:31	2	863491.9002	117318.8964	-74.48	-44.129	10851	-0.302365266	0.073095313	-1.484945014
3040	4/4/2004 20:22	B	1445289616	162773088	-75.039	-44.533	75078	-0.062050822	-0.340756163	-1.628850903

3040	4/4/2004 23:07	B	1445289616	162773088	-75.034	-44.625	9923	0.089001477	-0.324184189	-1.582925139
3040	4/6/2004 12:23	0	4697718710	205165923.2	-74.57	-43.366	134120	0.751851626	-0.455506329	-0.382559291
87759	4/1/2013 6:46	3	172718.2295	11330.77052	-73.19	-43.174	300	2.351729077	-1.127800141	0.313156514
87759	4/1/2013 7:21	B	8280037.618	2791476.882	-73.182	-43.189	1800	1.32240789	-1.248110716	0.390275593
87759	4/1/2013 8:37	2	240746.0245	4584.475463	-73.09	-43.144	4560	1.795447924	-1.135048007	0.197357407
87759	4/1/2013 9:05	1	5458568.428	726791.5717	-73.176	-43.147	1680	2.74030619	-0.914663705	0.116905382
87759	4/1/2013 9:37	1	1685535.187	2874693.313	-73.122	-43.163	1920	1.528504792	-1.211793877	0.253341526
87759	4/1/2013 10:43	B	48579203.13	12400441.37	-73.087	-43.175	3960	0.564388691	-1.48927111	0.343498803
87759	4/1/2013 11:11	B	47915951.34	7094057.661	-73.087	-43.201	1680	-0.256262732	-1.811264197	0.401326795
87759	4/1/2013 11:34	B	67628088.1	10226172.9	-73.078	-43.207	1380	-0.419448008	-1.976737135	0.408598846
87759	4/1/2013 19:13	1	90768531.12	5651766.875	-73.181	-43.182	27540	1.832224876	-1.226695515	0.350862434
87759	4/1/2013 20:50	B	147616410.9	22047335.62	-73.2	-43.157	5820	3.058243488	-0.982626785	0.190956576
87759	4/1/2013 21:55	B	11653018.83	1686581.165	-73.178	-43.155	3900	4.849733063	-0.996377673	0.26976652
87759	4/1/2013 22:33	B	7497807.765	2433217.235	-73.12	-43.137	2280	3.477384495	-0.984931223	0.246562339
87759	4/1/2013 22:55	A	300347.5762	519062.9238	-73.132	-43.182	1320	1.320879002	-1.31731623	0.49753195
87759	4/1/2013 23:32	A	61478.30188	90605.69812	-73.071	-43.187	2220	-0.171393552	-1.795150682	0.453328556

87759	4/2/2013 6:40	B	6141113.287	946936.7135	-72.948	-43.155	25680	-0.384912841	-2.271628798	0.443760989
87759	4/2/2013 10:54	B	1588914838	98774888.78	-72.959	-43.172	15240	-0.479919221	-2.538655427	0.457268542
87759	4/2/2013 19:09	B	10745260227	487078037.8	-73.064	-43.264	29700	-0.008137769	-2.469380852	0.494660036
87759	4/2/2013 20:40	A	32837890.71	267966.2916	-73.278	-43.286	5460	-0.12206193	-0.994090136	0.631725483
87759	4/2/2013 22:30	1	5834825.663	959422.8371	-73.269	-43.304	6600	0.187124704	-0.952427327	0.423453252
87759	4/2/2013 23:09	1	511052.707	1653148.293	-73.238	-43.315	2340	-0.259109086	-1.028112667	0.440733312
87759	4/3/2013 6:29	A	671571.1864	36967121.81	-73.168	-43.538	26400	-0.040157288	-0.25887874	0.622271629
87759	4/3/2013 8:09	B	1962597351	74155761.64	-73.194	-43.608	6000	0.252716194	-0.341279737	0.711902334
87759	4/3/2013 8:56	B	1563246947	85407653.46	-73.161	-43.618	2820	0.339558191	-0.369527477	0.799457735
87759	4/3/2013 9:55	2	417573.3486	8574.651412	-73.163	-43.665	3540	0.194616382	-0.538368269	0.899588603
87759	4/3/2013 10:46	B	5397588.619	746933.8812	-73.134	-43.632	3060	0.27643655	-0.425884836	0.879937764
87759	4/3/2013 18:50	0	196175175.4	674157.6303	-73.182	-43.585	29040	0.001677803	-0.286188681	0.691807076
87759	4/3/2013 20:27	A	24751331.77	84796398.73	-73.234	-43.549	5820	-0.65154186	-0.221267067	0.592161915
87759	4/3/2013 22:11	B	93448.35381	133305.6462	-73.144	-43.549	6240	0.386461786	-0.259005656	0.590830854
87759	4/3/2013 22:44	1	810243.8466	1025438.653	-73.096	-43.564	1980	0.072669846	-0.259627535	0.739651679
87759	4/3/2013 23:47	A	15580484.62	24653811.88	-73.08	-43.573	3780	-0.062779683	-0.27193377	0.784505136

87759	4/4/2013 6:19	B	2234498699	119104573.7	-73.23	-43.416	23520	-0.683769122	-0.538956249	0.322289412
87759	4/4/2013 7:49	B	1129847508	23306117.39	-73.21	-43.346	5400	-0.739876547	-0.929424993	0.31860978
87759	4/4/2013 7:59	B	300745979.6	5363762.851	-73.208	-43.346	600	-0.750359055	-0.925148451	0.321526838
87759	4/4/2013 9:27	B	316157148.3	4244253.711	-73.029	-43.289	5280	-0.146182244	-1.670148411	0.375482662
87759	4/4/2013 9:37	B	653969086.8	14450793.72	-73.011	-43.28	600	-0.033249154	-1.741734982	0.362377926
87759	4/4/2013 10:07	B	266088113.4	88734571.12	-72.94	-43.265	1800	0.185152836	-1.903127994	0.367136855
87759	4/4/2013 11:48	B	38070674.57	6566447.925	-72.779	-43.206	6060	0	-0.823912739	0
87759	4/4/2013 18:45	A	1414406.015	34389506.48	-72.958	-43.031	25020	0.422096232	-2.025375549	0.269886304
87759	4/4/2013 20:22	B	1630988028	32217029.16	-72.865	-42.976	5820	-0.016644406	-1.930223123	0.625174383
87759	4/4/2013 22:01	B	5236083.714	8218645.286	-72.99	-42.955	5940	0.105401748	-1.046868286	0.271266288
87759	4/4/2013 22:19	A	6308455.277	525869.7234	-72.984	-42.995	1080	0.017328538	-1.39361178	0.285324556
87759	4/4/2013 23:24	A	53391577.51	6331990.985	-72.937	-42.974	3900	0.389534497	-1.408353622	0.428091249
87759	4/5/2013 6:11	B	753193604.3	18899508.21	-72.703	-42.889	24420	0	-1.166918999	0
87759	4/5/2013 10:42	A	85582925.56	26314406.44	-72.984	-42.818	16260	-0.290409555	-0.900995338	0.293701071
87759	4/5/2013 11:23	B	135872255.2	19480807.34	-73.003	-42.807	2460	-0.24409801	-0.917102417	0.276048511
87759	4/5/2013 11:54	B	418559959.4	27265418.56	-73.029	-42.864	1860	-0.297335181	-0.65575457	0.181864291

87759	4/5/2013 18:32	B	1715285597	551163.7988	-73.417	-42.77	23880	-0.393870795	-0.308427975	0.095403733
87759	4/5/2013 21:17	B	2178150.633	8231149.867	-73.293	-42.768	9900	-0.867911431	-0.532057487	-0.116585719
87759	4/5/2013 21:50	B	47351579.84	214752578.7	-73.274	-42.776	1980	-0.833049943	-0.524748912	-0.116575031
87759	4/5/2013 23:01	B	61048197.95	76607004.55	-73.22	-42.79	4260	-0.675206064	-0.529982014	-0.107093331
87759	4/6/2013 6:05	B	385323655.9	133917406.6	-72.785	-42.415	25440	0.737687003	-2.041422661	0.627018725
87759	4/6/2013 6:59	B	236552714.5	62830013.48	-72.81	-42.443	3240	0.34656417	-2.762284471	0.535072737
87759	4/6/2013 9:18	B	657711304.3	1001491314	-72.872	-42.349	8340	2.919382127	-3.09328778	1.111624969
87759	4/6/2013 10:19	B	53564671.01	94266610.99	-72.902	-42.371	3660	2.485271216	-3.149455874	0.986533345
87759	4/6/2013 11:01	A	1681142192	42175328.13	-72.981	-42.295	2520	3.261320741	-3.149455874	1.517892048
87759	4/6/2013 11:47	B	48201265.58	4380978.916	-73.009	-42.272	2760	2.292462274	-3.149455874	1.595085119
87759	4/6/2013 18:24	B	4066549110	3296866560	-73.061	-41.993	23820	1.839458181	-3.149455874	0.556235518
87759	4/6/2013 19:57	B	5057547159	38723427.42	-73.003	-42.075	5580	3.256247799	-3.149455874	1.420561901
87759	4/6/2013 21:41	B	22464012.47	4880725.528	-73.043	-42.051	6240	3.112010157	-3.149455874	0.920052968
87759	4/6/2013 22:36	B	4277746.079	1276108.421	-73.07	-42.038	3300	2.066672118	-3.149455874	0.764658762
87759	4/6/2013 23:22	A	3399341.225	52905.27491	-73.064	-42.008	2760	1.131116767	-3.149455874	0.623869147
87759	4/7/2013 8:09	A	211192599.2	28120015.3	-72.953	-42.136	31620	-0.775102349	-3.149455874	1.426607682

87759	4/7/2013 8:58	B	355982311.2	409038047.3	-72.905	-42.159	2940	-1.072468295	-3.149455874	1.438611898
87759	4/7/2013 10:34	B	1039320911	184902689.1	-72.954	-42.133	5760	-0.701451128	-3.149455874	1.423222624
87759	4/7/2013 11:38	B	100415223.1	614824344.9	-72.781	-42.162	3840	-0.776175123	-3.149455874	1.385180531
87759	4/7/2013 19:47	B	1371757399	181763526	-72.924	-42.137	29340	-0.737094919	-3.149455874	1.426400039
87759	4/7/2013 20:26	B	35791743.65	3580642.849	-72.919	-42.127	2340	-0.495052367	-3.149455874	1.41477157
87759	4/7/2013 22:11	B	230653257	12297029.47	-72.862	-42.139	6300	-1.033311484	-3.149455874	0.565631405
87759	4/7/2013 22:49	B	4617760.5	695020.5	-72.868	-42.093	2280	-1.018780441	-3.149455874	0.593606196
87759	4/8/2013 7:18	B	282909.875	753248.625	-72.974	-42.218	30540	-0.704302847	-3.149455874	0.517960968
87759	4/8/2013 7:49	A	8779770.252	280184.2483	-73	-42.197	1860	-0.756526861	-3.149455874	0.547913917
87759	4/8/2013 8:35	B	4936978.824	1350337.676	-72.998	-42.189	2760	-0.823241217	-3.149455874	0.558106729
87759	4/8/2013 8:53	B	18284716.63	6105825.875	-72.983	-42.204	1080	-0.744709321	-3.149455874	0.535536015
87759	4/8/2013 9:32	A	2185913.19	83994.80958	-72.992	-42.24	2340	-0.55585594	-3.149455874	0.492948606
87759	4/8/2013 10:14	B	1805718.165	423642.8353	-72.999	-42.241	2520	-0.518695214	-3.149455874	0.490944397
87759	4/8/2013 11:26	B	2184840.951	659289.0486	-73.003	-42.239	4320	-0.511112227	-3.149455874	0.493235404
87759	4/8/2013 18:01	B	11013749.09	1530643.415	-72.922	-42.359	23700	-0.828164045	-3.149455874	0.384774207
87759	4/8/2013 19:38	B	465976446.4	882754242.6	-72.824	-42.382	5820	-1.063721659	-2.432774294	0.436262225

87759	4/8/2013 21:16	B	21031816.74	12249708.26	-72.876	-42.495	5880	-0.782649775	-3.149455874	0.393689094
87759	4/8/2013 22:58	B	15406382.07	16645499.93	-72.937	-42.497	6120	-0.08132449	-3.149455874	0.313512969
87759	4/8/2013 23:23	A	64497.57902	20125054.92	-72.967	-42.509	1500	0.181142005	-3.149455874	0.271338201
87759	4/9/2013 7:12	B	113457671.6	237390.8883	-72.895	-42.677	28140	-0.019539478	-1.967858544	0.205779921
87759	4/9/2013 7:27	B	19595339.57	341112.9295	-72.897	-42.679	900	0.042877171	-2.036609899	0.200547991
87759	4/9/2013 9:06	B	1634379367	50898257.69	-72.909	-42.815	5940	0.958957493	-1.109487297	-0.342551959
87759	4/9/2013 9:49	B	1046080.752	2592548.248	-72.911	-42.83	2580	0.868375995	-1.156233945	-0.335863794
87759	4/9/2013 11:15	A	26342996.59	16086913.91	-72.841	-42.924	5160	0.036711632	-1.633128075	-0.118439434
87759	4/9/2013 11:31	1	9563337.349	8919105.151	-72.832	-42.929	960	0.003869856	-1.609607015	-0.111225951
87759	4/9/2013 22:58	B	8120908460	2108342585	-73.224	-43.489	41220	-1.02788284	-0.307481533	-0.647355949
87759	4/9/2013 23:38	B	140567304	32588683.98	-73.295	-43.528	2400	-0.401607414	-0.171285498	-0.727586617
87759	4/10/2013 8:35	B	28122454799	10980767691	-73.508	-43.587	32220	-0.648604334	-0.19841685	-0.955179688
87759	4/10/2013 19:16	B	4702142391	445102569.8	-72.789	-43.071	38460	-0.345231147	-1.198333131	-0.169724802
87759	4/11/2013 6:43	B	3851332415	77574901.44	-74.139	-43.607	41220	-0.340941871	-0.149212029	-0.641583976
87759	4/11/2013 8:27	B	1228047241	281319200	-74.14	-43.585	6240	-0.24393093	-0.113677992	-0.650517189
87759	4/11/2013 20:45	B	38459265762	2283106283	-75.063	-43.678	44280	1.181832492	0.078280896	0.053021062

87759	4/11/2013 22:25	B	458841517.9	22369530.06	-75.333	-43.76	6000	-0.013342253	-0.425310527	1.261015305
87759	4/12/2013 8:12	1	7025859.866	264698.6336	-75.863	-44.034	35220	1.16894418	-0.195863523	2.058205423
87759	4/12/2013 9:38	B	1.81989E+11	4565323590	-75.91	-44.08	5160	1.371873684	-0.058862993	1.784672899
87759	4/12/2013 18:54	B	61245356472	5035773468	-76.37	-44.251	33360	-0.575620231	0.492889164	2.595451556
87759	4/12/2013 20:27	B	87021527.98	6763442.516	-76.438	-44.294	5580	-0.537697881	0.559339533	2.692597892
87759	4/13/2013 9:10	B	1073353883	178005585.1	-75.987	-43.963	45780	1.101561374	-0.034290987	1.954460555
87759	4/13/2013 23:01	B	917747240.9	47505551.57	-76.003	-43.505	49860	-1.016760473	0.303926158	2.867699683
87759	4/14/2013 10:24	B	622505517.5	169148145	-76.439	-43.675	40980	-0.381116464	0.503378632	2.667710856
87759	4/14/2013 21:49	B	2499740841	251058459.4	-76.924	-44.293	41100	-0.478250923	0.808452437	2.293898816
87759	4/15/2013 6:01	Z	11117708589	226260283.2	-77.211	-43.846	29520	-1.001873545	0.899199355	2.820807688
87759	4/15/2013 20:00	B	708390.0432	28270.95681	-75.233	-43.548	50340	-0.564336191	0.113947058	1.653869081
87759	4/15/2013 21:43	B	1926861.368	463547.1321	-75.235	-43.552	6180	-0.608321253	0.110342954	2.261199454
87759	4/15/2013 23:55	B	4489894.372	847410.6276	-75.244	-43.561	7920	-0.605380576	0.089630432	2.256733836
87759	4/16/2013 7:59	B	8795936.323	3302934.177	-75.391	-43.617	29040	-0.519129413	-0.105633753	2.284968379
87759	4/16/2013 11:23	B	12692789.12	2943283.878	-75.415	-43.595	12240	-0.554149374	-0.066920159	2.330764838
87759	4/16/2013 19:53	B	10822810.56	6714134.437	-75.764	-43.629	30600	-0.654611152	-0.044402288	2.265988386

87759	4/17/2013 7:16	B	142515126.3	5651098.711	-75.491	-43.359	40980	-0.750494572	0.332466422	2.838819388
87759	4/18/2013 7:16	B	71247780.01	3433944.989	-75.358	-43.534	86400	-0.426918003	0.082510083	2.566341397
87759	4/18/2013 8:47	B	127788453.7	53893328.82	-75.361	-43.53	5460	-0.443645569	0.083570372	2.576573514
87759	4/18/2013 8:50	B	9981011.539	1055281.461	-75.358	-43.53	180	-0.435272174	0.082510083	2.572006569
87759	4/18/2013 10:28	B	7023438.982	1096993.518	-75.37	-43.518	5880	-0.477510277	0.101248116	2.601034609
87759	4/18/2013 19:27	B	27934754.28	2333806.215	-75.409	-43.418	32340	-0.747149245	0.255051593	2.711508861
87759	4/18/2013 22:42	B	14719477.35	2303969.154	-75.409	-43.404	11700	0.02062721	0.271812291	2.729088855
87759	4/19/2013 11:58	B	41005220.28	3535836.218	-75.328	-43.552	47760	0.121682462	0.059812521	2.401120333
87759	4/19/2013 20:58	B	70706720.25	16510792.25	-75.08	-43.791	32400	0.649831815	-0.056137945	1.264146616
87759	4/19/2013 22:11	B	734868.7383	105424147.3	-75.2	-43.856	4380	2.490336846	-0.33516835	1.224445722
87759	4/20/2013 6:42	B	38391062.28	4469260.216	-75.304	-43.883	30660	1.860042669	-0.67159147	1.351790593
87759	4/20/2013 8:21	B	28968150.6	2092149.9	-75.322	-43.905	5940	1.53221278	-0.736750841	1.333823099
87759	4/20/2013 9:33	B	32466547.59	1940664.91	-75.346	-43.919	4320	1.287457799	-0.869616564	1.354285322
87759	4/20/2013 11:15	B	35550886.45	2484206.049	-75.363	-43.955	6120	0.280966357	-0.884912989	1.296980213
87759	4/20/2013 19:08	B	3.46136E+11	24851676566	-75.093	-43.823	28380	1.645738126	-0.131818065	1.207137049
87759	4/20/2013 20:55	B	1918914500	101711496.7	-75.069	-43.846	6420	0.825040551	-0.070633601	1.032132582

87759	4/21/2013 7:30	B	5456196351	108297508.6	-74.875	-43.964	38100	-0.500619833	0.009821186	0.656394888
87759	4/21/2013 9:19	B	23760471434	316529318.9	-74.879	-43.978	6540	-0.323220783	-0.01986699	0.637431751
87759	4/21/2013 10:50	B	341587072.9	19219776.08	-74.841	-43.989	5460	0.006632927	-0.01414187	0.56824527
87759	4/21/2013 21:21	B	118305559.2	25212720.78	-74.834	-43.986	37860	2.326908576	-0.005190092	0.818040274
87759	4/21/2013 23:06	B	239730511.8	11200800.69	-74.528	-44.103	6300	-0.079336409	-0.435782407	-0.209792038
87759	4/22/2013 8:07	B	315181480.5	10869142.01	-74.285	-44.234	32460	-0.962885361	-0.832752252	-0.478219051
87759	4/22/2013 22:41	B	63179189.28	16797367.72	-74.626	-44.158	52440	0.14632132	-0.507110332	-0.137855472
87759	4/23/2013 6:15	B	32306558.38	883366.1165	-74.88	-44.058	27240	2.535838265	-0.198415839	0.532565588
87759	4/23/2013 7:50	B	69502050	346112	-74.911	-44.131	5700	0.879060722	-0.437609075	0.489481127
87759	4/23/2013 10:06	B	38834484.5	1108560.5	-74.816	-44.161	8160	-0.227193832	-0.320700503	0.326459753
87759	4/23/2013 21:57	B	55335200	2796612.5	-74.966	-44.274	42660	0.551555094	-1.082676712	0.266027785
87759	4/23/2013 22:13	B	59637320.7	4050501.8	-74.936	-44.269	960	-0.00788755	-0.896563723	0.191761899
87759	4/24/2013 7:56	B	113827995.3	3288689.174	-75.068	-44.305	34980	-0.39290275	-2.020861479	0.472486164
87759	4/24/2013 9:35	B	136153218.5	23745179.99	-74.797	-44.271	5940	-0.238120157	-0.409978366	0.041335508
87759	4/24/2013 11:18	B	930969996.3	85630124.74	-74.691	-44.219	6180	-0.88311496	-0.454820655	-0.113696167
87759	4/24/2013 20:01	B	43474745.66	2813736.843	-73.938	-43.963	31380	0.690372314	-0.462215668	-0.216595103

87759	4/24/2013 22:00	B	40443719.16	3388693.84	-73.829	-43.928	7140	-0.788203328	-0.852029968	-0.202205144
87759	4/25/2013 9:13	B	630788776.9	55142883.58	-74.338	-44.023	40380	1.584902419	-0.184008405	-0.467499034
87759	4/25/2013 10:54	B	685661357.5	47503194.97	-74.429	-44.043	6060	0.98147812	-0.273743801	-0.412597686
87759	4/25/2013 21:34	B	761857241.8	54487298.73	-75.035	-44.195	38400	-0.451256224	-0.926159628	0.359000931
87759	4/26/2013 8:51	B	46572312720	2106164836	-74.44	-44.081	40620	1.106563797	-0.387259353	-0.428055082
87759	4/26/2013 19:41	B	1396561643	10480051.57	-74.704	-43.925	39000	1.597456778	0.096204375	0.663374389
87759	4/26/2013 21:17	B	2072235979	44958905.7	-74.686	-43.887	5760	-0.007177364	0.155040683	0.406444413
87759	4/27/2013 8:26	B	287009581.6	13192787.42	-73.146	-43.919	40140	-0.348216201	-1.491470643	-0.173762663
87759	4/27/2013 10:06	B	9375904883	110184025.9	-73.068	-43.95	6000	-0.317776269	-1.0856945	-0.109965558
87759	4/27/2013 10:56	B	2286214039	22931088.66	-73.2	-43.983	3000	0.183818498	-1.193863286	-0.08210348
87759	4/27/2013 22:48	B	5256152744	4906118976	-74.442	-43.985	42720	-0.193338315	-0.110456925	0.1939273
87759	4/27/2013 23:54	B	7901233264	3950504749	-74.364	-43.979	3960	-0.217825931	-0.092583161	0.200700281
87759	4/28/2013 7:08	B	2998664664	33128881.24	-74.197	-43.971	29640	0.062314296	-0.075126435	0.176781763
87759	4/28/2013 9:37	B	21781536.18	577556.3248	-74.214	-43.972	8940	0.020236258	-0.06238765	0.173042507
87759	4/28/2013 19:24	B	4644537290	35558654.3	-74.154	-43.946	35220	0.466356097	-0.159205262	0.259237557
87759	4/29/2013 7:35	B	21157572.19	7958.313279	-73.368	-44.05	43860	-1.221874737	-2.198256816	0.026317147

87759	4/29/2013 8:29	B	2131527.036	210537.4641	-73.366	-44.051	3240	-1.217413508	-2.198256816	0.025543222
87759	4/29/2013 10:08	B	6016202.199	509056.3012	-73.425	-43.879	5940	-0.83858 99	-1.809518387	-0.025203999
87759	4/29/2013 11:49	B	8672949.453	472763.0465	-73.444	-43.808	6060	-0.632899384	-0.966203432	-0.107533146
87759	4/29/2013 19:12	B	16168985.14	3274631.859	-73.414	-43.612	26580	-0.904868239	-0.292789739	-0.373631779
87759	4/29/2013 21:23	B	42675305.99	4800368.006	-73.426	-43.609	7860	0.222144089	-0.275294562	-0.78472317
87759	4/29/2013 23:01	B	1095495193	499603349.9	-73.724	-43.749	5880	-0.395865604	-1.263760025	-0.84281676
87759	4/30/2013 6:45	B	6608646666	2195266.893	-74.074	-43.755	27840	0.13063529	-0.497837676	0.032922181
87759	4/30/2013 8:08	B	14427939.01	310465.994	-73.978	-43.758	4980	0.369130064	-0.816062821	-0.049754721
87759	4/30/2013 8:54	B	26230385.02	2027243.975	-73.978	-43.702	2760	0.569186507	-0.66868783	-0.000126414
87759	4/30/2013 9:54	B	60240170.41	2456999.595	-73.909	-43.706	3600	1.121757817	-0.937424623	-0.280551339
87759	4/30/2013 10:37	B	60180013.75	2952388.748	-73.883	-43.71	2580	1.096708069	-1.037393221	-0.396154791
87759	4/30/2013 11:17	B	3349720153	4087367.639	-73.829	-43.737	2400	0.614302842	-1.355815706	-0.604708009
87759	4/30/2013 11:36	B	44735419.77	384433.2281	-73.84	-43.736	1140	0.71420334	-1.343623057	-0.566367457
87759	4/30/2013 19:01	A	8353304675	259721276.9	-73.8	-43.698	26700	0.39677342	-0.988786897	-0.730201833
87759	4/30/2013 22:12	B	226597102.6	14110235.38	-73.802	-43.732	11460	1.389975447	-1.341457605	-1.031105245
87759	4/30/2013 22:33	B	516125378.3	19502584.17	-73.798	-43.728	1260	1.415541881	-1.303485632	-1.047134157

87759	4/30/2013 22:47	A	8542.158293	131493.8417	-73.804	-43.733	840	1.390732443	-1.344012044	-1.028308044
87759	5/1/2013 6:33	A	334535.0353	8166001.465	-73.882	-43.693	27960	-0.585184836	-0.875638611	-1.187802214
87759	5/1/2013 6:48	B	3755988.5	6186568.5	-73.881	-43.695	900	-0.533513019	-0.914127639	-1.18477912
87759	5/1/2013 8:04	B	184321456.2	90996.31324	-73.877	-43.711	4560	-0.220442793	-1.053304673	-1.162041826
87759	5/1/2013 8:31	B	20950866.39	558442.6102	-73.877	-43.711	1620	-0.220442793	-1.053304673	-1.162041826
87759	5/1/2013 10:07	B	195161434.2	82306550.84	-73.872	-43.721	5760	0.032226818	-1.178295819	-1.139863785
87759	5/1/2013 10:50	B	5081672	678612.5	-73.866	-43.729	2580	0.248410501	-1.251887183	-1.118668771
87759	5/1/2013 18:46	B	21193620.31	1244373.692	-73.826	-43.766	28560	0.559322667	-2.098990018	-0.931535628
87759	5/1/2013 20:22	B	290095143.7	113272581.3	-73.838	-43.75	5760	1.481063605	-1.66590117	-1.029895802
87759	5/1/2013 22:26	B	4954698.958	1578757.542	-73.829	-43.758	7440	1.479492468	-1.868847771	-0.958333576
87759	5/1/2013 23:43	A	385705.4161	63937019.58	-73.841	-43.658	4620	-0.7897574	-0.716306696	-1.279769885
87759	5/1/2013 23:58	B	3691925.481	10439601.02	-73.84	-43.652	900	-0.768972118	-0.677915994	-1.276512311
87759	5/2/2013 6:08	B	81288485.74	7427975.261	-73.843	-43.571	22200	-0.130317691	-0.348878371	-1.104534124
87759	5/2/2013 7:51	1	1148536.97	159284.0301	-73.906	-43.566	6180	0.222038834	-0.302624193	-1.036134347
87759	5/2/2013 8:04	A	34341858.05	4078074.954	-73.908	-43.544	780	-0.324412247	-0.242145644	-0.994703018
87759	5/2/2013 9:39	B	60830251.57	25739520.93	-73.916	-43.529	5700	-0.235842908	-0.194693404	-0.973845319

87759	5/2/2013 10:13	A	1391068.038	21890570.46	-73.834	-43.521	2040	0.187308183	-0.200863	-1.162392964
87759	5/2/2013 20:08	B	11445704.8	2208069.704	-73.707	-43.429	35700	-0.391600535	0.026544515	-1.223351578
87759	5/2/2013 21:59	B	212320177.6	76977379.41	-73.741	-43.572	6660	-0.604388277	-0.340963304	-1.250449758
87759	5/2/2013 23:09	B	4946196112	13587904.99	-73.79	-43.632	4200	-0.850814659	-0.59762918	-1.186738384
87759	5/2/2013 23:40	B	7673516.386	1134981.614	-73.741	-43.646	1860	-0.489139669	-0.6279936	-1.237862713
87759	5/3/2013 6:10	B	104379868.2	479656151.8	-73.8	-43.727	23400	0.499186767	-1.292934046	-1.034048134
87759	5/3/2013 7:42	A	142019142.5	190107.5398	-73.807	-43.704	5520	0.053204131	-1.07620501	-1.100585366
87759	5/3/2013 9:20	A	567405.4145	133348.5855	-73.811	-43.724	5880	0.337401586	-1.280886879	-1.042258657
87759	5/3/2013 11:23	B	63538512.76	3461968.244	-73.859	-43.7	7380	-0.40947567	-0.992226153	-1.100037475
87759	5/3/2013 11:24	B	140320219.5	6550831.048	-73.859	-43.7	60	-0.40947567	-0.992226153	-1.100037475
87759	5/3/2013 20:02	B	127815408.4	17799876.55	-73.904	-43.692	31080	0.132129334	-0.820889041	-0.602949506
87759	5/3/2013 21:05	B	14246403.19	537957.8132	-73.825	-43.623	3780	-0.195688702	-0.559768433	-0.667623222
87759	5/3/2013 21:35	B	703725450.5	31906820.01	-73.773	-43.593	1800	-0.260253669	-0.437525586	-0.694157816
87759	5/3/2013 22:41	B	9381201099	990404313.9	-73.763	-43.634	3960	-0.588484731	-0.592872095	-0.7567085
87759	5/3/2013 23:12	A	41618.16939	34881.83061	-73.768	-43.651	1860	-0.660518007	-0.663840218	-0.767482626
87759	5/4/2013 7:29	1	443564107	36472573.5	-73.828	-43.734	29820	-0.678155428	-1.344980999	-0.767568621

87759	5/4/2013 7:39	0	1878375.785	4019572.715	-73.846	-43.741	600	-0.569014099	-1.444341527	-0.751276731
87759	5/4/2013 8:50	B	14319264.6	1915249.898	-73.817	-43.742	4260	-0.756699537	-1.550347414	-0.783073734
87759	5/4/2013 9:09	B	1103282.469	209674517.5	-73.827	-43.744	1140	-0.701501848	-1.544639369	-0.775177701
87759	5/4/2013 10:54	A	102049993.6	18537786.4	-73.83	-43.687	6300	-0.537521939	-0.878614961	-0.732090458
87759	5/4/2013 18:20	B	423065328.5	8680945.524	-73.787	-43.549	26760	0.175293147	-0.274489113	-0.591623913
87759	5/4/2013 19:44	B	383631719.3	24552993.17	-73.823	-43.539	5040	0.491910746	-0.242537966	-0.498972901
87759	5/4/2013 20:03	B	1876595894	338129128.4	-73.814	-43.55	1140	0.951097074	-0.283467576	-0.545642873
87759	5/4/2013 20:33	B	8840088.411	1404961.589	-73.755	-43.52	1800	1.987628773	-0.195492173	-0.420395124
87759	5/4/2013 21:08	B	3208085.73	252638.2705	-73.731	-43.506	2100	2.058752072	-0.158424463	-0.404160707
87759	5/4/2013 22:09	B	27751113.69	18199484.81	-73.698	-43.485	3660	2.068122593	-0.083989186	-0.426960668
87759	5/4/2013 22:46	B	321199395.7	83389984.32	-73.68	-43.503	2220	2.147203506	-0.125599065	-0.590000977
87759	5/4/2013 23:51	B	5143780.007	1040003.993	-73.64	-43.496	3900	1.516797434	-0.092034552	-0.711822945
87759	5/5/2013 7:20	2	614241.1208	30883.87919	-73.657	-43.615	26940	-0.536113891	-0.420335807	-0.814940538
87759	5/5/2013 8:28	B	83370438	27803214	-73.593	-43.646	4080	-0.690712445	-0.443343283	-0.794396592
87759	5/5/2013 8:59	B	10547853.58	3611958.921	-73.578	-43.652	1860	-0.70129278	-0.435365531	-0.79249882
87759	5/5/2013 10:11	B	13458358.76	1594383.744	-73.536	-43.658	4320	-0.950257694	-0.42477587	-0.794000644

87759	5/5/2013 10:17	B	10058697.11	2041811.386	-73.537	-43.664	360	-0.952228834	-0.442348328	-0.800136836
87759	5/5/2013 18:10	B	73344484.33	3340090.171	-73.45	-43.595	28380	-0.045829469	-0.226125943	-0.921693932
87759	5/5/2013 19:50	B	47953965.78	55122160.72	-73.624	-43.546	6000	-0.675494781	-0.183891086	-0.845412608
87759	5/5/2013 20:46	B	6357356.681	5048263.319	-73.63	-43.54	3360	-0.278629194	-0.181973705	-0.850866782
87759	5/5/2013 21:13	B	978158060.5	299592.0272	-73.624	-43.552	1620	-0.467157587	-0.198379409	-0.856159768
87759	5/5/2013 21:36	B	6741997.863	1219770.137	-73.627	-43.545	1380	-0.374772032	-0.183567969	-0.853077479
87759	5/5/2013 22:22	B	3993138	445568	-73.63	-43.542	2760	-0.340364305	-0.180700658	-0.853067097
87759	5/5/2013 23:18	2	467048.1716	132720.3284	-73.613	-43.61	3360	-0.440759629	-0.351899159	-0.808357795
87759	5/6/2013 7:05	A	185520.25	185520.25	-73.623	-43.725	28020	-0.857837903	-0.847941164	-0.808370057
87759	5/6/2013 7:20	B	290081.0771	85875.42294	-73.623	-43.724	900	-0.850134103	-0.847941164	-0.809938084
87759	5/6/2013 8:05	3	9843.379097	19541.1209	-73.644	-43.728	2700	-0.775707354	-0.904433779	-0.798611011
87759	5/6/2013 8:45	A	21657934.4	2037648.096	-73.672	-43.751	2400	-0.827580036	-1.109977394	-0.777736247
87759	5/6/2013 9:47	B	928386.2238	889170.7762	-73.628	-43.755	3720	-0.662243935	-1.090030024	-0.788833359
87759	5/6/2013 11:33	B	10887253.72	4364210.278	-73.63	-43.777	6360	0.007845668	-1.325141518	-0.75335773
87759	5/6/2013 18:00	B	41174284.94	5628756.056	-73.628	-43.766	23220	-0.407934681	-1.168545035	-0.775613763
87759	5/6/2013 19:21	B	29204307.2	15224153.3	-73.619	-43.808	4860	0.607778257	-1.633542867	-0.664335138

87759	5/6/2013 19:33	B	11450165.04	7216053.463	-73.624	-43.785	720	0.278372683	-1.39973339	-0.730144149
87759	5/6/2013 20:22	B	9303171.5	3594753.5	-73.611	-43.775	2940	-0.415030091	-1.190488148	-0.716473799
87759	5/6/2013 21:08	B	1036852159	205631563.9	-73.546	-43.767	2760	-0.657065364	-0.870390259	-0.721468329
87759	5/6/2013 21:21	B	48459313.88	7073722.622	-73.546	-43.767	780	-0.657065364	-0.870390259	-0.721468329
87759	5/6/2013 22:01	B	79102455.37	8490314.627	-73.554	-43.775	2400	-0.611783007	-0.942509856	-0.71235936
87759	5/6/2013 22:44	B	46020013.72	924678.7791	-73.557	-43.754	2580	-0.714703352	-0.84502668	-0.737263718
87759	5/6/2013 23:42	B	5167805.586	234506.9135	-73.555	-43.75	3480	-0.756485705	-0.825027877	-0.742517431
87759	5/7/2013 8:39	B	21890799.06	3885833.438	-73.569	-43.702	32220	-1.111673938	-0.651633187	-0.779125542
87759	5/7/2013 10:58	B	60772798.14	11215606.36	-73.591	-43.687	8340	-1.113901859	-0.583035213	-0.787519212
87759	5/7/2013 19:29	B	9549338.168	15612744.33	-73.53	-43.719	30660	-0.985858359	-0.648454605	-0.763541882
87759	5/7/2013 20:53	B	1966640.778	1665868.222	-73.528	-43.714	5040	-0.933438824	-0.62310747	-0.781412012
87759	5/7/2013 23:13	B	629531799	12435665.03	-73.533	-43.726	8400	-0.94395898	-0.68138643	-0.774960605
87759	5/8/2013 6:46	3	220514.5649	3410.435121	-73.477	-43.742	27180	-0.893992254	-0.630681198	-0.749469341
87759	5/8/2013 7:00	1	6373442.511	250241.9887	-73.466	-43.763	840	-0.923289269	-0.701590921	-0.729566351
87759	5/8/2013 8:26	A	161128.8321	633733.6679	-73.57	-43.723	5160	-0.995405884	-0.746546002	-0.780546288
87759	5/8/2013 9:01	A	505294.4934	102017.5066	-73.55	-43.737	2100	-0.964188106	-0.763280057	-0.768368711

87759	5/8/2013 10:39	A	219721.3546	205535.1454	-73.568	-43.76	5880	-1.002306145	-0.908130457	-0.756219603
87759	5/8/2013 19:12	B	69143423.74	20172776.26	-73.583	-43.748	30780	-1.018538313	-0.902997274	-0.767470303
87759	5/8/2013 20:43	A	22387601493	845888878.5	-73.587	-43.703	5460	-0.320041917	-0.689896555	-0.512284753
87759	5/8/2013 22:50	B	378255436.5	27159360.49	-73.679	-43.756	7620	-0.716913	-1.175341703	-0.485688019
87759	5/9/2013 6:32	B	2837332.148	15013331.85	-73.468	-43.823	27720	-1.035672545	-1.011701451	-0.439618664
87759	5/9/2013 8:07	B	1923595.748	1653981.252	-73.471	-43.838	5700	-0.989165926	-1.145205433	-0.446870062
87759	5/9/2013 20:48	A	49866754.44	30465407.56	-73.251	-43.752	45660	-0.924822376	-1.111871526	-0.435775015
87759	5/9/2013 22:11	B	289612177.3	218850175.2	-73.278	-43.772	4980	-0.966394885	-1.374917747	-0.425792148
87759	5/9/2013 22:27	A	107525.2239	6967.27613	-73.247	-43.804	960	-0.801169684	-2.129019868	-0.46463919
87759	5/10/2013 7:58	B	8651600.047	2777785.953	-73.253	-43.807	34260	-0.789616919	-2.162596485	-0.465406432
87759	5/10/2013 9:48	B	70750790.59	3317261.911	-73.185	-43.781	6600	-0.781880062	-1.631374214	-0.501944519
87759	5/10/2013 18:58	A	5379701361	286741056.5	-73.323	-43.744	33000	-1.173723823	-0.989813682	-0.407730032
87759	5/10/2013 20:19	A	7324723327	20129273.57	-73.396	-43.754	4860	-1.065325505	-0.862382799	-0.408959059
87759	5/10/2013 20:32	A	12582767.57	945984.932	-73.382	-43.756	780	-1.106324637	-0.907911752	-0.407707502
87759	5/10/2013 22:03	B	4484798.606	169715.8941	-73.401	-43.755	5460	-1.048565386	-0.836809052	-0.408560576
87759	5/10/2013 23:40	B	145502966	2199568.546	-73.422	-43.715	5820	-0.3649112	-0.620371634	-0.457412869

87759	5/11/2013 6:24	A	28461.22882	8998.771181	-73.414	-43.706	24240	-0.278915611	-0.611215012	-0.474057968
87759	5/11/2013 7:44	A	17594.38688	260345.6131	-73.396	-43.738	4800	-0.84354685	-0.784304618	-0.420079685
87759	5/11/2013 7:55	B	3571377.899	6086.601492	-73.395	-43.738	660	-0.84354685	-0.784304618	-0.420079685
87759	5/11/2013 9:24	1	9135228.453	14416.54655	-73.377	-43.748	5340	-1.058607577	-0.87963132	-0.40966996
87759	5/11/2013 11:29	B	24098806.65	7580455.847	-73.378	-43.76	7500	-1.143173226	-0.940190405	-0.406297468
87759	5/11/2013 20:06	B	85389070.61	31035987.89	-73.377	-43.803	31020	-0.858043859	-1.229348059	-1.011646531
87759	5/11/2013 21:44	B	44125154.28	79406730.72	-73.392	-43.787	5880	-0.83527737	-1.062863694	-1.003248316
87759	5/11/2013 23:18	B	160410548.3	38866808.19	-73.412	-43.749	5640	-0.790845941	-0.771298507	-0.973277622
87759	5/12/2013 6:10	B	21198530.08	1474186.918	-73.382	-43.707	24720	-0.968814263	-0.685182841	-0.926691989
87759	5/12/2013 20:00	0	6258146702	194972731.8	-73.349	-43.292	49800	0.270776128	-0.66681827	-1.078412228
87759	5/12/2013 20:12	B	239831134	11353718.5	-73.346	-43.292	720	0.262164003	-0.66681827	-1.082340694
87759	5/12/2013 21:16	B	271837387	22438603.53	-73.353	-43.29	3840	0.311027455	-0.662273661	-1.056943609
87759	5/12/2013 21:45	B	2760703.734	343253.2656	-73.348	-43.288	1740	0.252557422	-0.666702857	-1.074374821
87759	5/12/2013 22:54	B	3669673.835	317038.6652	-73.357	-43.274	4140	0.207964616	-0.668219463	-1.047880115
87759	5/13/2013 8:36	B	21268908.74	1015424.259	-73.294	-43.003	34920	-0.138789184	-0.264763383	-1.383247502
87759	5/13/2013 9:08	B	96177820.77	14598279.73	-73.28	-42.991	1920	-0.198831308	-0.279219288	-1.379510661

87759	5/13/2013 10:10	B	47638561.92	5145286.084	-73.254	-42.984	3720	-0.379596138	-0.320887823	-1.339192244
87759	5/13/2013 19:48	B	99263668.56	9959589.943	-73.287	-42.846	34680	-0.09153972	-0.280099487	-1.290356128
87759	5/13/2013 21:34	B	3526892300	4696471.618	-73.081	-42.993	6360	-0.545059379	-0.890281153	-1.166182389
87759	5/13/2013 22:33	A	722753606.2	923078.7899	-73.035	-42.992	3540	-0.526137287	-1.084272255	-1.115436972
87759	5/14/2013 7:20	B	58770793.71	15082428.79	-73.004	-42.934	31620	-0.428143946	-0.853988213	-1.010815899
87759	5/14/2013 7:35	B	13709797.3	7663393.202	-73.02	-42.927	900	-0.406610827	-0.773901115	-1.015282675
87759	5/14/2013 8:56	B	7491788.397	7166556.603	-73.009	-42.92	4860	-0.412142075	-0.799129882	-0.994229844
87759	5/14/2013 19:42	B	600741352.4	11966256.6	-73.052	-42.722	38760	-0.284359232	-1.196405339	-0.790385965
87759	5/14/2013 21:55	1	329613.0987	130791.4013	-73.136	-42.794	7980	-0.4485792	-0.615627144	-0.83412036
87759	5/14/2013 22:01	A	4388354.766	25310.23446	-73.148	-42.801	360	-0.329042582	-0.567883535	-0.857568623
87759	5/15/2013 7:12	B	74334624.5	23632812.5	-73.588	-43.035	33060	0.282263951	0.050630949	-0.743654431
87759	5/15/2013 7:52	B	32688085.28	13392318.72	-73.515	-43.024	2400	0.179640759	-0.023005785	-0.957783923
87759	5/15/2013 11:45	0	27043606.39	2122353.611	-72.918	-43.003	13980	-1.17619233	-2.055978277	-0.903126367
87759	5/15/2013 18:04	B	1266978881	254633052.4	-72.575	-43.033	22740	0	-0.421982872	0
87759	5/15/2013 19:44	B	1045089757	126732517.4	-72.517	-43.099	6000	0	-0.234903423	0
87759	5/15/2013 21:36	B	5502743.534	342649.4661	-72.975	-43.059	6720	0.147941717	-1.816987629	-0.908786217

87759	5/16/2013 8:31	B	9058556.506	3402137.994	-72.935	-43.041	39300	-0.465123712	-2.114511719	-0.857146557
87625	4/23/2013 20:07	B	1445289616	162773088	-72.716	-41.69	5370	0.545402868	-3.149455874	1.682822194
87625	4/23/2013 22:22	B	1445289616	162773088	-72.714	-41.707	8083	1.648269798	-2.480074331	1.739300603
87625	4/24/2013 7:40	B	1445289616	162773088	-72.693	-41.685	33462	2.053678252	-2.043784294	1.930335994
87625	4/24/2013 11:20	B	1445289616	162773088	-72.697	-41.68	13241	2.109635699	-2.010910187	1.936363313
87625	4/25/2013 6:00	B	1445289616	162773088	-72.557	-41.87	67184	0	-0.574120328	0
87625	4/25/2013 7:27	B	1445289616	162773088	-72.694	-41.798	5200	0	-1.593532125	1.542601631
87625	4/25/2013 7:34	B	1445289616	162773088	-72.68	-41.798	457	0	-1.499887789	1.547459493
87625	4/25/2013 9:11	B	1445289616	162773088	-72.591	-41.788	5813	0	-1.067567213	0
87625	4/25/2013 10:51	B	1445289616	162773088	-72.571	-41.784	5968	0	-1.032995663	0
87625	4/25/2013 11:17	B	1445289616	162773088	-72.565	-41.777	1562	0	-1.061979715	0
87625	4/25/2013 11:36	B	1445289616	162773088	-72.565	-41.777	1123	0	-1.049049068	0
87625	4/25/2013 18:14	B	1445289616	162773088	-72.49	-41.741	23925	0	-0.842723989	1.809735526
87625	4/25/2013 19:50	A	1846137731	80281855.35	-72.699	-41.667	5717	1.445216257	-1.753885738	1.828295175
87625	4/25/2013 20:01	B	1445289616	162773088	-72.668	-41.677	694	1.297135056	-1.784829357	1.799722336
87625	4/25/2013 21:32	A	1846137731	80281855.35	-72.771	-41.711	5450	1.443695706	-3.126025483	1.614455693

87625	4/25/2013 23:12	B	1445289616	162773088	-72.706	-41.699	5973	1.259762322	-2.381919707	1.723759688
87625	4/26/2013 7:14	B	1445289616	162773088	-72.874	-41.645	28953	-0.794860922	-3.149455874	0.109264471
87625	4/26/2013 8:59	B	1445289616	162773088	-72.798	-41.732	6313	-0.718164336	-3.149455874	0.18199435
87625	4/26/2013 11:10	B	1445289616	162773088	-72.714	-41.744	7815	-0.819812876	-2.881946402	0.20497336
87625	4/26/2013 19:35	B	1445289616	162773088	-72.832	-41.714	30327	-0.694703531	-3.149455874	0.161430005
87625	4/26/2013 19:48	B	1445289616	162773088	-72.803	-41.72	807	-0.705016138	-3.149455874	0.171220982
87625	4/26/2013 21:07	B	1445289616	162773088	-72.814	-41.725	4694	-0.698784013	-3.149455874	0.171946967
87625	4/26/2013 21:19	B	1445289616	162773088	-72.82	-41.724	713	-0.697529341	-3.149455874	0.170516998
87625	4/26/2013 21:26	B	1445289616	162773088	-72.815	-41.727	474	-0.69850386	-3.149455874	0.171757597
87625	4/26/2013 22:45	A	1846137731	80281855.35	-72.789	-41.668	4702	-0.721417122	-2.978932634	0.144473171
87625	4/27/2013 8:39	1	19838678.47	1565421.652	-72.802	-41.647	35672	-0.838139299	-2.874241837	0.086919479
87625	4/27/2013 10:05	2	863491.9002	117318.8964	-72.76	-41.659	5140	-0.89431115	-2.524315342	0.101403106
87625	4/27/2013 19:40	0	4697718710	205165923.2	-72.977	-41.65	34517	-1.051546793	-2.546618487	0.099738182
87625	4/27/2013 20:38	A	1846137731	80281855.35	-72.975	-41.664	3464	-1.128861739	-2.331053609	0.100358602
87625	4/27/2013 21:08	0	4697718710	205165923.2	-73.027	-41.707	1812	-1.079376728	-1.287881487	0.096316106
87625	4/27/2013 22:19	1	19838678.47	1565421.652	-73.044	-41.796	4245	-0.443657752	-1.232952506	0.102553206

87625	4/27/2013 22:45	A	1846137731	80281855.35	-73.039	-41.791	1549	-0.535601111	-1.255991053	0.100383088
87625	4/27/2013 23:57	B	1445289616	162773088	-73.003	-41.854	4353	-0.015311169	-1.650970274	0.186410534
87625	4/28/2013 7:05	2	863491.9002	117318.8964	-73.244	-42.087	25642	0.646694032	-3.149455874	0.538590184
87625	4/28/2013 8:02	2	863491.9002	117318.8964	-73.226	-42.127	3462	0.970933033	-3.149455874	0.528874682
87625	4/28/2013 8:41	B	1445289616	162773088	-73.221	-42.146	2310	1.26888417	-3.149455874	0.49819777
87625	4/28/2013 9:45	A	1846137731	80281855.35	-73.225	-42.198	3857	2.021989682	-3.149455874	0.327139935
87625	4/28/2013 10:41	B	1445289616	162773088	-73.232	-42.226	3343	2.200590922	-3.149455874	0.218140978
87625	4/28/2013 19:26	B	1445289616	162773088	-73.324	-42.431	31475	0.014911857	-2.057506924	-0.351913873
87625	4/28/2013 20:59	A	1846137731	80281855.35	-73.171	-42.431	5621	-0.365997915	-1.974215815	-0.359001466
87625	4/28/2013 21:08	B	1445289616	162773088	-73.17	-42.431	517	-0.365997915	-2.00160116	-0.359001466
87625	4/28/2013 21:53	A	1846137731	80281855.35	-73.15	-42.462	2682	0.114731056	-2.633420343	-0.311564186
87625	4/28/2013 22:35	B	1445289616	162773088	-73.085	-42.48	2576	0.580534228	-3.125530432	-0.181429198
87625	4/28/2013 23:38	B	1445289616	162773088	-73.059	-42.502	3743	0.453181446	-3.008673962	-0.125291869
87625	4/29/2013 8:22	B	1445289616	162773088	-72.566	-42.525	31461	0	-0.731105042	-0.335876818
87625	4/29/2013 11:51	B	1445289616	162773088	-72.759	-42.401	12522	-0.614641156	-1.566899362	-0.174237587
87625	4/29/2013 19:03	B	1445289616	162773088	-72.966	-42.496	25949	-0.528692994	-3.149455874	-0.156249045

87625	4/29/2013 19:12	B	1445289616	162773088	-72.972	-42.497	517	-0.530480932	-3.149455874	-0.155872479
87625	4/29/2013 20:40	B	1445289616	162773088	-72.929	-42.526	5308	-0.292594964	-3.149455874	-0.198283962
87625	4/29/2013 23:16	B	1445289616	162773088	-72.874	-42.547	9354	-0.233972222	-3.024068546	-0.245474508
87625	4/30/2013 6:33	B	1445289616	162773088	-73	-42.528	26182	-0.627158112	-3.149455874	-1.126629233
87625	4/30/2013 6:47	B	1445289616	162773088	-73.045	-42.542	843	-0.753343946	-2.803586436	-1.149552768
87625	4/30/2013 8:18	B	1445289616	162773088	-73.023	-42.554	5462	-0.775836774	-2.971127427	-1.147854004
87625	4/30/2013 10:36	B	1445289616	162773088	-73.062	-42.689	8321	-0.883241834	-1.342281509	-1.195107688
87625	4/30/2013 11:15	B	1445289616	162773088	-72.994	-42.66	2311	-0.943502184	-2.263152825	-1.164697404
87625	4/30/2013 11:53	B	1445289616	162773088	-72.982	-42.706	2299	-0.976116431	-2.069763833	-1.163390197
87625	4/30/2013 19:08	0	4697718710	205165923.2	-73.451	-42.951	26077	-0.915858449	0.003537834	-1.293698087
87625	4/30/2013 20:44	1	19838678.47	1565421.652	-73.329	-42.889	5744	-0.528597942	-0.155153611	-1.255977208
87625	4/30/2013 20:57	B	1445289616	162773088	-73.328	-42.889	818	-0.523862632	-0.161023853	-1.254094251
87625	4/30/2013 21:06	B	1445289616	162773088	-73.328	-42.888	540	-0.526510887	-0.158598163	-1.255718737
87625	4/30/2013 22:11	B	1445289616	162773088	-73.316	-42.907	3857	-0.503172637	-0.149812086	-1.239410041
87625	4/30/2013 22:31	0	4697718710	205165923.2	-73.294	-42.898	1247	-0.469957936	-0.190226255	-1.224932627
87625	4/30/2013 22:50	B	1445289616	162773088	-73.287	-42.896	1107	-0.45803232	-0.205168134	-1.217470449

87625	5/1/2013 6:26	B	1445289616	162773088	-73.246	-42.888	27396	1.306997584	-0.255148117	-1.246160456
87625	5/1/2013 8:28	B	1445289616	162773088	-73.078	-43.079	7297	1.26699908	-0.982420079	-1.364517563
87625	5/1/2013 10:07	B	1445289616	162773088	-73.036	-43.121	5965	1.703925012	-1.234519996	-1.425819638
87625	5/1/2013 22:04	B	1445289616	162773088	-72.892	-43.627	42987	0	-0.304691616	-0.948228594
84484	4/17/2015 7:36	B	57247422.63	12507250.37	-73.138	-41.941	4460	-0.617456172	-1.548300952	2.136389211
84484	4/17/2015 9:18	A	2063887.163	53195.33682	-73.154	-41.925	6102	-0.604452162	-1.802789988	2.189734728
84484	4/17/2015 10:05	A	252831.7357	116816.7643	-73.176	-41.935	2794	-0.607289957	-1.883422017	2.204834215
84484	4/17/2015 10:41	2	135948.6441	77469.35593	-73.135	-41.925	2183	-0.606658135	-1.590098	2.165669615
84484	4/17/2015 10:58	B	4051826.604	1171327.896	-73.132	-41.925	999	-0.607606112	-1.567458157	2.160818233
84484	4/17/2015 12:24	B	1649820423	218571689.9	-73.092	-41.949	5171	-0.634428208	-1.142659033	2.072726046
84484	4/17/2015 13:38	B	3858451.84	540716.6604	-73.071	-41.952	4480	-0.647572038	-0.975761185	2.0369298
84484	4/17/2015 14:18	B	215296548.2	1652509.831	-73.091	-41.994	2377	-0.662610589	-0.860801819	2.002361912
84484	4/17/2015 18:49	B	852000216.8	101045377.7	-73.164	-42	16263	-0.634548923	-1.011693332	2.089183302
84484	4/17/2015 20:27	B	5559699.197	8449795.303	-73.158	-42.012	5880	-0.6408303	-0.937714999	2.066620099
84484	4/17/2015 21:40	B	87259203.33	674129.1671	-73.13	-41.968	4368	-0.629197717	-1.273223696	2.093271349
84484	4/18/2015 0:01	B	5397155.764	522958.2361	-73.123	-41.98	8450	-0.599461083	-1.121300415	0.519146133

84484	4/18/2015 0:55	B	14015114.16	1457824.338	-73.12	-41.98	3235	-0.600034171	-1.102545752	0.518026439
84484	4/18/2015 2:39	B	414031083.3	25742159.18	-73.105	-41.997	6244	-0.634087142	-0.870885728	0.466245053
84484	4/18/2015 4:19	B	13162216.58	557403.9197	-73.099	-41.992	6025	-0.628271743	-0.883091598	0.474422966
84484	4/18/2015 7:25	A	230990.0581	4064885.942	-73.111	-41.99	11182	-0.618528653	-0.959678598	0.490437772
84484	4/18/2015 7:50	B	349284.3398	784980.1602	-73.111	-41.99	1494	-0.617760323	-0.96894273	0.491516018
84484	4/18/2015 7:59	B	548612.8193	2526867.681	-73.118	-41.994	498	-0.622449739	-0.917760816	0.485956408
84484	4/18/2015 9:04	B	77407860.82	7237444.182	-73.118	-41.997	3944	-0.626243071	-0.909833405	0.479869682
84484	4/18/2015 9:37	B	25377385.93	648623.0697	-73.116	-41.989	1940	-0.617760323	-0.978636458	0.491516018
84484	4/18/2015 10:13	B	6251396.238	198176.762	-73.101	-41.994	2173	-0.62979168	-0.877933869	0.471894509
84484	4/18/2015 11:18	2	38022.32989	81524.17011	-73.043	-41.904	3925	-0.530812347	-0.958421628	0.59619419
84484	4/18/2015 11:50	A	1861750.945	916845.0548	-73.048	-41.908	1875	-0.532822659	-0.965687145	0.592448627
84484	4/18/2015 12:16	B	2659733.158	752443.8417	-73.042	-41.901	1606	-0.523846408	-0.971425426	0.605572819
84484	4/18/2015 13:17	B	3889493.162	1444973.338	-73.033	-41.887	3622	-0.509941667	-1.013780322	0.624355733
84484	4/18/2015 13:57	B	4256312.061	811314.4393	-73.032	-41.887	2435	-0.508460964	-1.011600181	0.626374603
84484	4/19/2015 8:55	A	3964039.572	285022.9278	-73.176	-41.857	68236	-0.012696727	-1.111886336	1.31700523
84484	4/19/2015 9:19	B	608415.5114	123514.4886	-73.176	-41.857	1447	-0.012696727	-1.116125877	1.31700523

84484	4/19/2015 10:33	A	83986.85682	78639.64318	-73.157	-41.866	4435	-0.05058316	-1.273672029	1.28916123
84484	4/19/2015 10:52	B	4128726.481	153563.519	-73.159	-41.845	1158	-0.099288783	-1.009760277	1.359889298
84484	4/19/2015 11:17	B	823584.4629	214601.5371	-73.152	-41.861	1478	-0.083474515	-1.212984689	1.302450578
84484	4/19/2015 12:02	B	18228427.4	4660561.597	-73.147	-41.858	2737	-0.100183289	-1.092678966	1.307792708
84484	4/19/2015 20:02	B	479154737	977283381.5	-73.037	-41.834	28771	-0.608331269	-1.373953861	1.383644001
84484	4/19/2015 20:58	A	11883369.13	240755.873	-73.108	-41.867	3379	-0.230407275	-1.095798595	1.285106431
84484	4/19/2015 21:26	B	7849416.003	13382636.5	-73.068	-41.892	1689	-0.37142034	-1.027493148	1.247286137
84484	4/19/2015 21:44	B	16796348.54	33473701.46	-73.089	-41.903	1087	-0.236086883	-1.160357531	1.198851196
84484	4/19/2015 23:07	3	245934.3706	3025.629437	-73.137	-41.878	4938	-0.085955894	-1.383389872	1.245360159
84484	4/21/2015 8:25	A	5421501	5421501	-73.128	-41.873	119911	2.245113644	-1.240649677	0.777346143
84484	4/21/2015 8:30	B	2187796.305	3317048.695	-73.127	-41.87	278	2.265498136	-1.240649677	0.7837173
84484	4/21/2015 10:02	B	371440981	14034908.95	-73.133	-41.864	5536	2.435520995	-1.140948271	0.838785339
84484	4/21/2015 10:15	B	10826374.47	38282380.03	-73.095	-41.861	763	2.614528986	-1.051986288	0.877516405
84484	4/21/2015 12:57	2	83509.31196	58453.18804	-73.059	-41.879	9722	2.095569871	-1.009164204	0.745068052
84484	4/21/2015 13:32	B	2968728.055	923644.9448	-73.055	-41.875	2137	2.194567891	-1.008764286	0.769885655
84484	4/21/2015 13:54	B	998074.2829	308322.7171	-73.052	-41.876	1314	2.167260565	-1.022241651	0.766529339

84484	4/21/2015 21:31	B	2788884069	1176157336	-73.126	-41.93	27387	-0.357815554	-1.505573151	0.437717676
84484	4/21/2015 22:31	B	5100482.543	1534069.957	-73.148	-41.911	3609	0.479870235	-1.822652824	0.505530696
84484	4/21/2015 23:14	B	3270597.859	287147.1413	-73.146	-41.912	2613	0.479870235	-1.788607893	0.505530696
84484	4/21/2015 23:59	B	4902229.074	464988.9256	-73.138	-41.92	2668	-0.008874034	-1.692685742	0.466039088
84484	4/23/2015 8:08	B	345657544.4	705000255.6	-73.124	-41.883	115755	-0.098179073	-1.296811805	0.279245244
84484	4/23/2015 9:15	B	2560985.718	1720328.782	-73.123	-41.882	4012	-0.120575101	-1.296811805	0.273065077
84484	4/23/2015 9:55	B	2079657.581	513502.419	-73.117	-41.875	2373	-0.155348386	-1.181890038	0.25229095
84484	4/23/2015 10:55	B	3631552.924	486977.0756	-73.113	-41.871	3619	-0.194330637	-1.151349631	0.241674619
84484	4/23/2015 14:54	B	5894935.941	1819566.559	-73.088	-41.857	14321	-0.462707102	-1.039781621	0.187859681
84484	4/23/2015 21:28	B	1998513451	1510196361	-73.245	-41.807	23686	-0.654645225	-0.707799319	0.736584013
84484	4/23/2015 22:10	B	1293229.125	3533943.375	-73.257	-41.81	2512	-0.675067449	-0.698665569	0.73189139
84484	4/23/2015 23:47	B	7773243.679	2749274.821	-73.243	-41.796	5837	-0.51952365	-0.642074301	0.745920391
84484	4/25/2015 9:24	A	51550.75974	1076882.24	-73.127	-41.841	121009	-0.618115502	-0.990749516	0.206742027
84484	4/25/2015 10:09	B	665294.9643	604577.5357	-73.122	-41.836	2689	-0.555707311	-0.983684138	0.193994468
84484	4/25/2015 11:07	B	1179043.544	18643305.46	-73.165	-41.843	3490	-0.457427132	-1.006847017	0.249575393
84484	4/25/2015 11:29	A	744814054	14716563.01	-73.137	-41.849	1324	-0.760560289	-1.022228083	0.230519247

84484	4/25/2015 13:07	B	28243472.14	1570532.86	-73.174	-41.855	5873	-0.18375155	-1.102937972	0.284591171
84484	4/25/2015 14:04	B	7393531.287	537659.213	-73.188	-41.849	3403	0.147790498	-1.034528786	0.310652266
84484	4/25/2015 14:51	B	5344984.004	559318.4958	-73.189	-41.846	2816	0.130474461	-1.020522491	0.300746074
84484	4/25/2015 20:32	B	9640066.332	2139206.168	-73.222	-41.832	20447	0.520080671	-0.874142242	0.327329719
84484	4/25/2015 21:46	B	45876691.6	2444110.902	-73.209	-41.824	4477	-0.115056171	-0.813571056	0.271992101
84484	4/25/2015 22:22	A	76029.02922	7220.97078	-73.153	-41.846	2127	-0.6585129	-1.015312707	0.239671242
84484	4/25/2015 22:47	0	12020411.29	3448491.207	-73.149	-41.845	1497	-0.720615188	-1.013555237	0.234139017
84484	4/25/2015 23:31	B	323174726.5	52757523.5	-73.124	-41.847	2646	-0.654035656	-1.013325174	0.216619432
84484	4/27/2015 9:04	1	8706436.485	402189.5146	-73.121	-41.874	120816	-0.559117192	-1.221580932	-0.376327868
84484	4/27/2015 9:14	3	104512.5497	23553.45028	-73.123	-41.874	581	-0.589207379	-1.222140637	-0.37631345
84484	4/27/2015 10:27	1	264946.8589	167306.1411	-73.117	-41.88	4349	-0.328491711	-1.238464906	-0.364968301
84484	4/27/2015 10:43	A	5753972.296	2622232.704	-73.091	-41.874	962	-0.142066795	-1.075560437	-0.381539933
84484	4/27/2015 10:57	B	3097288.077	879371.9226	-73.103	-41.881	859	-0.048598388	-1.164846751	-0.361980453
84484	4/27/2015 12:06	A	15353342.81	591221.6916	-73.171	-41.858	4146	-1.179186151	-1.117642175	-0.372067993
84484	4/27/2015 12:38	B	8089691.523	1830062.977	-73.177	-41.855	1918	-1.177358636	-1.111886336	-0.371617859
84484	4/27/2015 20:19	1	140212596.6	22891224.43	-73.218	-41.832	27635	-0.734602954	-0.875091329	-0.34760124

84484	4/27/2015 21:19	A	1803434.544	556227.9565	-73.16	-41.857	3625	-1.167229087	-1.093443519	-0.373378036
84484	4/27/2015 21:26	B	532679.5223	132966.9777	-73.159	-41.857	398	-1.148990896	-1.10351417	-0.373673786
84484	4/27/2015 21:42	2	2707785.678	54432.32171	-73.156	-41.849	957	-1.16210618	-1.03968828	-0.376712954
84484	4/27/2015 21:55	B	763536.5192	220692.4808	-73.16	-41.857	782	-1.167229087	-1.093443519	-0.373378036
84484	4/27/2015 22:59	2	946134.5162	26717.98379	-73.132	-41.848	3856	-1.029464259	-1.029211463	-0.394805397
84484	4/27/2015 23:10	2	387785.7081	80799.29195	-73.135	-41.85	655	-1.055956779	-1.043276318	-0.388946792
84484	4/27/2015 23:24	A	193123.8766	25598.62343	-73.126	-41.855	879	-0.93057928	-1.050240867	-0.395890325
84484	4/29/2015 8:39	2	451313.2125	165643.2875	-73.107	-41.875	119700	0.230568743	-1.150190243	0.456063699
84484	4/29/2015 10:08	B	56578568.24	45870514.26	-73.115	-41.879	5305	0.445872651	-1.238464906	0.448561693
84484	4/29/2015 10:20	A	1907555.333	807541.1665	-73.154	-41.876	719	0.294910915	-1.448609729	0.344505501
84484	4/29/2015 11:06	A	4195027.006	3389.994222	-73.135	-41.863	2744	-0.236565468	-1.166086407	0.357308064
84484	4/29/2015 13:28	1	45229096.3	136359.6997	-73.186	-41.857	8553	-0.564522891	-1.112978006	0.283815806
84484	4/29/2015 14:24	B	6520529.716	539696.2836	-73.127	-41.871	3380	0.090129071	-1.241263548	0.393236307
84484	4/29/2015 20:58	B	18730700.09	13190882.41	-73.16	-41.86	23637	-0.482801129	-1.162504643	0.315114836
84484	4/29/2015 21:29	B	13864931	3558219.504	-73.172	-41.883	1841	0.301361432	-1.659320003	0.298665788
84484	4/29/2015 22:21	0	4995593692	225706873.2	-73.097	-41.889	3110	0.709148954	-1.208979933	0.510770746

84484	4/29/2015 22:37	A	99542.73185	183422.2681	-73.089	-41.888	961	0.370581989	-1.104735758	0.539155485
84484	4/30/2015 0:00	B	1995966.374	531398.1257	-73.061	-41.9	4984	-0.444337464	-0.997267799	0.422367736
84484	5/1/2015 8:18	2	75879.04332	24243.45668	-73.113	-42.028	116261	0.512683233	-0.733766231	0.27317564
84484	5/1/2015 9:17	B	7083871.962	917433.0378	-73.104	-42.045	3555	0.446008459	-0.656929122	0.349036349
84484	5/1/2015 9:57	1	317361.375	762859.125	-73.16	-42.021	2409	-0.30156327	-0.902531581	0.326678203
84484	5/1/2015 10:57	B	21478854.98	4556302.023	-73.173	-42.021	3587	-0.462001218	-0.969944621	0.348467022
84484	5/1/2015 11:39	B	393736659.8	36847180.17	-73.187	-42.014	2544	-0.51350641	-1.023462312	0.37320633
84484	5/1/2015 12:48	B	4676458.253	1861002.247	-73.148	-41.982	4149	-0.14169922	-1.216907791	0.246396181
84484	5/1/2015 13:44	B	2833513.873	613654.6273	-73.149	-41.97	3346	-0.086890876	-1.358044428	0.257189976
84484	5/1/2015 14:28	B	4467352.861	1280391.139	-73.146	-41.965	2652	-0.090629473	-1.425281075	0.259231342
84484	5/1/2015 20:38	B	13945476.29	875803.7052	-73.149	-41.883	22201	-0.970272403	-1.51712777	0.419325084
84484	5/1/2015 21:32	B	40724570.48	639009172	-73.265	-41.934	3196	-0.383578728	-1.327911027	0.609585235
84484	5/1/2015 22:21	B	16710121.38	161277283.1	-73.298	-41.933	2969	-0.533087946	-1.272638373	0.685434813
84484	5/1/2015 22:58	A	309192.3591	23406.14091	-73.285	-41.926	2234	-0.418789868	-1.246505946	0.655497035
84484	5/1/2015 23:11	B	359462.7746	66701.72541	-73.282	-41.929	777	-0.452805152	-1.240125554	0.653435362
84484	5/3/2015 8:26	A	31323332.86	55072013.14	-73.349	-42.009	119657	0.237379513	-1.945279005	0.485494771

84484	5/3/2015 9:35	B	79381109.6	69029726.9	-73.346	-41.987	4178	0.222178622	-1.7263056	0.564220584
84484	5/3/2015 10:05	1	5751053.712	373386.7883	-73.311	-41.996	1804	0.197679804	-1.5201322	0.488838395
84484	5/3/2015 10:39	B	27854471.38	61219933.12	-73.339	-41.986	2023	0.267308411	-1.700346982	0.565161873
84484	5/3/2015 11:18	B	9560404.568	8013845.432	-73.334	-41.995	2320	0.268511522	-1.641802696	0.519869198
84484	5/3/2015 12:06	B	18987176.42	8061892.084	-73.34	-41.991	2883	0.256761135	-1.700113428	0.532684766
84484	5/3/2015 12:20	B	10131953.01	2019489.487	-73.36	-41.986	836	0.083415295	-1.892372241	0.581761554
84484	5/3/2015 13:01	B	2420312.94	220203.5596	-73.377	-41.996	2489	-0.102531379	-1.868210848	0.546847463
84484	5/3/2015 13:44	A	12348426.04	838760.4588	-73.328	-41.987	2553	0.297220959	-1.553715321	0.542099515
84484	5/3/2015 20:17	B	34506544.68	5458069.819	-73.283	-42.007	23585	-0.14342528	-1.635386175	0.417463192
84484	5/3/2015 20:46	B	124467669.2	3910311.825	-73.25	-42.047	1733	-0.841014605	-1.72545065	0.316437692
84484	5/3/2015 21:51	A	878652.0434	159072.4566	-73.303	-42.063	3906	-0.77813549	-2.711629652	0.281416734
84484	5/3/2015 21:55	A	239764.875	80893.625	-73.309	-42.068	261	-0.76473221	-2.810897214	0.277836174
84484	5/3/2015 22:21	B	569128.5189	110829.9811	-73.307	-42.067	1551	-0.780101549	-2.784366412	0.278107758
84484	5/3/2015 23:38	B	96395542.3	17370506.2	-73.27	-42.017	4607	-0.38539904	-1.681202859	0.379471501
84484	5/3/2015 23:57	B	8606598.711	1185631.789	-73.217	-42.04	1128	-1.016208349	-1.205554457	0.326723436
84484	5/5/2015 8:04	A	10355.62722	109706.8728	-73.259	-41.891	115648	0.882757916	-1.276898488	0.0068075

84484	5/5/2015 9:07	B	1055604.5	366368	-73.259	-41.885	3776	1.303577555	-1.21998544	-0.02704975
84484	5/5/2015 9:14	A	433380.5	800	-73.246	-41.877	449	1.867002979	-1.296718364	-0.07383159
84484	5/5/2015 9:37	B	3860271.425	101951.0747	-73.256	-41.876	1352	2.122443306	-1.190038134	-0.093868743
84484	5/5/2015 10:52	B	729858.5	2060694.5	-73.224	-41.877	4497	1.916931686	-1.427609523	-0.055348778
84484	5/5/2015 10:57	2	189776.8101	87071.68994	-73.234	-41.885	325	1.272254483	-1.483386602	-0.008273273
84484	5/5/2015 11:14	1	6203069.464	389078.536	-73.242	-41.89	1012	0.614277173	-1.493116857	0.024238713
84484	5/5/2015 13:07	A	707893.2246	180469.2754	-73.246	-41.918	6742	-0.603046261	-1.539333579	0.053222231
84484	5/5/2015 14:06	B	31953167.64	9494212.864	-73.219	-41.881	3550	1.833561363	-1.577715445	-0.045469077
84484	5/5/2015 14:43	A	5386301.693	26930068.31	-73.237	-41.944	2216	-0.392159898	-1.543543386	0.043726705
84484	5/5/2015 20:26	B	14205362.4	33212362.6	-73.253	-41.951	20577	-0.639127873	-1.393218829	0.064552979
84484	5/5/2015 20:54	1	521317.4177	3805735.082	-73.28	-41.911	1680	-0.335797228	-1.241078257	0.0093487
84484	5/5/2015 21:27	B	87031075.15	3934044.851	-73.284	-41.898	2018	0.498915008	-1.200393912	-0.011657162
84484	5/5/2015 21:33	B	18515835.55	878097.4477	-73.29	-41.899	337	0.212588772	-1.207723152	-0.011314796
84484	5/5/2015 22:32	2	781550.8596	3762.140391	-73.213	-41.918	3542	0.340255328	-1.940527748	-0.020192127
84484	5/5/2015 23:08	1	236730.5	238004	-73.2	-41.931	2158	0.613185158	-1.914198629	-0.072243076
84484	5/5/2015 23:13	2	240961.5856	23762.41437	-73.199	-41.926	290	0.746498512	-1.953659412	-0.085699618

84484	5/7/2015 10:09	2	462765.0995	53599.9005	-73.241	-41.936	125778	-0.113291513	-1.561240107	-0.288376166
84484	5/7/2015 10:32	B	10814826.07	1599686.435	-73.242	-41.929	1409	-0.021700505	-1.565611105	-0.295582311
84484	5/7/2015 11:52	2	347277.2092	171738.7908	-73.268	-41.967	4771	-0.792379665	-1.289895794	-0.213810907
84484	5/7/2015 12:25	B	664554.5155	172434.4845	-73.284	-41.961	1985	-0.903491843	-1.287211934	-0.197713271
84484	5/7/2015 14:07	B	4947242.413	302503.587	-73.332	-41.925	6102	-0.338016594	-1.735192709	-0.244845335
84484	5/7/2015 20:01	B	61835212.89	343157315.1	-73.36	-41.853	21244	-0.146383596	-1.072541796	-0.449672203
84484	5/7/2015 21:08	B	5806225.384	900864.616	-73.403	-41.892	4015	-0.048589756	-1.586663867	-0.347084967
84484	5/7/2015 21:33	B	2922444.719	237409.7807	-73.401	-41.886	1497	-0.105956649	-1.533756522	-0.376668704
84484	5/7/2015 21:40	B	1984346.153	3020005.847	-73.405	-41.887	447	-0.080476852	-1.499626714	-0.369004003
84484	5/7/2015 22:24	2	425191.8805	29708.11946	-73.396	-41.892	2633	-0.083735613	-1.65334861	-0.347960245
84484	5/7/2015 22:44	2	977754.038	56660.46201	-73.383	-41.879	1224	-0.199321068	-1.498770118	-0.378109849
84484	5/7/2015 23:10	2	95937.49126	18723.00874	-73.418	-41.86	1530	-0.425470512	-1.042383417	-0.453901197
84484	5/8/2015 0:00	B	25917794.84	1734570.161	-73.429	-41.86	2994	-0.485782362	-1.000963086	-0.312658395
84484	5/9/2015 8:24	2	75853.41737	140021.0826	-73.24	-41.916	116650	2.621704136	-1.666936272	-0.469095287
84484	5/9/2015 9:15	B	75474509	129685.004	-73.246	-41.92	3079	2.77735683	-1.525366084	-0.422840725
84484	5/9/2015 10:06	0	6810454.897	15956259.6	-73.259	-41.909	3037	2.546208147	-1.36882891	-0.288432473

84484	5/9/2015 10:49	1	6889753.549	1524986.451	-73.236	-41.934	2556	3.012028246	-1.585942196	-0.525343814
84484	5/9/2015 10:57	B	5226572.685	1395035.815	-73.247	-41.925	526	2.863065198	-1.488511713	-0.407534912
84484	5/9/2015 12:29	B	63241780.83	223494591.7	-73.239	-41.938	5511	3.047813681	-1.574740038	-0.492071378
84484	5/9/2015 12:34	B	24768420.97	6611023.53	-73.245	-41.937	317	3.022246077	-1.493901522	-0.42621001
84484	5/9/2015 13:21	B	2354450	1064340.5	-73.245	-41.936	2814	3.013541521	-1.472064193	-0.421201255
84484	5/9/2015 14:15	B	2604596.737	1458709.263	-73.238	-41.929	3241	2.96043217	-1.613163959	-0.486803919
84484	5/9/2015 20:49	B	36629495.24	8031884.765	-73.232	-41.908	23601	2.315907245	-1.819044457	-0.520677859
84484	5/9/2015 21:19	A	23654892.34	125142.1631	-73.216	-41.909	1826	2.332254844	-1.976632811	-0.66304391
84484	5/9/2015 21:33	A	55326.12027	370486.3797	-73.167	-41.909	810	3.312183548	-2.017094693	-1.00450475
84484	5/9/2015 22:05	B	25592891.12	7864366.883	-73.189	-41.938	1955	3.197207972	-1.864573387	-1.000503415
84484	5/9/2015 23:13	2	461461.8784	3924.621561	-73.193	-41.949	4081	3.299976232	-1.795564129	-0.994378234
84484	5/9/2015 23:50	A	27732770.03	6600452.47	-73.206	-41.941	2182	3.178916555	-1.803683777	-0.859976796
84484	5/11/2015 9:50	B	210494999	2435946.005	-73.273	-41.954	122444	1.569317351	-1.271901759	-0.183097548
84484	5/11/2015 10:11	A	107728.6651	4186184.335	-73.318	-41.961	1225	0.071811425	-1.477712627	0.02201643
84484	5/11/2015 11:28	B	5538851.313	1803937.187	-73.329	-41.964	4659	-0.283120035	-1.710697531	0.048692774
84484	5/11/2015 12:42	B	9251613.706	574516.2938	-73.327	-41.946	4414	-0.312991839	-1.676236305	0.066200748

84484	5/11/2015 14:23	B	893968.6605	26822467.84	-73.068	-41.925	6068	0.352644474	-1.003287483	-1.046932721
84484	5/11/2015 20:25	B	84161731.76	233044486.7	-72.824	-41.897	21693	-0.682234921	-1.845208182	-0.442356936
84484	5/11/2015 20:44	B	5720923.045	13277478.95	-72.811	-41.896	1177	-0.713657989	-1.749291156	-0.432251676
84484	5/11/2015 22:01	B	410326649.1	2007007.942	-72.806	-41.852	4622	-0.876564239	-2.654729238	-0.422395971
84484	5/11/2015 22:24	1	8491066.153	803332.347	-72.84	-41.86	1343	-0.733445855	-2.828295173	-0.454185016
84484	5/11/2015 22:44	2	814890.1916	27270.80841	-72.83	-41.856	1219	-0.77303984	-2.802385723	-0.44216181
84484	5/11/2015 23:43	1	138829.952	194982.048	-72.865	-41.862	3516	-0.652585471	-2.930052169	-0.495179795
84484	5/11/2015 23:59	A	1342490.432	390574.5675	-72.853	-41.87	992	-0.659124786	-2.724742617	-0.472528851
84484	5/13/2015 8:17	B	156334841.8	118147262.7	-72.768	-42.015	116283	-0.710008915	-1.874014085	-0.52611256
84484	5/13/2015 9:19	B	133128.4033	10962.0967	-72.865	-41.955	3698	-0.623123758	-1.419623669	-0.323072993
84484	5/13/2015 10:25	B	8162537.341	1477967.659	-72.882	-41.935	3992	-0.605433695	-1.475775879	-0.275572452
84484	5/13/2015 10:57	B	2151289.978	277547.022	-72.889	-41.928	1896	-0.592457507	-1.532352329	-0.24591688
84484	5/13/2015 12:01	B	624753928.5	15672909.98	-72.851	-41.991	3853	-0.651276241	-1.721514903	-0.397203106
84484	5/13/2015 12:04	B	90628988.11	2288756.894	-72.85	-41.991	143	-0.652335669	-1.737781606	-0.400738247
84484	5/13/2015 12:54	B	14838260.09	469402.414	-72.852	-41.993	3045	-0.651994874	-1.734824193	-0.399346017
84484	5/13/2015 13:41	B	7759247.971	359666.0294	-72.855	-41.991	2817	-0.649372435	-1.704810381	-0.392572413

84484	5/13/2015 20:04	1	20249773.3	7932569.203	-72.799	-42.088	22971	-0.733623694	-2.744957759	-0.600253966
84484	5/13/2015 20:33	1	562934.2536	443737.7464	-72.715	-42.038	1758	-0.753772751	-2.219092466	-0.622612762
84484	5/13/2015 21:33	1	920638.1032	1518764.397	-72.672	-42.041	3584	-0.784799807	-1.713096403	-0.666518762
84484	5/13/2015 21:39	1	7941901.742	231922.7584	-72.678	-42.046	326	-0.783073382	-1.894722984	-0.666739505
84484	5/13/2015 23:14	3	64188.375	25284.125	-72.665	-42.091	5698	-0.805417032	-2.371483467	-0.737439353
84484	5/13/2015 23:18	2	151628.2388	10540.26123	-72.667	-42.092	240	-0.805417032	-2.43798165	-0.737439353
84484	5/15/2015 8:28	A	5384287.423	64230100.58	-72.97	-42.191	119448	-0.262875517	-1.923621182	2.926922681
84484	5/15/2015 8:55	B	5001417.294	8822463.206	-72.967	-42.197	1597	-0.261465731	-2.02501799	2.904864011
84484	5/15/2015 10:41	B	51496979.8	1971166.199	-72.964	-42.224	6346	-0.253595007	-2.665127532	2.824051731
84484	5/15/2015 12:36	B	38732839.07	16486991.43	-73.082	-42.188	6892	-0.269653723	-1.064796125	3.005816956
84484	5/15/2015 13:54	A	7341890930	228271408.1	-73.023	-42.135	4690	-0.285202777	-0.973258635	3.114242678
84484	5/15/2015 14:43	B	54014520.48	3665160.021	-73.011	-42.131	2966	-0.286186904	-0.977113778	3.117459117
84484	5/15/2015 22:25	A	56297730.38	301374.6235	-73.031	-42.512	27684	-0.131560967	-0.683878232	2.037632438
84484	5/15/2015 23:58	B	13448995.96	73491742.04	-73.014	-42.364	5611	-0.19305995	-1.032702739	2.461302402
84484	5/17/2015 9:21	A	1039978027	15775841.06	-72.577	-42.177	120197	-0.438927461	-1.764432714	-0.239535816
84484	5/17/2015 9:57	B	41632534	104282390.5	-72.553	-42.192	2161	-0.465270417	-1.312120853	-0.290185699

84484	5/17/2015 10:11	B	1895873.687	117426.3127	-72.543	-42.174	817	-0.460889638	-1.349916166	-0.265932448
84484	5/17/2015 10:59	A	85338.88421	285078.1158	-72.543	-42.167	2855	-0.456379356	-1.415629332	-0.250325778
84484	5/17/2015 11:35	B	24432824.47	3237940.527	-72.541	-42.17	2197	-0.460225226	-1.374962025	-0.259666509
84484	5/17/2015 12:21	B	55231610.35	5833210.654	-72.545	-42.164	2730	-0.453751249	-1.43639672	-0.240927449
84484	5/17/2015 14:02	B	3775006.746	592395.2538	-72.543	-42.17	6084	-0.458847972	-1.383989146	-0.257369979
84484	5/17/2015 14:51	B	138404981.3	138404981.3	-72.562	-42.166	2956	-0.443057469	-1.622952233	-0.230054774
84484	5/17/2015 21:20	A	113065454.4	7030229.568	-72.526	-42.182	23289	-0.475762281	-1.162494845	-0.298470062
84484	5/17/2015 21:37	B	12714889.4	2352895.104	-72.562	-42.187	1042	-0.454841658	-1.434509618	-0.272868935
84484	5/17/2015 22:35	A	891293.7237	106378.2763	-72.551	-42.182	3505	-0.458525379	-1.390494688	-0.271225786
84484	5/17/2015 22:55	3	51030.67072	5653.829283	-72.548	-42.175	1188	-0.456845055	-1.377227603	-0.26221552
84484	5/17/2015 23:15	2	272384.9138	120313.0862	-72.545	-42.177	1211	-0.459805618	-1.354015547	-0.271698817
84484	5/19/2015 8:10	2	116604.6966	24192.30338	-72.671	-42.183	118496	-0.741158631	-3.086349387	-0.523245997
84484	5/19/2015 8:32	A	893056.1627	28408.83727	-72.66	-42.186	1278	-0.753761074	-2.983849517	-0.531908532
84484	5/19/2015 9:50	1	6260635.648	74396.85246	-72.715	-42.202	4705	-0.758957679	-2.868115844	-0.54313139
84484	5/19/2015 10:10	A	1660880.786	15771.71435	-72.702	-42.193	1227	-0.746940288	-2.959259166	-0.528892341
84484	5/19/2015 12:13	B	2030837.365	565173.1353	-72.724	-42.205	7379	-0.752599824	-2.91057298	-0.54040499

84484	5/19/2015 13:21	B	4779121.991	730304.5094	-72.73	-42.2	4027	-0.746748273	-2.995206065	-0.536118218
84484	5/19/2015 14:06	B	5363846.252	1165822.748	-72.744	-42.21	2716	-0.757242848	-3.027757252	-0.543869712
84484	5/19/2015 14:55	B	111953366.6	2829307.89	-72.718	-42.187	2981	-0.725775756	-3.027757252	-0.514851633
84484	5/19/2015 21:05	B	15481621.06	1740637.444	-72.748	-42.161	22167	-0.641994773	-3.086349387	-0.457824385
84484	5/19/2015 22:10	0	11894466.57	5172453.926	-72.652	-42.221	3885	-0.815016946	-2.224496773	-0.571205705
84484	5/19/2015 22:26	A	284217.0356	106619.4644	-72.687	-42.217	950	-0.783156537	-2.382798752	-0.563030445
84484	5/19/2015 23:31	2	118646.9587	176699.5413	-72.636	-42.206	3911	-0.787444858	-2.561271258	-0.559250555
84484	5/19/2015 23:53	A	643310.0817	848950.4183	-72.633	-42.2	1339	-0.779367435	-2.655483608	-0.555891222
84484	5/21/2015 9:17	1	174774.1152	158868.3848	-72.67	-42.204	120242	-0.127260608	-2.625667774	-0.587335225
84484	5/21/2015 9:32	B	6370515.69	23774.80986	-72.67	-42.199	897	-0.103485689	-2.775405999	-0.577310227
84484	5/21/2015 12:37	B	11429133.83	2830942.671	-72.686	-42.214	11119	-0.12879816	-2.484388121	-0.607216892
84484	5/21/2015 14:15	B	19193125.7	1279175.3	-72.668	-42.174	5875	0.005698425	-3.086349387	-0.490157758
84484	5/21/2015 20:50	A	949924947.9	206593938.6	-72.843	-42.008	23709	0.10163763	-1.882668365	0.207698877
84484	5/21/2015 21:53	B	82972429.21	21044730.79	-72.851	-42	3746	0.083519082	-1.750553354	0.241807063
84484	5/21/2015 22:35	B	50657509.41	4039217.089	-72.82	-41.951	2509	0.146755526	-1.424813697	0.399555648
84484	5/21/2015 23:16	B	159472438.7	8751819.261	-72.808	-41.947	2460	0.170186436	-1.365640525	0.421932098

84484	5/23/2015 10:10	A	3700797.091	14735.90879	-73.255	-42.165	125651	-0.022519971	-2.073519122	-0.576511732
84484	5/23/2015 11:49	B	73159348.26	10841061.74	-73.26	-42.208	5945	0.548782236	-2.014489474	-0.755472031
84484	5/23/2015 11:56	B	15723005.06	1634459.937	-73.253	-42.212	417	0.633287479	-1.951465264	-0.769586815
84484	5/23/2015 12:48	B	183909.7678	1020044.732	-73.248	-42.215	3117	0.720033829	-1.844350643	-0.779754729
84484	5/23/2015 13:38	B	821506.4742	735446.0258	-73.242	-42.216	2996	0.778593478	-1.822192106	-0.78075344
84484	5/23/2015 14:28	B	6109452.005	5314524.495	-73.232	-42.232	3015	0.990663238	-1.780373199	-0.869366846
84484	5/23/2015 20:21	A	1273778.143	7573.85722	-73.225	-42.49	21177	2.391393479	-0.649840226	-1.681524199
84484	5/23/2015 21:26	B	3685304.734	614557.7664	-73.219	-42.506	3918	2.959307337	-0.590674931	-1.568038491
84484	5/23/2015 21:33	B	2185660.306	606661.6941	-73.222	-42.506	418	2.87135665	-0.587125352	-1.573462959
84484	5/23/2015 22:04	B	2355092.854	1009403.646	-73.224	-42.501	1870	2.763767737	-0.610926294	-1.594945108
84484	5/23/2015 22:20	B	2486965.329	520187.171	-73.226	-42.519	967	2.182980612	-0.526112076	-1.415526991
84484	5/25/2015 8:46	A	5266790188	776116374.1	-73.348	-42.732	123915	0.226604758	0.081674196	-1.912903402
84484	5/25/2015 9:23	A	2191804229	204885529.5	-73.344	-42.753	2238	0.28000953	0.092067241	-1.963721507
84484	5/25/2015 10:29	B	4022418.773	404167.2271	-73.35	-42.77	3953	-0.022572496	0.104283696	-2.040422618
84484	5/25/2015 10:42	A	128244275.2	13601997.78	-73.364	-42.737	773	0.091738211	0.106047141	-1.956897684
84484	5/25/2015 11:01	B	62919847.59	6039513.412	-73.366	-42.742	1133	0.001632954	0.109961829	-1.977501915

84484	5/25/2015 11:15	B	17341668.26	1761067.738	-73.364	-42.741	877	0.026158415	0.107990412	-1.972047319
84484	5/25/2015 12:55	A	33987435.26	99273.23518	-73.317	-42.792	5978	0.25305167	0.056814593	-2.093440197
84484	5/25/2015 13:46	A	235706189.8	19384419.23	-73.302	-42.816	3089	0.44545458	0.025521612	-2.183232956
84484	5/25/2015 14:32	B	100218422.2	280754.8055	-73.308	-42.81	2723	0.344163584	0.039195748	-2.170132478
84484	5/25/2015 20:03	B	28643549.53	762962.9715	-73.311	-42.799	19866	0.301109663	0.045393298	-2.124498668
84484	5/25/2015 21:02	B	10040008.07	1198766.427	-73.286	-42.817	3529	0.631220444	-0.003912848	-2.184691768
84484	5/25/2015 21:39	B	4295503.648	1255648.852	-73.272	-42.818	2244	0.85502005	-0.021770662	-2.178169316
84484	5/25/2015 21:45	B	3958924.139	1329238.361	-73.269	-42.815	327	0.827987796	-0.022827168	-2.15168136
84484	5/25/2015 22:38	A	5287811.637	8014.363415	-73.214	-42.767	3223	1.51315672	-0.131220781	-1.714748904
84484	5/25/2015 23:14	A	20904112.71	1502689.786	-73.21	-42.778	2151	1.740445715	-0.144822972	-1.732395601
84484	5/25/2015 23:41	B	20072448	331298	-73.212	-42.789	1602	1.922472942	-0.142311884	-1.789124151
84484	5/27/2015 8:21	1	2621560.117	254570.3834	-73.233	-43.043	117627	0.276468432	-0.034766086	-1.452768895
84484	5/27/2015 8:32	B	226981.4537	251083.0463	-73.237	-43.043	655	0.265512844	-0.029180787	-1.431343686
84484	5/27/2015 10:02	B	4938332.856	2615837.144	-73.228	-43.029	5381	0.27453439	-0.024135639	-1.481012157
84484	5/27/2015 13:51	B	26046966009	392706015.8	-73.236	-42.761	13725	-0.548310046	-0.092278811	-1.547350733
84484	5/27/2015 20:38	B	816265777.9	80703984.06	-73.184	-42.684	24462	-0.970008818	-0.212469959	-1.374141024

84484	5/27/2015 22:30	2	6835519.871	3764.128882	-73.226	-42.68	6679	-0.847882702	-0.109897621	-1.406488442
84484	5/27/2015 22:41	A	565875.8581	1054.141928	-73.222	-42.658	677	-0.98087567	-0.12547306	-1.385544845
84484	5/29/2015 9:18	0	692685.1748	11189559.83	-73.161	-42.404	124619	-0.479665305	-0.786023082	-0.005883818
84484	5/29/2015 9:32	B	3391304.308	2712575.692	-73.159	-42.4	875	-0.480723026	-0.782271177	-0.001606039
84484	5/29/2015 10:25	B	10953618.89	4585193.613	-73.15	-42.39	3159	-0.490018644	-0.773810194	0.001626823
84484	5/29/2015 11:00	A	94658279.44	5085381.558	-73.165	-42.381	2106	-0.488955431	-0.847685987	0.034438657
84484	5/29/2015 13:10	B	1425920.395	16582210.1	-73.098	-42.359	7809	-0.526444041	-0.708991284	-0.025199082
84484	5/29/2015 14:02	B	8179588705	1300.5	-73.168	-42.376	3126	-0.498244407	-0.77097297	0.018605429
84484	5/29/2015 14:54	B	39702880.8	338216.2014	-73.141	-42.368	3109	-0.503517185	-0.763592505	0.022875511
84484	5/29/2015 20:54	A	71565152.07	89252.93048	-73.13	-42.379	21567	-0.503039112	-0.726895122	-0.009496897
84484	5/29/2015 21:38	B	7921840.04	242464.9596	-73.127	-42.373	2680	-0.509109031	-0.73199434	-0.006133548
84484	5/29/2015 21:42	A	16055449.18	1320823.823	-73.122	-42.367	229	-0.513017341	-0.72651133	-0.005730897
84484	5/29/2015 23:19	A	81736.57217	32065.92783	-73.106	-42.357	5776	-0.524344011	-0.72416486	-0.009243231
84484	5/29/2015 23:20	B	156058.2569	6794.743144	-73.106	-42.354	108	-0.526001677	-0.722823866	-0.002455052
84484	5/29/2015 23:38	A	302749.3466	1184969.153	-73.125	-42.36	1032	-0.5182752	-0.75630976	0.011979454
84484	5/31/2015 8:12	B	254408121.7	21176991.28	-72.899	-42.339	117271	-0.571398441	-2.410690302	-0.832898914

84484	5/31/2015 9:21	A	1530936917	30244685.66	-72.975	-42.365	4152	-0.513751642	-1.3932083	-0.810660891
84484	5/31/2015 10:59	A	7741198.877	62877843.62	-73.075	-42.406	5838	-0.562091344	-0.670962426	-0.783367883
84484	5/31/2015 11:01	B	4718775.64	26959789.36	-73.075	-42.406	128	-0.562091344	-0.667387453	-0.783367883
84484	5/31/2015 12:35	B	21339071.49	11027547.01	-73.069	-42.425	5638	-0.566799133	-0.6588745	-0.823399806
84484	5/31/2015 13:25	B	1421575487	116898575.5	-73.085	-42.362	3001	-0.598391945	-0.704122232	-0.690192614
84484	5/31/2015 14:13	B	46072457.19	3920541.312	-73.089	-42.354	2906	-0.609696835	-0.716993592	-0.669211702
84484	5/31/2015 15:02	B	45119839.01	2428200.991	-73.088	-42.351	2954	-0.610734542	-0.718718448	-0.666185115
84484	5/31/2015 20:41	B	35727440.94	4700777.558	-73.113	-42.377	20308	-0.58250653	-0.704067438	-0.68777159
84484	5/31/2015 21:33	B	8046482.591	5914949.909	-73.127	-42.362	3125	-0.591867078	-0.742148982	-0.641189006
84484	5/31/2015 22:15	1	6904942.812	2197142.188	-73.17	-42.434	2537	-0.532029136	-0.7226348	-0.70674416
84484	5/31/2015 22:29	2	1149075.016	95493.98363	-73.17	-42.441	820	-0.530394185	-0.711165768	-0.720125311
84484	5/31/2015 23:17	B	51191.06688	8316601.433	-73.094	-42.453	2902	-0.572239653	-0.566161886	-0.844998185
84484	5/31/2015 23:55	A	972777.7289	736384.7711	-73.114	-42.382	2244	-0.581706304	-0.701873961	-0.69019816
84484	6/2/2015 9:53	0	26076489.35	15133743.65	-73.276	-43.176	122320	-0.464690938	-0.103098693	-1.452502294
84484	6/2/2015 10:30	B	64695249.69	18050950.81	-73.28	-43.182	2207	-0.475335547	-0.101076797	-1.444976039
84484	6/2/2015 11:39	B	198291041.4	5599627.09	-73.286	-43.208	4168	-0.520976123	-0.085737893	-1.421059948

84484	6/2/2015 13:29	1	3771754.875	1335983.625	-73.298	-43.137	6547	-0.403437727	-0.032436368	-1.416907725
84484	6/2/2015 14:25	B	8498307.378	1119723.122	-73.312	-43.241	3369	-0.381793183	-0.031653158	-1.371238642
84484	6/2/2015 20:07	A	452843.7223	1251278.278	-73.807	-43.485	20562	-0.103956214	0.38063558	0.668767476
84484	6/2/2015 21:17	B	43743116.47	7604408.532	-73.877	-43.549	4163	-0.451795365	0.271313703	0.630415958
84484	6/2/2015 21:49	B	20898611.1	3555436.902	-73.898	-43.567	1919	-0.430119029	0.246065494	0.655038244
84484	6/2/2015 22:52	B	36333668.35	1268356.15	-73.953	-43.629	3771	0.057188927	0.136194477	0.79216741
84484	6/2/2015 23:14	B	9783037.094	199585.4061	-73.955	-43.63	1318	0.058852288	0.133724246	0.796834471
84484	6/4/2015 10:14	0	2377586.333	2325137.667	-74.591	-43.364	126003	0.297887045	-0.747314964	1.976438227
84484	6/4/2015 12:09	B	276488777.7	90860360.25	-74.652	-43.406	6903	-0.663237161	-0.817391624	2.094897365
84484	6/4/2015 14:23	B	1086351433	241389384.1	-74.486	-43.257	8039	0.00386666	-0.468618441	1.857776655
84484	6/4/2015 21:33	B	14542064.78	1156393.223	-74.579	-43.078	25824	-0.303605366	-0.216183896	1.832093002
84484	6/4/2015 22:34	B	16062078.77	1285273.726	-74.582	-43.057	3667	-0.235718419	-0.171512229	1.843588352
84484	6/4/2015 23:31	B	328215992.2	149341415.8	-74.504	-42.813	3399	-0.28562775	-0.190818451	1.963723654
84485	4/13/2015 20:42	B	54084.87788	2154400.122	-73.299	-42.128	4257	0.20791559	-2.842544269	0.133834096
84485	4/13/2015 22:27	B	93530378.28	2910414.218	-73.296	-42.126	6303	0.246515405	-2.828801312	0.11405517
84485	4/13/2015 23:52	B	7802773.293	6331837.207	-73.303	-42.126	5121	0.321460794	-2.870526833	0.143783713

84485	4/14/2015 3:13	B	13384691.53	772604.9683	-73.283	-42.086	12041	-0.209046291	-2.679052975	0.244846841
84485	4/14/2015 3:56	A	45796.56353	6605.936465	-73.336	-42.079	2556	-0.835479259	-2.724722486	0.247618518
84485	4/14/2015 5:20	B	2285763.823	773344.1773	-73.357	-42.069	5041	-0.852057171	-2.421341931	0.277169034
84485	4/14/2015 6:56	B	3979046.699	729430.3012	-73.378	-42.059	5766	-0.858483015	-2.168930152	0.307673822
84485	4/14/2015 9:32	B	9161974.455	1802409.545	-73.402	-42.043	9395	-0.659669481	-2.199799771	0.350681585
84485	4/14/2015 10:35	A	160216.6033	28308.39674	-73.321	-42.103	3768	-0.868222538	-2.847160833	0.216098931
84485	4/14/2015 11:20	B	2986256.223	728768.7771	-73.316	-42.105	2662	-0.867596594	-2.864568579	0.212949713
84485	4/14/2015 13:44	B	5651089.775	1666971.225	-73.295	-42.12	8680	-0.842980496	-2.831267491	0.192302199
84485	4/14/2015 14:32	B	6162605.176	2298847.324	-73.298	-42.129	2862	-0.999247961	-2.829892647	0.186833705
84485	4/14/2015 17:35	A	80790.04915	5708232.451	-73.315	-42.027	10991	-0.75669842	-2.120026279	0.217739397
84485	4/14/2015 19:18	A	846092.4594	9987.540627	-73.331	-41.994	6168	-0.429353652	-1.628216959	0.277218362
84485	4/14/2015 20:30	B	1509957.504	313996.4956	-73.329	-41.985	4297	-0.31461883	-1.562356782	0.300700508
84485	4/14/2015 20:56	B	3880251.944	510100.5556	-73.31	-42.012	1605	-0.694172552	-1.831725706	0.22654028
84485	4/14/2015 21:53	A	57168.77294	44496.22706	-73.315	-42.013	3384	-0.717928475	-1.866826912	0.227531902
84485	4/14/2015 21:57	B	4506554.508	1660762.492	-73.313	-42.005	244	-0.623020502	-1.717045573	0.238889421
84485	4/14/2015 22:16	B	2205536806	83333784.13	-73.311	-41.988	1133	-0.50064891	-1.439616367	0.270157172

84485	4/14/2015 23:35	A	7888017.149	567359.3513	-73.31	-41.989	4791	-0.521949176	-1.46205988	0.263244698
84485	4/14/2015 23:53	B	852428.1612	104635.8388	-73.311	-41.986	1063	-0.48192405	-1.409604795	0.274692499
84485	4/15/2015 5:02	B	19753213.22	187050069.3	-73.397	-41.976	18518	0.025107244	-2.247248432	0.829513815
84485	4/15/2015 6:42	B	265255846.8	31450465.66	-73.393	-41.997	6029	-0.113534531	-1.866651734	0.74802014
84485	4/15/2015 7:57	B	6259097.826	523450.1743	-73.392	-41.997	4469	-0.11428266	-1.866651734	0.748502447
84485	4/15/2015 8:28	B	6227087.823	610397.1773	-73.392	-41.997	1878	-0.11428266	-1.866651734	0.748502447
84485	4/15/2015 9:42	B	6582225.054	846415.4457	-73.398	-41.993	4453	-0.075508846	-1.882142248	0.767001535
84485	4/15/2015 10:09	B	5612287.897	703529.1025	-73.406	-41.988	1600	-0.016871061	-1.92464809	0.799783306
84485	4/15/2015 10:54	A	1698515.745	1820597.255	-73.334	-42.023	2678	-0.451739811	-2.124439789	0.614719879
84485	4/15/2015 11:21	B	1615490.503	631282.4968	-73.327	-42.028	1638	-0.482808211	-2.196601053	0.602563436
84485	4/15/2015 11:41	B	2577899.809	862070.6914	-73.324	-42.03	1179	-0.497761374	-2.172906626	0.598504144
84485	4/15/2015 11:45	B	3377416.939	580395.5607	-73.322	-42.026	286	-0.494558071	-2.139287385	0.60527152
84485	4/15/2015 13:22	B	2493314.376	708514.6245	-73.31	-42.032	5786	-0.557805207	-2.164388589	0.588500657
84485	4/15/2015 17:26	B	7224874.437	1901650.063	-73.264	-42.043	14624	-0.681333943	-1.921589888	0.586745435
84485	4/15/2015 19:07	B	33806942.2	517170.2953	-73.284	-42.022	6105	-0.616111837	-1.869815009	0.617763662
84485	4/15/2015 20:20	B	34692419.69	697152.8106	-73.279	-42.023	4393	-0.620419699	-1.845335027	0.618036809

84485	4/15/2015 20:46	B	10666303.98	1017678.516	-73.299	-42.023	1522	-0.559814938	-2.027327619	0.612709048
84485	4/15/2015 21:58	A	223537.6502	18327.34983	-73.4	-41.979	4303	0.009495848	-2.183630869	0.818868556
84485	4/15/2015 23:11	B	10546750.64	3808435.861	-73.382	-41.994	4389	-0.141997226	-1.885861897	0.741346077
84485	4/16/2015 0:50	B	12363432.28	1111179.715	-73.44	-41.989	5942	0.113212801	-1.816429543	1.406427891
84485	4/16/2015 1:34	B	155377697.9	201167.1363	-73.441	-42.008	2664	0.109936317	-1.747614508	1.344663596
84485	4/16/2015 3:18	B	393839.5343	19288274.47	-73.437	-42.015	6249	0.112354781	-1.807440174	1.313012099
84485	4/16/2015 4:53	B	6310028.375	18906676.13	-73.455	-42.01	5677	0.096679113	-1.628333997	1.367397625
84485	4/16/2015 4:57	B	1703452.802	4650369.698	-73.452	-42.002	220	0.098735129	-1.650456177	1.382176648
84485	4/16/2015 6:33	A	411957458	2073938.008	-73.359	-42.028	5775	0.143655171	-2.267489256	1.10097413
84485	4/16/2015 7:48	B	125067574.7	236852525.8	-73.403	-42.023	4510	0.135440118	-2.141009337	1.209579548
84485	4/16/2015 8:46	B	13864278.41	10075210.59	-73.409	-42.023	3486	0.131931051	-2.119225685	1.216134416
84485	4/16/2015 9:25	B	1937566.496	1163819.504	-73.411	-42.024	2330	0.131931051	-2.105496597	1.216134416
84485	4/16/2015 11:14	B	7996727.733	672293.2669	-73.428	-41.965	6541	0.140586287	-2.38646802	1.475246077
84485	4/16/2015 13:01	B	18814588.73	539927.2736	-73.415	-41.93	6425	0.184223875	-2.184042268	1.5742497
84485	4/16/2015 14:37	B	13281228.03	736980.4726	-73.415	-41.93	5747	0.184223875	-2.184042268	1.5742497
84485	4/17/2015 8:07	B	35312618.49	5325544.011	-73.401	-41.944	63025	-0.55857709	-2.597985311	2.046948927

84485	4/17/2015 9:17	B	35459057.78	90429594.72	-73.389	-41.932	4163	-0.545081605	-2.424787351	2.05159813
84485	4/17/2015 10:54	B	8319412.288	14724557.71	-73.384	-41.926	5868	-0.535805916	-2.393377665	2.056614629
84485	4/17/2015 12:21	B	48794579.23	270777465.8	-73.325	-41.9	5200	-0.499228818	-1.433480587	2.009128723
84485	4/17/2015 12:42	B	2353817.912	8598359.088	-73.317	-41.926	1268	-0.511646959	-1.405995435	1.952408628
84485	4/17/2015 21:34	B	9678509.055	3994127.945	-73.248	-41.926	31894	-0.49386795	-1.488511713	1.843376151
84485	4/17/2015 23:15	B	7488991.285	17737729.71	-73.106	-42.101	6059	-0.594270467	-0.653517956	1.293408425
84485	4/17/2015 23:38	B	1967252.495	1480517.505	-73.109	-42.092	1415	-0.587712508	-0.645030609	1.316902587
84485	4/19/2015 10:58	B	1.46507E+11	5535588116	-73.081	-41.854	127172	-0.525821327	-1.150952647	0.63883317
84485	4/19/2015 12:01	B	408654457.3	32443701.25	-73.015	-41.89	3764	-0.590776293	-1.079358719	0.576384157
84485	4/19/2015 13:41	B	3.04196E+11	11493660285	-72.991	-41.869	6013	-0.703993599	-1.273921829	0.615741727
84485	4/19/2015 14:28	B	179254081.2	8227843.337	-72.995	-41.878	2851	-0.727700309	-1.280121744	0.610492746
84485	4/19/2015 21:18	B	32055382058	2301474139	-73.038	-41.894	24566	-0.549515198	-1.034664909	0.555368578
84485	4/19/2015 21:42	B	6331284.493	3355768.507	-73.015	-41.904	1459	-0.561884906	-1.02268016	0.546182843
84485	4/19/2015 22:35	0	43863745.05	88824.94671	-73.059	-41.851	3164	-0.384328069	-1.100813035	0.654344126
84485	4/19/2015 23:08	A	16151129.95	861614.5513	-73.061	-41.871	1963	-0.343070029	-1.028044368	0.597652292
84485	4/21/2015 10:12	B	51818454.51	314430998	-72.984	-42.237	126283	3.177515975	-2.49416848	-0.429687786

84485	4/21/2015 10:15	B	5912334.588	35504755.41	-72.975	-42.235	157	3.105848916	-2.621318881	-0.346414746
84485	4/21/2015 12:54	B	6603907.515	1410828.485	-72.892	-42.29	9567	1.96002602	-2.617231944	0.165724032
84485	4/21/2015 13:53	B	13986549344	528464418.3	-72.906	-42.265	3506	1.923395224	-2.248154582	0.149921779
84485	4/21/2015 14:35	1	533057.4749	297586.5251	-72.983	-42.272	2564	3.315648135	-1.773361668	-0.417410094
84485	4/21/2015 20:57	B	60536578.15	19445914.35	-73.047	-42.34	22888	1.076272799	-0.830395972	-0.918807805
84485	4/21/2015 21:22	B	54020925.88	11719484.12	-73.045	-42.321	1511	0.262103888	-0.88050157	-0.824121701
84485	4/21/2015 22:31	B	752999907.8	459638497.2	-73.008	-42.365	4137	2.471174943	-1.059050637	-0.986330374
84485	4/23/2015 10:52	B	15051966780	14036786245	-73.019	-41.814	130835	-0.341190454	-1.864636523	-0.793239008
84485	4/23/2015 13:52	B	59733274.56	24449021.94	-72.861	-41.71	10832	-0.145145104	-2.50787777	-0.474957941
84485	4/23/2015 20:29	B	187433517.3	4750087.658	-73.026	-41.83	23791	-0.575660279	-1.532938674	-0.824908976
84485	4/23/2015 20:56	B	1482071824	29277446.9	-73.102	-41.842	1639	0.13497612	-1.028548915	-0.703388217
84485	4/23/2015 21:31	B	45037998.35	98933877.65	-73.172	-41.81	2084	0.633763418	-0.769477456	-0.317829876
84485	4/23/2015 22:10	1	5075535.445	918049.5555	-73.179	-41.828	2326	0.913644776	-0.867946527	-0.304005743
84485	4/23/2015 23:10	B	27067359.56	20456716.94	-73.192	-41.838	3610	0.930874164	-0.956336134	-0.232860737
84485	4/23/2015 23:51	B	6820503.571	1586956.929	-73.171	-41.838	2448	1.115090206	-0.953134288	-0.361635946
84485	4/25/2015 10:08	B	1.23994E+11	10195172001	-73.131	-41.861	123447	-0.457047659	-1.207703731	-0.676333009

84485	4/25/2015 13:05	B	17180216.93	1018940.068	-73.175	-41.847	10645	-0.094557246	-1.0252757	-0.664370819
84485	4/25/2015 13:19	B	158969544.5	21207321.54	-73.153	-41.833	789	-0.526253093	-0.91562275	-0.72969871
84485	4/25/2015 14:10	B	68425717.82	11433384.68	-73.157	-41.815	3112	-0.713120397	-0.818125339	-0.752536922
84485	4/25/2015 14:55	B	23239378.84	2727666.156	-73.185	-41.827	2651	-0.22205093	-0.867274554	-0.693713022
84485	4/25/2015 20:33	B	60082534.57	36736557.93	-73.167	-41.831	20283	-0.45406868	-0.887854136	-0.714314021
84485	4/25/2015 21:51	B	8277065402	312740103.4	-73.212	-41.859	4680	0.541558879	-1.128960404	-0.555117469
84485	4/25/2015 22:17	B	30779049.89	239950.6064	-73.182	-41.851	1565	0.200806207	-1.06362751	-0.630224155
84485	4/25/2015 22:47	A	5677340.748	601821.7521	-73.174	-41.841	1802	-0.103883049	-0.957741764	-0.674660068
84485	4/27/2015 10:41	A	16811.5927	45308.4073	-73.144	-41.854	129272	-1.001206095	-1.060976632	-1.466473414
84485	4/27/2015 10:56	B	116955.1596	590865.3404	-73.157	-41.85	881	-1.092993582	-1.03968828	-1.458859307
84485	4/27/2015 12:08	A	12107.67204	30978.82796	-73.159	-41.846	4309	-1.081342286	-1.022184412	-1.458210091
84485	4/27/2015 13:32	B	1416244.5	453152	-73.159	-41.842	5044	-1.038520358	-0.985947515	-1.459131999
84485	4/27/2015 14:08	B	209041.5702	11807866.93	-73.151	-41.842	2180	-1.013851701	-0.986298736	-1.465918513
84485	4/27/2015 20:11	B	109043159.1	45999304.86	-73.134	-41.82	21794	-0.645658133	-0.879302277	-1.458434521
84485	4/27/2015 21:24	B	7415475.73	998266.7702	-73.167	-41.843	4334	-1.071069439	-1.000702308	-1.4551694
84485	4/27/2015 21:32	B	5444854.678	713443.8215	-73.17	-41.845	525	-1.09808236	-1.012103375	-1.454217262

84485	4/27/2015 21:46	B	397225324.3	7847160.666	-73.181	-41.859	802	-1.088462773	-1.141222625	-1.452522279
84485	4/27/2015 23:12	B	10401360.5	151250	-73.197	-41.875	5149	-0.711897023	-1.525608537	-1.433385003
84485	4/27/2015 23:26	B	158729774.5	23390519.98	-73.195	-41.868	876	-0.858661794	-1.332644166	-1.444009047
84485	4/29/2015 13:34	B	35855644.95	1428807368	-73.173	-41.815	137262	-0.237828262	-0.803848384	-0.710357611
84485	4/29/2015 22:39	A	156882.4579	205164.0421	-73.233	-41.898	32711	0.502104923	-1.728600523	-0.628423251
84485	4/29/2015 23:56	B	1005420.134	571415.8662	-73.245	-41.898	4591	0.433623114	-1.541052537	-0.591556959
84485	5/1/2015 8:21	B	16900464630	4387636868	-73.174	-41.851	116704	-0.965058984	-1.088601974	-0.452985025
84485	5/1/2015 9:15	A	102052.1851	20300.31487	-73.05	-41.924	3262	0.160952171	-0.943117572	-0.452436089
84485	5/1/2015 9:55	B	209050880.6	262983.8822	-73.021	-41.94	2419	0.935144268	-0.870444008	-0.387923849
84485	5/1/2015 10:02	B	513264038.5	792166.5403	-73.025	-41.943	426	0.895627723	-0.866960495	-0.41438016
84485	5/1/2015 10:54	A	81572349.2	400971.3014	-73.021	-41.943	3065	1.027820269	-0.868224501	-0.403894182
84485	5/1/2015 12:44	B	4228464.751	482049.249	-72.987	-41.968	6643	2.529682551	-0.881589518	-0.336130798
84485	5/1/2015 13:18	B	3935866.47	813764.0299	-73.022	-41.945	2027	1.0716685	-0.86156598	-0.413134498
84485	5/1/2015 13:43	B	14295204.5	120540.5	-73.02	-41.904	1514	0.136531226	-0.975091167	-0.305941541
84485	5/1/2015 14:27	2	682307.6606	8114.839421	-73.101	-41.866	2599	-0.7365922	-1.080848486	-0.434560296
84485	5/1/2015 21:17	B	56828460.5	3737378	-73.294	-41.779	24654	-1.256345956	-0.478207397	-0.38153643

84485	5/1/2015 22:22	B	36412230.21	705902.7942	-73.205	-41.773	3877	-1.099665066	-0.549597418	-0.392597683
84485	5/3/2015 10:03	B	32461993.94	33133698.56	-73.034	-42.039	128454	0.949931348	-0.592240683	-0.641805875
84485	5/3/2015 10:37	B	17369816692	4132054611	-72.952	-41.992	2042	0.1260749	-0.777584553	-0.856633052
84485	5/3/2015 12:07	B	20772085.27	2461610.734	-72.932	-42.062	5395	1.724612352	-0.936451335	-0.431997809
84485	5/3/2015 12:15	B	4371605.813	484564.6868	-72.945	-42.047	472	1.905321398	-0.898565388	-0.568555884
84485	5/3/2015 13:47	B	7970511.771	340910.7288	-72.902	-42.046	5538	1.584158312	-1.219920774	-0.508421491
84485	5/3/2015 20:50	B	25365361.41	911051.0889	-72.919	-41.956	25404	-0.558979871	-1.179962775	-0.91220304
84485	5/3/2015 21:52	A	22324625.56	856446.9417	-73.026	-41.92	3703	-0.200934232	-0.929175577	-0.887441741
84485	5/3/2015 21:55	B	1346618.312	729871.688	-73.017	-41.917	183	-0.262176251	-0.955924463	-0.897703759
84485	5/3/2015 22:23	B	1393819.467	1598993.033	-73.018	-41.904	1681	-0.416956475	-0.978645047	-0.938135298
84485	5/3/2015 23:34	A	20730.75366	72977.74634	-73.027	-41.925	4256	-0.156106622	-0.905862776	-0.863955484
84485	5/5/2015 9:09	0	3945689078	178307534.5	-72.903	-41.952	120886	2.765561356	-1.343129433	-2.902260911
84485	5/5/2015 9:16	1	3254554.851	91471.64854	-72.874	-41.957	421	2.507035339	-1.400083202	-2.916503842
84485	5/5/2015 10:53	B	36661259.31	7597645.691	-72.907	-41.951	5835	3.098620668	-1.257033846	-2.767817433
112696	4/9/2015 21:24	2	948763.2855	193373.7145	-73.31	-42.323	4230	0.675673873	-1.21991334	0.488500266
112696	4/9/2015 22:16	B	14158252.25	2379789.747	-73.302	-42.325	3105	0.712362921	-1.3017674	0.497632906

112696	4/9/2015 22:50	B	8991239.701	633530.7986	-73.276	-42.319	2070	0.76718982	-1.259611881	0.534494005
112696	4/15/2015 8:01	B	65258219609	1289015862	-73.271	-41.847	465020	0.278397602	-0.914689244	0.478500442
112696	4/15/2015 8:27	B	10789830.76	34430611.24	-73.269	-41.846	1552	0.288691154	-0.899878565	0.481861877
112696	4/15/2015 9:11	A	201549236.1	1554326.425	-73.251	-41.864	2635	0.196217162	-1.05235225	0.448463074
112696	4/15/2015 9:40	A	24459.80937	15872.69063	-73.259	-41.864	1770	0.196217162	-1.064500071	0.448463074
112696	4/15/2015 10:03	A	2448168.301	363402.1992	-73.25	-41.865	1356	0.162205734	-1.108463469	0.440616576
112696	4/15/2015 10:54	2	136225.4981	41107.00189	-73.252	-41.871	3061	0.140417012	-1.179631088	0.429291199
112696	4/15/2015 11:43	B	11436895.2	2537028.8	-73.24	-41.871	2965	0.1039428	-1.258239192	0.424076513
112696	4/15/2015 13:20	A	556187.4515	203933.5485	-73.245	-41.897	5841	-0.032234076	-1.526790766	0.378561176
112696	4/15/2015 14:18	B	1134249.588	320594.9122	-73.232	-41.904	3451	-0.111710827	-1.817333047	0.364098044
112696	4/15/2015 14:59	B	29504252	2449780.503	-73.197	-41.95	2458	-0.46667726	-1.738106048	0.299933211
112696	4/15/2015 20:16	B	120569311.8	607756070.7	-73.283	-42.035	19045	-0.42995602	-1.742741185	0.222993405
112696	4/15/2015 20:46	0	22144377.55	224583.4516	-73.288	-42.017	1777	-0.425192672	-1.80058914	0.217803228
112696	4/15/2015 21:29	B	42707246.67	9119505.833	-73.279	-42.036	2598	-0.470806698	-2.073891757	0.197140535
112696	4/15/2015 22:59	B	17186578256	20973992.56	-73.256	-42.128	5404	-0.361449735	-2.276599927	0.123728258
112696	4/15/2015 23:08	B	93308172.42	1038867.579	-73.267	-42.136	510	-0.334229351	-2.20548 87	0.119460623

112696	4/16/2015 11:22	B	16829658.17	4081719.831	-73.031	-42.519	44028	-0.442511247	-0.646535197	-0.513971302
112696	4/16/2015 12:51	B	21151213.87	484540.6264	-73.065	-42.477	5391	-0.321825641	-0.540075694	-0.469388868
112696	4/16/2015 13:01	B	1699215.509	6507220.991	-73.092	-42.492	550	-0.30597153	-0.501355222	-0.485312689
112696	4/16/2015 20:49	B	8393133.216	2777775.784	-73.031	-42.859	28124	0.478500663	-0.665340306	-0.888402764
112696	4/16/2015 21:46	B	9898028.007	644868.9933	-73.035	-42.888	3413	0.812066944	-0.605720761	-0.963931802
112696	4/16/2015 22:43	B	528855526	223066228	-73.032	-42.91	3421	0.914463092	-0.511406094	-1.054793026
112696	4/17/2015 10:41	B	137527445.5	25067363.52	-73.211	-43.456	43069	-0.4510023	-0.051565519	-0.490201812
112696	4/17/2015 12:38	B	219402979	7383435.511	-73.13	-43.42	6997	-0.526752883	-0.155835812	-0.469427967
112696	4/17/2015 13:36	B	19419844358	1596760644	-73.064	-43.467	3496	-0.50163109	-0.08541964	-0.500978165
112696	4/17/2015 14:22	B	306985278.5	12370140.04	-73.198	-43.478	2734	-0.49998174	-0.019307802	-0.52376995
112696	4/17/2015 20:24	A	316803.3998	15503065.6	-73.095	-43.794	21720	-0.915144927	0.426054117	-0.601969759
112696	4/17/2015 21:32	1	667964.1597	41016.34034	-73.152	-43.838	4132	-0.882570444	0.378868861	-0.598873414
112696	4/17/2015 23:17	B	26129213.47	680652297	-73.01	-43.893	6298	-0.939827112	0.404007402	-0.584998096
112696	4/18/2015 7:55	B	474829391.5	33871999.01	-73.046	-43.86	31041	-0.704062078	0.392596238	-1.091037536
112696	4/18/2015 9:05	B	172933570.3	11640824.16	-73.033	-43.848	4216	-0.699272366	0.404246369	-1.085410062
112696	4/18/2015 12:19	B	784021968.5	13770829.96	-73.181	-43.612	11628	-0.607349895	0.206277665	-0.906364532

112696	4/18/2015 13:13	B	1015795597	6506323.761	-73.133	-43.575	3240	-0.597857868	0.148982551	-0.87556813
112696	4/18/2015 13:59	B	1198062709	10601055.88	-73.133	-43.558	2784	-0.592287939	0.131730121	-0.859378415
112696	4/18/2015 21:22	B	23949944.06	2540358.443	-73.31	-43.661	26573	-0.478273144	0.371705031	-0.855573925
112696	4/19/2015 8:53	B	1.30087E+11	21304062865	-73.564	-43.747	41473	-0.576641792	-0.211393625	-1.535277517
112696	4/19/2015 11:13	B	27013462812	1565472511	-73.636	-44.001	8412	-0.38826802	-0.348003333	-1.589326117
112696	4/19/2015 21:10	B	20083213609	2788791937	-73.754	-45.087	35787	-0.904066192	-0.342479699	-1.407495747
112696	4/19/2015 21:24	B	960983042	134401427	-73.752	-45.086	841	-0.891277481	-0.320194883	-1.402564437
112696	4/20/2015 22:41	B	6190727559	1346153012	-75.427	-43.512	91014	0.491935333	-2.134899185	0.853396429
112696	4/20/2015 23:48	B	15654894.91	3017605.591	-75.433	-43.505	4019	-0.045088058	-1.601617996	1.016751444
112696	4/21/2015 13:47	B	613232325.8	138538954.2	-75.507	-43.417	50321	4.222152431	-0.679990425	1.252494275
112696	4/21/2015 22:28	B	18181163988	456466034.8	-75.641	-43.047	31284	-0.221229437	0.073915706	2.109842668
112696	4/22/2015 14:17	B	556403608.6	17352543.88	-75.746	-43.371	56928	1.618972441	-0.550833267	1.120032655
112696	4/23/2015 13:52	B	695158692.3	125678440.7	-75.975	-43.213	84906	-0.719491558	0.139450708	2.186370327
112696	4/26/2015 23:34	Z	131474644.7	4418060.338	-76.789	-43.403	294120	3.720746536	0.734943104	1.628059124
84481	4/4/2016 22:31	B	4647036.583	28446307.92	-73	-41.766	1398	1.355469792	-1.541021078	0.520679003
84481	4/4/2016 22:43	A	993019.4048	31281.59517	-73.002	-41.787	721	1.238813647	-1.531962099	0.779807005

84481	4/5/2016 0:17	B	23508424.94	38510847.56	-72.972	-41.801	5614	1.565022465	-1.885604202	0.58915983
84481	4/5/2016 0:23	B	7136871.217	10862051.28	-72.963	-41.79	385	1.253390703	-1.9394617	0.723385809
84481	4/5/2016 0:53	B	2511866.398	3041578.602	-72.961	-41.792	1808	1.323495753	-1.99907476	0.691024999
84481	4/5/2016 1:39	B	2109787.689	55636408.31	-72.96	-41.796	2757	1.516814952	-2.023293064	0.644756628
84481	4/5/2016 2:34	A	753151.3468	6305.153184	-72.97	-41.776	3254	0.948205136	-1.791644416	0.849900098
84481	4/5/2016 3:26	B	1067260.5	223780.5	-72.97	-41.773	3161	0.87012718	-1.74043 59	0.877744937
84481	4/5/2016 4:08	B	1498309.525	466828.975	-72.97	-41.772	2531	0.750115529	-1.729910224	0.897613942
84481	4/5/2016 7:02	B	4256717.555	1338540.945	-72.973	-41.763	10423	0.641747925	-1.630779019	0.964077909
84481	4/5/2016 8:36	B	6031664.186	1451204.814	-72.975	-41.765	5618	0.702898584	-1.603958984	0.93869184
84481	4/5/2016 10:04	A	8458855.187	128394.8131	-72.969	-41.786	5305	1.191653307	-1.864415497	0.766481909
84481	4/5/2016 11:53	B	2555645781	506651711.2	-72.926	-41.764	6551	0.078108232	-1.756212724	0.898711753
84481	4/5/2016 13:11	B	69961524.16	13167170.34	-72.924	-41.76	4686	0.028029292	-1.748561537	0.922800461
84481	4/5/2016 14:47	B	6710520.345	385615.6548	-72.918	-41.763	5747	0.018980637	-1.790135384	0.894463161
84481	4/5/2016 19:18	B	2122194716	312709763.7	-72.887	-41.78	16216	0.527376881	-2.275954189	0.758170896
84481	4/5/2016 20:54	A	128684.7224	2591.777595	-72.846	-41.805	5782	0.497403559	-2.905676682	0.586801009
84481	4/5/2016 21:29	B	661808.5855	177009.9145	-72.839	-41.808	2093	0.527166421	-2.954869676	0.610801541

84481	4/5/2016 22:15	B	102817800	1740.5	-72.834	-41.817	2797	0.828216792	-2.986995713	0.637936558
84481	4/5/2016 23:17	B	123936.2502	41200918.25	-72.811	-41.817	3719	1.04819739	-2.986995713	0.82743986
84481	4/6/2016 3:46	B	3391745.658	2838444.842	-72.777	-41.834	16108	0.426441447	-2.657686241	1.481375198
84481	4/6/2016 7:59	B	18958735.1	1801085.895	-72.761	-41.799	15206	0.460691955	-2.91977326	1.550750413
84481	4/6/2016 8:12	B	15126288.38	4566921.621	-72.739	-41.824	767	0.176404573	-2.579281984	1.602735558
84481	4/6/2016 20:43	B	28859339.52	7471792.982	-72.659	-41.823	45043	0	-1.329016236	0
84481	4/6/2016 21:04	B	172935055.9	26831505.05	-72.662	-41.785	1263	0	-1.442479958	2.086230261
84481	4/6/2016 22:38	B	97877166.32	17679290.18	-72.657	-41.785	5643	0	-1.398778539	0
84481	4/7/2016 1:53	B	67303202	961884.5	-72.632	-41.796	11731	0	-1.179687228	0
84481	4/7/2016 10:57	B	26957572267	74279777.31	-72.809	-42.101	32630	-0.558287597	-2.986995713	2.071083662
84481	4/7/2016 14:53	B	569091846.1	13148948.41	-72.849	-42.095	14135	-0.401650682	-2.986995713	1.923986074
84481	4/7/2016 18:54	B	508060736	34717810.46	-72.826	-42.093	14443	-0.444452116	-2.986995713	1.994113751
84481	4/7/2016 20:31	A	5077216.716	365096.2839	-72.898	-42.062	5876	1.089110811	-2.986995713	1.551610302
84481	4/7/2016 22:14	B	13102300.45	255368.5504	-72.907	-42.02	6129	2.736358053	-2.986995713	1.068697572
84481	4/7/2016 23:11	B	6962786120	314582916.9	-72.884	-42.054	3460	1.560832683	-2.986995713	1.446271074
84481	4/7/2016 23:43	B	6091849.21	542491.2897	-72.922	-42.01	1918	3.140544824	-2.986995713	0.767235091

84481	4/8/2016 0:47	B	11256190.21	3881730.792	-72.921	-42.006	3815	-0.284032285	-2.986995713	-0.038601125
84481	4/8/2016 1:17	B	3837040.816	1396623.684	-72.924	-42.01	1830	-0.233452227	-2.986995713	-0.026762937
84481	4/8/2016 1:31	B	4241521.485	1071179.015	-72.929	-42.027	821	-0.099889378	-2.986995713	0.024542088
84481	4/8/2016 3:14	B	5250668.192	1471798.308	-72.915	-42.035	6162	-0.107800842	-2.986995713	0.09616846
84481	4/8/2016 12:57	B	10640673.88	5591728.622	-72.93	-41.974	35026	-0.449672086	-2.986995713	-0.14959307
84481	4/8/2016 13:42	B	33458720.41	1376532.086	-72.958	-42.01	2693	-0.234973042	-2.986995713	-0.152603092
84481	4/8/2016 14:32	B	63092030.79	4630354.213	-72.941	-42.017	2991	-0.113017447	-2.986995713	-0.06682091
84481	4/8/2016 20:26	B	115446338.1	2006514.852	-72.917	-42.037	21211	-0.107800842	-2.986995713	0.09616846
84481	4/8/2016 21:38	B	10184186.94	62351981.06	-72.828	-42.025	4352	-0.426280745	-2.737611687	0.17817751
84481	4/9/2016 1:07	B	4322387.065	2446205.935	-72.763	-42.003	12501	0.886826991	-1.625505588	-0.101408575
84481	4/9/2016 4:25	B	13192336.82	3797228.179	-72.708	-41.991	11878	0	-1.330150912	0.048014755
84481	4/9/2016 10:07	B	44083378.46	5714036.04	-72.605	-41.999	20542	0	-0.941793799	0.476964415
84481	4/9/2016 11:02	B	13557732.55	7696387.45	-72.702	-41.968	3318	0	-1.055742999	0
84481	4/9/2016 12:28	B	516433663.8	15860573.24	-72.902	-42.069	5162	-0.619182182	-2.986995713	-0.008220269
84481	4/9/2016 14:11	B	239424764.3	59764288.17	-72.809	-42.049	6134	0.062104178	-2.746211136	0.282156016
84481	4/9/2016 18:27	B	30526356.08	207123008.9	-72.867	-42.035	15394	-0.295006564	-2.986995713	0.067056488

84481	4/9/2016 20:13	B	438858469.1	158443639.9	-72.892	-42.034	6336	-0.456410698	-2.986995713	-0.010423361
84481	4/9/2016 21:05	B	10193549.71	2798515.294	-72.869	-42.008	3129	-0.05591195	-2.92513543	-0.048137287
84481	4/10/2016 0:03	B	7292720.767	1028055.733	-72.861	-41.974	10708	0.857170222	-2.653419907	-0.264428235
84481	4/10/2016 4:10	B	15309149.33	1716121.173	-72.919	-41.887	14773	1.617516897	-2.854289912	-1.10043602
84481	4/10/2016 7:39	A	183367.255	48893.74499	-72.772	-41.775	12587	-0.424707325	-2.986995713	-0.936860331
84481	4/10/2016 8:04	B	447895.1419	136049.8581	-72.772	-41.775	1462	-0.424707325	-2.986995713	-0.936860331
84481	4/10/2016 9:06	B	1332248.363	464934.137	-72.765	-41.75	3766	-0.342617479	-2.928332235	-0.898145282
84481	4/10/2016 9:40	B	129464914.3	39781252.18	-72.768	-41.742	2022	-0.262254605	-2.950987179	-0.893477619
84481	4/10/2016 10:49	B	182025093.8	19217512.7	-72.798	-41.747	4122	-0.367896574	-2.963306812	-0.916475773
84481	4/10/2016 10:59	B	5525993.958	429094.5419	-72.782	-41.75	624	-0.441445231	-2.986995713	-0.925157943
84481	4/10/2016 12:15	B	3656362.331	449031.6688	-72.778	-41.735	4558	-0.032624908	-2.979722318	-0.864450082
84481	4/10/2016 13:51	B	10908197	251445.4991	-72.775	-41.73	5738	0.056107657	-2.945529929	-0.842450594
84481	4/10/2016 14:39	B	5071885.126	1518371.374	-72.779	-41.741	2860	-0.285763034	-2.986995713	-0.905490736
84481	4/10/2016 15:27	B	5021441.015	3680422.985	-72.772	-41.721	2911	0.263358058	-2.928021298	-0.802415254
84481	4/10/2016 23:03	B	19596150.81	1827493.685	-72.743	-41.643	27378	0.875294728	-1.777308313	-0.314690563
84481	4/10/2016 23:52	A	5180006.688	5929610.312	-72.89	-41.597	2894	-0.142957186	-2.629055923	0.00692496

84481	4/11/2016 1:15	B	49278453.81	13284946.19	-72.955	-41.555	4985	-0.036408208	-1.331569328	-0.133610201
84481	4/11/2016 3:04	B	53975497.94	16031870.06	-73.043	-41.496	6558	0	-0.658571313	-0.239750543
84481	4/11/2016 9:09	B	224714448.4	36434041.58	-72.87	-41.624	21932	-0.399769445	-2.755303996	0.075537792
84481	4/11/2016 10:24	B	2885299313	179367837	-72.847	-41.664	4493	0.000474401	-1.978522161	-0.086501696
84481	4/11/2016 10:34	B	104236667.8	4763340.74	-72.844	-41.663	583	-0.159722625	-1.957160177	-0.0759966
84481	4/11/2016 10:53	B	134025488.4	5784764.139	-72.832	-41.661	1132	-0.451555315	-1.893290286	-0.062873408
84481	4/11/2016 12:08	B	32098238.37	1051794.126	-72.801	-41.647	4494	-0.600792878	-1.877638156	-0.114142718
84481	4/11/2016 12:39	B	9561564.5	227138	-72.805	-41.622	1866	-0.489252405	-2.178878628	-0.152873691
84481	4/11/2016 13:27	A	326853.875	110072.625	-72.85	-41.591	2856	-0.401396658	-2.448458618	-0.0048352
84481	4/11/2016 13:44	B	3948778.682	1480929.818	-72.846	-41.603	1028	-0.379570777	-2.69668695	-0.00953817
84481	4/11/2016 14:20	B	370883475	452788.9907	-72.852	-41.592	2163	-0.408926952	-2.504574685	0.003982567
84481	4/11/2016 18:14	B	6720472.168	886715.8317	-72.88	-41.54	14054	-0.761704602	-1.307775229	-0.055492492
84481	4/11/2016 19:51	A	1.70207E+11	2566068311	-72.677	-41.587	5820	-0.78263195	-1.582356322	-0.266433203
84481	4/11/2016 21:26	B	601894213.4	23561675.06	-72.659	-41.587	5673	0	-0.95671585	0
84481	4/11/2016 21:35	B	263215298.9	13758423.6	-72.669	-41.586	583	0	-0.960612283	0
84481	4/11/2016 21:39	B	203946679.3	7949410.685	-72.665	-41.586	247	0	-0.955733541	0

84481	4/11/2016 22:50	A	2614251245	162521061.8	-72.857	-41.611	4244	-0.38873948	-2.83163687	-0.011520259
84481	4/11/2016 23:07	A	756945881.2	28605727.77	-72.865	-41.613	1037	-0.455229719	-2.928081454	0.056167045
84481	4/12/2016 0:32	B	2757614.984	433289.5156	-72.899	-41.618	5053	-0.973344163	-2.919398542	-0.084997423
84481	4/12/2016 1:52	B	3987120.938	817319.5622	-72.94	-41.622	4843	-0.697314325	-2.651150513	-0.120893599
84481	4/12/2016 7:22	B	15631264.66	2749521.34	-73.076	-41.643	19764	-0.598446211	-0.93555169	-0.517769142
84481	4/12/2016 8:51	B	7507085.783	2344719.217	-72.914	-41.612	5375	-0.887274951	-2.910452321	-0.093193858
84481	4/12/2016 10:26	B	10078376.98	2571808.02	-72.917	-41.607	5683	-0.860395352	-2.864758501	-0.098687154
84481	4/12/2016 11:29	B	11919922.21	2967350.786	-72.923	-41.609	3788	-0.851132476	-2.767754989	-0.100481287
84481	4/12/2016 13:07	B	13418847.56	1205617.443	-72.927	-41.611	5873	-0.830888112	-2.759000074	-0.101732598
84481	4/12/2016 14:54	B	13180618.02	1714744.479	-72.928	-41.609	6422	-0.826966927	-2.71752133	-0.104398834
84481	4/12/2016 19:39	A	55965.90455	2738.595449	-72.951	-41.546	17079	-0.456692106	-1.268631272	-0.320357639
84481	4/12/2016 21:03	B	1529149.641	377931.3593	-72.958	-41.54	5070	-0.485079412	-1.153452653	-0.355617348
84481	4/12/2016 21:11	B	2405485.819	238492.681	-72.955	-41.545	471	-0.463682988	-1.23181121	-0.331229506
84481	4/12/2016 21:22	B	3383627.57	4794858.93	-72.932	-41.55	679	-0.459912584	-1.374161153	-0.29068815
84481	4/12/2016 22:37	B	1821200.281	1180353.719	-72.939	-41.546	4487	-0.458068744	-1.309670257	-0.308924338
84481	4/12/2016 22:39	B	1794238.886	1158594.114	-72.936	-41.549	117	-0.458418022	-1.331494622	-0.297401742

84481	4/13/2016 0:30	B	3512786.341	1523058.159	-72.943	-41.537	6625	-1.040101138	-1.189739938	0.079770029
84481	4/13/2016 1:27	B	5607927.715	1179757.285	-72.946	-41.531	3445	-1.04089464	-1.100666816	0.070245558
84481	4/13/2016 2:18	B	7836063.823	713181.1767	-72.951	-41.533	3063	-1.039957305	-1.150392328	0.06639212
84481	4/13/2016 3:04	B	8145270.623	777481.8769	-72.954	-41.531	2791	-1.040369834	-1.094173261	0.061234478
84481	4/13/2016 10:06	B	27681162.46	775830.5392	-72.95	-41.581	25313	-1.041692357	-1.781907929	0.098607213
84481	4/13/2016 11:41	B	40066179.92	2492170.578	-72.99	-41.588	5689	-1.029256714	-1.467100736	0.050897241
84481	4/13/2016 12:50	B	32477586.43	5943816.065	-73.002	-41.587	4148	-1.028662273	-1.366930662	0.037777182
84481	4/13/2016 14:27	B	1892587784	2176427519	-72.925	-41.586	5786	-1.052561754	-2.25510313	0.125669509
84481	4/13/2016 19:27	B	16422048.44	1368736.564	-72.857	-41.633	18032	-1.047650132	-2.522556767	0.236397668
84481	4/13/2016 21:12	B	29947646.98	1468959.519	-72.864	-41.635	6267	-1.050496508	-2.561292682	0.230762474
84481	4/13/2016 22:17	B	4528792014	897736796.2	-72.832	-41.643	3926	-1.020397027	-2.182074472	0.270259018
84481	4/14/2016 9:40	B	162295141.5	7677470.488	-72.706	-41.727	40961	-1.250374318	-2.346252054	-0.190319928
84481	4/14/2016 11:19	B	676783600.4	56291504.06	-72.844	-41.693	5964	-1.25127928	-1.95151694	-0.245906512
84481	4/14/2016 12:30	B	140646493.3	12476681.15	-72.771	-41.719	4241	-1.253839286	-2.890867067	-0.21551141
84481	4/22/2016 1:41	A	226811.0501	2610313.95	-72.933	-43.089	652271	-1.218509185	-1.697955329	-3.149705931
84481	4/22/2016 5:04	B	131917524.5	28758528	-72.933	-43.096	12147	-1.217309279	-1.698077998	-3.147029777

84481	4/22/2016 11:21	B	11181309.99	84099003.01	-73.036	-43.409	22629	0	-1.917276638	0
84481	4/22/2016 14:46	B	286144640	21280962.52	-72.738	-43.618	12294	0	-0.304610321	0
84481	4/23/2016 9:14	B	232569906.9	13416625.65	-74.658	-43.859	66510	0.146350617	-1.096884939	-2.511298925
84481	4/23/2016 10:00	B	261013460.3	17296504.7	-74.768	-43.876	2726	-0.712369188	-0.919050955	-2.816160231
84481	4/23/2016 10:51	B	246129379.1	15347683.38	-74.806	-43.939	3073	-0.33846659	-1.096293269	-2.704718498
84481	4/23/2016 11:34	B	460288901.9	9401271.08	-74.762	-43.91	2571	0.189350515	-1.034840201	-2.676054264
84481	4/23/2016 12:25	B	394223239.5	10221919.03	-74.824	-43.926	3099	-0.734142539	-0.971683337	-2.772606968
84481	4/23/2016 19:16	B	3995873461	19234669.1	-74.997	-43.828	24622	3.590400378	-0.447953504	-2.395640648
84481	4/23/2016 22:38	B	2469606104	24182298.35	-75.234	-43.845	12134	0.27445341	-0.112127227	0.519519038
84481	4/23/2016 23:13	B	2650208757	23731619.24	-75.277	-43.845	2128	0.252931798	-0.052118926	0.690151625
84481	4/24/2016 6:39	B	1312188900	71719694.21	-75.929	-43.832	26758	-0.052559539	0.525098324	0.871580201
84481	4/24/2016 14:50	B	44224388.21	7944284.793	-75.581	-43.804	29458	4.499795162	0.249780084	-0.275877959
84481	4/24/2016 20:50	B	105476416.4	28793300.09	-75.171	-43.743	21607	1.375702176	-0.371023894	-0.193994562
84481	4/24/2016 23:46	Z	1292500051	335605435.2	-75.538	-42.542	10564	0.647544907	0.55016167	1.093013791
84494	4/6/2016 0:05	B	2775502.922	265379.0784	-73.14	-41.753	3114	0.676624441	-0.775260138	1.926458526
84494	4/6/2016 2:11	B	5610290.864	28265271.64	-73.138	-41.756	7572	0.692998957	-0.779629934	1.938179442

84494	4/7/2016 13:12	A	171074.9126	210490.0874	-72.899	-41.763	126047	1.056602213	-1.839710261	2.039977166
84494	4/7/2016 23:11	0	54987668260	2484371278	-72.645	-41.97	35915	0	-1.063134485	0
84494	4/7/2016 23:42	B	1357062292	84480147.71	-72.688	-41.95	1916	0	-1.005979027	0
84494	4/8/2016 1:29	B	7554635.192	2232494.808	-73.031	-41.958	6366	0.314633192	-2.601889641	1.420825186
84494	4/8/2016 6:23	B	63976346.72	4191794.278	-73.081	-41.955	17674	0.275118418	-2.265346646	1.286658885
84494	4/8/2016 8:06	B	80506226.55	7699038.446	-73.041	-41.972	6172	-0.461236562	-2.675160535	1.372125852
84494	4/8/2016 8:49	B	32880776.25	5744900.755	-73.069	-41.98	2583	-0.49075045	-2.725746801	1.363058848
84494	4/8/2016 9:24	B	5808888.149	1495776.851	-73.072	-41.984	2108	-0.523821231	-2.866382531	1.370571903
84494	4/8/2016 10:20	B	335223264.1	590329023.9	-72.977	-42.061	3332	1.158558244	-2.987227125	1.677091825
84494	4/8/2016 11:05	B	5267952.998	4132672.002	-72.999	-42.13	2720	0.674979738	-2.987227125	2.009257063
84494	4/8/2016 12:02	B	15809951.53	2716474.468	-72.997	-42.188	3430	-0.426581812	-2.987227125	2.090942342
84494	4/8/2016 12:53	B	198774627.2	228579648.8	-73.095	-42.144	3066	-0.096955116	-2.987227125	1.853068344
84494	4/8/2016 14:32	B	238879575.3	87007127.24	-73.309	-42.093	5931	-0.072370242	-2.987227125	1.789167663
84494	4/8/2016 18:38	B	3481945134	11331430872	-73.59	-42.144	14775	0.297240689	-0.689310314	0.851076701
84494	4/8/2016 20:24	1	2148497.524	358914.9765	-73.489	-41.981	6328	1.069684264	-1.200651842	1.26533362
84494	4/8/2016 22:02	B	147968442.6	6736306.389	-73.547	-41.938	5873	0.712784853	-0.828856807	0.899867455

84494	4/8/2016 23:19	0	6690205.766	3080760.734	-73.474	-41.977	4654	1.040808068	-1.296107364	1.349505872
84494	4/8/2016 23:27	2	8041083.161	501501.839	-73.474	-41.979	442	1.029304514	-1.293474525	1.353463167
84494	4/9/2016 0:28	B	6003112.5	1593112.5	-73.489	-41.975	3671	1.09728832	-1.189524763	1.268618407
84494	4/9/2016 4:33	B	23612192	4663458	-73.49	-41.99	14686	0.740751068	-1.234166983	1.086109014
84494	4/9/2016 6:16	B	244474113.1	228020046.9	-73.286	-41.97	6220	0.260893212	-2.987227125	1.342934641
84494	4/9/2016 8:25	B	4391393.885	12041458.62	-73.214	-41.972	7726	0.303581063	-2.987227125	1.325698064
84494	4/9/2016 10:59	A	14930.08001	57300.41999	-73.228	-41.993	9263	0.052894726	-2.987227125	1.414356088
84494	4/9/2016 11:27	B	6757730.569	362921.9308	-73.233	-41.982	1671	0.133285734	-2.987227125	1.377987268
84494	4/9/2016 11:48	B	1780929.136	146203.8643	-73.23	-41.981	1266	0.133285734	-2.987227125	1.377987268
84494	4/9/2016 12:36	B	1973739.307	338268.6932	-73.212	-41.983	2873	0.170283069	-2.987227125	1.361583134
84494	4/9/2016 13:05	B	18793976.75	1020655.245	-73.202	-41.996	1713	0.125222971	-2.987227125	1.393246816
84494	4/9/2016 14:06	A	10127388.37	6650461.625	-73.255	-42.023	3677	0.502028459	-2.987227125	1.537680954
84494	4/9/2016 15:51	B	15357454.66	3909918.341	-73.256	-42.033	6288	0.489410782	-2.987227125	1.585390749
84494	4/9/2016 18:30	B	17974330.52	3252489.482	-73.236	-42.022	9528	0.470327608	-2.987227125	1.529788593
84494	4/9/2016 20:11	A	63151992.62	5904217.383	-73.236	-41.916	6089	-0.060767577	-2.987227125	1.126662194
84494	4/9/2016 22:17	B	12208158.15	2126054.351	-73.246	-41.903	7541	-0.41603314	-2.987227125	1.101662499

84494	4/9/2016 22:46	A	129774.8716	24534.12844	-73.211	-41.973	1766	0.29523112	-2.987227125	1.327822776
84494	4/9/2016 23:19	B	717421.6539	125680.8461	-73.208	-41.97	1940	0.327689232	-2.987227125	1.314716223
84494	4/10/2016 0:00	B	1357952	368082	-73.209	-41.968	2499	0.334450649	-2.987227125	1.314337602
84494	4/10/2016 0:28	B	7632924.161	537372.8392	-73.209	-41.954	1640	0.635442012	-2.987227125	1.244012297
84494	4/10/2016 0:53	A	24195900.38	2882666.119	-73.279	-41.948	1542	0.262876721	-2.987227125	1.253083071
84494	4/10/2016 2:27	A	2332757.689	248927.3113	-73.225	-41.951	5597	0.534662124	-2.987227125	1.250624133
84494	4/10/2016 3:20	B	1087483.004	210451.4961	-73.207	-41.959	3195	0.049722196	-2.987227125	1.129967414
84494	4/10/2016 4:11	B	4959121.233	1029928.767	-73.192	-41.963	3082	0.028648695	-2.987227125	1.121864045
84494	4/10/2016 6:01	A	6894.313589	266635.6864	-73.173	-41.968	6590	0.060614607	-2.987227125	1.132435243
84494	4/10/2016 7:40	1	342287.6132	4567136.887	-73.218	-41.967	5950	-0.043260357	-2.987227125	1.161978292
84494	4/10/2016 7:59	B	3939868.709	1785696.291	-73.217	-41.966	1098	-0.005093595	-2.987227125	1.157474816
84494	4/10/2016 9:08	B	12184656.89	4547440.111	-73.211	-41.97	4169	-0.057971041	-2.987227125	1.172009037
84494	4/10/2016 9:37	B	46676781.73	1456179.268	-73.207	-41.94	1767	0.045076122	-2.987227125	1.066754734
84494	4/10/2016 10:46	B	391737642.4	51896100.11	-73.195	-41.975	4103	-0.055386251	-2.987227125	1.165244499
84494	4/10/2016 11:00	B	49216603.85	544296.6482	-73.193	-41.977	841	-0.116364602	-2.987227125	1.175959681
84494	4/10/2016 11:16	B	1891098.868	69165.13231	-73.191	-41.978	942	-0.127027436	-2.987227125	1.176935528

84494	4/10/2016 12:09	B	2335703.038	285356.9624	-73.185	-41.983	3208	-0.127391924	-2.987227125	1.175775488
84494	4/10/2016 12:33	B	11935480.87	3682263.626	-73.169	-41.994	1451	-0.429584842	-2.987227125	1.196361903
84494	4/10/2016 15:35	B	5033195.299	1416334.701	-73.179	-41.986	10927	-0.275310433	-2.987227125	1.186435829
84494	4/10/2016 18:18	B	9898548.694	1035185.806	-73.172	-41.975	9734	-0.016780781	-2.987227125	1.146287772
84494	4/10/2016 19:56	0	79030583233	3570632280	-73.007	-42.004	5899	-0.619200104	-2.987227125	1.289283461
84494	4/10/2016 20:34	B	168681556.9	6868675.637	-72.986	-42.009	2279	-0.503921462	-2.987227125	1.330663933
84494	4/10/2016 21:49	B	60476215.01	4094133.995	-72.964	-42.026	4498	-0.037303195	-2.987227125	1.342733822
84494	4/10/2016 22:18	1	36737687.07	63401.93325	-72.991	-42.009	1750	-0.488533019	-2.987227125	1.320378988
84494	4/10/2016 23:33	B	27080505.66	25259080.34	-73.101	-42.015	4513	-0.74749816	-2.987227125	1.26536631
84494	4/11/2016 1:11	B	19818086.67	1661726.329	-73.058	-41.981	5895	-0.22607368	-2.79095102	1.29428114
84494	4/11/2016 2:06	B	5782629.195	1650051.805	-73.003	-42.016	3246	-0.370256402	-2.987227125	1.286526687
84494	4/11/2016 2:59	B	7848617.841	756983.1586	-73.093	-41.983	3208	-0.457864154	-2.803583918	1.261341934
84494	4/11/2016 3:43	A	114405.6696	216354.3304	-73.055	-41.9	2667	0.337698681	-1.397052544	1.009112262
84494	4/11/2016 5:44	B	31953314.03	9191952.471	-73.006	-41.827	7258	-0.529849953	-1.485769425	0.852098736
84494	4/11/2016 7:29	B	431961974.9	51261187.63	-72.965	-41.901	6270	2.202367319	-2.237110231	1.047887155
84494	4/11/2016 10:53	B	194754123.6	10689745.39	-72.942	-41.952	12272	1.669193615	-2.987227125	1.590233371

84494	4/11/2016 12:04	B	202660984.7	16370769.84	-72.852	-41.905	4232	0.598993965	-2.338766811	1.133251811
84494	4/11/2016 18:12	B	279368266.8	12596858.18	-73.165	-42.363	22075	-0.832213911	-1.187554165	1.210448769
84494	4/11/2016 21:46	B	11232975174	85982279.04	-73.023	-42.226	12845	0.973633519	-2.987227125	1.815605608
84494	4/11/2016 23:12	0	86957656144	3285588753	-73.074	-42.235	5135	0.989218731	-2.947578423	1.777842686
84494	4/11/2016 23:27	B	7201000664	326370740.6	-73.057	-42.238	916	0.978129141	-2.882202731	1.772762636
84494	4/12/2016 0:31	B	351547905	37568009.47	-73.047	-42.263	3875	1.004949492	-2.362616669	1.685027338
84494	4/12/2016 0:46	2	1423945.11	14317.38987	-73.087	-42.277	868	0.741239492	-1.969791384	1.512335698
84494	4/12/2016 1:42	A	370620.4131	75109.5869	-73.025	-42.267	3391	1.093368679	-2.588068469	1.753514898
84494	4/12/2016 3:23	B	1713557.836	534727.1644	-73.034	-42.309	6052	1.353669595	-2.296637916	1.560297755
84494	4/12/2016 5:06	B	27757453.41	45458775.09	-73.031	-42.311	6192	1.290010849	-2.358408585	1.574598681
84494	4/12/2016 5:40	3	9724.817581	27881.68242	-73.023	-42.283	2039	1.05530348	-2.483316325	1.728288384
84494	4/12/2016 7:13	B	4446595.242	1630417.258	-73.007	-42.283	5576	0.85822098	-2.672226528	1.785630715
84494	4/12/2016 9:03	B	2922821.944	675764.0565	-72.996	-42.285	6571	0.705072304	-2.821265644	1.800399626
84494	4/12/2016 9:55	A	101478.0413	5826908.459	-72.979	-42.265	3113	0.417549348	-2.987227125	1.929326743
84494	4/12/2016 10:14	B	809717.0511	3199749.449	-72.977	-42.262	1158	0.37747858	-2.987227125	1.951560401
84494	4/12/2016 10:36	B	852801.4438	1433303.056	-72.975	-42.262	1302	0.37747858	-2.987227125	1.951560401

84494	4/12/2016 12:08	B	2125971.55	1030609.45	-72.969	-42.271	5508	0.304191134	-2.987227125	1.931389686
84494	4/12/2016 13:08	B	412544103.9	67375576.57	-73.014	-42.34	3648	0.904519317	-2.885692938	1.582438528
84494	4/12/2016 14:04	B	4845849.829	1135743.171	-72.95	-42.3	3321	-0.036014612	-2.987227125	1.886729105
84494	4/12/2016 14:55	B	30821506.93	5721195.565	-72.962	-42.317	3078	0.184553778	-2.987227125	1.803106715
84494	4/12/2016 19:35	B	394725678.5	20790946.48	-72.891	-42.252	16779	0.49795748	-2.987227125	2.078595367
84494	4/12/2016 21:21	B	46390763010	14167062.83	-73.012	-42.092	6381	1.148123171	-2.987227125	1.940278176
84494	4/12/2016 22:50	B	92087544.54	9783827.957	-73.027	-42.046	5322	-0.316982366	-2.987227125	1.652706839
84494	4/13/2016 0:26	B	508216.6012	1889565.899	-72.846	-41.936	5761	0.940701703	-2.107224989	1.832161293
84494	4/13/2016 1:24	B	24132786.18	4839582.818	-72.986	-41.962	3516	0.655701367	-2.987227125	1.552258931
84494	4/13/2016 5:28	1	1102669.813	585371.1868	-72.813	-41.805	14644	-0.289746654	-2.987227125	2.103349983
84494	4/13/2016 7:10	B	7227481574	518922160.9	-72.76	-41.833	6073	-0.244085616	-2.633826373	2.197129421
84494	4/13/2016 8:24	B	75401462.24	8090894.757	-72.767	-41.84	4470	0	-2.474160063	2.206329246
84494	4/13/2016 10:13	A	1.73368E+11	5390298656	-72.742	-41.857	6520	-0.158387192	-2.441281028	2.209510646
84494	4/13/2016 11:02	B	8408802571	1828453478	-72.779	-41.818	2949	-0.274173246	-2.950582776	2.177763918
84494	4/13/2016 11:42	B	24826468.03	2690776.469	-72.783	-41.799	2418	-0.323670864	-2.987227125	2.150959142
84494	4/13/2016 12:46	B	105104230.1	14597492.42	-72.748	-41.782	3849	-0.401049481	-2.77453518	2.172097164

84494	4/13/2016 13:45	B	216245766.3	24433933.69	-72.854	-41.875	3486	-0.02360283	-2.437322644	2.214420074
84494	4/13/2016 14:27	B	3118128248	1327494.565	-72.907	-41.821	2574	-0.298236248	-2.987227125	2.020003149
84494	4/13/2016 19:35	B	370388202.8	196907425.7	-72.852	-41.862	18429	-0.060027386	-2.593538084	2.178821484
84494	4/13/2016 20:42	B	23900293.47	61538799.53	-72.836	-41.827	4033	-0.197418353	-2.840490719	2.117007225
84494	4/13/2016 21:11	B	8766843.657	4054233.343	-72.84	-41.82	1724	-0.229115956	-2.910363249	2.096554127
84494	4/13/2016 22:34	B	2240022.133	917846.3668	-72.848	-41.801	5007	-0.351899025	-2.894321541	2.043588845
84494	4/14/2016 0:03	B	6526621.438	391179.0625	-72.845	-41.825	5365	-0.20637279	-2.90761811	2.100163521
84494	4/14/2016 0:08	B	168163712.7	17755072.27	-72.832	-41.823	296	-0.199712961	-2.883392755	2.116507099
84494	4/14/2016 1:08	B	5230351.012	541969.4881	-72.838	-41.813	3580	-0.253808618	-2.987227125	2.090450613
84494	4/14/2016 4:26	1	175926.9437	569021.5563	-72.845	-41.873	11887	-1.311415082	-2.262032656	1.545146955
84494	4/14/2016 6:54	B	335212540.3	10424336.23	-72.857	-41.86	8888	-1.320427656	-2.619159376	1.542585573
84494	4/14/2016 7:59	B	1269440.472	656022.0277	-72.853	-41.859	3881	-1.319882345	-2.579477761	1.543744613
84494	4/14/2016 8:37	B	9121710.87	374018.1304	-72.863	-41.855	2310	-1.32530253	-2.7670738	1.541325479
84494	4/14/2016 9:42	B	4982859.071	770781.9292	-72.876	-41.854	3874	-1.334442496	-2.904669628	1.538242823
84494	4/14/2016 9:57	B	313593894.4	29314298.08	-72.884	-41.872	892	-1.320009981	-2.934154448	1.535362965
84494	4/14/2016 11:40	B	9535270.926	668187.0743	-72.899	-41.871	6178	-1.325265193	-2.987227125	1.532079325

84494	4/14/2016 12:11	B	7052287.621	402496.8786	-72.9	-41.87	1847	-1.326679953	-2.987227125	1.532295003
84494	4/14/2016 12:31	B	13089200.45	527332.0511	-72.918	-41.879	1252	-1.315325717	-2.839859542	1.527801007
84494	4/14/2016 13:17	B	7153378.123	809872.3771	-72.907	-41.869	2758	-1.332655281	-2.987227125	1.531312672
84494	4/14/2016 14:09	B	9319175.983	246101.0167	-72.909	-41.867	3070	-1.33355778	-2.987227125	1.530596816
84494	4/14/2016 15:53	B	7432930.397	729250.1028	-72.917	-41.863	6279	-1.343359592	-2.801192511	1.530129572
84494	4/14/2016 19:13	B	415294135.2	477577391.3	-72.899	-41.881	11997	-1.314104946	-2.987227125	1.531963894
84494	4/14/2016 20:55	1	1251502.494	1327064.006	-72.801	-41.805	6082	-1.294124838	-2.987227125	1.553505806
84494	4/14/2016 21:47	B	31726852.96	7949905.537	-72.788	-41.796	3168	-1.287132567	-2.987227125	1.554784704
84494	4/14/2016 22:15	B	44745728.38	13688232.12	-72.784	-41.788	1685	-1.284748547	-2.987227125	1.554086652
84494	4/14/2016 23:33	A	136393.457	1671.542983	-72.847	-41.808	4666	-1.315732847	-2.956258597	1.542000692
84494	4/15/2016 0:03	B	563922	149604.5	-72.847	-41.81	1787	-1.316508667	-2.955111614	1.542287627
84494	4/15/2016 2:26	A	48718.1849	1346634.815	-72.867	-41.901	8599	-1.291636953	-2.629973903	1.534504608
84494	4/15/2016 6:37	B	25849902.96	8336699.537	-72.886	-41.931	15070	-0.378004288	-2.987227125	0.893286723
84494	4/15/2016 10:03	A	284583528.3	61931076.72	-72.974	-41.822	12324	-0.628955539	-1.80575873	0.688145375
84494	4/15/2016 11:24	B	56249449.42	561979.0774	-72.974	-41.852	4868	-0.413634258	-1.666962591	0.747010034
84494	4/15/2016 11:42	B	74814079.17	3991519.333	-72.932	-41.875	1069	-0.300983747	-2.585626349	0.781391305

84494	4/15/2016 13:47	B	122314279.8	6524584.68	-72.939	-41.903	7535	-0.211436867	-2.633950331	0.861558758
84494	4/15/2016 15:30	B	20003759.18	4125947.316	-72.914	-41.912	6175	-0.270533062	-2.902790089	0.865137046
84494	4/15/2016 19:04	B	253732089.1	1985899.878	-72.924	-41.933	12793	-0.256074359	-2.932876849	0.93006461
84494	4/15/2016 21:23	B	7109926.037	689950.463	-73.123	-41.979	8378	-0.274082824	-2.802404414	1.337474192
84494	4/15/2016 22:08	B	8773278.25	536894.2497	-73.155	-42.046	2699	0.553535334	-2.987227125	1.210591788
84494	4/15/2016 22:27	B	9601651.213	584663.2866	-73.156	-42.049	1152	0.506647025	-2.987227125	1.201263201
84494	4/15/2016 23:06	B	8098132.765	946117.735	-73.192	-42.077	2333	-0.755270167	-2.987227125	1.07482706
84494	4/16/2016 0:51	B	29128837.22	25354007.78	-73.328	-42.139	6286	-0.411507989	-2.686945478	0.895394059
84494	4/16/2016 2:01	B	197618681.6	171953123.4	-73.349	-42.159	4176	-0.266769388	-2.213859457	0.831410011
84494	4/16/2016 2:55	B	161796657	92274284.01	-73.42	-42.25	3281	-0.455649594	-0.934964675	0.740278444
84494	4/16/2016 6:35	B	21974841052	551302980.6	-73.331	-42.145	13153	-0.985898117	-2.912804946	0.898117734
84494	4/16/2016 8:48	B	284098331.7	8272896.8	-73.237	-42.138	8001	-1.13528765	-2.987227125	0.911890602
84494	4/16/2016 11:15	2	196704.6758	275137.8242	-72.797	-42.135	8822	-0.072214005	-2.987227125	1.627177499
84494	4/16/2016 12:48	B	29613286.25	29613286.25	-72.634	-42.126	5564	-0.708474952	-2.576707159	1.439509142
84494	4/16/2016 13:26	B	30517090.77	2194225.734	-72.581	-42.081	2282	-1.254260785	-1.360263365	1.516645548
84494	4/16/2016 14:21	B	2263066.383	459479.6165	-72.477	-42.065	3317	-1.253945481	-0.805687883	1.523699859

84494	4/16/2016 15:05	B	2576379.476	658999.0238	-72.518	-42.064	2641	-1.275278659	-0.981726746	1.52381177
84494	4/16/2016 20:49	B	103354310.1	1865707.873	-72.74	-41.781	20623	0.007708953	-2.644031382	0.819382651
84494	4/16/2016 22:28	A	348984106.7	2687293.269	-72.805	-41.761	5938	0.241339285	-2.962732051	0.578582379
84494	4/16/2016 22:49	B	47217330.02	11301032.48	-72.825	-41.732	1269	-0.137070265	-2.712982598	0.498572481
84494	4/16/2016 23:37	2	1386830.052	252002.4475	-72.855	-41.721	2882	-0.252955036	-2.072916676	0.476557536
84494	4/17/2016 0:04	0	651385.9886	4780872.511	-72.893	-41.734	1612	-0.192267591	-1.592586349	0.467714254
84494	4/17/2016 0:26	B	12005450.04	4345864.464	-72.887	-41.72	1356	-0.143691503	-1.733686552	0.493852335
84494	4/17/2016 1:14	A	73634.27878	44039.72122	-72.881	-41.677	2876	-0.181306904	-1.921962486	0.612491416
84494	4/17/2016 1:40	B	471795.8535	156974.6465	-72.882	-41.678	1532	-0.174945012	-1.921962486	0.611850689
84494	4/17/2016 3:22	A	3075084.112	19621.88829	-73.022	-41.614	6156	-0.428883291	-1.341292658	1.162995316
84494	4/17/2016 4:38	B	11515200.5	3003700.5	-73.042	-41.566	4546	0.081062807	-0.921998911	1.310601817
84494	4/17/2016 5:04	B	8425492.45	1493374.05	-73.041	-41.568	1551	0.079786899	-0.92260378	1.306275294
84494	4/17/2016 6:22	B	249682633.8	175801791.2	-72.981	-41.584	4678	0.041925504	-1.470187591	1.268841179
84494	4/17/2016 8:03	A	1361433.009	2072490.991	-72.943	-41.579	6047	-0.140606202	-1.920766294	1.364583692
84494	4/17/2016 8:25	A	6801900.462	4800820.538	-72.986	-41.601	1342	-0.013836946	-1.614400784	1.216033704
84494	4/17/2016 9:25	B	9707334.327	11972395.67	-72.962	-41.584	3571	0.096864389	-1.750591838	1.302225891

84494	4/17/2016 10:38	A	80854.21888	4282.281116	-73.022	-41.653	4373	-0.775496152	-1.437639705	1.09914217
84494	4/17/2016 11:06	B	1552548.192	354421.8075	-73.023	-41.653	1692	-0.800002217	-1.437639705	1.096711636
84494	4/17/2016 11:30	B	1195641.37	369571.6301	-73.025	-41.656	1428	-0.8021982	-1.337476568	1.092471594
84494	4/17/2016 11:46	B	1115764.192	347810.3075	-73.025	-41.656	990	-0.805518742	-1.329852732	1.092024936
84494	4/17/2016 12:13	A	337161863.8	255608593.2	-72.94	-41.688	1601	0.258831984	-1.967031014	0.974954205
84494	4/17/2016 13:05	B	197407781.6	56185762.92	-72.933	-41.668	3136	1.16125262	-2.391620161	1.04100314
84494	4/17/2016 18:43	B	40703818597	3804691679	-72.949	-41.639	20279	0.891888486	-2.695721141	1.203746458
84494	4/17/2016 20:20	B	18456957.5	1651062.502	-72.947	-41.633	5840	0.749365585	-2.616851741	1.223957211
84494	4/17/2016 21:40	B	9263827.777	1993354.723	-72.949	-41.63	4798	0.674677249	-2.590982647	1.234028354
84494	4/17/2016 22:02	B	3579626.375	1888254.125	-72.913	-41.642	1284	2.420720641	-2.791465958	1.216892126
84494	4/17/2016 22:17	B	3149923.622	2706368.378	-72.912	-41.64	946	2.42706331	-2.777483018	1.240748872
84494	4/17/2016 23:33	0	2076601.094	792342.906	-72.879	-41.691	4565	0.463447686	-1.791830767	0.830351364
84494	4/18/2016 0:01	B	216246484.7	56368627.78	-72.894	-41.673	1670	1.440985401	-2.074459155	0.911014836
84494	4/18/2016 1:04	B	70456222.44	23097234.56	-72.873	-41.673	3749	1.741866986	-1.969158541	0.899388359
84494	4/18/2016 1:21	B	1800531.133	481593.8667	-72.864	-41.672	1012	1.905070557	-1.959573714	0.921486534
84494	4/18/2016 2:12	B	2664985.629	713424.8711	-72.848	-41.674	3063	1.971945824	-1.92044189	0.972299207

84494	4/18/2016 6:11	B	37453200.27	13576089.73	-73.03	-41.537	14340	0.695484381	-0.850416692	1.445516951
84494	4/18/2016 7:51	B	7952053.383	2027058.617	-73.051	-41.537	6043	0.685556661	-0.778636973	1.448679532
84494	4/18/2016 9:17	B	7705149.597	868903.4033	-73.041	-41.554	5118	0.574611455	-0.883307461	1.382129154
84494	4/18/2016 10:04	B	8098992.78	1491529.72	-73.064	-41.567	2810	0.286929134	-0.800229918	1.320870694
84494	4/18/2016 10:53	B	21543455.61	613316.8914	-73.069	-41.625	2953	-0.464003269	-0.963750341	1.169854983
84494	4/18/2016 11:16	B	10563373.61	25041252.89	-73.021	-41.633	1412	-0.844318291	-1.426126333	1.138213424
84494	4/18/2016 12:44	B	7119473.577	39407222.42	-73.009	-41.664	5271	-0.814865907	-1.649086233	1.132950488
84494	4/18/2016 14:22	B	1327652.906	2518103.594	-73.003	-41.707	5843	-1.045316189	-1.417800428	1.097361065
84494	4/18/2016 18:33	B	8828527.992	7177817.008	-72.958	-41.863	15074	0.639318544	-1.937609917	1.297428003
84494	4/18/2016 21:51	B	8667184.943	5816995.557	-72.939	-41.974	11887	0.235908128	-2.987227125	1.932981181
84494	4/18/2016 23:11	B	4563887.213	48850457.79	-72.958	-41.996	4805	-0.276187553	-2.987227125	2.018562789
84494	4/18/2016 23:37	B	3677927.659	31141965.34	-72.961	-42.032	1589	0.273642021	-2.987227125	1.984626594
84494	4/19/2016 2:42	A	1057012.726	181832.274	-73.217	-42.236	11073	-0.69369172	-2.987227125	1.030568922
84494	4/19/2016 3:34	B	11209916.11	2419927.887	-73.259	-42.274	3112	-1.199071435	-1.969187881	0.68061313
84494	4/19/2016 7:45	B	27908356.2	7608848.795	-73.283	-42.255	15047	-1.219307916	-2.314733088	0.665532934
84494	4/19/2016 8:58	B	54397757.52	26759480.98	-73.197	-42.155	4391	-0.228222966	-2.987227125	0.822066266

84494	4/19/2016 9:12	B	11729571.13	5114457.375	-73.198	-42.154	858	-0.212867729	-2.987227125	0.82624275
84494	4/19/2016 10:42	B	16560618.9	2431666.102	-73.201	-42.19	5404	-0.673352458	-2.987227125	0.743233884
84494	4/19/2016 10:56	A	102211.7495	12400.75046	-73.211	-42.206	843	-0.967724849	-2.987227125	0.717227466
84494	4/19/2016 12:50	B	30492808.89	4067499.114	-73.163	-42.268	6846	-1.048876831	-2.154897331	0.743460713
84494	4/19/2016 18:16	B	8366143.735	1802481.265	-73.105	-42.315	19540	-0.923056308	-1.57880363	0.824632396
84494	4/19/2016 20:01	B	387924342.5	43533776.04	-73.23	-42.203	6270	-0.919273804	-2.929622523	0.712187196
84494	4/19/2016 21:40	B	112470684.5	15158755.99	-73.199	-42.161	5976	-0.237681028	-2.987227125	0.80827222
84494	4/19/2016 22:33	1	85489497.45	3867214.549	-73.246	-42.152	3190	-0.589636116	-2.987227125	0.754165327
84494	4/19/2016 22:59	A	2389298	684.5	-73.234	-42.171	1536	-0.622992579	-2.959418007	0.741159481
84494	4/19/2016 23:05	B	4531838.414	116809.5856	-73.244	-42.162	390	-0.613252049	-2.962025112	0.742758759
84494	4/20/2016 0:46	B	2326324.5	425964.5	-73.254	-42.151	6006	-0.632124579	-2.987227125	0.739631112
84494	4/20/2016 2:20	A	1228831.971	222033.0294	-73.321	-42.129	5698	-0.901850067	-2.835789835	0.675577989
84494	4/20/2016 5:53	B	3250608.799	1146739.201	-73.38	-42.085	12761	0.110934802	-1.884842905	0.510785985
84494	4/20/2016 9:10	B	6631547.998	2433158.002	-73.347	-42.121	11829	-0.086583067	-2.383751235	0.375536455
84494	4/20/2016 10:26	B	48542548.56	13763711.44	-73.382	-42.13	4567	-0.491778747	-1.706798691	0.382087995
84494	4/20/2016 10:39	B	13915539.96	5453872.539	-73.359	-42.107	775	0.148149894	-2.213792172	0.42794442

84494	4/20/2016 12:18	B	12723377.07	1885355.425	-73.338	-42.126	5940	-0.037347249	-2.494839856	0.377047912
84494	4/20/2016 15:24	A	133580379.5	81633335.02	-73.314	-42.169	11112	-0.289491462	-2.611635755	0.396382106
84494	4/20/2016 19:44	B	51528801.62	17117300.88	-73.34	-42.139	15601	-0.349804004	-2.46108601	0.348365079
84494	4/20/2016 20:20	B	68983631.03	6657333.472	-73.336	-42.151	2182	-0.473530581	-2.545728328	0.346008891
84494	4/20/2016 21:28	B	318501194.6	10107897.91	-73.349	-42.101	4072	0.297868999	-2.347727879	0.451358957
84494	4/20/2016 22:02	B	4636780575	1438943.532	-73.421	-42.139	2042	-0.675602775	-1.605750136	0.381349771
84494	4/20/2016 22:49	A	1241635.053	4607.446941	-73.393	-42.126	2803	-0.510252215	-1.583620739	0.407129877
84494	4/21/2016 0:28	B	1934086.699	431978.3008	-73.424	-42.124	5941	-0.725051608	-1.173912279	0.439509324
84494	4/21/2016 1:56	1	3977465.946	640420.5539	-73.316	-42.189	5298	-0.387268327	-2.19727065	0.425209986
84494	4/21/2016 2:53	B	15352562.73	6573635.771	-73.319	-42.197	3426	-0.405885765	-1.988710943	0.440341433
84494	4/21/2016 3:40	A	24896263.88	8315182.625	-73.204	-42.18	2816	0.021350722	-2.987227125	0.654530107
84494	4/21/2016 5:40	B	199411982.9	229313218.1	-73.203	-42.128	7235	0.539370035	-2.987227125	0.778708981
84494	4/21/2016 7:15	B	1058242318	8100902.525	-73.146	-42.109	5652	-0.415655761	-2.987227125	0.990226476
84494	4/21/2016 8:25	A	56132.24617	769380.2538	-73.099	-42.153	4206	-0.642678885	-2.987227125	0.96231061
84494	4/21/2016 8:43	B	5611287.594	4883306.906	-73.061	-42.175	1087	-0.763837315	-2.987227125	0.945967131
84494	4/21/2016 10:01	B	4686959.736	1976362.264	-73.008	-42.185	4674	-0.488930874	-2.987227125	0.892261815

84494	4/21/2016 11:53	B	185167709.2	2223540.775	-73.009	-42.162	6722	-0.208008154	-2.987227125	0.851475887
84494	4/21/2016 13:32	B	753978638.9	12962234.14	-72.942	-42.173	5965	-0.352012307	-2.987227125	0.730997278
84494	4/21/2016 15:00	B	189003064.5	4172684.521	-72.912	-42.173	5295	-0.290117953	-2.987227125	0.666840894
84494	4/21/2016 21:14	B	511743350.2	337086942.3	-73.444	-42.039	22419	-0.272643063	-1.61514925	0.581118379
84494	4/21/2016 22:19	B	89794974.25	19764549.75	-73.426	-41.952	3877	-0.783346713	-1.61394575	0.605519229
84494	4/21/2016 22:37	A	633849.675	107520.325	-73.451	-41.971	1077	-0.913599246	-1.466625608	0.618832309
84494	4/21/2016 23:07	A	42292.30198	28947.69802	-73.457	-41.972	1845	-0.956978565	-1.461012207	0.623123541
84494	4/22/2016 0:00	B	5950914.248	8450204.252	-73.495	-41.981	3127	-0.964872697	-1.152632312	0.637751333
84494	4/22/2016 0:18	B	882569.3479	784495.6521	-73.488	-41.987	1117	-1.017164509	-1.226212945	0.632239055
84494	4/22/2016 1:40	B	2377472.794	778937.7059	-73.55	-41.999	4923	-0.729924315	-0.847094236	0.64133276
84494	4/22/2016 2:29	B	2314687.386	50379962.61	-73.408	-42.057	2934	0.166505678	-1.88469189	0.54516947
84494	4/22/2016 4:57	A	90924720.64	7726704.36	-73.461	-42.019	8889	-0.992814978	-1.406724383	0.646990409
84494	4/22/2016 5:24	1	1343195.661	311669.3387	-73.389	-42.069	1591	-0.587324139	-1.988118834	0.603106726
84494	4/22/2016 7:11	A	8949288.859	52333379.64	-73.354	-42.017	6425	-1.153422006	-2.987227125	0.691629218
84494	4/22/2016 8:50	B	71748424.81	40405803.69	-73.329	-42.036	5927	-1.057325335	-2.987227125	0.693286346
84494	4/22/2016 9:41	B	286247063.4	1012146669	-73.327	-42.038	3061	-1.020789598	-2.987227125	0.692127348

84494	4/22/2016 10:09	B	23208329.32	1694827.182	-73.29	-42.035	1710	-1.112860616	-2.987227125	0.709200137
84494	4/22/2016 11:21	B	7599788.357	136624.6426	-73.252	-42.062	4309	-0.894494166	-2.987227125	0.715915719
84494	4/22/2016 12:55	B	6956450	494018	-73.226	-42.094	5610	-0.799531669	-2.987227125	0.718281766
84494	4/22/2016 13:03	B	17014871.36	706165.6356	-73.236	-42.086	517	-0.774903061	-2.987227125	0.709730705
84494	4/22/2016 14:47	B	156765488.2	4297288.832	-73.363	-42.007	6241	-1.187169685	-2.987227125	0.690109685
84494	4/22/2016 19:28	B	126902932	13673020.52	-73.31	-42.099	16866	-0.418266785	-2.987227125	0.595494027
84494	4/22/2016 21:00	B	134646479.6	2191832.874	-73.339	-42.129	5516	-0.689894318	-2.495704167	0.506322141
84494	4/22/2016 21:07	B	112435906.1	562487.9021	-73.34	-42.128	421	-0.670306823	-2.476117546	0.507551751
84494	4/22/2016 21:53	B	205578382.3	11093852.24	-73.342	-42.07	2722	-0.467550722	-2.620868528	0.629383494
84494	4/22/2016 22:26	B	10746859.47	605748.5251	-73.312	-42.135	2020	-0.590323348	-2.946331325	0.531911788
84494	4/22/2016 23:35	B	6286849.09	3573323.91	-73.2	-42.185	4116	-0.829359699	-2.987227125	0.712998032
84494	4/23/2016 0:02	B	1245535.871	440700.6285	-73.19	-42.194	1638	-0.874986373	-2.987227125	0.727768254
84494	4/23/2016 0:21	B	5396057.612	667850.8877	-73.212	-42.18	1144	-0.763488122	-2.987227125	0.692609603
84494	4/23/2016 1:14	B	6990283.862	1119804.138	-73.163	-42.228	3176	-0.893213314	-2.987227125	0.774508939
84494	4/23/2016 4:43	B	8671829.283	1526755.717	-73.095	-42.295	12530	-1.019423315	-1.759740535	1.009885847
84494	4/23/2016 5:17	B	8942152.972	1392749.528	-73.108	-42.324	2031	-1.19693769	-1.581206184	1.031731345

84494	4/23/2016 7:00	B	8371789.156	2344169.344	-73.086	-42.372	6204	-1.16328847	-2.157743247	1.042552428
84494	4/23/2016 8:14	B	130821259.6	20796372.94	-73.058	-42.366	4428	-1.187425712	-2.61575541	1.052196984
84494	4/23/2016 8:36	B	62266699.61	18997690.89	-73.024	-42.386	1320	-1.157922167	-2.679866485	1.059348443
84494	4/23/2016 10:56	A	293056.8893	442932.1107	-73.05	-42.613	8391	-1.17194192	-2.057053925	0.775187676
84494	4/23/2016 11:38	B	5193899.953	522914.5467	-73.059	-42.61	2504	-1.151682272	-2.134453827	0.779353897
84494	4/23/2016 14:23	B	12839808.82	585583.68	-73.168	-42.575	9919	-0.911406849	-2.067238636	0.815724287
84494	4/23/2016 20:53	0	50773844.13	6801958.367	-72.919	-42.176	23411	0.207136424	-2.987227125	0.484812102
84494	4/23/2016 21:32	B	1162415762	313436568.3	-72.942	-42.17	2325	-0.162198618	-2.987227125	0.556581129
84494	4/23/2016 22:10	B	6169551.664	11684458.84	-72.969	-42.175	2285	-0.337372473	-2.987227125	0.628546739
84494	4/23/2016 23:16	B	1304903.625	767634.875	-73.014	-42.165	3976	-0.477803074	-2.987227125	0.725623083
84494	4/23/2016 23:48	B	2275905.241	733185.2591	-73.014	-42.181	1920	-0.404925864	-2.987227125	0.732531618
84494	4/24/2016 0:59	B	6724460.761	43881201.74	-73.176	-42.212	4235	-1.092386305	-2.987227125	1.047665932
84494	4/24/2016 1:53	B	16505571.25	28686169.25	-73.245	-42.216	3278	-1.0899164	-2.797361949	1.065158593
84494	4/24/2016 2:43	B	28865036.22	26894332.78	-73.309	-42.217	2970	-0.90878077	-2.033311429	1.039395387
84494	4/24/2016 5:05	B	182482629	13393900.96	-73.267	-42.213	8531	-0.379007633	-2.575659589	1.025585874
84494	4/24/2016 6:47	B	86102189.53	4977222.965	-73.38	-42.207	6104	-0.484269326	-1.340555414	0.901545109

84494	4/24/2016 8:07	B	69447937.42	3279671.08	-73.279	-42.218	4784	-0.387155704	-2.420826543	0.996758094
84494	4/24/2016 10:14	B	71650233.41	2977834.586	-73.329	-41.984	7626	-0.997697154	-2.987227125	1.192535533
84494	4/24/2016 10:25	B	24281953.46	2080259.04	-73.334	-41.989	699	-0.977922021	-2.987227125	1.186412981
84494	4/24/2016 11:20	B	283534991.6	4375644.885	-73.325	-41.98	3299	-1.017782771	-2.987227125	1.190692637
84494	4/24/2016 12:00	B	166790858.5	2972189.999	-73.23	-41.954	2354	-0.584440271	-2.987227125	1.105824114
84494	4/24/2016 13:19	B	83679940.43	2885679.567	-73.154	-41.902	4777	0.333178925	-2.513279107	0.83656017
84494	4/24/2016 14:01	B	19558775756	53720660.68	-73.199	-41.933	2488	0.296231647	-2.686997107	0.860829281
84494	4/24/2016 19:09	B	38911747.83	1058681.165	-73.004	-41.819	18530	-0.432969443	-1.52146333	0.398361354
84494	4/24/2016 20:41	B	3743205115	2284920685	-72.815	-41.838	5510	-1.012466096	-2.849631297	0.249663802
84494	4/24/2016 21:38	A	54072912.11	7979196.391	-72.934	-41.934	3407	0.754029608	-2.899662791	0.395761131
84494	4/24/2016 22:03	B	16045807.52	3594562.981	-72.867	-41.932	1506	0.494331624	-2.64800937	0.427987219
84494	4/24/2016 23:15	B	25596221.93	2371178.573	-72.879	-41.904	4334	-0.567530648	-2.83092884	0.31983807
84494	4/24/2016 23:44	B	38144069.78	2393935.221	-72.917	-41.876	1707	-1.016452571	-2.862742092	0.268778063
84494	4/25/2016 0:19	B	20083448.16	3135700.336	-72.861	-41.903	2105	-0.557494149	-2.539775688	0.328797133
84494	4/25/2016 3:09	B	5570276774	42636798.77	-72.879	-41.804	10191	0.72515411	-2.867624535	0.306459529
84494	4/25/2016 3:55	B	94802766.37	1556838.635	-72.82	-41.787	2798	0.950411986	-2.987227125	0.398346263

84494	4/25/2016 4:49	B	42800117.23	1906858.766	-72.81	-41.772	3222	1.279254235	-2.974508529	0.475892514
84494	4/25/2016 6:31	B	5261000.735	18577185.77	-72.744	-41.709	6120	-0.025008626	-2.865348401	0.765173053
84494	4/25/2016 8:15	B	26175069.73	139184.7737	-72.705	-41.641	6259	1.440658983	-1.582932311	1.075084165
84494	4/25/2016 8:28	B	6024882.594	68247.40594	-72.742	-41.644	769	1.240356299	-1.776519897	1.010119672
84494	4/25/2016 9:33	B	47177143.73	3886560.768	-72.735	-41.616	3902	1.841066043	-1.526730844	1.212805059
84494	4/25/2016 9:47	B	11448369.14	990687.3618	-72.733	-41.619	824	1.760438803	-1.553042012	1.185153073
84494	4/25/2016 10:10	B	30293292.04	1230270.461	-72.737	-41.631	1389	1.580707409	-1.660652765	1.096477083
84494	4/25/2016 11:22	B	7099595.403	6649491.097	-72.899	-41.574	4299	2.587324202	-2.019139909	1.475128002
84494	4/25/2016 11:42	B	10004878.7	686558.3033	-72.915	-41.605	1193	2.124768983	-2.833952431	1.217918398
84494	4/25/2016 12:05	B	12587872.62	1212739.879	-72.928	-41.592	1415	2.257507114	-2.324982805	1.337978022
84494	4/25/2016 13:41	B	47352672.09	1637156.407	-72.965	-41.604	5715	1.870730563	-2.02229942	1.291120983
84494	4/25/2016 15:19	B	42419246.87	4605674.128	-72.972	-41.587	5883	2.070246489	-1.646830558	1.427463988
84494	4/25/2016 18:49	B	41573018.73	10577837.27	-73.027	-41.545	12639	2.065603269	-0.912077395	1.818726096
84494	4/25/2016 20:31	A	1234143.418	25701.5816	-72.81	-41.733	6121	1.227906938	-2.810315685	0.750721117
84494	4/25/2016 21:05	A	8248038.105	1532124.395	-72.803	-41.749	2036	1.648892654	-2.953018488	0.65725177
84494	4/25/2016 21:56	B	2384567.879	524340.6209	-72.789	-41.764	3063	1.463758898	-2.987227125	0.54314756

84494	4/25/2016 22:18	B	1852349.307	1009677.193	-72.793	-41.757	1309	1.65578529	-2.987227125	0.598988165
84494	4/25/2016 22:42	A	438718.8055	1851.194467	-72.817	-41.737	1424	1.292566481	-2.864751758	0.743861273
84494	4/25/2016 23:31	B	877046.8877	249983.6123	-72.804	-41.752	2931	1.789822816	-2.953018488	0.619903492
84494	4/25/2016 23:55	B	1796592.556	1009937.944	-72.811	-41.745	1455	1.660512015	-2.901494368	0.682092267
84494	4/26/2016 1:14	B	2942313.405	969822.5948	-72.784	-41.776	4720	0.990593175	-2.987227125	0.474417822
84494	4/26/2016 1:57	B	1920376071	137877697.1	-72.779	-41.788	2611	0.532424023	-2.987227125	0.417910465
84494	4/26/2016 2:46	B	21610280.8	1496277.705	-72.766	-41.807	2960	0.182614177	-2.944088986	0.374093944
84494	4/26/2016 3:36	B	31459674.8	3744185.703	-72.813	-41.781	2947	0.454615766	-2.987227125	0.408054033
84494	4/26/2016 6:17	B	8411125.428	1346891.572	-72.827	-41.797	9687	0.636806751	-2.987227125	0.34014844
84494	4/26/2016 8:02	B	571877931.1	25842481.92	-72.802	-41.705	6303	-0.100507875	-2.433614557	0.678712039
84494	4/26/2016 9:42	A	6712654.987	35622.01288	-72.884	-41.722	5977	0.106820738	-1.757278977	0.644180203
84494	4/26/2016 11:06	A	77222.94294	11134.05706	-72.84	-41.779	5039	0.600232423	-2.82792264	0.414694374
84494	4/26/2016 11:44	B	679201.0502	206658.9498	-72.843	-41.78	2330	0.640023727	-2.777795709	0.401531038
84494	4/26/2016 15:04	B	16417683.81	203080.6945	-72.842	-41.769	11995	0.539961634	-2.712492347	0.460488955
84494	4/26/2016 18:40	B	287556982.5	140987918	-72.813	-41.719	12942	-0.155160226	-2.587624264	0.647550962
84494	4/26/2016 20:18	1	3460031.916	19480.58422	-72.92	-41.653	5900	1.13929554	-2.66512599	0.939142416

84494	4/26/2016 20:36	B	9640745.778	459539.2224	-72.926	-41.67	1085	0.873366976	-2.317770579	0.83981477
84494	4/26/2016 21:37	B	16187471.29	3680689.208	-72.949	-41.653	3642	1.295353326	-2.667931464	0.946331363
84494	4/26/2016 22:00	B	85883354.4	18718893.6	-72.946	-41.641	1342	1.44186158	-2.704554476	1.019210471
84494	4/26/2016 22:14	A	6739738.707	2597.792917	-72.94	-41.658	884	1.183690591	-2.625996206	0.919158385
84494	4/26/2016 23:23	B	86260030.32	7094042.178	-72.933	-41.668	4124	0.971066716	-2.391620161	0.85009448
84494	4/26/2016 23:34	A	18292.10318	13990.39682	-72.93	-41.664	647	0.981933737	-2.507711642	0.866644276
84494	4/26/2016 23:52	1	109044.5	740544.5	-72.935	-41.68	1082	0.810511232	-2.150245005	0.790708843
84494	4/27/2016 1:00	B	1118292.84	463768.1602	-72.948	-41.662	4084	1.171886561	-2.552893288	0.891214851
84494	4/27/2016 1:34	B	6361567.216	3395197.784	-72.96	-41.673	2050	1.117441272	-2.234043257	0.827276736
84494	4/27/2016 2:29	B	23094652.2	4167884.302	-72.957	-41.654	3306	1.325438671	-2.570156331	0.939549117
84494	4/27/2016 3:13	B	935803.11	291116.89	-72.963	-41.647	2641	0.891290754	-2.564436153	0.924253204
84494	4/27/2016 4:06	B	1534992.079	525651.9209	-72.97	-41.639	3188	1.052593993	-2.310357513	0.968528227
84494	4/27/2016 6:06	B	4393972.679	753311.3211	-72.978	-41.64	7201	1.104527608	-2.156872448	0.975840774
84494	4/27/2016 7:48	A	317453.3088	77029.19123	-73.002	-41.688	6080	0.198733076	-1.521291721	0.736493285
84494	4/27/2016 9:20	B	2675085.719	1010962.781	-73.005	-41.678	5525	0.374988062	-1.580448965	0.776524211
84494	4/27/2016 10:22	A	1840361.101	219211.3995	-73.025	-41.686	3723	0.232189711	-1.272740356	0.750297937

84494	4/27/2016 10:45	B	573113.9896	126134.5104	-73.025	-41.686	1389	0.232189711	-1.272740356	0.750297937
84494	4/27/2016 10:59	B	830915.2617	196403.2383	-73.031	-41.687	845	0.266816563	-1.233322416	0.762615934
84494	4/27/2016 12:03	B	4368414.13	7687688.37	-72.97	-41.656	3831	0.738979055	-2.414241527	0.87387358
84494	4/27/2016 13:40	B	5516330.179	2807546.821	-72.96	-41.637	5824	1.049003423	-2.481951233	0.978237525
84494	4/27/2016 14:38	B	74251058.84	54113.66246	-72.949	-41.657	3462	0.694355908	-2.655320581	0.875379925
84494	4/27/2016 18:25	B	266296927.3	121157975.2	-73.008	-41.608	13658	1.750444092	-1.445717446	1.190372489
84494	4/27/2016 20:08	1	11631789.13	629121.3701	-73.046	-41.62	6186	1.605771495	-1.123552403	1.152586436
84494	4/27/2016 21:42	B	2798946148	412459578.2	-73.032	-41.65	5630	1.18268551	-1.17324145	0.988689976
84494	4/27/2016 21:53	B	59748703.8	14028185.2	-73.053	-41.623	672	1.542852015	-1.055646556	1.133201372
84494	4/27/2016 23:06	B	107607115	540383.5337	-73.051	-41.68	4357	0.429223562	-1.072690603	0.811865599
84494	4/27/2016 23:19	1	1468939.066	88005.93382	-73.026	-41.661	795	0.775986163	-1.312038636	0.870729675
84494	4/28/2016 0:52	B	3610936.331	5107809.669	-73.008	-41.68	5585	0.362563073	-1.556170609	0.773770574
84494	4/28/2016 3:00	B	2830878.99	1681554.01	-73.011	-41.711	7634	0.46429454	-1.361943893	1.085197774
84494	4/28/2016 4:34	1	616655.5215	655145.4785	-72.997	-41.689	5681	0.659962156	-1.616300629	1.179794122
84494	4/28/2016 5:58	B	11604637	2327795.996	-72.998	-41.706	5000	0.494262918	-1.499906771	1.092835399
84494	4/28/2016 8:52	B	69204745.7	9314778.301	-72.929	-41.744	10486	0.00357877	-1.534041766	0.915820117

84494	4/28/2016 9:00	B	12674002.98	1741945.518	-72.926	-41.742	454	0.038314419	-1.54444229	0.926215115
84494	4/28/2016 9:52	B	26464540.83	4840081.669	-72.954	-41.658	3135	0.864089276	-2.573133844	1.306339143
84494	4/28/2016 10:33	B	2372139922	35763134.49	-72.939	-41.652	2455	0.899220429	-2.714553079	1.333947973
84494	4/28/2016 11:32	B	1781803.768	142224.7324	-72.931	-41.642	3511	0.980158823	-2.783862919	1.391795757
84494	4/28/2016 12:43	B	3308460.943	414817.5567	-72.927	-41.635	4291	1.024623079	-2.818882054	1.423359461
84494	4/28/2016 13:29	B	6572141.123	296557.3772	-72.937	-41.644	2763	0.948937344	-2.754588126	1.368798142
84494	4/28/2016 14:20	B	6161133.735	1066900.765	-72.92	-41.626	3077	1.100227962	-2.842228144	1.476509083
84494	4/28/2016 15:11	B	6507504.163	1371390.337	-72.941	-41.63	3003	1.092601441	-2.715282109	1.473423916
84494	4/28/2016 18:15	B	40241548.2	70861061.8	-73.008	-41.655	11051	0.89041882	-1.752724882	1.349659742
84494	4/28/2016 20:01	B	6377485.015	1996230.985	-73.015	-41.675	6354	0.743187627	-1.464975262	1.244722152
84494	4/28/2016 21:05	B	6378102.542	1624666.458	-73.012	-41.684	3877	0.690250656	-1.427744912	1.207418625
84494	4/28/2016 21:39	B	4873508.872	13445655.63	-73.027	-41.69	2030	0.674094006	-1.247197908	1.199695896
84494	4/28/2016 22:44	B	1586439.075	1515985.925	-73.044	-41.677	3921	0.749006237	-1.162855194	1.268496394
84494	4/28/2016 23:03	B	1877192.5	1877192.5	-73.042	-41.692	1116	0.663121646	-1.142281216	1.200629991
84494	4/29/2016 0:30	B	1002453.5	1971036.5	-73.059	-41.644	5211	0.935872667	-1.056398038	1.458697816
84494	4/29/2016 0:54	B	1004330.64	829029.8601	-73.064	-41.617	1441	1.034452575	-0.965740538	1.603757785

84494	4/29/2016 1:49	B	580528085.1	306444398.9	-73.02	-41.56	3307	1.338004952	-1.0246613	1.918644432
84494	4/29/2016 2:38	A	37751947.3	24779537.7	-72.989	-41.593	2934	1.29006041	-1.513190489	1.715147554
84494	4/29/2016 4:10	A	17739801.52	20395930.48	-73.043	-41.581	5545	1.355565276	-1.00573261	1.477952741
84494	4/29/2016 5:51	B	33337256.48	58696586.02	-73.031	-41.579	6036	1.431875144	-1.061489576	1.49158005
84494	4/29/2016 7:26	B	3160766.777	663535.7234	-73.049	-41.61	5702	1.185097006	-1.086550766	1.303387032
84494	4/29/2016 8:29	B	154908149	3889743.523	-73.041	-41.645	3766	0.946339302	-1.207125336	1.09165131
84494	4/29/2016 8:42	B	2949372.652	823692.3482	-73.055	-41.613	820	1.156437404	-1.038232379	1.285800629
84494	4/29/2016 9:08	B	3369278.949	973150.0513	-73.055	-41.613	1510	1.156437404	-1.038232379	1.285800629
84494	4/29/2016 9:22	B	5412518.967	405731.0329	-73.054	-41.629	844	1.0733559	-1.075351836	1.195594223
84494	4/29/2016 10:31	0	77925424.35	6425064.646	-72.996	-41.683	4159	0.642126384	-1.664221283	0.862697546
84494	4/29/2016 11:01	B	160805669.7	78859540.25	-73.002	-41.685	1805	0.609525707	-1.589889122	0.847948008
84494	4/29/2016 11:46	B	192242.9675	458073.5325	-72.99	-41.698	2706	0.50816384	-1.557490207	0.784665419
84494	4/29/2016 12:11	B	1871522.956	569259.5441	-72.991	-41.682	1476	0.646948879	-1.755663019	0.860063632
84494	4/29/2016 12:24	B	710402.0996	694660.4004	-72.986	-41.702	793	0.475642628	-1.586770157	0.766712279
84494	4/29/2016 12:35	B	887336.6538	691155.8462	-72.993	-41.69	647	0.58020701	-1.629664031	0.825871108
84494	4/29/2016 13:56	B	2442576.007	928311.9928	-72.988	-41.685	4868	0.603503336	-1.745496724	0.832079723

84494	4/29/2016 15:35	B	4234939.519	1867686.981	-72.97	-41.707	5969	0.397433506	-1.640521834	0.727206613
84494	4/29/2016 18:04	A	195130.7992	21178195.7	-73.009	-41.651	8932	0.923506043	-1.747609516	1.040343609
84494	4/29/2016 19:46	B	4420717.965	2968000.535	-73.006	-41.648	6107	0.939039729	-1.76356936	1.047724005
84494	4/29/2016 20:38	B	4635831.101	794447.3985	-72.997	-41.616	3094	1.206177344	-1.680006273	1.216617875
84494	4/29/2016 21:28	B	4976496.725	399736.2755	-72.999	-41.599	3047	1.378234564	-1.509002979	1.344329907
84494	4/29/2016 22:15	B	3800645.427	782641.0733	-73	-41.59	2818	1.46003982	-1.425878115	1.411191963
84494	4/29/2016 22:41	B	4631021.021	888703.9789	-72.996	-41.584	1543	1.494867992	-1.396073482	1.433652738
84494	4/30/2016 0:06	B	609562940	372082152.5	-72.981	-41.628	5078	1.114428549	-1.935410368	1.141300683
84494	4/30/2016 0:25	A	28255.90239	12797.09761	-73	-41.641	1181	0.997765448	-1.850598637	1.080019469
84494	4/30/2016 2:10	A	19969291.33	3260214.674	-73.008	-41.629	6299	1.10798236	-1.599387013	1.162479896
84494	4/30/2016 3:57	B	34935454.91	30379890.09	-72.968	-41.646	6384	0.812680793	-2.452679094	1.102843301
84494	4/30/2016 7:13	B	3164167178	8691072.272	-72.982	-41.735	11757	0.761265784	-1.427011356	0.687214255
84494	4/30/2016 8:38	B	10820280.67	338785.8254	-72.966	-41.743	5121	0.83483268	-1.475410372	0.640147478
84494	4/30/2016 8:56	B	7098912	400512.5	-72.933	-41.755	1097	0.970621659	-1.644302348	0.592055782
84494	4/30/2016 10:17	B	825380455.9	20720634.09	-72.952	-41.816	4820	0.041816296	-2.366371191	0.271545807
84494	4/30/2016 10:29	B	94762981.5	1966620.499	-72.933	-41.825	772	-0.150192453	-2.612383655	0.240920868

84494	4/30/2016 11:25	B	16364920.5	552300.5	-72.922	-41.843	3340	-0.433283087	-2.818293595	0.19797535
84494	4/30/2016 11:53	B	9443623.208	1921781.792	-72.885	-41.87	1687	-0.844533093	-2.935984541	0.167395889
84494	4/30/2016 12:02	B	11519720.85	1977851.649	-72.878	-41.867	509	-0.782546686	-2.871245835	0.173311512
84494	4/30/2016 12:12	B	7565929.073	778205.427	-72.873	-41.866	602	-0.739578477	-2.809888781	0.178639138
84494	4/30/2016 13:47	B	3963203.22	1013445.28	-72.881	-41.896	5704	-1.021517031	-2.862188497	0.150638056
84494	4/30/2016 14:35	B	13085267.53	2950914.971	-72.901	-41.887	2899	-0.940754422	-2.987227125	0.149621174
84494	4/30/2016 15:16	B	4297588.757	779207.7428	-72.882	-41.878	2466	-0.898769737	-2.918489065	0.161780545
84494	4/30/2016 19:35	2	1022911.907	21489.09341	-72.972	-41.775	15506	0.914738795	-1.73028895	0.459090608
84494	4/30/2016 21:11	B	168231810.9	70962054.06	-72.948	-41.749	5805	0.811395227	-1.571172406	0.625364222
84494	4/30/2016 21:48	2	1530551.259	5305.240737	-72.927	-41.749	2209	0.678409833	-1.611902985	0.637423304
84494	4/30/2016 21:50	B	187669.3636	11465.13638	-72.927	-41.749	123	0.618674213	-1.611990313	0.647005707
84494	4/30/2016 22:32	3	22951.77603	15694.72397	-72.948	-41.729	2476	0.399951277	-1.539220814	0.718186734
84494	4/30/2016 23:32	B	1001701.585	324838.9148	-72.948	-41.718	3650	0.374119852	-1.624634392	0.765531558
84494	5/1/2016 0:19	B	2780324.899	1047573.601	-72.946	-41.713	2800	0.389806244	-1.662994649	0.775197816
84494	5/1/2016 1:13	B	17229163.28	2205607.218	-72.976	-41.643	3257	0.869901761	-2.298384987	1.134081941
84494	5/1/2016 1:55	B	206380499.6	135340594.9	-72.997	-41.635	2510	0.949227408	-1.835355592	1.18504249

84494	5/1/2016 3:35	A	263022.8543	43939.6457	-73.035	-41.611	5977	1.05794014	-1.189431678	1.305526236
84494	5/1/2016 5:13	A	2974320.236	7352148.764	-73.034	-41.622	5920	0.978543484	-1.23398854	1.243757669
84494	5/1/2016 5:29	B	41169671.02	21734074.98	-73.044	-41.58	918	1.227538281	-0.992261291	1.488592252
84494	5/1/2016 7:00	B	6135529.205	1965509.295	-73.075	-41.563	5455	1.076592717	-0.763853784	1.575644726
84494	5/1/2016 8:46	B	3958514.339	1425581.661	-73.064	-41.589	6394	1.10030001	-0.900760348	1.445831036
84494	5/1/2016 9:17	B	3821712.5	3821712.5	-73.079	-41.597	1865	1.050805357	-0.860346083	1.408340375
84494	5/1/2016 10:02	B	3883216.485	1295042.015	-73.052	-41.646	2649	0.790166129	-1.100149142	1.123919159
84494	5/1/2016 10:56	2	170116.0393	28493.96074	-73.024	-41.67	3276	0.640736107	-1.370686439	0.990689741
84494	5/1/2016 11:35	B	522643967.3	5775621.16	-73.025	-41.658	2349	0.710818758	-1.336801013	1.039190123
84494	5/1/2016 11:44	B	10930196.69	231841.8061	-73.025	-41.658	519	0.710818758	-1.336801013	1.039190123
84494	5/1/2016 13:13	B	11678042.91	547485.5904	-73.079	-41.686	5325	0.568608497	-0.912857002	0.971102964
84494	5/1/2016 15:00	2	123344.6939	243597.8061	-72.895	-41.514	6458	2.255711633	-1.064408391	1.836876962
84494	5/1/2016 19:24	B	24954195.28	2533513.718	-72.796	-41.471	15828	0	-0.749421268	0
84494	5/1/2016 21:05	A	783575.296	1369927.204	-72.938	-41.489	6032	2.22758856	-0.844703473	2.060131582
84494	5/1/2016 21:15	B	21188.56365	2343715.436	-72.935	-41.486	606	2.241665875	-0.815663825	2.063150878
84494	5/1/2016 21:31	B	188263.4377	412440.5623	-72.928	-41.487	963	2.274080788	-0.829813964	2.057506374

84494	5/1/2016 22:19	B	857936.4083	485226.0917	-72.927	-41.477	2915	2.267009683	-0.780921357	2.096041818
84494	5/1/2016 22:49	B	1784071.142	1072629.858	-72.927	-41.473	1803	2.267009683	-0.763238388	2.104044452
84494	5/1/2016 23:06	B	1448975.094	881507.4061	-72.927	-41.476	1004	2.267009683	-0.774940988	2.096041818
84494	5/2/2016 0:09	B	13661844.54	53445.46104	-72.926	-41.494	3778	2.256195566	-0.877587998	2.006463484
84494	5/2/2016 0:49	B	4911499.09	202940.9096	-72.916	-41.495	2418	2.317377506	-0.885884619	1.98816558
84494	5/2/2016 1:30	B	3002914.234	423066.7657	-72.91	-41.493	2465	2.333497219	-0.886410025	1.988757694
84494	5/2/2016 4:50	A	825597540.8	26644300.15	-72.811	-41.564	11971	0.386636844	-1.673063283	0.653903204
84494	5/2/2016 8:36	B	264863168.7	39474445.76	-72.757	-41.59	13575	-0.106833003	-1.567662089	0.455863059
84494	5/2/2016 8:56	B	141948300	51522038.46	-72.73	-41.638	1172	-0.415963176	-1.688824912	0.320872033
84494	5/2/2016 9:52	B	17188735.18	1239019.316	-72.691	-41.68	3356	-0.679731226	-2.262181464	0.204656515
84494	5/2/2016 10:33	B	154140755.1	1215737.943	-72.714	-41.734	2482	-1.094140197	-2.415437008	0.167847408
84494	5/2/2016 11:06	B	4880133.866	126344.6344	-72.673	-41.727	1981	-1.150733571	-1.916335853	0.154032654
84494	5/2/2016 12:53	B	28184572.05	2517037.953	-72.745	-41.693	6400	-0.720237288	-2.920579047	0.229628876
84494	5/2/2016 13:45	B	7635196.239	938488.2607	-72.737	-41.713	3110	-0.864971633	-2.816708088	0.203297543
84494	5/2/2016 14:41	B	5741561.397	1649320.603	-72.787	-41.718	3372	-0.869989086	-2.787346816	0.23753896
84494	5/2/2016 19:08	B	10730748.76	2856629.738	-72.803	-41.782	16043	-1.23687739	-2.987227125	0.201092891

84494	5/2/2016 21:05	B	30273950.34	51370314.16	-73.103	-41.645	7002	0	-0.808583064	0.531833031
84494	5/2/2016 22:08	A	3236655.895	152329.1046	-73.035	-41.585	3815	-0.616526795	-1.085363433	0.589512303
84494	5/2/2016 22:24	A	230715633	8719324.001	-73.047	-41.604	922	-0.685179372	-1.084831891	0.561864545
84494	5/2/2016 22:40	B	16859906.16	1480343.843	-73.045	-41.6	952	-0.680035886	-1.061397215	0.569782206
84494	5/2/2016 23:50	A	8711717.376	4280185.124	-73.073	-41.612	4190	-0.734766057	-0.921195078	0.561920357
84494	5/3/2016 0:04	B	684129.7257	398098.7743	-73.067	-41.608	854	-0.721994545	-0.941632832	0.561969609
84494	5/3/2016 0:27	A	643313.6515	184056.3485	-73.051	-41.623	1390	-0.724183878	-1.079811448	0.534200011
84494	5/3/2016 1:10	B	1043427.699	287366.3006	-73.047	-41.614	2586	-0.702626529	-1.112933794	0.545781768
84494	5/3/2016 1:35	B	1121495.557	539018.4426	-73.049	-41.618	1508	-0.709864203	-1.109030 26	0.537411292
84494	5/3/2016 2:51	B	2410553.439	929869.0614	-73.038	-41.603	4549	-0.663527074	-1.138545914	0.560938379
84494	5/3/2016 4:34	B	4282938.108	1769481.892	-73.042	-41.61	6178	-0.769123345	-1.126098782	0.709829597
84494	5/3/2016 5:00	B	667926.5682	1170979.432	-73.07	-41.632	1546	-0.800312266	-0.965418679	0.699469224
84494	5/3/2016 7:59	B	400891.8726	5095086.627	-73.041	-41.596	10754	-0.724917287	-1.073935968	0.725588642
84494	5/3/2016 8:21	B	267749931.3	267749931.3	-73.035	-41.588	1299	-0.685600321	-1.105028554	0.734488703
84494	5/3/2016 8:27	B	29837509.25	29837509.25	-73.033	-41.588	410	-0.671721173	-1.097199351	0.733638842
84494	5/3/2016 10:12	B	5572418.566	3891754.434	-73.023	-41.567	6244	-0.507532477	-1.03309411	0.759569723

84494	5/3/2016 10:37	A	666545.3356	5977.164401	-73.048	-41.6	1513	-0.741494432	-1.059600745	0.728883683
84494	5/3/2016 11:19	B	7028750.848	3712011.652	-73.057	-41.589	2534	-0.729355624	-0.952471908	0.748728257
84494	5/3/2016 11:43	A	306210.2358	496662.7642	-73.067	-41.601	1472	-0.755145124	-0.921373662	0.742938276
84494	5/3/2016 12:10	B	4772229.834	2531794.666	-73.047	-41.604	1601	-0.752673844	-1.084831891	0.720753655
84494	5/3/2016 12:33	B	1550017.625	609952.875	-73.046	-41.598	1395	-0.741494432	-1.069256248	0.728883683
84494	5/3/2016 14:14	3	32818.69493	29811.80507	-73.041	-41.608	6044	-0.765427967	-1.135917902	0.708109448
84494	5/3/2016 19:00	A	8165412.113	10804590.39	-73.069	-41.575	17138	-0.722537991	-0.829071144	0.763596259
84494	5/3/2016 20:47	B	49050812.77	745025.73	-73.07	-41.571	6426	-0.692546299	-0.819876404	0.772165629
84494	5/3/2016 21:54	B	2942603.66	249624.8401	-73.07	-41.565	4023	-0.694795317	-0.7860472	0.77773196
84494	5/3/2016 22:01	B	3921721.543	295836.9565	-73.068	-41.567	409	-0.692546299	-0.796903576	0.774161472
84494	5/3/2016 22:16	B	3400752.426	276164.0739	-73.066	-41.568	903	-0.692546299	-0.798509396	0.774161472
84494	5/3/2016 23:34	A	102663083.8	2047970.651	-73.064	-41.597	4714	-0.751057187	-0.916114536	0.743586325
84494	5/3/2016 23:45	B	26121439.92	490890.0765	-73.061	-41.598	660	-0.746224333	-0.948004245	0.741093214
84494	5/4/2016 0:06	B	384796.8504	3561821.15	-73.071	-41.597	1235	-0.75353331	-0.907549712	0.745616655
84494	5/4/2016 0:52	B	757863.7231	518664.7769	-73.073	-41.602	2776	-0.762389355	-0.905706499	0.742165386
84494	5/4/2016 2:28	B	8217437.714	13442276.79	-73.05	-41.599	5734	-0.741494432	-1.019111779	0.728883683

84494	5/4/2016 4:12	B	2000996.836	813268.1636	-73.015	-41.641	6274	-0.884739111	-1.558677529	0.665624459
84494	5/4/2016 6:32	B	957741785.8	126893727.2	-73.058	-41.773	8391	-1.031091635	-1.028080674	0.592189533
84494	5/4/2016 7:47	B	117664473.6	12610504.94	-73.056	-41.805	4517	-1.059249113	-1.04322012	0.574552591
84494	5/4/2016 8:05	A	186840377.7	1023514.35	-73.054	-41.834	1075	-1.072441542	-1.118683617	0.562851688
84494	5/4/2016 9:23	B	29318719.02	1141474.979	-73.038	-41.866	4633	-1.053910945	-1.295494764	0.543259979
84494	5/4/2016 10:00	B	24927256.13	2270552.873	-73.061	-41.88	2218	-1.057252493	-1.287928378	0.560881689
84494	5/4/2016 11:05	A	80164224.44	409012.5642	-73.063	-41.983	3945	-1.05044296	-2.802644839	0.578107101
84494	5/4/2016 11:24	B	11240088.23	760874.2726	-73.065	-41.982	1108	-1.046635811	-2.7773875	0.580398412
84494	5/4/2016 11:44	A	2647764.763	1398783.737	-73.051	-41.973	1183	-1.099742881	-2.671480893	0.569034662
84494	5/4/2016 13:54	B	105555490.3	10244240.25	-73.253	-42.138	7851	0.043737299	-2.987227125	0.854565684
84494	5/4/2016 14:50	B	467124502.4	59174702.62	-73.236	-42.168	3342	0.298595903	-2.958424824	0.734362186
84494	5/5/2016 3:53	B	5196590365	162151321.3	-73.137	-42.353	47003	-0.728988198	-1.397846393	0.431550729
84494	5/5/2016 7:59	A	5541184.78	772785.22	-73.105	-42.45	14727	-0.651223166	-1.395434297	0.32409467
84494	5/5/2016 9:12	B	6930364.5	1412880.5	-73.086	-42.54	4367	-0.577922021	-2.070677728	0.207552791
84494	5/5/2016 9:30	B	8612548.772	1545468.228	-73.079	-42.561	1079	-0.579544721	-2.473774205	0.189176208
84494	5/5/2016 10:59	2	701941.8254	433838.1746	-73.111	-42.553	5354	-0.581279993	-2.027647437	0.155166315

84494	5/5/2016 11:02	3	138012.6996	85821.80041	-73.114	-42.55	184	-0.583567886	-2.000008106	0.154112413
84494	5/5/2016 11:12	A	373657.8148	1254.685244	-73.09	-42.558	587	-0.57842806	-2.307183475	0.177943029
84494	5/5/2016 11:55	B	18472894.72	5952131.285	-73.129	-42.536	2616	-0.58700157	-1.770483102	0.160152975
84494	5/5/2016 12:42	B	20612534.58	50585435.92	-73.146	-42.577	2772	-0.550543738	-2.215887403	0.073326377
84494	5/5/2016 13:39	B	1662757.612	643816.8883	-73.063	-42.5	3418	-0.597278485	-1.930506417	0.299524766
84494	5/5/2016 15:19	B	2780770.195	4017322.305	-73.083	-42.562	6035	-0.577041634	-2.408113526	0.180234493
84494	5/6/2016 14:52	A	483441.2445	138535.2555	-72.917	-42.563	84774	-0.419652224	-2.596802989	-0.106359901
84494	5/6/2016 18:25	1	1793618	600608	-72.963	-42.618	12801	-0.480223119	-2.925565143	-0.103891126
84494	5/6/2016 20:07	A	4366386474	461318007.5	-73.076	-42.539	6081	0.429173597	-2.284185667	0.026868607
84494	5/6/2016 21:47	B	2874108745	1517282	-73.16	-42.63	6040	-0.301859258	-2.267325105	-0.30034599
84494	5/6/2016 22:03	B	65040099.39	319263.1084	-73.163	-42.631	910	-0.302728734	-2.207561419	-0.308748089
84494	5/6/2016 22:45	B	256231249.7	30384592.77	-73.177	-42.649	2553	-0.283549554	-1.690317957	-0.366797075
84494	5/6/2016 23:03	B	30239838.28	1353603.72	-73.199	-42.639	1103	-0.290577963	-1.628228051	-0.357691482
84494	5/6/2016 23:36	B	29129918.6	845842.4049	-73.239	-42.661	1940	-0.265299146	-1.105799017	-0.443175788
84494	5/7/2016 0:21	B	11463883.53	383568.9713	-73.261	-42.693	2723	-0.242949714	-0.875626977	-0.541345708
84494	5/7/2016 0:49	B	10548972.59	488419.9056	-73.285	-42.706	1696	-0.237350505	-0.759459404	-0.591092325

84494	5/7/2016 3:04	B	13200953.9	1039067.104	-73.383	-42.764	8091	-0.475309707	-0.36143506	-0.675606256
84494	5/7/2016 4:46	B	51395781.56	19601100.94	-73.297	-42.767	6111	-0.329977483	-0.497236523	-0.67845676
84494	5/7/2016 8:57	B	19542482.14	3345143.86	-73.257	-42.748	15026	-0.30696744	-0.63130687	-0.607617186
84494	5/7/2016 10:41	B	7587473.262	6291557.238	-73.2	-42.733	6241	-0.385987569	-0.800965814	-0.508545268
84494	5/7/2016 11:46	A	1869979629	305255193	-73.291	-42.695	3910	-0.372387331	-0.813230623	-0.49223666
84494	5/7/2016 13:30	B	6687965.228	772311.2724	-73.338	-42.675	6268	-0.452650878	-0.726347945	-0.465753563
84494	5/7/2016 14:31	B	5862616.777	429897.2227	-73.298	-42.613	3631	-0.559688363	-1.113041445	-0.319002172
84494	5/7/2016 18:18	B	17855259.69	939865.307	-73.314	-42.522	13643	-1.097261989	-1.688797576	-0.205407546
84494	5/7/2016 19:55	A	3808642.594	84426.40571	-73.133	-42.609	5793	-0.396026224	-2.59144665	-0.175165136
84494	5/7/2016 21:28	A	38871.63858	32308.86142	-72.873	-42.612	5591	0.707274882	-2.206436924	0.098804956
84494	5/7/2016 22:21	B	990316.6836	22842773.32	-72.841	-42.625	3195	0.149040936	-1.8021289	0.124652516
84494	5/7/2016 22:50	1	390843.5742	516885.4258	-72.871	-42.592	1741	0.807407966	-2.02221277	0.004348344
84494	5/7/2016 23:10	A	24806907.56	35652.44017	-72.88	-42.604	1187	0.860007614	-2.234480177	0.072178114
84494	5/7/2016 23:58	B	6574538.075	1673447.925	-72.908	-42.58	2875	0.530312069	-2.533395445	-0.063156672
84494	5/8/2016 0:34	B	3496770.902	866527.0982	-72.801	-42.596	2162	0	-1.524484969	0
84494	5/8/2016 1:10	B	1014678.144	887533.8562	-72.81	-42.548	2183	-0.075978943	-2.22916134	-0.032415087

84494	5/8/2016 1:57	B	1162184.809	556747.1905	-72.766	-42.53	2784	-0.497825807	-2.158843037	0.173244495
84494	5/8/2016 3:38	B	3830455.872	1410797.128	-72.741	-42.505	6076	-0.922996639	-2.393428898	0.30087334
84494	5/8/2016 4:32	B	12253277.31	1520901.189	-72.816	-42.539	3241	-0.585736964	-2.387315713	0.172304135
84494	5/8/2016 7:30	1	3823375.34	452906.6597	-72.829	-42.529	10685	-0.491998409	-2.549684077	0.188095123
84494	5/8/2016 10:26	1	3670125.853	98626.64653	-72.876	-42.614	10542	-1.199350399	-2.251459757	0.108381482
84494	5/8/2016 11:27	B	17136285.91	3268239.094	-72.869	-42.616	3673	-1.215374474	-2.170235779	0.11054491
84494	5/8/2016 12:34	B	415744367.9	22180100.13	-72.817	-42.517	4033	-0.489715244	-2.856127338	0.225864472
84494	5/8/2016 14:12	1	5134520.441	309900.0591	-72.893	-42.457	5895	-0.486753157	-2.987227125	0.376307485
84494	5/8/2016 18:02	B	334769758.9	76150761.07	-72.886	-42.418	13799	-0.730461628	-2.974197898	0.421306239
84494	5/8/2016 21:25	A	15163.90405	103262.596	-72.864	-42.503	12160	-0.263690179	-2.813704692	0.236752967
84494	5/8/2016 21:53	B	2813192	8320.5	-72.863	-42.505	1654	-0.283817423	-2.78715814	0.232534561
84494	5/8/2016 22:35	B	319416.8404	1950505.66	-72.799	-42.49	2543	-0.752499209	-2.976229423	0.282562237
84494	5/8/2016 23:37	A	1391331.658	1491573.342	-72.769	-42.449	3695	-0.791238081	-2.608966167	0.343943515
84494	5/9/2016 0:19	B	1602933.338	688941.1622	-72.753	-42.472	2574	-0.917573965	-2.810219821	0.320314595
84494	5/9/2016 2:29	B	259192.7531	3131849.247	-72.762	-42.42	7761	-0.708786389	-2.106311513	0.390119355
84494	5/9/2016 4:07	B	1382300.03	1228644.47	-72.736	-42.395	5907	-0.690280179	-1.395766695	0.612237789

84494	5/9/2016 5:39	B	30870554.53	17557498.47	-72.812	-42.445	5474	-0.481706295	-2.952697831	0.522070672
84494	5/9/2016 7:20	A	4612128.838	116261.6624	-72.898	-42.424	6078	-0.321737914	-2.980937153	0.540961984
84494	5/9/2016 8:35	B	1337085.37	384850.6301	-72.917	-42.418	4518	-0.278976895	-2.987227125	0.549943281
84494	5/9/2016 8:54	B	5472784.691	4440991.809	-72.894	-42.429	1107	-0.327382753	-2.982884049	0.53425952
84494	5/9/2016 9:20	B	1780823.926	1087984.074	-72.904	-42.428	1593	-0.308695193	-2.985055587	0.534199262
84494	5/9/2016 10:12	A	2963806005	111990269.5	-72.91	-42.409	3127	-0.315136612	-2.976743839	0.576180859
84494	5/9/2016 10:45	1	292249.2232	259887.2768	-72.883	-42.422	1992	-0.353097741	-2.976743839	0.550197516
84494	5/9/2016 10:52	B	1388921.532	455603.4678	-72.882	-42.422	409	-0.353097741	-2.976369436	0.550197516
84494	5/9/2016 12:28	B	14495840.47	2993284.535	-72.878	-42.428	5731	-0.358944766	-2.975967299	0.539858731
84494	5/9/2016 13:54	B	22082993.54	2162268.96	-72.884	-42.483	5152	-0.365787416	-2.987227125	0.395983526
84494	5/9/2016 14:40	B	10555632.33	1707980.171	-72.882	-42.492	2800	-0.380748833	-2.934505156	0.376362765
84494	5/9/2016 15:33	B	4008835.784	983050.7157	-72.892	-42.495	3167	-0.377131936	-2.904537874	0.363493129
84494	5/9/2016 19:31	B	987241399.2	602619943.3	-72.898	-42.567	14274	-0.667974711	-2.237150161	0.213134843
84494	5/9/2016 20:26	B	161635360.5	43235622.01	-72.866	-42.603	3308	-0.782054401	-2.033225634	0.172161835
84494	5/9/2016 21:20	B	446876259.4	7141358.632	-72.834	-42.583	3258	-0.679272949	-1.764752544	0.218880626
84494	5/9/2016 21:31	B	65564.91934	310973.5807	-72.826	-42.584	653	-0.681085062	-1.747458948	0.218635446

84494	5/9/2016 22:00	B	536566.1466	267993.8534	-72.824	-42.58	1730	-0.664433733	-1.782002399	0.231486709
84494	5/9/2016 22:26	A	2813806.78	2129605.72	-72.855	-42.631	1559	-0.846642662	-2.0449207	0.137460702
84494	5/9/2016 23:10	A	25036436.15	2312256.347	-72.836	-42.651	2639	-0.890273094	-1.90733825	0.111510676
84494	5/9/2016 23:41	B	3031551.37	823487.1297	-72.823	-42.625	1842	0	-1.604252185	0.14514466
84494	5/10/2016 2:58	B	378383428.1	20170836.93	-72.813	-42.809	11850	0	-0.993191457	-0.031391116
84494	5/10/2016 3:50	B	354921796.4	92144687.61	-72.813	-42.828	3117	0	-1.031357113	-0.131908236
84494	5/10/2016 5:25	A	51900.87563	1709539.124	-72.698	-42.83	5662	0	-0.721327705	0
84494	5/10/2016 7:07	B	6657600.5	184832	-72.649	-42.865	6122	0	-0.589407296	0
84494	5/10/2016 8:22	B	3962224.861	544160.1395	-72.654	-42.893	4500	0	-0.652430184	0
84494	5/10/2016 10:14	B	79749.9484	136770.0516	-72.917	-42.932	6736	-1.186969241	-1.242946218	-0.244003008
84494	5/10/2016 10:32	B	330861.4529	2220279.047	-72.934	-42.93	1090	-1.22881457	-1.146927814	-0.244319337
84494	5/10/2016 11:51	B	1268762.08	639080.4205	-72.922	-42.979	4757	-1.183289429	-1.458048169	-0.262641227
84494	5/10/2016 12:14	B	1637091.179	737956.8213	-72.913	-42.976	1334	-1.166825904	-1.514359203	-0.264854941
84494	5/10/2016 13:32	B	453460173.8	5011354.693	-72.892	-43.033	4702	-0.852388236	-2.349455655	-0.319249575
84494	5/10/2016 14:28	B	7928749.173	246527.3274	-72.887	-43.057	3386	-0.749651646	-2.064335766	-0.34014571
84494	5/10/2016 15:15	B	4697193.627	1178126.873	-72.894	-43.053	2789	-0.756076224	-2.041599467	-0.33457691

84494	5/10/2016 19:25	B	810146710	261280.5231	-72.909	-42.962	15032	-1.182068179	-1.482453652	-0.259860378
84494	5/10/2016 21:03	B	295036535.5	11194706.98	-72.92	-42.996	5850	-1.102487325	-1.632591334	-0.269671732
84494	5/10/2016 21:33	B	97603.02323	642539.4768	-72.938	-43.013	1780	-1.037497744	-1.49140072	-0.26521533
84494	5/10/2016 22:15	A	8219037.04	10867713.46	-72.909	-43.1	2568	-0.382802429	-1.466015749	-0.371774122
84494	5/10/2016 22:42	B	11367005.31	4719524.687	-72.907	-43.115	1582	-0.200537889	-1.355331022	-0.398367722
84494	5/10/2016 23:07	B	20084214.51	7187939.986	-72.909	-43.131	1520	0.001364086	-1.251836761	-0.451463347
84494	5/10/2016 23:57	B	50080986.01	31187682.49	-73.009	-43.092	2976	-0.361152366	-1.901469754	-0.170920351
84494	5/11/2016 0:26	B	81632184	31853336	-73.013	-43.114	1731	-0.117288483	-1.901780698	-0.162566792
84494	5/11/2016 1:49	B	160799058.7	40523610.28	-73.072	-43.129	5031	-0.190684838	-1.315101355	0.010275001
84494	5/11/2016 2:41	B	379928107.5	48962045	-73.079	-43.14	3089	-0.203371989	-1.217471639	0.045597155
84494	5/11/2016 6:52	B	1486337670	15012704.11	-73.358	-43.379	15063	-0.370701238	-0.555215352	-0.192920816
84494	5/11/2016 8:06	B	405221703.7	272057774.8	-73.486	-43.424	4420	0.088220033	-0.289139871	-0.591610607
84494	5/11/2016 8:37	B	156549699.7	197496673.3	-73.513	-43.435	1871	0.068122438	-0.251141286	-0.703979881
84494	5/11/2016 9:52	B	3020882	2431012.5	-73.581	-43.455	4501	-0.165770769	-0.339498711	-0.935354302
84494	5/11/2016 11:23	B	14749986.31	8843970.693	-73.637	-43.475	5464	-0.554036692	-0.468062953	-1.032470809
84494	5/11/2016 11:32	B	49727919516	261841924.4	-73.644	-43.482	543	-0.548936973	-0.46812543	-1.036843958

84494	5/11/2016 11:47	B	89391706.9	799979.5967	-73.644	-43.481	911	-0.542403455	-0.476535431	-1.03492805
84494	5/11/2016 12:59	B	65682086.78	803846.2185	-73.716	-43.517	4285	0.153021381	-0.69239935	-0.83373575
84494	5/11/2016 13:13	B	58881750.96	2788015.539	-73.755	-43.539	866	-0.0216145	-0.920692585	-0.686369802
84494	5/11/2016 20:55	B	224485534.1	44516296.38	-74.206	-43.636	27729	0.696796727	-2.043566648	0.82400962
84494	5/11/2016 21:07	B	49331094.55	10794731.95	-74.187	-43.627	695	0.710420008	-1.858711627	0.796261325
84494	5/11/2016 22:38	B	50437242.47	15653054.03	-74.435	-43.599	5502	-0.530655791	-1.222347133	1.259263243
84494	5/12/2016 0:00	A	22489.1555	6567.844503	-74.437	-43.702	4873	0.081344307	-2.903584155	0.958803324
84494	5/12/2016 1:27	B	12418592.51	10070001.99	-74.553	-43.732	5259	-0.152905029	-1.317746282	0.914416073
84494	5/12/2016 1:38	B	2864520.878	2037240.122	-74.552	-43.732	639	-0.151723826	-1.317746282	0.915063561
84494	5/12/2016 2:23	B	2190474.09	1012520.41	-74.638	-43.75	2724	-0.277038031	-1.427063923	0.895280348
84494	5/12/2016 6:45	B	54382341.95	2607430.55	-74.599	-43.749	15690	0.162976048	-1.226919793	0.416225207
84494	5/12/2016 9:43	A	106912796.6	2711473.917	-74.353	-43.977	10693	0.137233239	-2.025490143	-0.377956166
84494	5/12/2016 10:49	B	79335213.61	16327406.89	-74.373	-44.011	3930	-0.079766609	-1.34285483	-0.425811348
84494	5/12/2016 11:13	B	49565708.33	11110978.17	-74.368	-44.007	1462	-0.035984412	-1.378590836	-0.415790879
84494	5/12/2016 19:02	B	319221556.2	28566386.3	-74.409	-43.935	28167	0.411722747	-2.570659388	-0.097253251
84494	5/12/2016 21:52	B	56598696.01	260961.9945	-74.182	-43.801	10174	0.315405268	-2.987227125	-0.150433925

84494	5/12/2016 22:20	B	7747551.429	32038119.07	-74.167	-43.828	1682	0.239221729	-2.987227125	-0.27176763
84494	5/12/2016 23:35	B	8348650295	444984561.3	-74.134	-43.79	4478	0.10608581	-2.987227125	-0.222545383
84494	5/12/2016 23:36	B	1131810205	60329287.27	-74.134	-43.79	76	0.10608581	-2.987227125	-0.222545383
84494	5/12/2016 23:48	B	108962914.7	4958037.753	-74.124	-43.786	756	0.069511886	-2.94444793	-0.224271237
84494	5/13/2016 2:48	B	26160714.03	1088809.974	-74.068	-43.756	10772	-0.218769559	-2.987227125	-0.257012019
84494	5/13/2016 6:34	B	41161726.56	3021523.442	-74.009	-43.719	13588	-0.497180054	-2.987227125	-0.336166605
84494	5/13/2016 8:07	A	41911008.19	117991.809	-73.979	-43.729	5533	-0.757198442	-2.987227125	-0.396534659
84494	5/13/2016 9:22	B	3821526.04	256698.96	-73.943	-43.723	4509	-1.051834674	-2.987227125	-0.436604047
84494	5/13/2016 10:22	B	33570721.53	3169968.465	-73.848	-43.688	3585	-1.253947279	-2.920583303	-0.458526562
84494	5/13/2016 10:53	B	28230007.92	2341014.58	-73.82	-43.682	1885	-1.175154312	-2.831023533	-0.454045916
84494	5/13/2016 11:08	B	14912833.5	930891.4964	-73.797	-43.683	880	-1.07345727	-2.816250183	-0.446337103
84494	5/13/2016 13:17	B	19259466.08	2015346.418	-73.707	-43.654	7763	-0.655000264	-1.633167227	-0.494541479
84494	5/13/2016 13:38	B	20358602.32	2664679.679	-73.686	-43.652	1245	-0.74550453	-1.299322085	-0.523892307
84494	5/13/2016 18:50	B	38681392.58	27244065.42	-73.558	-43.661	18751	-1.094955623	-0.71050039	-0.515474845
84494	5/13/2016 20:29	B	95943595.38	2410245.122	-73.444	-43.692	5924	-0.352394576	-0.833979148	-0.397650905
84494	5/13/2016 21:43	B	54325496.33	1571920.67	-73.393	-43.693	4440	-0.143109188	-0.732098826	-0.325505783

84494	5/13/2016 23:26	B	199223181.2	2331437.34	-73.317	-43.726	6206	-0.370710032	-0.878412633	-0.127953367
84494	5/14/2016 0:43	B	2947745425	86629787.75	-73.271	-43.737	4594	-0.515721073	-1.068475276	-0.085123301
84494	5/14/2016 3:22	B	1404613060	20631678.18	-73.138	-43.745	9564	-0.409769416	-2.168953977	-0.13420352
84494	5/14/2016 4:02	B	358992142.5	29536477.98	-73.214	-43.746	2397	-0.682510232	-1.378836266	-0.030390683
84494	5/14/2016 8:02	B	254391607.5	26860221.54	-73.099	-43.778	14355	1.086845604	-2.960226115	-0.37098463
84494	5/14/2016 9:44	B	76284846.21	5747899.793	-73.079	-43.773	6141	1.234407354	-2.88917958	-0.399247309
84494	5/14/2016 10:33	B	436081627.3	19818329.2	-73.116	-43.711	2935	-0.767940135	-2.565694048	-0.170358778
84494	5/14/2016 10:59	B	144058180.4	6948571.565	-73.087	-43.756	1546	0.416265196	-2.744152877	-0.305857552
84494	5/14/2016 13:04	A	18909748574	590420999	-72.881	-43.624	7546	-1.013363057	-0.819852093	-0.05302549
84494	5/14/2016 13:48	B	3801215413	33409077	-72.814	-43.61	2639	0	-0.690978648	-0.026602961
84494	5/14/2016 14:44	B	879281894.5	28381918.01	-72.97	-43.609	3342	-1.010995892	-1.08696745	-0.075068584
84494	5/14/2016 18:37	B	1117935613	36354864.5	-72.848	-43.534	13946	0	-0.567590852	0
84494	5/14/2016 20:23	B	3754214132	4937228.039	-72.809	-43.54	6417	0	-0.482748915	0
84494	5/14/2016 21:06	B	181420709.3	339675.2148	-72.811	-43.531	2546	0	-0.472812978	0
84494	5/14/2016 21:31	B	99619026.51	925418.4932	-72.858	-43.543	1497	0	-0.620355124	0
84494	5/15/2016 0:31	B	5429480.638	3888112.362	-73.284	-43.489	10780	-0.547932653	-0.922285984	-0.337579771

84494	5/15/2016 3:40	0	2464746.809	13390647.19	-73.438	-43.253	11377	1.514876607	-0.698957128	-0.868581342
84494	5/15/2016 6:12	B	145269842.5	45975960.01	-73.444	-43.175	9117	-0.96633835	-1.226327825	-0.672814534
84494	5/15/2016 7:47	B	276773793.1	13667871.86	-73.444	-43.178	5672	-0.947025539	-1.180153584	-0.671156205
84494	5/15/2016 8:26	B	168960071.6	28733060.87	-73.448	-43.152	2386	-0.917225277	-1.461311251	-0.667307426
84494	5/15/2016 10:07	B	1534986746	38508234.36	-73.434	-43.05	6023	-0.977111509	-1.010083625	-0.533485103
84494	5/15/2016 10:45	B	31838988.59	737143.4076	-73.407	-43.033	2299	-1.013247537	-0.90022116	-0.513861091
84494	5/15/2016 12:40	B	109750014.7	6843426.318	-73.372	-43.058	6866	-1.096791252	-1.018129451	-0.519919512
84494	5/15/2016 14:23	B	464997070.5	21074414.55	-73.351	-43.05	6234	-1.174383244	-0.910902746	-0.505694027
84494	5/15/2016 18:23	B	23653184.45	1068980.554	-73.261	-42.937	14392	-0.228628907	-0.480464649	-0.36019585
84494	5/15/2016 20:04	B	23284231.55	22761941.45	-73.176	-42.849	6032	0.104167194	-0.487317815	-0.024395595
84494	5/15/2016 21:21	B	18515505.87	5168379.134	-73.16	-42.871	4640	0.170748895	-0.56861329	-0.074405413
84494	5/15/2016 21:49	B	8970315.253	3941157.247	-73.12	-42.885	1660	0.256030119	-0.687941482	-0.069377655
84494	5/15/2016 22:19	B	9855456.088	924226.4122	-73.063	-42.918	1795	0.394735304	-0.953191751	-0.056339529
84494	5/15/2016 22:56	B	10425701.99	1096932.513	-73.049	-42.904	2251	0.398185092	-0.876523366	0.039947946
84494	5/15/2016 23:58	B	28422400.12	4493816.877	-73.029	-42.913	3675	0.447696658	-0.937153035	0.075229301
84494	5/16/2016 2:31	B	6897143.429	5551312.571	-72.898	-42.866	9191	-0.781290373	-1.076094897	0.489584887

84494	5/16/2016 3:26	B	22456882590	2370837480	-72.897	-42.879	3298	-0.806868923	-1.303816955	0.519180989
84494	5/16/2016 5:00	B	14873447.37	2711281.63	-72.828	-42.868	5673	0.624190055	-1.323025398	0.573363609
84494	5/16/2016 5:56	B	83162981.41	646276.5897	-72.804	-42.828	3345	0	-1.016389303	0.493862628
84494	5/16/2016 8:04	B	34924593.02	851061.4818	-72.706	-42.796	7704	0	-0.725246405	0
84494	5/16/2016 8:49	B	30903221.5	1302460.5	-72.674	-42.78	2655	0	-0.646053909	0
84494	5/16/2016 9:42	1	35461782.45	1910992.052	-72.84	-42.768	3216	-0.370728829	-1.230503331	0.425001493
84494	5/16/2016 11:19	A	42485.54609	19316.45391	-72.905	-42.691	5792	0.448718766	-2.407727145	0.643456074
84494	5/16/2016 12:07	B	436793519.2	64417781.78	-72.919	-42.639	2896	-0.068999117	-2.963357838	0.831717795
84494	5/16/2016 13:09	A	2312103.763	610216.2368	-72.893	-42.579	3737	0.286837365	-2.282446639	0.824520272
84494	5/16/2016 14:42	B	56414797.84	36994906.16	-72.882	-42.472	5571	1.393587616	-2.987227125	0.773313497
84494	5/16/2016 18:16	B	9782199.209	21458445.29	-72.959	-42.289	12845	0.09098613	-2.987227125	1.133598729
84494	5/16/2016 19:54	B	70488009.39	1403141.109	-72.935	-42.228	5877	-0.968582857	-2.987227125	1.230212777
84494	5/16/2016 21:45	A	18018321.22	370392.7801	-73.089	-42.159	6669	1.81813083	-2.987227125	1.006827601
84494	5/16/2016 21:53	B	6285553.136	922811.8644	-73.099	-42.153	450	2.170372566	-2.987227125	0.915162916
84494	5/16/2016 22:56	B	43239425.97	13441632.53	-73.104	-42.148	3808	2.325708982	-2.987227125	0.86056294
84494	5/16/2016 23:35	B	949827058.9	100277415.1	-73.144	-42.124	2298	1.269006647	-2.987227125	0.50765291

84494	5/17/2016 0:27	B	5984990.57	700565.4297	-73.194	-42.092	3157	1.97261191	-2.987227125	0.679749398
84494	5/17/2016 1:13	B	5115856.75	697004.25	-73.194	-42.092	2723	1.97261191	-2.987227125	0.679749398
84494	5/17/2016 1:21	A	259451.264	139657.736	-73.187	-42.095	515	1.983623281	-2.987227125	0.644253344
84494	5/17/2016 2:59	1	7187534.816	395366.184	-73.221	-42.073	5891	0.706356356	-2.987227125	0.869966727
84494	5/17/2016 4:41	A	993965258.6	1204390.364	-73.098	-42.004	6093	-0.025617829	-2.987227125	1.183834783
84494	5/17/2016 5:41	B	2749472340	78613839.65	-73.104	-41.973	3596	0.469873753	-2.664665757	1.076407351
84494	5/17/2016 7:28	B	1.67042E+11	33112600888	-73.206	-41.985	6444	-0.385760875	-2.987227125	1.119642063
84494	5/17/2016 8:35	B	53943602.32	10124503.68	-73.219	-41.975	4011	-0.617651018	-2.987227125	1.073621553
84494	5/17/2016 9:16	B	46341315.6	8216256.904	-73.209	-41.989	2475	-0.597110221	-2.987227125	1.08738182
84494	5/17/2016 10:20	B	18689927833	844453291.4	-73.225	-42.091	3809	0.292075535	-2.987227125	1.03624248
84494	5/17/2016 11:00	B	486489164	13946976.51	-73.198	-42.115	2404	-0.129639345	-2.987227125	0.98939284
84494	5/17/2016 11:38	A	26437958343	202412092.8	-73.128	-42.137	2304	0.382178999	-2.987227125	1.102635782
84494	5/17/2016 12:00	A	10115809.62	10921102.88	-73.149	-42.155	1275	0.47086802	-2.987227125	1.156391406
84494	5/17/2016 12:42	B	99705898.95	55429039.55	-73.14	-42.173	2534	0.429966619	-2.987227125	1.184136758
84494	5/17/2016 13:15	B	191294233.2	51844575.76	-73.135	-42.176	1982	0.349058376	-2.987227125	1.203912441
84494	5/17/2016 13:41	B	291476613.7	48861116.77	-73.08	-42.12	1587	0.077651453	-2.987227125	1.424712128

84494	5/17/2016 14:24	B	289568943.6	15565522.93	-73.064	-42.172	2536	-0.151705641	-2.987227125	1.361211067
84494	5/17/2016 19:44	B	88630633244	2756215044	-73.154	-42.007	19210	-0.282260749	-2.987227125	1.376882675
84494	5/17/2016 21:17	B	466381207.8	138740724.7	-72.983	-41.979	5603	1.785165809	-2.987227125	1.22224496
84494	5/17/2016 22:52	2	747769.5275	40208.47253	-73.005	-41.898	5686	1.126547713	-1.911224153	0.654359663
84494	5/17/2016 23:11	B	66677685.94	2522606.558	-72.992	-41.902	1179	1.312051517	-2.098820092	0.663883586
84494	5/18/2016 0:21	B	12839636.07	2441176.431	-72.948	-41.91	4166	1.006895534	-2.565041945	0.607364596
84494	5/18/2016 0:54	B	75266291.92	1674990.579	-72.941	-41.914	2011	1.148250473	-2.688300607	0.622932971
84494	5/18/2016 2:41	A	14942315.35	564853.1473	-72.815	-41.976	6405	0.953441899	-1.714499217	0.715338327
84494	5/18/2016 4:25	B	2219634.61	419895.3899	-72.723	-42.005	6229	0.097963743	-1.575415456	0.494587261
84494	5/18/2016 5:32	B	4509324.445	395891.5549	-72.673	-42.042	3999	-0.485101057	-1.73430026	0.654807743
84494	5/18/2016 7:14	B	19869587.54	977984.9631	-72.584	-42.083	6146	-1.280003558	-1.412861553	0.729847499
84494	5/18/2016 8:54	B	176252271.8	24401802.16	-72.636	-42.198	5996	-0.644916642	-2.660280156	0.542953468
84494	5/18/2016 10:32	0	12242929.7	230903.2998	-72.83	-42.207	5871	-0.159111099	-2.987227125	1.09098459
84494	5/18/2016 11:03	B	78244755.53	55126154.97	-72.804	-42.207	1845	0.262950248	-2.987227125	1.004076727
84494	5/18/2016 11:46	B	16106676.92	4157547.576	-72.82	-42.22	2624	0.010316626	-2.987227125	1.053995309
84494	5/18/2016 12:42	A	923863.5	309829.5	-72.836	-42.21	3345	-0.208843631	-2.987227125	1.107254209

84494	5/18/2016 13:16	B	888114.5443	140958.4557	-72.85	-42.213	2041	-0.339077116	-2.987227125	1.145941976
84494	5/18/2016 14:04	B	2138312	333744.5	-72.842	-42.203	2873	-0.303149422	-2.987227125	1.122976525
84494	5/18/2016 19:39	B	212568610.8	98045211.65	-73.081	-42.084	20099	-0.264590272	-2.987227125	1.210630225
84494	5/18/2016 20:46	B	193822656	79711740.98	-72.996	-42.009	4007	0.473231303	-2.987227125	0.88881712
84494	5/18/2016 22:49	B	19325870.75	2562307.245	-72.927	-41.822	7368	-0.473017739	-2.70686341	0.122105091
84494	5/19/2016 0:01	B	334955276.1	55008558.35	-72.959	-41.827	4373	-0.444159395	-2.189370509	0.102096871
84494	5/19/2016 2:20	A	45135.48596	32506.51404	-72.879	-41.834	8333	-0.5870661	-2.961605833	0.178421924
84494	5/19/2016 4:04	A	15903.95072	39184.54928	-72.855	-41.788	6211	0.855374047	-2.718121297	0.410493367
84494	5/19/2016 5:22	B	1224658.941	407666.0586	-72.844	-41.769	4717	1.179610183	-2.712492347	0.54024932
84494	5/19/2016 8:18	B	3842992.349	2331836.151	-72.809	-41.727	10554	0.827603712	-2.702987906	0.672890297
84494	5/19/2016 8:46	B	5321351.455	19441888.55	-72.817	-41.75	1629	0.418719598	-2.901494368	0.680615913
84494	5/19/2016 9:50	B	5893930.2	10737442.3	-72.799	-41.721	3839	0.542460412	-2.729569592	0.698178472
84494	5/19/2016 10:29	B	207996243.7	113601.3397	-72.777	-41.631	2364	0.32402859	-1.883185041	0.453850239
84494	5/19/2016 11:43	B	13404373.43	445614.5712	-72.834	-41.6	4473	2.47150283	-2.515751845	0.50728962
84494	5/19/2016 14:36	B	186820605.7	17794702.83	-73.124	-41.658	10378	0	-0.745026027	0
84494	5/19/2016 19:17	A	18544059.41	588585951.1	-72.72	-41.675	16825	-0.154521067	-2.538370161	0.697762535

84494	5/19/2016 21:05	B	286299587.9	62612894.61	-72.664	-41.803	6512	0	-1.446671588	0
84494	5/19/2016 22:20	A	16417212.89	117780.1132	-72.794	-41.766	4460	0.37588342	-2.987227125	0.673461169
84494	5/19/2016 23:33	B	16024147.94	5089788.063	-72.845	-41.838	4385	-0.550589859	-2.739585953	0.330483423
84494	5/20/2016 0:00	B	13028477.25	2158527.746	-72.847	-41.819	1643	-0.394362072	-2.934154448	0.351685054
84494	5/20/2016 2:01	A	90866.61613	367702.3839	-72.795	-41.735	7273	-0.061610656	-2.94021851	0.727807458
84494	5/20/2016 2:57	B	3346637.262	1310887.738	-72.816	-41.739	3351	0.440020211	-2.884601214	0.686377428
84494	5/20/2016 6:50	0	158594893.9	9871731.114	-72.765	-41.745	13993	0.824612009	-2.929316541	0.601483435
84494	5/20/2016 8:33	B	39183666.67	8736655.325	-72.788	-41.747	6153	0.659345623	-2.987227125	0.633130953
84494	5/20/2016 9:42	A	852820.8244	646381.6756	-72.804	-41.763	4139	1.643636028	-2.963606875	0.580266646
84494	5/20/2016 11:38	B	918186581.3	28547829.21	-72.765	-41.783	6960	1.961626434	-2.915750887	0.317419573
84494	5/20/2016 11:44	B	17378989.64	454308.8588	-72.763	-41.783	388	1.919808814	-2.910645441	0.324646312
84494	5/20/2016 13:20	B	5867490.742	366443.7584	-72.77	-41.775	5735	2.164976135	-2.987227125	0.392476805
84494	5/20/2016 14:18	B	9976653.403	567645.0971	-72.772	-41.772	3455	2.231732379	-2.987227125	0.416823789
84494	5/20/2016 15:05	B	9434568.12	566336.3797	-72.772	-41.768	2823	2.104995392	-2.987227125	0.446364143
84494	5/20/2016 21:18	B	5753092.285	10704008.21	-73.013	-41.784	22413	-0.393991077	-1.375594853	0.388977283
84494	5/20/2016 21:55	B	2946361.598	1203083.402	-72.998	-41.754	2231	-0.215274762	-1.418080211	0.398359772

84494	5/20/2016 23:41	A	907347.1364	594395.3636	-72.858	-41.772	6343	0.654562053	-2.464048773	0.614080921
84494	5/21/2016 1:26	B	246234609.3	69818073.22	-72.877	-41.771	6293	0.226849766	-2.097017323	0.621033905
84494	5/21/2016 1:38	1	760489.0884	575835.9116	-72.89	-41.761	721	-0.026407383	-1.845253087	0.633870103
84494	5/21/2016 3:22	A	492333.3911	6018550.609	-72.886	-41.735	6266	0.32402695	-1.708153887	0.61085445
84494	5/21/2016 4:57	B	1395524.204	593638.296	-72.889	-41.732	5669	0.140833319	-1.679190872	0.44679924
84494	5/21/2016 6:41	A	94478.17008	4243.829923	-72.799	-41.788	6255	2.328541955	-2.987227125	0.103480617
84494	5/21/2016 8:21	B	50207534.21	396688.2938	-72.778	-41.788	5999	1.975387868	-2.987227125	0.070729444
84494	5/21/2016 9:17	A	11139.45225	15829.54775	-72.796	-41.79	3364	2.05698379	-2.987227125	0.068385909
84494	5/21/2016 9:36	B	205142003	14729647.46	-72.794	-41.785	1124	2.603078462	-2.987227125	0.135072151
84494	5/21/2016 10:56	B	4458110.67	347926.33	-72.801	-41.79	4794	2.166831613	-2.987227125	0.093251682
84494	5/21/2016 11:12	B	5013778.839	361650.161	-72.804	-41.796	939	2.029433705	-2.987227125	0.063763961
84494	5/21/2016 13:05	B	55998681309	2984765876	-72.726	-41.807	6792	1.036425323	-2.987227125	-0.015043561
84494	5/21/2016 18:56	B	136308180	18293926	-72.789	-41.747	21096	0.621515768	-2.987227125	0.424422058
84494	5/21/2016 20:41	B	305727.3326	83415282.67	-72.692	-41.73	6267	1.297061435	-2.202149769	0.472850223
84494	5/21/2016 21:31	B	4910626.082	8026454.418	-72.683	-41.713	3021	1.30033917	-2.134573513	0.576822668
84494	5/21/2016 22:25	0	40239984.88	896723.617	-72.656	-41.781	3238	0	-1.407939598	0.303299925

84494	5/21/2016 23:09	B	142708384.2	9522098.332	-72.684	-41.716	2625	1.17069038	-2.087528987	0.561477141
84494	5/21/2016 23:32	B	1328378281	95373633.38	-72.663	-41.667	1391	2.526574626	-1.817987408	0.439552255
84494	5/22/2016 0:04	B	584813.8394	21174151.16	-72.722	-41.669	1889	2.500690142	-2.315231945	0.27483958
84494	5/22/2016 1:20	A	18603.33454	113249.1655	-72.767	-41.634	4612	-0.064017294	-1.773932107	0.107983713
84494	5/22/2016 2:14	B	925806.4644	275066.0356	-72.779	-41.62	3195	0.013105477	-1.935996613	0.120874274
84494	5/22/2016 2:57	A	258654197.6	21267743.42	-72.788	-41.617	2578	0.10841925	-2.04308573	0.144545547
84494	5/22/2016 4:40	B	6632701.771	33376527.23	-72.93	-41.622	6202	0.180992655	-2.795223344	0.088345234
84494	5/22/2016 4:46	B	752555.8889	2909810.611	-72.932	-41.624	368	0.217614129	-2.735969819	0.073176333
84494	5/22/2016 6:22	B	5127068.417	3501213.583	-72.986	-41.609	5725	0.405771456	-1.678847299	0.03472868
84494	5/22/2016 8:08	B	7203123.48	1069758.52	-73.048	-41.611	6392	0.807546755	-1.086550766	0.080284508
84494	5/22/2016 8:51	B	37309296.7	1174717.803	-73.042	-41.611	2550	0.728721423	-1.141216424	0.05653527
84494	5/22/2016 10:36	B	3553005186	17377459.44	-73.017	-41.669	6314	0.385175769	-1.450218115	-0.039069165
84494	5/22/2016 12:45	B	2603246.571	639093.4287	-73.007	-41.677	7739	0.500544601	-1.592071838	-0.018761452
84494	5/22/2016 18:46	B	24490559.95	206512420	-72.978	-41.732	21686	0.872506788	-1.424723917	0.256520199
84494	5/22/2016 21:06	B	2280410.998	5124291.502	-73.003	-41.743	8386	0.476079396	-1.305333095	0.277835902
84494	5/22/2016 21:40	B	3247314	4058486	-72.998	-41.739	2041	0.569178946	-1.33834	0.267161811

84494	5/22/2016 22:07	B	2669285.499	5260363.001	-72.982	-41.732	1635	0.772185943	-1.41895206	0.252780015
84494	5/22/2016 23:21	A	1.2786E+11	5776792173	-73.031	-41.858	4407	-0.614798438	-1.37284124	0.530835154
84494	5/22/2016 23:32	B	1082781975	42859109.34	-73.018	-41.855	661	-0.333520661	-1.327849615	0.533274752
84494	5/23/2016 2:38	B	67091947821	20454971.04	-73.067	-41.95	11187	2.696237458	-2.263078295	-0.197664515
84494	5/23/2016 4:16	B	10243822.71	335305.7942	-73.086	-42.005	5879	0.756909737	-2.987227125	0.112315197
84494	5/23/2016 6:18	A	8110.135712	172194.8643	-73.057	-42.05	7328	0.939208549	-2.987227125	0.339471828
84494	5/23/2016 7:51	B	1518720.894	522545.6065	-73.068	-42.096	5545	0.097116526	-2.987227125	0.564326981
84494	5/23/2016 8:31	B	45865371.84	232809.1595	-73.07	-42.111	2412	-0.167343134	-2.987227125	0.603783779
84494	5/23/2016 9:08	B	7629606.907	173194.0933	-73.07	-42.11	2231	-0.167343134	-2.987227125	0.603783779
84494	5/23/2016 11:44	A	60480.875	144193.625	-73.056	-42.094	9365	0.177607678	-2.987227125	0.543682724
84494	5/23/2016 11:49	B	371614.4913	75530.50873	-73.054	-42.095	296	0.177607678	-2.987227125	0.543682724
84494	5/23/2016 12:25	B	407793912.2	15422032.79	-73.032	-42.092	2154	0.299393566	-2.987227125	0.512289693
84494	5/23/2016 13:17	B	19438863.1	26924685.4	-73.09	-42.155	3108	-0.99652941	-2.987227125	0.692075608
84494	5/23/2016 13:59	B	63163376.37	3627040.63	-72.992	-42.08	2541	0.836047284	-2.987227125	0.368918043
84494	5/23/2016 15:42	B	2327122.355	3944253.645	-72.993	-42.088	6146	0.653095655	-2.987227125	0.412244832
84494	5/23/2016 18:38	B	6416794.523	1024311.977	-72.942	-42.121	10614	0.353921382	-2.987227125	0.374534071

84494	5/23/2016 20:14	0	69485841.89	5222812.613	-72.862	-42.111	5749	0.109809112	-2.987227125	0.082382954
84494	5/23/2016 21:25	B	2407197225	129530875.3	-72.835	-42.101	4235	-0.480990008	-2.987227125	0.002454037
84494	5/23/2016 21:56	B	939963943.1	17104279.43	-72.827	-42.097	1856	-0.68200218	-2.987227125	-0.013273951
84494	5/23/2016 22:18	B	16138399.77	1020162.726	-72.83	-42.085	1332	-0.858970885	-2.987227125	-0.024747691
84494	5/23/2016 23:01	B	170721666.8	57526.17697	-72.81	-42.076	2595	-1.059261766	-2.987227125	-0.036008957
84494	5/23/2016 23:12	B	7941742.284	212307.7164	-72.845	-42.074	671	-0.647068764	-2.987227125	-0.016635541
84494	5/24/2016 0:01	B	10701815.56	645374.9418	-72.831	-42.062	2889	-0.934832286	-2.957159287	-0.032302449
84494	5/24/2016 0:37	B	4689229.454	984799.0459	-72.877	-42.066	2182	0.040184537	-2.987227125	-0.01360641
84494	5/24/2016 2:19	B	20963965.77	1884459.23	-72.912	-42.014	6107	-0.470639496	-2.987227125	-0.133636078
84494	5/24/2016 3:12	A	221328.5338	17401.96618	-73.073	-42.017	3175	0.968427551	-2.987227125	0.173714602
84494	5/24/2016 3:55	A	68046.23274	59928.26726	-73.018	-42.024	2604	0.971769063	-2.987227125	0.144150896
84494	5/24/2016 6:02	B	34336755.84	5737828.657	-73.064	-42.024	7625	-1.287264076	-2.987227125	0.369926989
84494	5/24/2016 9:01	A	83491662150	9901505975	-72.645	-42.193	10756	-1.079461527	-2.987227125	0.247180058
84494	5/24/2016 9:42	B	2680669372	125258988.9	-72.663	-42.169	2429	-1.1441731	-2.987227125	0.208617439
84494	5/24/2016 11:20	B	56710.06	606722.94	-72.98	-42.136	5887	-1.209537945	-2.987227125	0.379686419
84494	5/24/2016 12:54	B	601216.7352	4445855.265	-72.89	-42.104	5656	-1.084760115	-2.987227125	0.320646293

84494	5/24/2016 13:38	B	3809675.35	3573685.15	-72.904	-42.116	2623	-1.076510718	-2.987227125	0.335326088
84494	5/24/2016 15:21	B	14961135.13	43443405.38	-72.947	-42.21	6184	-1.217218342	-2.987227125	0.400864648
84494	5/24/2016 20:02	B	33206885.6	16293514.9	-72.939	-42.188	16828	-1.178104149	-2.987227125	0.391393522
84494	5/24/2016 21:49	B	1276752438	2248409252	-72.92	-42.259	6428	-1.101424665	-2.987227125	0.390720494
84494	5/24/2016 22:32	A	3056468.28	46552.71967	-72.918	-42.237	2611	-1.114333004	-2.987227125	0.389810183
84494	5/24/2016 22:54	B	4331429.013	2460633.487	-72.926	-42.232	1318	-1.139737318	-2.987227125	0.394464079
84494	5/24/2016 23:33	A	5399492171	1174082954	-72.898	-42.215	2334	-1.098343226	-2.987227125	0.380588786
84494	5/25/2016 1:15	B	13485701.53	3129655.465	-72.91	-42.221	6094	-1.096344752	-2.987227125	0.380992322
84494	5/25/2016 1:59	B	47209743.1	2143835.396	-72.905	-42.217	2678	-1.082966033	-2.987227125	0.376906209
84494	5/25/2016 2:51	B	9006004.856	385967.644	-72.907	-42.218	3128	-1.093629942	-2.987227125	0.379441132
84494	5/25/2016 3:37	A	82824.5	8580.5	-72.954	-42.175	2754	-1.191285314	-2.987227125	0.391380734
84494	5/25/2016 5:50	B	8309441.688	1832351.312	-72.991	-42.158	7950	-1.286819614	-2.987227125	0.916418422
84494	5/25/2016 7:37	0	37221215.43	9951211.072	-72.892	-42.229	6416	-1.119305651	-2.987227125	0.856683161
84494	5/25/2016 9:21	A	35959309.71	637898.791	-72.902	-42.248	6237	-1.124538115	-2.987227125	0.852616061
84494	5/25/2016 10:30	0	93263584.72	2979139.783	-72.825	-42.239	4167	-1.129525906	-2.95730722	0.809792068
84494	5/25/2016 11:00	B	122094323.7	26550374.79	-72.815	-42.239	1773	-1.130121569	-2.958338941	0.802778725

84494	5/25/2016 12:20	B	1719894370	206287438.1	-72.843	-42.195	4809	-1.061516502	-2.987227125	0.827600538
84494	5/25/2016 13:24	B	533051834.6	96047366.41	-72.904	-42.228	3842	-1.120219659	-2.987227125	0.862138698
84494	5/25/2016 14:58	B	70449651.64	554698.8586	-72.901	-42.266	5625	-1.128784184	-2.987227125	0.840720959
84494	5/25/2016 18:15	A	626464071.3	626464071.3	-72.918	-42.007	11825	-0.495168435	-2.987227125	0.678946651
84494	5/25/2016 19:49	B	483117478.1	249108748.4	-72.919	-41.99	5650	-0.563033678	-2.987227125	0.637421992
84494	5/25/2016 21:33	A	162537.5	58587.5	-72.868	-42.053	6226	-0.819129565	-2.987227125	0.768978937
84494	5/25/2016 22:01	3	128436.3109	7208.189079	-72.939	-42.067	1728	-0.549580371	-2.987227125	0.800845406
84494	5/25/2016 23:09	B	1862769.68	543451.3205	-72.936	-42.066	4051	-0.558388088	-2.987227125	0.800306386
84494	5/25/2016 23:45	B	2630502.805	811879.6951	-72.946	-42.059	2166	-0.478321372	-2.987227125	0.781459101
84494	5/26/2016 1:36	B	166121136.3	166121136.3	-73.062	-41.998	6645	-0.665107851	-2.987227125	0.679004825
84494	5/26/2016 3:19	A	122445.4031	178409.0969	-73.161	-42.081	6220	-0.968712888	-2.987227125	0.806149143
84494	5/26/2016 4:54	B	3711378.052	1297815.948	-73.201	-42.094	5672	0.317477241	-2.987227125	0.847273433
84494	5/26/2016 7:26	B	4820972.85	1569287.15	-73.25	-42.13	9140	0.167745838	-2.987227125	0.755940511
84494	5/26/2016 8:37	B	6248822.564	1352509.436	-73.134	-42.099	4232	1.001904795	-2.987227125	1.001218447
84494	5/26/2016 11:52	A	8908016.183	945006.317	-72.83	-42.347	11682	-0.823889524	-1.93785784	0.872630152
84494	5/26/2016 11:56	B	2339711.496	385125.0036	-72.824	-42.35	298	-0.847874538	-1.831955724	0.876421056

84494	5/26/2016 13:02	B	8248756.264	2679680.236	-72.749	-42.379	3958	-0.823408132	-1.303541105	0.843228023
84494	5/26/2016 14:37	B	153824678.2	5914755.844	-72.661	-42.506	5668	-0.577847354	-1.285543566	0.449902363
84494	5/26/2016 19:45	B	34299268568	860477825.7	-72.917	-42.298	18497	-0.320178628	-2.987227125	0.855285648
84494	5/26/2016 23:08	B	1274519072	13540808	-73.018	-42.289	12179	-0.01337951	-2.55324836	0.819614898
84494	5/27/2016 0:32	B	1135500703	37612319.26	-73.088	-42.296	5000	-0.81226184	-1.877873024	0.754777412
84494	5/27/2016 1:18	B	416706070.9	22102353.6	-72.983	-42.33	2813	-0.536331974	-2.953382396	0.767799562
84494	5/27/2016 2:10	B	48159772.71	2703912.293	-72.928	-42.358	3093	-0.161619727	-2.987227125	0.866300594
84494	5/27/2016 2:56	B	3553848431	1131753.598	-72.838	-42.416	2753	-0.285456186	-2.866907985	1.047519603
84494	5/27/2016 4:36	B	30098815.96	52970982.54	-72.744	-42.458	6002	0.999369878	-2.506104533	0.848426077
84494	5/27/2016 5:33	A	18376877.56	283338.9424	-72.75	-42.398	3428	-0.467981868	-1.534647157	0.90225398
84494	5/27/2016 7:13	0	16343193.11	514926.8926	-72.818	-42.389	6011	0.273539929	-1.974712874	1.001145016
84494	5/27/2016 9:41	B	526775736	95869336.96	-72.781	-42.4	8866	-0.128222019	-1.773367271	0.932080765
84494	5/27/2016 10:07	B	7628333.913	2363162.087	-72.747	-42.42	1586	0.024920008	-1.92211591	0.904493283
84494	5/27/2016 11:22	B	1245281946	24596750.37	-72.726	-42.413	4482	-0.095779914	-1.600580303	0.863770237
84494	5/27/2016 11:45	B	16990854.19	317868.3051	-72.736	-42.415	1355	-0.121237036	-1.700388711	0.877195149
84494	5/27/2016 12:40	B	7000518.92	244233.5801	-72.726	-42.418	3297	0.053190191	-1.661285412	0.857517449

84494	5/27/2016 13:01	B	10551866.68	336555.818	-72.715	-42.418	1286	0.127456308	-1.621311541	0.836405336
84494	5/27/2016 13:34	B	54051971.82	9750318.678	-72.69	-42.413	1969	0	-1.366759033	0.793553644
84494	5/27/2016 14:21	B	5099949.644	431062.8561	-72.661	-42.415	2813	0	-1.168837561	0.746484205
84494	5/27/2016 15:14	B	5133438.739	527131.7606	-72.637	-42.412	3169	0	-1.032570429	0.706285191
84494	5/27/2016 20:58	A	80670.39633	1046638.604	-72.739	-42.492	20647	2.023378614	-2.599989578	0.639698786
84494	5/27/2016 22:23	B	137288	332794200.5	-72.782	-42.488	5095	1.67858864	-2.96515235	0.916686846
84494	5/27/2016 22:39	A	784588.0693	18005.93073	-72.803	-42.463	964	0.481232035	-2.957630587	1.079602813
84494	5/28/2016 0:01	B	13046553.86	724666.1406	-72.843	-42.467	4936	-0.397685925	-2.987227125	1.191329007
84494	5/28/2016 0:54	B	4756643.67	584068.8298	-72.877	-42.472	3163	-0.604248804	-2.987227125	1.202665734
84494	5/28/2016 2:39	A	1143982110	71115444.16	-72.937	-42.471	6342	-0.496653956	-2.987227125	1.136247717
84494	5/28/2016 3:29	B	15299453.17	510087.3306	-72.963	-42.474	2997	-0.521347185	-2.987227125	1.114942233
84494	5/28/2016 4:11	1	1012223.457	162637.5431	-72.997	-42.445	2529	-0.923008341	-2.673017462	1.157132839
84494	5/28/2016 5:20	A	9846976.099	402724118.4	-72.978	-42.587	4108	1.396702145	-2.974198302	0.86865712
84494	5/28/2016 7:01	A	1518088400	8163647.504	-73.014	-42.482	6086	-0.921077044	-2.804332601	1.160173689
84494	5/28/2016 8:40	B	35540752.98	23880435.02	-72.907	-42.54	5891	1.309656293	-2.60970241	1.022523959
84494	5/28/2016 9:52	B	6424294.828	1158427.672	-72.87	-42.527	4361	1.26932988	-2.514546732	1.068376828

84494	5/28/2016 10:47	B	155305149.8	22885444.16	-72.841	-42.494	3275	0.254282032	-2.905367672	1.141305057
84494	5/28/2016 11:30	B	5340255.277	583513.2232	-72.84	-42.508	2579	0.692426291	-2.856252	1.104670517
84494	5/28/2016 12:14	B	532442.7191	103692143.3	-72.906	-42.515	2665	0.247497379	-2.82950798	1.15959343
84494	5/28/2016 12:29	B	4360319.701	3081320.299	-72.877	-42.504	899	0.12802009	-2.806306531	1.178881645
84494	5/28/2016 13:55	B	119961647.5	11244078.98	-72.889	-42.563	5162	2.95693551	-2.170472775	0.803656489
84494	5/28/2016 14:48	B	9368453.524	1095896.976	-72.876	-42.572	3138	3.91329825	-2.008330574	0.655791077
84494	5/28/2016 15:40	B	16904847.59	2584706.911	-72.874	-42.575	3152	3.900829926	-1.971287679	0.652047144
84494	5/28/2016 19:17	A	1.93323E+11	2141227864	-72.509	-42.493	13030	0.028270262	-2.824870572	1.18600676
84494	5/28/2016 22:05	B	975344261.2	299689822.8	-72.944	-42.495	10081	-0.608599624	-2.977582482	1.200690498
84494	5/28/2016 22:15	B	486097895.2	64421743.27	-72.944	-42.498	587	-0.608599624	-2.978204717	1.200690498
84494	5/28/2016 23:37	B	192017890.1	22768028.36	-72.924	-42.464	4889	-0.941871215	-2.987227125	1.223291993
84494	5/28/2016 23:51	B	6285982.322	11901788.18	-72.959	-42.476	850	-0.919147314	-2.987227125	1.200251645
84494	5/29/2016 1:17	B	1565469.793	840492.2075	-72.971	-42.471	5165	-0.930160378	-2.987227125	1.19103447
84494	5/29/2016 1:34	B	1783308.643	908585.8574	-72.968	-42.476	1010	-0.9235362	-2.987227125	1.191491067
84494	5/29/2016 2:09	B	4333060.976	29290541.02	-72.941	-42.463	2154	-0.901154612	-2.987227125	1.212324682
84494	5/29/2016 3:08	B	13262007.65	16352661.35	-72.948	-42.435	3523	-0.587335008	-2.987227125	1.168451148

84494	5/29/2016 5:09	B	2193212.017	736312.9835	-72.958	-42.427	7218	-0.702702977	-2.987227125	0.450692996
84494	5/29/2016 6:44	0	1.58193E+11	3968575517	-72.887	-42.483	5749	-0.117487077	-2.987227125	0.299550309
84494	5/29/2016 8:28	B	1099706510	78596582.42	-72.861	-42.487	6214	0.043519124	-2.955612439	0.183137396
84494	5/29/2016 9:19	2	190609.5737	24615.42627	-72.823	-42.49	3059	-0.024282474	-2.970855234	0.094743189
84494	5/29/2016 9:43	B	4379079.447	1080243.053	-72.826	-42.492	1472	-0.018910064	-2.955612439	0.091240803
84494	5/29/2016 10:18	0	5695669.27	1838173.23	-72.822	-42.478	2078	-0.025737804	-2.987227125	0.114520019
84494	5/29/2016 11:04	B	137717349.7	41080312.78	-72.804	-42.477	2757	-0.089791054	-2.982294368	0.067065692
84494	5/29/2016 11:20	B	2458729774	1298012475	-72.782	-42.489	932	-0.225556925	-2.975383446	0.026839611
84494	5/29/2016 13:37	B	47981210	9515666.997	-72.779	-42.524	8260	-0.665482403	-2.609516941	-0.044549387
84494	5/29/2016 15:21	B	18706127.35	3388669.651	-72.757	-42.536	6221	-0.830578105	-1.981976785	-0.081608242
84494	5/29/2016 19:11	B	11802862.83	1442293.166	-72.733	-42.536	13801	-0.89903193	-1.652784774	-0.100346044
84494	5/29/2016 19:56	B	44794134.93	458307.0673	-72.745	-42.522	2729	-0.862508919	-2.120942334	-0.086860454
84494	5/29/2016 20:51	B	3762001.039	9473869.461	-72.665	-42.515	3257	-1.12094721	-1.224141371	-0.13596306
84494	5/29/2016 21:30	B	2864837.498	1970980.502	-72.635	-42.505	2375	-1.072903678	-1.06273208	-0.143369535
84494	5/29/2016 23:16	B	2859406.42	307794.5796	-72.805	-42.498	6329	-0.148770438	-2.970730572	0.044625735
84494	5/29/2016 23:42	B	2496165.554	347056.9455	-72.81	-42.494	1571	-0.087593645	-2.981728274	0.062096958

84494	5/30/2016 0:54	B	3269371.142	481253.8584	-72.757	-42.48	4324	-0.581460255	-2.890920088	-0.051298991
84494	5/30/2016 1:51	B	19254091.4	2773325.599	-72.835	-42.504	3407	0.091709315	-2.864983675	0.084584811
84494	5/30/2016 3:29	1	1781175.234	147049.7662	-72.966	-42.542	5901	0.697061993	-2.987227125	0.113114564
84494	5/30/2016 6:41	B	4210055783	2964537249	-72.893	-42.58	11486	-1.21035537	-2.468594964	-0.436348012
84494	5/30/2016 8:16	B	29066.70829	47429094.29	-72.917	-42.6	5718	-1.21115024	-2.766747703	-0.435363005
84494	5/30/2016 8:53	2	175202.8719	79629.62806	-72.896	-42.573	2245	-1.213594761	-2.238839557	-0.432934953
84494	5/30/2016 9:32	0	12536039.59	169778.9105	-72.871	-42.587	2343	-1.267948747	-2.013836915	-0.422784449
84494	5/30/2016 9:47	B	6268546.027	3098058.973	-72.91	-42.562	895	-1.15572661	-2.486864532	-0.442009606
84494	5/30/2016 10:34	B	410102.6508	16672595.85	-72.904	-42.561	2801	-1.168306764	-2.404335032	-0.437609225
84494	5/30/2016 11:07	B	14505291.93	29578096.57	-72.884	-42.541	2011	-1.113427791	-2.339247299	-0.423866304
84494	5/30/2016 11:25	B	1071956.656	483211.8441	-72.883	-42.538	1063	-1.096290859	-2.370271543	-0.422546158
84494	5/30/2016 11:38	B	237990.3663	616750.6337	-72.884	-42.536	748	-1.094754366	-2.404922112	-0.421928773
84494	5/30/2016 14:06	B	26586571.39	2823305.106	-72.876	-42.515	8923	-0.995068975	-2.71798079	-0.410033929
84494	5/30/2016 14:56	B	7077753.443	538131.5572	-72.871	-42.514	3004	-0.99914055	-2.723863816	-0.403445037
84494	5/30/2016 19:00	B	69715641.99	2668156.51	-72.955	-42.516	14594	-0.875848142	-2.987227125	-0.492453294
84494	5/30/2016 20:35	B	51523249.24	15998263.26	-73.087	-42.464	5749	-0.611879424	-1.52070959	-0.636722328

84494	5/30/2016 21:04	B	1781782.857	6974267.143	-73.04	-42.46	1704	-0.676357605	-1.913809763	-0.584192122
84494	5/30/2016 21:46	B	36538666.52	482274.483	-73.07	-42.535	2529	-0.591344862	-2.248192545	-0.6210235
84494	5/30/2016 22:47	A	45026.64076	9521.859242	-73.144	-42.475	3647	-0.677258998	-1.591461421	-0.678073255
84494	5/30/2016 23:36	B	1167392	264264.5	-73.175	-42.507	2938	-0.939984769	-1.878590209	-0.706253084
84494	5/31/2016 0:30	B	5795645.854	1521404.146	-73.19	-42.518	3249	-0.952107806	-1.811445716	-0.720152572
84494	5/31/2016 1:11	B	20803506.84	7666257.162	-73.18	-42.521	2463	-1.044065232	-1.810363073	-0.718972389
84494	5/31/2016 2:26	A	2024820.968	182021.5324	-72.963	-42.505	4504	-0.79731116	-2.987227125	-0.503702263
84494	5/31/2016 3:16	2	425837.7419	9926.758085	-72.943	-42.544	2980	-1.056573005	-2.920448865	-0.46806075
84494	5/31/2016 4:46	B	6090861.639	1664956.361	-72.857	-42.551	5414	-1.183231074	-2.092862622	-0.480465077
84494	5/31/2016 6:27	0	8751194.757	1019033.243	-72.959	-42.55	6072	-0.931225644	-2.987227125	-0.54557552
84494	5/31/2016 8:03	2	109626.4086	450306.0914	-72.944	-42.531	5780	-0.831206186	-2.957259843	-0.553479485
84494	5/31/2016 9:21	0	8468839.823	1284730.177	-72.915	-42.482	4654	-0.791779945	-2.987227125	-0.541233439
84494	5/31/2016 10:08	B	124108410.6	13678594.39	-72.895	-42.459	2847	-0.800639426	-2.987227125	-0.500699369
84494	5/31/2016 11:17	B	221051967.7	33725482.31	-72.876	-42.438	4125	-0.796946169	-2.982884049	-0.460548222
84494	5/31/2016 11:51	B	298461792.6	133311807.4	-72.898	-42.436	2020	-0.735288798	-2.985200356	-0.477113734
84494	5/31/2016 12:53	B	315509529.4	133483192.6	-72.867	-42.418	3723	-0.793338285	-2.962455509	-0.42771181

84494	5/31/2016 13:45	B	389744016.8	127147455.2	-72.827	-42.399	3115	-0.888078009	-2.464206561	-0.38244781
84494	5/31/2016 18:44	B	1547698408	208482214.3	-73.42	-42.466	17968	-0.738561764	-1.018053598	-0.848600759
84494	5/31/2016 20:32	B	36467719.16	48728625.84	-73.219	-42.39	6439	-1.205227011	-0.925871631	-0.639269864
84494	5/31/2016 22:04	B	723941979.2	62135030.79	-73.298	-42.455	5528	-0.42742916	-1.042324413	-0.745133876
84494	5/31/2016 23:19	B	1252480162	8244207.74	-73.305	-42.46	4540	-0.343760524	-1.101980003	-0.753605683
84494	5/31/2016 23:55	B	295679097.8	28421014.24	-73.224	-42.467	2150	-0.456855125	-1.482068071	-0.702364807
84494	6/1/2016 0:05	B	251621258.9	39162543.57	-73.221	-42.455	604	-0.714824621	-1.356808822	-0.677598403
84494	6/1/2016 0:57	B	30464643.75	206519517.3	-73.137	-42.444	3083	-0.438931981	-1.324798786	-0.729411713
84494	6/1/2016 1:15	B	3460557.766	15478842.23	-73.137	-42.443	1118	-0.450969242	-1.304206944	-0.725466432
84494	6/1/2016 2:46	B	34092843.26	20814357.74	-73.099	-42.408	5461	-0.4360147	-1.596623459	-0.653130467
84494	6/1/2016 3:43	B	16347896968	20039449.97	-73.092	-42.459	3425	-0.551709136	-1.612183117	-0.748803857
84494	6/1/2016 4:35	B	12480622.15	4912922.849	-73.031	-42.477	3116	-0.942438003	-2.303074121	-0.65510736
84494	6/1/2016 6:19	A	8515294.994	21748382.01	-73.136	-42.448	6247	-0.764529908	-1.349836493	-0.700408824
84494	6/1/2016 8:00	B	17521767.68	2192230.821	-73.126	-42.425	6002	-0.718559259	-1.350853575	-0.660626257
84494	6/1/2016 9:39	B	16187278.07	2237465.935	-73.115	-42.423	5948	-0.700337301	-1.408827703	-0.651716779
84494	6/1/2016 10:27	B	15180538.77	3298959.226	-73.108	-42.422	2886	-0.686859984	-1.470681375	-0.643634903

84494	6/1/2016 10:50	B	21962854.35	5161398.646	-73.105	-42.427	1423	-0.692356875	-1.461159522	-0.652621939
84494	6/1/2016 12:02	B	6742122.503	23563962.5	-72.939	-42.402	4314	-0.791139291	-2.987227125	-0.489800252
84494	6/1/2016 12:37	B	5578320.073	2780816.927	-72.906	-42.427	2105	-0.930436481	-2.985130467	-0.494740398
84494	6/1/2016 13:24	B	24590404.53	3131712.473	-72.904	-42.433	2777	-0.950856925	-2.987227125	-0.502793725
84494	6/1/2016 14:14	B	420060246.7	55650046.27	-72.928	-42.477	3017	-1.003574627	-2.987227125	-0.549612834
84494	6/1/2016 18:32	A	1805060361	45844395.75	-72.891	-42.53	15461	-1.153307176	-2.634389947	-0.534944413
84494	6/1/2016 20:15	B	647059970.4	81179390.06	-72.99	-42.515	6194	-0.951000097	-2.96144382	-0.611661694
84494	6/1/2016 21:39	B	425113257.6	35625243.39	-73.004	-42.466	5014	-0.869649516	-2.652946018	-0.624822975
84494	6/1/2016 23:07	B	2788876426	502542329.9	-73.128	-42.385	5312	-0.676363121	-1.553973803	-0.594554621
84494	6/1/2016 23:19	B	139103381.2	129723668.8	-73.129	-42.387	719	-0.694508299	-1.443871969	-0.59683296
84494	6/1/2016 23:36	A	14254066.33	24914838.67	-73.096	-42.348	1013	-0.645749869	-1.932583154	-0.512286766
84494	6/2/2016 1:14	B	266388338.8	197697953.2	-73.127	-42.32	5903	-0.695660831	-1.4355205	-0.492417813
84494	6/2/2016 2:31	B	11923191767	450607729.6	-73.098	-42.286	4596	-0.534512469	-1.827690966	-0.399966677
84494	6/2/2016 3:21	B	7673191.115	512863.3847	-73.054	-42.261	3031	-0.428723389	-2.415976352	-0.313143111
84494	6/2/2016 4:13	B	466777.4736	37855211.53	-72.939	-42.226	3079	-0.658096798	-2.987227125	-0.53812198
84494	6/2/2016 5:59	A	1015418148	11341337.12	-72.753	-42.24	6353	-0.873899635	-2.748869032	-0.7272633

84494	6/2/2016 8:56	B	1280412.558	3008799504	-72.895	-42.188	10648	-0.633401437	-2.987227125	-0.534306372
84494	6/2/2016 9:19	0	384800.564	9438524.436	-72.896	-42.174	1358	-0.628777382	-2.987227125	-0.514393223
84494	6/2/2016 10:39	B	11341846093	428552377.4	-72.878	-42.207	4807	-0.650254357	-2.987227125	-0.564308116
84494	6/2/2016 12:11	B	54463207.89	119638129.1	-72.897	-42.222	5531	-0.664983008	-2.987227125	-0.577546771
84494	6/2/2016 13:07	B	7443099.064	6191283.436	-72.898	-42.22	3349	-0.663485973	-2.987227125	-0.57523453
84494	6/2/2016 14:51	B	6167997.638	1154144.862	-72.84	-42.208	6230	-0.69288978	-2.987227125	-0.628420134
84494	6/2/2016 18:19	B	14243529346	1005226418	-72.883	-42.251	12530	-0.769360595	-2.987227125	-0.663078533
84494	6/2/2016 21:29	B	10063499288	311726844.6	-72.798	-42.377	11396	-1.065322402	-2.01278384	-0.797496378
84494	6/3/2016 0:33	B	61260.77777	19646424.22	-72.886	-42.341	11043	-0.897972139	-2.840074853	-0.730652916
84494	6/3/2016 0:51	B	422014.5525	80839.94753	-72.884	-42.355	1059	-0.928097109	-2.766216817	-0.745722685
84494	6/3/2016 2:11	1	1188530.178	34151.82237	-72.857	-42.313	4803	-0.880628128	-2.706200745	-0.724086034
84494	6/3/2016 3:06	B	11506881278	519911753.9	-72.842	-42.297	3292	-0.845720129	-2.873794499	-0.705901687
84494	6/3/2016 7:36	A	1849153.62	677728.8804	-72.921	-42.368	16178	-0.031114583	-2.987227125	-0.817537393
84494	6/3/2016 9:00	B	13206625.44	4185768.563	-72.912	-42.363	5031	-0.028626868	-2.930001241	-0.84396205
84494	6/3/2016 9:19	B	2222717.665	11033702.33	-72.844	-42.361	1192	-0.548207967	-2.166461151	-1.002909124
84494	6/3/2016 10:27	0	300411506.2	18706615.8	-72.918	-42.282	4053	-0.039827241	-2.987227125	-0.711554717

84494	6/3/2016 10:33	1	1068410.097	45012.40311	-72.916	-42.284	366	-0.045681202	-2.987227125	-0.714153707
84494	6/3/2016 11:02	B	14616716.09	419638.4084	-72.873	-42.33	1736	-0.180889323	-2.872775358	-0.899468209
84494	6/3/2016 12:05	B	117170353.6	21283170.36	-72.934	-42.245	3793	0.034487083	-2.987227125	-0.596315617
84494	6/3/2016 12:39	B	210135205.4	158794391.6	-72.972	-42.28	1999	-0.027109231	-2.987227125	-0.547682592
84494	6/3/2016 13:36	B	433460973.7	23116566.27	-73.006	-42.256	3438	-0.027713765	-2.858592173	-0.409188732
84494	6/3/2016 14:28	B	1090736718	16450067.17	-73.016	-42.283	3157	-0.058134248	-2.763668451	-0.477352682
84494	6/3/2016 15:12	0	16433418.82	1151371574	-72.887	-42.322	2607	-0.13260639	-2.971440674	-0.840991284
84494	6/3/2016 18:10	B	5101432395	6738054789	-72.857	-42.286	10658	-0.113672258	-2.987227125	-0.851537843
84494	6/3/2016 19:47	0	805157428.6	75373295.4	-72.911	-42.29	5878	-0.062347242	-2.987227125	-0.741747641
84494	6/3/2016 20:43	B	153677394.4	28344716.11	-72.907	-42.296	3321	-0.078563259	-2.987227125	-0.756895578
84494	6/3/2016 21:29	B	6359386529	395446425.4	-72.92	-42.314	2756	-0.092352011	-2.987227125	-0.774834776
84494	6/3/2016 22:16	B	462905940.5	48871944.52	-72.895	-42.281	2847	-0.060793317	-2.987227125	-0.766264607
84494	6/3/2016 22:44	B	4956563.198	762153.302	-72.919	-42.306	1697	-0.081019121	-2.987227125	-0.744671934
84494	6/4/2016 0:01	B	5182924.262	667534.2383	-72.921	-42.314	4601	-0.08333114	-2.987227125	-0.747072966
84494	6/4/2016 0:23	B	3590545.929	1005294.571	-72.92	-42.313	1333	-0.082416652	-2.987227125	-0.74911565
84494	6/4/2016 1:47	B	2728829.703	1332388.297	-72.912	-42.324	5005	-0.088908948	-2.987227125	-0.783822751

84494	6/4/2016 2:41	0	57341809.07	2657855.433	-72.981	-42.357	3265	-0.206984489	-2.987227125	-0.63489779
84494	6/4/2016 3:26	1	1379911.514	371470.9862	-72.938	-42.35	2667	-0.076741103	-2.987227125	-0.748193934
84494	6/4/2016 7:25	1	6198331.488	306251.012	-72.951	-42.438	14350	1.113811373	-2.987227125	-0.8174638
84494	6/4/2016 8:28	B	248386991.5	60216829.52	-72.951	-42.441	3764	0.987499667	-2.987227125	-0.810943629
84494	6/4/2016 8:37	B	253005079	80989789.97	-72.949	-42.456	540	0.424394513	-2.987227125	-0.772327311
84494	6/4/2016 10:28	1	1868244.5	180000	-72.977	-42.544	6668	-0.216754743	-2.987227125	-0.664087454
84494	6/4/2016 11:37	B	272728012.5	2115624.5	-73.017	-42.568	4159	-0.903775506	-2.937299379	-0.611396665
84494	6/4/2016 11:47	B	153587127.2	3332841.322	-73.024	-42.571	590	-0.863142058	-2.884895661	-0.610715935
84494	6/4/2016 12:06	B	2110855065	95736237.77	-73.047	-42.531	1162	-0.786332371	-2.587367361	-0.560305764
84494	6/4/2016 13:14	B	3920000	192200	-73.089	-42.534	4090	-0.603498034	-1.981878721	-0.509570711
84494	6/4/2016 14:03	B	13791752	341138	-73.092	-42.536	2892	-0.587414385	-1.98783351	-0.512296188
84494	6/4/2016 18:00	B	25979045.8	883879.1977	-73.158	-42.505	14263	-0.432271582	-1.811541967	-0.362491169
84494	6/4/2016 19:46	B	58525380.5	1026744.5	-72.986	-42.486	6317	-0.274033457	-2.907012399	-0.660369662
84494	6/4/2016 21:26	B	573408884.1	53609435.92	-73.048	-42.526	6011	-0.774853235	-2.493270272	-0.554516635
84494	6/4/2016 22:22	A	304952.9735	68080.02647	-73.014	-42.536	3366	-0.767005134	-2.896633897	-0.599141119
84494	6/4/2016 23:30	A	246247746.5	1914385.962	-73.087	-42.57	4089	-0.458060794	-2.448617499	-0.575087004

84494	6/5/2016 0:01	B	2029473.747	652759.2525	-73.132	-42.562	1876	-0.276680314	-2.068620955	-0.513589575
84494	6/5/2016 1:26	B	1566326659	6576516037	-72.997	-42.565	5112	-0.894614483	-2.938116393	-0.602141836
84494	6/5/2016 2:23	A	286139.1249	69881.87509	-73.039	-42.529	3389	-0.784061285	-2.579278708	-0.56349539
84494	6/5/2016 3:11	0	13640979.5	4706614.5	-73.059	-42.534	2854	-0.748667667	-2.392085348	-0.544867768
84494	6/5/2016 4:00	B	55598410.2	17257833.8	-73.076	-42.533	2967	-0.844055477	-2.093663383	-0.484804374
84494	6/5/2016 4:47	B	265106325.9	7128787.138	-73.083	-42.544	2801	-0.869554512	-2.228447514	-0.488424883
84494	6/5/2016 5:30	0	1336139.384	1944945.616	-73.025	-42.476	2587	-0.229818916	-2.38197474	-0.58381253
84494	6/5/2016 7:12	1	43177336.59	874441.4057	-73.056	-42.458	6121	-0.334477446	-1.751758644	-0.486331068
84494	6/5/2016 8:05	B	161126853.9	212669291.1	-73.071	-42.439	3220	-0.292663038	-1.538245074	-0.442056248
84494	6/5/2016 8:49	B	127396199.6	164668768.4	-73.061	-42.453	2621	-0.328837259	-1.630947466	-0.477025755
84494	6/5/2016 9:42	A	4307926.57	584125.9298	-73.064	-42.562	3188	-0.981300104	-2.607122903	-0.512978502
84494	6/5/2016 10:02	B	1570302285	112752389.3	-73.083	-42.573	1186	-0.834707334	-2.503141454	-0.518859241
84494	6/5/2016 11:21	B	28515402.17	3503380.332	-73.087	-42.599	4764	-0.533679882	-2.33408693	-0.561407406
84494	6/5/2016 11:33	B	12584546.38	2746688.12	-73.101	-42.586	665	-0.566651892	-2.35706698	-0.53524963
84494	6/5/2016 11:44	B	4915489.121	1018311.879	-73.103	-42.587	673	-0.550224282	-2.366759858	-0.534212331
84494	6/5/2016 12:51	A	438512957	185195111	-72.98	-42.585	4061	-0.181146543	-2.956343989	-0.474884701

84494	6/5/2016 14:32	B	1889313338	71481386.77	-72.989	-42.534	6014	0.556581632	-2.987227125	-0.648999392
84494	6/5/2016 19:36	B	492777272.4	225572788.1	-72.656	-42.657	18278	0	-0.684584378	0
84494	6/5/2016 21:16	B	33757587.95	6178428.047	-72.849	-42.544	5997	1.313881977	-2.205661502	-1.236242994
84494	6/5/2016 22:00	B	30769641.9	6417807.102	-72.861	-42.529	2626	0.94443817	-2.421947937	-1.259901458
84494	6/5/2016 22:20	B	1037580.166	78558690.33	-72.924	-42.529	1217	1.438637567	-2.835569099	-0.962821167
84494	6/5/2016 22:52	B	7017601.848	5791283.152	-72.945	-42.529	1920	1.359611927	-2.957259843	-0.838653539
84494	6/5/2016 23:38	B	5774577.932	515890.0682	-72.961	-42.536	2747	1.356158142	-2.987227125	-0.727961077
84494	6/6/2016 0:07	B	5102723.879	399481.1205	-72.956	-42.535	1731	1.356158142	-2.987227125	-0.727961077
84494	6/6/2016 1:07	B	5709200.363	483644.6372	-72.976	-42.522	3583	0.534004149	-2.987227125	-0.691371896
84494	6/6/2016 2:51	0	1800733710	35572900.41	-73.123	-42.502	6287	-0.59288914	-1.622138944	-0.39649529
84494	6/6/2016 5:16	B	31505358.09	3607746.915	-73.239	-42.483	8645	-0.505507482	-1.60573836	-0.18221222
84494	6/6/2016 8:38	A	17387587.71	12361973.29	-72.828	-42.556	12147	-0.54945893	-2.010801354	-0.890497533
84494	6/6/2016 9:18	B	537564539.9	194141780.1	-72.812	-42.576	2400	0	-1.758923783	-0.906317621
84494	6/6/2016 9:51	B	12767974.28	2790578.721	-72.795	-42.573	1992	0	-1.738301066	0
84494	6/6/2016 10:58	B	3632957.576	549783.4242	-72.757	-42.59	4001	0	-1.277140895	0
84494	6/6/2016 12:44	B	23903761.72	5732183.281	-72.679	-42.569	6360	0	-0.975978358	0

84494	6/6/2016 13:22	B	4559405.644	427250.8565	-72.699	-42.542	2305	0	-1.283758993	-1.106341148
84494	6/6/2016 19:13	B	13680704.47	2798851.534	-72.608	-42.51	21022	0	-0.909641966	-1.160591076
84494	6/6/2016 21:33	B	19129915.38	333454709.6	-72.891	-42.594	8416	-0.037712618	-2.340056807	-0.766614208
84494	6/6/2016 22:21	B	1312241.377	1725328.623	-72.943	-42.607	2882	-0.438701807	-2.962505363	-0.656086256
84494	6/6/2016 23:08	B	15226505.14	1087639.862	-72.984	-42.61	2843	-0.610816665	-2.820314134	-0.65659826
84494	6/6/2016 23:47	B	16627710.75	1279611.247	-73.01	-42.626	2325	-0.396217101	-2.331877386	-0.694715815
84494	6/7/2016 0:50	B	2225098.701	638073.799	-72.947	-42.621	3749	-0.183512427	-2.96073531	-0.688378911
84494	6/7/2016 2:26	A	296226929.4	21271569.13	-73.008	-42.502	5811	-0.549872959	-2.850308886	-0.545232211
84494	6/7/2016 3:17	B	13832783.92	2511505.075	-73.049	-42.489	3021	-0.511642871	-2.049876031	-0.459496656
84494	6/7/2016 4:07	B	10798657.38	2403259.124	-73.085	-42.483	3028	-0.596182836	-1.613606987	-0.668254397
84494	6/7/2016 6:49	A	24522989.37	522523.1251	-72.827	-42.391	9713	-0.845509477	-2.178556408	-0.605275648
84494	6/7/2016 8:25	0	1150502238	276448544.4	-72.888	-42.375	5765	-0.770774441	-2.812866912	-0.558032133
84494	6/7/2016 9:00	B	455785215.5	116210169.5	-72.884	-42.362	2065	-0.77075923	-2.762023503	-0.543236348
84494	6/7/2016 9:41	A	42946200.82	6740143.679	-72.899	-42.396	2463	-0.75848033	-2.955676108	-0.582550893
84494	6/7/2016 10:32	A	114806.7943	6187.205719	-72.832	-42.399	3095	-0.838285102	-2.408535193	-0.614196078
84494	6/7/2016 11:18	A	845799.2869	687813.7131	-72.783	-42.388	2721	-0.882288243	-1.583111145	-0.623278041

84494	6/7/2016 12:16	B	114665170.3	114665170.3	-72.793	-42.352	3507	-0.878007261	-1.402526604	-0.575610572
84494	6/7/2016 13:58	B	8214144.353	3391674.147	-72.752	-42.322	6092	0	-1.242876319	0
84494	6/7/2016 15:29	B	21807204.63	575822303.9	-72.897	-42.379	5492	-0.756337622	-2.892171567	-0.553414965
84494	6/7/2016 19:05	A	795068.5742	3598792.426	-72.85	-42.319	12966	-0.798490222	-2.599189408	-0.499881552
84494	6/7/2016 20:44	A	111502.8779	5051.122066	-72.881	-42.317	5931	-0.763039781	-2.95452662	-0.477531839
84494	6/7/2016 23:36	1	4008742.973	142310.027	-72.952	-42.423	10339	-0.704979725	-2.987227125	-0.60574357
84494	6/8/2016 0:30	B	17495387.6	3910152.901	-72.976	-42.457	3230	-0.702799754	-2.954815591	-0.654451932
84494	6/8/2016 2:04	A	549489.9107	1217026.089	-72.877	-42.479	5654	-0.828887355	-2.987227125	-0.711413206
84494	6/8/2016 3:05	B	43553179.26	13572209.74	-72.887	-42.489	3627	-0.829129459	-2.950877046	-0.723919115
84494	6/8/2016 3:42	B	33785449.13	10911421.37	-72.884	-42.489	2245	-0.830204457	-2.941095402	-0.722899058
84494	6/8/2016 4:56	B	41110309.64	10743022.86	-72.882	-42.484	4412	0.229643696	-2.970248868	0.100303417
84494	6/8/2016 6:38	B	2866451448	31666044.12	-72.864	-42.38	6110	-0.658652287	-2.40085655	0.267561837
84494	6/8/2016 8:14	A	138873701.6	6302544.949	-72.73	-42.339	5763	0	-1.11396151	0
84494	6/8/2016 8:31	B	19807969.81	1134848.186	-72.732	-42.339	1066	0	-1.114059769	0
84494	6/8/2016 9:27	0	1783544.871	1968628.129	-72.867	-42.372	3320	-0.729851251	-2.62605568	0.295229542
84494	6/8/2016 10:04	A	3061670.654	9872269.346	-72.843	-42.362	2253	-0.50547475	-2.166461151	0.244668026

84494	6/8/2016 10:08	A	1529380.607	40655.39313	-72.848	-42.356	209	-0.48768252	-2.256046809	0.244002929
84494	6/8/2016 11:11	A	106315.438	36485.062	-72.862	-42.372	3762	-0.7029325	-2.581552514	0.288963818
84494	6/8/2016 11:44	B	50036078.61	66211283.89	-72.868	-42.359	1980	-0.628431051	-2.557333645	0.288604448
84494	6/8/2016 11:53	B	1344889.424	1535760.576	-72.867	-42.358	576	-0.609226362	-2.523535609	0.28595356
84494	6/8/2016 13:37	B	2290430.178	1078409.822	-72.894	-42.356	6228	-0.76350325	-2.840074853	0.330888762
84494	6/8/2016 14:27	B	3014879.417	1316419.083	-72.903	-42.357	3025	-0.81919665	-2.872775358	0.344909083
84494	6/8/2016 21:17	B	18114063.39	2149061.109	-73.003	-42.304	24562	-0.951132529	-2.778416039	0.467135328
84494	6/8/2016 21:45	B	27893746.3	1034472.195	-72.969	-42.248	1667	-0.508179632	-2.987227125	0.450511147
84494	6/8/2016 22:59	B	300855261.9	4540438.094	-72.827	-42.17	4455	0.120061766	-2.987227125	0.175557707
84494	6/9/2016 1:12	B	7793352	460800	-72.837	-42.16	8003	0.146892541	-2.987227125	0.211737532
84494	6/9/2016 1:46	B	9419878.386	454628.1137	-72.839	-42.174	2050	0.106534046	-2.987227125	0.197332897
84494	6/9/2016 2:43	B	8792712.353	699808.1471	-72.871	-42.194	3403	-0.006678594	-2.987227125	0.24690554
84494	6/9/2016 3:31	B	358582144.7	336321.3496	-72.889	-42.283	2881	-0.340824677	-2.987227125	0.262306088
84494	6/9/2016 9:10	B	1066909568	5966014.357	-72.975	-42.6	20322	1.238600379	-2.972337042	-0.273376453
84494	6/9/2016 11:10	A	1362110.346	112694.6544	-72.933	-42.702	7234	2.633310873	-1.912857106	-0.591309291
84494	6/9/2016 11:23	B	1402585.269	1084947.231	-72.906	-42.721	766	1.892833654	-1.840573334	-0.836169577

84494	6/9/2016 11:31	B	1478404.95	1193031.55	-72.905	-42.726	487	1.800552557	-1.783344375	-0.871098799
84494	6/9/2016 12:49	B	73770324.3	2347830.203	-72.876	-42.683	4655	1.018044445	-2.611174842	-0.670793103
84494	6/9/2016 13:05	B	43705972.66	2288352.339	-72.888	-42.686	968	1.259333517	-2.594734151	-0.644942723
84494	6/9/2016 18:43	B	176657266.6	18451689.93	-72.859	-42.617	20255	-0.646700899	-1.993393245	-0.542651474
84494	6/9/2016 20:20	B	264940562.5	146243242.5	-72.834	-42.645	5877	-0.25916283	-1.821401247	-0.58030809
84494	6/9/2016 20:54	B	32843806.15	23585329.85	-72.82	-42.632	1993	0	-1.60823671	-0.551597803
84494	6/9/2016 21:37	B	29857567.91	7179252.589	-72.865	-42.541	2592	1.191901119	-2.219628272	-0.48448306
84494	6/9/2016 22:08	B	86572152	28919592	-72.894	-42.519	1881	2.645199811	-2.744070603	-0.384637257
84494	6/9/2016 22:27	B	206472032.9	9332420.146	-72.877	-42.537	1126	1.450726449	-2.394741512	-0.492945132
84494	6/9/2016 23:10	0	9757994.705	11970327.8	-72.86	-42.493	2579	3.83223678	-2.888995781	-0.052353261
84494	6/9/2016 23:42	1	156449257.9	5918630.063	-72.867	-42.537	1944	1.554463729	-2.331884383	-0.471305357
84494	6/10/2016 1:16	B	25273901.75	3791004.251	-72.926	-42.525	5643	2.212882054	-2.879144462	-0.380019744
84494	6/10/2016 1:23	B	2998569.794	747631.2057	-72.928	-42.528	391	2.181523213	-2.882871451	-0.386008278
84494	6/10/2016 3:06	A	9595876.045	897268.9546	-72.955	-42.481	6205	1.510149531	-2.987227125	0.071078971
84494	6/10/2016 4:48	B	6407703.731	52527578.77	-72.782	-42.471	6085	3.934137717	-2.917386112	-0.036205487
84494	6/10/2016 6:15	B	105024802.3	32521197.7	-72.725	-42.43	5260	4.067421239	-1.812511484	-0.296128024

84494	6/10/2016 7:56	B	19488980.65	3627983.346	-72.724	-42.354	6011	0	-1.058255643	0
84494	6/10/2016 9:21	1	957917.1074	20821.39259	-72.944	-42.303	5130	2.149716826	-2.987227125	0.281093513
84494	6/10/2016 10:42	B	1910367274	72194656.74	-72.994	-42.307	4829	0.380340671	-2.987227125	0.447692034
84494	6/10/2016 11:03	B	10004519.49	7217920.506	-72.963	-42.285	1280	1.198440068	-2.987227125	0.330601492
84494	6/10/2016 12:19	B	4455189.761	574068.2385	-72.953	-42.299	4571	1.707471687	-2.987227125	0.319937938
84494	6/10/2016 14:24	B	7422436.839	808188.1606	-72.982	-42.275	7492	0.523560646	-2.960547283	0.380107556
84494	6/10/2016 20:16	B	27471216.26	1380762.244	-73.079	-42.185	21145	-0.127907538	-2.987227125	0.650614532
84494	6/10/2016 21:31	B	27204930.96	3080039.539	-73.013	-42.192	4465	-0.083833438	-2.987227125	0.573248038
84494	6/10/2016 23:04	B	61566268.85	6654573.15	-72.878	-42.148	5615	3.69968254	-2.987227125	0.573924347
84494	6/10/2016 23:18	0	48192294646	2568735127	-72.872	-42.122	800	3.083874319	-2.987227125	0.670280803
84494	6/11/2016 1:55	B	383423432	56042284.5	-72.898	-42.087	9410	2.340512822	-2.987227125	0.655964223
84494	6/11/2016 2:41	0	584347272.7	22102729.75	-72.873	-42.118	2813	3.082693498	-2.987227125	0.583236245
84494	6/11/2016 7:36	B	645019971.8	216109922.7	-72.841	-42.093	17682	1.647181073	-2.987227125	-0.298844695
84494	6/11/2016 8:52	B	45220341802	2811668302	-72.873	-42.119	4560	1.640360899	-2.987227125	-0.039349433
84494	6/11/2016 8:54	B	209173162.8	15028399.69	-72.877	-42.121	81	1.650981572	-2.987227125	-0.034130247
84494	6/11/2016 10:05	B	6866982.808	387602.1918	-72.952	-42.147	4289	1.396988515	-2.987227125	-0.08810018

84494	6/11/2016 10:30	B	9826835.843	334974.1572	-72.998	-42.182	1529	1.928412548	-2.987227125	-0.083760978
84494	6/11/2016 11:44	B	46092265.03	19441811.47	-72.941	-42.226	4429	2.568496786	-2.987227125	-0.304960022
84494	6/11/2016 13:25	B	5619127.611	1837197.389	-72.93	-42.246	6054	2.995021036	-2.987227125	-0.435591657
84494	6/11/2016 14:12	B	250976420.7	1287124.333	-72.887	-42.223	2782	3.153924264	-2.987227125	-0.654190157
84494	6/11/2016 18:19	B	130354906.9	98561575.58	-72.916	-42.129	14864	0.537200478	-2.987227125	0.115945069
84494	6/11/2016 19:58	B	1149889489	100984392.1	-72.816	-41.883	5938	-0.260571004	-1.848377963	-0.342376061
84494	6/11/2016 22:48	B	18283403044	976184176.3	-72.868	-41.877	10183	-0.257517551	-2.673158443	-0.119696541
84494	6/12/2016 0:29	B	13389095.21	4330537.287	-73.036	-41.774	6067	-0.682735207	-1.171662739	0.031153643
84494	6/12/2016 1:41	B	28646186.31	9054166.195	-73.065	-41.72	4339	-0.846369887	-0.932587713	0.097163321
84494	6/12/2016 2:31	B	71953895.77	223084618.2	-73.065	-41.744	2956	-0.72745851	-0.932783715	0.09608066
84494	6/12/2016 4:00	B	6043922.271	13790146.73	-73.091	-41.698	5370	0	-0.841293354	0.127203431
84494	6/12/2016 10:07	A	733925697.4	18591184.55	-73.432	-42.129	21999	-0.739616764	-1.149347818	0.362276641
84494	6/12/2016 11:46	B	2032868850	34792091.15	-73.457	-42.206	5977	-0.076020124	-0.94055721	0.196183324
84494	6/12/2016 13:46	B	4884503620	151953429.2	-73.334	-42.214	7171	-0.359863799	-2.111243625	0.43077045
84494	6/12/2016 15:31	A	660705920.4	157876600.6	-73.052	-42.213	6280	0.717270425	-2.987227125	0.118512069
84494	6/12/2016 22:22	0	831555.7945	16306024.71	-73.266	-42.171	24678	-0.204637491	-2.820372418	0.528238631

84494	6/12/2016 22:40	B	2169537851	150883677.6	-73.239	-42.196	1081	-0.568609587	-2.688677204	0.471236753
84494	6/13/2016 0:17	A	24737.04923	349501.4508	-73.272	-42.182	5848	-0.423248177	-2.681326829	0.500124354
84494	6/13/2016 5:34	B	22659214.77	5087341.733	-73.32	-42.188	18986	-0.411893462	-2.155602677	0.460741469
84494	6/13/2016 7:24	B	22832756149	2134206804	-73.357	-42.219	6582	-0.253139633	-2.759895509	0.389117366
84494	6/13/2016 8:28	B	20609577.17	33058011.83	-73.169	-42.286	3868	-0.455176497	-1.724989304	0.192041752
84494	6/13/2016 9:42	B	8026624796	362656573.6	-73.087	-42.246	4436	-0.327254742	-2.69712631	0.270149182
84494	6/13/2016 10:45	B	15049265.1	78852299.9	-73.244	-42.22	3802	-0.257297187	-2.848185651	0.396313073
84494	6/13/2016 11:26	B	283076302.2	498288833.8	-73.258	-42.2	2445	-0.318473002	-2.620545689	0.450480772
84494	6/13/2016 11:52	B	209148575.7	13457486.79	-73.294	-42.199	1531	-0.347067166	-2.192560713	0.448160957
84494	6/13/2016 13:24	B	3269050.558	461569.9425	-73.383	-42.189	5542	-0.486256987	-1.449372376	0.411862265
84494	6/13/2016 15:09	A	1593503712	414122393.2	-73.119	-42.187	6324	-0.173316814	-2.987227125	0.400892434
84494	6/13/2016 19:36	B	1189160188	31607264.57	-73.11	-42.077	16016	-0.738311726	-2.987227125	0.514632095
84494	6/13/2016 21:17	B	43094762.6	11618291.9	-73.226	-42.026	6023	-0.949150565	-2.987227125	0.582882552
84494	6/13/2016 21:58	B	142933241.8	314137464.2	-73.182	-42.041	2488	-0.849415004	-2.987227125	0.568085698
84494	6/13/2016 23:36	B	13690365058	4210410398	-73.25	-42.12	5881	-1.061732422	-2.987227125	0.62054982
84494	6/13/2016 23:48	B	2761050910	853261096.8	-73.252	-42.121	690	-0.99784229	-2.987227125	0.615719202

84494	6/14/2016 1:44	B	379532814.7	35716397.85	-73.086	-42.081	6974	-0.616534543	-2.987227125	0.461054447
84494	6/14/2016 2:35	B	230190773	15870499.49	-73.053	-42.095	3085	-0.470975895	-2.987227125	0.398191852
84494	6/14/2016 3:25	B	93214373.44	329637867.1	-73.187	-42.094	3011	-0.978415454	-2.987227125	0.60017974
84494	6/14/2016 5:27	B	1193051.233	21411813.27	-73.221	-42.128	7269	-1.038089638	-2.987227125	0.276157954
84494	6/14/2016 7:13	B	716089.4108	4249466.589	-73.261	-42.141	6387	-1.102829632	-2.987227125	0.285622092
84494	6/14/2016 8:46	B	151134138.3	36667015.71	-73.259	-42.293	5577	-0.741145915	-1.604423307	0.085277818
84494	6/14/2016 10:15	B	94421946.65	6879447.852	-73.251	-42.294	5329	-0.744883002	-1.588931428	0.082771451
84494	6/14/2016 11:55	A	3144362.896	394179.6038	-73.01	-42.226	6018	-0.786027137	-2.987227125	-0.087073329
84494	6/14/2016 13:02	B	301771911	4332111.528	-72.959	-42.216	4011	-0.764290187	-2.987227125	-0.162506124
84494	6/14/2016 14:04	B	104872383.6	10015401.42	-72.912	-42.203	3741	-0.778679028	-2.987227125	-0.232791523
84494	6/14/2016 14:48	B	129231145.1	196966232.9	-73.08	-42.229	2593	-0.768110862	-2.987227125	0.012603247
84494	6/14/2016 19:29	B	407552994.3	18978312.24	-73.222	-42.205	16885	-0.812541436	-2.987227125	0.201420926
84494	6/14/2016 21:06	B	45226508.26	1449718578	-73.313	-42.213	5805	-0.894460948	-1.939922973	0.212272878
84494	6/14/2016 21:32	B	435398.6528	3691979.347	-73.329	-42.217	1559	-0.899948945	-1.70115413	0.205898197
84494	6/14/2016 22:12	B	823927.0694	2804201.931	-73.352	-42.214	2421	-0.937121163	-1.521226025	0.204959004
84494	6/14/2016 23:16	B	2945092448	111277092.4	-73.345	-42.249	3840	-0.836979285	-1.441961174	0.160833454

84494	6/14/2016 23:52	B	41486458.25	1670646.755	-73.355	-42.254	2168	-0.83134526	-1.386540389	0.154343335
84494	6/15/2016 1:38	B	20970149.3	1341044.704	-73.366	-42.285	6343	-0.786237028	-1.168152404	0.106640878
84494	6/15/2016 4:41	B	11064062.4	4921714.604	-73.056	-42.263	11013	-0.458825229	-2.333742008	-0.531845182
84494	6/15/2016 5:17	A	184470.1427	2280819.857	-73.012	-42.33	2113	-0.850558977	-2.801081115	-0.642588146
84494	6/15/2016 9:41	B	258101309.5	136658727	-73.037	-42.344	15855	-0.831474736	-2.657125467	-0.670811761
84494	6/15/2016 10:33	B	4573928562	1402556.579	-73.044	-42.391	3151	-0.811631826	-2.145264362	-0.742446348
84494	6/15/2016 14:25	B	700304917.6	92917840.85	-73.38	-42.409	13910	-0.78569461	-0.714471125	-1.054578594
84494	6/15/2016 21:08	B	50459379.5	17165913.5	-73.369	-42.363	24141	-0.662782227	-0.721447298	-0.978364077
84494	6/15/2016 22:01	B	41380134.56	13215669.94	-73.428	-42.389	3175	-0.68453068	-0.599999226	-1.007290482
84494	6/15/2016 22:44	B	6711589346	51416058.7	-73.487	-42.44	2580	-0.767169812	-0.731416674	-1.063557866
84494	6/15/2016 22:44	B	4936844779	37845619.83	-73.485	-42.439	55	-0.767169812	-0.731416674	-1.063557866
84494	6/16/2016 0:20	B	8633594.693	149787829.8	-73.329	-42.368	5759	-0.707743665	-0.746566067	-0.988035405
84494	6/16/2016 0:33	B	188996109.5	86107239.47	-73.332	-42.381	725	-0.725069134	-0.735518715	-1.004132699
84494	6/16/2016 1:02	B	40259945.42	35711339.58	-73.415	-42.402	1755	-0.743509716	-0.635216755	-1.035326063
84494	6/16/2016 2:41	B	107237461.1	26602823.95	-73.386	-42.409	5952	-0.782032126	-0.704554351	-1.052090292
84494	6/16/2016 4:23	B	531936179.3	89564020.65	-73.374	-42.403	6131	-0.916228787	-0.697733919	-1.039187831

84494	6/16/2016 5:09	B	586397185.2	27545627.31	-73.394	-42.429	2734	-0.93481185	-0.784606898	-1.049260847
84494	6/16/2016 6:47	B	1064697788	87546274.09	-73.377	-42.433	5870	-0.903628581	-0.825672519	-1.034986098
84494	6/16/2016 8:21	B	415011853.9	13427204.14	-73.391	-42.405	5685	-0.999918078	-0.690428167	-1.05139285
84494	6/16/2016 8:32	B	151898787.9	5093626.619	-73.398	-42.403	652	-1.006994803	-0.671261317	-1.055318644
84494	6/16/2016 10:10	B	15048986915	467909545.4	-73.404	-42.429	5894	-0.852176768	-0.748309103	-1.071587862
84494	6/16/2016 11:50	A	690588.4742	9964356.026	-73.47	-42.41	5995	-0.311384987	-0.587684169	-1.019463895
84494	6/16/2016 12:23	B	291033021.5	69290685.03	-73.483	-42.427	1964	-0.208515048	-0.632659045	-1.040152181
84494	6/16/2016 12:31	B	14020320.09	7672212.911	-73.482	-42.441	466	-0.329808019	-0.69297456	-1.070365548
84494	6/16/2016 13:59	0	405906592.9	1634097740	-73.435	-42.265	5283	-0.393684374	-0.792240928	-0.871979167
84494	6/16/2016 19:03	B	21842354624	6175874576	-73.419	-42.31	18277	-0.279420701	-0.662736776	-0.980350378
84494	6/16/2016 20:40	A	5248027.711	378874.7892	-73.437	-42.408	5784	-0.748009792	-0.628189291	-1.04827889
84494	6/16/2016 21:54	B	9708442.251	1891950.249	-73.442	-42.415	4424	-0.682628489	-0.652357589	-1.05592144
84494	6/16/2016 22:26	B	5836222.68	1626939.82	-73.442	-42.413	1934	-0.685814017	-0.64441186	-1.053309526
84494	6/16/2016 23:32	B	50281138971	2679997416	-73.435	-42.457	3977	-0.80789126	-0.801641377	-1.110522448
84494	6/16/2016 23:45	0	313957.4394	7666385.061	-73.451	-42.458	785	-0.773267334	-0.826977703	-1.11049902
84494	6/17/2016 0:10	B	367001758.8	7702009.732	-73.474	-42.472	1504	-0.637717327	-0.843781484	-1.09346094

84494	6/17/2016 1:13	B	262340777.7	6891792.279	-73.461	-42.475	3753	-0.769036184	-0.884832888	-1.103680536
84494	6/17/2016 1:42	B	6063673.303	260139.6965	-73.46	-42.481	1773	-0.751414694	-0.915286475	-1.095831392
84494	6/17/2016 6:27	B	4294303824	298925144.2	-73.168	-42.541	17103	0.173530787	-1.51002896	-0.297818003
84494	6/17/2016 8:05	B	12565084.5	83282418	-73.406	-42.557	5867	0.087302317	-1.567835714	-0.992699995
84494	6/17/2016 9:43	B	2974979622	58909245.56	-73.398	-42.502	5866	0.451005281	-1.385664768	-0.907602294
84494	6/17/2016 10:14	B	41285174	25722786.5	-73.398	-42.553	1880	0.006483976	-1.669447272	-1.026681799
84494	6/17/2016 11:05	B	3992141.412	722197.088	-73.408	-42.514	3041	0.257447254	-1.435190589	-0.941371354
84494	6/17/2016 11:28	B	4318860.5	860672	-73.407	-42.512	1405	0.268173548	-1.432921662	-0.937836406
84494	6/17/2016 12:04	A	3779095.815	5281808.185	-73.498	-42.458	2157	0.179800144	-0.714023262	-0.887062391
84494	6/17/2016 13:44	B	889374043.5	72529457.48	-73.553	-42.393	5966	-0.143411946	-0.38866786	-0.639853402
84494	6/17/2016 18:50	B	38120536616	758888826.4	-73.545	-42.375	18374	0.667431256	-0.52349185	-0.655749773
84494	6/17/2016 20:29	A	57667726.77	6858117.233	-73.389	-42.483	5953	0.085936393	-1.163060784	-0.827651556
84494	6/17/2016 21:38	A	91625.68389	40439.31611	-73.505	-42.385	4121	-0.485632467	-0.461197992	-0.567746015
84494	6/17/2016 23:26	A	4524274616	241163321.5	-73.327	-42.377	6511	0.641704845	-0.727906466	-0.830404447
84494	6/17/2016 23:35	B	836703507.6	38855304.86	-73.329	-42.374	501	0.823671773	-0.73952595	-0.814001418
84494	6/18/2016 1:58	A	4858276.512	21999135.99	-73.449	-42.472	8592	-0.233155311	-0.893041153	-0.862891394

84494	6/18/2016 2:53	B	27978405.93	4109708.565	-73.482	-42.48	3288	-0.54351911	-0.843660509	-0.905082428
84494	6/18/2016 3:35	B	218476776	78905234.52	-73.422	-42.454	2516	-0.17421096	-0.88818699	-0.808544422
84494	6/18/2016 6:22	A	83241.69276	2080.807244	-73.493	-42.42	10043	0.398901736	-0.58459428	-0.606636439
84494	6/18/2016 8:02	B	11273129.82	31216956.68	-73.522	-42.406	6002	0.256331724	-0.477438909	-0.658407739
84494	6/18/2016 9:08	B	10085912.72	2006195.779	-73.524	-42.385	3931	-0.029653902	-0.423998392	-0.642257776
84494	6/18/2016 9:23	B	2625258.163	532831.8374	-73.527	-42.382	938	-0.075774411	-0.415337075	-0.644885868
84494	6/18/2016 9:48	B	1260833.406	1567423.594	-73.567	-42.367	1479	-0.60821791	-0.31685403	-0.707930247
84494	6/18/2016 10:48	B	99334514.27	10532247.73	-73.605	-42.382	3591	-0.453379809	-0.276091932	-0.772944532
84494	6/18/2016 11:00	B	13237135.83	1075876.67	-73.608	-42.383	731	-0.44452906	-0.267195243	-0.778870291
84494	6/18/2016 14:15	B	4871141854	24428438.29	-73.424	-42.428	11699	-0.436707067	-0.710365606	-0.54331044
84494	6/18/2016 15:03	B	782817428.4	33897570.11	-73.419	-42.442	2870	-0.462824902	-0.816309398	-0.565107188
84494	6/18/2016 20:22	B	2178376470	253726218.7	-73.417	-42.444	19169	-0.513973737	-0.827651828	-0.565848246
84494	6/18/2016 21:06	A	84699.36646	47423.13354	-73.41	-42.419	2631	-0.912171241	-0.71757504	-0.532838092
84494	6/18/2016 22:03	B	3878704.927	1183783.573	-73.404	-42.41	3385	-0.973691682	-0.681861048	-0.530530162
84494	6/18/2016 22:51	B	3503468.877	1086792.123	-73.396	-42.404	2936	-0.991138794	-0.680531241	-0.531691719
84494	6/18/2016 23:12	B	5567594.792	418034.2077	-73.398	-42.392	1234	-0.943833227	-0.655555648	-0.538405531

84494	6/19/2016 0:54	B	4948587.525	711457.4753	-73.387	-42.377	6145	-0.871199376	-0.675214615	-0.546460951
84494	6/19/2016 1:41	A	51654424.38	2370658.115	-73.244	-42.365	2775	0.176851341	-0.83692311	-0.133364514
84494	6/19/2016 3:23	B	2457637782	959619851.2	-73.17	-42.318	6123	-0.070626875	-1.242110026	-0.393863851
84494	6/19/2016 6:11	B	1337327736	608437417.8	-73.242	-42.377	10077	-0.363936828	-0.866027942	-0.601156741
84494	6/19/2016 10:34	1	940852.2749	69544.22512	-73.204	-42.387	15823	0.175170011	-0.981160142	-0.524728488
84494	6/19/2016 10:57	B	52431.34872	8317189.651	-73.216	-42.392	1330	0.040092611	-0.946023867	-0.557120106
84494	6/19/2016 12:38	B	12249007.25	12249007.25	-73.111	-42.405	6056	0.208410121	-1.51627067	-0.135204786
84494	6/19/2016 13:08	A	56387676.18	49986876.32	-73.075	-42.389	1831	-0.532512376	-2.030893719	-0.065473005
84494	6/19/2016 13:59	B	3399566.748	1712519.752	-73.047	-42.41	3043	-0.320052336	-1.986356174	-0.003251185
84494	6/19/2016 14:41	B	997178040.8	22148651.67	-72.998	-42.474	2509	-0.043382922	-2.711215909	-0.018528004
84494	6/19/2016 20:11	B	786759997.9	86346279.09	-73.07	-42.413	19829	-0.252099831	-1.768400689	-0.022413481
84494	6/19/2016 20:36	B	908871612.5	12029512.5	-73.069	-42.372	1469	-0.583486975	-2.37527435	-0.074145445
84494	6/19/2016 21:53	B	1473151617	23365075.06	-73.072	-42.376	4640	-0.325166671	-2.05963114	-0.106265239
84494	6/19/2016 22:17	B	5188764768	234430254.9	-73.142	-42.386	1422	0.423389232	-1.376546602	-0.254999624
84494	6/20/2016 0:33	A	8202984.257	2972964.243	-73.384	-42.389	8162	-0.390201031	-0.677854945	-0.68088818
84494	6/20/2016 0:45	B	1358881.561	371596.9393	-73.384	-42.389	736	-0.390201031	-0.677854945	-0.68088818

84494	6/20/2016 2:56	A	72337613073	1428832539	-73.588	-42.409	7854	-0.111193407	-0.442358862	-1.013532181
84494	6/20/2016 3:49	B	473130709.6	26156832.93	-73.61	-42.416	3205	-0.763542725	-0.348186267	-1.111657138
84494	6/20/2016 4:42	B	550611074.7	16089970.34	-73.545	-42.398	3189	-0.772973902	-0.414465707	-1.34252463
84494	6/20/2016 7:44	1	1450492.762	149348.2378	-73.182	-42.357	10899	1.158577803	-1.128805796	-0.650394997
84494	6/20/2016 8:29	A	49611449.39	56989675.61	-73.224	-42.369	2710	1.179145285	-0.907080737	-0.848771387
84494	6/20/2016 10:26	B	65716189.4	3574475.599	-73.193	-42.379	7031	1.474097107	-1.05531701	-0.632160404
84494	6/20/2016 12:07	B	5101389.668	352026.3316	-73.103	-42.371	6016	-0.289319251	-1.860161286	-0.333443514
84494	6/20/2016 12:43	B	18187861.33	510756.6726	-73.113	-42.387	2154	-0.34972156	-1.612713685	-0.345876389
84494	6/20/2016 14:21	A	192762.1973	1462.802728	-73.049	-42.305	5925	1.116824773	-2.088044721	-0.570371916
84494	6/20/2016 18:17	A	364467591.2	48757187.33	-73.083	-42.264	14158	-0.128959618	-2.260824668	-0.552355488
84494	6/20/2016 19:57	B	1488333129	214381983.2	-73.156	-42.286	5974	-0.242402352	-1.728403382	-0.507109614
84494	6/20/2016 21:42	A	314767.0555	75200.94445	-73.283	-42.384	6338	-0.806085787	-0.793382744	-0.941721061
84494	6/20/2016 22:46	1	841417.0755	815616.9245	-73.29	-42.356	3793	-0.907744708	-0.831949539	-0.922401117
84494	6/20/2016 23:25	A	69451321.35	105810350.7	-73.313	-42.396	2362	-0.427345716	-0.759844726	-0.964578722
84494	6/21/2016 0:08	A	2913843.139	5909293.861	-73.281	-42.405	2565	-0.544600895	-0.825590413	-0.944289956
84494	6/21/2016 0:33	A	130133962.3	5962506.725	-73.252	-42.385	1507	0.012933381	-0.835763504	-0.913275916

84494	6/21/2016 0:57	B	107588836	12482568.96	-73.257	-42.395	1451	0.098633433	-0.838221268	-0.904286594
84494	6/21/2016 2:44	B	130840824.9	41720809.58	-73.275	-42.433	6406	0.528166586	-0.993558041	-0.895344324
84494	6/21/2016 3:31	B	271286519.7	75388377.28	-73.277	-42.456	2823	0.86193303	-1.114786482	-0.863090876
84494	6/21/2016 4:19	B	7392209.277	1990000.723	-73.292	-42.485	2862	-0.65817045	-1.274545975	-1.078419822
84494	6/21/2016 5:53	B	7382020.9	1405221.6	-73.287	-42.498	5648	-0.429607833	-1.349713671	-1.05869456
84494	6/21/2016 7:29	B	421394.9288	11773391.57	-73.299	-42.402	5751	0.112852218	-0.796046884	-0.907955216
84494	6/21/2016 8:32	B	1021382.838	1136705.662	-73.303	-42.404	3806	0.124575164	-0.797400252	-0.917003846
84494	6/21/2016 9:50	B	9787348.005	60424.99523	-73.303	-42.437	4676	-0.233864933	-0.943640266	-1.028222452
84494	6/21/2016 10:14	B	16418087.73	357074.7747	-73.31	-42.444	1431	-0.340094985	-0.948795293	-1.04160111
84494	6/21/2016 11:25	B	9220974.031	497390.4685	-73.311	-42.442	4279	-0.340094985	-0.935830114	-1.04160111
84494	6/21/2016 12:00	B	13416394.46	837250.036	-73.349	-42.41	2119	0.304576946	-0.736685908	-0.998949595
84494	6/21/2016 13:58	A	685376204.5	90993560.55	-73.148	-42.343	7055	-0.055100806	-1.248027151	-0.520410487
84494	6/21/2016 21:25	A	2486268990	132635832.4	-73.6	-42.393	26815	-0.606540433	-0.337094542	-1.114185875
84494	6/21/2016 21:57	B	1144493697	51928016.99	-73.564	-42.411	1917	-0.756534104	-0.419516296	-1.149003264
84494	6/21/2016 22:51	B	12359.4829	1042620.517	-73.476	-42.432	3276	-0.649082434	-0.665035273	-1.192037762
84494	6/21/2016 23:39	A	731961.3655	12077.13451	-73.406	-42.476	2823	-0.794931446	-1.076922901	-1.226788773

84494	6/22/2016 0:15	B	29524.5	12024608	-73.434	-42.492	2161	-0.996526048	-1.076490159	-1.236580637
84494	6/22/2016 1:22	B	2242569.801	1330960.699	-73.429	-42.519	4025	-0.759714693	-1.311676156	-1.209813702
84494	6/22/2016 2:22	B	5005891.72	3438112.78	-73.42	-42.541	3616	-0.690002212	-1.513556148	-1.180953239
84494	6/22/2016 4:01	B	81317842.57	4409231.427	-73.39	-42.574	5976	-0.80095663	-1.534054318	-0.948947065
84494	6/22/2016 7:18	B	17915362.37	249000.131	-73.347	-42.508	11779	0.032276278	-1.556614288	-0.922995206
84494	6/22/2016 9:24	B	9594700.11	876572.3897	-73.252	-42.569	7595	0.750275174	-1.361602342	-0.914541752
84494	6/22/2016 12:44	B	6009540130	718199159.6	-72.987	-42.422	12005	-0.694253334	-2.987227125	-0.469173997
84494	6/22/2016 13:35	B	2247490118	179411822.7	-72.852	-42.394	3036	2.019542269	-2.909336092	-0.657467036
84494	6/22/2016 21:33	B	840663181.4	38010770.55	-72.976	-42.35	28671	-0.349332371	-2.987227125	-0.416031346
84494	6/22/2016 23:14	B	8167186226	369706434.6	-73.18	-42.328	6073	-0.567439533	-1.511152101	-0.409923232
84494	6/23/2016 0:13	B	39194971953	1770885563	-73.227	-42.347	3508	0.096758051	-1.616300179	-0.464854578
84494	6/23/2016 2:02	B	993234546.4	182019378.1	-73.069	-42.45	6585	1.982187386	-1.513349514	-0.888327677
84494	6/23/2016 3:39	B	250705704.2	15341964.76	-73.081	-42.416	5795	2.007111974	-1.641632985	-0.801738675
84494	6/23/2016 7:07	A	4479169252	202377397	-72.939	-42.335	12499	0.104705374	-2.969051252	-0.337424264
84494	6/23/2016 8:56	B	13505880.3	113693239.7	-73.08	-42.29	6507	-0.614242898	-1.814037371	-0.527188591
84494	6/23/2016 9:56	1	16057660.26	4191780.743	-73.084	-42.349	3611	-1.047244295	-2.094864328	-0.471265617

84494	6/23/2016 10:31	A	44209.5259	165750.9741	-73.113	-42.355	2078	-0.881509433	-1.698051584	-0.465019596
84494	6/23/2016 10:34	A	40522.42212	31968.07788	-73.114	-42.355	230	-0.887791331	-1.682792329	-0.464237402
84494	6/23/2016 11:38	B	1091303.38	322540.6202	-73.134	-42.367	3796	-0.58905787	-1.437495649	-0.487157005
84494	6/23/2016 11:44	B	1083659.71	431377.2903	-73.131	-42.365	377	-0.659436221	-1.462966897	-0.480628759
84494	6/23/2016 12:08	B	284148346.6	4285065.38	-73.127	-42.324	1464	-0.931641036	-1.410563338	-0.465950069
84494	6/23/2016 15:05	B	19568835.48	3014387.021	-73.122	-42.288	10587	-0.579558813	-1.749961648	-0.508592935
84494	6/23/2016 19:30	B	1454771812	77573006.91	-73.09	-42.444	15897	-0.732343051	-1.411478773	-0.593565897
84494	6/23/2016 21:02	B	866205675.2	141405777.8	-73.154	-42.469	5547	-0.620769123	-1.510483673	-0.637871037
84494	6/23/2016 21:49	A	40371.18635	9085.813651	-73.073	-42.462	2788	-0.904926978	-1.558585599	-0.576804319
84494	6/23/2016 22:48	B	479211200	39403530.5	-73.08	-42.408	3570	-0.738286556	-1.709337509	-0.521647072
84494	6/23/2016 23:26	B	22004538.94	151777.5595	-73.042	-42.441	2277	-0.721569527	-1.773576775	-0.532002113
84494	6/23/2016 23:59	B	6995499.673	299261.327	-73.029	-42.439	1973	-0.675507577	-2.036244764	-0.519272544
84494	6/24/2016 0:26	B	3081416.777	302455.2231	-73.046	-42.439	1650	-0.747119661	-1.759360412	-0.537258195
84494	6/24/2016 2:26	B	4875282246	39068516.6	-73.201	-42.293	7174	-0.683606467	-1.636327436	-0.436871258
84494	6/24/2016 5:19	B	36103671.68	5745930.321	-73.27	-42.232	10362	0.492000702	-2.710685343	-0.401815782
84494	6/24/2016 6:53	B	2554556229	238787491.1	-73.292	-42.281	5682	-0.021387371	-1.851195141	-0.581423536

84494	6/24/2016 8:35	B	672210603.1	55689633.94	-73.143	-42.263	6072	-0.205571615	-2.279800174	-0.580640765
84494	6/24/2016 9:42	B	102905211.5	34548270.5	-73.069	-42.31	4044	0.115825722	-1.850341102	-0.729080847
84494	6/24/2016 11:41	B	7070169652	1681878413	-73.097	-42.362	7121	0.957443272	-2.057349588	-0.890720808
84494	6/24/2016 11:55	B	167043766.2	36729555.83	-73.096	-42.361	857	0.86146268	-2.061588561	-0.883815753
84494	6/24/2016 13:01	A	42790826.22	2695016.278	-73.171	-42.355	3948	0.006817844	-1.180337223	-0.63977548
84494	6/24/2016 14:46	B	28860053.17	7305807.831	-73.17	-42.366	6325	0.161207047	-1.165702274	-0.625137663
84494	6/24/2016 19:18	A	114507397.8	330124.6702	-73.301	-42.381	16281	0.168053104	-0.780593664	-0.592878216
84494	6/24/2016 20:51	B	5788257.933	3037724.567	-73.319	-42.389	5632	0.5125414	-0.752241979	-0.656252893
84494	6/24/2016 21:20	A	12928621.9	148067.0985	-73.289	-42.34	1711	-0.165757217	-0.957150782	-0.554500644
84494	6/24/2016 21:58	B	5543087.826	2236402.174	-73.282	-42.358	2297	-0.206352241	-0.83209531	-0.526507802
84494	6/25/2016 1:25	B	191967712.1	65477737.87	-72.819	-42.425	12429	2.949326364	-2.723818325	-0.836118917
84494	6/25/2016 4:35	B	14499770770	8850807216	-72.872	-42.493	11397	1.23496779	-2.863808257	-1.26502911
84494	6/25/2016 5:04	B	418968291.4	141010849.1	-72.85	-42.512	1724	1.136133336	-2.750116985	-1.171152204
84494	6/25/2016 6:45	0	47277701.26	3981063.236	-72.889	-42.541	6045	0.910474279	-2.36277983	-0.93928814
84494	6/25/2016 7:50	B	153191636.3	41978780.7	-72.854	-42.561	3930	0.327573179	-1.924629439	-0.965101518
84494	6/25/2016 8:07	B	965859395.5	213950667	-72.861	-42.558	980	0.412121963	-1.97808989	-0.95128317

84494	6/25/2016 9:34	B	4985655.634	20934586.87	-72.944	-42.562	5217	1.42576505	-2.932830054	-0.597098673
84494	6/25/2016 9:49	A	8104279.085	582224.9149	-72.944	-42.595	923	0.958581233	-2.962505363	-0.484071642
84494	6/25/2016 11:16	A	230952.5423	202945.4577	-72.924	-42.567	5248	1.178202876	-2.695094709	-0.681013527
84494	6/25/2016 12:44	B	11273379.81	4394805.188	-72.918	-42.581	5248	1.146288426	-2.68276709	-0.674332223
84494	6/25/2016 19:02	B	109803088.8	14563965.71	-72.943	-42.487	22705	1.744976232	-2.987227125	-1.174786765
84494	6/25/2016 20:39	0	774526971.1	1365352.936	-72.857	-42.502	5826	1.300815084	-2.835419505	-1.218624236
84494	6/25/2016 20:47	B	166487783.8	23869170.68	-72.857	-42.502	442	1.29623382	-2.824063752	-1.22659543
84494	6/25/2016 21:52	B	681356213.3	836995367.7	-72.835	-42.517	3884	1.101603695	-2.823508219	-1.206027888
84494	6/25/2016 21:58	B	757135.0672	168391.4328	-72.843	-42.507	390	1.199993008	-2.829153698	-1.24500467
84494	6/25/2016 22:26	1	46751590.61	2501955.395	-72.868	-42.5	1678	1.373636663	-2.852645703	-1.218761382
84494	6/25/2016 23:45	B	1261632.807	46000577.69	-72.814	-42.502	4764	1.186366662	-2.937986791	-1.33268791
84494	6/26/2016 0:55	B	1010967.448	867903.0518	-72.799	-42.496	4171	1.070825569	-2.963169652	-1.390592459
84494	6/26/2016 1:08	B	15474951.39	39617578.61	-72.801	-42.503	775	1.109070539	-2.955371712	-1.358641276
84494	6/26/2016 2:32	B	1785655.25	1785655.25	-72.785	-42.5	5023	0.815300396	-2.907393879	-1.402642501
84494	6/26/2016 3:31	B	5337662.169	2368969.831	-72.781	-42.497	3597	0.724860022	-2.911362853	-1.405895702
84494	6/26/2016 4:12	0	17206419.28	5380713.716	-72.805	-42.455	2429	0.135045947	-2.951418968	-0.785866638

84494	6/26/2016 4:49	B	67537170.43	35220092.07	-72.792	-42.439	2244	-0.454271898	-2.842296741	-0.838128168
84494	6/26/2016 6:34	0	22897593.92	4617986.583	-72.894	-42.494	6281	0.455750696	-2.920909765	-0.274884168
84494	6/26/2016 8:11	B	3130086109	461385485.1	-72.923	-42.476	5790	0.499207682	-2.987227125	-0.245088008
84494	6/26/2016 9:23	B	75948747.36	3434217.139	-72.943	-42.469	4346	0.884495804	-2.987227125	-0.279429845
84494	6/26/2016 10:37	B	4942644.973	9968173.527	-73.019	-42.511	4446	0.096138729	-2.740527577	-0.240619197
84494	6/26/2016 10:56	B	2412268.385	1476134.115	-73.028	-42.489	1160	1.361231293	-2.456050045	-0.315617912
84494	6/26/2016 12:23	B	79075140.5	26859405.5	-72.934	-42.419	5199	1.142909643	-2.987227125	-0.592908626
84494	6/26/2016 13:57	B	14433034892	362081874.2	-72.922	-42.561	5619	-0.800088552	-2.878432984	-0.238637606
84494	6/26/2016 15:38	B	50429338.34	18905133.66	-72.964	-42.616	6066	-0.591424595	-2.931345954	-0.11767985
84494	6/26/2016 18:48	B	62687970.92	14916097.08	-72.945	-42.598	11440	-0.294142638	-2.962505363	-0.185141
84494	6/26/2016 20:16	B	10285996.71	12405473.79	-73.032	-42.501	5264	0.735964633	-2.45738972	-0.251524663
84494	6/26/2016 21:39	B	23365947.48	4961573.023	-73.073	-42.513	4955	0.316487142	-1.836471315	-0.100624944
84494	6/26/2016 21:57	B	4917794997	459674601.6	-73.062	-42.505	1116	0.484063415	-1.820131561	-0.108638884
84494	6/26/2016 22:13	B	30837675.63	6203350.874	-73.079	-42.512	947	0.353465286	-1.820191681	-0.084673924
84494	6/26/2016 23:16	B	4378723.271	1006500.729	-73.075	-42.518	3751	0.19595634	-1.873812637	-0.081575448
84494	6/26/2016 23:33	B	9509038.088	494961.912	-73.043	-42.481	1048	1.841694787	-2.094607423	-0.332376991

84494	6/27/2016 0:55	B	20314205.48	276219.5201	-73.062	-42.454	4938	2.560092199	-1.625639323	-0.481585177
84494	6/27/2016 2:09	B	2661580527	315561533.6	-73.078	-42.476	4419	2.048934481	-1.609518961	-0.255237717
84494	6/27/2016 3:05	B	15749065.23	2030687.275	-73.084	-42.474	3337	2.12126299	-1.578779205	-0.245154064
84494	6/27/2016 3:52	B	10631892	1474292.497	-73.084	-42.473	2825	2.199289486	-1.578779205	-0.256591178
84494	6/27/2016 6:22	A	14116.64297	59048.35703	-72.949	-42.481	9008	-0.167139879	-2.987227125	0.24942284
84494	6/27/2016 7:59	A	57133.28509	4429.214912	-72.925	-42.468	5833	-0.284772529	-2.987227125	0.242337902
84494	6/27/2016 8:59	B	959619.9213	306128.5787	-72.913	-42.465	3573	-0.237379506	-2.987227125	0.232443029
84494	6/27/2016 9:04	B	1225890.874	283791.6256	-72.914	-42.468	334	-0.249464421	-2.987227125	0.234800726
84494	6/27/2016 10:30	B	2633368.101	734140.399	-72.897	-42.464	5165	-0.090141392	-2.987227125	0.210357785
84494	6/27/2016 10:43	B	2737767.911	824764.5894	-72.897	-42.463	742	-0.074445118	-2.987227125	0.207420712
84494	6/27/2016 11:48	B	200760722	6160.5	-72.893	-42.442	3936	0.086813382	-2.987227125	0.144620901
84494	6/27/2016 13:39	B	66465023.35	15495493.65	-72.976	-42.442	6666	0.187763775	-2.987227125	0.084247391
84494	6/27/2016 14:26	B	73129176.11	17505196.89	-72.973	-42.442	2813	0.200761603	-2.987227125	0.092767613
84494	6/27/2016 18:37	A	424981.3027	2334527.697	-72.821	-42.533	15042	1.201554465	-2.530274689	-0.230213503
84494	6/27/2016 20:21	A	76202876.68	4062497.823	-72.814	-42.505	6209	0.876934599	-2.937986791	-0.128616591
84494	6/27/2016 22:01	B	179767172.5	15853309.52	-72.652	-42.378	6031	0	-0.90481199	0

84494	6/27/2016 22:48	B	21191698752	103772810.7	-72.625	-42.413	2817	0	-0.92592444	0
84494	6/27/2016 23:09	B	153623920.9	9058317.57	-72.595	-42.369	1288	0	-0.742495933	0
84494	6/28/2016 3:38	B	10967204.13	2708700.366	-72.456	-42.294	16101	0	-0.580361677	-0.726070096
84494	6/28/2016 5:14	B	176554197.4	98694701.13	-72.729	-42.42	5744	-0.969970125	-1.695378486	-0.324384316
84494	6/28/2016 6:11	B	32595061685	491428997.9	-72.73	-42.344	3464	0	-1.143443769	0
84494	6/28/2016 7:49	0	13366598.16	2606394.341	-72.774	-42.371	5837	-0.852578406	-1.377023154	-0.295691006
84494	6/28/2016 8:33	A	144953585.5	2776880.475	-72.742	-42.349	2676	0	-1.137274113	0
84494	6/28/2016 9:01	B	7823244.406	1448987.594	-72.742	-42.348	1668	0	-1.13127093	0
84494	6/28/2016 9:34	B	6599344.5	1765320.5	-72.744	-42.347	1973	0	-1.137274113	0
84494	6/28/2016 10:12	0	11937289839	451046591.4	-72.783	-42.411	2269	-0.907947251	-1.842277768	-0.283788255
84494	6/28/2016 10:36	B	74980318.27	3437568.233	-72.783	-42.407	1451	-0.865022949	-2.016283089	-0.271530632
84494	6/28/2016 11:12	A	239143.8954	3176.104648	-72.818	-42.398	2173	-0.707802711	-2.154666359	-0.240577959
84494	6/28/2016 11:39	B	1106821.842	286018.1581	-72.825	-42.397	1601	-0.684994224	-2.295839393	-0.234331077
84494	6/28/2016 12:56	B	2609951.623	441521.3771	-72.852	-42.398	4625	-0.617431218	-2.711809437	-0.199529328
84494	6/28/2016 14:55	B	6721270.326	1630142.674	-72.791	-42.369	7168	-0.809779827	-1.476347681	-0.291184315
84494	6/28/2016 20:08	B	172594693.4	16168943.15	-72.837	-42.479	18734	-0.409051452	-2.987227125	-0.15302003

84494	6/28/2016 20:55	B	30153466.62	2341599.879	-72.742	-42.454	2846	-0.930098677	-2.388204014	-0.310553999
84494	6/28/2016 21:55	B	6185612.85	20101672.15	-72.767	-42.442	3595	-0.869990283	-2.540765565	-0.283987786
84494	6/28/2016 22:31	A	4754186.858	119519.6418	-72.791	-42.402	2173	-0.826310503	-1.983830368	-0.266038275
84494	6/28/2016 22:54	B	33798284.61	373937.8943	-72.797	-42.389	1357	-0.809609018	-1.740781491	-0.27222324
84494	6/29/2016 0:00	B	1869737.456	304623.0439	-72.806	-42.367	3979	-0.779445457	-1.567239962	-0.288085188
84494	6/29/2016 0:14	B	1906900.88	373208.1203	-72.804	-42.368	855	-0.783539763	-1.558583209	-0.286767374
84494	6/29/2016 0:34	B	22022066.28	5725837.719	-72.808	-42.359	1175	-0.771429048	-1.513598404	-0.294318073
84494	6/29/2016 1:29	B	2729315.223	561679.2767	-72.817	-42.339	3313	-0.741383356	-1.725301973	-0.306338098
84494	6/29/2016 2:28	B	2079648.673	1386444.327	-72.82	-42.327	3537	-0.714043109	-1.856269221	-0.31444754
84494	6/29/2016 3:10	B	2622111.488	1841978.512	-72.82	-42.333	2530	-0.732679565	-1.800538548	-0.309195151
84494	6/29/2016 4:54	B	1048004.437	11599020.56	-72.786	-42.326	6257	0	-1.445534635	-1.116285366
84494	6/29/2016 6:00	B	27407710.1	4060731.901	-72.796	-42.37	3953	0.185714553	-1.519008654	-1.031435445
84494	6/29/2016 7:34	B	189846753.1	30993515.35	-72.823	-42.387	5617	0.10182837	-2.076505946	-0.933113189
84494	6/29/2016 8:48	B	38071584.38	8281220.117	-72.807	-42.403	4445	-0.147138672	-2.193052016	-0.985118803
84494	6/29/2016 9:47	B	2584134.598	213749.9021	-72.788	-42.392	3517	-0.127671536	-1.704508153	-1.037392279
84494	6/29/2016 10:30	B	3605127.137	744365.3635	-72.807	-42.411	2589	-0.212964683	-2.35967818	-0.981068565

84494	6/29/2016 11:23	B	77553750.99	18543091.01	-72.965	-42.424	3210	-0.244855734	-2.987227125	-0.921461793
84494	6/29/2016 12:25	B	236240591.1	26308633.91	-72.962	-42.38	3696	-0.643153054	-2.987227125	-0.803604777
84494	6/29/2016 13:00	B	4795564.573	719531.9272	-72.935	-42.356	2092	-0.502656902	-2.987227125	-0.803875026
84494	6/29/2016 13:48	B	361607658.1	13666111.9	-72.935	-42.355	2885	-0.502656902	-2.987227125	-0.803875026
84494	6/29/2016 14:42	B	13117822.39	584585.6093	-72.917	-42.359	3275	-0.296749816	-2.970271307	-0.807202064
84494	6/29/2016 20:20	B	14197233.64	1902758.859	-72.922	-42.314	20229	1.244648013	-2.987227125	-0.989206879
84494	6/29/2016 22:02	B	32587731.6	796912.9005	-72.91	-42.376	6130	-0.601574809	-2.952355082	-0.785980884
84494	6/30/2016 0:28	B	639768836.8	93051645.69	-73.083	-42.483	8799	-0.826116519	-1.63403651	-0.814014784
84494	6/30/2016 1:18	B	252981303.5	39900280.98	-73.095	-42.49	3000	-0.695290684	-1.620394729	-0.835447601
84494	6/30/2016 4:30	B	5745219988	406044975.5	-73.282	-42.581	11513	1.176498369	-1.434285604	-1.07543376
84494	6/30/2016 5:50	B	3265529070	485459610	-73.172	-42.525	4753	0.214947842	-1.777938814	-0.999523045
84494	6/30/2016 7:29	B	701650603.4	7771041.588	-73.092	-42.564	5972	1.415351612	-2.35060223	-0.77689345
84494	6/30/2016 8:36	B	11222316.38	728590.124	-73.007	-42.567	4001	-0.126882533	-2.987227125	-0.713034812
84494	6/30/2016 9:26	B	6339085.144	727381.3565	-72.97	-42.578	2997	-0.152963237	-2.987227125	-0.715797286
84494	6/30/2016 10:07	B	9862549.664	1070396.336	-72.93	-42.586	2472	0.890336047	-2.815430518	-0.700536738
84494	6/30/2016 11:02	B	291930444.1	69448608.37	-72.863	-42.495	3337	2.218591158	-2.858020266	-1.131301629

84494	6/30/2016 11:54	A	249610388.3	3826254.191	-72.85	-42.508	3074	2.81643624	-2.813346356	-1.276630272
84494	6/30/2016 12:34	B	409280278.5	409280278.5	-72.849	-42.513	2410	2.920637389	-2.750116985	-1.331869827
84494	6/30/2016 14:15	B	145637995	22974713.47	-72.851	-42.517	6068	2.947956008	-2.741648766	-1.341473455
84494	6/30/2016 15:55	B	47270257.76	1025294.743	-72.759	-42.508	5979	0.779844252	-2.607241212	-1.380860969
84494	6/30/2016 19:47	A	110560.4524	2753.547639	-72.908	-42.552	13953	1.978857099	-2.547135451	-0.948197462
84494	6/30/2016 21:25	B	1530418.263	514354.2374	-72.88	-42.55	5884	2.631572194	-2.199980723	-1.155603017
84494	6/30/2016 23:08	A	59542.50187	6765.998135	-72.847	-42.503	6152	2.742752108	-2.839880109	-1.262488357
84494	7/1/2016 0:16	B	4898112605	1768907735	-72.818	-42.475	4120	1.32474498	-2.987227125	-1.124155226
84494	7/1/2016 0:54	B	303332197.9	106387356.6	-72.817	-42.471	2240	1.043206087	-2.982111674	-1.109149428
84494	7/1/2016 1:53	B	16830248.1	5576584.4	-72.816	-42.472	3556	1.203324705	-2.982294368	-1.121854772
84494	7/1/2016 2:31	B	80796646.93	17572109.07	-72.793	-42.51	2304	1.545302179	-2.932238615	-1.349290278
84494	7/1/2016 4:10	B	2890894.738	1083918.262	-72.792	-42.537	5916	0	-2.459030256	-1.408296343
84494	7/1/2016 5:31	B	99548260.23	3853599.768	-72.869	-42.648	4830	2.470337923	-2.415359717	-0.986125744
84494	7/1/2016 7:19	A	114826269.7	25083092.83	-72.94	-42.567	6480	1.08665826	-2.878432984	-0.629922558
84494	7/1/2016 8:23	A	12484105.44	13374787.06	-72.921	-42.588	3857	1.139954513	-2.744042372	-0.60412079
84494	7/1/2016 8:51	B	1022992.431	522362.0692	-72.922	-42.588	1709	1.139954513	-2.744042372	-0.60412079

84494	7/1/2016 10:39	B	916961284.5	65835248.5	-72.894	-42.54	6439	4.887828944	-2.437720485	-1.170513298
84494	7/1/2016 11:21	A	11006923.99	217790.0066	-72.856	-42.556	2518	5.018529219	-1.954567277	-1.382264584
84494	7/1/2016 11:40	B	699083.378	82629.622	-72.852	-42.559	1147	4.739774464	-1.919902338	-1.392673082
84494	7/1/2016 12:19	B	1356304.5	283504.5	-72.843	-42.563	2383	4.755930005	-1.890801197	-1.366365192
84494	7/1/2016 12:56	B	1902191.048	430235.4515	-72.833	-42.569	2186	4.308076849	-1.84520519	-1.369886558
84494	7/1/2016 13:53	A	51297663.81	774500.6945	-72.733	-42.568	3453	0	-1.309989637	0
84494	7/1/2016 14:45	B	4905788.719	658192.281	-72.809	-42.577	3063	0	-1.741025774	-1.32689462
84494	7/1/2016 15:36	B	6434448.446	26254872.05	-72.774	-42.595	3110	0	-1.341554388	0
84494	7/1/2016 19:34	A	49825298.93	30421750.07	-72.851	-42.642	14266	2.174153991	-2.047127305	-1.052194415
84494	7/1/2016 21:22	B	2255956.339	704540.1607	-72.857	-42.662	6501	1.696092823	-2.307362384	-1.038557232
84494	7/1/2016 22:38	1	8699854.158	54562.34211	-72.877	-42.651	4503	2.378644005	-2.531931528	-0.933074913
84494	7/1/2016 23:59	A	2743310.799	25447506.2	-72.866	-42.608	4916	2.88342853	-2.081853523	-0.960339099
84494	7/2/2016 0:17	B	2119280.553	1205175.947	-72.867	-42.606	1058	2.962592016	-2.090882704	-0.961283854
84494	7/2/2016 0:29	B	6688729.178	327837.3215	-72.845	-42.579	714	3.799802994	-1.782254285	-1.212631854
84494	7/2/2016 2:10	0	1756605232	258938456	-72.827	-42.536	6055	3.629017492	-2.443435505	-1.731194466
84494	7/2/2016 3:02	B	103488628	19200448.53	-72.833	-42.53	3106	3.964560767	-2.569516105	-1.729548684

84494	7/2/2016 3:52	B	128261141.8	20762468.16	-72.837	-42.53	3046	4.231887589	-2.500880002	-1.715351526
84494	7/2/2016 5:23	B	267536.1802	1157155.82	-72.887	-42.541	5438	1.1915499	-2.345801573	-1.15272951
84494	7/2/2016 6:59	B	91633011.45	7555545.549	-72.929	-42.59	5783	0.766253897	-2.840912987	-0.736962435
84494	7/2/2016 8:43	A	566145065.1	114606981.4	-72.897	-42.664	6189	0.72468842	-2.722806088	-0.821625366
84494	7/2/2016 9:50	A	17204935.08	948289.4231	-72.881	-42.627	4067	0.901809632	-2.431166624	-0.891531686
84494	7/2/2016 10:12	B	846473.9383	86558.56174	-72.884	-42.626	1277	0.928605044	-2.45060798	-0.867817217
84494	7/2/2016 10:46	B	1346771.751	273632.2486	-72.881	-42.625	2033	0.878139449	-2.416416598	-0.901384026
84494	7/2/2016 11:31	B	2617785.563	237754.937	-72.889	-42.59	2701	1.45992743	-2.265435924	-0.938607641
84494	7/2/2016 11:56	B	41389358	5220030.496	-72.83	-42.547	1504	-0.13084195	-2.184955637	-1.269119392
84494	7/2/2016 12:27	A	622544.767	93267.73299	-72.827	-42.529	1890	0.27682882	-2.583727909	-1.250523101
84494	7/2/2016 13:28	B	172625.6185	827318.8815	-72.837	-42.5	3671	1.003919591	-2.908190411	-1.123231176
84494	7/2/2016 14:26	B	1915591.155	932853.8446	-72.829	-42.467	3462	0.15272635	-2.987227125	-0.968528175
84494	7/2/2016 15:17	B	4416099.693	1879326.307	-72.819	-42.465	3049	0.072362084	-2.976996223	-0.960533015
84494	7/2/2016 19:20	B	4806566056	217162676.2	-72.787	-42.352	14566	0.588028185	-1.475174768	-1.415835488
84494	7/2/2016 21:00	B	20922456.48	1263948.517	-72.848	-42.314	6029	2.188582794	-2.639625265	-1.189140854
84494	7/2/2016 22:25	2	262534.6178	38922.38224	-72.924	-42.416	5078	-0.2803586	-2.987227125	-0.609911369

84494	7/3/2016 0:10	B	15494317.58	2014300.918	-73.082	-42.357	6312	0.775026321	-2.208039293	-0.911611321
84494	7/3/2016 1:32	B	11810263.19	1508863.308	-72.969	-42.411	4911	-0.757541948	-2.987227125	-0.541280325
84494	7/3/2016 2:43	B	276324944.5	78123256.02	-72.877	-42.451	4261	0.80242251	-2.987227125	-0.820641057
84494	7/3/2016 3:25	0	133625699	1600471.472	-72.815	-42.424	2542	1.437482106	-2.713952812	-1.018069823
84494	7/3/2016 8:34	0	2482207.517	43253201.48	-72.878	-42.475	18510	1.095600039	-2.987227125	-0.884647004
84494	7/3/2016 9:39	A	63771929.51	8473756.988	-72.79	-42.5	3941	1.461111865	-2.947143516	-0.813747285
84494	7/3/2016 10:16	A	235903.0838	312333.9162	-72.877	-42.476	2207	1.077584926	-2.987227125	-0.895923724
84494	7/3/2016 11:56	B	87980580.63	279181799.9	-72.799	-42.632	6003	0	-1.316502282	0
84494	7/3/2016 13:18	B	22817241.02	14252975.48	-72.762	-42.692	4889	0	-1.187843678	0
84494	7/3/2016 14:47	B	18920255.93	8942897.068	-72.708	-42.744	5389	0	-0.839821951	0
84494	7/3/2016 20:52	A	236272.6819	1442319.818	-72.923	-42.696	21874	1.493304712	-2.151548151	-0.801641015
84494	7/3/2016 21:36	A	106788.5849	128805.4151	-72.91	-42.677	2631	1.238766269	-2.579571628	-0.776695318
84494	7/3/2016 21:59	A	2211290.869	484141.131	-72.902	-42.679	1384	1.351231946	-2.590126128	-0.821092426
84494	7/3/2016 23:12	B	249924057.6	4937656.872	-72.892	-42.645	4358	1.162187072	-2.713642146	-0.847435916
84494	7/3/2016 23:38	1	83216330.07	13587239.93	-72.874	-42.641	1590	0.942155411	-2.454086535	-0.940061466
84494	7/3/2016 23:45	1	676550.2316	1177409.768	-72.86	-42.642	438	0.755812702	-2.183671668	-0.977799136

84494	7/4/2016 1:22	B	7192026.716	1593741.784	-72.833	-42.639	5810	-0.048896721	-1.783497133	-1.026566535
84494	7/4/2016 2:22	A	356882.7562	2702097.744	-72.829	-42.495	3611	0.862266258	-2.936292354	-0.999829404
84494	7/4/2016 5:04	B	71043910.59	25515019.41	-72.808	-42.399	9691	1.531258846	-2.022512165	-0.983669525
84494	7/4/2016 6:40	A	724488.1392	86788.86081	-72.831	-42.421	5742	0.776016462	-2.764183709	-0.779878078
84494	7/4/2016 8:21	A	31619.6184	9577.3816	-72.799	-42.42	6069	1.307751913	-2.550048485	-0.878113045
84494	7/4/2016 9:29	B	19029440.65	9331427.85	-72.819	-42.392	4069	1.70416966	-2.039407531	-0.963295677
84494	7/4/2016 9:46	B	1402606.737	426977.2634	-72.838	-42.41	1061	1.188985864	-2.665495276	-0.7837454
84494	7/4/2016 11:08	B	3166217.45	357315.0496	-72.839	-42.431	4914	0.284355046	-2.918196536	-0.735003576
84494	7/4/2016 12:49	B	3821140.476	1282131.524	-72.838	-42.424	6054	0.556319026	-2.820403203	-0.742684642
84494	7/4/2016 14:30	B	3434615.208	2557957.292	-72.849	-42.416	6027	0.785996257	-2.819429125	-0.719982652
84494	7/4/2016 20:46	B	411588882.2	38477617.76	-72.895	-42.566	22574	0.560539593	-2.223220081	-0.856406875
84494	7/4/2016 21:31	B	28925931.95	2516180.545	-72.898	-42.568	2689	0.583534846	-2.269578879	-0.853211746
84494	7/4/2016 23:16	B	39552764.12	4321506.38	-72.91	-42.562	6334	0.683703556	-2.486864532	-0.822026405
84494	7/5/2016 1:04	B	843429700.7	200654139.3	-72.888	-42.561	6461	0.458860522	-2.200677689	-0.877538856
84494	7/5/2016 1:11	B	15290890.73	3692179.77	-72.897	-42.578	422	0.62686117	-2.317374254	-0.819839504
84494	7/5/2016 4:52	B	922236.6423	1396707.858	-72.87	-42.486	13265	-0.877569857	-2.955612439	-0.71019522

84494	7/5/2016 6:31	A	58807.60687	3378.893128	-72.874	-42.542	5928	-0.95250057	-2.262059686	-0.692235807
84494	7/5/2016 8:10	0	2787392.486	3104179.514	-72.908	-42.463	5945	-0.785319641	-2.987227125	-0.666182439
84494	7/5/2016 9:01	B	214877450.2	66468345.82	-72.94	-42.479	3054	-0.782690664	-2.987227125	-0.621051282
84494	7/5/2016 9:19	A	106097.6388	29360.3612	-72.903	-42.514	1083	-0.907788488	-2.824069389	-0.668819675
84494	7/5/2016 10:34	1	504256.0851	42872.41495	-72.91	-42.528	4534	-0.901417023	-2.750104483	-0.655512577
84494	7/5/2016 10:58	B	1807216.468	508610.0323	-72.934	-42.515	1450	-0.843113522	-2.939003913	-0.626822315
84494	7/5/2016 11:01	B	1876259.662	514746.8378	-72.937	-42.515	121	-0.832699349	-2.945790884	-0.620865164
84494	7/5/2016 12:32	B	31748942.81	6916937.687	-72.906	-42.531	5505	-0.909139059	-2.677392769	-0.657438095
84494	7/5/2016 18:48	B	8950884.191	2008012.809	-73.004	-42.547	22565	-0.842680556	-2.987227125	-0.525137223
84494	7/5/2016 22:12	A	25937936.82	201477.6832	-72.957	-42.567	12215	-0.908616878	-2.987227125	-0.588525833
84494	7/5/2016 22:48	B	2905486.225	132417.7753	-72.966	-42.57	2145	-0.909415668	-2.987227125	-0.578416867
84494	7/5/2016 23:52	B	4162677.341	307349.1592	-72.982	-42.569	3832	-0.906617129	-2.987227125	-0.559861061
84494	7/6/2016 0:33	B	12411437.85	249407.1482	-72.979	-42.568	2502	-0.902130821	-2.987227125	-0.564294495
84494	7/6/2016 1:01	B	12501298.08	353150.4171	-72.981	-42.568	1663	-0.901772902	-2.987227125	-0.562795392
84494	7/6/2016 3:21	B	19038994.63	1809983.365	-73.016	-42.572	8397	-0.910089475	-2.912833074	-0.521203987
84494	7/6/2016 4:08	B	24596771.33	2163065.671	-73.005	-42.566	2819	-0.431193711	-2.987227125	-1.042892631

84494	7/6/2016 6:15	B	26142856.66	3584504.338	-73.028	-42.567	7615	-0.435567769	-2.914556417	-1.02850714
84494	7/6/2016 9:00	B	27994933.76	2509436.238	-73.031	-42.541	9901	-0.518380168	-2.762638059	-0.966203852
84494	7/6/2016 10:14	B	33408771.19	2575301.311	-73.018	-42.535	4454	-0.522366937	-2.887262184	-0.972938082
84494	7/6/2016 12:01	B	101076612.6	4781620.355	-72.972	-42.459	6408	-0.572291167	-2.987227125	-0.918193509
84494	7/6/2016 12:14	B	107475677.8	3523591.182	-72.97	-42.452	797	-0.563115671	-2.987227125	-0.916605668
84494	7/6/2016 13:52	B	1363793832	112136870	-72.804	-42.37	5899	0.126180277	-1.593343266	-1.384194568
84494	7/6/2016 15:29	B	24129851.48	4886273.519	-72.678	-42.418	5800	0	-1.346931841	-1.735088553
84494	7/6/2016 18:37	B	8979194.822	1112217.678	-72.577	-42.4	11245	0	-0.744713995	0
84494	7/6/2016 20:21	B	22259146.59	3093117.912	-72.525	-42.347	6243	-0.402365212	-0.590871204	-2.32632769
84494	7/6/2016 21:39	B	3645954181	1658791021	-72.751	-42.413	4732	-0.007161237	-1.470281475	-1.654455247
84494	7/6/2016 22:24	A	1302657.243	94345.25733	-72.761	-42.41	2679	0.023477269	-1.875060873	-1.501011172
84494	7/6/2016 23:01	B	873112.0949	1419216.905	-72.714	-42.391	2202	0	-1.248205197	-1.664178675
84494	7/6/2016 23:19	B	2034037.093	402703.907	-72.729	-42.391	1105	0	-1.271857442	-1.61885307
84494	7/7/2016 0:10	B	769801.0992	12263544.9	-72.765	-42.4	3051	0.041519953	-1.650711125	-1.485489331
84494	7/7/2016 0:44	B	1559078.004	1711264.496	-72.728	-42.418	2034	-0.018308949	-1.672401404	-1.614784757
84494	7/7/2016 2:09	B	10234995.43	3620979.071	-72.724	-42.47	5076	-0.156388451	-2.439896286	-1.594360282

84494	7/7/2016 2:56	B	28669927.05	10030279.45	-72.722	-42.469	2842	-0.144702493	-2.396631861	-1.594814348
84494	7/7/2016 3:45	B	14469098.31	13119430.19	-72.738	-42.465	2967	-0.110419186	-2.551895697	-1.559898737
84494	7/7/2016 6:09	B	831391567.8	6365724.738	-72.718	-42.418	8610	-0.761411654	-1.621311541	-2.232347123
84494	7/7/2016 8:10	B	15133297.81	490524.6889	-72.723	-42.433	7249	-0.764827076	-1.821749798	-2.213059035
84494	7/7/2016 9:46	A	23707.35871	20275.14129	-72.706	-42.415	5754	-0.759378294	-1.50548454	-2.248230268
84494	7/7/2016 10:36	A	208141220.1	577172.8997	-72.708	-42.411	3010	-0.759378294	-1.489267741	-2.248230268
84494	7/7/2016 11:22	1	522407.3175	392813.6825	-72.734	-42.414	2776	-0.753137703	-1.628411785	-2.20978025
84494	7/7/2016 11:31	0	142650995.3	5401441.688	-72.745	-42.403	546	-0.743826394	-1.595923766	-2.192306573
84494	7/7/2016 13:13	B	76843196.36	16227936.14	-72.749	-42.408	6101	-0.738636189	-1.696694616	-2.186223408
84494	7/7/2016 13:26	A	740502.9627	210397.0373	-72.76	-42.405	809	-0.73278847	-1.755810775	-2.17317135
84494	7/7/2016 15:08	B	281401063.1	5124513.898	-72.746	-42.568	6085	0	-1.403635361	0
84494	7/7/2016 18:29	B	334395660.5	24472008	-72.745	-42.57	12065	0	-1.405620626	0
84494	7/7/2016 20:06	B	3.2006E+11	9951088364	-72.753	-42.461	5824	-0.772740828	-2.881606032	-2.146053704
84494	7/7/2016 21:48	0	574623.0465	16271466.95	-72.772	-42.435	6124	-0.735140502	-2.504049455	-2.144895812
84494	7/7/2016 22:00	B	1809375.006	8542187.494	-72.756	-42.441	743	-0.752522628	-2.372136689	-2.169931054
84494	7/8/2016 0:29	B	1386481412	86198153.25	-72.795	-42.478	8948	-0.741702972	-2.977361612	-2.093263855

84494	7/8/2016 1:53	B	18761837.73	1552262.77	-72.804	-42.481	4994	-0.73810908	-2.982464463	-2.07758873
84494	7/8/2016 2:36	B	28907243.58	130774.9171	-72.81	-42.456	2617	-0.718169966	-2.962563344	-2.074883274
84494	7/8/2016 3:26	0	4534566.717	862974.2835	-72.843	-42.506	3004	-0.724857066	-2.839880109	-2.007917912
84494	7/8/2016 6:01	B	618848021.7	212614195.3	-72.886	-42.535	9249	-0.815145977	-2.427128326	-1.797716149
84494	7/8/2016 7:40	B	4512355858	174289695.4	-72.901	-42.567	5980	-0.801883089	-2.052461023	-1.814768973
84494	7/8/2016 8:44	B	14854364.54	574857.962	-72.853	-42.558	3801	-0.833842754	-1.940899298	-1.846280429
84494	7/8/2016 10:16	B	5443018.989	574389.5107	-72.861	-42.568	5525	-0.825817606	-1.857182408	-1.847599244
84494	7/8/2016 11:02	B	8702651.214	1091413.786	-72.858	-42.573	2794	-0.826062876	-1.861549542	-1.85290982
84494	7/8/2016 12:00	B	9068180.315	506264.6849	-72.857	-42.576	3448	-0.826062876	-1.874722448	-1.85290982
84494	7/8/2016 12:41	B	6684757.649	18500430.85	-72.864	-42.569	2472	-0.823286566	-1.873250423	-1.844713734
84494	7/8/2016 19:56	B	10116627.24	1292085.259	-72.891	-42.546	26096	-0.807779538	-2.377882163	-1.799078906
84494	7/8/2016 21:35	B	6740256.859	1940467.141	-72.819	-42.517	5941	-0.863106289	-2.86064802	-1.857898926
84494	7/8/2016 22:17	B	131649685.2	1059390.794	-72.796	-42.457	2537	-0.89434983	-2.947765075	-1.876270538
84494	7/9/2016 4:44	B	54773336.8	7733708.197	-72.805	-42.339	23211	-1.104949045	-1.535917936	-1.51768292
84494	7/9/2016 5:41	B	273016708.9	84980456.08	-72.813	-42.313	3437	0	-1.975685056	-1.502553415
84494	7/9/2016 7:21	B	86071740.86	27129047.64	-72.812	-42.26	5973	-1.169304481	-2.770030781	-1.491569086

84494	7/9/2016 10:08	B	250026212.6	4829927.929	-72.863	-42.331	10010	-1.124926856	-2.720934456	-1.478218667
84494	7/9/2016 10:34	B	243693835.3	6827161.171	-72.864	-42.33	1588	-1.125436614	-2.730444908	-1.476892062
84494	7/9/2016 11:46	B	1349075936	4127376.333	-72.855	-42.262	4307	-1.167784919	-2.987227125	-1.468757957
84494	7/9/2016 12:51	B	10130922113	200117531.6	-72.886	-42.345	3909	-1.113739615	-2.452370228	-1.487103954
84494	7/9/2016 14:24	A	1241277.042	1875167.458	-72.812	-42.419	5583	-1.065992439	-2.575719006	-1.545729314
84494	7/9/2016 19:45	A	3009890.364	85895.63617	-72.837	-42.53	19278	-0.989687494	-2.526513283	-1.58864434
84494	7/9/2016 21:25	B	51058285.7	15998100.3	-72.813	-42.57	5964	-0.996730869	-1.839645749	-1.620496852
84494	7/9/2016 22:25	B	3273215277	648858396	-72.864	-42.518	3595	-0.988777903	-2.622335783	-1.57335615
84494	7/9/2016 22:52	B	18496968.43	3851452.574	-72.866	-42.518	1654	-0.986895121	-2.681714203	-1.56543516
84494	7/9/2016 23:27	B	3958223.746	13301170.25	-72.959	-42.563	2069	-0.898196597	-2.987227125	-1.533620882
84494	7/10/2016 0:07	A	173164.0833	50379668.42	-72.951	-42.564	2423	-0.900752651	-2.987227125	-1.541822484
84494	7/10/2016 0:32	B	2950020.5	3894840.5	-72.961	-42.581	1474	-0.875655515	-2.987227125	-1.552201679
84494	7/10/2016 1:01	B	6890438.555	5360651.445	-72.953	-42.566	1775	-0.898656846	-2.987227125	-1.540506062
84494	7/10/2016 2:43	B	13291459.18	5790363.324	-72.96	-42.575	6104	-0.882278563	-2.987227125	-1.54905704
84494	7/10/2016 5:36	A	1093317850	12296443.14	-72.835	-42.89	10363	-1.066582532	-1.633176656	-1.966107617
84494	7/10/2016 7:14	B	280594791.9	3887614.641	-72.807	-42.928	5912	-1.115949912	-1.66886414	-1.998517229

84494	7/10/2016 8:32	B	204118620.2	664151490.3	-72.935	-42.929	4686	-1.056762416	-1.146927814	-1.999806519
84494	7/10/2016 8:53	B	9932073.865	23893752.14	-72.94	-42.938	1236	-1.061771545	-1.146062107	-2.00700807
84494	7/10/2016 10:03	B	11873910.15	12401766.85	-72.955	-42.965	4228	-1.090469558	-1.22485048	-2.032607583
84494	7/10/2016 10:09	B	9421313.073	14830775.43	-72.952	-42.961	335	-1.085300514	-1.220709167	-2.028236171
84494	7/10/2016 11:37	B	32142466.12	484940.3796	-73.085	-42.97	5254	-0.985617226	-1.429914339	-2.073153294
84494	7/10/2016 12:30	B	1508190.343	214906.6568	-73.085	-42.968	3217	-0.985617226	-1.422395729	-2.073153294
84494	7/10/2016 19:34	0	4571690.859	1142273.641	-73.104	-42.591	25400	-0.729810731	-2.368427566	-1.470504635
84494	7/10/2016 21:11	B	467210146.2	224095696.3	-73.116	-42.571	5844	-0.76805044	-2.240373526	-1.434562077
84494	7/10/2016 22:56	B	385607.3187	6974957.681	-73.056	-42.542	6305	-0.867249208	-2.545090898	-1.452728245
84494	7/10/2016 23:55	B	132446288.5	40965.95476	-73.048	-42.54	3546	-0.875447386	-2.523975923	-1.456500823
84494	7/11/2016 0:00	B	8247413.586	16958.41351	-73.049	-42.539	293	-0.875447386	-2.531492736	-1.456500823
84494	7/11/2016 2:19	B	4117388.665	634999.3353	-73.034	-42.514	8345	-0.924885604	-2.492685244	-1.449115336
84494	7/11/2016 9:43	B	16492761.8	1691031.197	-73.042	-42.515	26624	-0.995158122	-2.415458693	-1.527913921
84494	7/11/2016 11:22	B	58519908.51	4871149.492	-73.049	-42.519	5966	-0.990162152	-2.307556447	-1.524015226
84494	7/11/2016 12:10	B	9153204.568	7611275.432	-73.023	-42.49	2856	-1.034999392	-2.555448613	-1.525494996
84494	7/11/2016 21:03	Z	2741517013	108999289.9	-72.835	-42.439	32001	-1.106103838	-2.957630587	-1.631057056

84494	7/11/2016 22:25	B	361782464.2	111179228.8	-72.699	-42.471	4917	-1.104445249	-2.066423359	-1.708328002
84494	7/12/2016 1:20	B	13108921.24	13937216.76	-72.648	-42.385	10496	0	-0.915079198	0
84494	7/12/2016 6:55	B	20074580.1	5994584.9	-72.483	-42.326	20087	-1.037462279	-0.549419015	-1.881256542
84494	7/12/2016 9:22	B	545421090.8	357748466.2	-72.83	-42.274	8849	-0.917039993	-2.777787793	-1.625729948
84494	7/12/2016 11:18	B	87991262.33	53203590.17	-72.831	-42.274	6922	-0.912682996	-2.897467411	-1.623199569
84494	7/12/2016 12:16	A	65391.20904	1338465.291	-72.828	-42.222	3521	-0.919471007	-2.987227125	-1.581615616
84494	7/12/2016 13:23	B	10356431.97	3417133.03	-72.863	-42.183	3997	-0.921584391	-2.987227125	-1.521309911
84494	7/12/2016 14:22	B	19998354.63	12440382.37	-72.838	-42.218	3553	-0.918551996	-2.987227125	-1.568532818
84494	7/12/2016 15:06	B	18067092.1	15241332.4	-72.859	-42.212	2644	-0.914193334	-2.987227125	-1.548065139
84494	7/12/2016 19:15	B	8333334.798	1222237.202	-72.778	-42.321	14934	0	-1.387147855	-1.711134688
84494	7/12/2016 20:52	B	23181410.76	1007073.736	-72.744	-42.363	5831	0	-1.158207311	-1.770363591
84494	7/12/2016 21:56	B	53173203.7	365542.3045	-72.737	-42.38	3826	0	-1.243343434	-1.804860923
84494	7/12/2016 22:31	B	54601250	356168	-72.731	-42.378	2090	0	-1.195767149	-1.811665885
84494	7/12/2016 23:33	B	122235813.2	12936898.83	-72.759	-42.37	3694	-0.90426707	-1.27773517	-1.774189995
84494	7/13/2016 0:56	B	16561735.52	981526.9775	-72.758	-42.374	4996	-0.90426707	-1.319805163	-1.777295845
84494	7/13/2016 1:48	B	13842744.24	957230.2622	-72.765	-42.378	3132	-0.908167931	-1.360295568	-1.780974495

84494	7/13/2016 3:25	B	70932114.5	212790718.5	-72.752	-42.435	5796	-0.927693075	-2.216550992	-1.842957947
84494	7/13/2016 6:42	B	2827793.405	1330239.095	-72.782	-42.499	11859	-0.713973792	-2.867847765	-2.209654518
84494	7/13/2016 8:59	B	3638565.314	3909501.186	-72.759	-42.498	8228	-0.790031389	-2.748320486	-2.211991606
84494	7/13/2016 9:26	B	33280672.42	16355567.58	-72.722	-42.445	1606	-0.766118774	-2.001506545	-2.138004898
84494	7/13/2016 10:08	B	13438360.82	5120348.178	-72.769	-42.406	2485	-0.4346902	-1.842277768	-2.050322641
84494	7/13/2016 10:38	B	3258278.413	248414.0871	-72.823	-42.358	1852	-0.022622668	-1.794446324	-1.862694129
84494	7/13/2016 11:43	B	147426408.9	10588416.08	-72.835	-42.394	3870	-0.012645569	-2.427005465	-1.950496085
84494	7/13/2016 22:22	Z	343535924.1	2014363.949	-72.708	-42.494	38319	-0.898575128	-2.122529078	-2.211831157
84494	7/13/2016 22:49	B	9586970450	11704.5	-72.76	-42.497	1635	-0.818213979	-2.673994772	-2.211183863
84494	7/14/2016 0:28	B	218083897.3	18545555.16	-72.86	-42.468	5922	-0.087962628	-2.987227125	-2.146201183
84494	7/14/2016 1:01	B	214631054.8	19949719.71	-72.838	-42.475	1991	-0.263400983	-2.987227125	-2.175634468
84494	7/14/2016 2:17	B	51586609.59	1476258.91	-72.887	-42.478	4597	0.188691682	-2.987227125	-2.160921865
84494	7/14/2016 2:58	B	18393118644	1143398501	-72.857	-42.5	2467	-0.023155052	-2.869611842	-2.241274102
84494	7/14/2016 4:39	B	24909133.56	2089892.443	-72.879	-42.532	6006	-0.50440216	-2.427704497	-2.193084287
84494	7/14/2016 6:27	B	78264971.59	254713046.4	-72.945	-42.532	6522	1.296518688	-2.958293198	-2.174486751
84494	7/14/2016 8:11	B	2768511.031	3987027.469	-72.962	-42.529	6239	1.953239868	-2.987227125	-2.086310135

84494	7/14/2016 9:34	B	4692031.352	530393.6476	-72.977	-42.504	4957	1.763285	-2.96144382	-1.856841465
84494	7/14/2016 10:05	B	5600842.984	605629.5161	-72.976	-42.496	1890	1.554019137	-2.96144382	-1.80919845
84494	7/14/2016 10:53	B	12796054.94	287257.5567	-72.983	-42.466	2850	0.963340178	-2.895274555	-1.655578064
84494	7/14/2016 11:50	B	9744920.087	507152.9134	-72.983	-42.46	3448	0.696378488	-2.904882187	-1.618468298
84494	7/14/2016 12:53	B	17216550.85	427491.1539	-72.993	-42.427	3755	-0.125629047	-2.763084306	-1.537849398
84494	7/14/2016 13:43	B	16842004.77	583912.2294	-73	-42.413	3008	-0.154553413	-2.715280941	-1.504825871
84494	7/14/2016 14:25	B	22237090.21	355330.2912	-73	-42.398	2545	0.105866722	-2.809840975	-1.486614663
84494	7/14/2016 18:48	A	197765.9175	528394.0825	-72.915	-42.405	15751	1.276522744	-2.982884049	-1.923536053
84494	7/14/2016 20:29	B	154111999.1	60175507.45	-72.932	-42.366	6057	1.492423156	-2.987227125	-1.65016509
84494	7/14/2016 20:56	B	2758436.85	935188.1504	-72.947	-42.375	1605	1.316141229	-2.987227125	-1.668188884
84494	7/14/2016 22:10	B	3342186.574	718889.9264	-72.952	-42.366	4442	1.151799411	-2.987227125	-1.593101084
84494	7/14/2016 22:20	B	2384325.5	1340055.5	-72.96	-42.367	627	1.043410962	-2.987227125	-1.576508044
84494	7/14/2016 23:07	2	4122368.777	341603.7229	-73.051	-42.404	2815	0.028947261	-2.039194953	-1.404651747
84494	7/15/2016 0:05	B	184248256.9	2037795.055	-73.075	-42.385	3451	0.31522773	-2.139375794	-1.299166536
84494	7/15/2016 0:12	B	8333333.881	104940.1185	-73.074	-42.386	436	0.318826396	-2.138904224	-1.309318737
84494	7/15/2016 0:50	B	3302404.753	515656.2466	-73.066	-42.394	2268	0.269938919	-2.103362631	-1.338407254

84494	7/15/2016 1:01	B	2117753.327	295848.6734	-73.057	-42.389	665	0.127131249	-2.265901846	-1.347176254
84494	7/15/2016 1:59	B	2131896.567	488268.4326	-73.05	-42.388	3489	0.108184772	-2.314315029	-1.35082059
84494	7/15/2016 2:40	A	26375498850	521072682.5	-73.134	-42.439	2495	0.498892573	-1.418733234	-1.464348053
84494	7/15/2016 3:35	B	545603456.6	40118891.93	-73.157	-42.464	3243	1.048798345	-1.542900257	-1.214073048
84494	7/15/2016 4:17	B	249178853.7	19970311.34	-73.183	-42.479	2563	0.725771628	-1.827597664	-1.139057862
84494	7/15/2016 6:14	B	323140115.6	63766088.91	-73.25	-42.513	6982	0.053589202	-1.528869284	-1.036828838
84494	7/15/2016 8:01	B	416248786.5	15777925.99	-73.234	-42.486	6449	0.384542276	-1.657436216	-1.109957606
84494	7/15/2016 10:38	B	18329206.55	728718.4495	-73.091	-42.449	9421	0.80645596	-1.433478941	-1.644051182
84494	7/15/2016 11:32	B	5055027.453	1129897.547	-73.022	-42.43	3210	-0.491662093	-2.258973124	-1.533580761
84494	7/15/2016 12:28	B	6140984.529	1206304.471	-73.017	-42.419	3362	-0.458321256	-2.422658839	-1.523401916
84494	7/15/2016 14:12	B	5900465.804	1774616.696	-72.964	-42.4	6242	1.428711061	-2.987227125	-1.710054831
84494	7/15/2016 18:33	B	134111252.8	1153059.721	-72.956	-42.375	15684	1.679202258	-2.987227125	-1.719407671
84494	7/15/2016 21:58	B	14315716.16	1232542.344	-72.888	-42.333	12316	1.647316225	-2.889125611	-1.628015917
84494	7/16/2016 1:22	B	2621535.389	3815415.111	-72.942	-42.354	12245	1.914892201	-2.987227125	-1.62481427
84494	7/16/2016 3:19	B	45210927.92	11190288.08	-72.943	-42.343	6978	1.634314685	-2.987227125	-1.545759685
84494	7/16/2016 4:00	B	34637994.61	24703153.89	-73.003	-42.353	2485	0.220722058	-2.950875379	-1.479454486

84494	7/16/2016 7:50	B	71857510.47	31052089.53	-72.991	-42.389	13766	0.349913262	-2.880183758	-1.607562185
84494	7/16/2016 8:46	B	56777086.81	20444349.69	-73.092	-42.462	3399	-0.225490175	-1.498533382	-1.532670537
84494	7/16/2016 9:21	B	63546182.75	12399041.75	-73.115	-42.485	2098	0.545683965	-1.601364005	-1.420923741
84494	7/16/2016 11:51	1	1602864.626	1336809.374	-72.962	-42.591	8988	0.113273092	-2.987227125	-1.867303482
84494	7/16/2016 12:07	B	12983664.24	3861450.258	-72.962	-42.591	962	0.115454604	-2.987227125	-1.868159432
84494	7/16/2016 13:45	B	133418942.7	13434530.31	-72.86	-42.598	5873	-0.542049883	-1.886627361	-1.962528835
84494	7/16/2016 20:05	A	1395388994	63066150.35	-73.102	-42.478	22816	-0.183388358	-1.618983791	-1.564313103
84494	7/16/2016 21:28	B	641744964.2	338760364.8	-72.868	-42.41	4949	-0.037873176	-2.964163896	-2.077601946
84494	7/16/2016 22:50	B	91764812.39	139887628.1	-72.835	-42.47	4933	-1.057642098	-2.987227125	-2.138285945
84494	7/16/2016 23:07	B	86106034.24	433146.2598	-72.848	-42.451	1020	-1.086971882	-2.987227125	-2.145988294
84494	7/16/2016 23:18	B	149273430.2	7957672.282	-72.848	-42.444	677	-1.073367118	-2.987227125	-2.146939738
84494	7/17/2016 0:37	B	97671014.65	125710.3457	-72.734	-42.456	4714	-0.747681363	-2.386504606	-2.142348037
84494	7/17/2016 0:55	B	10986803.48	88839.01747	-72.764	-42.453	1090	-0.707661446	-2.615648376	-2.138543186
84494	7/17/2016 2:53	B	121463370.7	13272261.26	-72.961	-42.56	7106	-0.012810734	-2.987227125	-1.947233548
84494	7/17/2016 3:40	A	511306.5833	103898.4167	-73.042	-42.564	2805	0.858617891	-2.80781894	-1.64617531
84494	7/17/2016 5:54	B	24516820.7	3589455.796	-73.118	-42.631	8007	-0.97383438	-2.153384489	-1.48742821

84494	7/17/2016 7:33	B	1374872569	439696.8607	-73.145	-42.775	5979	-0.917727591	-0.589233396	-1.507923386
84494	7/17/2016 10:15	B	105278354.2	3491616.274	-73.223	-42.885	9717	-0.437371324	-0.469178817	-1.71872279
84494	7/17/2016 11:16	B	113073414.8	4395889.72	-73.227	-42.905	3633	-0.495715068	-0.510926785	-1.763751543
84494	7/17/2016 11:44	B	35299959.5	3912388.998	-73.184	-42.868	1728	-0.549656179	-0.511440967	-1.661868152
84494	7/17/2016 13:23	B	550276546.1	290472752.4	-72.987	-42.831	5922	-1.23683062	-0.684087851	-1.595229017
84494	7/17/2016 19:50	A	50342651.58	1130000.923	-72.827	-43.004	23197	-0.817236001	-2.42526376	-1.871409486
84494	7/17/2016 21:35	B	21680372.9	1405355.104	-72.949	-42.969	6306	-0.701871379	-1.252724696	-1.743468587
84494	7/17/2016 22:37	B	24387766.6	284858.3951	-72.939	-42.901	3706	-0.932717963	-1.027371861	-1.653490514
84494	7/18/2016 4:58	B	109012867.6	17852940.4	-72.768	-42.742	22908	0	-1.134406302	0
84494	7/18/2016 5:40	B	96246692.33	7695320.169	-72.711	-42.78	2502	0	-0.783230698	0
84494	7/18/2016 11:48	B	218851127.4	22046786.56	-73.434	-43.265	22083	-0.371455686	-0.641885314	-1.829626562
84494	7/18/2016 12:27	B	6504989.477	1089511.023	-73.459	-43.248	2302	-0.739830534	-0.714888104	-1.890592215
84494	7/18/2016 13:05	1	822267.0286	244965.4714	-73.435	-43.221	2293	-0.572640971	-0.877852404	-1.851556354
84494	7/18/2016 13:57	B	7379184.097	705624.9032	-73.454	-43.233	3155	-0.750660011	-0.791097634	-1.888275551
84494	7/18/2016 14:41	B	2210327072	45000	-73.446	-43.247	2621	-0.662742243	-0.718990337	-1.87804698
84494	7/18/2016 19:42	B	8209719.497	1098289.503	-73.559	-43.403	18033	-0.552280146	-0.300630591	-1.74053152

84494	7/18/2016 22:22	B	99826577.43	950859.0652	-73.902	-43.531	9652	-0.829344249	-1.31558281	-1.429165568
84494	7/19/2016 2:59	A	2.13863E+11	9665457768	-74.323	-43.5	16576	-1.115671253	-1.096997001	-1.309820983
84494	7/19/2016 4:36	B	6097131658	573120590.7	-74.498	-43.498	5834	-0.474436626	-1.137583252	-1.09130616
84494	7/19/2016 9:49	B	3.37206E+11	10484962868	-74.506	-43.397	18793	-0.508090401	-0.982373728	-1.012554586
84494	7/19/2016 11:38	B	3639239830	1654647.155	-74.459	-43.464	6502	-0.517291879	-0.964632191	-0.984859047
84494	7/19/2016 13:37	B	9671961109	300713987.3	-74.701	-43.358	7165	-0.654572335	-2.253057232	-0.845184977
84494	7/19/2016 14:17	B	928219.677	191756.323	-74.701	-43.362	2399	-0.581372399	-2.1667865	-0.794167148
84494	7/19/2016 19:32	A	295958.3941	1949362.606	-74.849	-43.203	18920	-0.665227257	-0.528976675	-0.743238997
84494	7/19/2016 23:20	B	36040337.15	10475851.35	-75.221	-43.149	13663	-0.675065794	0.080975791	-0.284608923
84494	7/20/2016 4:14	A	29573979.65	6359012.846	-74.992	-42.951	17661	0.51803738	-0.285178668	-0.618069373
84494	7/20/2016 5:21	B	171668799.2	7541175.325	-75.021	-43.041	4004	0.009388486	-0.282307584	-0.520051081
172007	2/4/2019 2:36	B	3716344.794	1520143.706	-73.538	-44.309	686	-0.662117441	-0.787952635	2.272838007
172007	2/4/2019 3:41	B	5921998.559	18191013.94	-73.516	-44.311	3938	-0.621085464	-1.499921069	2.4604766
172007	2/4/2019 4:14	B	3732037.481	4194252.519	-73.518	-44.31	1989	-0.574775723	-1.503420019	2.469221978
172007	2/4/2019 6:34	B	3595671.462	673690.5384	-73.504	-44.284	8352	-0.320374623	-1.859755992	2.525764029
172007	2/4/2019 8:13	B	6035912.003	876828.4972	-73.517	-44.251	5956	-0.29340175	-2.262375038	2.585162162

172007	2/4/2019 9:53	B	5517918.036	1473386.464	-73.538	-44.242	6027	-0.222881644	-2.302039901	2.569011839
172007	2/4/2019 11:53	B	637967309.9	780936.5912	-73.542	-44.21	7171	-0.486447231	-2.191767936	2.637011081
172007	2/4/2019 12:04	B	17203879.68	928488.3241	-73.509	-44.211	663	-0.539682493	-2.341330726	2.640780618
172007	2/4/2019 13:24	A	341087967.1	15410855.42	-73.409	-44.202	4833	-0.362379159	-2.866799476	2.497977561
172007	2/4/2019 13:56	B	1609957.276	506915.2244	-73.356	-44.22	1922	-0.634190292	-2.866799476	2.434124017
172007	2/6/2019 1:58	B	74149522.7	2630293.798	-73.579	-43.997	129671	-0.228167818	-0.596842506	1.297332133
172007	2/6/2019 2:54	B	125989292.1	135090000.4	-73.561	-43.984	3404	-0.228443862	-0.553585825	1.333298918
172007	2/6/2019 3:38	B	13141898.5	150838.5041	-73.571	-43.965	2591	-0.154708758	-0.486446315	1.358086125
172007	2/6/2019 7:53	B	70525591.67	195336933.3	-73.637	-43.938	15314	0.062746448	-0.344762365	1.283255305
172007	2/6/2019 9:37	B	6827339.474	518725.0258	-73.612	-43.971	6238	-0.170925605	-0.419709377	1.291482362
172007	2/6/2019 10:49	B	31211556.11	248906.3901	-73.626	-43.987	4326	-0.164850301	-0.425679334	1.250242484
172007	2/6/2019 11:12	A	697934707.8	31541212.22	-73.626	-43.985	1395	-0.164850301	-0.430645158	1.250242484
172007	2/6/2019 11:52	B	4394881.59	389854.9101	-73.616	-43.982	2395	-0.185698232	-0.442481308	1.264426766
172007	2/6/2019 12:41	3	117368.0944	43601.90561	-73.571	-44.018	2969	-0.137385659	-0.680222086	1.26154172
172007	2/6/2019 12:53	B	245769.63	63288.36995	-73.572	-44.018	687	-0.134608091	-0.680222086	1.263292906
172007	2/6/2019 13:13	B	1400052.786	156263.7137	-73.569	-44.027	1199	-0.133734417	-0.748518986	1.25138178

172007	2/6/2019 13:27	B	1304392.584	210639.9163	-73.568	-44.026	848	-0.133734417	-0.748518986	1.25138178
172007	2/6/2019 14:58	B	15273709.8	1730038.705	-73.569	-44.038	5475	-0.200468128	-0.759507896	1.222788946
172007	2/8/2019 1:14	B	25286544421	1572436272	-74.393	-43.884	123327	2.338030006	0.152272032	1.361949642
172007	2/8/2019 1:41	B	40683047.02	451084.9822	-74.376	-43.87	1611	2.224915676	0.140368442	1.414586485
172007	2/8/2019 2:15	1	30847344.37	1924248.634	-74.466	-43.868	2037	1.329053449	0.069893535	1.512568875
172007	2/8/2019 2:49	A	54867259.38	277763.1167	-74.478	-43.861	2066	1.246385414	0.030377736	1.544054882
172007	2/8/2019 3:18	B	2637190.919	111423.5808	-74.487	-43.857	1718	1.126594716	0.003180131	1.574120883
172007	2/8/2019 3:30	B	2293434.309	168268.1914	-74.487	-43.858	744	1.134998992	0.007420142	1.564958606
172007	2/8/2019 3:56	B	2027004.232	302764.7677	-74.51	-43.851	1568	0.725200145	-0.042705893	1.589935888
172007	2/8/2019 7:30	2	389345.1064	340114.8936	-74.722	-43.843	12821	0.893189585	-0.416370573	1.686641505
172007	2/8/2019 9:06	1	80735520.07	3650484.927	-74.768	-43.854	5758	1.415684152	-0.456456509	1.955742324
172007	2/8/2019 9:52	B	8158774.354	1512347.646	-74.792	-43.853	2787	0.949673248	-0.513800488	2.109670275
172007	2/8/2019 10:26	A	2449427.316	155497.6838	-74.831	-43.868	2010	-0.335553043	-0.527697951	2.242650139
172007	2/8/2019 11:24	B	1741862.21	313942.2902	-74.877	-43.874	3507	-0.590700206	-0.542535403	2.228136217
172007	2/8/2019 13:04	B	2987779.365	763805.6345	-74.877	-43.874	6006	-0.578271947	-0.545864326	2.226702546
172007	2/8/2019 13:40	B	23894861782	471966042.8	-74.867	-43.835	2125	-0.119064449	-0.692579985	2.197439657

172007	2/8/2019 14:20	B	97490955.5	1889321.002	-74.876	-43.822	2446	0.34969014	-0.867401182	2.124564824
172007	2/10/2019 11:10	B	16502248069	1679865783	-74.847	-44.428	161387	0.727642984	-1.301892339	1.610351391
172007	2/10/2019 12:43	B	861472445.6	39543107.38	-74.953	-44.564	5559	0.679263037	-1.738157892	1.646726085
172007	2/10/2019 13:36	A	5562527.55	17256.95036	-75.098	-44.552	3188	0.306758668	-1.48177451	1.678704544
172007	2/10/2019 14:41	B	8912987.639	2578081.361	-75.143	-44.544	3919	-0.256075184	-1.877054227	1.753774864
172007	2/12/2019 3:09	B	60486482641	2732801621	-76.568	-44.474	131285	0.594454304	-0.627631441	2.723398787
172007	2/12/2019 12:40	B	55179278.51	4479526.489	-75.928	-43.911	34250	-0.283028341	-0.122149195	2.90166694
172007	2/14/2019 3:32	A	82676862.95	822331.0474	-74.416	-43.41	139888	-0.126691793	-0.361037232	1.412059855
172007	2/14/2019 8:01	B	772109552.6	274537392.4	-74.263	-43.186	16173	-0.888621756	-0.578481427	1.115285053
172007	2/14/2019 11:14	B	2399102811	49165943.75	-74.264	-43.373	11574	-0.79348257	-0.20517894	1.113843968
172007	2/14/2019 13:40	A	446870189.4	16912123.05	-74.298	-43.454	8760	-0.249765265	-0.217237729	1.217148561
172007	2/14/2019 15:35	B	44245792.88	2701009.624	-74.175	-43.403	6873	-0.679979776	-0.360108055	1.11687661
172007	2/14/2019 21:19	B	331570497.3	9903232.651	-74.303	-43.478	20682	-0.159744455	-0.191990575	1.292277987
172007	2/16/2019 3:28	B	107244290.7	1145724898	-73.84	-43.545	108483	0.346399063	-0.493245245	2.890853028
172007	2/16/2019 9:15	B	22957264730	112300871.5	-73.865	-43.582	20856	0.266389503	-0.353142987	2.984693244
172007	2/16/2019 11:34	B	361645627.4	5588108.576	-73.865	-43.583	8351	0.255212847	-0.341304458	3.00143775

172007	2/16/2019 12:04	B	98137774.28	595078.2196	-73.802	-43.585	1763	0.248486204	-0.322034629	3.023674112
172007	2/16/2019 14:14	B	2078745994	374550622.7	-73.833	-43.564	7797	0.34343732	-0.415406644	2.958610758
172007	2/16/2019 14:48	B	94698845.81	15609996.69	-73.826	-43.562	2092	0.371326885	-0.445550575	2.93531418
172007	2/16/2019 21:59	B	131066729.7	166399.2751	-73.827	-43.528	25858	0.511509031	-0.617750843	2.795455321
172007	2/18/2019 2:46	A	274724.944	12737.55605	-74.018	-43.631	103609	-0.135833861	-0.183605165	1.329082714
172007	2/18/2019 4:28	B	14995155.26	13040621.24	-74.009	-43.627	6095	-0.138460987	-0.195344294	1.312593882
172007	2/18/2019 11:18	A	434226.9732	11713.52677	-74.097	-43.75	24589	0.194318873	0.138194252	1.46372505
172007	2/18/2019 12:33	B	17406698.04	5163324.462	-74.09	-43.753	4528	0.204188237	0.134917073	1.438471672
172007	2/18/2019 12:55	B	54071527.02	1268133.976	-74.076	-43.732	1297	0.068024434	0.0993756	1.426124049
172007	2/18/2019 13:35	B	6068087.671	289430.8293	-74.062	-43.723	2403	-0.012579202	0.075415707	1.391976577
172007	2/18/2019 21:12	B	18558303.55	1217690.945	-73.995	-43.689	27429	-0.444711483	-0.036207903	1.260926723
172007	2/18/2019 22:36	A	245472.2107	38369.78926	-74.103	-43.757	5038	0.294407154	0.147558993	1.45909905
172007	2/18/2019 23:30	B	8500651.754	2217905.246	-74.113	-43.765	3235	0.449370552	0.167501716	1.463858936
172007	2/20/2019 0:43	B	82959910010	19734764983	-74.381	-43.589	90805	-1.005469161	-0.034392426	1.436226844
172007	2/20/2019 1:31	B	799080858.6	210173049.4	-74.408	-43.598	2844	-0.990418398	-0.053003205	1.43796331
172007	2/20/2019 2:19	B	352839675.7	97901936.76	-74.416	-43.602	2896	-0.965642093	-0.069466192	1.440025565

172007	2/20/2019 2:35	B	411222890.9	89168301.6	-74.415	-43.605	943	-0.963444002	-0.071664789	1.441429597
172007	2/20/2019 4:49	B	22820434.89	3293555.606	-74.474	-43.621	8044	-0.470401869	-0.104825939	1.457609026
172007	2/20/2019 10:21	B	79405593.12	4810299.884	-74.568	-43.606	19963	0.498615194	-0.273681016	1.375699657
172007	2/20/2019 12:03	B	33922195.86	696918.1393	-74.541	-43.599	6078	0.474573901	-0.27201674	1.355007386
172007	2/20/2019 12:53	B	10372919879	1868985498	-74.636	-43.572	3014	0.532221148	-0.365301719	1.372196586
172007	2/20/2019 22:27	B	65397916.28	8907228.716	-75.116	-43.519	34443	-0.419248893	-2.475012591	2.839028321
172007	2/20/2019 23:14	B	745304288.7	19697416.29	-75.159	-43.507	2823	-0.452513628	-2.614936479	2.838493614
172007	2/22/2019 4:08	B	122513877.3	24809468685	-74.411	-43.501	104051	5.012241734	-0.3974317	0.592372537
172007	2/22/2019 9:44	A	5027849396	156508116.2	-74.386	-43.4	20159	4.666377321	-0.75251701	0.699891987
172007	2/22/2019 10:54	2	544079.5797	19872.92033	-74.546	-43.362	4168	3.268348197	-0.83751341	0.553984909
172007	2/22/2019 11:18	A	4554678.852	380395.1483	-74.548	-43.367	1451	3.41517602	-0.875397252	0.583071009
172007	2/22/2019 12:10	B	6294239.052	1462485.448	-74.576	-43.324	3120	1.591616898	-0.875997232	0.443764181
172007	2/22/2019 13:53	B	8077889.851	2463542.649	-74.572	-43.331	6204	1.849951576	-0.917631207	0.466231479
172007	2/22/2019 15:31	B	23761855.15	4493397.346	-74.629	-43.39	5861	2.314243136	-1.617276305	1.180043173
172007	2/22/2019 20:27	B	1832546.578	11319146.42	-74.503	-43.33	17780	1.941169946	-0.609659005	0.360484784
172007	2/22/2019 22:12	B	39953125327	441370691.9	-74.456	-43.517	6296	0.662625384	-0.46248887	1.251189801

172007	2/22/2019 22:14	B	1554138263	17251937.87	-74.456	-43.517	104	0.67687261	-0.451967558	1.250110848
172007	2/24/2019 0:42	B	190178123.9	672093362.6	-74.57	-43.569	95272	-0.259191969	-0.377733637	0.826066491
172007	2/24/2019 1:44	B	29943225.15	40235143.85	-74.573	-43.574	3706	-0.316930583	-0.366760931	0.818790962
172007	2/24/2019 2:25	B	16017510.92	24357814.08	-74.561	-43.575	2456	-0.254679683	-0.367243277	0.829071173
172007	2/24/2019 2:55	B	378564316	39966964.03	-74.588	-43.543	1799	0.009288283	-0.448743104	0.872751977
172007	2/24/2019 3:23	B	6397472.692	686531.3081	-74.57	-43.542	1706	0.071050273	-0.476103408	0.899734228
172007	2/24/2019 3:37	B	7181342.552	642542.4482	-74.59	-43.546	816	0.303688035	-0.487428982	0.911679492
172007	2/24/2019 4:07	B	5905217.506	566996.9942	-74.587	-43.546	1802	0.285240097	-0.487428982	0.905096427
172007	2/24/2019 7:40	B	266412196.7	9073089.824	-74.487	-43.434	12799	0.258983024	-0.697227088	0.602714137
172007	2/24/2019 9:23	B	135447122.3	14979604.22	-74.508	-43.431	6169	0.197761141	-0.691867495	0.603278448
172007	2/24/2019 9:47	B	164161090.2	8651352.289	-74.479	-43.435	1449	0.251471164	-0.689174932	0.604709743
172007	2/24/2019 11:41	A	62129321.99	35287463.01	-74.596	-43.497	6849	0.452026912	-0.769170886	0.957585853
172007	2/24/2019 12:03	B	7527550.144	2327576.356	-74.6	-43.501	1319	0.222072374	-0.743821787	1.001872489
172007	2/24/2019 12:09	B	8256141.698	967492.8018	-74.602	-43.501	368	0.216246831	-0.729555225	1.009988845
172007	2/24/2019 13:15	B	2525783.982	423682.0184	-74.612	-43.51	3982	0.186242502	-0.705503554	1.040154443
172007	2/24/2019 13:18	B	2573906.35	430044.1502	-74.612	-43.509	139	0.176254211	-0.705503554	1.039971976

172007	2/24/2019 13:42	A	170439911.2	892288.7775	-74.613	-43.469	1439	1.092905237	-1.045339377	0.972044549
172007	2/24/2019 14:48	B	171846121.5	14139877.04	-74.63	-43.478	3948	0.900647431	-1.004389119	1.025806314
172007	2/24/2019 21:11	2	4445740.629	114801.8708	-74.605	-43.526	22979	0.235392473	-0.615552497	1.001864434
172007	2/24/2019 21:49	B	1456288.252	173936.7477	-74.609	-43.529	2282	0.346356895	-0.589102183	0.996002754
172007	2/26/2019 1:29	B	4357459259	164643805.9	-74.474	-43.542	99629	-0.784858898	-0.157076452	0.814547504
172007	2/26/2019 2:50	B	122013319.3	3959900.718	-74.376	-43.521	4842	-0.601891054	-0.136891596	0.798635482
172007	2/26/2019 10:30	B	5460365483	6661075.664	-74.244	-43.465	27642	0.593753385	-0.143086616	0.351647974
172007	2/26/2019 10:43	B	143342405.3	427667.6644	-74.241	-43.465	769	0.503462765	-0.147041068	0.335897352
172007	2/26/2019 11:09	B	44887934.37	177918.1343	-74.269	-43.471	1548	0.770528851	-0.133433696	0.383681954
172007	2/26/2019 11:23	B	22598109.25	158163.248	-74.277	-43.47	828	0.742379342	-0.126066271	0.399597435
172007	2/26/2019 12:06	B	2489211060	262794722.9	-74.365	-43.516	2609	0.636596037	-0.08705877	0.622745324
172007	2/26/2019 12:33	B	104352299.5	10296422.96	-74.379	-43.51	1575	0.124439812	-0.100619432	0.721148376
172007	2/26/2019 13:02	0	156665150.4	20773606.1	-74.353	-43.486	1739	0.235019878	-0.128186054	0.717674535
172007	2/26/2019 13:42	B	741334603.7	6049362.833	-74.355	-43.508	2437	-0.182482972	-0.131175604	0.759968023
172007	2/26/2019 14:09	A	9241556.207	1363504.293	-74.369	-43.509	1603	-0.318798513	-0.134584394	0.775016805
172007	2/26/2019 14:37	B	1221915.418	200987.0817	-74.367	-43.509	1665	-0.311914375	-0.132521775	0.773999258

172007	2/26/2019 14:41	B	125182388.1	4759444.444	-74.354	-43.506	261	-0.232995633	-0.136297555	0.767687882
172007	2/26/2019 21:26	B	17522389.13	1678115.872	-74.331	-43.516	24297	0.220252076	-0.073209663	0.690701306
172007	2/28/2019 0:41	0	117117080.7	50036308.29	-74.236	-43.573	98121	-0.623436686	-0.077300849	0.707478725
172007	2/28/2019 0:57	B	184257039.6	47729932.89	-74.243	-43.574	960	-0.623436686	-0.084570631	0.707478725
172007	2/28/2019 1:04	B	258509867.2	46464055.29	-74.242	-43.574	393	-0.623436686	-0.085754828	0.707478725
172007	2/28/2019 1:59	0	9919351.652	1043685.348	-74.218	-43.566	3317	-0.586271571	-0.116171857	0.707577188
172007	2/28/2019 2:21	B	45651001.11	11661661.39	-74.218	-43.566	1283	-0.613944461	-0.116263568	0.709075358
172007	2/28/2019 2:38	B	3783012252	617565448.1	-74.213	-43.58	1037	-0.692997609	-0.113376513	0.717412649
172007	2/28/2019 2:51	B	1113613115	841552647.3	-74.203	-43.578	793	-0.692997609	-0.107577338	0.717412649
172007	2/28/2019 3:41	B	55744553.78	617040.7176	-74.203	-43.578	3006	-0.749941192	-0.113246408	0.72831873
172007	2/28/2019 4:19	B	2930175.185	129730.8152	-74.205	-43.578	2294	-0.762528342	-0.113246408	0.729487852
172007	2/28/2019 6:56	B	6140806.294	542506.206	-74.21	-43.577	9393	-0.737222535	-0.104835655	0.72499496
172007	2/28/2019 8:44	0	63910987.78	525362.7185	-74.199	-43.627	6488	-0.181959516	-0.006597215	0.660862819
172007	2/28/2019 10:16	B	20427840.56	1795325616	-74.302	-43.646	5487	-0.068197428	0.081429221	0.661261969
172007	2/28/2019 11:06	B	370458554.2	9023518.26	-74.205	-43.637	3012	-0.136092144	0.024307249	0.630225121
172007	2/28/2019 11:44	A	44138444.35	98307725.65	-74.188	-43.646	2307	-0.031355383	0.008575735	0.625966191

172007	2/28/2019 12:00	B	50072959.66	63632952.84	-74.189	-43.647	929	-0.018842389	0.005685348	0.622454755
172007	2/28/2019 12:46	B	5841362	505012.5	-74.2	-43.646	2752	-0.039447477	0.009437736	0.614714611
172007	2/28/2019 13:58	0	10788728326	407698414.4	-74.291	-43.569	4341	-0.392699008	-0.008453525	0.686681768
172007	2/28/2019 14:37	B	237719067.7	167384884.8	-74.313	-43.595	2347	-0.467167116	0.011390948	0.699473263
172007	3/2/2019 1:21	B	18383328.36	2399539216	-73.999	-43.584	125050	-0.812936	-0.170440655	1.033611459
172007	3/2/2019 1:29	B	1036219.926	124374764.1	-74	-43.583	475	-0.821583971	-0.173176263	1.035787668
172007	3/2/2019 2:23	1	1458199.983	60298.01741	-73.955	-43.695	3256	-0.842500719	-0.01658196	1.077197634
172007	3/2/2019 2:28	B	4112712	935712	-73.955	-43.693	290	-0.846233103	-0.027856501	1.077616938
172007	3/2/2019 2:56	A	68303220.19	83904.80767	-73.959	-43.699	1650	-0.806247016	-0.008601725	1.064455911
172007	3/2/2019 3:44	2	628511.7646	22213.23543	-73.998	-43.701	2917	-0.699350759	-0.004115176	1.024953067
172007	3/2/2019 4:02	B	6603365.808	142295.1916	-74	-43.692	1076	-0.751426594	-0.03135144	1.031347358
172007	3/2/2019 4:40	B	17853280.84	2151353.156	-74.014	-43.703	2244	-0.654241105	0.006224034	1.011230467
172007	3/2/2019 5:15	B	14342776.25	2126800.746	-74.019	-43.699	2142	-0.616502512	0.003195292	0.999281982
172007	3/2/2019 6:36	B	4011794.459	598586.5406	-74.016	-43.697	4830	-0.657198481	-0.007438577	1.008501952
172007	3/2/2019 8:18	2	235583.6781	14790.82188	-74.066	-43.689	6116	-0.434739465	-0.014090425	0.943406461
172007	3/2/2019 9:33	B	25126894.42	153124970.1	-74.096	-43.681	4515	-0.3634226	-0.004673838	0.910919194

172007	3/2/2019 9:59	1	1117071.114	439866.8861	-74.066	-43.688	1570	-0.430553061	-0.01557824	0.941582987
172007	3/2/2019 10:29	B	84910364.39	21977744.61	-74.066	-43.689	1799	-0.421206024	-0.014180084	0.939508232
172007	3/2/2019 11:11	0	83598299360	3158693075	-74.131	-43.66	2526	-0.42643241	-0.026944455	0.939165305
172007	3/4/2019 0:19	B	5196983.658	127504932.8	-74.26	-43.505	133650	0.174542597	-0.129344772	0.158199118
172007	3/4/2019 0:43	A	38704.29286	57420.20714	-74.281	-43.485	1471	0.074845906	-0.116302915	0.150183965
172007	3/4/2019 1:57	B	1379156.668	382241.8319	-74.303	-43.47	4432	-0.078525254	-0.102889905	0.138231302
172007	3/4/2019 2:19	1	3523714.468	265322.032	-74.247	-43.493	1319	0.236482696	-0.160558139	0.141207705
172007	3/4/2019 2:23	B	2539938.364	302886.6362	-74.246	-43.493	214	0.238815584	-0.177444646	0.140038903
172007	3/4/2019 2:54	B	82270172.82	9799205.18	-74.248	-43.5	1896	0.335738669	-0.173042708	0.163798505
172007	3/4/2019 3:44	2	66011.80879	105308.6912	-74.223	-43.505	2993	0.488337283	-0.200461096	0.166855566
172007	3/4/2019 3:58	B	2816160.039	36132.46131	-74.217	-43.493	831	0.402707084	-0.22760526	0.111079718
172007	3/4/2019 4:38	A	3518626.926	8251473.074	-74.209	-43.465	2421	0.029576808	-0.284231951	0.020262015
172007	3/4/2019 7:51	B	79681549.57	9776703.432	-74.219	-43.453	11534	-0.150184444	-0.248300034	0.016412255
172007	3/4/2019 9:31	2	296043.6233	56408.87665	-74.273	-43.5	6032	0.206847999	-0.105468436	0.19387881
172007	3/4/2019 10:20	A	25054.64118	9905.858819	-74.301	-43.491	2948	0.118236875	-0.086138547	0.191598347
172007	3/4/2019 11:52	A	63770.49612	813086.0039	-74.254	-43.379	5504	-0.670905974	-0.218209373	-0.025665104

172007	3/4/2019 12:16	B	7711674.875	3554399.625	-74.232	-43.396	1431	-0.6917475	-0.254994487	-0.050039188
172007	3/4/2019 12:40	B	126097822.1	36304860.37	-74.239	-43.41	1431	-0.572059519	-0.252388683	-0.038004504
172007	3/4/2019 13:31	A	196806693.8	12252028.66	-74.227	-43.485	3077	0.225169316	-0.222532825	0.095566177
172007	3/4/2019 13:41	B	42297240.64	2331324.363	-74.23	-43.496	605	0.350604719	-0.20598109	0.138655902
172007	3/4/2019 14:18	B	107392834.2	5742608.339	-74.228	-43.506	2189	0.452013875	-0.194597839	0.17454529
172007	3/6/2019 1:31	B	1147065.753	232445944.7	-74.144	-43.537	126817	-0.631704053	-0.289764662	0.587589369
172007	3/6/2019 1:40	B	215928.4251	25344906.07	-74.145	-43.543	538	-0.661058329	-0.284585958	0.591066176
172007	3/6/2019 2:14	A	385098.5131	71118.48691	-74.131	-43.568	2058	-0.869927581	-0.260828039	0.598031235
172007	3/6/2019 3:20	B	35956626.66	8555033.838	-74.098	-43.603	3940	-0.827180099	-0.192421388	0.571177243
172007	3/6/2019 3:53	B	2227605.311	516077.1893	-74.094	-43.619	1948	-0.752446231	-0.153948769	0.54937796
172007	3/6/2019 7:29	B	5366731.607	968046.8934	-74.015	-43.673	12994	-0.43510991	-0.069654689	0.415392055
172007	3/6/2019 9:13	B	54139149.78	44150366.72	-74.021	-43.659	6243	-0.579942694	-0.109213547	0.462445502
172007	3/6/2019 11:18	B	239651.7303	1869993.27	-74.133	-43.587	7480	-0.928603289	-0.193053048	0.599848049
172007	3/6/2019 11:29	B	831353.0878	403719.4122	-74.135	-43.586	645	-0.92652402	-0.198529873	0.600613872
172007	3/6/2019 13:04	B	3457115.895	257048.105	-74.087	-43.561	5716	-0.581917129	-0.347092461	0.542301773
172007	3/6/2019 13:04	B	3126762.665	204506.3349	-74.086	-43.561	23	-0.575922034	-0.347092461	0.540686632

172007	3/6/2019 13:36	B	1635695.701	165858.799	-74.09	-43.564	1933	-0.627798782	-0.337014873	0.550986105
172007	3/6/2019 14:40	B	22232142.69	629585.813	-74.116	-43.54	3815	-0.448358682	-0.340627479	0.561621611
172007	3/6/2019 15:18	A	2454885.5	7224780.5	-74.184	-43.551	2305	-0.907282826	-0.205378492	0.619100135
172007	3/8/2019 2:57	1	183197.6901	97660.80995	-73.79	-43.644	128325	-0.182352772	-0.136383242	0.663969605
172007	3/8/2019 3:12	2	10420260.66	207247.3377	-73.79	-43.649	856	-0.131828841	-0.118991099	0.660592769
172007	3/8/2019 4:15	B	4360905.173	1124122.827	-73.827	-43.655	3787	0.375945033	-0.125900004	0.596987335
172007	3/8/2019 7:06	B	33980182.52	1726634.483	-73.896	-43.634	10262	-0.266041151	-0.183507949	0.419414589
172007	3/8/2019 8:45	A	1984713.541	993786.9591	-74.078	-43.495	5950	0.845314016	-0.604423356	0.570666919
172007	3/8/2019 10:25	B	14909886.53	5111826.47	-74.192	-43.455	5986	2.202886961	-0.339930247	0.212027056
172007	3/8/2019 12:03	B	55842.73792	524309.7621	-74.231	-43.532	5883	0.632382504	-0.158170066	0.90575394
172007	3/8/2019 12:21	B	2010756.863	351029.6371	-74.235	-43.532	1112	0.642674406	-0.147440423	0.90553712
172007	3/8/2019 12:45	B	2190324.5	188498	-74.262	-43.53	1417	0.557983815	-0.090317227	0.900177846
172007	3/8/2019 12:57	B	5143808.695	197764.305	-74.262	-43.529	729	0.562126049	-0.097605697	0.901198944
172007	3/8/2019 13:59	2	63339.25	63339.25	-74.207	-43.533	3733	0.816355614	-0.196552209	0.889659742
172007	3/8/2019 14:42	A	19699088.76	1153196.243	-74.235	-43.495	2545	3.407987234	-0.20598109	0.746154742
172007	3/10/2019 0:52	B	14307756475	5165617630	-74.306	-43.498	123029	0.2144354	-0.124080014	0.657364885

172007	3/10/2019 1:33	B	39582913253	8607101052	-74.347	-43.483	2442	0.224661772	-0.130028545	0.657968355
172007	3/10/2019 1:54	1	4395504.277	878620.723	-74.349	-43.484	1280	0.218159598	-0.132574853	0.661389839
172007	3/10/2019 2:27	3	18669.87221	8992.627787	-74.361	-43.464	1980	0.45973243	-0.166981076	0.65967008
172007	3/10/2019 4:12	B	7810372.431	1526824.069	-74.341	-43.418	6308	0.210995542	-0.153697983	0.482024097
172007	3/10/2019 8:22	B	1334476.994	52372263.01	-74.353	-43.372	14960	-0.015409296	-0.210905664	0.422419661
172007	3/10/2019 10:02	B	18831512.5	18831512.5	-74.336	-43.415	6031	0.105804217	-0.149730453	0.454593657
172007	3/10/2019 10:55	B	27881640.81	219417.1938	-74.344	-43.426	3170	0.289215257	-0.153817085	0.503219959
172007	3/10/2019 11:13	B	6749115.519	66454.98123	-74.349	-43.429	1063	0.360736062	-0.164193283	0.524068298
172007	3/10/2019 12:15	B	4731611.313	283688.6869	-74.355	-43.427	3770	0.386042181	-0.169913973	0.538049304
172007	3/10/2019 13:22	A	2600818721	344557711.5	-74.365	-43.494	3994	0.33606522	-0.190444871	0.72438178
172007	3/10/2019 14:01	B	67586812.39	7710086.107	-74.371	-43.503	2313	0.033692814	-0.178055778	0.785711668
172007	3/10/2019 15:00	B	13043033.48	12200735.52	-74.422	-43.519	3582	-0.184030538	-0.237001922	0.88773021
172007	3/10/2019 15:33	B	3084879.563	2249754.437	-74.44	-43.523	1942	-0.31353625	-0.254120053	0.918276276
172007	3/12/2019 0:41	B	19672182124	6044451328	-74.073	-43.585	119315	1.268487749	-0.408767967	0.58076669
172007	3/12/2019 2:04	0	873049571.4	17264235.13	-74.057	-43.554	4951	1.479450329	-0.419027899	0.602422222
172007	3/12/2019 2:52	B	38235939.83	6236085.169	-74.062	-43.555	2866	1.567289787	-0.439339959	0.630030716

172007	3/12/2019 3:35	A	1627015.561	502186.939	-73.982	-43.55	2622	2.190156468	-0.523358585	0.681081927
172007	3/12/2019 3:44	B	1289561.774	220170.7258	-73.981	-43.548	545	2.207609272	-0.528800228	0.676971051
172007	3/12/2019 4:32	B	1547445.993	438466.5074	-73.959	-43.55	2854	2.094197962	-0.53440291	0.705503401
172007	3/12/2019 7:59	A	132375.8104	162270.6896	-73.877	-43.522	12451	1.11938792	-0.679770684	0.576912347
172007	3/12/2019 9:36	B	1635179.059	499544.941	-73.836	-43.518	5814	0.829977823	-0.669104041	0.518058859
172007	3/12/2019 10:23	B	9050548.474	470316.526	-73.855	-43.519	2830	0.947062885	-0.685250485	0.554208737
172007	3/12/2019 11:29	A	11862451843	535958128.8	-73.97	-43.534	3932	1.89577979	-0.636410497	0.600889866
172007	3/12/2019 11:51	B	162503828	7726622.021	-73.972	-43.535	1339	2.258166194	-0.587935928	0.592970216
172007	3/12/2019 12:02	B	73916986.69	2935005.809	-73.972	-43.534	653	2.38701364	-0.592552968	0.579169963
172007	3/12/2019 12:42	B	51845352.58	4816689.422	-73.984	-43.536	2385	2.449720013	-0.577470203	0.578428422
172007	3/14/2019 1:28	A	2338682.764	12729.73603	-74.242	-43.52	132385	0.391245037	-0.152966993	0.819111716
172007	3/14/2019 1:43	1	15002024.32	1992536.678	-74.212	-43.519	882	0.55997314	-0.185958619	0.821379393
172007	3/14/2019 2:13	B	4139137.722	996704.2777	-74.221	-43.506	1804	1.539455907	-0.210044414	0.779748511
172007	3/14/2019 2:47	B	1299773.738	298754.7624	-74.217	-43.505	2030	1.710253224	-0.212673474	0.777781418
172007	3/14/2019 3:28	B	152996.688	16964035.81	-74.183	-43.507	2438	1.369497389	-0.280210295	0.863962189
172007	3/14/2019 3:52	B	393678.7359	1615183.764	-74.183	-43.507	1473	1.369497389	-0.280210295	0.863962189

172007	3/14/2019 7:36	B	8626582.645	7028103.855	-74.229	-43.45	13434	0.984181021	-0.255574781	0.387720338
172007	3/14/2019 9:15	B	2023977689	22361461.12	-74.224	-43.463	5948	1.697914618	-0.242852402	0.439853638
172007	3/14/2019 12:08	B	252570.4492	4924562.051	-74.234	-43.484	10383	2.613268761	-0.216828959	0.581394958
172007	3/14/2019 12:32	B	405558.4691	328051.5309	-74.233	-43.486	1413	2.547642275	-0.218280428	0.607265471
172007	3/14/2019 13:11	B	1016738	462722	-74.238	-43.49	2336	2.590593717	-0.19486182	0.647715744
172007	3/14/2019 13:33	B	6325921.488	317094.5116	-74.242	-43.503	1330	1.963210449	-0.164687621	0.719628827
172007	3/14/2019 14:12	A	29720705.54	10112.96116	-74.301	-43.501	2321	1.720306553	-0.084631505	0.70766885
172007	3/16/2019 1:19	0	35923880.74	3831503.763	-74.134	-43.501	126418	-0.269583145	-0.393829078	0.681008667
172007	3/16/2019 2:08	A	211684.202	110083.798	-74.168	-43.496	2983	0.24869037	-0.338041538	0.641197528
172007	3/16/2019 3:01	B	925023.8284	243332.6716	-74.187	-43.497	3164	0.744847875	-0.289156616	0.588907908
172007	3/16/2019 3:12	1	1671675.612	1788826.888	-74.153	-43.502	663	-0.05589976	-0.35704133	0.672223248
172007	3/16/2019 3:46	B	32471578.12	2198829.884	-74.163	-43.511	2062	0.167259571	-0.31441588	0.68469593
172007	3/16/2019 8:52	B	190806763	4978615.022	-74.257	-43.499	18350	1.333734167	-0.200536509	0.518909654
172007	3/16/2019 10:21	A	44641.4166	10411.0834	-74.223	-43.501	5324	1.316491585	-0.207220608	0.527377645
172007	3/16/2019 11:05	B	1070581653	113025217.9	-74.318	-43.496	2632	1.493668315	-0.119943943	0.540396713
172007	3/16/2019 11:57	B	5443368.484	518636.0164	-74.328	-43.491	3142	1.763572911	-0.08790252	0.603755129

172007	3/16/2019 12:46	B	1377737.252	110679862.7	-74.173	-43.489	2908	0.460225292	-0.316980918	0.604316574
172007	3/16/2019 12:59	B	3429386.097	25055810.9	-74.174	-43.492	780	0.460225292	-0.329093199	0.604316574
172007	3/18/2019 2:41	B	51303562.41	4635175274	-74.125	-43.483	135731	2.372659547	-0.532349422	0.180135376
172007	3/18/2019 3:05	B	283869447.2	15142092.78	-74.114	-43.469	1474	2.372659547	-0.532349422	0.180135376
172007	3/18/2019 8:30	B	73818583.09	11017025.91	-74.154	-43.506	19491	1.977709455	-0.314046062	0.352646548
172007	3/18/2019 10:04	B	331794127.7	12772154.3	-74.122	-43.475	5639	2.288946871	-0.245870601	0.119691331
172007	3/18/2019 11:43	A	581244.5943	72895.90571	-74.349	-43.465	5932	2.839872676	-0.14568178	0.29295515
172007	3/18/2019 12:50	A	93782.49425	1124729.506	-74.401	-43.49	4040	2.042161986	-0.243332323	0.658914898
172007	3/18/2019 14:31	B	166472053.4	109166395.1	-74.35	-43.45	6047	2.365650059	-0.196206547	0.359667894
172007	3/20/2019 0:41	A	5517368.403	731994.0975	-74.31	-43.455	122966	2.47078456	-0.111400011	-0.271887452
172007	3/20/2019 2:15	A	2139758.692	1131909.808	-74.316	-43.491	5679	4.093275432	-0.086397813	0.113585619
172007	3/20/2019 2:24	B	641638842.9	76074599.62	-74.298	-43.493	500	3.892821916	-0.091093378	0.052385017
172007	3/20/2019 3:24	B	8655020.241	8071624.759	-74.245	-43.476	3611	1.530170162	-0.185170292	-0.274333676
172007	3/20/2019 4:03	B	1087322.66	586969.34	-74.239	-43.474	2360	0.928035965	-0.209857399	-0.318966937
172007	3/20/2019 8:05	B	12720437.86	32559187.14	-74.275	-43.498	14538	3.127788112	-0.111600535	-0.073088858
172007	3/20/2019 9:47	A	122655.2138	31044.78624	-74.348	-43.469	6067	4.395441034	-0.143603577	0.136568426

172007	3/20/2019 10:20	B	10052239.13	3926683.367	-74.34	-43.477	1990	4.282189652	-0.128012172	0.12732224
172007	3/20/2019 10:39	B	167039283.3	51324976.66	-74.359	-43.44	1177	4.221350689	-0.142527294	0.038259672
172007	3/22/2019 1:28	A	10466282.99	16203.5145	-74.356	-43.493	139704	0.43662616	-0.129165774	0.148401512
172007	3/22/2019 1:44	B	1972790.728	59390.27231	-74.354	-43.493	951	0.440529509	-0.129640451	0.143633647
172007	3/22/2019 2:42	2	14595546.94	551799.0628	-74.265	-43.522	3498	0.329067154	-0.082487589	0.047751745
172007	3/22/2019 4:27	B	2182318.544	430459.9564	-74.299	-43.559	6277	0.068300016	-0.017552022	0.198830201
172007	3/22/2019 7:41	B	7668528.116	677766.384	-74.37	-43.593	11650	-0.553571787	-0.004975095	0.35632297
172007	3/22/2019 9:25	A	6253288.03	196596.9704	-74.143	-43.498	6249	-0.754458584	-0.396920509	-0.039420076
172007	3/22/2019 11:16	B	7168951.475	1482653.525	-74.109	-43.494	6667	-0.829968094	-0.524907291	-0.046095217
172007	3/22/2019 13:10	B	11411469.36	1704423.138	-74.226	-43.551	6838	-0.233025046	-0.137825046	0.089018254
172007	3/22/2019 14:13	B	455097579.1	6877028.896	-74.413	-43.47	3814	-0.556181265	-0.135710562	-0.087681178
172007	3/24/2019 2:42	B	5897502898	374386435.2	-74.39	-43.495	131317	0.02383999	-0.091357773	0.221517193
172007	3/24/2019 3:43	B	2078575.506	137505674.5	-74.308	-43.498	3682	0.233541278	-0.092791887	0.213950341
172007	3/24/2019 4:25	B	1612981.413	1161780.587	-74.336	-43.479	2495	0.223144355	-0.119848283	0.232639898
172007	3/24/2019 9:00	B	17607981.07	707069.4309	-74.487	-43.391	16496	3.002409921	-0.515430982	-0.48068732
172007	3/24/2019 10:18	B	485533755.4	2694971.084	-74.243	-43.445	4653	3.112932757	-0.150735716	0.016235887

172007	3/24/2019 12:59	B	53672563.9	614981.1048	-74.14	-43.46	9708	1.157099824	-0.401184249	-0.656801757
172007	3/26/2019 8:41	1	11057280.42	825492.0821	-74.321	-43.62	157281	-0.630468906	0.066693595	0.386633614
172007	3/26/2019 9:31	A	5905509.353	9416212.647	-74.342	-43.596	3038	-0.69817987	0.022060949	0.346636372
172007	3/26/2019 10:16	B	13368028.37	6066738.13	-74.34	-43.587	2701	-0.661939083	0.00755069	0.334641171
172007	3/26/2019 10:56	B	1534436163	69343690.14	-74.345	-43.541	2386	-0.718289788	-0.054939353	0.288880021
172007	3/26/2019 12:32	B	572514577.4	175921711.1	-74.409	-43.517	5744	-0.450081761	-0.165898584	0.228174095
172007	3/26/2019 12:45	B	906319482.6	164751491.9	-74.393	-43.524	811	-0.450081761	-0.169106685	0.228174095
172007	3/26/2019 13:23	B	183102429.1	26074773.43	-74.406	-43.509	2254	-0.256665014	-0.193663106	0.197746941
172007	3/26/2019 14:10	B	4432721.782	1870075.218	-74.4	-43.516	2835	-0.240498913	-0.195397474	0.198946018
172007	3/26/2019 15:07	B	373477821.5	20600590.96	-74.426	-43.594	3437	-0.532689504	-0.079684442	0.307212421
172007	3/28/2019 2:22	B	2480201784	3522545537	-74.356	-43.753	126866	-1.069003923	0.05297721	0.536299193
172007	3/28/2019 2:59	B	1793729662	45013164.75	-74.42	-43.846	2251	-1.060643189	0.054862201	0.535630348
172007	3/28/2019 4:04	B	15423351.49	1215757.508	-74.461	-43.893	3862	-0.909541657	0.078973631	0.528672419
172007	3/28/2019 9:55	B	50080032	741762	-74.672	-44.081	21082	-0.89213012	-0.058345517	0.219149077
172007	3/28/2019 10:18	B	34459069.07	1805749.433	-74.703	-44.113	1389	-0.819909627	-0.119088962	0.217911167
172007	3/28/2019 11:33	A	30096350586	1137152314	-74.592	-44.064	4462	-0.815055026	0.045352186	0.204633345

172007	3/28/2019 12:01	B	130920411.4	3412005.099	-74.586	-44.062	1687	-0.693543676	0.100179474	0.212276998
172007	3/28/2019 12:12	A	29356582.72	1828166.281	-74.541	-44.063	645	-0.674458007	0.120449645	0.21008239
172007	3/28/2019 13:17	B	5027201.401	217801.0991	-74.574	-44.072	3911	-0.773441297	0.094154707	0.199051747
172007	3/28/2019 13:49	B	49173784.37	4612526.129	-74.591	-44.063	1930	-0.738887767	0.064865305	0.215896471
172007	3/28/2019 14:22	1	11143920.5	3362	-74.621	-44.061	1992	-0.797065779	0.040775488	0.220329368
172007	3/30/2019 2:16	A	405891649.6	33378558.45	-74.482	-43.99	129249	-1.178133153	0.15933507	0.433991161
172007	3/30/2019 3:21	B	63033062.64	6182774.358	-74.517	-43.982	3903	-1.179451738	0.149123928	0.432102998
172007	3/30/2019 7:53	B	429452723.5	654880101	-74.528	-44.042	16305	-1.147343779	0.201434052	0.429490491
172007	3/30/2019 9:31	B	321083626.4	424904009.6	-74.494	-44.079	5881	-1.135013225	0.197478802	0.429751175
172007	3/30/2019 10:29	B	3943423.957	33176938.54	-74.437	-44.085	3478	-1.137587174	0.190261434	0.43428101
172007	4/1/2019 0:39	0	49963644.21	2719408.792	-74.581	-44.036	137415	-1.10505866	0.128041156	0.461948252
172007	4/1/2019 2:39	0	37744749111	1426140347	-74.447	-44.003	7161	-1.122930983	0.183760918	0.453235505
172007	4/1/2019 3:17	B	90685463.54	3739442.963	-74.444	-44.008	2308	-1.119184929	0.204821997	0.448576221
172007	4/1/2019 4:18	B	1165871.188	265826.8124	-74.508	-44.012	3680	-1.128653897	0.177052548	0.454864333
172007	4/1/2019 9:07	A	99392492.41	3756040.087	-74.551	-43.982	17329	-1.188029998	0.143185933	0.46346529
172007	4/1/2019 10:20	2	78722.5624	90767.4376	-74.462	-44.018	4361	-1.075516021	0.230931604	0.437994661

172007	4/2/2019 0:03	B	96776925.54	6852268.965	-74.121	-44.095	49388	-0.896364764	0.275977204	-0.292160841
172007	4/2/2019 1:23	B	217674112.5	508032	-74.129	-43.97	4824	-0.76161951	0.383744083	-0.193849554
172007	4/2/2019 2:57	A	29366984.14	2877496.363	-74.245	-43.828	5590	-1.075987362	0.293605391	-0.129405374
172007	4/2/2019 10:36	B	1100012037	67538887.87	-74.193	-43.719	27541	-0.911679886	0.179556707	-0.216450634
172007	4/2/2019 11:34	B	2029435097	20769931.79	-74.312	-43.845	3501	-0.806276503	0.18016825	-0.145934357
172007	4/2/2019 12:13	B	7618150936	191367314.1	-74.315	-43.866	2337	-0.823828971	0.175139694	-0.115350417
172007	4/2/2019 12:49	B	299004750.6	7556411.881	-74.404	-43.905	2170	-0.89685235	0.178292001	-0.084382703
172007	4/2/2019 13:41	1	14887647.16	19834.84118	-74.405	-43.906	3127	-0.876385269	0.165441492	-0.084551242
172007	4/2/2019 14:24	B	51483080.66	257543.3414	-74.444	-43.881	2539	-0.778803703	0.114388579	-0.118176652
172007	4/3/2019 1:27	A	7187517.789	336652.2114	-74.255	-43.891	39824	-0.827606553	0.307721518	0.003859395
172007	4/3/2019 2:35	B	184904740.5	97607117.55	-74.284	-43.897	4066	-0.868181928	0.298035012	0.005025775
172007	4/3/2019 2:50	B	12209977.27	6078184.732	-74.274	-43.89	897	-0.864585987	0.296190459	0.004735529
172007	4/3/2019 8:47	2	377239.9332	29981.0668	-74.324	-43.915	21393	-0.939237619	0.269569965	-0.000337896
172007	4/4/2019 2:13	B	6058362.21	2199302783	-74.303	-43.903	62771	-0.632417893	0.273433885	0.02115073
172007	4/4/2019 2:49	B	1398631.554	3905577.446	-74.313	-43.895	2166	-0.532681239	0.265370937	0.009723319
172007	4/4/2019 8:36	B	7014213.206	1676771.794	-74.377	-43.825	20850	-0.056216778	0.131981001	-0.205779615

172007	4/4/2019 10:12	B	312107816.9	1202162217	-74.306	-43.875	5746	-0.116289455	0.238623602	-0.041832943
172007	4/4/2019 11:11	B	78053357.89	2431331.114	-74.293	-43.898	3556	-0.738282294	0.278177709	0.030156442
172007	4/4/2019 12:02	B	2365668.593	222431.907	-74.277	-43.901	3011	-0.870841338	0.302112443	0.042429685
172007	4/4/2019 13:36	A	4005254734	19638586.36	-74.211	-43.863	5659	-0.241204865	0.311343058	-0.001113794
172007	4/4/2019 14:08	B	218810703	3142587.522	-74.199	-43.861	1909	-0.07234788	0.319436563	-0.02250741
172007	4/5/2019 0:38	B	233880703.1	1431573909	-74.227	-43.83	37835	-0.038071973	0.26943207	-0.244987467
172007	4/5/2019 1:54	B	1549030890	18000982.58	-74.224	-43.777	4539	0.06811512	0.242104085	-0.282517944
172007	4/5/2019 2:30	B	2930372412	2214364680	-74.28	-43.767	2177	0.060606568	0.235794187	-0.299153082
172007	4/5/2019 3:38	A	64297.65936	3518.340644	-74.239	-43.754	4043	-0.006657555	0.215887092	-0.342151806
172007	4/5/2019 8:21	B	67900.05397	4939564.446	-74.227	-43.809	17020	-0.188181629	0.286827375	-0.213561749
172007	4/5/2019 10:00	B	1328827.79	1538049.21	-74.249	-43.809	5899	-0.408255898	0.271621953	-0.188245392
172007	4/5/2019 10:20	B	6088970.377	57565752.12	-74.285	-43.825	1211	-0.496875175	0.251913181	-0.156096824
172007	4/5/2019 11:59	A	1260760.206	393785.7939	-74.354	-43.938	5962	-1.136239117	0.257444249	-0.054338954
172007	4/5/2019 12:23	B	2474706.012	484734.4879	-74.317	-43.931	1447	-1.132184988	0.288773146	-0.051484721
172007	4/5/2019 12:38	B	7932144.5	193442	-74.331	-43.939	862	-1.104542425	0.283430637	-0.053556515
172007	4/5/2019 13:22	B	5932533.877	389379.123	-74.34	-43.949	2664	-1.052160511	0.279933633	-0.057982438

172007	4/6/2019 0:13	B	101554529.6	1295744.944	-74.352	-43.874	39051	-1.145692772	0.21539415	-0.24465014
172007	4/6/2019 2:15	B	1712574997	203054107.6	-74.274	-43.87	7352	-1.121744546	0.261481145	-0.254833359
172007	4/6/2019 2:39	B	161516055.9	26905456.62	-74.302	-43.884	1429	-1.116440949	0.266633966	-0.256711262
172007	4/6/2019 3:18	B	4374936115	1514701.545	-74.286	-43.887	2320	-1.101267743	0.283171158	-0.261783993
172007	4/6/2019 4:53	B	204009833.5	3637681.04	-74.189	-43.87	5707	-1.036704127	0.303328084	-0.281557263
172007	4/6/2019 8:13	B	23515963.05	944216.9517	-74.318	-43.936	11995	-1.139718713	0.286729647	-0.244688026
172007	4/6/2019 11:35	A	1213367.716	248952.7841	-74.517	-43.94	12116	-1.134734686	0.083708088	-0.23464462
172007	4/6/2019 11:49	A	1095031260	41386045.89	-74.521	-43.93	824	-1.120183187	0.076711127	-0.236539603
172007	4/6/2019 12:03	B	55039021.06	2262775.442	-74.519	-43.928	867	-1.108322866	0.065818441	-0.238649778
172007	4/7/2019 1:26	A	4467595.022	7557.97795	-74.488	-43.992	48209	-1.238683024	0.183496531	0.394478389
172007	4/7/2019 2:08	A	70016181.51	2649028.994	-74.501	-43.97	2516	-1.235216952	0.142558376	0.393807711
172007	4/7/2019 2:17	A	132125.8624	2202.637582	-74.504	-43.972	530	-1.2365893	0.137634212	0.393415937
172007	4/7/2019 2:53	2	8035933.97	304476.0299	-74.443	-43.975	2168	-1.207568308	0.204045288	0.404192263
172007	4/7/2019 3:27	B	1448781.761	199759.2386	-74.425	-43.974	1997	-1.196259989	0.222891744	0.409417847
172007	4/7/2019 3:56	B	1643760.721	266947.7794	-74.411	-43.975	1746	-1.190605292	0.235816162	0.412003699
172007	4/7/2019 4:34	0	19176028.01	26930644.49	-74.419	-43.995	2283	-1.200249705	0.243212265	0.407726154

172007	4/8/2019 1:02	A	1962690.373	545121.6268	-74.337	-43.978	73716	-0.767472233	0.306956096	-0.729103625
172007	4/8/2019 1:53	A	4520081.374	899043.6262	-74.321	-43.993	3008	-0.790717859	0.334044504	-0.745291143
172007	4/8/2019 1:53	B	3279406.423	307448.0766	-74.322	-43.993	31	-0.790717859	0.334044504	-0.745291143
172007	4/8/2019 2:30	B	78911470.24	219110.2591	-74.304	-43.99	2239	-0.838675362	0.349206836	-0.772649447
172007	4/8/2019 3:35	2	161850.25	161850.25	-74.241	-43.977	3885	-0.931227197	0.378120976	-0.82737643
172007	4/8/2019 4:14	A	1194958.625	3324775.875	-74.264	-44.008	2304	-0.884010122	0.358228512	-0.801640447
172007	4/8/2019 7:49	B	84659630.13	28338299.87	-74.194	-44.043	12930	-0.906406361	0.343260306	-0.822022784
172007	4/8/2019 9:29	A	2403179.66	128434.8404	-74.29	-43.969	5985	-0.859710264	0.341708565	-0.78263413
172007	4/9/2019 0:38	A	1317583.766	82301.23395	-74.233	-43.885	54534	-0.924720203	0.333979583	-0.858112036
172007	4/9/2019 1:34	B	59857015.42	13036721.08	-74.248	-43.831	3399	-0.91870714	0.306205384	-0.864811224
172007	4/9/2019 3:13	3	186509.903	9974.097045	-74.022	-43.711	5899	-1.046542691	0.033552937	-0.95928282
172007	4/9/2019 3:50	0	53311440.78	169903.7213	-73.954	-43.668	2256	-1.051853232	-0.090560136	-0.973779431
172007	4/9/2019 9:19	1	5570028.54	630743.9604	-73.931	-43.563	19704	-0.998753508	-0.471719368	-0.986890348
172007	4/9/2019 11:55	B	33492383.5	7372062.999	-73.837	-43.501	9398	-1.011646961	-0.768771452	-1.035090111
172007	4/9/2019 12:54	B	285704663.3	5750509.237	-73.942	-43.568	3547	-1.028799818	-0.458205385	-1.003787368
172007	4/9/2019 13:08	B	107121361	2760643.52	-73.939	-43.569	827	-1.024469341	-0.440393758	-0.99996253

172007	4/9/2019 13:32	B	218865042	63012.5	-73.932	-43.572	1411	-1.024763634	-0.413403642	-0.999572568
172007	4/9/2019 14:09	B	284535448.2	10483852.34	-73.903	-43.568	2238	-1.027019051	-0.426286335	-1.00228413
172007	4/10/2019 0:11	B	32403749.71	1359954.788	-73.809	-43.539	36109	-1.168629572	-0.552240765	-0.939390899
172007	4/10/2019 1:32	B	23456212.09	5661896.412	-73.919	-43.573	4863	-1.244213766	-0.420074716	-0.932154898
172007	4/10/2019 1:51	B	21622377.84	3247804.663	-73.913	-43.568	1174	-1.243700287	-0.440311023	-0.932166947
172007	4/10/2019 2:54	A	193534.1471	157524.3529	-73.936	-43.599	3749	-1.252628816	-0.293154368	-0.926669237
172007	4/10/2019 3:10	B	20081134.68	3601155.317	-73.922	-43.567	956	-1.241165939	-0.419856719	-0.93057129
172007	4/10/2019 3:32	B	25466530.87	60366019.63	-73.924	-43.573	1327	-1.243652678	-0.412606814	-0.930436557
172007	4/10/2019 9:04	A	1827214263	97392931.3	-73.924	-43.648	19907	-1.273020646	-0.162950831	-0.923394735
172007	4/10/2019 10:45	B	47253383.06	2393452.938	-73.915	-43.648	6073	-1.276374387	-0.146039542	-0.92264902
172007	4/10/2019 11:28	A	7526738014	284413496.2	-73.968	-43.602	2569	-1.247060289	-0.219905591	-0.921704065
172007	4/10/2019 12:39	B	90071663.21	4487737.786	-74.027	-43.59	4265	-1.177238464	-0.307716068	-0.909317302
172007	4/10/2019 13:48	B	85401250.26	1274694.737	-74.063	-43.59	4127	-1.137951901	-0.271205393	-0.896130637
172007	4/10/2019 14:14	2	1139702.424	230257.5756	-74.058	-43.625	1581	-1.151552183	-0.171349493	-0.896303354
172007	4/10/2019 14:55	B	104854.6584	1552905.342	-74.064	-43.624	2454	-1.143493585	-0.16915108	-0.893712707
172007	4/11/2019 1:20	A	1148998.625	384495.875	-74.089	-43.551	37513	-0.034680731	-0.381209047	-1.483794493

172007	4/11/2019 2:29	1	61141615.63	1540912.865	-74.153	-43.538	4132	0.289558733	-0.315815025	-1.332443059
172007	4/11/2019 2:58	B	16709112.89	3836667.112	-74.152	-43.539	1721	0.305460224	-0.309102084	-1.31528364
172007	4/11/2019 3:11	2	67394.63168	29409.86832	-74.125	-43.534	788	0.223810136	-0.357364336	-1.377967562
172007	4/11/2019 8:55	A	324137.9217	65510.57825	-74.074	-43.521	20660	-0.457828852	-0.515568591	-1.5160622
172007	4/12/2019 1:03	A	12985.48348	49191.01652	-74.089	-43.635	58080	1.199862976	-0.120083983	-1.348599259
172007	4/12/2019 2:12	2	79553.48467	21664.51533	-74.091	-43.624	4116	1.097449835	-0.147072624	-1.37116385
172007	4/12/2019 2:48	B	27569597.84	13526122.16	-74.106	-43.628	2151	1.211770446	-0.138714009	-1.323934203
172007	4/12/2019 2:51	A	3134592.469	83032.53091	-74.103	-43.622	221	1.182438221	-0.146200263	-1.331926107
172007	4/12/2019 3:50	B	15076578.38	2096782.62	-74.132	-43.64	3536	1.698175483	-0.075736747	-1.11000015
172007	4/12/2019 4:29	B	8021365.495	1055765.005	-74.138	-43.644	2326	1.697345675	-0.053337601	-1.072800466
172007	4/12/2019 8:43	2	421647.4753	38063.02471	-74.051	-43.623	15270	0.496191944	-0.177173614	-1.535700684
172007	4/13/2019 0:37	A	3337122.14	10362.86008	-74.056	-43.613	57212	-1.014947717	-0.20828112	-1.179536426
172007	4/13/2019 0:53	B	101644816.3	11099246.22	-74.122	-43.622	959	-1.094415853	-0.169070626	-1.176139196
172007	4/13/2019 1:49	B	162990109.7	3336806.771	-74.094	-43.627	3367	-1.108969358	-0.147727736	-1.173876961
172007	4/13/2019 3:01	B	16020137.52	1504451.479	-74.071	-43.629	4333	-1.098990103	-0.147727736	-1.172525483
172007	4/13/2019 3:31	B	48716075.33	245713960.7	-74.169	-43.624	1787	0.46064391	-0.08294261	-1.111084273

172007	4/13/2019 4:13	B	2222008.056	8183403.944	-74.171	-43.631	2507	1.40685013	-0.051615066	-1.00447933
172007	4/13/2019 8:25	B	6169487.796	1776361.204	-74.18	-43.693	15135	0.288354561	0.074283707	-0.849729432
172007	4/13/2019 11:34	A	1.75357E+11	5452107103	-74.248	-43.529	11330	0.800028421	-0.140100711	-1.117435799
172007	4/13/2019 13:15	B	522554803.8	19327230.23	-74.227	-43.5	6057	-0.104052108	-0.221212387	-0.909612021
172007	4/13/2019 13:56	B	227412887.7	18693698.85	-74.224	-43.491	2466	-0.209721589	-0.228084873	-0.908803202
172007	4/13/2019 14:50	B	46724855.03	679082.9694	-74.227	-43.517	3250	0.313353689	-0.195797849	-0.946846597
172007	4/14/2019 2:08	2	161881.4216	46170.57835	-73.733	-43.681	40669	-0.077121439	-0.011691642	-1.263066051
172007	4/14/2019 2:26	B	40777470.87	12537982.13	-73.705	-43.695	1054	-0.036965905	0.01325804	-1.264599478
172007	4/14/2019 3:50	B	74796958.23	152188732.3	-73.774	-43.699	5062	-0.577572821	0.000235442	-1.306719268
172007	4/14/2019 8:19	B	2867951254	56797958.47	-73.837	-43.676	16146	-0.773736644	-0.061610983	-1.028977927
172007	4/14/2019 9:58	B	4845298.825	235335911.2	-74.036	-43.672	5954	-0.944147549	-0.023182973	-0.984974063
172007	4/14/2019 11:34	B	340793374.8	257561767.7	-74.101	-43.718	5745	-0.616601129	0.071405823	-0.985265781
172007	4/14/2019 13:36	B	23504672.57	9541752.427	-74.138	-43.76	7342	-0.199678071	0.157848811	-1.070264965
172007	4/14/2019 13:49	B	5930781.18	1073839.82	-74.114	-43.743	780	-0.194431748	0.132359646	-1.041542937
172007	4/14/2019 14:35	B	83883967.81	11180002.69	-74.098	-43.655	2766	-0.610630242	-0.050733563	-0.945592553
172007	4/14/2019 15:09	B	62470602.24	11124072.26	-74.08	-43.658	2006	-0.58904063	-0.077919379	-0.943046057

172007	4/15/2019 1:28	B	2402005.612	215089727.4	-73.815	-43.549	37120	-0.648325413	-0.515480735	-1.273015804
172007	4/15/2019 1:47	B	603752.2209	19141392.28	-73.815	-43.547	1185	-0.722516241	-0.533291089	-1.2781015
172007	4/15/2019 2:11	B	164152829.4	7416771.13	-73.813	-43.507	1404	-0.674829285	-0.674359197	-1.287857611
172007	4/15/2019 2:51	A	755113742.5	28538038.48	-73.804	-43.489	2413	-0.684341589	-0.7528144	-1.289167025
172007	4/15/2019 3:28	A	33488.9991	18693.5009	-73.763	-43.459	2213	-0.666956297	-0.812904597	-1.2951234
172007	4/15/2019 8:07	B	8647978.235	2216968.265	-73.775	-43.425	16743	-0.640174824	-0.937246542	-1.346118006
172007	4/15/2019 9:47	B	670355080.2	471997406.3	-73.855	-43.553	6008	0.149885317	-0.943911167	-1.249103035
172007	4/15/2019 11:10	B	2166885934	115526063.5	-73.9	-43.506	4997	-0.379367162	-0.760788662	-1.079009131
172007	4/15/2019 12:07	A	165055458	6244662.963	-73.938	-43.532	3394	-0.64560313	-0.635471915	-1.032125574
172007	4/15/2019 13:11	B	707773.2902	59795975.21	-73.977	-43.535	3855	-0.519491593	-0.577470203	-0.968738173
172007	4/15/2019 14:12	B	432436.7952	54998507.2	-73.943	-43.535	3682	-0.582055589	-0.591457973	-1.019255558
172007	4/15/2019 14:49	B	423533.2115	1534951.788	-73.949	-43.536	2206	-0.554872062	-0.58967975	-1.012322482
172007	4/16/2019 1:00	A	5799120.796	1380707.704	-73.858	-43.481	36664	0.306124736	-1.031076178	-0.791041841
172007	4/16/2019 2:26	1	25886771.14	2430841.36	-73.736	-43.482	5169	-0.910424721	-0.604589004	-1.001247352
172007	4/16/2019 3:07	1	5271736.266	176996.7338	-73.678	-43.469	2441	-0.140343954	-0.432102626	-0.992011979
172007	4/16/2019 13:24	B	310917802	17134694.49	-73.179	-43.181	36995	-0.636132257	0.384553051	-1.092731468

172007	4/17/2019 0:39	A	15272939.7	951932.8047	-73.02	-43.161	40545	0.454707282	0.319513859	-0.341834249
172007	4/17/2019 1:47	B	469947656	9286889.016	-72.985	-43.125	4065	0.093632301	0.250017188	-0.211925928
172007	4/17/2019 2:09	A	2748507.432	4275.067872	-72.95	-43.11	1299	-0.127282868	0.226545371	-0.236597578
172007	4/17/2019 2:44	3	979195.186	31037.81404	-72.952	-43.087	2084	0.348009864	0.186435342	-0.287802737
172007	4/17/2019 4:26	B	2028590.791	465130.2088	-72.907	-43.045	6136	0.093315581	0.098298281	-0.157082787
172007	4/17/2019 9:24	1	6179440.801	728661.6992	-73.08	-43.173	17864	-0.74382454	0.344503769	-0.429835525
172007	4/17/2019 10:18	B	35370935.86	40442870.64	-73.089	-43.176	3236	-0.842825501	0.360581692	-0.421358024
172007	4/17/2019 11:08	A	78288.66001	29553.83999	-73.088	-43.21	3022	-1.044031209	0.394883377	-0.40641104
172007	4/17/2019 11:38	B	1361964.475	383392.5248	-73.085	-43.213	1827	-1.063694128	0.395691639	-0.40520363
172007	4/18/2019 0:11	1	18561928.9	845856.0998	-72.986	-43.203	45182	0.214146687	0.370971162	-0.52310218
172007	4/18/2019 1:41	B	2467708213	111494909.7	-72.992	-43.178	5354	0.277414769	0.343008519	-0.48229207
172007	4/18/2019 2:25	A	12822.75873	70137.24127	-73.008	-43.169	2669	0.058969985	0.330982395	-0.457403661
172007	4/18/2019 3:21	B	1696482	428738	-72.984	-43.161	3327	-0.335967898	0.316589979	-0.438691416
172007	4/18/2019 3:27	B	1089918.395	2198074.605	-72.97	-43.155	367	-0.499531447	0.308682948	-0.450668416
172007	4/18/2019 4:09	A	23258.26034	19794.23966	-72.988	-43.133	2517	-0.294798134	0.262452223	-0.449849497
172007	4/18/2019 9:15	1	32792416.19	1243734.307	-73.151	-43.143	18412	0.314149542	0.310142029	-0.557096863

172007	4/19/2019 1:28	B	489404629.8	23692914.74	-73.126	-43.181	58331	0.430847817	0.359879928	-0.51212306
172007	4/19/2019 3:05	A	1508979.05	48381.45021	-73.07	-43.176	5847	0.455880559	0.346802686	-0.553885185
172007	4/19/2019 3:47	A	11107.18791	294822.8121	-73.083	-43.151	2523	0.374198444	0.309936539	-0.617797827
172007	4/19/2019 9:00	A	158897529.2	3139877.253	-73.212	-43.146	18762	0.456211604	0.33489494	-0.658389679
172007	4/20/2019 1:01	A	34862.43374	30747.56626	-73.33	-43.204	57668	0.602943061	0.310797771	-1.09238508
172007	4/20/2019 2:42	B	578001.8099	4494478.69	-73.318	-43.205	6062	0.578886927	0.319299824	-1.049507658
172007	4/20/2019 8:46	B	190769044.5	11877938	-73.087	-42.884	21832	-0.586601873	-0.170509388	-1.124213747
172007	4/20/2019 10:43	1	3643342.535	102213.465	-73.157	-43.059	6997	-0.221898104	0.17756618	-1.124598694
172007	4/20/2019 11:14	B	1.08276E+11	828779613.4	-73.041	-43.044	1915	-0.246992234	0.170960997	-1.125944865
172007	4/21/2019 0:37	B	11168134.87	152829366.1	-72.925	-43	48149	-0.620756722	0.011951538	-0.849597557
172007	4/21/2019 2:25	A	17863071.98	228982.5241	-73.008	-43.004	6492	-0.417225847	0.017949962	-0.967307955
172007	4/21/2019 2:46	B	52616020.62	6266892.376	-73.01	-43.003	1267	-0.420101941	0.016518484	-0.973147063
172007	4/21/2019 3:03	A	704197596	31818060.49	-72.978	-42.991	984	-0.434395226	0.001224645	-0.975171104
172007	4/21/2019 8:36	A	108164.8741	94435.12591	-72.972	-43.006	19992	-0.478419595	0.017680555	-0.914131376
172007	4/21/2019 10:16	B	2062480.5	351960.5	-73.007	-43.022	6026	-0.372383128	0.055376099	-0.955655766
172007	4/21/2019 10:40	B	24018998.31	21763.68878	-73.009	-43.041	1420	-0.336577679	0.093042595	-0.928943464

172007	4/22/2019 0:14	0	4040044328	125612424.5	-72.963	-42.98	48819	-0.436814589	-0.004714676	-0.594655011
172007	4/22/2019 2:04	2	737398.8432	57043.65681	-72.943	-43.025	6608	-0.477997597	0.059848734	-0.604259527
172007	4/22/2019 2:31	A	2447133.897	52852.1028	-72.935	-43.02	1629	-0.495151717	0.046214478	-0.588469129
172007	4/22/2019 2:46	A	1995559.439	187753.0612	-72.965	-43.028	883	-0.37661115	0.066797481	-0.638395587
172007	4/22/2019 3:42	B	1017735.583	276826.4168	-72.982	-43.034	3386	-0.208466376	0.07935741	-0.687418863
172007	4/22/2019 4:22	B	4179254.773	489917.2265	-72.965	-43.014	2401	-0.364145875	0.034699341	-0.633091054
172007	4/22/2019 8:27	1	20872861.17	1499957.327	-73.002	-43.056	14687	-0.038367061	0.122265747	-0.757347576
172007	4/22/2019 9:53	B	2766900.721	360716.2788	-73.012	-43.072	5198	0.009450394	0.168743888	-0.795876395
172007	4/22/2019 10:02	B	3229532.397	375120.1026	-73.016	-43.075	538	0.0267974	0.172600142	-0.806970995
172007	4/23/2019 1:42	B	172127356.9	90862545.61	-72.94	-43.028	56376	0.455491103	0.049678855	-0.591439098
172007	4/23/2019 2:22	B	3941997.763	42123904.74	-72.929	-43.017	2409	0.70016768	0.042426225	-0.625545968
172007	4/23/2019 3:21	A	48870538.21	5175714.795	-72.951	-43	3514	0.741076573	0.00793963	-0.64762012
172007	4/23/2019 8:14	A	68259.59011	255334.9099	-73.068	-43.078	17583	0.718288116	0.186347138	-0.87364273
172007	4/23/2019 9:55	A	4119108.271	1072452.729	-73.091	-43.075	6065	0.268015164	0.193630656	-0.960136811
172007	4/23/2019 11:07	A	2188219.163	117467.3369	-73.114	-43.094	4345	0.395931177	0.230211026	-1.01490374
172007	4/23/2019 11:18	B	19231926.72	727115.7825	-73.111	-43.094	620	0.48204922	0.231081866	-1.005932298

172007	4/23/2019 12:05	A	10991023.62	3975508.884	-73.102	-43.106	2860	1.212044328	0.239736979	-0.935264811
172007	4/23/2019 13:05	A	394848974.3	32479368.22	-73.053	-43.104	3557	0.991115497	0.228675772	-0.81169142
172007	4/23/2019 13:44	A	1541267785	82156240.15	-73.04	-43.105	2376	0.707179482	0.224527026	-0.741411719
172007	4/23/2019 13:45	B	42099778.55	1907962.451	-73.041	-43.104	76	0.661658871	0.226831868	-0.736485075
172007	4/24/2019 1:00	A	1324736.407	33863.5931	-72.983	-43.034	40459	0.036782033	0.07935741	-0.453321493
172007	4/24/2019 1:23	A	49578.83109	63203.66891	-72.978	-43.033	1389	0.146726621	0.071873216	-0.46130011
172007	4/24/2019 1:58	A	905347.1349	86045.36509	-72.983	-43.01	2105	0.646509952	0.031681072	-0.50656923
172007	4/24/2019 2:07	2	1437434.762	15575.23752	-72.966	-43.012	542	0.965361277	0.030531029	-0.532936067
172007	4/24/2019 2:35	B	6120985.96	217780.5405	-72.978	-43.013	1701	0.686742266	0.033902892	-0.505901028
172007	4/24/2019 3:02	B	44681860.13	60530.36976	-72.981	-43.014	1569	0.690681474	0.035809696	-0.505079522
172007	4/24/2019 3:40	A	67711068.94	5576917.063	-72.945	-42.982	2296	1.094020893	-0.02962738	-0.674447556
172007	4/24/2019 8:03	B	1804056365	4988425.085	-72.897	-43.029	15804	0.377022858	0.072792427	-0.467427828
172007	4/24/2019 10:42	1	493452.375	192132.125	-73.022	-43.127	9550	-0.350863298	0.257589141	-0.55680924
172007	4/24/2019 10:48	B	1810059.304	382251.1957	-73.024	-43.125	329	-0.260876999	0.256379235	-0.563870256
172007	4/25/2019 0:38	1	3920597.007	937859.9928	-72.976	-43.086	49782	-0.523353486	0.186785811	-0.391947013
172007	4/25/2019 1:53	B	9981517.448	2665445.052	-72.976	-43.09	4541	-0.622800273	0.197450568	-0.388564273

172007	4/25/2019 2:40	B	56510554747	3512951193	-73.028	-43.153	2803	-0.811683684	0.245720499	-0.386172655
172007	4/25/2019 3:25	B	77553814.78	3626621.716	-73.022	-43.15	2697	-0.750494723	0.294937951	-0.398542104
172007	4/25/2019 10:19	B	42804562.52	5631767.484	-73.04	-43.105	24834	0.344653411	0.22398081	-0.457809476
172007	4/25/2019 11:54	B	20097931.14	2646822.859	-73.028	-43.096	5737	0.503585598	0.206688155	-0.455022144
172007	4/25/2019 12:24	B	1704833.263	2754296.737	-73.026	-43.085	1760	0.858301858	0.191884157	-0.481066611
172007	4/25/2019 13:07	B	1073207.242	1121923.258	-73.018	-43.083	2578	0.708917034	0.189536484	-0.458675035
172007	4/25/2019 13:24	B	1218387.028	442521.4721	-73.018	-43.089	1062	0.612632222	0.196098842	-0.454903506
172007	4/26/2019 0:14	A	2849075523	177113326.4	-72.949	-42.98	38974	-0.420199832	-0.011855373	-0.308665181
172007	4/26/2019 1:44	B	76369442.12	1640957.879	-72.969	-42.988	5403	-0.431033659	-0.01895877	-0.308066197
172007	4/26/2019 2:23	B	39753667659	599328501.2	-72.955	-42.987	2332	-0.344153515	-0.013649766	-0.329448981
172007	4/26/2019 2:58	1	9662296.725	453961.7754	-73.007	-42.993	2078	-0.261049624	-0.004016536	-0.357438445
172007	4/26/2019 9:19	B	3076008747	453274693.1	-73.096	-42.984	22905	-0.211139115	-0.007358721	-0.436225862
172007	4/26/2019 11:26	B	791741321.7	6061248.776	-73.094	-42.994	7577	-0.218972938	0.008779772	-0.452861763
172007	4/27/2019 1:31	A	239913516.4	6020570.124	-72.949	-42.987	50738	-0.395748978	-0.021218226	-0.550028838
172007	4/27/2019 2:01	2	851276.5919	5613.408132	-72.976	-43.008	1813	-0.454000362	0.024758906	-0.548626525
172007	4/27/2019 2:39	3	510148.634	54573.36605	-72.968	-43.031	2264	-0.545162395	0.068012949	-0.536047997

172007	4/27/2019 3:14	B	719261.0062	156143.9938	-72.969	-43.039	2101	-0.566417001	0.0849116	-0.536433783
172007	4/27/2019 3:40	B	8988999.191	11894140.81	-72.975	-43.044	1536	-0.565157994	0.09604863	-0.545766993
172007	4/27/2019 9:07	0	5044277926	76733482.19	-73.272	-43.1	19617	-0.527494032	0.201470071	-0.627831356
172007	4/27/2019 10:53	B	6667604185	131944460.4	-73.295	-43.095	6395	-0.508214112	0.185353392	-0.648933029
172007	4/28/2019 1:22	B	127033844.7	18769677.77	-73.06	-42.829	52134	1.924858565	-0.478982607	-0.730124078
172007	4/28/2019 1:40	B	5176163.952	1365317.048	-73.05	-42.824	1079	1.870056579	-0.512084431	-0.713756522
172007	4/28/2019 2:18	B	7502593.166	2733734.834	-73.025	-42.845	2297	1.863282576	-0.455093919	-0.815465735
172007	4/28/2019 3:19	B	2832571.29	790808.7101	-73.015	-42.864	3650	1.537433113	-0.392976996	-0.888927378
172007	4/28/2019 8:55	B	16429606.73	4846093.771	-72.901	-42.904	20164	0.143970475	-0.277267475	-0.698572089
172007	4/28/2019 10:43	B	287489044	217244732.5	-72.869	-42.917	6430	-0.158492716	-0.199593107	-0.701731732
172007	4/28/2019 12:05	B	2820619.62	326270.38	-72.862	-42.96	4939	-0.330807128	-0.092501716	-0.714698866
172007	4/28/2019 12:49	B	25302909.56	475082.9408	-72.847	-42.952	2657	-0.384774438	-0.107619791	-0.683792341
172007	4/28/2019 13:43	B	6016375082	227321343.3	-72.82	-42.956	3204	-0.36694885	-0.116680355	-0.6855041
172007	4/29/2019 0:34	B	22680305.42	3767233.079	-72.905	-42.902	39110	0.356693753	-0.28196167	-0.778104803
172007	4/29/2019 1:10	B	56281942.55	2180673.953	-72.859	-42.906	2158	0.031088226	-0.277587811	-0.741521851
172007	4/29/2019 2:54	B	1239236870	380772498.6	-72.936	-42.93	6221	0.345338862	-0.18858204	-0.898250198

172007	4/29/2019 3:39	B	985900.5633	2619879.437	-72.925	-42.946	2713	0.352924264	-0.125531677	-0.964366934
172007	4/29/2019 8:45	B	6749389.078	1503221.422	-72.948	-43.009	18364	-0.16416784	0.024202254	-1.061228248
172007	4/29/2019 10:18	A	14668078.72	920127.7769	-73.013	-42.979	5583	-0.137221595	-0.039520657	-1.218916653
172007	4/29/2019 11:34	B	14619519.14	1046077.859	-72.962	-43.01	4558	-0.198835439	0.024550563	-1.097171595
172007	4/29/2019 11:57	B	42454438.26	161402.2395	-72.963	-42.962	1390	0.308498289	-0.081778526	-1.128263922
172007	4/29/2019 13:20	B	24213840.5	395160.5	-72.979	-42.972	4927	0.116561969	-0.053916748	-1.166662171
172007	4/29/2019 14:21	B	14458384.1	791040.8995	-72.986	-42.976	3692	0.008032789	-0.048447233	-1.177783269
172007	4/30/2019 0:12	B	3.01087E+11	7553048831	-73.04	-42.823	35474	-0.285092199	-0.10173923	-1.186133321
172007	4/30/2019 1:36	B	44568343.16	2242634.839	-73.006	-42.947	5016	-0.316090794	-0.108804395	-1.192950923
172007	4/30/2019 2:39	B	1408029537	818828862.9	-73.13	-42.88	3758	1.159211883	-0.295802144	-1.156419797
172007	4/30/2019 3:18	B	325391.96	2651702.54	-73.138	-42.819	2370	1.65575269	-0.431773944	-0.86575911
172007	4/30/2019 8:35	1	208168.0084	560768.4916	-73.194	-42.937	18996	-0.576080381	-0.044343081	-1.38322729
172007	4/30/2019 10:12	B	71819674.42	20332410.58	-73.233	-42.923	5822	-0.631572052	-0.087579725	-1.381371059
172007	4/30/2019 11:01	2	679738.3112	81824.1888	-73.124	-42.897	2932	0.024279053	-0.188272333	-1.307473137
172007	4/30/2019 11:29	B	35123661.31	2637327.189	-73.134	-42.915	1731	-0.457357977	-0.1370815	-1.344264285
172007	5/1/2019 2:16	A	5153650.599	2351954.401	-73.118	-42.839	53204	-0.184479533	-0.347141473	-0.993580241

172007	5/1/2019 2:24	A	484807.7127	12842.2873	-73.14	-42.849	494	-0.201398671	-0.307842127	-1.027614912
172007	5/1/2019 2:57	0	835337227.7	37768738.82	-73.187	-42.857	1981	-0.204333626	-0.292224256	-1.056979814
172007	5/1/2019 4:00	B	56135707.43	8259272.568	-73.199	-42.851	3754	-0.168036703	-0.277159849	-1.082525525
172007	5/1/2019 10:04	B	269244354.2	28034020.26	-73.247	-42.877	21813	-0.285830878	-0.195949273	-1.188651059
172007	5/1/2019 10:29	B	365488499	17449333	-73.28	-42.837	1531	-0.117961392	-0.246204378	-1.151047193
172007	5/2/2019 1:01	B	56076345.18	5225051.321	-73.099	-42.968	52306	-0.412009062	-0.021770829	-1.008316568
172007	5/2/2019 1:54	B	654845750	7243432.504	-73.08	-42.949	3213	-0.785483341	-0.061081022	-1.027043596
172007	5/2/2019 2:38	A	53458263.16	591153.8356	-73.089	-42.968	2635	-0.465964173	-0.028116132	-1.013174912
172007	5/2/2019 3:36	B	6620198.662	4663263.838	-73.059	-42.958	3461	-0.797703834	-0.063888498	-1.03421399
172007	5/2/2019 8:09	B	8815208.122	875737.8779	-73.035	-42.994	16384	-0.693539822	0.004207652	-1.020975367
172007	5/2/2019 11:41	B	27644314.28	1585077.719	-73.027	-43.007	12719	-0.755407757	0.029654583	-1.025508478
172007	5/2/2019 12:19	B	58226446.76	3904173.743	-73.011	-42.983	2300	-0.86521463	-0.017227636	-1.042878982
172007	5/3/2019 0:34	A	4380154.591	27114.40903	-73.028	-43.003	44099	-0.32391361	0.023937138	-0.929253989
172007	5/3/2019 1:34	A	17230781.22	4107599.785	-73.03	-43.013	3566	-0.296954215	0.044067147	-0.929629062
172007	5/3/2019 2:04	B	46810338.69	3374086.314	-73.025	-42.987	1822	-0.511444963	-0.010605435	-0.947733981
172007	5/3/2019 2:13	A	23710.07569	9818.924306	-73.023	-42.991	541	-0.477222268	-0.001557371	-0.945250378

172007	5/3/2019 3:56	B	2145514.574	336758.4262	-73.018	-43	6180	-0.420337783	0.016107767	-0.944717058
172007	5/3/2019 7:58	B	1609673.673	29912448.33	-72.952	-43.01	14489	1.081657242	0.024347126	-0.949574052
172007	5/4/2019 1:49	A	76673.09662	18976.90338	-73.038	-43.059	64310	-1.024260978	0.132763139	-0.992042968
172007	5/4/2019 1:55	A	60396.21414	278642.2859	-73.051	-43.057	316	-0.921550695	0.135765647	-0.986202423
172007	5/4/2019 3:36	B	6884222.136	933048.3644	-73.035	-43.036	6094	-0.984693529	0.090101806	-0.986559367
172007	5/4/2019 9:28	B	28697239.46	1060770.54	-72.973	-43.072	21096	-0.653440842	0.166632203	-0.958554956
172007	5/4/2019 11:30	B	53542529089	65413864.53	-73.101	-43.236	7353	0.304192767	0.396680071	-0.712684322
172007	5/4/2019 12:38	B	6978643.348	51241985.65	-73.1	-43.252	4028	-0.185058859	0.430673107	-0.723726518
172007	5/4/2019 13:22	B	12900926	17241366.5	-73.104	-43.265	2655	-0.374067343	0.437470978	-0.702923497
172007	5/4/2019 14:56	B	1766352.816	1922225.684	-73.109	-43.287	5632	-0.707656076	0.431243988	-0.674753318
172007	5/5/2019 1:39	2	117489.921	49896.57903	-73.328	-43.328	38607	0.182500019	0.245831947	-0.580743089
172007	5/5/2019 2:38	B	1936086.885	541225.115	-73.337	-43.342	3553	0.227560766	0.227467793	-0.580931512
172007	5/5/2019 3:12	B	2180792.793	1232187.707	-73.339	-43.342	2041	0.264259766	0.225363725	-0.594541357
172007	5/5/2019 9:11	A	872993708.2	62694882.31	-73.242	-43.181	21530	0.023163675	0.306656044	-0.84538032
172007	5/5/2019 11:06	B	259363.1843	681497.3157	-73.365	-43.202	6916	-0.012550949	0.2798771	-0.884611704
172007	5/5/2019 12:16	B	1310183.932	724464.5684	-73.39	-43.207	4156	-0.010564807	0.244554105	-0.93232446

172007	5/5/2019 12:57	A	10900643.61	95822.8879	-73.356	-43.281	2479	0.232110917	0.231645778	-0.746447291
172007	5/5/2019 14:02	B	16183134.48	1292756.02	-73.307	-43.347	3869	-0.04612105	0.253646174	-0.508666177
172007	5/5/2019 14:39	B	16698852.64	1573049.86	-73.305	-43.38	2253	-0.384542593	0.248858195	-0.445714649
172007	5/6/2019 1:29	B	292668659.2	15965865.81	-73.283	-43.423	38984	-0.799648	0.24118338	-0.437880749
172007	5/6/2019 2:11	B	98779956.86	14912255.64	-73.322	-43.446	2517	-0.783555774	0.235238222	-0.43596274
172007	5/6/2019 9:03	B	1241408581	202945619.6	-73.492	-43.381	24737	0.45499946	0.129366755	-0.749796281
172007	5/6/2019 12:59	B	88213256307	982525750.1	-73.666	-43.405	14127	-0.190914951	0.14660194	-0.839866159
172007	5/6/2019 14:16	B	172584239.1	41168053.89	-73.414	-43.26	4675	-0.041018614	0.171796587	-0.901527749
172007	5/7/2019 1:51	B	1.08025E+11	3362063760	-73.386	-43.308	41663	0.132567269	0.200648983	-0.776659783
172007	5/7/2019 2:34	B	48259477813	817920.5	-73.306	-43.268	2603	0.203105971	0.205952585	-0.782310734
172007	5/7/2019 2:57	B	1850448613	9078060.5	-73.285	-43.25	1372	0.759677679	0.218061991	-0.83523821
172007	5/7/2019 8:52	1	891555.6527	67069.34726	-73.42	-43.185	21310	1.126687606	0.227345307	-1.202913485
172007	5/7/2019 12:46	B	396930898.7	162232317.3	-73.422	-43.201	14038	0.866771917	0.211285383	-1.097192513
172007	5/7/2019 13:16	B	102478829.1	10830859.87	-73.414	-43.219	1785	0.904470301	0.209160195	-1.067630746
172007	5/8/2019 0:10	B	235388664.2	91914648.3	-73.442	-43.139	39238	0.241895281	0.273601059	-1.33545462
172007	5/8/2019 1:31	B	28432219.78	2150792.725	-73.378	-43.171	4840	0.768853198	0.280896429	-1.230024413

172007	5/8/2019 2:13	A	31403495.56	8191716.938	-73.41	-43.122	2549	-0.384018609	0.30724601	-1.402035242
172007	5/8/2019 2:45	B	3523858.636	10856397.86	-73.378	-43.116	1945	-0.374970815	0.321851588	-1.393634322
172007	5/8/2019 3:11	B	3183317.154	2989891.346	-73.381	-43.113	1528	-0.442315745	0.322586174	-1.40016 77
172007	5/8/2019 3:53	B	8790895.536	4140146.464	-73.385	-43.11	2528	-0.539716467	0.318188575	-1.409916369
172007	5/8/2019 8:39	B	105602275.5	35204865.5	-73.407	-43.041	17131	-1.148503525	0.271676399	-1.462814658
172007	5/8/2019 9:53	B	5806583.58	2821014.92	-73.425	-43.03	4465	-1.16819806	0.26342069	-1.467208009
172007	5/9/2019 1:10	B	28449444.5	1458220.5	-73.245	-43.036	54995	-0.858070667	0.18278433	-1.07276427
172007	5/9/2019 1:49	B	43296117.67	1326447.329	-73.244	-43.036	2366	-0.853287176	0.18278433	-1.070200638
172007	5/9/2019 2:34	B	16192005.71	5003824.795	-73.244	-43.034	2675	-0.858009498	0.183257127	-1.07304528
172007	5/9/2019 2:49	B	13065242.11	2591366.886	-73.254	-43.041	953	-0.85858881	0.19559009	-1.076112435
172007	5/9/2019 3:31	B	578825168.3	714036.232	-73.272	-43.067	2467	-0.813782274	0.241437602	-1.064279916
172007	5/9/2019 8:29	B	28770447.22	1373796.779	-73.24	-43.075	17916	-0.803924105	0.258009588	-1.04513325
172007	5/9/2019 11:07	B	35306874.89	1807213.607	-73.246	-43.055	9469	-0.836317008	0.209977901	-1.049361091
172007	5/9/2019 11:23	B	91607457.07	3473127.93	-73.197	-43.06	945	-0.84177411	0.212720836	-1.03392244
172007	5/9/2019 12:23	B	6644861.287	256003.7131	-73.185	-43.061	3594	-0.875355773	0.197036949	-1.004261354
172007	5/9/2019 13:18	B	6375665.494	483059.5063	-73.183	-43.061	3322	-0.881009512	0.198406018	-1.000051742

172007	5/9/2019 14:13	B	512853609.4	3929019.067	-73.189	-43.029	3274	-0.85805775	0.146146343	-0.996268376
172007	5/10/2019 0:58	B	1542029087	47949403.02	-73.106	-43.022	38743	-1.26597245	0.068493771	-1.125268981
172007	5/10/2019 2:28	0	21789588331	984461916.8	-73.056	-43.01	5399	-1.25808183	0.057917256	-1.122228644
172007	5/10/2019 3:10	B	1010502880	1235228.87	-73.047	-43.016	2522	-1.255921679	0.047511473	-1.120865599
172007	5/10/2019 8:16	A	137405.1203	416133.3797	-72.937	-42.97	18359	-1.218211683	-0.061421511	-1.102202015
172007	5/10/2019 10:51	B	75118655.11	28503.38694	-72.91	-42.97	9257	-1.215866509	-0.060011702	-1.098272239
172007	5/10/2019 12:13	B	13147796.25	219944.7479	-72.916	-42.974	4949	-1.218232093	-0.057386518	-1.098998376
172007	5/10/2019 12:56	B	9996603.755	385846.7446	-72.909	-42.972	2576	-1.217044228	-0.058933169	-1.098403964
172007	5/10/2019 14:35	B	135294034.2	440304286.3	-73.049	-43.003	5941	-1.229658999	-0.048084254	-1.113486045
172007	5/11/2019 0:35	B	13244314875	411835417.9	-73.015	-42.854	35970	-0.295711165	-0.336077537	-0.831650063
172007	5/11/2019 2:09	A	406679.6982	99065.30176	-73.014	-42.881	5668	-0.294777373	-0.334396697	-0.832976311
172007	5/11/2019 2:12	B	96158.96739	25502.03261	-73.014	-42.881	208	-0.294777373	-0.334396697	-0.832976311
172007	5/11/2019 2:48	B	747864.5	186050	-73.02	-42.884	2156	-0.304635595	-0.314350499	-0.849035879
172007	5/11/2019 8:06	B	7870802.444	1572314.556	-73.008	-42.872	19036	-0.273219401	-0.376366583	-0.809143884
172007	5/12/2019 0:11	B	121220344	3405616.464	-72.974	-42.967	57911	-0.449490008	-0.069306691	-0.995393243
172007	5/12/2019 1:52	B	34447271.17	4479161.825	-72.99	-42.909	6045	0.233425953	-0.246453375	-0.890275099

172007	5/12/2019 2:31	B	655473497.4	61768603.15	-73.025	-42.958	2345	-0.177244165	-0.130832476	-0.97137221
172007	5/12/2019 3:08	B	868585345.2	103299955.3	-73.009	-42.961	2254	-0.338771849	-0.092620554	-0.98695224
172007	5/12/2019 3:29	B	86642382.12	152577194.9	-72.972	-42.96	1235	-0.4147449	-0.076527062	-0.991677049
172007	5/12/2019 9:35	B	24625831.39	512361.614	-72.992	-43.081	21956	-0.525581566	0.18138691	-1.010785775
172007	5/13/2019 1:47	B	225580990	4793260.473	-72.988	-43.094	58314	0.677235913	0.203724591	-1.056956373
172007	5/13/2019 2:08	B	78575126.11	6174778.386	-73.02	-43.109	1291	0.54077375	0.229795736	-1.025026817
172007	5/13/2019 2:41	B	105410963.3	9483814.668	-73.013	-43.113	1995	0.483440466	0.232945891	-0.976191965
172007	5/13/2019 3:47	B	317689314.3	2443142.173	-72.921	-43.146	3951	-0.078117349	0.288237826	-0.904136848
172007	5/13/2019 9:20	B	943228270.1	58642134.89	-72.687	-43.147	19945	1.41511694	0.250023791	-1.097227745
172007	5/13/2019 10:56	B	124940281.9	1017768.131	-72.916	-43.029	5773	-0.012220742	0.068669094	-1.255228789
172007	5/13/2019 13:15	B	53515624.48	13897760.52	-73.083	-42.96	8341	0.596372405	-0.049246406	-1.304841584
172007	5/13/2019 13:34	B	20781567.56	3401488.942	-73.108	-42.939	1172	0.070710335	-0.086688389	-1.31176109
172007	5/14/2019 2:44	A	2052784.153	6284.346532	-73.238	-43.049	47376	0.469107003	0.203454137	-1.634887636
172007	5/14/2019 3:28	A	16059.29715	98536.70285	-73.268	-43.061	2664	0.580002239	0.230052985	-1.684014493
172007	5/14/2019 9:10	A	1374720217	42746551.41	-73.115	-43.078	20508	1.137766598	0.217996686	-1.231972161
172007	5/14/2019 10:40	B	126198573.9	5790604.617	-73.123	-43.055	5405	0.361840391	0.169548401	-1.206574544

172007	5/14/2019 12:06	B	2362500649	385654299.8	-73.137	-43.02	5128	1.172110984	0.181692652	-1.274446429
172007	5/15/2019 2:27	B	83910107972	410511720.7	-73.171	-43.267	51687	-0.558840634	0.258613485	-1.051959737
172007	5/15/2019 3:07	A	189351566.3	538258.7416	-73.345	-43.325	2404	-0.504612889	0.241027254	-1.035154083
174067	2/4/2019 11:17	A	1279827.679	10550.82074	-73.569	-44.441	14507	-0.106805612	-1.424079169	1.304747613
174067	2/4/2019 13:15	B	1318438.577	1408063.923	-73.571	-44.441	7093	0.077391811	-1.859177661	1.367870169
174067	2/4/2019 15:17	A	104562.1746	68191.82545	-73.33	-44.478	7335	-0.697258279	-2.834404816	1.562634238
174067	2/4/2019 17:02	B	21867453.45	5315585.049	-73.27	-44.485	6319	-0.747234444	-2.555802755	1.553735171
174067	2/4/2019 18:04	B	26576873.62	67813430.38	-73.362	-44.454	3707	-0.76682161	-2.489167045	1.560728181
174067	2/5/2019 1:17	B	194080270.7	5226645.283	-73.35	-44.574	25948	0.214884134	-1.189998458	1.291487907
174067	2/5/2019 2:17	0	4328248795	65358913.4	-73.421	-44.701	3599	0.155991205	-0.929033979	1.183846656
174067	2/5/2019 3:19	B	5402333637	204167916.1	-73.439	-44.752	3749	-0.007100545	-0.624154562	1.067696127
174067	2/5/2019 5:13	A	30484.48668	176296.0133	-73.354	-44.741	6837	-0.136756871	-0.740723335	0.948936107
174067	2/5/2019 6:19	B	19986600.02	1474544.479	-73.343	-44.741	3963	-0.148667624	-0.747841543	0.953184333
174067	2/5/2019 11:04	3	112457.8086	15492.69138	-73.358	-44.736	17125	-0.123465336	-0.716212017	0.954759393
174067	2/5/2019 15:04	3	1004138.62	2147.879634	-73.441	-44.819	14347	-0.251410203	-2.215732457	0.746025914
174067	2/5/2019 16:06	2	2000753.027	43059.47291	-73.346	-44.898	3747	-0.029033635	-2.518454007	0.864032311

174067	2/5/2019 18:17	B	65157918.98	14291266.02	-73.283	-45.042	7874	0	-0.823925564	1.082126124
174067	2/6/2019 1:03	0	2120776651	53271409.19	-73.665	-45.02	24347	2.419246035	-1.506975405	0.600723198
174067	2/6/2019 4:19	B	84639538.62	116194893.9	-73.747	-45.017	11760	1.490145029	-1.338488441	0.651969187
174067	2/6/2019 5:02	B	6264193983	515071898.4	-73.804	-45.054	2597	1.399742552	-1.26628814	0.670739275
174067	2/6/2019 6:03	B	2530851805	4083340.033	-73.785	-45.03	3622	1.270925505	-1.16248719	0.674011409
174067	2/6/2019 8:14	1	2406430.655	1101897.845	-73.699	-45.022	7904	1.159179489	-1.051326042	0.679438585
174067	2/6/2019 14:06	0	100554103.2	6339621.789	-73.683	-45.077	21067	1.488972044	-0.767911008	0.923890411
174067	2/6/2019 14:12	B	81277056.82	5908945.684	-73.675	-45.074	405	1.488972044	-0.767911008	0.923890411
174067	2/6/2019 16:18	A	466979.6389	88321.36113	-73.673	-45.062	7569	1.52774264	-0.877026092	0.870362581
174067	2/6/2019 23:12	B	10535178.86	939029.6363	-73.614	-45.057	24821	1.95951629	-0.998962971	0.863023807
174067	2/6/2019 23:30	B	59605458.11	344676.3946	-73.64	-45.064	1076	1.958752862	-0.94599953	0.891972618
174067	2/7/2019 1:12	0	10558827196	51666392.38	-73.62	-45.092	6131	-0.133720202	-0.772780443	0.077633289
174067	2/7/2019 2:35	B	145556866.5	6545745.994	-73.604	-45.093	4967	0.006336898	-0.707087764	0.09537264
174067	2/7/2019 3:07	A	72429.64626	92635.35374	-73.624	-45.094	1917	0.032432019	-0.683429521	0.09973758
174067	2/7/2019 4:12	1	625180.7316	63669.76842	-73.614	-45.102	3918	0.054989153	-0.628421163	0.099565054
174067	2/7/2019 4:29	A	529248.25	529248.25	-73.604	-45.105	976	0.042505305	-0.621705935	0.096806707

174067	2/7/2019 5:37	1	260463339.7	9844048.763	-73.62	-45.099	4125	0.03841197	-0.620144099	0.096127897
174067	2/7/2019 6:11	1	5397289.286	340208.7139	-73.622	-45.104	2031	0.042837778	-0.620952907	0.097783275
174067	2/7/2019 6:30	B	2034559.802	581825.1983	-73.605	-45.111	1127	0.042837778	-0.620952907	0.097783275
174067	2/7/2019 7:14	A	60281113.31	497578.6928	-73.576	-45.092	2626	-0.080384595	-0.678929535	0.075867762
174067	2/7/2019 10:41	B	41523937.14	6654185.357	-73.539	-45.101	12461	0.02914768	-0.686312771	0.099922867
174067	2/7/2019 12:25	A	2838347.944	419572.0562	-73.677	-45.071	6213	0.149744494	-0.767911008	0.165093642
174067	2/7/2019 13:46	1	2347422.581	1023797.419	-73.732	-45.048	4896	0.26340916	-0.760759658	0.241085848
174067	2/7/2019 14:37	B	4975556.44	198020.5599	-73.703	-45.055	3006	0.285984164	-0.723050591	0.265087377
174067	2/7/2019 15:02	A	17730422.89	2103383.608	-73.739	-45.032	1498	0.286176951	-0.736047754	0.274123103
174067	2/7/2019 15:20	A	855802.4311	315146.0689	-73.745	-45.035	1130	0.266457404	-0.742854096	0.266764171
174067	2/7/2019 15:27	A	667778.2369	16726.26306	-73.745	-45.036	388	0.270225777	-0.742087171	0.273578444
174067	2/7/2019 15:59	B	2033866.895	154095.605	-73.751	-45.033	1916	0.270441183	-0.749044918	0.275072119
174067	2/7/2019 16:20	3	24294.91478	43489.08522	-73.735	-45.038	1243	0.257665617	-0.757322839	0.255997343
174067	2/7/2019 16:29	A	2162733.942	562726.0579	-73.738	-45.04	577	0.251040388	-0.773976784	0.245048056
174067	2/7/2019 16:58	A	12143.97982	70548.02018	-73.709	-45.053	1734	0.223288469	-0.816792706	0.210990539
174067	2/7/2019 17:33	B	768040.1285	147241.8715	-73.712	-45.051	2095	0.208119761	-0.821805259	0.20280956

174067	2/7/2019 18:43	B	2109131.129	368951.3714	-73.718	-45.05	4210	0.241975068	-0.789278802	0.226464863
174067	2/7/2019 23:01	B	6343650.612	1543841.888	-73.738	-45.036	15480	0.23847019	-0.782859904	0.240280979
174067	2/8/2019 0:39	B	7057319.666	29593818.83	-73.723	-45.049	5891	0.09274586	-0.841075668	0.592688842
174067	2/8/2019 2:19	1	1412108.465	539767.5353	-73.664	-45.054	5976	0.234397309	-0.97638212	0.585330651
174067	2/8/2019 3:41	1	1199341.058	19961.44206	-73.639	-45.076	4916	0.387455521	-0.806146824	0.6304758
174067	2/8/2019 4:12	B	8096288	1702012.5	-73.634	-45.078	1848	0.422995199	-0.847071261	0.62415615
174067	2/8/2019 4:39	A	107802.5463	19647.45373	-73.629	-45.069	1632	0.424732659	-0.858235936	0.609162686
174067	2/8/2019 5:14	A	2153614.405	177638.5953	-73.654	-45.075	2118	0.353793268	-0.826032132	0.626862273
174067	2/8/2019 6:18	A	9972578	10368	-73.657	-45.066	3835	0.282381669	-0.851288764	0.606932031
174067	2/8/2019 7:34	1	533942.3183	63747.68171	-73.668	-45.05	4530	0.229810091	-1.005474077	0.571895987
174067	2/8/2019 10:29	B	1116401.334	5839165.166	-73.666	-45.045	10502	0.230864074	-1.089803197	0.553460993
174067	2/8/2019 12:09	1	38131880.89	1724889.107	-73.631	-45.043	6038	0.355301277	-1.19563018	0.529629308
174067	2/8/2019 12:46	B	4551212.235	877412.7654	-73.627	-45.041	2181	0.40002007	-1.219881873	0.528986138
174067	2/8/2019 13:26	B	45041601.25	73915.75246	-73.629	-45.052	2450	0.442996922	-1.10775286	0.556894411
174067	2/8/2019 14:31	B	58977785.28	2231875.222	-73.622	-45.075	3871	0.454035215	-0.838473122	0.625127543
174067	2/8/2019 15:01	B	5580334.25	209030.7504	-73.62	-45.078	1798	0.446862895	-0.793689761	0.637283055

174067	2/8/2019 15:34	A	14036044.09	30825623.91	-73.655	-45.105	1998	0.435987001	-0.772780443	0.654850567
174067	2/8/2019 16:07	B	2548409.136	778143.3635	-73.619	-45.08	1986	0.451009533	-0.816143771	0.62970456
174067	2/8/2019 16:14	B	2477587.879	673910.6207	-73.626	-45.074	415	0.456766965	-0.842748126	0.627678057
174067	2/8/2019 16:38	A	475526118.8	34150206.16	-73.632	-45.085	1442	0.461903699	-1.007382892	0.565111842
174067	2/8/2019 17:20	B	101495274.6	422017.8773	-73.691	-45.034	2479	0.441999752	-1.20215994	0.530641293
174067	2/8/2019 17:47	B	5350479.402	956619.0982	-73.62	-45.077	1659	0.457461661	-0.98083293	0.579998545
174067	2/8/2019 18:24	B	42625992	11853338	-73.64	-45.086	2214	0.451009533	-0.816143771	0.62970456
174067	2/9/2019 0:29	B	60430.16083	1384132.339	-73.64	-45.042	21894	-0.112776299	-1.226257037	-0.323928274
174067	2/9/2019 2:09	A	25041.95881	100536.0412	-73.612	-45.014	5988	0.129761248	-1.726451622	-0.239218156
174067	2/9/2019 3:16	0	1065587913	48149455.15	-73.611	-44.998	4041	0.16392905	-1.921718665	-0.22127503
174067	2/9/2019 4:27	3	94372.17639	12828.82361	-73.614	-45.007	4241	0.132024523	-1.895794053	-0.227811917
174067	2/9/2019 5:27	1	4369113.055	296625.4445	-73.612	-45.013	3634	0.098615769	-1.725383951	-0.248515868
174067	2/9/2019 6:12	A	82852613.52	5497298.984	-73.599	-45.039	2659	0.078898228	-1.434202984	-0.275208959
174067	2/9/2019 6:32	B	14617872.99	1454897.507	-73.596	-45.043	1238	0.054749387	-1.386322849	-0.287901828
174067	2/9/2019 7:15	1	1363045.399	2298284.601	-73.624	-45.015	2546	0.034961654	-1.45469021	-0.274055056
174067	2/9/2019 10:16	B	83751488.98	12086519.52	-73.647	-45.005	10885	-0.011985328	-2.013011821	-0.244429098

174067	2/9/2019 14:14	B	57056834.93	40178217.57	-73.632	-44.965	14262	-0.019901138	-2.168532373	-0.186433794
174067	2/9/2019 14:36	B	6624640.622	4390116.378	-73.642	-44.958	1312	-0.027758826	-2.192466988	-0.183515283
174067	2/9/2019 15:18	B	42762019.26	1618413.736	-73.646	-44.957	2519	-0.066655053	-1.980690943	-0.184966974
174067	2/9/2019 15:33	B	2340010.062	1187060.438	-73.654	-44.952	936	-0.066655053	-1.96405895	-0.184966974
174067	2/9/2019 16:20	3	1622919.582	86872.91756	-73.654	-44.96	2799	-0.150565217	-1.962149993	-0.203214966
174067	2/9/2019 17:29	B	2569678.366	342981.6342	-73.666	-44.96	4121	-0.154137565	-1.962149993	-0.204885351
174067	2/9/2019 17:37	B	2989464.07	330348.4301	-73.662	-44.961	489	-0.119742638	-1.978713808	-0.197637714
174067	2/9/2019 18:37	B	1428008.078	4748890.422	-73.62	-44.954	3615	0.151336385	-2.427942067	-0.148281706
174067	2/9/2019 23:35	B	5853005.015	2757783.485	-73.6	-44.948	17873	0.364933593	-2.639387646	-0.103599125
174067	2/10/2019 0:14	B	35004151.8	374283322.7	-73.611	-44.997	2314	1.155718505	-2.680735251	-0.146302443
174067	2/10/2019 1:15	0	17856474584	555187483.6	-73.612	-45.038	3712	0.426636381	-2.405506023	-0.232873193
174067	2/10/2019 2:53	A	341212.1018	21099.89818	-73.634	-45.011	5875	0.456833249	-1.736786308	-0.283849603
174067	2/10/2019 4:17	2	142007.5647	27397.43533	-73.602	-44.988	5011	0.91731389	-2.454777986	-0.158006964
174067	2/10/2019 4:31	A	2885446.458	267323.542	-73.609	-44.986	869	1.099436438	-2.585376257	-0.130236177
174067	2/10/2019 5:08	A	31463326.45	158433.5526	-73.586	-44.976	2175	1.471192109	-2.752316785	-0.083865881
174067	2/10/2019 6:11	A	3493190.978	4301121.522	-73.579	-44.967	3803	2.039646105	-2.846125673	0.007287878

174067	2/10/2019 6:52	B	956663.5168	229418.4832	-73.573	-44.963	2448	2.226955559	-2.846125673	0.059640647
174067	2/10/2019 10:03	B	5961604.5	609408	-73.542	-44.946	11484	2.297425401	-2.866799476	0.307479317
174067	2/10/2019 12:39	B	8305609.433	1683035.567	-73.534	-44.938	9328	2.06139755	-2.866799476	0.382980508
174067	2/10/2019 13:26	B	11873767.68	2450074.322	-73.535	-44.939	2858	2.060058639	-2.866799476	0.394274743
174067	2/10/2019 14:03	B	1549391.624	446209.376	-73.527	-44.933	2214	2.217368672	-2.866799476	0.356669573
174067	2/10/2019 14:14	A	966661	966661	-73.559	-44.944	632	2.268747671	-2.866799476	0.336191944
174067	2/10/2019 14:57	B	4192775.627	1184066.873	-73.555	-44.941	2585	2.322012504	-2.866799476	0.306327694
174067	2/10/2019 15:46	1	107981673.7	4879262.263	-73.529	-44.946	2942	2.213984908	-2.866799476	0.328542669
174067	2/10/2019 15:56	B	2776264.372	134487.6284	-73.527	-44.945	586	2.231125344	-2.866799476	0.32003036
174067	2/10/2019 16:39	A	122317390.3	19979854.69	-73.523	-44.953	2585	2.423884663	-2.866799476	0.256007556
174067	2/10/2019 17:37	B	1948465.819	42748999.18	-73.584	-44.943	3488	2.388242122	-2.802563018	0.086215393
174067	2/10/2019 18:13	B	1935659.755	196370.7451	-73.586	-44.953	2150	2.153565443	-2.759738712	0.014954165
174067	2/11/2019 0:42	B	143386689.4	1254233.12	-73.616	-45.053	23375	1.235328446	-1.17651766	-0.293714818
174067	2/11/2019 1:45	1	413759.1109	61441.38912	-73.631	-45.033	3765	1.080134373	-1.336562343	-0.294602791
174067	2/11/2019 2:27	A	196587.7307	111682.7693	-73.634	-45.037	2495	0.63202834	-1.300927025	-0.324661162
174067	2/11/2019 4:02	B	7787841.902	11873608.1	-73.65	-45.033	5750	0.115721193	-1.267323451	-0.353678309

174067	2/11/2019 4:19	B	975385	975385	-73.65	-45.033	1010	0.000974949	-1.276392692	-0.358788653
174067	2/11/2019 6:32	B	4134604.33	424258.1702	-73.672	-45.008	7957	-0.017428203	-1.486732247	-0.353720614
174067	2/11/2019 7:35	B	6349591.945	641800.5553	-73.649	-45.006	3762	0.130407765	-1.671687761	-0.347334675
174067	2/11/2019 11:38	A	78230734.23	383174.2704	-73.608	-44.949	14631	1.706713808	-2.545617183	-0.237730433
174067	2/11/2019 12:10	B	2616693.018	104317.4825	-73.608	-44.945	1898	1.64972686	-2.44490076	-0.231654384
174067	2/11/2019 13:13	A	1138408.97	1615153.53	-73.653	-44.943	3759	0.748666185	-1.995600108	-0.262890016
174067	2/11/2019 13:53	B	6165927.326	208673.1736	-73.614	-44.934	2437	1.162408254	-2.09076561	-0.23064643
174067	2/11/2019 14:32	B	67266190.15	7174834.854	-73.603	-44.944	2353	1.583043513	-2.199088307	-0.213886884
174067	2/11/2019 15:28	B	5966790.735	418831.765	-73.6	-44.942	3333	1.18964355	-2.318862724	-0.257322538
174067	2/11/2019 15:36	B	6755405.543	661684.4567	-73.602	-44.944	485	1.138246604	-2.269448314	-0.258970423
174067	2/11/2019 16:13	B	1039825.392	254567.6084	-73.66	-44.945	2194	0.564175559	-1.935772486	-0.280303332
174067	2/11/2019 17:13	B	2088655.174	461090.8259	-73.663	-44.948	3591	0.568922817	-1.702235466	-0.273180167
174067	2/11/2019 17:15	B	2095788.114	512564.3861	-73.66	-44.942	136	0.568922817	-1.695205387	-0.273180167
174067	2/11/2019 17:53	B	2919202.686	530865.8136	-73.665	-44.95	2269	0.541642139	-1.758660303	-0.276243071
174067	2/11/2019 23:52	B	13246999.18	1421033.823	-73.645	-44.936	21547	3.478631776	-2.344437445	0.140985866
174067	2/12/2019 0:16	A	42597.3442	361127.1558	-73.58	-44.886	1437	3.759682455	-2.286472501	0.261976695

174067	2/12/2019 1:34	A	1534767.849	1431357.151	-73.572	-44.893	4693	3.419018715	-2.465615371	0.324593692
174067	2/12/2019 1:51	B	403232.3389	152792.1611	-73.572	-44.893	1036	3.651218429	-2.514195752	0.31393502
174067	2/12/2019 3:16	B	2124796.701	430041.7992	-73.568	-44.894	5083	3.689975269	-2.574036257	0.34351075
174067	2/12/2019 3:41	B	2413689.28	560872.7197	-73.569	-44.894	1480	3.667549627	-2.492479799	0.327720854
174067	2/12/2019 4:31	A	152734608.9	14322999.57	-73.607	-44.881	3046	3.759682455	-2.383633264	0.261976695
174067	2/12/2019 5:31	2	442601.2696	24661.23037	-73.576	-44.894	3586	3.599175515	-2.492479799	0.325167363
174067	2/12/2019 5:35	A	9545181.484	364047.0164	-73.586	-44.893	222	3.662226863	-2.526354501	0.33593873
174067	2/12/2019 6:09	3	496651.7801	16104.71994	-73.544	-44.884	2059	3.15913451	-2.703818583	0.447306107
174067	2/12/2019 7:11	0	28559890.09	16256033.91	-73.548	-44.868	3724	3.001658282	-2.626219195	0.405982923
174067	2/12/2019 7:12	B	9799454.26	5207770.74	-73.548	-44.868	23	3.001658282	-2.626219195	0.405982923
174067	2/12/2019 7:50	B	2521358.302	562961.698	-73.58	-44.895	2319	3.727691919	-2.528995762	0.308464544
174067	2/12/2019 11:25	1	510399.5706	86662.9294	-73.612	-44.993	12900	5.302909245	-2.377330953	-0.067172578
174067	2/12/2019 13:06	B	70670846.29	14907406.71	-73.586	-44.999	6069	4.74753171	-1.747785913	0.348094209
174067	2/12/2019 13:20	B	248254899.6	328451410.9	-73.563	-45.008	831	4.179777439	-1.606889483	0.387316669
174067	2/12/2019 13:41	B	1469682.037	1380834.463	-73.57	-45.016	1254	4.002757069	-1.39086865	0.342254563
174067	2/12/2019 14:05	A	8773564.039	98059.96093	-73.632	-45.056	1440	3.677400395	-1.105816273	0.25359355

174067	2/12/2019 15:04	A	74701.25741	657251.7426	-73.609	-45.085	3545	2.532981729	-0.773828531	0.03411949
174067	2/12/2019 15:21	B	1064455.589	555948.4112	-73.609	-45.085	1034	1.911060026	-0.745521925	-0.065000626
174067	2/12/2019 15:54	A	86633.73058	2036695.269	-73.648	-45.068	1933	1.266121203	-0.764237421	-0.181146472
174067	2/12/2019 16:27	B	3182007.255	7516031.245	-73.63	-45.076	2004	1.371889163	-0.742007858	-0.160034049
174067	2/12/2019 16:56	A	356998.514	977723.486	-73.625	-45.095	1713	1.834127452	-0.745754322	-0.07970582
174067	2/12/2019 17:03	B	1143642.39	1266927.61	-73.625	-45.08	461	1.834127452	-0.746856936	-0.07970582
174067	2/12/2019 17:34	B	13388925.12	34850659.88	-73.604	-45.076	1862	2.512290262	-0.790421891	0.011735195
174067	2/12/2019 23:44	B	552582.7585	13656333.24	-73.512	-45.011	22157	1.367318924	-2.866799476	0.685417474
174067	2/13/2019 1:24	A	15222927.71	582457.2887	-73.478	-44.796	6034	0.755163917	-1.865747098	0.776853045
174067	2/13/2019 3:03	B	7920375.413	1508139.087	-73.439	-44.725	5958	-0.168865135	-0.898803886	0.849611876
174067	2/13/2019 3:38	A	596660.9028	53151.59717	-73.519	-44.767	2051	0.097611635	-1.052394411	0.810431023
174067	2/13/2019 5:12	3	82393.68608	2616.313921	-73.5	-44.768	5675	-0.004346867	-1.166181184	0.839422291
174067	2/13/2019 5:21	2	198691.5382	42485.46184	-73.481	-44.767	501	-0.10347604	-1.115015427	0.85553673
174067	2/13/2019 7:31	B	30897599.94	5087712.557	-73.512	-44.767	7827	-0.352980138	-1.376223518	0.901941314
174067	2/13/2019 11:12	A	1651570.677	2868302.323	-73.344	-44.895	13232	-0.63776866	-2.368970724	0.981732472
174067	2/13/2019 13:02	A	168677000.5	13951612.03	-73.35	-44.867	6631	-0.616353315	-2.321454646	0.979240817

174067	2/13/2019 13:34	B	31791370.29	13495482.21	-73.374	-44.877	1901	-0.602573465	-2.329527486	0.979229393
174067	2/13/2019 14:40	A	461973.5272	34838.97277	-73.355	-44.877	4004	-0.600474836	-2.287911365	0.978597445
174067	2/13/2019 15:12	0	26105006.31	2023458.691	-73.314	-44.883	1896	-0.625339498	-2.276723319	0.980520809
174067	2/13/2019 15:32	B	62781604.55	12283223.95	-73.314	-44.882	1203	-0.65994432	-2.234476889	0.985664166
174067	2/13/2019 16:36	0	896836943.1	17719481.38	-73.352	-44.894	3827	-0.690054031	-2.158024714	0.982782951
174067	2/13/2019 16:55	B	16902629.56	1217663.442	-73.358	-44.896	1162	-0.693015054	-2.03851846	0.981158037
174067	2/13/2019 17:13	A	107539.6729	49110.32714	-73.342	-44.913	1057	-0.694169146	-1.96855026	0.978663101
174067	2/13/2019 18:16	B	1460923.835	248064.6649	-73.367	-44.919	3794	-0.635767876	-1.986662589	0.957343726
174067	2/13/2019 23:34	B	9309863.455	1338333.045	-73.477	-44.936	19080	0.113552156	-2.158930019	0.823404854
174067	2/14/2019 0:50	B	241791214.9	25955205.59	-73.458	-44.901	4525	0.342034783	-2.309134711	1.781983471
174067	2/14/2019 1:10	0	982255619.3	44383465.67	-73.455	-44.92	1249	0.360885692	-2.336455981	1.784794151
174067	2/14/2019 2:51	B	454267763.7	42510037.31	-73.47	-44.938	6055	0.451554339	-2.620791123	1.817990249
174067	2/14/2019 3:27	A	23576828.66	5146597.34	-73.461	-44.893	2120	0.490070903	-2.828472687	1.830990853
174067	2/14/2019 4:34	B	23346.85085	12330454.15	-73.451	-44.886	4065	0.616789341	-2.866799476	1.806765694
174067	2/14/2019 5:06	1	6864687.76	317848.7404	-73.464	-44.865	1908	0.699458731	-2.866799476	1.766994434
174067	2/14/2019 5:26	1	20272071.47	401891.0311	-73.506	-44.869	1188	0.712502803	-2.866799476	1.724101077

174067	2/14/2019 6:02	B	2143725.538	191198.9624	-73.504	-44.861	2191	0.716403533	-2.866799476	1.657854294
174067	2/14/2019 6:27	2	108779.2795	20987.22048	-73.502	-44.853	1463	0.92792384	-2.793584326	1.641779013
174067	2/14/2019 7:07	A	3338970.092	251166.9076	-73.496	-44.829	2408	1.657866446	-2.429474452	1.66363921
174067	2/14/2019 11:03	B	3823444.805	1169693.195	-73.496	-44.739	14187	2.991449457	-0.815172708	1.906552885
174067	2/14/2019 12:36	B	4819932.84	3050939.66	-73.495	-44.714	5547	2.612468748	-0.734761243	1.865970256
174067	2/14/2019 14:11	B	468369405.2	25107855.8	-73.587	-44.722	5721	3.343175928	-0.853974713	1.779609375
174067	2/14/2019 14:59	B	9647252.371	601722.1289	-73.522	-44.761	2839	3.780425384	-1.028734715	1.672669213
174067	2/14/2019 15:12	B	85360354.91	5308743.586	-73.518	-44.768	781	3.794582679	-1.05534049	1.643757745
174067	2/14/2019 15:52	B	24439470.22	2837774.777	-73.505	-44.777	2443	3.601441044	-1.447417928	1.604872885
174067	2/14/2019 16:14	A	474035.7151	542590.2849	-73.519	-44.79	1312	3.520300661	-1.558124695	1.608667839
174067	2/14/2019 16:42	B	681877.0331	158254.9669	-73.516	-44.79	1657	3.234744446	-1.658300884	1.608704768
174067	2/14/2019 17:58	B	70285914.29	4112828.214	-73.41	-44.817	4566	2.091153697	-2.178285722	1.55403338
174067	2/15/2019 0:21	B	34102018.34	4448574436	-73.73	-44.847	22974	0.280259697	-1.601557381	1.561280056
174067	2/15/2019 0:57	0	439046463	11022250.99	-73.721	-44.945	2157	0.379901649	-1.69127449	1.58295263
174067	2/15/2019 2:43	B	53153830.67	11736419.33	-73.742	-45.002	6388	0.63194133	-1.980690943	1.637114372
174067	2/15/2019 4:07	2	200685.2247	51099.7753	-73.62	-44.929	5029	0.249974913	-2.261523036	1.561337661

174067	2/15/2019 4:33	B	5111951.406	1140373.594	-73.62	-44.929	1585	0.096274598	-2.231141854	1.545060799
174067	2/15/2019 5:08	1	248718.7547	72766.24534	-73.601	-44.916	2056	-0.135201034	-2.249745101	1.526860462
174067	2/15/2019 6:10	A	1329087.268	90304.73182	-73.572	-44.911	3764	-0.266177537	-2.587177819	1.528641802
174067	2/15/2019 12:29	B	1024456.674	4559004.326	-73.53	-44.863	22698	0.500597073	-2.866799476	1.574185251
174067	2/15/2019 13:46	0	212015518.6	34745705.43	-73.508	-44.842	4653	0.223654037	-2.648817684	1.551580348
174067	2/15/2019 14:11	B	11480680.89	153617.1084	-73.524	-44.849	1499	0.035180546	-2.48629457	1.536849751
174067	2/15/2019 14:46	B	1082193.399	2962091.101	-73.556	-44.855	2089	-0.085746972	-2.200200596	1.529785416
174067	2/15/2019 14:54	B	1767744.896	310305.6037	-73.565	-44.833	489	-0.090064414	-2.200200596	1.529043458
174067	2/15/2019 15:27	B	2190074.315	1477445.685	-73.559	-44.855	1965	-0.178668613	-2.293999557	1.518524297
174067	2/15/2019 15:53	B	4061513.154	1113787.846	-73.56	-44.857	1550	-0.258575944	-2.414068903	1.51126967
174067	2/15/2019 16:32	B	21763034	7255178	-73.537	-44.864	2365	-0.113437798	-2.602181616	1.521925196
174067	2/15/2019 17:37	B	2072872.986	291103.0139	-73.538	-44.861	3896	0.11879783	-2.730774408	1.539788703
174067	2/15/2019 18:11	B	4527135.438	2581354.562	-73.541	-44.863	2031	0.20483471	-2.752021957	1.546330617
174067	2/15/2019 23:53	A	128093107.7	1951232.309	-73.584	-44.959	20513	0.54879737	-2.82397517	1.627871496
174067	2/16/2019 0:50	A	549827335.5	183281325.5	-73.628	-44.93	3429	-0.083903968	-2.802563018	1.607659201
174067	2/16/2019 2:01	B	57030765.72	8073407.28	-73.64	-44.907	4284	-0.28962649	-2.60704872	1.585801226

174067	2/16/2019 3:08	B	39042696.3	6716998.199	-73.629	-44.908	3968	-0.070082274	-2.627923992	1.590187823
174067	2/16/2019 3:13	B	34815068	3508901.999	-73.633	-44.907	308	-0.001041727	-2.671355899	1.592245779
174067	2/16/2019 4:09	A	652874.1562	109318.3438	-73.53	-44.871	3380	0.356663954	-2.866799476	1.588079702
174067	2/16/2019 4:40	B	769456.8273	162199.6727	-73.536	-44.866	1887	0.431093016	-2.866799476	1.587814174
174067	2/16/2019 4:43	B	1658110.391	477962.109	-73.53	-44.866	148	0.376714633	-2.866799476	1.583584276
174067	2/16/2019 5:20	B	2732372.924	274549.0763	-73.535	-44.865	2224	0.28369548	-2.866799476	1.578182963
174067	2/16/2019 7:29	B	16643332.09	695424.4127	-73.538	-44.838	7743	0.167079902	-2.52753717	1.586564225
174067	2/16/2019 10:38	A	30571.625	70398.875	-73.51	-44.83	11315	0.756548201	-2.421413846	1.649577721
174067	2/16/2019 12:20	2	942300.1703	16389.82973	-73.503	-44.861	6125	1.338160383	-2.866799476	1.693448548
174067	2/16/2019 13:24	A	1180681.749	9911068.751	-73.503	-44.856	3851	1.635808163	-2.866799476	1.737266138
174067	2/16/2019 13:43	B	7740820.041	1700872.459	-73.483	-44.891	1158	1.635976572	-2.866799476	1.742698856
174067	2/16/2019 14:32	B	69377994.45	22031559.55	-73.519	-44.866	2945	1.386604612	-2.866799476	1.697634018
174067	2/16/2019 14:37	A	6502785.25	6502785.25	-73.501	-44.867	296	1.350552003	-2.866799476	1.68949957
174067	2/16/2019 15:05	B	634116.7936	157741.2064	-73.5	-44.869	1663	0.972584056	-2.866799476	1.640967881
174067	2/16/2019 15:18	A	27220.70192	49568.29808	-73.524	-44.866	775	0.699851766	-2.866799476	1.613741108
174067	2/16/2019 16:10	1	51983768.18	30798.31829	-73.558	-44.875	3135	0.630505272	-2.866799476	1.608994568

174067	2/16/2019 16:43	A	2358599912	59173081.59	-73.532	-44.862	1995	0.552269821	-2.866799476	1.601362097
174067	2/16/2019 17:13	2	133396.6745	18984.32546	-73.519	-44.846	1774	0.552482976	-2.680540568	1.622670589
174067	2/16/2019 23:21	B	78313814.82	19722613.68	-73.643	-44.809	22069	-0.109725043	-2.034924208	1.559912222
174067	2/17/2019 0:32	B	1511737611	397465781.9	-73.619	-44.824	4257	0.39585039	-2.286275853	1.656806864
174067	2/17/2019 1:03	B	1003139813	288173455	-73.606	-44.828	1863	0.409847715	-2.398237918	1.665809329
174067	2/17/2019 2:34	B	222368469.4	6990829.098	-73.573	-44.85	5489	0.43459631	-2.7829823	1.677096211
174067	2/17/2019 3:18	B	65176950.34	111422.1609	-73.541	-44.864	2605	0.660229727	-2.866799476	1.7143568
174067	2/17/2019 3:50	B	102230225.6	4083794.362	-73.585	-44.848	1946	0.880254684	-2.866799476	1.752835421
174067	2/17/2019 4:24	B	263154543.7	138990058.8	-73.471	-44.877	2055	1.236085691	-2.866799476	1.805991362
174067	2/17/2019 4:34	A	555386.3185	41436.18145	-73.487	-44.871	570	1.390126778	-2.866799476	1.82803064
174067	2/17/2019 5:28	1	68986562.52	2620635.982	-73.474	-44.867	3251	1.689150352	-2.866799476	1.898218102
174067	2/17/2019 6:09	A	5874760339	221987033	-73.486	-44.867	2461	1.88486715	-2.866799476	1.945896173
174067	2/17/2019 6:17	B	65919849.83	12305043.17	-73.451	-44.87	475	1.88486715	-2.866799476	1.945896173
174067	2/17/2019 7:07	B	460204546.7	38141925.31	-73.464	-44.847	3029	2.118740196	-2.866799476	1.994501807
174067	2/17/2019 10:26	A	15382014.29	1268307.706	-73.417	-44.837	11943	2.617272925	-2.581576668	2.092060493
174067	2/17/2019 12:07	B	349615922.7	2677103.258	-73.398	-44.85	6005	1.704649227	-2.76601955	1.918613386

174067	2/17/2019 13:09	B	5444885.971	6981124.029	-73.5	-44.856	3728	1.554727297	-2.866799476	1.872237604
174067	2/17/2019 14:39	B	2677594.657	4060386.343	-73.449	-44.904	5432	2.52366921	-1.999586238	2.17436135
174067	2/17/2019 15:12	A	338800.5343	9085131.966	-73.426	-44.989	1944	2.468401982	-1.491879074	2.240520192
174067	2/17/2019 15:52	B	22325870.64	502821.8632	-73.429	-44.991	2436	2.144589634	-1.168971297	2.199550252
174067	2/17/2019 16:05	B	19102380.5	2738	-73.423	-45.003	775	1.986875605	-1.125296471	2.191380623
174067	2/17/2019 16:24	B	496008.4575	607184.0425	-73.427	-45.004	1102	1.898038044	-1.098258383	2.196592805
174067	2/17/2019 16:53	A	193503.4791	1739857.521	-73.421	-44.993	1790	1.79619594	-1.054522683	2.201824154
174067	2/17/2019 17:28	A	8800297.91	400504.59	-73.412	-45.029	2098	1.518218792	-0.962893695	2.209683017
174067	2/18/2019 0:29	B	1023520831	431692301.9	-73.352	-44.897	25219	0.274401229	-1.745046857	2.32485495
174067	2/18/2019 2:07	B	27966965.73	519276.7721	-73.352	-44.908	5921	0.160910176	-2.451899358	2.364862113
174067	2/18/2019 2:54	1	1253123.964	773194.5362	-73.395	-44.899	2778	0.057504077	-2.485813014	2.398794689
174067	2/18/2019 4:20	2	236827.5321	21644.46794	-73.338	-44.924	5182	-0.071521033	-1.840718542	2.450765278
174067	2/18/2019 4:34	2	92220.19714	20892.30286	-73.372	-44.927	862	0.041252021	-1.82213242	2.416831845
174067	2/18/2019 5:11	B	2254114.615	398310.3852	-73.377	-44.936	2192	0.183704379	-1.66948283	2.372417552
174067	2/18/2019 5:43	B	2825394.77	699527.2301	-73.377	-44.94	1937	0.214048096	-1.561615659	2.365729057
174067	2/18/2019 6:23	B	3205560.412	940518.0881	-73.38	-44.946	2391	0.266760277	-1.54635773	2.340648744

174067	2/18/2019 7:28	A	86368.34513	181354.1549	-73.416	-44.934	3895	0.330330428	-1.733097915	2.2833219
174067	2/18/2019 10:16	B	22958474.25	1670400.254	-73.42	-44.901	10111	0.29852219	-2.606365406	2.267477588
174067	2/18/2019 11:49	B	10461532.31	725912.6908	-73.424	-44.906	5524	0.324581126	-2.472611641	2.241064417
174067	2/18/2019 12:32	B	113028.2319	37067663.77	-73.444	-44.902	2592	0.351517947	-2.058004775	2.215216988
174067	2/18/2019 13:32	B	8726167.366	1486449.634	-73.449	-44.951	3645	0.359404149	-1.877594666	2.219865398
174067	2/18/2019 14:13	B	88146636.87	1555948.125	-73.365	-44.888	2450	0.307572396	-2.532449455	2.257828019
174067	2/18/2019 14:54	B	133928316.8	4216771.687	-73.362	-44.882	2468	0.260190268	-2.866799476	2.286343521
174067	2/18/2019 15:28	0	127915681.6	46247404.95	-73.412	-44.895	1990	0.24362482	-2.866799476	2.295053981
174067	2/18/2019 15:53	B	11610756.6	3078765.4	-73.417	-44.894	1511	0.239313607	-2.866799476	2.293536771
174067	2/18/2019 16:37	A	2699523393	40769256.83	-73.37	-44.887	2644	0.222801736	-2.866799476	2.290391279
174067	2/18/2019 17:11	3	611987.2144	28843.28563	-73.428	-44.866	2022	0.227237688	-2.866799476	2.274040992
174067	2/18/2019 17:30	B	616574.7534	136827.7466	-73.437	-44.863	1159	0.236373983	-2.866799476	2.258792823
174067	2/19/2019 0:08	A	395768.6926	487508.3074	-73.462	-44.839	23906	-0.021097138	-2.593394915	1.286723562
174067	2/19/2019 1:52	B	1716405.55	551550.4498	-73.459	-44.84	6218	-0.037904219	-2.633855256	1.263597973
174067	2/19/2019 2:27	A	117511.4678	3588587.032	-73.487	-44.842	2094	-0.046229853	-2.634946114	1.250408276
174067	2/19/2019 4:07	2	171349.0687	20444.93128	-73.422	-44.843	5985	0.007077075	-2.640255161	1.358696416

174067	2/19/2019 4:11	2	130969.8371	23242.66295	-73.423	-44.845	274	0.007077075	-2.655878907	1.358696416
174067	2/19/2019 5:24	3	85047.9529	4776.547099	-73.494	-44.856	4346	-0.063913576	-2.816246158	1.256677313
174067	2/19/2019 7:04	B	63685862.3	4577267.703	-73.554	-44.892	6018	-0.101400125	-2.866799476	1.258417429
174067	2/19/2019 11:45	1	38203741.57	7587242.431	-73.384	-44.907	16870	-0.078155045	-2.694328924	1.35278694
174067	2/19/2019 13:58	B	1603916.555	198907.4445	-73.514	-44.898	8005	-0.105483179	-2.866799476	1.281967623
174067	2/19/2019 14:36	B	6101658.148	1306738.852	-73.525	-44.897	2249	-0.1138297	-2.866799476	1.256579552
174067	2/19/2019 15:10	B	80667596.21	29132087.79	-73.536	-44.893	2053	-0.114612802	-2.866799476	1.239618211
174067	2/19/2019 15:24	B	10711587.43	2173105.573	-73.543	-44.891	814	-0.117221193	-2.866799476	1.226803784
174067	2/19/2019 15:42	B	46574542.18	1764569.824	-73.533	-44.879	1071	-0.117301505	-2.824865222	1.224827939
174067	2/19/2019 16:12	B	6580484.932	213171.5683	-73.539	-44.878	1813	-0.119831567	-2.767287266	1.211996362
174067	2/19/2019 16:53	B	33406672.08	1105680.925	-73.554	-44.895	2464	-0.127916461	-2.776718486	1.217575928
174067	2/19/2019 17:57	B	40887472.07	2103543.933	-73.576	-44.9	3852	-0.135520357	-2.714787805	1.221554803
174067	2/19/2019 18:32	B	51724620.5	2834580.5	-73.584	-44.903	2124	-0.137017985	-2.714787805	1.22465616
174067	2/19/2019 23:28	B	17201410.81	7678599.189	-73.555	-44.904	17739	-0.092884086	-2.866799476	1.274610247
174067	2/20/2019 1:03	B	2904824308	1048742566	-73.435	-44.828	5722	0.11485334	-2.727852852	1.217906287
174067	2/20/2019 1:40	2	526614.2254	322574.2746	-73.429	-44.835	2209	0.136843577	-2.526514808	1.229721943

174067	2/20/2019 2:02	B	964464.1772	217660.8228	-73.429	-44.836	1299	0.145498494	-2.498983878	1.236862156
174067	2/20/2019 3:23	B	3019010.431	845180.0686	-73.417	-44.832	4842	0.152915297	-2.417397787	1.264669441
174067	2/20/2019 4:03	B	15696445.43	1016017.066	-73.409	-44.852	2430	0.139218458	-2.661815912	1.335985161
174067	2/20/2019 4:29	B	145765980.5	715411.9905	-73.444	-44.881	1568	0.119270297	-2.708881225	1.391687955
174067	2/20/2019 5:04	B	7733815.5	38880018.5	-73.351	-44.894	2057	0.088348539	-2.543695623	1.458609808
174067	2/20/2019 5:38	A	299535252.3	11333487.71	-73.347	-44.924	2068	0.059984787	-2.10275674	1.506918535
174067	2/20/2019 6:06	B	39976032.83	3686112.165	-73.346	-44.924	1665	0.042336218	-1.921436585	1.532093688
174067	2/20/2019 6:50	1	3984460.015	398540.4846	-73.337	-44.921	2628	0.030494211	-1.718711646	1.564902848
174067	2/20/2019 7:47	B	25820262.94	6352403.556	-73.311	-44.931	3441	0.014460869	-1.477225989	1.607581523
174067	2/20/2019 9:51	B	128630470.7	10979365.77	-73.262	-44.945	7434	-0.011207863	-1.133045704	1.690743331
174067	2/20/2019 11:32	A	2463013.535	93438.96459	-73.258	-44.944	6066	-0.016299117	-1.087811744	1.706919662
174067	2/20/2019 13:14	B	191014.8205	1063389.68	-73.318	-44.935	6144	0.022131087	-1.517389287	1.597309983
174067	2/20/2019 13:25	A	6652157.728	6206298.772	-73.309	-44.909	669	0.031000411	-1.552264379	1.590088899
174067	2/20/2019 13:51	B	8451359.872	1132497.128	-73.298	-44.891	1554	0.062646367	-1.643810489	1.571739695
174067	2/20/2019 15:00	B	17990210.33	3777204.174	-73.287	-44.904	4093	0.04859039	-1.51544868	1.598494789
174067	2/20/2019 15:30	A	1139652664	150991558.6	-73.273	-44.924	1843	0.027836029	-1.449224488	1.617719832

174067	2/20/2019 16:25	B	9116323.404	745223.0961	-73.292	-44.931	3254	0.016768484	-1.416446108	1.621030316
174067	2/20/2019 17:07	B	23818403.78	831372.7244	-73.327	-44.902	2536	0.049931225	-1.601736974	1.587671282
174067	2/20/2019 17:35	B	22304601.51	1366123.492	-73.322	-44.902	1683	0.068440594	-1.674625291	1.566036163
174067	2/20/2019 23:47	B	44313327.79	4924714.208	-73.387	-44.899	22301	0.084845284	-2.340624832	1.494495033
174067	2/21/2019 0:35	B	77850448.96	4842239.044	-73.342	-44.898	2889	-0.700560082	-2.349114361	-0.119201334
174067	2/21/2019 1:33	B	28054073.28	610774.7249	-73.337	-44.873	3499	-0.670682244	-2.052793678	-0.131870347
174067	2/21/2019 3:09	B	11397364.24	1636238.259	-73.325	-44.87	5744	-0.66601767	-1.970328384	-0.133765347
174067	2/21/2019 4:07	B	15534738	1627208	-73.343	-44.884	3500	-0.624506103	-2.343194475	-0.125542801
174067	2/21/2019 4:44	B	22548035.5	34067492.5	-73.367	-44.889	2206	-0.647812629	-2.466435402	-0.117904705
174067	2/21/2019 5:25	B	2140914.038	1807327.962	-73.364	-44.896	2488	-0.719276963	-2.345661991	-0.108237214
174067	2/21/2019 6:27	B	15005468.61	221679.8855	-73.361	-44.935	3730	-0.857955863	-1.717173742	-0.102046541
174067	2/21/2019 7:24	B	38954232.96	33875479.54	-73.329	-44.937	3369	-0.864575122	-1.479873498	-0.103784307
174067	2/21/2019 11:22	B	1174363420	211596805.3	-73.287	-44.956	14285	-0.895159577	-1.459167905	-0.104436123
174067	2/21/2019 12:45	B	26345493.81	3358420.69	-73.32	-44.942	5006	-0.891606263	-1.545135733	-0.103734334
174067	2/21/2019 14:41	A	122639524.7	1850463.771	-73.376	-44.916	6927	-0.824883342	-1.950102035	-0.099569775
174067	2/21/2019 15:20	A	150463.4137	184229.0863	-73.375	-44.928	2334	-0.811632363	-1.80609033	-0.09988636

174067	2/21/2019 15:36	B	497144.4209	180065.5791	-73.376	-44.928	990	-0.776621207	-1.799821259	-0.100366673
174067	2/21/2019 16:00	B	1060528.314	326520.6859	-73.38	-44.93	1424	-0.744294932	-1.853757591	-0.100522341
174067	2/21/2019 16:41	B	2221716.938	872388.0623	-73.403	-44.916	2476	-0.617028919	-2.203410338	-0.100421878
174067	2/21/2019 17:14	B	58244585.97	2663532.527	-73.409	-44.885	1954	-0.514106361	-2.600259198	-0.106290475
174067	2/21/2019 17:47	B	12412262.02	3244414.978	-73.357	-44.897	2008	-0.52413227	-2.733801176	-0.111268339
174067	2/22/2019 0:01	B	481854962.4	96003380.09	-73.332	-44.957	22427	0.587154503	-1.458404216	-0.063742095
174067	2/22/2019 1:20	B	1166140972	123115569.1	-73.34	-44.937	4727	0.631394015	-1.283898325	-0.098680742
174067	2/22/2019 2:56	B	84136719.67	79410.32684	-73.375	-44.978	5811	0.576246405	-1.077259552	-0.163329365
174067	2/22/2019 3:37	B	12858729.24	184463.2633	-73.375	-44.981	2411	0.548876462	-1.065290046	-0.179618386
174067	2/22/2019 3:49	B	21865976.29	467321.7139	-73.375	-44.981	740	0.606922784	-1.122529267	-0.184052063
174067	2/22/2019 4:22	B	342283.4407	12430118.56	-73.405	-44.98	1988	0.761332257	-1.223913314	-0.161228564
174067	2/22/2019 4:34	B	7032692.204	14335276.3	-73.41	-44.975	726	0.84931471	-1.267592227	-0.148373334
174067	2/22/2019 5:12	A	1247937.105	4392.894581	-73.41	-44.958	2284	0.971848556	-1.394001968	-0.119493269
174067	2/22/2019 5:23	A	1313842.166	3521.834351	-73.405	-44.958	630	0.953961806	-1.401634085	-0.127306781
174067	2/22/2019 6:04	A	25318910.84	1144214.164	-73.42	-44.946	2489	1.264994866	-1.496810039	-0.12568893
174067	2/22/2019 7:07	A	145257.2938	1130003.206	-73.424	-44.962	3791	1.224036682	-1.472481684	-0.140537263

174067	2/22/2019 7:44	A	356398.3454	19018.15456	-73.403	-44.954	2196	0.988349326	-1.440284642	-0.100312292
174067	2/22/2019 11:05	B	307229388.9	102412037.6	-73.407	-44.947	12086	0.524696585	-1.04040612	-0.172281325
174067	2/22/2019 12:45	B	814238890.2	994180.3482	-73.367	-45	5968	0.246512564	-0.925549822	-0.224814052
174067	2/22/2019 13:53	B	9107261.694	237416.8056	-73.367	-45.001	4091	0.234053407	-0.940814259	-0.232616228
174067	2/22/2019 14:12	0	309371881.8	37041354.22	-73.426	-45.013	1149	0.281556936	-0.981685817	-0.241096937
174067	2/22/2019 15:10	A	6244175.821	5824698.179	-73.414	-44.999	3452	0.542709127	-1.183508167	-0.261113329
174067	2/22/2019 15:27	A	105010673.3	47831269.22	-73.424	-44.988	1057	0.645253333	-1.220728333	-0.267388159
174067	2/22/2019 15:47	A	61994.67914	602318.3209	-73.434	-44.983	1180	0.748836017	-1.25790274	-0.265428944
174067	2/22/2019 16:51	3	69806.20501	104278.795	-73.412	-44.99	3810	0.46593893	-1.104622314	-0.23732056
174067	2/22/2019 17:26	B	3601635.016	62493.48355	-73.404	-44.996	2113	0.393266358	-1.012649247	-0.229779408
174067	2/23/2019 1:15	A	4098501.332	38766.6684	-73.362	-44.953	28160	-0.169792854	-1.3988371	0.336599896
174067	2/23/2019 2:27	A	673760.1165	336382.3835	-73.381	-44.937	4314	-0.158068389	-1.542561585	0.340061202
174067	2/23/2019 2:48	B	25809402	662432.5017	-73.334	-44.955	1236	-0.146749728	-1.47415175	0.335857304
174067	2/23/2019 3:26	B	17134368.27	609948.7276	-73.34	-44.953	2321	-0.169792854	-1.398178769	0.336599896
174067	2/23/2019 5:11	B	65297577.76	3571894.739	-73.35	-44.947	6265	-0.215600296	-1.45073597	0.343455148
174067	2/23/2019 5:38	B	11438664.17	1160918.331	-73.37	-44.948	1644	-0.23977732	-1.45073597	0.34586408

174067	2/23/2019 6:50	B	13076040.94	1326047.062	-73.377	-44.949	4335	-0.17286232	-1.446761952	0.338050549
174067	2/23/2019 7:20	B	4033345.85	1790375.15	-73.372	-44.95	1768	-0.169792854	-1.419929025	0.336599896
174067	2/23/2019 12:36	B	29586520.35	435852.6504	-73.392	-45.047	18993	-0.105671571	-0.756566987	0.170735067
174067	2/23/2019 13:48	A	2907024.125	970416.375	-73.393	-45.078	4293	-0.22018619	-0.616396471	0.161178949
174067	2/23/2019 14:49	B	1474566.92	306138.0801	-73.401	-45.088	3680	-0.21436267	-0.599755452	0.15619824
174067	2/23/2019 14:58	A	25236356.52	2360908.482	-73.419	-45.071	549	-0.176679485	-0.621432398	0.152615805
174067	2/23/2019 15:28	A	86799872.85	758277.6476	-73.389	-45.05	1784	-0.112539299	-0.712237168	0.157694583
174067	2/23/2019 16:36	B	84223403.99	8996942.514	-73.398	-45.059	4083	-0.197069551	-0.657534712	0.166132962
174067	2/23/2019 17:07	A	1163798.408	303473.5921	-73.389	-45.08	1819	-0.260911491	-0.601849519	0.170003056
174067	2/24/2019 0:52	A	1448004.476	1352005.524	-73.438	-45.028	27934	0.567577352	-1.084447443	0.369717974
174067	2/24/2019 2:17	B	35135047.57	91080.92504	-73.466	-44.981	5115	-0.167449792	-1.230370547	0.441099066
174067	2/24/2019 2:33	1	3514889.367	700144.6326	-73.412	-44.988	916	-0.167449792	-1.166651776	0.441099066
174067	2/24/2019 3:12	B	1333021.349	230945.1509	-73.416	-44.987	2344	-0.31195626	-1.063526725	0.463636706
174067	2/24/2019 3:40	A	116664.0246	4096.975366	-73.39	-44.972	1727	-0.436598984	-1.17178215	0.474266822
174067	2/24/2019 5:20	B	1789202.53	438539.9696	-73.407	-44.97	5952	-0.390871365	-1.251574564	0.471432147
174067	2/24/2019 6:26	A	37527.88362	4334.616383	-73.381	-44.94	3989	0.323562529	-1.56034602	0.520224337

174067	2/24/2019 7:03	B	3223541.804	229435.196	-73.412	-44.942	2230	0.209914997	-1.553775631	0.503850759
174067	2/24/2019 12:27	B	76707629.56	6986011.439	-73.347	-44.941	19405	0.234095951	-1.654505674	0.622509836
174067	2/24/2019 12:50	B	82429189.24	6478519.262	-73.322	-44.901	1408	0.100016208	-1.613775467	0.630090376
174067	2/24/2019 13:22	B	38004030.74	641797.2607	-73.347	-44.938	1884	-0.044704703	-1.542638318	0.634663237
174067	2/24/2019 14:28	A	5033404672	227411792.3	-73.312	-44.921	3987	-0.326417433	-1.469940301	0.651808212
174067	2/24/2019 15:04	A	773643.1801	586837.3199	-73.309	-44.926	2176	-0.381227111	-1.472928403	0.657079094
174067	2/24/2019 16:08	B	1864552.087	423121.9128	-73.329	-44.932	3809	-0.098459902	-1.54165961	0.642973991
174067	2/24/2019 16:20	B	4756055.845	1220781.155	-73.327	-44.931	702	-0.012099272	-1.548962493	0.636132386
174067	2/24/2019 16:42	B	2758287.221	746033.7788	-73.328	-44.931	1341	0.014395535	-1.575881603	0.635241261
174067	2/24/2019 18:27	B	4556055.125	1613381.375	-73.336	-44.943	6332	0.242732402	-1.474563033	0.60580758
174067	2/25/2019 0:12	B	292686724.2	13229660.27	-73.35	-44.937	20660	1.809862262	-1.52117377	0.548307978
174067	2/25/2019 0:41	A	17382.61359	97834.38641	-73.365	-44.946	1741	1.60657771	-1.483204258	0.538995291
174067	2/25/2019 1:33	B	982238.5329	268415.9671	-73.368	-44.949	3152	1.28474885	-1.439860236	0.514703358
174067	2/25/2019 1:53	B	2955916.766	170621.2339	-73.366	-44.946	1161	1.290552205	-1.47415175	0.508042148
174067	2/25/2019 2:23	A	28774.72012	32166.27988	-73.384	-44.945	1827	0.955651013	-1.494897852	0.477045863
174067	2/25/2019 3:21	B	1108310.119	286774.8811	-73.396	-44.947	3493	0.807684558	-1.504369052	0.466344041

174067	2/25/2019 4:24	1	391953.9004	196776.5996	-73.363	-44.933	3742	2.077757078	-1.70461188	0.587898161
174067	2/25/2019 5:03	B	24435134.68	5534573.816	-73.366	-44.933	2332	2.077757078	-1.70461188	0.587898161
174067	2/25/2019 6:07	1	4543652.145	911332.8552	-73.379	-44.945	3852	0.560576772	-1.459673856	0.461515715
174067	2/25/2019 6:40	A	294452.7093	12761.79069	-73.4	-44.967	2024	-0.131204477	-1.269025625	0.444061612
174067	2/25/2019 10:36	B	3385900.471	1206952.029	-73.452	-45.017	14128	0.47496245	-1.224643167	0.389242688
174067	2/25/2019 12:09	B	17838598.34	3015266.66	-73.474	-45.04	5578	0.755575157	-1.173506777	0.347836949
174067	2/25/2019 13:59	B	443876594.3	52628159.75	-73.437	-45.028	6614	0.578760061	-1.102059523	0.38661019
174067	2/25/2019 14:36	B	140404869.4	5314311.598	-73.441	-45.022	2231	0.408069688	-1.044176494	0.421340161
174067	2/25/2019 14:48	B	8056831.691	728184.8091	-73.441	-45.034	730	0.401363368	-1.01829113	0.42347409
174067	2/25/2019 15:46	B	4540105.469	450686.5306	-73.441	-45.041	3466	0.055910107	-0.863683398	0.509708111
174067	2/25/2019 16:06	B	4561587.519	1015152.481	-73.443	-45.039	1175	-0.089580056	-0.811949467	0.52552049
174067	2/25/2019 16:24	2	72747.10821	47655.39179	-73.369	-45.026	1117	-0.231206508	-0.771795588	0.539222484
174067	2/25/2019 17:31	1	8474769.428	796067.572	-73.38	-45.016	3989	-0.289143342	-0.775265368	0.531196744
174067	2/25/2019 18:02	B	4780605.451	19924.54878	-73.382	-45.021	1871	-0.203068428	-0.812333313	0.525639402
174067	2/25/2019 23:38	B	731658.8598	6156738.14	-73.449	-45.021	20135	0.225262172	-1.359924626	0.424840602
174067	2/26/2019 0:34	B	52116391.16	46869.83993	-73.468	-44.958	3398	-0.059746744	-1.39262301	0.493810319

174067	2/26/2019 1:20	0	6448737019	200503279.6	-73.45	-44.929	2711	0.164582961	-1.527717171	0.573012363
174067	2/26/2019 2:10	A	605007.8401	230452.1599	-73.323	-44.936	3002	-0.168394464	-1.50571189	0.608667461
174067	2/26/2019 2:55	1	1906535.631	750149.369	-73.362	-44.931	2701	0.016995847	-1.724899698	0.602685801
174067	2/26/2019 4:04	B	9026681.031	6758389.469	-73.347	-44.943	4189	0.14687337	-2.052122699	0.597266778
174067	2/26/2019 4:29	A	316010.1782	5919.821789	-73.401	-44.918	1500	0.127654362	-2.129136619	0.597963978
174067	2/26/2019 4:33	B	139654.509	43361.49101	-73.395	-44.92	213	0.127654362	-2.129136619	0.597963978
174067	2/26/2019 5:40	B	1320839.954	331585.0463	-73.374	-44.914	4028	-0.062401854	-2.26684286	0.619429244
174067	2/26/2019 6:08	A	188170.6733	7462.326726	-73.391	-44.903	1660	-0.183619658	-2.600259198	0.633025922
174067	2/26/2019 6:14	1	6612797.975	905496.5246	-73.408	-44.902	402	-0.183619658	-2.600259198	0.633025922
174067	2/26/2019 7:23	B	29829255.53	6655149.47	-73.409	-44.901	4128	-0.19062801	-2.728311498	0.636233273
174067	2/26/2019 10:21	B	64271726.91	39605590.09	-73.406	-44.879	10683	-0.071708457	-2.284755248	0.620283114
174067	2/26/2019 11:59	A	160232985.8	2416862.651	-73.331	-44.93	5857	0.180753149	-1.793371608	0.583942443
174067	2/26/2019 13:23	B	16625576.74	2683985.757	-73.303	-44.939	5073	0.22469784	-1.66627928	0.566324515
174067	2/26/2019 14:17	A	4217580.01	872268.9897	-73.414	-44.929	3228	0.28889851	-1.803554808	0.560254642
174067	2/26/2019 15:09	B	5419949.232	993369.2678	-73.44	-44.928	3115	0.390657351	-2.005388033	0.578023423
174067	2/26/2019 15:27	B	4569789.11	365110.8901	-73.449	-44.922	1061	0.390657351	-2.018233357	0.578023423

174067	2/26/2019 15:58	B	7360005.226	1935227.274	-73.397	-44.936	1892	0.284335314	-1.848721397	0.557890625
174067	2/26/2019 16:09	B	3968954.18	752058.3202	-73.389	-44.931	646	0.278706089	-1.791098949	0.557787558
174067	2/26/2019 17:08	B	2670079.027	3067700.973	-73.391	-44.925	3524	0.227905019	-1.806373881	0.571627691
174067	2/26/2019 17:43	B	1214342.318	724577.6816	-73.394	-44.926	2082	0.1998195	-1.933636662	0.582322578
174067	2/27/2019 0:18	1	1291424.324	80500.67624	-73.458	-44.884	23697	0.430870214	-2.866799476	0.702201296
174067	2/27/2019 0:48	A	21360510.83	1145563.167	-73.476	-44.884	1841	0.451947638	-2.866799476	0.711492708
174067	2/27/2019 2:05	A	17088812.11	437048.8866	-73.487	-44.904	4599	0.364453444	-2.790145897	0.68554663
174067	2/27/2019 2:24	B	7306534.944	213906.0559	-73.492	-44.906	1174	0.361574336	-2.76978479	0.688284315
174067	2/27/2019 4:06	B	6141879.936	361929.0639	-73.51	-44.921	6073	0.767703529	-2.866799476	0.667138987
174067	2/27/2019 4:17	B	4486049.062	478432.9378	-73.519	-44.922	701	0.842979192	-2.866799476	0.660269797
174067	2/27/2019 5:22	1	2287024.783	14025.21664	-73.528	-44.915	3907	0.63444187	-2.866799476	0.705598303
174067	2/27/2019 5:57	B	24721451.53	723672.969	-73.539	-44.902	2048	0.297606219	-2.866799476	0.734402809
174067	2/27/2019 11:51	A	342866682.5	32053174.49	-73.513	-44.963	21277	0.668719764	-2.503505205	0.532997426
174067	2/27/2019 13:34	B	36685059.96	6061310.537	-73.518	-44.969	6196	0.598825688	-1.931836308	0.466121433
174067	2/27/2019 14:39	B	34618190.53	3904437.968	-73.51	-44.984	3880	0.432242662	-1.252218369	0.408136721
174067	2/27/2019 15:08	B	2888614.563	3075639.937	-73.412	-45.006	1717	0.362783299	-1.125296471	0.374170185

174067	2/27/2019 15:26	B	3072633.062	2353669.438	-73.414	-45.008	1105	0.341626463	-1.065487635	0.359599178
174067	2/27/2019 15:44	B	3412004.19	1913500.81	-73.416	-45.01	1067	0.33023944	-1.023102341	0.34693426
174067	2/27/2019 16:46	B	2974447.1	297791.4002	-73.422	-45.02	3703	0.297483983	-0.966960776	0.322841961
174067	2/27/2019 17:24	2	87235.7101	91965.2899	-73.411	-45.008	2272	0.324854444	-1.014821594	0.357158042
174067	2/28/2019 0:17	A	1980333.459	10561.04065	-73.479	-44.836	24792	1.468644177	-2.516702839	0.337418628
174067	2/28/2019 3:38	2	27457188.91	3637789.59	-73.468	-44.844	12094	1.289477156	-2.666292866	0.389224911
174067	2/28/2019 4:05	A	8790238.052	98441.94771	-73.474	-44.847	1575	1.217765571	-2.704997447	0.395237123
174067	2/28/2019 5:06	B	86288293.41	5365535.087	-73.476	-44.839	3665	1.187252704	-2.632764399	0.37841477
174067	2/28/2019 5:20	B	5741068.381	346914.1186	-73.477	-44.838	833	1.20173169	-2.527311859	0.365888477
174067	2/28/2019 6:13	B	3702468.301	2802031.699	-73.475	-44.829	3198	1.082260488	-2.29113012	0.336813505
174067	2/28/2019 6:42	B	918871.1815	190367.3185	-73.461	-44.814	1749	1.106020741	-2.225629164	0.314167694
174067	2/28/2019 7:20	A	1061019.188	4502070.812	-73.485	-44.828	2276	1.589395072	-2.234158323	0.268416255
174067	2/28/2019 11:35	B	51428180.08	8326936.422	-73.536	-44.83	15315	2.183956848	-2.291096175	0.24937308
174067	2/28/2019 13:24	B	647309447.8	489146177.2	-73.48	-44.851	6515	1.411911109	-2.194334334	0.27591074
174067	2/28/2019 14:06	B	1829339919	45891089.78	-73.476	-44.815	2523	1.052177996	-2.063836144	0.310439665
174067	2/28/2019 15:24	B	11579935.74	368180.7558	-73.454	-44.815	4671	0.460922807	-2.223121788	0.372098477

174067	2/28/2019 15:38	B	3253042471	122912428.7	-73.477	-44.842	872	0.23016998	-2.329789167	0.396041167
174067	2/28/2019 15:46	B	74273034.93	2827125.073	-73.461	-44.846	465	0.104478132	-2.436332565	0.410484008
174067	2/28/2019 16:24	A	17303605138	434230783.4	-73.465	-44.855	2274	0.217814921	-2.761857351	0.416691565
174067	2/28/2019 17:03	B	4755824.613	10405015.39	-73.423	-44.868	2350	0.955715415	-2.824389083	0.362423324
174067	2/28/2019 18:06	B	3245517.974	1611370.526	-73.374	-44.895	3765	0.29611024	-2.793901328	0.371349131
174067	3/1/2019 1:24	A	93180800.98	11051633.52	-73.392	-44.907	26313	-0.332793874	-2.110587549	0.09891306
174067	3/1/2019 1:33	B	39679128.48	3503072.018	-73.386	-44.909	533	-0.352898411	-2.03805519	0.101485647
174067	3/1/2019 3:18	B	2800581.247	608763.7533	-73.318	-44.919	6315	-0.308485383	-1.755428378	0.101311973
174067	3/1/2019 5:14	B	336213410.7	414134.3043	-73.349	-44.882	6958	-0.20041954	-2.809147849	0.034066612
174067	3/1/2019 5:33	A	74351.31512	5314.684883	-73.416	-44.867	1104	-0.229276897	-2.822874427	0.025886759
174067	3/1/2019 6:21	1	1562583.595	34054.90537	-73.439	-44.851	2920	-0.289649975	-2.782541035	0.011373615
174067	3/1/2019 11:30	A	5341702.369	449398.1311	-73.432	-44.806	18528	-0.318594908	-1.975649405	0.035005305
174067	3/1/2019 12:55	B	1773474.907	367310.0933	-73.428	-44.803	5056	-0.313661263	-1.974613086	0.03331657
174067	3/1/2019 13:05	B	1928871.733	374008.2667	-73.438	-44.805	623	-0.315688845	-2.041589514	0.034916089
174067	3/1/2019 13:35	A	275624714.3	4190176.172	-73.441	-44.874	1831	-0.317583935	-2.513247948	0.02958721
174067	3/1/2019 14:40	0	625303420.9	4806887.598	-73.454	-44.884	3881	-0.221095988	-2.866799476	0.009810486

174067	3/1/2019 15:08	B	19257636.25	1371199.746	-73.45	-44.885	1681	-0.186852066	-2.866799476	0.014468656
174067	3/1/2019 15:28	B	11026183.16	4286090.836	-73.454	-44.901	1201	-0.196927208	-2.688562279	0.022498999
174067	3/1/2019 16:05	B	281762.5767	54252481.42	-73.374	-44.907	2240	-0.226895563	-1.92896912	0.047556564
174067	3/1/2019 16:42	B	7052237.373	3470662.627	-73.37	-44.95	2192	-0.255167397	-1.450066066	0.033993902
174067	3/1/2019 17:09	B	1686609.508	587698.992	-73.36	-44.966	1591	-0.262235821	-1.203853864	0.024652313
174067	3/1/2019 18:23	B	1204827.403	2762291.097	-73.302	-45.009	4488	0	-0.750484671	0
174067	3/2/2019 1:28	A	208230089.5	33995684.98	-73.398	-44.913	25502	-0.909302468	-2.214263502	0.209552231
174067	3/2/2019 2:35	B	186207463.6	8490058.903	-73.343	-44.886	3997	-0.922363918	-2.647780882	0.212607378
174067	3/2/2019 3:05	B	18143646.02	1118648.482	-73.338	-44.887	1817	-0.922111331	-2.773905851	0.21208801
174067	3/2/2019 3:42	A	37480181.38	12540519.13	-73.421	-44.894	2213	-0.923438363	-2.84630112	0.210989909
174067	3/2/2019 4:18	B	145907112.8	4588865.657	-73.438	-44.882	2155	-0.921767778	-2.866799476	0.209039755
174067	3/2/2019 4:29	B	18276236.8	1007759.703	-73.437	-44.882	687	-0.921688403	-2.866799476	0.20860793
174067	3/2/2019 4:53	B	2679330.766	53743.7337	-73.439	-44.879	1439	-0.920487827	-2.866799476	0.207631407
174067	3/2/2019 5:24	2	523462.1822	129650.3178	-73.459	-44.873	1848	-0.922370936	-2.866799476	0.207181752
174067	3/2/2019 5:28	B	562549.3482	103755.6518	-73.46	-44.873	217	-0.9226669	-2.866799476	0.206984435
174067	3/2/2019 6:03	B	2366432.641	402349.8585	-73.477	-44.87	2086	-0.921421953	-2.866799476	0.205843106

174067	3/2/2019 6:42	B	27690485.75	36643451.25	-73.463	-44.877	2386	-0.921142494	-2.866799476	0.205988426
174067	3/2/2019 7:04	B	1388550.276	1225229.724	-73.461	-44.876	1286	-0.919884999	-2.866799476	0.205988359
174067	3/2/2019 11:16	A	28989.332	46363.168	-73.373	-44.933	15147	-0.887419225	-1.750217603	0.201069735
174067	3/2/2019 12:34	B	1441118.781	331594.2194	-73.38	-44.94	4661	-0.878527876	-1.545400125	0.197379621
174067	3/2/2019 12:57	B	9185980.735	4186421.765	-73.382	-44.945	1355	-0.876814291	-1.540665143	0.196729168
174067	3/2/2019 13:04	B	2212099.573	396129.4267	-73.378	-44.944	454	-0.876814291	-1.521999254	0.196729168
174067	3/2/2019 14:16	B	4355018.333	539582.6667	-73.378	-44.954	4301	-0.863273088	-1.356607861	0.188880199
174067	3/2/2019 14:42	A	204155.6273	12920.87274	-73.404	-44.965	1569	-0.859670561	-1.288984555	0.186333386
174067	3/2/2019 14:42	B	164424.5142	10555.48575	-73.404	-44.965	24	-0.859670561	-1.288984555	0.186333386
174067	3/2/2019 15:08	1	7531834.415	827277.5854	-73.36	-44.964	1565	-0.86014242	-1.311397685	0.186679308
174067	3/2/2019 15:49	B	70980349.56	18435646.94	-73.351	-44.955	2408	-0.870838715	-1.501068461	0.191968924
174067	3/2/2019 16:21	A	11810726.18	2150106.319	-73.409	-44.926	1963	-0.883754485	-1.803554808	0.197410475
174067	3/2/2019 16:56	B	11652992.5	2080697.496	-73.428	-44.918	2067	-0.901613564	-2.404421131	0.203896193
174067	3/2/2019 17:28	B	123005067.2	635619.3115	-73.432	-44.885	1926	-0.919072121	-2.866799476	0.209082387
174067	3/2/2019 17:58	B	153308.6863	8914157.314	-73.408	-44.875	1828	-0.929581614	-2.824389083	0.212377379
174067	3/3/2019 0:21	B	131058693.4	463555417.1	-73.44	-44.798	22964	-0.940066952	-2.760773494	0.450779899

174067	3/3/2019 1:14	B	976975.1498	339281.3502	-73.387	-44.89	3179	-0.939652566	-2.777952717	0.448281975
174067	3/3/2019 2:00	B	2970427.962	183058.5376	-73.378	-44.922	2780	-0.938180828	-2.101836219	0.444798008
174067	3/3/2019 2:27	B	4587420.5	141512	-73.38	-44.915	1622	-0.937897687	-2.101836219	0.44499613
174067	3/3/2019 3:31	B	4075344.018	508698.4822	-73.362	-44.944	3791	-0.934605838	-1.539144911	0.442399498
174067	3/3/2019 4:03	B	5771096.109	2700342.391	-73.361	-44.945	1931	-0.93476254	-1.548795129	0.44328664
174067	3/3/2019 4:38	B	164962616	55063064	-73.338	-44.914	2100	-0.934995721	-1.696583908	0.446054697
174067	3/3/2019 5:14	A	10268213.37	4673177.133	-73.322	-44.906	2185	-0.934864709	-1.859849276	0.449612661
174067	3/3/2019 5:40	0	767559840.5	47753831.54	-73.304	-44.892	1522	-0.935696918	-1.911950905	0.451661952
174067	3/3/2019 6:13	B	1632561301	134241587.5	-73.294	-44.886	2033	-0.936929472	-1.877176689	0.452717727
174067	3/3/2019 7:20	A	581131.5678	2430074.932	-73.327	-44.904	4012	-0.937130913	-1.832882512	0.453347213
174067	3/3/2019 11:04	B	29835387.75	4064712.246	-73.303	-44.922	13395	-0.932393562	-1.453372108	0.451018532
174067	3/3/2019 12:30	B	49952944.18	4240364.819	-73.309	-44.932	5191	-0.930813884	-1.361932452	0.44911781
174067	3/3/2019 12:42	B	64532333.14	4849711.361	-73.318	-44.942	733	-0.930813884	-1.362933212	0.44911781
174067	3/3/2019 13:46	B	1258611386	24860784.61	-73.299	-44.956	3835	-0.930706991	-1.358371173	0.449528713
174067	3/3/2019 14:11	A	1358231.536	327544.9642	-73.296	-44.93	1468	-0.930819787	-1.398557562	0.449745484
174067	3/3/2019 15:01	0	14703596.99	4632723.013	-73.348	-44.914	3000	-0.933270477	-1.615775372	0.450485552

174067	3/3/2019 15:28	B	38639812.89	7987459.612	-73.348	-44.914	1660	-0.935372641	-1.838501015	0.45102927
174067	3/3/2019 15:50	B	65066564.11	13930915.89	-73.359	-44.914	1298	-0.935870466	-1.95883875	0.450623227
174067	3/3/2019 16:33	B	2098277182	94884590.16	-73.358	-44.909	2569	-0.935988889	-2.102723565	0.449474522
174067	3/3/2019 16:41	B	11474619.83	458344.6677	-73.355	-44.91	515	-0.935988889	-2.088084589	0.449474522
174067	3/3/2019 17:07	B	3823833.667	293110.8327	-73.361	-44.92	1541	-0.935654247	-1.971423681	0.447336337
174067	3/3/2019 17:38	B	2191927.946	41012856.55	-73.359	-44.919	1854	-0.936043213	-1.896002512	0.446199176
174067	3/4/2019 1:06	A	44737979815	883653865.9	-73.289	-44.923	26895	-0.939287114	-2.101561595	0.453584606
174067	3/4/2019 1:32	A	67665256.08	16099786.42	-73.352	-44.923	1554	-0.938967866	-2.129903739	0.453305192
174067	3/4/2019 2:42	B	18453295.42	103274.5753	-73.354	-44.902	4190	-0.938854747	-2.389428925	0.454612257
174067	3/4/2019 3:36	B	7953796.259	524506.2407	-73.352	-44.898	3247	-0.936803499	-2.55596765	0.452537068
174067	3/4/2019 4:12	B	165988.4717	30349733.53	-73.387	-44.899	2180	-0.935902066	-2.245415838	0.44827845
174067	3/4/2019 5:04	B	194027723.9	6041125.084	-73.399	-44.957	3096	-0.937432535	-1.545832799	0.443400818
174067	3/4/2019 5:22	A	144103357.6	176878.8961	-73.369	-44.95	1057	-0.937021155	-1.47001372	0.441712662
174067	3/4/2019 10:48	B	28840369.78	3970330.72	-73.471	-45.028	19583	-0.936644919	-0.872847877	0.432153013
174067	3/4/2019 12:32	A	124571.737	54294.76298	-73.327	-44.951	6242	-0.937593791	-1.323947129	0.45332701
174067	3/4/2019 13:21	B	22197648.53	1595865.466	-73.316	-44.946	2918	-0.938737185	-1.313547121	0.456095576

174067	3/4/2019 13:43	B	3118923.293	75768.70655	-73.313	-44.948	1321	-0.938900865	-1.297557612	0.456395565
174067	3/4/2019 14:43	B	2280307.219	279395.2815	-73.304	-44.947	3613	-0.939191603	-1.287212757	0.456685182
174067	3/4/2019 14:58	B	2586450.86	342129.1401	-73.31	-44.947	917	-0.939191603	-1.297557612	0.456685182
174067	3/4/2019 15:17	A	34092873.39	1292139.113	-73.313	-44.943	1157	-0.938268984	-1.304149742	0.455393953
174067	3/4/2019 15:37	B	4240567.383	38412.61683	-73.329	-44.95	1143	-0.937643459	-1.309399094	0.454712608
174067	3/4/2019 16:33	B	3192360.632	255193.8678	-73.324	-44.947	3395	-0.937593791	-1.333067504	0.45332701
174067	3/4/2019 16:43	A	204137.7285	75594.77148	-73.328	-44.952	590	-0.937593791	-1.323947129	0.45332701
174067	3/4/2019 17:22	B	5624150.213	92608.28712	-73.326	-44.955	2346	-0.938152077	-1.259677827	0.453106559
174067	3/5/2019 1:05	B	9359265.055	2142579.945	-73.303	-44.951	27760	-0.846646411	-1.343753367	0.239278705
174067	3/5/2019 2:30	B	208625329.5	341774312.5	-73.436	-44.981	5113	-0.836065443	-1.163490662	0.217923578
174067	3/5/2019 3:16	1	5107225.569	598038.9311	-73.459	-44.984	2786	-0.834424065	-1.222763617	0.208481167
174067	3/5/2019 4:47	B	25670668.06	3874150.437	-73.493	-45.005	5454	-0.835837982	-1.15198342	0.21601552
174067	3/5/2019 5:34	A	797866.0447	1876666.455	-73.383	-44.935	2807	-0.844116598	-1.504369052	0.231532599
174067	3/5/2019 6:09	B	2353037.929	474784.5714	-73.372	-44.931	2082	-0.849329134	-1.760023284	0.236222639
174067	3/5/2019 6:38	A	220213.7903	333450.7097	-73.413	-44.928	1747	-0.851964723	-1.893960295	0.235448426
174067	3/5/2019 7:12	B	700406.7573	229045.7427	-73.403	-44.923	2034	-0.853694516	-1.975613489	0.236301484

174067	3/5/2019 10:42	B	4361941.87	1054666.63	-73.395	-44.937	12598	-0.846565488	-1.583080584	0.228563563
174067	3/5/2019 12:23	B	255505599.6	98648.92532	-73.441	-44.983	6084	-0.833895731	-1.832822737	0.201764579
174067	3/5/2019 12:57	A	301899.0195	1204389.48	-73.481	-44.965	2016	-0.8378047	-2.304197099	0.202976533
174067	3/5/2019 13:10	B	13327940.94	3773701.561	-73.488	-44.936	815	-0.839786719	-2.29432832	0.204935315
174067	3/5/2019 14:39	B	6498	11980512.5	-73.459	-44.966	5327	-0.833276016	-1.701807487	0.202156973
174067	3/5/2019 14:47	A	1554604.204	18360.7958	-73.46	-44.986	498	-0.833321989	-1.567385334	0.202284937
174067	3/5/2019 15:20	B	856740.5	128018	-73.457	-44.987	1932	-0.833049646	-1.537542217	0.202108128
174067	3/5/2019 16:15	B	1835666.422	379358.5777	-73.453	-44.988	3326	-0.83343003	-1.524164268	0.202642643
174067	3/5/2019 16:23	B	2964283.031	190651.469	-73.455	-44.982	478	-0.833523998	-1.606735654	0.202980722
174067	3/5/2019 16:56	B	2352146.639	4433330.361	-73.466	-44.982	2007	-0.835149969	-1.802948925	0.203608282
174067	3/6/2019 0:28	B	4821813.91	24245230.09	-73.549	-44.934	27079	-0.846507313	-2.866799476	-0.291841682
174067	3/6/2019 0:40	A	196821.0604	7863.439598	-73.515	-44.925	744	-0.847166846	-2.866799476	-0.29190393
174067	3/6/2019 2:05	B	215357769.5	2387392.456	-73.537	-44.99	5094	-0.85647686	-2.866799476	-0.28179259
174067	3/6/2019 2:22	A	3662666.292	956218.2082	-73.516	-44.977	1014	-0.856380587	-2.866799476	-0.282082603
174067	3/6/2019 2:48	B	6854025.894	33830.60558	-73.521	-44.96	1577	-0.854419775	-2.866799476	-0.281712006
174067	3/6/2019 4:31	B	10189890.56	1258834.437	-73.541	-44.98	6191	-0.858331018	-2.819008612	-0.274183341

174067	3/6/2019 6:19	B	425853599.1	259945457.9	-73.539	-44.954	6468	-0.854767989	-2.866799476	-0.277713258
174067	3/6/2019 6:59	B	13350282.79	7665009.707	-73.542	-44.957	2363	-0.852889451	-2.866799476	-0.281703597
174067	3/6/2019 10:30	B	3967449.022	1158467.478	-73.464	-44.899	12692	-0.848122691	-2.688381663	-0.314926734
174067	3/6/2019 12:06	B	6044376098	187927458.7	-73.467	-44.952	5782	-0.852485263	-1.872163229	-0.311406613
174067	3/6/2019 12:38	B	48308171.24	1910533.76	-73.464	-44.954	1871	-0.854328294	-1.768956292	-0.310528002
174067	3/6/2019 14:10	B	20126383.26	670292.7362	-73.461	-44.968	5566	-0.857490682	-1.396090897	-0.31497169
174067	3/6/2019 14:18	B	1301146272	25702790.87	-73.462	-44.974	478	-0.857964497	-1.365089612	-0.315962516
174067	3/6/2019 14:25	B	175053358.8	3480051.741	-73.459	-44.975	418	-0.858244482	-1.358639256	-0.317245092
174067	3/6/2019 15:03	B	38744400.34	889768.1608	-73.458	-44.981	2241	-0.858812984	-1.133037515	-0.322803037
174067	3/6/2019 15:59	B	373473.0121	1540791.488	-73.376	-44.972	3393	-0.860003547	-0.987386343	-0.32688732
174067	3/6/2019 16:05	B	598401.5662	227943.4338	-73.376	-44.987	363	-0.860458063	-0.969118812	-0.327270621
174067	3/6/2019 16:41	B	11807038.71	748835.2935	-73.382	-45.009	2129	-0.861758492	-0.89188252	-0.323344569
174067	3/6/2019 17:41	B	2947720.472	295385.5278	-73.384	-45.008	3609	-0.863747865	-0.897234031	-0.319631263
174067	3/6/2019 18:19	B	418779.1759	1234965.824	-73.395	-45.034	2291	-0.864136363	-0.85434661	-0.316530372
174067	3/7/2019 0:28	A	391445.1834	296702.8166	-73.352	-45.107	22136	0.217553291	-0.402917582	0.09904577
174067	3/7/2019 1:38	B	1587346.373	271031.6272	-73.352	-45.121	4225	0.207157707	-0.365062188	0.074086442

174067	3/7/2019 2:06	B	1830370.181	651232.3189	-73.353	-45.112	1629	0.209410763	-0.378429544	0.084850859
174067	3/7/2019 2:28	B	2194517.815	765944.6848	-73.353	-45.113	1367	0.217553291	-0.392777052	0.094072668
174067	3/7/2019 4:06	1	181906.7688	493311.2312	-73.344	-45.084	5879	0.233992151	-0.455226561	0.141995622
174067	3/7/2019 4:17	B	4473628.703	903648.2971	-73.345	-45.085	618	0.233089011	-0.455226561	0.144654356
174067	3/7/2019 4:24	B	2466863.849	295734.6508	-73.345	-45.086	465	0.233992151	-0.455226561	0.141995622
174067	3/7/2019 5:54	B	1998588.279	436971.7214	-73.344	-45.095	5352	0.235920938	-0.444244229	0.135442523
174067	3/7/2019 6:10	B	58757253.8	41371188.7	-73.358	-45.087	982	0.252723688	-0.474493423	0.142080863
174067	3/7/2019 6:32	A	2481195.486	3603.014151	-73.365	-45.072	1325	0.271197067	-0.53777532	0.156820163
174067	3/7/2019 7:34	B	2537090.13	568282.8704	-73.368	-45.075	3703	0.287646013	-0.566705618	0.150212341
174067	3/7/2019 10:19	B	392249809.1	12198243.94	-73.4	-44.995	9897	0.534240998	-0.814026111	0.254736107
174067	3/7/2019 13:47	B	27350635.13	691569.8713	-73.411	-45.017	12500	0.55883891	-0.857648634	0.273752437
174067	3/7/2019 15:27	B	16169083.7	635016.3047	-73.396	-45.011	6002	0.568723661	-0.83734905	0.301292811
174067	3/7/2019 15:50	B	129931.2649	3322838.735	-73.375	-45.011	1357	0.580287686	-0.854817853	0.307345857
174067	3/7/2019 16:14	A	5144780.012	11216584.99	-73.413	-45.031	1466	0.590190496	-0.934275438	0.319591486
174067	3/7/2019 17:20	A	91216401.64	130812.8622	-73.414	-44.974	3920	0.498930034	-1.163490662	0.391440243
174067	3/7/2019 17:59	B	5071500.793	220727.7071	-73.415	-44.962	2359	0.430860746	-1.368554449	0.408280344

174067	3/8/2019 0:17	A	43686.98584	8995.514163	-73.388	-45.016	22661	1.081217219	-0.882870938	0.268511729
174067	3/8/2019 1:05	B	1465520.53	137753.9697	-73.392	-45.026	2910	0.720055451	-0.850994966	0.230224938
174067	3/8/2019 2:46	B	4109490.648	1665494.352	-73.393	-45.007	6083	1.55409861	-0.941272477	0.310130067
174067	3/8/2019 3:37	A	487602599.5	71851171.03	-73.391	-44.993	3030	1.885359297	-0.990229891	0.375230157
174067	3/8/2019 4:13	2	120117.4384	27037.06161	-73.393	-44.992	2138	1.862050351	-0.982786881	0.383117343
174067	3/8/2019 4:34	A	1569811.813	1200449.187	-73.384	-45.009	1286	1.748899164	-0.967679424	0.358890699
174067	3/8/2019 5:20	B	16471968.19	4407582.314	-73.385	-45.003	2734	1.862050351	-0.982417702	0.383117343
174067	3/8/2019 5:35	0	739155543	39518275	-73.357	-44.981	898	1.862050351	-1.011675755	0.383117343
174067	3/8/2019 6:11	3	168965.512	11539.48799	-73.398	-44.979	2200	1.603414835	-1.104385443	0.462077672
174067	3/8/2019 7:14	B	7768125.342	1854051.658	-73.413	-44.969	3775	1.181296096	-1.30541895	0.521132569
174067	3/8/2019 10:05	A	16495145.58	2443198.425	-73.44	-44.911	10279	0.457427029	-2.256841618	0.564836908
174067	3/8/2019 11:44	B	145080636.2	10420778.26	-73.474	-44.905	5931	0.597536035	-2.410463597	0.565624872
174067	3/8/2019 13:23	B	17629255	1462021.998	-73.426	-44.921	5941	0.48699757	-2.070334578	0.533260809
174067	3/8/2019 14:56	B	60201129.53	7142395.466	-73.406	-44.937	5577	0.349132121	-1.602593801	0.528574555
174067	3/8/2019 15:06	B	186399219.1	13667397.41	-73.428	-44.951	596	0.336210942	-1.58793692	0.529342901
174067	3/8/2019 15:20	B	4967187.923	1714897.077	-73.393	-44.941	804	0.317589313	-1.566359929	0.53663862

174067	3/8/2019 15:40	B	96556967.78	4362794.719	-73.394	-44.942	1253	0.307765388	-1.552467321	0.539539924
174067	3/8/2019 15:56	B	2226597.755	102700.245	-73.395	-44.943	928	0.307765388	-1.549249666	0.539539924
174067	3/8/2019 16:31	B	5671801.792	348680.2083	-73.432	-44.951	2124	0.520423483	-1.527523417	0.558176946
174067	3/8/2019 17:00	B	5464009.598	353115.4024	-73.429	-44.95	1731	0.598959108	-1.535460415	0.567353798
174067	3/8/2019 17:38	B	32674.55906	11381681.44	-73.432	-44.952	2255	0.741202489	-1.549659713	0.570210333
174067	3/9/2019 0:06	B	11410246.86	925070.1428	-73.474	-44.999	23319	-0.188713657	-1.441978665	-0.029739356
174067	3/9/2019 0:36	B	27034111.16	395813.8371	-73.421	-44.976	1774	-0.316233254	-1.452406881	-0.010826921
174067	3/9/2019 2:09	B	16990974.34	639210.656	-73.429	-44.983	5608	-0.59149186	-1.221145909	0.012543118
174067	3/9/2019 3:14	1	197395728.3	10523216.24	-73.344	-44.987	3864	-0.62292156	-1.056317149	0.012813424
174067	3/9/2019 4:04	B	51524097.07	2339049.433	-73.351	-45.012	2990	-0.62594189	-0.959408491	0.010195907
174067	3/9/2019 5:15	B	66308794.91	4393610.094	-73.431	-44.995	4266	-0.636235586	-0.953891902	0.010205045
174067	3/9/2019 7:34	B	17665273.45	89059945.05	-73.39	-45.013	8372	-0.582413312	-0.761419662	-0.022492533
174067	3/9/2019 11:31	B	928536545.5	4553539.499	-73.372	-45.141	14201	0.155339702	-0.347020566	-0.157494012
174067	3/9/2019 12:56	B	25897168.78	497007.2172	-73.362	-45.157	5131	0.345916957	-0.287723133	-0.208064317
174067	3/9/2019 13:12	B	64270677.15	769874.8489	-73.349	-45.167	958	0.312845037	-0.287723133	-0.205435711
174067	3/9/2019 14:25	B	4808941280	53169893.4	-73.344	-45.111	4327	-0.248342617	-0.463796065	-0.100248374

174067	3/9/2019 15:37	B	57031244.23	1486024.77	-73.356	-45.03	4362	-0.595610851	-0.793616653	-0.009207648
174067	3/9/2019 16:39	B	28493423.41	7479477.589	-73.407	-44.977	3699	-0.771291761	-0.969049498	0.019612421
174067	3/9/2019 17:14	1	55167008	5940.5	-73.391	-44.986	2115	-0.81925689	-1.006752618	0.022660078
174067	3/9/2019 17:15	B	7288562	10368	-73.39	-44.986	43	-0.81925689	-1.006752618	0.022660078
174067	3/10/2019 1:34	A	239832.6368	273371.8632	-73.357	-44.996	29947	0	-0.847067177	0.041126758
174067	3/10/2019 1:43	B	557258.9509	354355.5491	-73.372	-45.006	561	-0.439176245	-0.851377988	0.040585537
174067	3/10/2019 2:49	B	326582470.6	26860926.38	-73.401	-44.942	3935	-0.431884389	-0.956228606	0.04444068
174067	3/10/2019 3:12	B	3141680.675	927537.8247	-73.377	-44.985	1358	-0.49541953	-1.067989176	0.068547081
174067	3/10/2019 3:48	A	31623940.35	628958.1487	-73.363	-44.942	2180	-0.783899739	-1.46720509	0.094470618
174067	3/10/2019 4:30	B	10771155.98	858952.5186	-73.358	-44.92	2547	-0.940214805	-2.074965671	0.10428686
174067	3/10/2019 5:36	B	3589425.608	545066.8923	-73.35	-44.887	3962	-0.964874446	-2.562220941	0.106237193
174067	3/10/2019 6:35	A	38421.69861	52318.30139	-73.398	-44.929	3502	-0.722000158	-1.798463648	0.092475878
174067	3/10/2019 11:24	A	1353532.166	3461114.334	-73.482	-44.876	17348	-0.419519705	-2.866799476	0.067099872
174067	3/10/2019 13:05	B	2053858.318	393004.1821	-73.491	-44.875	6046	-0.322166923	-2.866799476	0.058567749
174067	3/10/2019 13:51	B	58569787.8	1475706.702	-73.465	-44.94	2790	-0.115970348	-2.460261745	0.031485549
174067	3/10/2019 14:10	B	19140622.58	358855.9199	-73.449	-44.937	1134	-0.101304287	-2.103916216	0.024522751

174067	3/10/2019 15:23	B	13422930.59	1005945.912	-73.438	-44.96	4406	-0.139122175	-1.663556431	0.017807802
174067	3/10/2019 17:00	B	11820572.32	589632.1774	-73.439	-44.957	5805	-0.266050255	-1.524736239	0.037707898
174067	3/11/2019 1:23	B	26109367.58	1890797.421	-73.324	-45.045	30144	0.324349259	-0.630234141	0.138759464
174067	3/11/2019 2:53	B	3788169.292	302976.7075	-73.36	-45.028	5396	0.662658466	-0.762381082	0.149702734
174067	3/11/2019 3:38	B	597384.8547	830724.1453	-73.388	-44.964	2724	0.271341715	-1.220007082	0.261435935
174067	3/11/2019 5:07	B	52786109.03	2096831.971	-73.371	-44.864	5361	-0.631265591	-2.756986852	0.319901778
174067	3/11/2019 6:12	B	145153416.8	26308113.2	-73.43	-44.792	3882	0.102798572	-1.675679258	0.229328863
174067	3/11/2019 6:50	B	67071927.01	1032485.493	-73.434	-44.753	2268	0.067642324	-0.941614804	0.133795836
174067	3/11/2019 11:11	A	7453.321582	208998.6784	-73.473	-44.514	15657	1.035307584	-2.676486104	-0.017162592
174067	3/11/2019 12:49	B	1787263.374	431817.6265	-73.504	-44.441	5909	-0.220708477	-2.557947139	0.030596738
174067	3/11/2019 13:45	B	989250826.4	131050706.6	-73.598	-44.514	3338	-0.19659156	-1.978229289	0.064419999
174067	3/11/2019 15:04	B	82977176.04	256623.9632	-73.59	-44.521	4749	0.97718061	-2.041244739	0.137522257
174067	3/11/2019 16:37	B	6419140.564	323820.4356	-73.537	-44.498	5555	0.316147461	-1.816971829	0.096204142
174067	3/11/2019 17:08	B	31661544.88	3349364.124	-73.505	-44.492	1905	0.05438712	-1.826775344	0.084495274
174067	3/11/2019 18:14	B	5822635.532	641574.468	-73.502	-44.482	3927	-0.291141318	-2.008619239	0.069245503
174067	3/12/2019 1:06	A	1.33299E+11	7104880180	-73.465	-44.263	24703	3.85306146	-2.62191147	0.121834396

174067	3/12/2019 2:19	A	24861306.46	6476982.542	-73.445	-44.257	4436	3.503422206	-2.761277525	0.415070808
174067	3/12/2019 3:27	B	1552278.775	256329.7248	-73.441	-44.247	4035	2.619445021	-2.82313522	0.525523014
174067	3/12/2019 3:41	B	266448714.4	57937928.07	-73.44	-44.255	877	2.485120293	-2.821575783	0.543226444
174067	3/12/2019 4:16	B	3987114.892	826269.6078	-73.438	-44.248	2068	2.545781526	-2.82313522	0.532033876
174067	3/12/2019 5:07	B	849842357.3	138726515.7	-73.438	-44.24	3070	2.798203067	-2.821575783	0.481422474
174067	3/12/2019 6:29	2	245878.296	115259.704	-73.468	-44.236	4923	2.777546781	-2.800899109	0.397039113
174067	3/12/2019 10:59	B	8688336.525	14121073.48	-73.537	-44.192	16226	0.977483315	-2.100808066	0.41296574
174067	3/12/2019 12:39	2	21866.35376	106696.1462	-73.474	-44.2	5967	1.476345091	-2.431981783	0.475998867
174067	3/12/2019 13:21	B	3027510.219	565734.7806	-73.469	-44.193	2524	1.407581851	-2.476212638	0.513665049
174067	3/12/2019 14:26	B	5768388.842	1123417.658	-73.462	-44.186	3892	1.304820087	-2.283343739	0.520718631
174067	3/12/2019 14:57	B	84525188.79	2124583.708	-73.469	-44.189	1883	1.181140694	-2.294914719	0.479626188
174067	3/12/2019 14:58	B	57325748.1	1444276.901	-73.471	-44.189	43	1.181140694	-2.294914719	0.479626188
174067	3/12/2019 15:37	A	514036.4478	2904146.052	-73.488	-44.213	2373	1.348527194	-2.355414576	0.427861387
174067	3/12/2019 16:13	B	11271218.93	3984611.571	-73.492	-44.216	2155	1.645411148	-2.492968683	0.394585177
174067	3/12/2019 16:36	B	9303656.405	2819024.595	-73.494	-44.218	1334	1.810198608	-2.500294257	0.383354793
174067	3/12/2019 17:54	A	1424528.861	557321.1394	-73.475	-44.214	4719	1.864263606	-2.60027268	0.441799284

174067	3/13/2019 1:02	B	2027817155	108093837.4	-73.409	-44.358	25649	0.127662542	-2.866799476	0.09581712
174067	3/13/2019 1:51	B	93838212.67	6024271.328	-73.404	-44.373	2942	-0.067375782	-2.866799476	0.098990851
174067	3/13/2019 3:17	B	5113034769	272539871.6	-73.376	-44.454	5199	0.058800267	-2.866799476	0.155188609
174067	3/13/2019 4:29	B	5526208.692	3683932.308	-73.374	-44.479	4276	0.195539956	-2.866799476	0.168948226
174067	3/13/2019 5:29	A	12304511.88	186372.1195	-73.391	-44.467	3607	0.234591583	-2.866799476	0.179008784
174067	3/13/2019 6:09	A	1692115.176	53017.32448	-73.36	-44.47	2411	0.195829456	-2.866799476	0.19835511
174067	3/13/2019 7:12	B	1238206.687	275379.8125	-73.346	-44.487	3773	0.26007182	-2.725555321	0.20125636
174067	3/13/2019 12:26	B	11138539.05	1110595.454	-73.288	-44.541	18863	-0.156027697	-1.290989656	0.205008459
174067	3/13/2019 13:58	A	73968783.97	5314232.534	-73.206	-44.532	5502	-0.446243988	-1.17173697	0.267652197
174067	3/13/2019 14:41	B	14728665.74	703630.7568	-73.189	-44.526	2556	-0.521201765	-1.09147477	0.28579693
174067	3/13/2019 14:43	B	32019.79543	5892457.205	-73.191	-44.526	154	-0.528209513	-1.084394352	0.287172056
174067	3/13/2019 15:19	B	1030678.086	663056.4143	-73.175	-44.53	2168	-0.531287597	-1.075056046	0.287085932
174067	3/13/2019 15:54	A	16404769.25	6482237.248	-73.254	-44.516	2108	-0.482315925	-1.203673527	0.282392411
174067	3/13/2019 16:27	B	15014659.53	4130825.467	-73.263	-44.516	1929	-0.396057168	-1.390169911	0.267486571
174067	3/13/2019 16:55	B	10091935.35	3076817.153	-73.272	-44.515	1703	-0.332860817	-1.526254941	0.259712848
174067	3/13/2019 17:36	0	90487056.02	4147048.977	-73.262	-44.519	2456	-0.279547123	-1.578585253	0.25084062

174067	3/13/2019 23:38	B	284662979.6	28386020.93	-73.314	-44.501	21732	-0.378305557	-1.280596604	0.255713931
174067	3/14/2019 0:48	A	14461442	840.5	-73.213	-44.535	4215	-0.293273718	-1.180871854	0.146919847
174067	3/14/2019 1:19	B	56844837.04	3030711.955	-73.199	-44.529	1865	-0.285396363	-1.1870041	0.147558709
174067	3/14/2019 2:53	2	413557.9771	10574.02287	-73.235	-44.536	5604	-0.205395025	-1.213337325	0.135720544
174067	3/14/2019 4:43	A	24608429.13	1312722.865	-73.28	-44.541	6616	0.100794319	-1.1379276	0.072879454
174067	3/14/2019 5:12	A	177419.204	104068.796	-73.283	-44.563	1758	0.111719627	-1.032664213	0.047403474
174067	3/14/2019 5:47	B	671027.3221	168813.1779	-73.285	-44.566	2060	0.152191308	-1.024317311	0.026120182
174067	3/14/2019 6:22	B	1149188.462	369816.038	-73.288	-44.567	2129	0.152191308	-1.010347416	0.026120182
174067	3/14/2019 6:51	B	1727327.972	469553.028	-73.287	-44.564	1740	0.166193612	-1.02920527	0.033235499
174067	3/14/2019 12:20	A	1411080.317	154682.1832	-73.268	-44.481	19703	-0.250144623	-2.378164813	0.231127205
174067	3/14/2019 13:31	B	2873476.765	558565.7352	-73.267	-44.48	4270	-0.250144623	-2.378164813	0.231127205
174067	3/14/2019 14:10	B	280830774.3	8732262.685	-73.281	-44.501	2360	-0.115425022	-2.272824956	0.209446352
174067	3/14/2019 15:34	B	5720474.764	478558.2362	-73.289	-44.512	5035	0.14043702	-1.910478183	0.162954753
174067	3/14/2019 16:12	B	91564318.05	48334910.95	-73.309	-44.497	2293	0.088272256	-2.215032845	0.188399861
174067	3/14/2019 16:32	B	3458512.498	1409688.502	-73.299	-44.49	1197	0.058548145	-2.428342964	0.20874291
174067	3/14/2019 17:14	B	19275656.9	1203044.098	-73.296	-44.479	2516	-0.076872448	-2.665154923	0.236749412

174067	3/15/2019 0:37	A	7201651.297	519461.2026	-73.305	-44.475	26590	-0.026078031	-2.572254366	0.843322261
174067	3/15/2019 0:46	A	692359.4486	340256.5514	-73.27	-44.48	522	-0.014596785	-2.533639613	0.842198495
174067	3/15/2019 2:17	B	4430156.746	781839.7536	-73.281	-44.475	5449	0.140120795	-2.630408436	0.832303704
174067	3/15/2019 2:28	B	3305488.254	295641.7459	-73.288	-44.479	697	0.147904132	-2.614910567	0.830239084
174067	3/15/2019 4:07	B	3744323.823	303620.6774	-73.299	-44.472	5899	0.234997199	-2.70788034	0.827902928
174067	3/15/2019 4:31	B	90798516.28	3436525.724	-73.295	-44.473	1476	0.234997199	-2.66601808	0.827902928
174067	3/15/2019 5:25	A	45974.22213	43190.77787	-73.288	-44.466	3241	0.225483865	-2.570103073	0.834961952
174067	3/15/2019 6:16	B	993818.6856	234843.8144	-73.291	-44.462	3067	0.059059237	-2.510473749	0.849153139
174067	3/15/2019 6:33	B	1815059.346	2083024.654	-73.263	-44.475	968	0.041062524	-2.442894534	0.8499298
174067	3/15/2019 7:10	A	473578.4813	2839209.519	-73.29	-44.438	2250	0.20999066	-2.388388323	0.834405318
174067	3/15/2019 12:04	A	468481195.8	24973444.7	-73.239	-44.514	17631	0.259079888	-2.358675284	0.810161166
174067	3/15/2019 13:46	B	83602622.98	4803429.521	-73.25	-44.492	6141	0.127124819	-2.478085205	0.826040781
174067	3/15/2019 14:34	B	197575419.8	20927312.69	-73.267	-44.474	2880	0.055786697	-2.581997027	0.843132015
174067	3/15/2019 15:15	B	4028499.654	399590.3455	-73.283	-44.466	2435	0.152217415	-2.570103073	0.838106778
174067	3/15/2019 16:05	B	3142821.18	509556.8205	-73.297	-44.454	3013	0.280140518	-2.680059033	0.819306331
174067	3/15/2019 16:14	2	635349.4302	88859.06981	-73.299	-44.475	522	0.298095167	-2.744972722	0.814900628

174067	3/16/2019 0:29	B	232982353.8	144121294.7	-73.321	-44.447	29710	-0.107945157	-2.148969742	0.226545907
174067	3/16/2019 1:59	B	145221509.9	726398.5925	-73.292	-44.434	5425	-0.242669174	-1.986966517	0.22521566
174067	3/16/2019 2:08	B	66975896.32	366226.1819	-73.292	-44.434	528	-0.324666187	-2.016294282	0.22938085
174067	3/16/2019 3:37	A	15198150.54	1604998.46	-73.283	-44.456	5352	-0.611215453	-2.374356382	0.248307586
174067	3/16/2019 4:19	B	15211999.95	579481.053	-73.277	-44.474	2492	-0.498837809	-2.581997027	0.258335765
174067	3/16/2019 4:29	B	19942.68169	516335.8183	-73.284	-44.476	594	-0.466162661	-2.627631135	0.260857692
174067	3/16/2019 5:07	2	311473.0621	65591.43793	-73.31	-44.471	2286	-0.150001349	-2.782065103	0.282335875
174067	3/16/2019 6:03	B	3888843.767	697926.2329	-73.32	-44.464	3351	0.39433464	-2.790134201	0.27624921
174067	3/16/2019 6:08	A	8458177.529	2013168.471	-73.324	-44.458	305	0.39433464	-2.790134201	0.27624921
174067	3/16/2019 11:58	B	6846082.033	1669807.967	-73.383	-44.422	20988	3.850749341	-2.866799476	-0.016698589
174067	3/16/2019 13:20	B	28810926	825479.0049	-73.345	-44.46	4940	1.20130663	-2.820800311	0.252563224
174067	3/16/2019 13:34	B	25154434.29	1086128.208	-73.349	-44.455	870	0.798630388	-2.820800311	0.273130727
174067	3/16/2019 14:08	B	29525680.02	971410.4771	-73.324	-44.471	2035	0.248065845	-2.80327682	0.286302497
174067	3/16/2019 15:03	A	19137600.4	4978146.098	-73.267	-44.459	3261	-0.084643439	-2.664047181	0.26726907
174067	3/16/2019 15:55	B	2158805.243	4729111.257	-73.293	-44.453	3129	-0.229680453	-2.484482514	0.253878529
174067	3/16/2019 16:35	B	903497.8132	682482.6868	-73.288	-44.453	2428	-0.302295629	-2.388388323	0.250449552

174067	3/16/2019 17:31	B	1867509.131	829057.3689	-73.287	-44.455	3309	-0.385598078	-2.385977426	0.249302899
174067	3/16/2019 18:12	B	3127621.732	545231.2677	-73.277	-44.458	2505	-0.429961971	-2.449191687	0.253655704
174067	3/17/2019 0:09	A	333598937.2	6617151.256	-73.316	-44.584	21401	-0.61664583	-1.738690665	-0.353785526
174067	3/17/2019 1:25	B	126029285	4054485.467	-73.343	-44.611	4551	-0.577612741	-1.848923882	-0.376614769
174067	3/17/2019 1:58	B	406897271.8	3312056.738	-73.391	-44.592	1989	-0.547610068	-2.517043572	-0.374814381
174067	3/17/2019 3:14	A	33777757.91	1287511.087	-73.513	-44.484	4574	-0.690778468	-2.580296017	-0.351210277
174067	3/17/2019 4:06	A	369883.625	129550.875	-73.494	-44.522	3108	-0.577172819	-2.550027774	-0.37370196
174067	3/17/2019 5:44	B	28811502.54	943387.9585	-73.5	-44.555	5895	-0.501826736	-2.602584809	-0.407475633
174067	3/17/2019 6:26	B	6889664508	136083512.6	-73.507	-44.542	2504	-0.502143742	-2.599907872	-0.408555182
174067	3/17/2019 11:39	B	5160130410	129446070.8	-73.65	-44.516	18778	-0.527196378	-2.405808656	-0.393093016
174067	3/17/2019 13:21	B	430040812.6	19641127.45	-73.543	-44.522	6103	-0.561829542	-2.272790736	-0.386925202
174067	3/17/2019 13:34	B	9484757.037	5388151.963	-73.507	-44.523	772	-0.570558413	-2.241709007	-0.385980972
174067	3/17/2019 15:15	B	3018404.518	589947.9823	-73.53	-44.514	6099	-0.577812333	-2.241709007	-0.383941508
174067	3/17/2019 15:35	A	9092.713373	500219.7866	-73.507	-44.523	1161	-0.533426856	-2.445655617	-0.389981395
174067	3/17/2019 15:38	A	412431.3259	57844.67408	-73.511	-44.531	181	-0.527367237	-2.461253538	-0.391115316
174067	3/17/2019 16:10	B	15079925.81	9890980.186	-73.517	-44.53	1965	-0.502143742	-2.608804259	-0.408555182

174067	3/17/2019 17:13	B	196473997.4	9207227.113	-73.454	-44.642	3756	-0.665346142	-1.431702602	-0.441654925
174067	3/18/2019 0:54	A	1711301.152	5810841.348	-73.41	-44.884	27660	-0.642062513	-2.824389083	-0.978264507
174067	3/18/2019 2:31	B	391443.85	25478430.15	-73.365	-44.928	5807	-0.722842082	-2.290489384	-0.952874435
174067	3/18/2019 2:46	B	212071.478	2389346.522	-73.361	-44.908	915	-0.729906485	-2.111526225	-0.950575635
174067	3/18/2019 4:30	B	385506.6797	11672239.32	-73.371	-44.963	6230	-0.735096265	-1.215838974	-0.964509142
174067	3/18/2019 5:29	B	262455655.4	3957127.067	-73.363	-44.997	3577	-0.750238293	-0.884262877	-0.965435711
174067	3/18/2019 6:03	B	3790230.874	148329.1258	-73.36	-45.013	2009	-0.733816919	-0.790069383	-0.959683474
174067	3/18/2019 11:32	A	20720974.69	230041.8148	-73.36	-45.102	19722	-0.695527775	-0.425710607	-0.896889562
174067	3/18/2019 12:31	B	2477952.775	202205.7255	-73.355	-45.122	3576	-0.692715626	-0.374035513	-0.883955498
174067	3/18/2019 13:10	B	3595178.166	264708.3339	-73.352	-45.129	2358	-0.69300836	-0.337347324	-0.877336395
174067	3/18/2019 14:44	B	188736097.3	13553762.73	-73.353	-45.116	5619	-0.697693882	-0.35788108	-0.880521692
174067	3/18/2019 15:11	B	12899126.16	761753.838	-73.339	-45.111	1602	-0.696877063	-0.362139392	-0.884044688
174067	3/18/2019 15:51	B	6083612.131	264010.3689	-73.326	-45.109	2384	-0.697956463	-0.387489165	-0.888717322
174067	3/18/2019 16:55	A	14084456.88	637581.6164	-73.369	-45.056	3881	-0.702747148	-0.587550984	-0.924732806
174067	3/18/2019 17:34	B	7154884.334	83419.66592	-73.376	-45.037	2315	-0.71533004	-0.699827751	-0.945028777
174067	3/19/2019 0:21	B	98467029.75	44823916.25	-73.373	-44.896	24447	-0.656126248	-2.290489384	-0.948801719

174067	3/19/2019 1:34	B	15413216.97	32772.03126	-73.362	-44.889	4339	-0.714997734	-2.613729605	-0.938228278
174067	3/19/2019 2:27	B	6038333.643	238180.3568	-73.366	-44.875	3199	-0.757227062	-2.460306621	-0.934187997
174067	3/19/2019 3:47	B	5858939.446	639185.5535	-73.376	-44.875	4791	-0.697958039	-2.636635636	-0.941226374
174067	3/19/2019 4:03	B	3851721.441	1239744.559	-73.377	-44.88	966	-0.658950032	-2.752700654	-0.94614198
174067	3/19/2019 5:23	A	1261072.021	7460.479168	-73.412	-44.948	4805	-0.713312749	-1.497849219	-0.997402737
174067	3/19/2019 6:47	B	2475858.189	585294.3111	-73.429	-44.956	5038	-0.765565015	-1.535460415	-1.004945574
174067	3/19/2019 7:24	B	18607706.69	801795.8142	-73.415	-44.922	2255	-0.693200483	-1.885232901	-0.996270623
174067	3/19/2019 13:03	B	39980589.26	8338543.24	-73.405	-44.863	20302	-0.641716101	-2.620033402	-0.94977115
174067	3/19/2019 13:42	1	2389522.934	1947999.566	-73.37	-44.919	2364	-0.632201743	-2.215658699	-0.953247163
174067	3/19/2019 14:14	B	4696151545	212192977.9	-73.392	-44.909	1885	-0.625621086	-2.027621407	-0.958364978
174067	3/19/2019 15:18	B	164547021.2	7388020.826	-73.385	-44.931	3859	-0.621757006	-1.937945358	-0.961056101
174067	3/19/2019 16:30	B	525599381	19885887.54	-73.312	-44.916	4339	-0.619900585	-1.956470675	-0.960759362
174067	3/19/2019 17:10	1	82109.31446	1070875.686	-73.37	-44.92	2398	-0.619900585	-1.963159207	-0.960759362
174067	3/19/2019 23:52	B	48131840.59	5332979.41	-73.409	-44.978	24077	-0.761250088	-1.286957384	-1.01186685
174067	3/20/2019 1:31	A	3012340.006	446144.9935	-73.421	-44.964	5993	-0.781136053	-1.338038809	-0.971233706
174067	3/20/2019 3:32	B	15522663.99	2378364.007	-73.408	-44.984	7235	-0.787725104	-1.093435099	-0.979316129

174067	3/20/2019 4:46	B	17867933.65	1523858.849	-73.401	-44.986	4467	-0.800637892	-0.94735308	-0.989141607
174067	3/20/2019 5:13	A	1927399.907	284589.093	-73.396	-45.03	1587	-0.813599156	-0.838352777	-0.997507983
174067	3/20/2019 5:26	A	42194.72653	7823.273475	-73.381	-45.027	758	-0.819429004	-0.81284044	-0.998996856
174067	3/20/2019 6:30	B	1381122	236672	-73.368	-45.042	3855	-0.837237785	-0.677734472	-1.006436345
174067	3/20/2019 7:07	B	3059336	9141144	-73.338	-45.058	2225	-0.849942981	-0.572713621	-1.009442076
174067	3/20/2019 11:06	2	24462.02245	160702.9776	-73.339	-45.154	14367	-0.888213508	-0.254447635	-1.03120135
174067	3/20/2019 12:48	3	22650.64488	11767.35512	-73.382	-45.172	6101	-0.912569121	-0.263820159	-1.044839742
174067	3/20/2019 13:19	B	621465.3011	138485.1989	-73.379	-45.18	1866	-0.910938157	-0.240406943	-1.044269913
174067	3/20/2019 13:40	B	10833301.33	278643.1709	-73.32	-45.173	1234	-0.906302404	-0.236785241	-1.041959585
174067	3/20/2019 15:02	B	16634912	169362	-73.312	-45.169	4955	-0.893848659	-0.19217701	-1.033561356
174067	3/20/2019 16:16	B	10732417.66	758928.8366	-73.275	-45.217	4414	0	-0.076261366	-1.036811648
174067	3/20/2019 16:41	B	26004738.86	10841415.14	-73.296	-45.176	1493	0	-0.113668123	-1.036722214
174067	3/20/2019 17:53	B	12871927.36	3016411.142	-73.397	-45.103	4320	-0.873687961	-0.355015348	-1.025409327
174067	3/21/2019 1:07	B	53996907.45	847864.5498	-73.385	-45.078	26066	-0.957921155	-0.438352196	-0.96715973
174067	3/21/2019 3:13	A	725013.8272	78046.17283	-73.314	-45.119	7558	-0.963377596	-0.301482506	-0.967049713
174067	3/21/2019 4:27	B	12115468.13	655613.8655	-73.443	-45.082	4447	-0.954681483	-0.458691736	-0.971100703

174067	3/21/2019 5:05	B	4247011509	191883054.9	-73.333	-45.132	2261	-0.954559683	-0.45467755	-0.970042887
174067	3/21/2019 6:09	B	54524330.28	2084056.215	-73.286	-45.1	3852	-0.959070106	-0.34469458	-0.96498334
174067	3/21/2019 6:44	B	7637103.493	770379.0066	-73.278	-45.099	2075	0	-0.311874613	-0.963032521
174067	3/21/2019 12:38	3	59162.80039	5173.699606	-73.301	-45.147	21230	-0.96994261	-0.209362808	-0.964239154
174067	3/21/2019 14:34	B	2336185.922	526758.5779	-73.263	-45.154	6996	0	-0.144707842	-0.961917152
174067	3/21/2019 14:53	B	2339963.746	694997.2536	-73.262	-45.158	1146	0	-0.156151942	-0.962102121
174067	3/21/2019 15:52	B	484951363	1339377.01	-73.284	-45.116	3548	-0.967835908	-0.262573413	-0.965035283
174067	3/21/2019 16:26	B	1567002.489	3985322.511	-73.322	-45.107	2032	-0.961317377	-0.329755036	-0.966466638
174067	3/22/2019 2:04	B	8282348.329	2954614.171	-73.435	-45.004	34696	-0.976188123	-0.967750031	-0.740958004
174067	3/22/2019 2:37	B	21044276.05	1467298.449	-73.42	-45.053	1933	-0.976691678	-0.845588555	-0.739558543
174067	3/22/2019 4:30	B	31502540.38	1738836.12	-73.339	-45.087	6820	-0.984872911	-0.519024928	-0.738527311
174067	3/22/2019 5:43	B	35132444.02	1283208.475	-73.325	-45.101	4331	-0.985621745	-0.430670362	-0.738410848
174067	3/22/2019 10:41	B	42952221.87	922516.1289	-73.346	-45.076	17888	-0.986106042	-0.494859124	-0.739924702
174067	3/22/2019 12:24	B	108691219.7	5319184.839	-73.338	-45.054	6201	-0.985244665	-0.568604045	-0.740608832
174067	3/22/2019 14:15	B	503915324.4	12729495.63	-73.342	-45.04	6680	-0.981693861	-0.667222107	-0.741414374
174067	3/22/2019 14:40	B	233188775.1	10646911.4	-73.347	-45.039	1472	-0.980230658	-0.677734472	-0.741664193

174067	3/22/2019 15:31	B	122219532.2	6742298.308	-73.335	-45.045	3095	-0.980699217	-0.719881634	-0.742011155
174067	3/22/2019 16:20	B	31230676.68	360303.3233	-73.372	-45.031	2910	-0.98162734	-0.761932391	-0.742465248
174067	3/22/2019 17:10	B	29393572.6	75272263.9	-73.39	-45.028	3003	-0.981282128	-0.799923657	-0.742299871
174067	3/23/2019 0:45	B	24131072.5	1799452.498	-73.436	-45.076	27284	-0.159861179	-0.703511432	-0.462950923
174067	3/23/2019 2:22	B	32466419.85	1621468.146	-73.411	-45.07	5834	-0.161930592	-0.703511432	-0.461118124
174067	3/23/2019 5:26	B	93037442.88	241486.1232	-73.448	-45.11	11016	-0.092232053	-0.537794983	-0.528114648
174067	3/23/2019 6:05	B	71159603.32	410393.6796	-73.436	-45.113	2343	-0.098211464	-0.520615932	-0.527270606
174067	3/23/2019 7:43	B	38445453.28	700452.7222	-73.418	-45.111	5876	-0.125804041	-0.509510751	-0.519306428
174067	3/23/2019 12:15	B	1009003151	310025829.3	-73.352	-45.116	16338	-0.189945106	-0.495740326	-0.484436602
174067	3/23/2019 13:45	B	10324636.11	950856.3854	-73.369	-45.095	5426	-0.208435234	-0.511797963	-0.466162
174067	3/23/2019 15:45	B	13122283.42	443079.0822	-73.382	-45.072	7181	-0.234313189	-0.604384441	-0.433980154
174067	3/23/2019 16:10	A	92964794.2	1402452.299	-73.402	-45.049	1501	-0.235446606	-0.657004013	-0.417869386
174067	3/23/2019 16:49	A	1534745.451	49027.0488	-73.374	-45.051	2334	-0.233817814	-0.682391919	-0.403026638
174067	3/24/2019 0:34	B	12729965.3	1902921.201	-73.267	-45.06	27891	0	-0.392674595	-0.299660926
174067	3/24/2019 1:09	B	92335103	6027449.5	-73.315	-45.095	2098	0	-0.349043232	-0.298095514
174067	3/24/2019 3:39	B	56086.25855	1817306.241	-73.313	-45.142	9015	0.382187951	-0.245547933	-0.382899041

174067	3/24/2019 4:28	B	4997026.01	47284010.99	-73.32	-45.154	2945	0.429778962	-0.23396891	-0.405146716
174067	3/24/2019 5:44	B	368266219.5	19629509.53	-73.322	-45.151	4539	0.539846289	-0.235605128	-0.41469361
174067	3/24/2019 7:20	B	6500612.43	690128.5699	-73.336	-45.164	5790	0.752460733	-0.2235138	-0.442376681
174067	3/24/2019 12:05	B	9873108.899	2335247.601	-73.371	-45.194	17091	1.339275259	-0.191664927	-0.487538112
174067	3/24/2019 13:16	B	5334735.349	4979802.651	-73.335	-45.129	4279	0.297285593	-0.302703223	-0.341705941
174067	3/24/2019 13:24	B	2682323.054	1567481.446	-73.336	-45.127	433	0.271712311	-0.311663313	-0.335014221
174067	3/24/2019 14:57	B	3897499.12	746280.8803	-73.334	-45.121	5630	0.09658984	-0.33058012	-0.311602868
174067	3/24/2019 15:26	B	6854920.115	449350.3852	-73.335	-45.122	1722	0.09658984	-0.33058012	-0.311602868
174067	3/24/2019 16:28	A	119591453.5	4519859.511	-73.359	-45.113	3729	0.072755754	-0.338358424	-0.310088077
174067	3/24/2019 17:07	1	267897.0164	69559.98365	-73.329	-45.117	2303	0.050907335	-0.333635552	-0.308684055
174067	3/25/2019 0:22	B	131255965.6	19247922.44	-73.257	-45.104	26125	0.648328447	-0.267090463	-0.422443356
174067	3/25/2019 0:35	B	544233429.9	1506342.616	-73.298	-45.126	781	0.648328447	-0.264610695	-0.434314399
174067	3/25/2019 2:03	B	25725380.86	4648451.145	-73.284	-45.13	5287	0.75233663	-0.243850362	-0.452506505
174067	3/25/2019 2:16	B	342711.6544	94413.34556	-73.31	-45.138	744	0.75233663	-0.245472802	-0.452506505
174067	3/25/2019 3:13	B	39011910.72	7734917.78	-73.317	-45.118	3437	0.604285631	-0.269753919	-0.408594913
174067	3/25/2019 4:19	B	2731803.617	531976.383	-73.313	-45.111	3975	0.500356026	-0.291260804	-0.383415108

174067	3/25/2019 4:42	B	2639536.458	591107.5419	-73.311	-45.129	1360	0.53059199	-0.297660313	-0.394158668
174067	3/25/2019 5:23	A	1398747.091	28335.40885	-73.329	-45.123	2458	0.473524111	-0.312223959	-0.373771293
174067	3/25/2019 6:26	A	220214.3155	710195.6845	-73.34	-45.114	3770	0.143586081	-0.372515564	-0.326005511
174067	3/25/2019 11:48	A	69269784.47	7313871.531	-73.367	-45.041	19348	-0.623030523	-0.714681608	-0.218972213
174067	3/25/2019 12:57	B	2825700.509	478048.4914	-73.376	-45.035	4148	-0.442044494	-0.744153905	-0.219302524
174067	3/25/2019 14:26	B	825619470.3	31195585.68	-73.369	-45.047	5324	-0.491117289	-0.732872549	-0.220798489
174067	3/25/2019 14:39	B	20880334.78	822722.2205	-73.371	-45.046	775	-0.491117289	-0.732872549	-0.220798489
174067	3/25/2019 15:44	B	424313219	19171695.05	-73.377	-45.044	3901	-0.337100549	-0.775433095	-0.224118154
174067	3/25/2019 16:08	A	43897.74772	34623.25228	-73.386	-45.037	1421	-0.288247659	-0.793775025	-0.222791994
174067	3/25/2019 16:44	B	13816084.86	524448.1352	-73.388	-45.033	2214	-0.15948404	-0.806230495	-0.224339234
174067	3/25/2019 17:49	1	1169447.194	3934976.806	-73.386	-45.041	3863	-0.337100549	-0.768602145	-0.224118154
174067	3/26/2019 1:46	B	1096346.71	1133.790395	-73.302	-45.13	28634	-0.007761799	-0.254358392	-0.365157353
174067	3/26/2019 4:05	B	3522416.133	374686.3669	-73.306	-45.121	8351	-0.269069012	-0.288845145	-0.335350688
174067	3/26/2019 4:26	B	365027513.3	446592.7252	-73.304	-45.097	1271	-0.574783	-0.344185684	-0.317491768
174067	3/26/2019 6:40	A	2384025.611	128027.3888	-73.375	-45.054	7999	-0.372040362	-0.655652105	-0.341788618
174067	3/26/2019 11:41	2	3270381.058	10037.44205	-73.366	-45.009	18062	0.011754612	-0.835561386	-0.344325115

174067	3/26/2019 13:52	B	4509777.193	384077.3074	-73.384	-45.011	7872	0.140805821	-0.85868075	-0.356653367
174067	3/26/2019 15:32	1	140872120.7	14878921.82	-73.354	-45.051	5990	-0.338349943	-0.716414484	-0.351596638
174067	3/26/2019 15:45	B	13460538.64	868465.863	-73.354	-45.046	811	-0.338349943	-0.692528188	-0.351596638
174067	3/26/2019 16:23	A	1443098637	36218637.03	-73.389	-45.039	2242	-0.338349943	-0.689846454	-0.351596638
174067	3/26/2019 17:27	B	138346588.4	10303460.09	-73.411	-45.032	3839	-0.243081643	-0.731767641	-0.35789816
174067	3/26/2019 23:54	B	256018401.3	31138099.67	-73.557	-44.998	23242	-0.003644861	-0.811796556	-0.370369434
174067	3/27/2019 1:14	A	1.53545E+11	4773905444	-73.382	-45.125	4774	-0.324594364	-0.716686341	-0.403558734
174067	3/27/2019 4:04	B	1012352.502	122772329.5	-73.355	-45.131	10205	-0.114731309	-0.50132331	-0.310236485
174067	3/27/2019 5:33	B	13855253.4	19396871.6	-73.372	-45.104	5362	-0.14331938	-0.476597994	-0.293891813
174067	3/27/2019 11:28	B	7669039.464	1601429.536	-73.329	-45.08	21295	-0.211424814	-0.443710991	-0.290599519
174067	3/27/2019 13:46	B	29416122.67	2501389.83	-73.336	-45.061	8288	-0.093663383	-0.55477261	-0.351040995
174067	3/27/2019 15:01	B	4360470.123	3076680.377	-73.357	-45.045	4522	-0.279335522	-0.700807809	-0.394881966
174067	3/27/2019 15:27	A	144886.6769	202227.8231	-73.38	-45.041	1509	-0.324594364	-0.743586828	-0.403558734
174067	3/27/2019 16:08	B	1043200.866	327759.134	-73.381	-45.041	2497	-0.336734666	-0.768652143	-0.405407795
174067	3/27/2019 16:37	B	2086924.5	304980.5	-73.382	-45.041	1711	-0.336734666	-0.768652143	-0.405407795
174067	3/27/2019 17:47	B	2539356.938	574655.5622	-73.381	-45.041	4219	-0.324594364	-0.768705713	-0.403558734

174067	3/28/2019 0:40	B	14796327.81	1039246.695	-73.387	-45.057	24746	-0.046906069	-0.596566177	-0.151476621
174067	3/28/2019 1:23	1	13722033.51	346222.4929	-73.368	-45.113	2594	-0.12649238	-0.426575519	-0.130673398
174067	3/28/2019 3:37	A	31635414.27	984608.2282	-73.324	-45.113	8070	-0.207983332	-0.346459315	-0.080392448
174067	3/28/2019 5:20	A	210377.186	5147.813959	-73.314	-45.122	6186	-0.252513787	-0.281226333	-0.061306245
174067	3/28/2019 5:21	B	170297.0396	4343.460417	-73.312	-45.122	39	-0.252513787	-0.281226333	-0.061306245
174067	3/28/2019 6:04	B	1140754.507	119519.9933	-73.307	-45.128	2594	-0.265974523	-0.262328706	-0.057117206
174067	3/28/2019 11:16	B	1245679947	102429827.5	-73.292	-45.047	18727	-0.121262382	-0.446251614	-0.079046042
174067	3/28/2019 14:35	B	34945734.97	3182878.034	-73.264	-45.065	11919	0.168175546	-0.689846454	-0.145121471
174067	3/28/2019 15:02	B	47702269.73	418511.2727	-73.32	-45.032	1608	0.398034576	-0.747881803	-0.1257446
174067	3/28/2019 15:43	A	764743.9817	5663624.018	-73.398	-45.035	2467	0.329157552	-0.792933452	-0.148750643
174067	3/28/2019 16:17	B	14572918.66	1238363.338	-73.359	-45.048	2069	0.132392876	-0.731339303	-0.152687847
174067	3/28/2019 16:44	1	919617.3418	24725.15816	-73.367	-45.052	1597	0.078036231	-0.709185882	-0.155710938
174067	3/28/2019 17:24	2	26033.12062	66391.87938	-73.396	-45.047	2397	0.239672998	-0.774070347	-0.167602101
174067	3/29/2019 0:09	A	29133754.69	22022529.81	-73.349	-45.118	24314	-0.14720338	-0.405650256	-0.157747528
174067	3/29/2019 1:50	B	73491800.38	220421112.1	-73.35	-45.132	6019	-0.186317117	-0.339671958	-0.133388807
174067	3/29/2019 3:13	A	14140371.03	440020.9742	-73.297	-45.113	5031	-0.228034712	-0.297431195	-0.119722174

174067	3/29/2019 5:07	B	7923126.183	1583650.317	-73.289	-45.11	6846	0	-0.282939321	-0.107795473
174067	3/29/2019 14:34	B	54700475.43	4551812.571	-73.234	-45.097	34005	0	-0.29292833	-0.058182834
174067	3/29/2019 14:59	B	34440074.13	7701550.867	-73.244	-45.114	1502	0	-0.333089763	-0.056418043
174067	3/29/2019 15:23	B	22538798.52	4750570.481	-73.303	-45.087	1425	0	-0.377174253	-0.046580717
174067	3/29/2019 15:37	B	30692361.86	812687.1434	-73.306	-45.067	849	0	-0.418245897	-0.046580717
174067	3/29/2019 16:25	B	67407660.5	91592	-73.331	-45.04	2884	0	-0.566513219	0
174067	3/29/2019 16:40	B	15643576.48	248520.0169	-73.358	-45.039	880	0	-0.586341941	0
174067	3/30/2019 1:02	2	621023.4606	96761.53942	-73.316	-45.151	30127	-0.850658413	-0.23293867	-0.333133384
174067	3/30/2019 2:50	B	265803343.5	6668624.508	-73.34	-45.141	6457	-0.853223267	-0.274688222	-0.339455795
174067	3/30/2019 4:30	B	11460787.07	537398.9252	-73.356	-45.129	6003	-0.855630185	-0.337347324	-0.34739956
174067	3/30/2019 4:39	B	9565013.309	845175.1907	-73.36	-45.127	542	-0.855293904	-0.340107757	-0.348063482
174067	3/30/2019 5:03	B	8543660.403	597912.0968	-73.357	-45.121	1461	-0.855017354	-0.355957111	-0.348595554
174067	3/30/2019 5:21	B	7582687.16	1092615.34	-73.343	-45.13	1080	-0.855680015	-0.368510417	-0.349674227
174067	3/30/2019 6:20	A	622644.6237	40229.87629	-73.354	-45.089	3536	-0.85298824	-0.464858599	-0.354621163
174067	3/30/2019 12:30	A	2452019.27	362097.2298	-73.331	-44.948	22207	-0.823978756	-1.38985121	-0.369144868
174067	3/30/2019 15:08	A	1223826.501	246447.4986	-73.359	-44.941	9446	-0.823011859	-1.550064768	-0.381415698

174067	3/31/2019 2:30	A	154847.5514	629384.9486	-73.413	-44.956	40924	-0.205577454	-1.425164928	-0.763165067
174067	3/31/2019 2:30	B	94419.70117	350622.2988	-73.412	-44.958	46	-0.205577454	-1.425164928	-0.763165067
174067	3/31/2019 6:02	B	4522629.497	735239.0028	-73.446	-44.955	12719	-0.160840273	-1.494365995	-0.79027696
174067	3/31/2019 6:35	B	4916021.115	3834889.385	-73.415	-44.97	1976	-0.184185323	-1.451475595	-0.78346614
174067	3/31/2019 12:20	2	978718.2903	216806.7097	-73.36	-44.912	20672	-0.350362676	-2.13757013	-0.644170698
174067	3/31/2019 14:35	B	9727831.719	1809498.781	-73.35	-44.899	8117	-0.393358977	-2.364374704	-0.622893429
174067	3/31/2019 15:48	B	5671501.057	2272705.443	-73.351	-44.899	4349	-0.393358977	-2.30960211	-0.622893429
174067	3/31/2019 16:21	B	26535840.92	11195777.58	-73.349	-44.908	2006	-0.393358977	-2.26545057	-0.622893429
174067	4/1/2019 0:18	B	9092960.744	4441844.256	-73.323	-44.906	28635	-0.697736973	-1.504641297	-0.733992875
174067	4/1/2019 0:37	B	27183144.27	6666151.728	-73.332	-44.912	1148	-0.713949542	-1.442060065	-0.733042064
174067	4/1/2019 1:55	A	898479.5135	278394.4865	-73.257	-44.94	4661	-0.757819265	-1.086637624	-0.7328157
174067	4/1/2019 2:22	B	83601.49539	1030927.005	-73.262	-44.941	1602	-0.769768218	-1.06589715	-0.731020567
174067	4/1/2019 3:39	A	923562.4324	220309.5676	-73.242	-44.942	4610	-0.78692292	-0.995970228	-0.728665206
174067	4/1/2019 4:32	A	19906.91443	53679.58557	-73.283	-44.935	3221	-0.731183154	-1.218858539	-0.736369785
174067	4/1/2019 5:38	A	9820131.749	152493.2508	-73.276	-44.912	3943	-0.714708696	-1.435850899	-0.731460512
174067	4/1/2019 6:14	A	4008203.946	124973.0543	-73.314	-44.918	2131	-0.695354365	-1.447716086	-0.737356911

174067	4/1/2019 12:08	B	15023925.39	6354404.613	-73.275	-44.918	21289	-0.755597513	-1.184186683	-0.728795472
174067	4/1/2019 14:06	A	89650.77984	14694.22016	-73.261	-44.946	7049	-0.773624963	-1.031595029	-0.731035203
174067	4/1/2019 15:20	B	1519276.811	297941.1886	-73.261	-44.948	4465	-0.773007854	-1.011742647	-0.731679594
174067	4/1/2019 16:03	B	4064991.421	122911.0792	-73.258	-44.949	2584	-0.776743267	-1.011742647	-0.731437013
174067	4/1/2019 17:03	B	5961471.84	194925.1596	-73.259	-44.952	3579	-0.776743267	-0.996109738	-0.731437013
174067	4/1/2019 17:40	B	5780163.304	342838.6963	-73.258	-44.953	2243	-0.773934215	-0.979318836	-0.7320189
174067	4/2/2019 0:28	B	34644276.2	372328.8013	-73.265	-44.937	24467	-0.740311391	-0.996362704	-0.998508032
174067	4/2/2019 1:22	B	49064418	247808	-73.265	-44.938	3254	-0.744121877	-0.931719503	-0.994396333
174067	4/2/2019 2:08	B	60000655.31	105660451.2	-73.213	-44.941	2734	-0.746177903	-0.876639171	-0.991557682
174067	4/2/2019 3:12	B	147327456	9162245.011	-73.192	-44.939	3861	-0.753392958	-0.818908118	-0.985965801
174067	4/2/2019 4:22	B	9837115.458	395839.042	-73.175	-44.94	4209	-0.745076002	-0.920898487	-0.992350298
174067	4/2/2019 5:13	A	384072.5718	98003.92817	-73.277	-44.924	3062	-0.718741383	-1.190070107	-1.02011318
174067	4/2/2019 6:29	B	9514682.554	1439175.946	-73.305	-44.92	4560	-0.701592204	-1.483824633	-1.042394568
174067	4/2/2019 6:56	B	6741084.248	918595.7523	-73.315	-44.917	1594	-0.698889116	-1.548780224	-1.046829144
174067	4/2/2019 7:36	B	6719922.557	831517.4426	-73.326	-44.915	2406	-0.69650638	-1.59386208	-1.051117995
174067	4/2/2019 13:34	B	92569450.09	15123154.41	-73.29	-44.942	21445	-0.706425094	-1.30439139	-1.031054141

174067	4/2/2019 14:34	B	3199308.189	191556.8112	-73.295	-44.935	3602	-0.706223821	-1.337310372	-1.031121992
174067	4/2/2019 15:13	B	5817311.791	227180.7093	-73.295	-44.928	2341	-0.705557003	-1.380009154	-1.03378727
174067	4/2/2019 16:45	B	5547515.05	664148.95	-73.314	-44.913	5558	-0.699566081	-1.555204261	-1.048964641
174067	4/2/2019 17:24	A	9870807112	149013848.3	-73.429	-44.926	2315	-0.696158367	-1.561144229	-1.050665188
174067	4/3/2019 0:15	B	2754394066	1564279258	-73.408	-45.068	24688	-0.582260289	-0.693424883	-1.081031946
174067	4/3/2019 0:56	B	38477469.42	11080513.08	-73.422	-45.06	2439	-0.593271076	-0.612440709	-1.067227605
174067	4/3/2019 1:54	B	1791969407	264114066.4	-73.392	-45.121	3481	-0.619990258	-0.503779312	-1.040635378
174067	4/3/2019 2:32	B	10046533.64	1091014.856	-73.404	-45.134	2286	-0.6343532	-0.450499485	-1.027651442
174067	4/3/2019 2:46	B	5182151.22	173125.7804	-73.377	-45.118	860	-0.635686424	-0.444205782	-1.026227892
174067	4/3/2019 4:27	B	12653429.02	1033531.978	-73.442	-45.16	6016	-0.661381011	-0.360674702	-1.005427496
174067	4/3/2019 5:35	B	864537611.4	62076421.09	-73.363	-45.065	4073	-0.606020887	-0.557472428	-1.053489135
174067	4/3/2019 5:50	B	20573956.43	1778883.573	-73.369	-45.065	954	-0.593512099	-0.617562717	-1.067046498
174067	4/3/2019 13:05	B	36001965.98	1792463.022	-73.348	-44.982	26058	-0.550802555	-0.993196894	-1.159995037
174067	4/3/2019 14:10	B	1449282674	54761428.12	-73.321	-44.935	3890	-0.547975777	-1.303640273	-1.165646512
174067	4/3/2019 15:41	B	40923013.13	2321282.867	-73.327	-44.923	5476	-0.556973489	-1.708202547	-1.174717951
174067	4/3/2019 16:57	B	18333329.88	1427168.117	-73.322	-44.904	4593	-0.561869759	-1.788176902	-1.181565796

174067	4/4/2019 2:05	B	674495382.5	2039310.021	-73.271	-45.115	32861	-0.893898299	-0.365272966	-1.081927803
174067	4/4/2019 4:05	B	532045386.2	8355303.824	-73.369	-45.101	7200	-0.887583309	-0.419889595	-1.086024282
174067	4/4/2019 6:55	B	217494818.6	27737701.43	-73.447	-45.06	10197	0	-0.637106229	-1.106089873
174067	4/4/2019 11:38	B	11220771446	348867702.9	-73.329	-44.871	16956	-0.792394828	-2.018432703	-1.11526397
174067	4/4/2019 13:15	B	50383504.63	332535.3656	-73.288	-44.849	5842	-0.801153679	-1.469177956	-1.118987808
174067	4/4/2019 13:48	B	274307670.6	44780095.89	-73.338	-44.849	1951	-0.803665322	-1.393964471	-1.120691311
174067	4/4/2019 14:10	B	56679304.5	199712	-73.273	-44.846	1369	-0.804397421	-1.574020432	-1.122550822
174067	4/4/2019 15:29	A	483095.1235	577941.3765	-73.359	-44.885	4739	-0.794762609	-2.495336949	-1.131311667
174067	4/4/2019 16:37	B	140520264.4	5324619.589	-73.384	-44.946	4091	-0.790850299	-1.950102035	-1.133129988
174067	4/4/2019 17:13	B	21131937.13	1232242.873	-73.385	-44.928	2137	-0.792604107	-1.799186439	-1.132138546
174067	4/5/2019 1:30	B	4458723242	138631919	-73.389	-44.904	29795	-0.578177856	-1.99985171	-1.629655845
174067	4/5/2019 3:38	B	435673463.9	57720333.09	-73.313	-44.928	7674	-0.584558411	-2.077028668	-1.628231397
174067	4/5/2019 4:53	B	172550098	9711874.477	-73.365	-44.91	4512	-0.593392386	-2.094941056	-1.624772265
174067	4/5/2019 11:18	B	62098003.86	891174.6369	-73.389	-44.931	23137	-0.570110461	-1.738072937	-1.643672619
174067	4/5/2019 13:19	B	2027447879	330956209.1	-73.376	-44.906	7234	-0.619456072	-1.947896003	-1.630660281
174067	4/5/2019 13:41	B	341312123.9	511148.6238	-73.351	-44.909	1314	-0.629193162	-1.970563122	-1.630832353

174067	4/5/2019 14:58	B	1291337382	1587679.162	-73.351	-44.904	4611	-0.652033514	-1.81974297	-1.63446641
174067	4/5/2019 15:23	B	199236818.4	21048804.08	-73.319	-44.917	1506	-0.650964637	-1.649872983	-1.639230306
174067	4/5/2019 16:19	1	3167030.172	92082.32775	-73.311	-44.924	3374	-0.648500553	-1.481716671	-1.651394169
174067	4/5/2019 16:58	B	14546101.65	368963.3515	-73.316	-44.923	2325	-0.656129203	-1.418857663	-1.65590494
174067	4/5/2019 17:21	1	1990790.299	1410214.201	-73.298	-44.935	1388	-0.658220188	-1.389813675	-1.662523902
174067	4/5/2019 17:57	B	2664551.126	673304.8744	-73.296	-44.938	2191	-0.658707168	-1.318476918	-1.665879121
174067	4/6/2019 0:59	B	1889143839	85357742.2	-73.257	-44.93	25285	-0.948358218	-1.61110047	-1.785422597
174067	4/6/2019 1:22	1	14571733.4	223411.6026	-73.314	-44.906	1376	-0.949079168	-1.620118202	-1.784809741
174067	4/6/2019 3:12	B	193703980	13910365.02	-73.317	-44.896	6609	-0.952472706	-1.739230076	-1.782624897
174067	4/6/2019 5:18	B	625373420.6	92151109.44	-73.327	-44.878	7579	-0.955184435	-1.935293957	-1.780816449
174067	4/6/2019 5:35	A	1011297543	45691119.69	-73.332	-44.889	993	-0.955184435	-1.935293957	-1.780816449
174067	4/6/2019 6:15	B	10447489.84	414400.6575	-73.331	-44.887	2407	-0.956520169	-1.948077056	-1.780256386
174067	4/6/2019 14:32	B	13186454.88	2120862.121	-73.322	-44.881	29846	-0.969300038	-2.015178153	-1.776746277
174067	4/6/2019 15:07	B	2115839.347	388923.1532	-73.349	-44.868	2080	-0.972835939	-2.125122576	-1.776197091
174067	4/6/2019 16:31	B	3098878.033	254277.9671	-73.367	-44.902	5065	-0.950106622	-2.417710016	-1.781179349
174067	4/6/2019 17:40	B	5827972.083	950938.4166	-73.372	-44.911	4102	-0.944554522	-2.165625748	-1.783038354

174067	4/7/2019 1:14	B	174927398.3	12615750.18	-73.341	-45.078	27249	-0.866832225	-0.532253923	-1.066711695
174067	4/7/2019 2:11	B	207330346.9	17247197.6	-73.307	-45.086	3425	-0.868988219	-0.484455585	-1.071353397
174067	4/7/2019 2:46	B	36655478.77	670606.2294	-73.329	-45.091	2106	-0.86943798	-0.494042301	-1.07258808
174067	4/7/2019 4:29	B	442350874.2	233518458.8	-73.458	-45.086	6170	-0.866189939	-0.644796709	-1.064620665
174067	4/7/2019 5:06	B	37830281.26	3082881.244	-73.462	-45.056	2226	-0.864689328	-0.732256788	-1.060171187
174067	4/7/2019 12:39	A	81049.31489	29821.18511	-73.327	-44.887	27162	-0.817101052	-1.886919159	-0.978291517
174067	4/7/2019 14:09	B	1471085159	55587667.62	-73.345	-44.89	5391	-0.816908265	-1.84307081	-0.977436812
174067	4/7/2019 14:18	1	5514230.877	398655.623	-73.32	-44.887	578	-0.817114519	-1.853130183	-0.978167509
174067	4/7/2019 14:59	B	1798854.901	323657.5991	-73.328	-44.887	2441	-0.817193481	-1.915074254	-0.97856063
174067	4/7/2019 15:38	A	7517061.26	548284.7398	-73.325	-44.892	2356	-0.817088675	-1.838670135	-0.979551622
174067	4/7/2019 16:33	1	1598980.119	585669.881	-73.301	-44.905	3301	-0.817018749	-1.66896927	-0.98181363
174067	4/7/2019 16:43	B	2511601.123	349142.8767	-73.331	-44.893	569	-0.816667306	-1.57266523	-0.981340549
174067	4/7/2019 17:17	A	18183.05725	52579.44275	-73.292	-44.9	2054	-0.814461897	-1.383203469	-0.980093027
174067	4/8/2019 1:03	B	12764248.9	3354609.605	-73.192	-44.941	27941	-0.819717775	-0.911554298	-1.881900699
174067	4/8/2019 1:37	B	121947909.6	14462734.92	-73.266	-44.927	2074	-0.81835749	-1.002686713	-1.881697049
174067	4/8/2019 2:19	B	2837716.701	259775.2992	-73.273	-44.928	2513	-0.814398336	-1.126607425	-1.878179065

174067	4/8/2019 2:42	B	43412085.94	12279399.06	-73.271	-44.936	1384	-0.813340303	-1.162356655	-1.876709409
174067	4/8/2019 4:05	B	4182014.713	689755.2872	-73.282	-44.939	4964	-0.807623706	-1.349862336	-1.871927044
174067	4/8/2019 5:29	3	162327.6764	4860.323587	-73.342	-44.884	5043	-0.793614525	-2.182136306	-1.887564927
174067	4/8/2019 6:34	B	64965.26701	5590799.233	-73.353	-44.873	3911	-0.790863714	-2.208839915	-1.890247521
174067	4/8/2019 7:12	B	743395.6999	1178214.3	-73.365	-44.869	2262	-0.788368913	-2.315860497	-1.887838135
174067	4/8/2019 12:28	A	1199233.011	312056.9892	-73.325	-44.9	18970	-0.798397654	-1.877425426	-1.882103072
174067	4/8/2019 13:44	B	53155.18619	1226541.314	-73.302	-44.908	4567	-0.805133423	-1.479011725	-1.881597945
174067	4/8/2019 13:47	B	6706.065298	3227358.935	-73.298	-44.915	197	-0.805133423	-1.45559098	-1.881597945
174067	4/8/2019 14:42	B	987432.797	295987.703	-73.308	-44.916	3259	-0.804064868	-1.465947394	-1.878080524
174067	4/8/2019 15:26	B	4695874.965	952807.5346	-73.317	-44.925	2674	-0.80325738	-1.49029032	-1.872811212
174067	4/8/2019 16:21	A	15293512.3	177692.7008	-73.308	-44.93	3266	-0.803691165	-1.467325712	-1.86983864
174067	4/8/2019 16:53	B	15137061.16	2481871.841	-73.315	-44.93	1956	-0.803194788	-1.467325712	-1.869471557
174067	4/9/2019 0:48	B	56258085.48	27639347.52	-73.298	-44.92	28461	-0.835862456	-1.607392995	-1.7405512
174067	4/9/2019 2:29	A	304298.467	494514.033	-73.323	-44.897	6097	-0.832827323	-1.855597554	-1.745993241
174067	4/9/2019 3:38	B	14148.25359	7528790.246	-73.34	-44.892	4102	-0.830323775	-2.206343404	-1.74765308
174067	4/9/2019 4:42	B	28953569.78	3838044.716	-73.348	-44.879	3847	-0.829486656	-2.368970724	-1.75078525

1740<br>67	4/9/2019<br>6:10	A	1442833<br>72.4	3619878.<br>141	-<br>73.3<br>67	-<br>44.8<br>93	5306	-<br>0.82873<br>5278	-<br>2.49100<br>1535	-<br>1.74729<br>4974
1740<br>67	4/9/2019<br>13:20	B	3118229<br>3.04	4575700.<br>956	-<br>73.4<br>37	-<br>44.8<br>62	2580<br>7	-<br>0.82796<br>8982	-<br>2.52343<br>3746	-<br>1.75812<br>299
1740<br>67	4/9/2019<br>15:57	2	124295.0<br>275	147303.4<br>725	-<br>73.3<br>29	-<br>44.8<br>84	9379	-<br>0.83162<br>9313	-<br>1.93203<br>5099	-<br>1.75260<br>693
1740<br>67	4/9/2019<br>16:13	B	1594542.<br>522	428167.9<br>785	-<br>73.3<br>29	-<br>44.8<br>83	977	-<br>0.83214<br>0262	-<br>1.90192<br>6738	-<br>1.75107<br>1788
1740<br>67	4/9/2019<br>16:34	A	5573092<br>3.72	4583902.<br>775	-<br>73.3<br>32	-<br>44.9<br>13	1280	-<br>0.83233<br>1078	-<br>1.97744<br>8771	-<br>1.74919<br>0182
1740<br>67	4/10/2019<br>0:38	B	6906859<br>69.2	6457031<br>1.26	-<br>73.3<br>2	-<br>44.8<br>81	2900<br>9	-<br>0.84068<br>639	-<br>2.14071<br>8334	-<br>1.74752<br>5067
1740<br>67	4/10/2019<br>2:11	B	7184519<br>90.2	2714679<br>5.78	-<br>73.3<br>27	-<br>44.8<br>74	5610	-<br>0.83972<br>5519	-<br>2.26923<br>7555	-<br>1.74810<br>9806
1740<br>67	4/10/2019<br>2:16	B	6656371<br>7.78	2527340.<br>217	-<br>73.3<br>3	-<br>44.8<br>74	281	-<br>0.83947<br>7175	-<br>2.28791<br>1365	-<br>1.74787<br>9116
1740<br>67	4/10/2019<br>3:12	1	4814463<br>0.55	1217706.<br>446	-<br>73.4<br>07	-<br>44.8<br>85	3389	-<br>0.83813<br>5775	-<br>2.44487<br>5397	-<br>1.74513<br>3291
1740<br>67	4/10/2019<br>4:10	B	7767413.<br>66	324672.8<br>401	-<br>73.3<br>56	-<br>44.8<br>82	3439	-<br>0.83762<br>9395	-<br>2.56507<br>0251	-<br>1.74543<br>8293
1740<br>67	4/10/2019<br>4:30	B	7763884.<br>697	450275.3<br>033	-<br>73.3<br>55	-<br>44.8<br>83	1239	-<br>0.83615<br>3147	-<br>2.63522<br>9192	-<br>1.74507<br>8947
1740<br>67	4/10/2019<br>6:29	3	10775.31<br>19	6991.188<br>105	-<br>73.4<br>33	-<br>44.8<br>71	7117	-<br>0.82896<br>6033	-<br>2.86679<br>9476	-<br>1.74519<br>1184
1740<br>67	4/10/2019<br>12:06	B	4538847<br>62.3	1411706<br>3.65	-<br>73.5<br>21	-<br>44.8<br>66	2023<br>8	-<br>0.82751<br>5044	-<br>2.86679<br>9476	-<br>1.74415<br>0545
1740<br>67	4/10/2019<br>14:26	B	5373218<br>4.39	546998.1<br>134	-<br>73.4<br>65	-<br>44.8<br>86	8367	-<br>0.82677<br>2377	-<br>2.86679<br>9476	-<br>1.73937<br>0003
1740<br>67	4/10/2019<br>14:35	B	1682137.<br>653	715487.3<br>47	-<br>73.4<br>35	-<br>44.8<br>84	543	-<br>0.82702<br>1069	-<br>2.86679<br>9476	-<br>1.73911<br>8994
1740<br>67	4/10/2019<br>15:38	B	1251589.<br>575	460844.4<br>245	-<br>73.4<br>46	-<br>44.8<br>84	3823	-<br>0.82867<br>7775	-<br>2.50401<br>8505	-<br>1.73218<br>5136

174067	4/10/2019 16:00	2	1129909.055	10875.94474	-73.391	-44.926	1284	-0.830002433	-1.887310408	-1.724389697
174067	4/10/2019 16:11	B	526205.0632	90020.93677	-73.389	-44.926	691	-0.830898598	-1.794248534	-1.72185442
174067	4/10/2019 17:17	3	34359.99285	104466.0072	-73.359	-44.916	3924	-0.837163359	-2.017868795	-1.729668533
174067	4/10/2019 17:53	B	688770.2371	183894.2629	-73.358	-44.915	2189	-0.837234088	-2.084748302	-1.731184413
174067	4/11/2019 0:21	B	108163178.9	160665.5651	-73.346	-44.875	23258	-0.795600824	-2.445789019	-1.83145809
174067	4/11/2019 1:46	B	30990559.39	218036.6079	-73.343	-44.882	5108	-0.795616118	-2.63569213	-1.822628423
174067	4/11/2019 2:03	A	14982.68769	47423.81231	-73.368	-44.911	1027	-0.795991923	-2.201642624	-1.811635254
174067	4/11/2019 4:16	B	2571983.664	519836.8363	-73.361	-44.908	7963	-0.793783921	-2.49162125	-1.815214702
174067	4/11/2019 4:26	A	2543038.4	212181.6	-73.386	-44.894	626	-0.792773429	-2.723570538	-1.819331634
174067	4/11/2019 5:29	1	4100115.742	82509.25789	-73.453	-44.851	3787	-0.788644565	-2.740551885	-1.84095539
174067	4/11/2019 6:11	1	8040736.662	1314963.838	-73.412	-44.817	2516	-0.788096613	-2.323349185	-1.856876567
174067	4/11/2019 11:56	A	6015275.797	188766.2035	-73.386	-44.811	20654	-0.794545949	-1.725004326	-1.865470697
174067	4/11/2019 13:52	B	27788889.46	4604143.539	-73.39	-44.818	6967	-0.793888556	-2.039562397	-1.850774537
174067	4/11/2019 14:10	B	238486542.6	2659569.927	-73.397	-44.862	1067	-0.794614268	-2.094751046	-1.848585714
174067	4/11/2019 15:16	B	385936.0187	1031374.481	-73.368	-44.853	3961	-0.796008571	-2.280988312	-1.839060291
174067	4/11/2019 15:49	A	75316860.75	2848456.25	-73.375	-44.882	2011	-0.795261913	-2.417896228	-1.832579485

174067	4/11/2019 15:58	B	38844512.97	1492699.529	-73.405	-44.875	549	-0.795672453	-2.49862895	-1.831752513
174067	4/11/2019 16:57	B	5292755.731	363602.7685	-73.368	-44.889	3529	-0.795096527	-2.626492573	-1.823982974
174067	4/11/2019 17:32	B	127711188.4	2524716.596	-73.362	-44.886	2085	-0.795017601	-2.644053286	-1.822349731
174067	4/12/2019 1:14	B	3718805511	115624003.5	-73.316	-44.918	27736	-0.961465243	-2.69523332	-1.644010697
174067	4/12/2019 1:53	B	20514739.64	188080.8566	-73.403	-44.901	2309	-0.961634344	-2.608925311	-1.644069831
174067	4/12/2019 2:23	B	21534239.56	18726245.44	-73.387	-44.904	1827	-0.961634344	-2.555563979	-1.644069831
174067	4/12/2019 4:09	B	4691344.41	1837811.59	-73.397	-44.906	6334	-0.963166303	-2.51086876	-1.644746997
174067	4/12/2019 5:09	B	1430053400	233438412.7	-73.402	-44.896	3654	-0.964033742	-2.688562279	-1.645684838
174067	4/12/2019 6:50	B	9098127.417	1872949.583	-73.414	-44.889	6057	-0.963519488	-2.866799476	-1.646906494
174067	4/12/2019 11:44	2	1488534.04	13425.95992	-73.368	-44.874	17617	-0.954843725	-2.49862895	-1.64241513
174067	4/12/2019 13:44	B	6827108.983	494249.517	-73.383	-44.887	7205	-0.956143406	-2.644053286	-1.641324227
174067	4/12/2019 14:56	B	5186745.775	709248.2252	-73.373	-44.89	4336	-0.951094906	-2.365984421	-1.636882171
174067	4/12/2019 15:24	A	373188.154	3241884.346	-73.334	-44.904	1665	-0.950438918	-1.902247644	-1.6337926
174067	4/12/2019 15:37	2	1814567.679	101545.3208	-73.334	-44.922	790	-0.949764258	-1.718711646	-1.63156792
174067	4/12/2019 16:35	A	22282105.29	1386062.712	-73.344	-44.954	3487	0	-1.116130144	-1.63162336
174067	4/12/2019 17:14	A	757652.9054	29183.09457	-73.338	-44.992	2294	0	-0.879454823	0

174067	4/12/2019 17:22	B	1448778.489	178572.0112	-73.352	-44.973	475	0	-0.89935806	-1.634898754
174067	4/13/2019 1:44	B	1565030105	48681273.16	-73.391	-44.9	30125	-0.937874994	-2.370188332	-1.724423715
174067	4/13/2019 2:20	B	92243973.92	1570418.582	-73.393	-44.905	2165	-0.937421565	-2.435004225	-1.725404126
174067	4/13/2019 3:38	A	832517.6857	379618.3143	-73.381	-44.893	4712	-0.937163478	-2.687904092	-1.727456386
174067	4/13/2019 4:50	B	119274012.5	1173512	-73.398	-44.905	4312	-0.938285923	-2.600259198	-1.7286272
174067	4/13/2019 5:27	B	10357715.26	547207.2395	-73.398	-44.903	2212	-0.938907021	-2.523142233	-1.729083693
174067	4/13/2019 5:37	B	9320876.502	643981.498	-73.401	-44.9	632	-0.939840319	-2.5550203	-1.730061966
174067	4/13/2019 7:10	B	9531711.908	1139793.092	-73.411	-44.911	5549	-0.941229703	-2.350526215	-1.730667441
174067	4/13/2019 11:31	B	16922412.37	1607656.135	-73.397	-44.915	15689	-0.941542179	-2.24223741	-1.730791167
174067	4/13/2019 13:17	B	7085493.819	1826767.181	-73.425	-44.903	6330	-0.94220596	-2.533582608	-1.732843326
174067	4/13/2019 14:29	B	20116546.62	1273553.378	-73.428	-44.905	4341	-0.939972028	-2.51086876	-1.730051972
174067	4/13/2019 14:58	B	113879758.1	27093222.91	-73.326	-44.913	1737	-0.93812513	-2.437884085	-1.727691404
174067	4/13/2019 16:13	B	53028589.27	11532095.23	-73.327	-44.913	4506	-0.937664083	-2.13757013	-1.721008291
174067	4/13/2019 17:54	B	3914030.792	537859.7077	-73.328	-44.914	6056	-0.934772336	-1.867799365	-1.714924876
174067	4/14/2019 1:32	B	5043317504	190556519.6	-73.396	-44.889	27461	-0.973791597	-2.609549028	-1.729139301
174067	4/14/2019 1:52	B	328030010.1	12441926.95	-73.38	-44.887	1218	-0.973791597	-2.597214536	-1.729139301

174067	4/14/2019 3:10	B	93056976.92	2555833.079	-73.39	-44.89	4634	-0.973889876	-2.613729605	-1.729695046
174067	4/14/2019 3:39	B	45079514.97	1003523.533	-73.389	-44.888	1761	-0.973741886	-2.597214536	-1.729639554
174067	4/14/2019 4:52	B	14976288.5	1841408.503	-73.365	-44.89	4371	-0.972542064	-2.470471822	-1.72846801
174067	4/14/2019 5:06	B	5339235.87	1097465.13	-73.352	-44.886	874	-0.972002728	-2.470471822	-1.728056717
174067	4/14/2019 5:21	B	27697239.94	34161314.56	-73.346	-44.884	890	-0.971708406	-2.41564217	-1.728018639
174067	4/14/2019 6:08	B	2088135212	64923576.04	-73.362	-44.888	2843	-0.971111605	-2.373153651	-1.727380216
174067	4/14/2019 11:18	A	1355878.899	4603.601054	-73.324	-44.882	18595	-0.969329869	-1.870583871	-1.723059473
174067	4/14/2019 14:33	B	4807029.018	607119.4818	-73.319	-44.885	11651	-0.969853854	-1.764322452	-1.722794394
174067	4/14/2019 15:12	B	6196382.668	598250.3317	-73.32	-44.887	2349	-0.970228006	-1.853130183	-1.723101573
174067	4/14/2019 15:54	A	1431015.44	172773.0597	-73.329	-44.888	2522	-0.970348175	-1.967414837	-1.723928802
174067	4/14/2019 16:28	B	946389.1009	177375.8991	-73.331	-44.889	2072	-0.970889288	-1.967414837	-1.724280121
174067	4/14/2019 17:32	A	6393480.237	1663406.263	-73.319	-44.906	3830	-0.971518031	-1.803507784	-1.723385636
174067	4/14/2019 18:14	B	1106695.127	143681.3731	-73.327	-44.916	2520	-0.972481876	-1.711582963	-1.723179553
174067	4/15/2019 1:20	B	31050365.65	974854.3531	-73.322	-44.899	25547	-0.913270125	-1.927902978	-1.821637982
174067	4/15/2019 4:27	B	30139848	381064.5	-73.344	-44.896	11251	-0.912511756	-2.121823262	-1.823276695
174067	4/15/2019 5:11	B	1087857711	78108946.75	-73.382	-44.869	2596	-0.910828108	-2.112943412	-1.823483997

174067	4/15/2019 6:26	2	44109.05625	110471.4437	-73.333	-44.888	4523	-0.910384005	-1.990873804	-1.82386356
174067	4/15/2019 11:05	B	10795995.35	753419.154	-73.338	-44.907	16761	-0.916040749	-1.986177877	-1.822881031
174067	4/15/2019 12:46	B	9988372.559	944573.4411	-73.335	-44.905	6015	-0.91519742	-2.003565334	-1.823373719
174067	4/15/2019 14:07	B	4738791257	179049937.6	-73.336	-44.908	4899	-0.914491547	-2.045860937	-1.824571308
174067	4/15/2019 16:12	A	592962.2749	99960.22507	-73.342	-44.904	7482	-0.914953409	-2.093971705	-1.824684044
174067	4/15/2019 16:40	B	710713.3471	127999.1529	-73.343	-44.905	1650	-0.914587724	-2.045860937	-1.824294241
174067	4/15/2019 17:12	2	731694.7746	291171.2254	-73.333	-44.894	1950	-0.912030883	-2.167153301	-1.824033398
174067	4/15/2019 17:55	A	17336699.81	28696.6898	-73.332	-44.864	2557	-0.903451049	-1.700589677	-1.826531451
174067	4/16/2019 1:08	A	542035443.3	4152301.732	-73.346	-44.873	25975	-0.891114216	-1.941581097	-1.783270745
174067	4/16/2019 2:49	B	32275751.02	1963693.977	-73.343	-44.856	6101	-0.891222607	-1.856052493	-1.786523456
174067	4/16/2019 5:27	B	2987171757	538227536.2	-73.313	-44.874	9483	-0.891130959	-1.988548109	-1.787626459
174067	4/16/2019 6:08	B	2408719440	523759754.8	-73.316	-44.874	2423	-0.891130959	-2.062861853	-1.787626459
174067	4/16/2019 10:54	B	39448353.2	151932419.3	-73.365	-44.869	17198	-0.890172459	-2.401863969	-1.788183324
174067	4/16/2019 12:34	B	1695970.539	1120574.461	-73.369	-44.875	5987	-0.88986278	-2.430196039	-1.789014008
174067	4/16/2019 13:45	B	2643258.589	1216981.911	-73.365	-44.869	4230	-0.890397418	-2.252290615	-1.787886953
174067	4/16/2019 14:38	B	11505928.54	4158177.464	-73.346	-44.871	3208	-0.890794229	-2.155999983	-1.785343043

174067	4/16/2019 14:48	B	1288221.269	269743.7311	-73.344	-44.871	583	-0.890794229	-2.129492445	-1.785343043
174067	4/16/2019 15:51	B	3389987.451	3163117.549	-73.373	-44.858	3806	-0.890740691	-2.200692411	-1.790231487
174067	4/16/2019 16:29	B	30250044.69	57300067.31	-73.364	-44.857	2259	-0.89074233	-2.204728831	-1.791431747
174067	4/16/2019 16:50	B	1610639.467	2082173.033	-73.363	-44.856	1250	-0.890735756	-2.204728831	-1.7918565
174068	4/28/2019 2:16	B	15364215.13	3855969.873	-72.95	-42.892	4240	1.840394434	-0.311775821	1.309332458
174068	4/28/2019 11:59	B	52888654.89	1937003.105	-72.964	-42.926	34954	1.38608144	-0.210422604	0.82205005
174068	4/28/2019 21:42	B	75873839.35	8397841.653	-73.092	-43.075	34989	-0.13601194	0.191973602	0.59712392
174068	4/28/2019 23:24	B	20115281.47	4014760.533	-73.069	-43.091	6112	-0.420009953	0.210118527	0.506830432
174068	4/29/2019 1:18	B	31108.01968	9951698.48	-73.066	-43.088	6855	0.1390774	0.20124981	0.055951615
174068	4/29/2019 3:40	B	9874920.144	158750055.9	-73.156	-43.073	8519	0.328893247	0.20871078	0.883429623
174068	4/29/2019 9:50	B	5284500.5	3097560.5	-73.216	-43.069	22189	-0.127000517	0.22841484	1.2798918
174068	4/29/2019 10:16	B	48442023.3	1759359.198	-73.186	-43.02	1578	1.58494365	0.133111532	0.809308233
174068	4/29/2019 11:32	B	42029942.16	7137386.841	-73.203	-43.097	4558	0.16000509	0.269921617	1.16426204
174068	4/29/2019 12:35	B	217589843.7	9833982.76	-73.03	-43.015	3783	-0.212774664	0.043591034	0.047158511
174068	4/29/2019 14:20	B	7783820.278	863350.2218	-72.965	-42.985	6265	0.62139463	-0.028983948	0.214952625
174068	4/29/2019 22:43	B	20153707.99	2906358.513	-72.994	-42.961	30219	0.746650374	-0.08921747	-0.149650784

174068	4/30/2019 11:30	B	80009765.09	1335687.407	-72.948	-42.935	46003	0.666247075	-0.178390501	0.739162426
174068	4/30/2019 13:57	B	76599313.1	1807415.897	-72.876	-42.902	8837	-0.061755287	-0.285140381	1.416919509
174068	4/30/2019 22:35	B	12197309.96	5145515.039	-73.035	-42.845	31050	2.81657771	-0.454807267	0.965533244
174068	4/30/2019 23:49	B	2940975.717	914785.2834	-73.03	-42.847	4422	2.736580622	-0.436731281	0.962647061
174068	5/1/2019 2:55	B	10477971.72	2167514.776	-73.024	-42.862	11190	0.14668997	-0.39237104	0.713136721
174068	5/1/2019 3:57	B	6469106.18	1266384.32	-73.009	-42.88	3727	0.026510037	-0.348659457	0.620237047
174068	5/1/2019 4:36	B	6581925.665	1399636.835	-73.01	-42.886	2331	-0.02593168	-0.321696999	0.5643348
174068	5/1/2019 10:01	B	166242079.8	425751521.2	-73.074	-42.949	19475	-0.869124724	-0.081053814	0.173914272
174068	5/1/2019 13:54	B	6232770.347	2240349.653	-73.068	-42.968	14024	-1.041054069	-0.038617484	0.158270737
174068	5/1/2019 14:23	B	7460418.541	7888853.959	-73.052	-42.997	1694	-1.103072397	0.012790766	0.159896445
174068	5/1/2019 23:29	B	21397996.71	5184672.288	-73.044	-43.054	32791	-0.828093979	0.128081875	0.228373642
174068	5/2/2019 0:00	B	80020838.78	13361819.22	-73.039	-43.065	1866	-0.623593919	0.150030756	0.413696941
174068	5/2/2019 2:12	B	43549739.07	6527302.932	-73.009	-43.088	7916	-0.21445919	0.195010134	0.476938897
174068	5/2/2019 8:12	B	82280050.28	6612802.215	-72.833	-43.031	21597	0.056822744	0.070214026	0.763365702
174068	5/2/2019 12:00	B	12062620152	1127509452	-72.863	-42.989	13690	0.423593586	-0.023555027	0.511142622
174068	5/2/2019 13:19	B	68328468.56	2262559.442	-72.833	-42.981	4694	-0.175646865	-0.037404033	0.490039301

174068	5/2/2019 14:34	B	30158457.63	1987462.366	-72.884	-42.97	4506	0.800657019	-0.067396801	0.346461216
174068	5/2/2019 21:18	B	17204986.95	1613315.545	-72.921	-42.976	24252	0.737508532	-0.05640928	0.273676776
174068	5/2/2019 22:07	B	45990637.73	3816579.274	-72.899	-42.947	2960	1.704240265	-0.127222356	0.497834779
174068	5/3/2019 0:36	B	44280712.03	903400.4692	-72.852	-42.935	8929	3.740302974	-0.17432205	1.076290718
174068	5/3/2019 1:34	B	52633986.27	3928208.232	-72.914	-42.976	3476	2.28969717	-0.052589166	0.333483695
174068	5/3/2019 10:16	A	485580.7414	597061.7586	-73.136	-42.86	31335	1.157693324	-0.274771507	1.023216857
174068	5/3/2019 11:09	B	2906337.188	699136.8118	-73.135	-42.859	3183	1.157693324	-0.274771507	1.023216857
174068	5/3/2019 12:58	B	1440354.951	6561137.549	-73.061	-42.842	6542	2.516865786	-0.427703398	1.30801425
174068	5/3/2019 14:11	B	1424802.755	921367.7446	-73.048	-42.836	4392	3.020408125	-0.462407694	1.472968143
174068	5/3/2019 22:24	B	11332661337	3453439.542	-73.05	-42.907	29580	0.314297329	-0.227071874	1.118435441
174068	5/4/2019 1:53	B	49958470.68	1821809.818	-73.06	-42.909	12506	-0.253720596	-0.211970283	0.896317636
174068	5/4/2019 9:55	B	13388284.9	2288421.101	-73.092	-42.886	28923	-0.266345596	-0.250375723	0.874333425
174068	5/4/2019 13:51	B	106771530.4	369343.6478	-73.075	-42.996	14178	-0.411107807	0.024348134	0.828628912
174068	5/4/2019 21:50	B	68394882.5	3091342.499	-73.061	-43.089	28732	-0.592040518	0.205966785	0.69139737
174068	5/5/2019 1:41	B	7335717.341	1087035.659	-73.031	-43.132	13884	0.39781586	0.264092891	2.23242344
174068	5/5/2019 11:46	B	38791699.79	953118.2107	-73.029	-43.032	36249	0.392652419	0.080397849	1.400477914

174068	5/5/2019 12:18	B	294363960.5	935151.9549	-73.039	-43.057	1925	0.890098234	0.13061258	1.573850534
174068	5/5/2019 12:47	B	47863946.62	275243.881	-73.105	-43.051	1734	-0.00291952	0.13966528	1.191583199
174068	5/5/2019 13:21	B	158939284.7	443439.8496	-73.048	-43.074	2087	0.964298656	0.176493591	1.700842297
174068	5/5/2019 14:01	B	23436334.31	1027202.191	-73.132	-43.077	2359	-0.252166464	0.2094982	1.241974726
174068	5/6/2019 11:14	B	1645104247	42042365.03	-73.149	-43.033	76368	0.80999902	0.131749461	0.968290879
174068	5/6/2019 14:18	B	2897429388	110974376.2	-73.38	-43.061	11096	-0.477276531	0.272562809	-0.105204519
174068	5/6/2019 21:22	B	1322039842	7038196.346	-73.154	-42.957	25394	0.057021149	-0.008578556	0.675380047
174068	5/6/2019 22:33	B	3579592836	39829766.82	-73.14	-42.942	4257	0.676596912	-0.065174333	0.905263353
174068	5/6/2019 23:05	B	2224767978	1353919.182	-73.037	-42.958	1948	0.362706455	-0.074127403	1.226155399
174068	5/7/2019 0:39	B	344191016.8	2874681.713	-72.987	-42.971	5616	0.517591709	-0.05841608	1.562737707
174068	5/7/2019 1:51	B	206083274.2	7533209.793	-72.982	-42.987	4323	0.447214057	-0.021384263	1.656778828
174068	5/7/2019 10:44	B	136804801.8	18034688.24	-73.112	-42.908	31993	0.405796721	-0.165964395	0.821869643
174068	5/7/2019 11:39	B	421814990.1	835596.3869	-73.023	-42.899	3288	-0.313028196	-0.271491028	1.144630439
174068	5/7/2019 22:02	B	314829795.8	4015809.177	-72.999	-42.872	37388	0.268050031	-0.384805178	1.342400844
174068	5/8/2019 11:33	B	102014022.6	2591839.856	-73.016	-42.862	48636	-0.142917027	-0.403506685	1.190694363
174068	5/8/2019 23:43	B	3014101399	23876060.87	-72.701	-42.887	43834	0	-0.247630695	0

174068	5/9/2019 2:52	B	241307204.2	4312090.288	-72.85	-42.864	11363	-0.907518124	-0.426819906	1.37099783
174068	5/9/2019 11:18	B	119139366	4659027.038	-72.733	-42.86	30340	0	-0.362221451	0
174068	5/10/2019 8:18	B	190760306.9	26374347.61	-73.129	-42.991	75586	-1.202828382	0.044670578	0.159367524
174068	5/10/2019 9:53	B	169390935.4	26160419.14	-73.158	-42.998	5718	-1.225745637	0.074956035	0.154509878
174068	5/10/2019 12:55	1	2853660.5	112338	-73.147	-43.077	10934	-1.173396884	0.215890933	0.167354075
174068	5/10/2019 14:33	B	248131573.8	37137520.66	-73.15	-43.093	5866	-1.157350662	0.242424917	0.173716136
174068	5/11/2019 2:15	B	1477363782	300530890.3	-73.17	-43.197	42081	-1.176083888	0.391742785	0.641045974
174068	5/11/2019 3:48	B	2599339983	2776957541	-73.151	-43.201	5627	-1.163142683	0.390813271	0.63924542
174068	5/11/2019 10:16	B	314787317.3	26026125.18	-72.905	-43.037	23271	-0.761767918	0.07806766	1.092454515
174068	5/11/2019 11:55	B	41969588.29	11876412.21	-72.948	-43.017	5939	-0.670354982	0.041731249	1.00451029
174068	5/11/2019 13:37	B	3405145.678	437916.8222	-72.988	-43.046	6090	-0.746630794	0.099128362	0.857306825
174068	5/11/2019 14:14	B	4853602.74	448317.2598	-72.987	-43.051	2226	-0.758415952	0.10787502	0.865589863
174068	5/11/2019 21:38	B	8305212925	10128597.25	-72.972	-43.06	26639	-0.792822614	0.123683584	0.910945191
174068	5/11/2019 22:03	B	340794932.4	2614110.146	-72.961	-43.07	1535	-0.821952598	0.158294109	0.926447533
174068	5/12/2019 0:10	B	36468795.95	840900.0527	-72.95	-43.089	7573	-0.08340053	0.19014032	0.645371702
174068	5/12/2019 1:56	B	16132663.76	829661.2403	-72.957	-43.075	6384	-0.306081165	0.165347949	0.579008385

174068	5/12/2019 2:25	B	13865166.52	1077529.983	-72.962	-43.078	1750	-0.251148231	0.171253672	0.590716467
174068	5/12/2019 3:04	B	39311218.77	2283762.229	-72.981	-43.049	2313	-0.873948127	0.104441877	0.477756862
174068	5/12/2019 3:36	B	18507223.28	535017.7198	-73	-43.049	1946	-0.87893495	0.108879613	0.459982362
174068	5/12/2019 9:39	B	11246362.23	2308600.265	-73.105	-43	21771	0.064869728	0.051267137	0.383166584
174068	5/12/2019 9:53	B	17842007.97	1344877.035	-73.092	-42.99	824	0.109112639	0.021449562	0.313932561
174068	5/12/2019 22:45	B	129598280.4	1813712.134	-73.074	-43.019	46313	-0.730605119	0.07040948	0.43990931
174068	5/12/2019 23:45	B	1660716391	2126303.975	-72.998	-43.043	3602	-0.937694205	0.094334798	0.454305655
174068	5/13/2019 1:22	B	142681003.3	1506619.216	-73.004	-43.029	5846	0.405779628	0.071243069	-0.30132241
174068	5/13/2019 2:06	B	5828198.758	1061662.242	-73.014	-43.032	2643	0.556234976	0.076959323	-0.238501025
174068	5/13/2019 2:43	B	6354549.671	1346630.829	-72.958	-43.073	2230	2.605184595	0.162721594	-0.203632581
174068	5/13/2019 3:26	B	5065168.157	870972.8427	-72.955	-43.1	2555	3.136251564	0.209701006	0.246233845
174068	5/13/2019 3:46	B	4892192	1190424.5	-72.962	-43.095	1207	3.138411311	0.199877106	0.247717985
174068	5/13/2019 7:43	B	13109761.83	1297068.667	-72.875	-43.191	14224	0.488272642	0.354714521	0.360352156
174068	5/13/2019 9:29	B	68959715.72	40061984.78	-72.88	-43.194	6339	0.365484313	0.359371466	0.360087741
174068	5/14/2019 3:25	Z	17567869.2	50175612.8	-73.039	-43.129	64586	0.204903936	0.260240963	0.404930644
174068	5/14/2019 13:08	B	103312017.1	2041147.905	-73.023	-43.085	34987	1.181474525	0.191303713	-0.068087427

174068	5/15/2019 2:29	B	96183868.15	9133730.351	-73.025	-43.155	48059	-0.622162858	0.31088047	0.254485063
174068	5/15/2019 3:02	B	80102069.39	113362524.6	-73.112	-43.119	1993	0.130646839	0.267248072	0.025435403
174068	5/15/2019 12:49	B	1165672328	2152812.5	-73.255	-43.213	35183	-0.038387657	0.369757328	0.359968089
174068	5/16/2019 1:09	B	524634812.7	456214648.3	-73.267	-43.227	44418	-0.391655814	0.354890072	-0.491953365
174068	5/16/2019 3:49	B	8073862.146	4680110.354	-73.344	-43.232	9582	-0.316836634	0.274073578	-0.503497137
174068	5/16/2019 20:42	B	45624578.62	10034757.88	-73.111	-43.112	60793	-0.538989729	0.255974629	-0.72361803
174068	5/16/2019 21:09	B	476725323.6	233736806.9	-73.222	-43.126	1619	-0.552868126	0.315534777	-0.902135157
174068	5/16/2019 22:18	B	5926500.932	1291085.568	-73.219	-43.148	4124	-0.483213361	0.348838873	-0.805612011
174068	5/17/2019 0:55	B	78153018.77	119179321.2	-73.208	-43.158	9460	-0.35852335	0.361114795	-1.428187028
174068	5/17/2019 2:23	A	480723360.9	5312465.603	-73.166	-43.1	5275	-0.515381278	0.258098954	-1.734286251
174068	5/17/2019 2:40	B	22772007.11	772151.3874	-73.156	-43.102	1022	-0.487019196	0.254918921	-1.735638145
174068	5/17/2019 4:08	B	12461359.77	1279293.233	-73.147	-43.077	5254	-0.582527605	0.215890933	-1.831996876
174068	5/17/2019 11:03	B	40903376.43	2574529.565	-73.129	-43.101	24900	-0.450851363	0.245030976	-1.746493716
174068	5/17/2019 13:06	B	14010468.3	5567139.702	-73.119	-43.154	7380	-0.383992473	0.324756678	-1.475901988
174068	5/17/2019 14:07	B	11253594.35	2936604.154	-73.272	-43.278	3678	-0.395844921	0.326225049	-0.743634262
174068	5/17/2019 23:26	B	2402358257	39019303.86	-73.213	-43.607	33556	-0.111274542	-0.054675972	0.261117465

174068	5/18/2019 0:16	B	642030506.4	10897481.61	-73.192	-43.661	2983	0.223824178	-0.203424973	-0.084785958
174068	5/18/2019 10:39	B	33372019516	5447589191	-73.347	-43.512	37366	-0.292075563	0.162964118	-1.024688943
174068	5/18/2019 11:36	B	156946041.4	20158521.13	-73.455	-43.503	3409	0.231473199	0.069756103	-0.782512173
174068	5/18/2019 12:16	B	227623226.8	19533257.17	-73.519	-43.483	2441	0.590087969	-0.048902369	-0.135971669
174068	5/18/2019 13:15	B	68246244.5	72962	-73.531	-43.525	3536	0.139515421	-0.038516155	-0.141699683
174068	5/19/2019 8:14	B	21154454491	318940148.7	-73.15	-43.181	68338	-0.114617965	0.370359747	-0.241920655
174068	5/19/2019 11:56	B	153488285.2	4729115.803	-73.07	-43.226	13312	-0.009505104	0.408656256	-0.150638764
174068	5/20/2019 10:37	A	615070.4172	13097.58281	-73.303	-43.149	81651	0.54684224	0.347851346	-0.01518252
174068	5/20/2019 11:28	A	2817079.85	1370.149897	-73.33	-43.158	3049	-0.073889604	0.336598746	0.03316144
174068	5/20/2019 11:51	A	383210.2638	773605.7362	-73.299	-43.135	1396	0.813988689	0.338680342	-0.177466332
174068	5/20/2019 12:06	A	100911.1699	1098898.83	-73.288	-43.145	887	0.851586214	0.348225354	-0.127248687
174068	5/21/2019 3:15	B	216387404.4	57450508.14	-73.242	-43.123	54557	0.593748176	0.322028297	-1.12745829
174068	5/21/2019 11:02	B	224672760.9	20823959.65	-73.22	-43.032	28032	-0.142863691	0.168399998	-0.440865442
174068	5/21/2019 11:44	B	1448253.691	87094138.81	-73.175	-42.989	2514	-0.719839762	0.06903935	-0.277137949
174068	5/21/2019 21:24	B	13660584.47	1253898.026	-72.847	-42.941	34784	2.214397063	-0.147135831	-0.133030769
174068	5/22/2019 12:07	B	20709174.73	6393133.771	-72.854	-42.945	53004	-0.121998836	-0.134882234	0.04660307

174068	5/23/2019 0:32	B	578350615.6	1860585.449	-72.465	-42.827	44661	0	0.022622222	0
174068	5/23/2019 12:22	B	5991382057	1429491708	-72.873	-42.924	42589	-0.724456314	-0.215411747	0.139407343
174068	5/23/2019 22:03	B	770261246.4	8025946.072	-72.999	-43.002	34909	-1.058895427	0.010363105	-0.022978798
174068	5/24/2019 3:22	B	3383063576	1504942.893	-72.995	-43.062	19111	-0.871578703	0.139073337	-1.15711107
174068	5/24/2019 13:38	B	2494107429	19827855.84	-72.907	-43.054	36992	-0.899301454	0.115071824	-1.169095395
174068	5/24/2019 14:00	B	2432064594	36984258.82	-72.915	-43.033	1277	-0.874957288	0.069631163	-1.126699172
174068	5/24/2019 21:30	B	107827829.5	6882729.04	-73.118	-43.206	27001	-1.112810848	0.391388517	-1.352320024
174068	5/24/2019 23:10	B	48071298.93	133138435.6	-73.204	-43.183	5989	-1.120969461	0.386329289	-1.310525776
174068	5/24/2019 23:42	B	775443.4024	520302.5976	-73.233	-43.212	1924	-1.126783044	0.383912827	-1.312974152
174068	5/25/2019 1:11	B	1958811.939	924308.5612	-73.281	-43.233	5342	-1.094441338	0.343282661	-1.393517737
174068	5/25/2019 2:56	B	3558761.489	1374127.511	-73.336	-43.256	6345	-1.077545352	0.270288215	-1.373767678
174068	5/25/2019 12:03	B	31168657.88	2686770.619	-73.238	-43.177	32789	-1.041108121	0.378636787	-1.349487149
174068	5/25/2019 12:38	B	15906699.68	3632558.816	-73.249	-43.21	2094	-1.073872031	0.372969893	-1.385671426
174068	5/25/2019 13:19	B	32060985.01	2545367.488	-73.172	-43.176	2488	-0.966230155	0.370856303	-1.342713868
174068	5/26/2019 3:35	Z	13205975.09	655906.9113	-73.01	-43.094	51330	-1.160266881	0.205803368	-1.082144265
174068	5/27/2019 22:16	B	3322471551	310682409.6	-72.712	-42.958	153699	0	-0.046644782	-0.435678438

174068	5/28/2019 15:05	B	574251940.3	114998532.7	-72.728	-42.953	60531	-0.1931538	-0.076356809	1.327405269
174068	5/29/2019 12:21	B	2.4715E+11	1.21176E+11	-72.929	-43.033	76535	3.266132898	0.066230417	0.818533463
174068	5/29/2019 21:58	B	7316093.418	483866020.6	-73.041	-43.058	34626	0.628638206	0.130020648	0.165351634
174068	5/29/2019 22:14	B	358727.2882	337989.2118	-73.039	-43.057	955	0.628638206	0.13061258	0.165351634
174068	5/30/2019 1:20	B	7211343.175	1323467.325	-73.015	-43.037	11146	0.559067081	0.089030823	0.384901034
174068	5/30/2019 1:47	B	5958619.957	1164105.043	-73.028	-43.045	1638	0.052501324	0.107252407	0.310320576
174068	5/30/2019 2:19	B	7962899.484	1729771.016	-73.02	-43.039	1898	0.368223469	0.091271232	0.375852375
174068	5/30/2019 9:23	B	18379661.5	1481588.501	-72.947	-42.973	25453	0.294457806	-0.059220903	1.354558018
174068	5/30/2019 10:25	B	97183022.78	350789.2227	-72.94	-42.904	3741	-0.667395605	-0.276190348	1.731962016
174068	5/30/2019 11:57	B	86855781.89	505732.1073	-72.944	-42.85	5503	-0.663829695	-0.48214857	1.80723962
174068	5/30/2019 12:17	B	21323604.81	977295.1907	-73.016	-42.862	1184	0.278628338	-0.403506685	1.716841205
174068	5/30/2019 21:47	B	551295999.2	446952250.8	-72.989	-42.869	34242	-0.199008535	-0.397816767	1.773984752
174068	5/31/2019 0:33	B	43264890.5	14425590.5	-72.971	-42.908	9964	-0.476450884	-0.265496283	0.284982475
174068	5/31/2019 1:54	B	25485203.7	3959714.801	-72.989	-42.906	4834	-0.452876097	-0.265117212	0.261890277
174068	5/31/2019 9:17	B	22775615.78	1673920.216	-72.971	-42.819	26563	-1.034969517	-0.634868387	0.428552021
174068	5/31/2019 11:56	B	16164781.04	23403284.96	-72.971	-42.83	9586	-1.056129017	-0.578008976	0.434126901

174068	5/31/2019 13:58	B	4787092.891	13748453.11	-73.002	-42.825	7282	-0.988421147	-0.582079893	0.404462024
174068	5/31/2019 21:37	B	38328281.96	1867370.539	-72.993	-42.826	27522	-1.008146826	-0.584177181	0.413339376
174068	5/31/2019 23:17	B	14291799.45	2742549.046	-73.065	-42.834	6005	-0.781342	-0.450652981	0.280860696
174068	6/1/2019 8:57	B	34457573.23	2667407.772	-73.181	-42.858	34822	-1.135662582	-0.258589272	-1.551639115
174068	6/1/2019 12:00	B	40878361.27	8651077.228	-73.204	-42.859	11006	-1.124849047	-0.239849165	-1.539061294
174068	6/1/2019 13:38	1	10499150.19	866356.3131	-73.171	-42.914	5828	-1.111722115	-0.112721357	-1.575872472
174068	6/1/2019 21:19	B	65830450.83	15058845.17	-73.175	-42.95	27715	-1.093228086	-0.008282655	-1.581324085
174068	6/1/2019 23:39	B	53858768.08	22194209.92	-73.184	-42.967	8400	-1.078295944	0.032744229	-1.575328515
174068	6/2/2019 1:53	B	71792365.29	9795335.714	-73.188	-42.94	8042	-1.112973411	-0.033284236	-1.360099874
174068	6/2/2019 2:51	B	8599098.887	3758161.613	-73.238	-42.957	3481	-1.042012961	0.035279054	-1.312853054
174068	6/3/2019 2:36	B	52690379.29	2603077.715	-73.253	-42.933	85444	-0.609976324	-0.026583557	-0.624974159
174068	6/3/2019 12:26	0	357853361.4	1875202.585	-73.35	-42.892	35419	-0.445290752	-0.027031199	-0.921634896
174068	6/3/2019 14:38	B	249273604.3	18043328.65	-73.471	-42.869	7910	-0.6530974	0.08132624	-0.926589019
174068	6/3/2019 21:17	B	7155761498	19819978202	-73.218	-42.854	23984	-0.008702941	-0.236101297	-0.959497886
174068	6/4/2019 0:20	B	9655244.384	3512428.616	-73.309	-42.86	10966	0.905476939	-0.126766679	-1.047199213
174068	6/4/2019 2:26	B	139080617.1	9441856.907	-73.346	-42.859	7558	0.563763622	-0.096306072	-1.11477816

174068	6/4/2019 8:32	B	81719639.35	4323005.648	-73.443	-42.851	21937	-0.138545928	0.033624462	-1.097674357
174068	6/4/2019 13:34	B	503036641.9	3184590.641	-73.382	-42.853	18141	0.202338756	-0.056192742	-1.17307129
174068	6/4/2019 13:57	B	741626122.9	15620049.59	-73.437	-42.838	1343	-0.109947621	0.003533104	-1.199955068
174068	6/5/2019 2:23	B	3465672666	16964265.95	-73.298	-42.881	44762	0.968552919	-0.101434589	-0.708654944
174068	6/5/2019 8:10	B	1013684447	4503649.481	-73.362	-42.882	20864	0.489298267	-0.033101815	-0.866838414
174068	6/5/2019 12:14	B	33977521.5	12497715.5	-73.224	-42.891	14596	0.676905034	-0.137481074	-0.344167151
174068	6/5/2019 14:33	B	44221074.44	27413226.56	-73.176	-42.901	8371	0.372163404	-0.149281435	-0.131766734
174068	6/6/2019 2:04	B	44591701.57	10421214.93	-73.072	-42.944	41466	-0.793281329	-0.102126966	0.069384496
174068	6/6/2019 2:41	B	78149438.12	5160658.379	-73.123	-42.935	2230	-0.578079384	-0.096068597	0.034038272
174068	6/6/2019 9:42	B	55850771.4	14714197.1	-73.085	-42.916	25213	-0.484135756	-0.177772278	-0.017387973
174068	6/7/2019 21:51	A	1917829.829	207215.1712	-73.029	-43.101	130166	-0.992174307	0.218443192	-0.546146436
174068	6/8/2019 2:29	B	45862815.03	2061061.971	-72.963	-43.203	16707	-1.065246172	0.370530694	-1.523535624
174068	6/8/2019 10:15	B	29459453.07	3925379.931	-73.048	-43.362	27953	0	0.329270433	-1.281318223
174068	6/8/2019 11:51	B	57241557.58	17721274.92	-73.053	-43.377	5754	0	0.305925244	-1.252020301
174068	6/8/2019 12:15	B	32017976.28	9771024.222	-73.093	-43.352	1428	-1.015299029	0.355706146	-1.26150969
174068	6/8/2019 12:57	B	7514636202	1787713044	-73.104	-43.412	2545	-0.760693213	0.268455616	-1.154340655

174068	6/8/2019 13:16	B	74287051.56	34917473.44	-73.131	-43.419	1100	-0.669360621	0.261593718	-1.105009873
174068	6/9/2019 0:07	B	106971524.9	1005308.052	-73.538	-43.273	39082	-0.506906441	-0.00763119	-0.105026267
174068	6/9/2019 2:51	B	45846743.14	1162729.856	-73.095	-43.191	9817	-0.253420462	0.370989365	-1.888500097
174068	6/9/2019 14:13	B	52099577.39	2194922.611	-73.071	-43.138	40941	-0.098303998	0.27921467	-1.97740088
174068	6/10/2019 13:12	B	96668037.88	4718942.118	-73.102	-43.359	82706	-0.411896175	0.343980799	-2.261790167
174068	6/10/2019 13:54	B	86257468.56	3145221.443	-73.077	-43.367	2520	-0.362758367	0.326622758	-2.237280227
174068	6/11/2019 1:28	B	106317362	1039682	-73.058	-43.228	41648	0.454321836	0.407609013	-2.938853901
174068	6/11/2019 8:51	B	132988707.1	3375942.904	-72.971	-43.134	26585	0.197435753	0.263035133	-2.324273104
174068	6/14/2019 22:16	B	9447433.38	238071.62	-73.253	-43.214	307511	0.142619074	0.370759788	-1.009276449
174068	6/15/2019 2:28	B	2190516516	10713749.38	-73.274	-43.308	15102	-0.601138959	0.312022383	-0.27526644
174068	6/15/2019 12:17	B	109969381.7	4888848.805	-73.303	-43.456	35379	0.992672503	0.241480134	0.348290545
174068	6/15/2019 13:08	B	167010764.4	2441640.586	-73.444	-43.544	3048	0.852992446	0.11049006	0.89275538
174068	6/15/2019 13:51	B	266310224.6	6649449.872	-73.481	-43.567	2555	3.201048937	0.087623732	0.143718522
174068	6/16/2019 0:34	B	479926990.9	24158163.06	-74.012	-43.902	38579	0.49475156	0.281769787	-0.513103205
174068	6/16/2019 2:36	B	1269124731	208594909.6	-73.999	-43.907	7369	0.363747882	0.270746137	-0.473709419
174070	2/9/2019 22:12	B	12057656.49	2663976.009	-73.509	-44.19	3306	-0.109446467	-2.104007309	-0.065340051

174070	2/9/2019 22:19	0	39801083630	1237485595	-73.506	-44.202	445	-0.73822135	0.26294687	-0.626530019
174070	2/9/2019 23:02	A	119401338.6	54344866.45	-73.571	-44.189	2534	-0.040940241	-1.175543905	0.339317266
174070	2/9/2019 23:59	B	15448543.72	3418140.781	-73.573	-44.191	3448	-0.024209728	-1.243228658	-0.039337507
174070	2/10/2019 0:36	B	14117758.02	428006.9821	-73.567	-44.188	2221	0.337332509	-1.244218079	0.603287246
174070	2/10/2019 1:17	A	132770.9738	18949.02623	-73.542	-44.205	2443	0.030270673	-1.773027419	0.683200699
174070	2/10/2019 1:34	A	1018426.952	2911435.548	-73.544	-44.215	1001	0.390142089	-1.976863283	0.655482948
174070	2/10/2019 2:09	1	3663982.544	518425.9556	-73.531	-44.222	2103	0.63290476	-2.243900221	0.658836576
174070	2/10/2019 3:00	B	31417788.8	935019.6991	-73.508	-44.235	3115	0.909348978	-2.598834425	0.648275469
174070	2/10/2019 5:29	B	68862396.57	148304647.4	-73.389	-44.229	8927	0.000881218	-2.866475189	0.77851576
174070	2/10/2019 7:03	B	82694189.93	30522759.07	-73.382	-44.214	5653	0.119538347	-2.866475189	0.841381979
174070	2/10/2019 8:48	0	2188355659	136053465	-73.505	-44.21	6263	-0.314263182	-2.465076922	0.802218852
174070	2/10/2019 9:32	B	47913603.7	6746902.299	-73.513	-44.213	2662	-0.021160969	-2.294792458	0.737438118
174070	2/10/2019 10:27	A	254014.7155	384415.7845	-73.572	-44.23	3259	1.221920229	-1.988568546	0.47249898
174070	2/10/2019 11:03	B	1598793.515	416396.9853	-73.581	-44.23	2186	1.136709722	-1.907931034	0.435366966
174070	2/10/2019 11:14	B	1610568.717	154456.2825	-73.577	-44.227	682	1.073861501	-1.885358161	0.493128593
174070	2/10/2019 12:05	B	28114246.22	2027222.785	-73.601	-44.215	3009	0.725775127	-1.473590342	0.427854007

174070	2/10/2019 12:47	0	4122941870	45856678.75	-73.595	-44.205	2565	0.654743295	-1.427316087	0.479935952
174070	2/10/2019 12:54	B	221261055.1	5905151.403	-73.585	-44.203	426	0.605437862	-1.379325151	0.497837082
174070	2/10/2019 13:33	B	64372229.44	2920731.058	-73.591	-44.195	2339	0.634648082	-1.280852475	0.503825905
174070	2/10/2019 14:39	A	12391.37802	29610.62198	-73.551	-44.201	3947	0.121619222	-1.610554909	0.661889352
174070	2/10/2019 15:13	B	5006624.804	562877.6961	-73.534	-44.226	2052	0.769457104	-2.375565853	0.64134191
174070	2/10/2019 20:09	B	8571696.944	2248303.056	-73.514	-44.313	17723	0.09884427	-1.554410018	0.325766783
174070	2/10/2019 21:06	B	804144.0656	9025368.434	-73.568	-44.334	3410	-0.465290515	-1.133590444	0.163359741
174070	2/10/2019 21:44	A	29185255.38	1558997.616	-73.543	-44.364	2302	-0.17373083	-1.476564315	0.25793761
174070	2/10/2019 22:42	A	108419.1167	1226233.383	-73.558	-44.337	3456	-0.288707373	-1.231584573	0.19241235
174070	2/10/2019 23:21	B	25459280.64	1412024.36	-73.565	-44.356	2390	-0.469620499	-1.188547765	0.195578343
174070	2/10/2019 23:31	1	2871274.365	282438.1353	-73.559	-44.36	570	-0.419750147	-1.27629204	0.203510883
174070	2/11/2019 0:25	B	2049410.211	215734.7891	-73.559	-44.378	3221	-0.446183855	-1.334357227	0.244647518
174070	2/11/2019 1:01	2	89951.24774	48649.25226	-73.546	-44.338	2184	-0.400451262	-1.355139975	0.292282783
174070	2/11/2019 1:13	2	286434.9386	16603.56142	-73.529	-44.335	720	-0.104374331	-1.445320548	0.311545036
174070	2/11/2019 2:26	A	27137524.53	2235823.973	-73.55	-44.316	4365	-0.134415119	-1.274159131	0.259239779
174070	2/11/2019 2:59	B	5803824.5	65884.5	-73.549	-44.327	1987	-0.320707137	-1.272283817	0.283958703

174070	2/11/2019 3:25	A	205277.6533	5431.346707	-73.552	-44.321	1598	-0.28059539	-1.210732228	0.271387252
174070	2/11/2019 4:22	B	1014553.272	279635.7277	-73.552	-44.319	3409	-0.233881563	-1.207727648	0.265149238
174070	2/11/2019 5:13	B	45286.47487	18206548.03	-73.563	-44.326	3056	-0.464723378	-1.136864733	0.280585252
174070	2/11/2019 8:40	B	2770957971	69511558.53	-73.582	-44.315	12405	-0.416966659	-1.119250482	0.281468933
174070	2/11/2019 9:09	B	445775.4125	398072.5875	-73.581	-44.315	1733	-0.416966659	-1.114578174	0.281468933
174070	2/11/2019 10:14	B	2751188.662	665289.8383	-73.567	-44.326	3894	-0.549464029	-1.11988828	0.284143781
174070	2/11/2019 10:46	B	3362404.425	574685.5747	-73.54	-44.316	1927	0.03547416	-1.288387381	0.267433684
174070	2/11/2019 10:56	B	3883762.683	670899.8168	-73.546	-44.318	582	-0.11294851	-1.274029768	0.268954836
174070	2/11/2019 11:35	1	10955654.07	590753.9339	-73.584	-44.284	2394	0.416259624	-1.375080298	0.269920817
174070	2/11/2019 11:37	B	1342999.224	103153.2756	-73.585	-44.284	81	0.416259624	-1.375080298	0.269920817
174070	2/11/2019 12:25	0	6009724.5	17177244.5	-73.588	-44.285	2895	0.269093195	-1.362118418	0.298063932
174070	2/11/2019 12:33	A	10148947.94	75650.56049	-73.616	-44.277	491	-0.036380365	-1.275853861	0.37412427
174070	2/11/2019 12:41	B	1772261.043	29831.45674	-73.611	-44.276	449	-0.045463414	-1.291707715	0.375939114
174070	2/11/2019 13:10	0	424431.9881	3091924.512	-73.568	-44.263	1780	1.590088384	-1.68980699	0.36264891
174070	2/11/2019 13:50	B	368317230.4	13916882.08	-73.629	-44.249	2405	0.561675465	-1.482064466	0.482071128
174070	2/11/2019 14:20	B	81541149.65	2297827.349	-73.629	-44.249	1757	0.241621907	-1.308389429	0.439257126

174070	2/11/2019 14:58	B	5113439.266	321673.2339	-73.643	-44.241	2322	0.228822088	-1.238976806	0.39621969
174070	2/11/2019 20:53	B	855860881.4	186102416.6	-73.598	-44.258	21303	1.680709508	-1.661626358	0.349542546
174070	2/11/2019 21:15	B	1044027.36	1707558.64	-73.543	-44.284	1299	1.06912722	-1.507247968	0.250480892
174070	2/11/2019 22:30	A	5638832138	43168751.65	-73.604	-44.287	4476	-0.048422698	-1.241903691	0.330087866
174070	2/11/2019 23:00	B	89895403.16	4686545.345	-73.626	-44.279	1795	-0.328066376	-1.205692101	0.37697666
174070	2/11/2019 23:06	B	74372301.52	1117047.476	-73.618	-44.292	381	-0.37886204	-1.131903945	0.347746295
174070	2/12/2019 0:13	0	41966195.06	231587.4431	-73.539	-44.288	4021	0.421375529	-1.50042749	0.663827043
174070	2/12/2019 0:37	B	4194653.549	1955448.951	-73.516	-44.296	1456	0.591522413	-1.621674289	0.583641735
174070	2/12/2019 0:59	B	4617916.432	1489997.568	-73.514	-44.295	1304	0.472006789	-1.621674289	0.577266947
174070	2/12/2019 1:27	A	3051679.985	40506132.51	-73.523	-44.342	1671	1.068447827	-1.627866898	0.293558569
174070	2/12/2019 2:22	A	2635803.701	18112800.8	-73.459	-44.337	3322	0.536015695	-2.759259742	0.475544011
174070	2/12/2019 2:38	B	28215592.31	12018872.19	-73.523	-44.343	929	0.941725588	-1.746963064	0.264920457
174070	2/12/2019 3:09	0	3437302163	10024563.58	-73.486	-44.332	1866	1.273480425	-1.357707411	0.360111041
174070	2/12/2019 4:10	0	63083745.31	9613003.69	-73.547	-44.311	3672	1.242072277	-1.277500467	0.55450969
174070	2/12/2019 6:44	B	506882874.1	138520486.4	-73.578	-44.282	9241	0.064306022	-1.423362151	0.701599695
174070	2/12/2019 8:26	3	99680.35407	3623.645927	-73.532	-44.316	6091	1.384611694	-1.337655293	0.49035102

174070	2/12/2019 8:46	B	402211.986	98660.51403	-73.53	-44.315	1229	1.384611694	-1.358451678	0.49035102
174070	2/12/2019 9:24	B	1145823.538	245274.4623	-73.525	-44.311	2261	1.223030473	-1.433039628	0.500319433
174070	2/12/2019 10:03	A	145484.459	13692.041	-73.556	-44.278	2332	0.030955782	-1.50524822	0.69339751
174070	2/12/2019 10:43	B	2855452.271	407988.2289	-73.515	-44.287	2437	0.214636653	-1.688068288	0.621103069
174070	2/12/2019 11:06	A	54524.95204	1909887.548	-73.522	-44.317	1343	1.262581199	-1.487309793	0.469292883
174070	2/12/2019 11:17	B	379014.5903	622297.9097	-73.519	-44.313	672	1.262581199	-1.519477357	0.469292883
174070	2/12/2019 12:00	B	953001.5497	478820.9503	-73.514	-44.307	2613	0.904913916	-1.55528029	0.516653768
174070	2/12/2019 12:13	B	6053143.04	37057271.46	-73.515	-44.308	772	0.952717457	-1.535689767	0.522146363
174070	2/12/2019 12:54	0	20202110.11	1778568.394	-73.535	-44.296	2464	0.64548351	-1.435256163	0.621574722
174070	2/12/2019 13:55	0	12775545.12	5504787.385	-73.485	-44.304	3637	0.183705609	-2.05731341	0.51014103
174070	2/12/2019 14:00	A	8425237.274	1151881.226	-73.483	-44.308	311	0.196110817	-2.107053184	0.498251626
174070	2/12/2019 14:33	B	11589137.86	1828935.138	-73.469	-44.312	1977	0.022750092	-2.411930002	0.501051511
174070	2/12/2019 15:41	A	6080653.308	3514476.692	-73.452	-44.311	4077	-0.374560238	-2.622862712	0.546894054
174070	2/12/2019 20:48	B	9489033.5	6065811.5	-73.393	-44.315	18403	-0.628074259	-2.866475189	0.670294171
174070	2/12/2019 22:21	A	18798171.09	52241.41067	-73.553	-44.285	5596	0.284673403	-1.454633292	0.691617547
174070	2/12/2019 22:24	B	1993583.199	12500.80065	-73.553	-44.285	202	0.284673403	-1.454633292	0.691617547

174070	2/13/2019 0:07	B	634835.3187	8690797.181	-73.44	-44.29	6187	-0.015578591	-2.757725891	0.222454229
174070	2/13/2019 0:40	B	54500678.63	26725678.37	-73.455	-44.313	1946	-0.373283878	-2.629274158	0.217248626
174070	2/13/2019 1:08	A	62692.66119	2473677.339	-73.439	-44.313	1671	-0.264715229	-2.733221705	0.191945722
174070	2/13/2019 1:56	B	809523.1646	468201.3354	-73.455	-44.317	2876	-0.402172913	-2.603384978	0.232672735
174070	2/13/2019 2:10	B	921886.8394	553739.6606	-73.469	-44.317	888	-0.518457871	-2.41401626	0.264900529
174070	2/13/2019 2:44	B	94784532.67	22870717.33	-73.473	-44.268	1998	0.562817206	-2.363889758	0.391745977
174070	2/13/2019 4:00	A	95940425.99	77928696.51	-73.571	-44.291	4603	0.145185826	-1.362738269	0.562463202
174070	2/13/2019 4:25	B	9922998.143	5466430.357	-73.585	-44.295	1462	0.028861398	-1.267077429	0.546531608
174070	2/13/2019 6:34	1	857777.6444	348114.8556	-73.649	-44.24	7733	-0.292164194	-1.188968781	0.52628203
174070	2/13/2019 8:05	B	2635477.133	697487.3675	-73.7	-44.241	5457	0.099510061	-0.823105663	0.449346699
174070	2/13/2019 9:51	B	22310516.82	123797733.2	-73.637	-44.244	6396	-0.054363221	-1.312297655	0.579081615
174070	2/13/2019 10:00	B	1431677.178	6314651.822	-73.634	-44.233	498	-0.047163034	-1.321188562	0.582181611
174070	2/13/2019 10:32	B	1034271.978	1952436.022	-73.642	-44.234	1933	-0.181435174	-1.235852514	0.560110598
174070	2/13/2019 11:40	B	4162569.005	242374.9955	-73.659	-44.201	4106	0.338518482	-0.946624231	0.461119597
174070	2/13/2019 11:56	B	6379196.239	202229.7613	-73.649	-44.202	956	0.434947157	-0.995608576	0.485470007
174070	2/13/2019 12:33	B	22818873.01	20204886.99	-73.593	-44.178	2228	1.473881334	-1.087410819	0.663335974

174070	2/13/2019 13:21	B	41660709.55	14387906.45	-73.595	-44.177	2843	1.42619814	-1.101968703	0.678954294
174070	2/13/2019 13:43	B	14474252.75	2932619.746	-73.566	-44.181	1344	0.469381512	-1.18395432	0.818032176
174070	2/13/2019 13:50	B	15358296.78	2886651.216	-73.576	-44.175	426	0.888212207	-1.13649435	0.773984622
174070	2/13/2019 14:11	B	19572142.26	217398.7418	-73.561	-44.172	1261	0.388215903	-1.156219683	0.830165215
174070	2/13/2019 15:14	A	6567276.747	47785783.75	-73.619	-44.263	3761	0.142608378	-1.334838617	0.572069092
174070	2/13/2019 15:57	B	55014703.34	26994979.16	-73.633	-44.281	2611	-0.022106324	-1.160914076	0.473380474
174070	2/13/2019 20:26	0	106361247.3	640055573.2	-73.45	-44.298	16135	-0.38720342	-2.62784881	0.224340127
174070	2/13/2019 21:53	A	1819527904	14062350.19	-73.455	-44.363	5221	0.070806662	-2.866475189	0.362040918
174070	2/13/2019 22:13	B	2075357.351	99187.14897	-73.455	-44.377	1196	0.228467645	-2.866475189	0.424291012
174070	2/13/2019 23:32	B	3194888.058	256604.9424	-73.456	-44.409	4745	0.167027363	-2.866475189	0.566020202
174070	2/13/2019 23:53	B	4017694.279	1242057.721	-73.49	-44.399	1234	-0.038405278	-2.613848615	0.469495466
174070	2/14/2019 0:25	A	10547.44601	121801.554	-73.493	-44.406	1955	0.710251946	-2.624953079	1.257338589
174070	2/14/2019 0:48	B	423200	137288	-73.491	-44.405	1323	0.714644712	-2.624953079	1.258434399
174070	2/14/2019 1:31	B	1015361.615	473936.3851	-73.494	-44.425	2626	0.578096918	-2.725587292	1.345807054
174070	2/14/2019 1:59	B	1828976.668	1040085.832	-73.494	-44.428	1659	0.552843332	-2.769311122	1.358910203
174070	2/14/2019 2:28	1	24470190.72	55347.27967	-73.504	-44.41	1770	0.675718197	-2.466664455	1.24506601

174070	2/14/2019 2:59	A	356421875.6	11090945.43	-73.482	-44.413	1808	0.671323279	-2.582163042	1.280717454
174070	2/14/2019 3:27	2	235104.1201	39872.87989	-73.505	-44.407	1734	0.682886524	-2.430984369	1.216949596
174070	2/14/2019 3:47	B	16674350.39	3116062.607	-73.486	-44.418	1201	0.654979406	-2.711012682	1.325740273
174070	2/14/2019 4:13	1	1309946.377	495151.6234	-73.506	-44.405	1526	0.679748813	-2.387717995	1.195104677
174070	2/14/2019 5:10	B	61375300.1	16888138.4	-73.508	-44.411	3437	0.668951224	-2.41707179	1.228212246
174070	2/14/2019 6:21	B	102879463.2	15821461.28	-73.545	-44.363	4247	0.029404058	-1.474989659	0.899306108
174070	2/14/2019 8:06	1	57280037.62	7602032.883	-73.575	-44.296	6284	-0.73968719	-1.291382374	0.830148238
174070	2/14/2019 9:44	A	163552.5023	5991.9977	-73.591	-44.239	5874	-0.347231493	-1.801897946	0.781996316
174070	2/14/2019 11:44	B	94652239.78	1877828.224	-73.59	-44.285	7218	-0.418161305	-1.29881407	0.76768228
174070	2/14/2019 12:11	B	5471517.559	967772.9415	-73.643	-44.293	1596	-0.527136774	-0.995184445	0.624206824
174070	2/14/2019 12:59	B	5290799.561	1101858.939	-73.648	-44.287	2898	-0.574106325	-1.022431717	0.611960535
174070	2/14/2019 13:11	B	4891434.561	13286545.94	-73.667	-44.286	716	-0.674601254	-0.933878314	0.575480243
174070	2/14/2019 13:21	B	6823118.671	1016709.829	-73.65	-44.289	641	-0.593159405	-1.008248869	0.608478988
174070	2/14/2019 14:56	B	7464138.899	4682713.601	-73.443	-44.372	5687	0.415685177	-2.866475189	1.230131093
174070	2/14/2019 15:33	0	1290065.406	18916559.59	-73.401	-44.36	2187	0.513606814	-2.866475189	1.360120243
174070	2/14/2019 20:16	B	279687209.7	24431102.84	-73.322	-44.124	17014	0.080751905	-2.84883054	1.368173105

174070	2/14/2019 21:22	0	1366252.698	10230247.8	-73.503	-44.151	3931	-0.265805105	-1.351077307	1.057413482
174070	2/14/2019 21:56	B	48201783.64	3724424.863	-73.517	-44.117	2079	-0.09751955	-1.151177345	1.118202481
174070	2/14/2019 22:59	A	581957782.3	768770.1668	-73.524	-44.189	3742	-0.671556827	-1.739320114	0.944755533
174070	2/14/2019 23:23	B	42374014.6	946881.3996	-73.514	-44.176	1480	-0.543686924	-1.522093796	0.958208481
174070	2/14/2019 23:46	B	71314008.25	71314008.25	-73.575	-44.182	1337	-0.580590564	-1.218763783	0.867193578
174070	2/15/2019 1:11	1	2327728.037	257702.4629	-73.554	-44.246	5118	0.215073448	-2.076867045	2.343380416
174070	2/15/2019 1:28	1	1050854.352	484018.6481	-73.593	-44.24	995	0.077234982	-1.787736183	2.530870988
174070	2/15/2019 2:08	2	1070150.623	16474.37706	-73.555	-44.249	2415	0.210956018	-1.991094027	2.345772916
174070	2/15/2019 2:39	A	173249267.6	1921968.885	-73.577	-44.235	1886	0.276419573	-1.997872385	2.448613095
174070	2/15/2019 2:47	B	43952870.76	1093202.236	-73.578	-44.236	472	0.276419573	-1.956252055	2.448613095
174070	2/15/2019 3:09	1	564347.9022	423078.5978	-73.566	-44.24	1286	0.28955691	-2.000572303	2.404448901
174070	2/15/2019 3:34	B	113660108.3	20569314.17	-73.566	-44.241	1530	0.245987362	-2.132826311	2.358071288
174070	2/15/2019 3:50	1	4252293.411	3717669.089	-73.541	-44.241	938	0.132025769	-2.264971773	2.292763716
174070	2/15/2019 4:18	B	21023033.08	5997301.425	-73.546	-44.242	1699	0.150525857	-2.225723716	2.304094844
174070	2/15/2019 5:25	B	47580713.56	12397698.94	-73.553	-44.244	4035	0.215073448	-2.118607739	2.343380416
174070	2/15/2019 7:44	B	99187370.34	18770626.16	-73.57	-44.247	8309	0.269037467	-1.911056873	2.422159191

174070	2/15/2019 9:09	B	6278379.39	1065929.11	-73.572	-44.247	5127	0.274423405	-1.897892425	2.443014962
174070	2/15/2019 9:31	B	29245.81902	50235011.18	-73.568	-44.255	1291	0.232850766	-1.840394513	2.410676046
174070	2/15/2019 10:52	A	4953407723	308007421.3	-73.612	-44.213	4858	0.171500814	-1.96523466	2.339074684
174070	2/15/2019 11:49	B	4348363.645	72509946.86	-73.525	-44.217	3463	-0.010977385	-2.209982034	2.219529746
174070	2/15/2019 12:24	0	148458750	300859243	-73.54	-44.241	2091	0.186053417	-2.271055891	2.321011645
174070	2/15/2019 12:48	B	478498633.8	12694426.67	-73.59	-44.216	1444	0.1722139	-1.566034188	2.513548573
174070	2/15/2019 14:32	B	370780070.5	30348269.46	-73.837	-44.221	6230	0.696793364	-0.30483871	2.352376802
174070	2/15/2019 15:10	B	145844163.8	13757889.23	-73.906	-44.236	2265	0.382000655	-0.185621134	2.154672112
174070	2/15/2019 20:54	B	2505805994	332477920	-73.489	-44.103	20625	1.438390243	-1.367586356	2.388178064
174070	2/15/2019 22:35	0	319961261.7	52419638.28	-73.36	-44.127	6105	1.01962808	-2.811650746	2.91850196
174070	2/15/2019 22:57	B	96270078.63	20139019.37	-73.34	-44.122	1322	1.049689368	-2.866475189	2.85862074
174070	2/16/2019 0:09	B	159899833.6	25219843.39	-73.267	-44.107	4274	0.157190597	-2.348822639	2.939243147
174070	2/16/2019 1:11	B	584364.9326	27185453.57	-73.214	-44.038	3736	0.701658312	-1.03079254	2.782627422
174070	2/16/2019 1:44	0	3878156304	43190508.58	-73.299	-44.047	1978	0.55849494	-1.234565292	2.71131805
174070	2/16/2019 2:24	1	2020477.261	319155.7394	-73.276	-44.042	2404	0.531393029	-1.642238945	2.622923677
174070	2/16/2019 3:25	1	2125451.314	512451.1863	-73.32	-44.038	3668	1.627983495	-2.077454229	2.731989597

174070	2/16/2019 3:26	A	2708798.403	19615994.1	-73.319	-44.037	76	1.627983495	-2.077454229	2.731989597
174070	2/16/2019 4:28	1	3407515.246	192557.2542	-73.305	-44.033	3667	1.117313275	-1.834284066	2.617509674
174070	2/16/2019 5:06	B	2350342.401	879978.5993	-73.317	-44.039	2328	1.561661496	-2.069494567	2.727716849
174070	2/16/2019 7:38	0	2735988.901	718884.0994	-73.326	-44.02	9104	1.862511155	-1.654518643	2.653745421
174070	2/16/2019 9:20	A	107679559.1	2727900.885	-73.205	-43.978	6132	0.406925019	-1.066612462	2.491819842
174070	2/16/2019 10:19	B	8441940.5	1988018	-73.186	-43.967	3547	0.253343024	-1.190046243	2.498000905
174070	2/16/2019 11:39	B	7821012.5	2544768	-73.215	-43.967	4755	0.306568746	-1.178288471	2.429478689
174070	2/16/2019 13:12	B	36497394.41	17390.08933	-73.525	-44.041	5631	1.216756106	-1.050632212	3.062816682
174070	2/16/2019 14:11	B	11692245.35	2783889.149	-73.573	-44.064	3529	0.348912436	-0.822066897	2.837223311
174070	2/16/2019 14:47	B	21224260.61	5253667.392	-73.572	-44.064	2141	0.348912436	-0.816980144	2.837223311
174070	2/16/2019 20:20	A	39389149.04	20831219.96	-73.655	-44.111	19998	-0.551374223	-0.551896233	2.739368664
174070	2/16/2019 22:00	A	12302532.5	45050365.5	-73.467	-44.195	5985	1.729654449	-2.496708301	2.434723937
174070	2/16/2019 22:35	B	63469623.33	36524696.67	-73.493	-44.239	2120	0.33238975	-2.695200998	2.253646778
174070	2/16/2019 23:12	0	17496283.8	299254.6962	-73.566	-44.254	2227	0.881389519	-1.858391042	2.443137704
174070	2/16/2019 23:34	1	504319.9358	365148.5642	-73.579	-44.24	1319	1.065581571	-1.921169547	2.605623099
174070	2/16/2019 23:56	B	102994782.3	7481842.67	-73.621	-44.243	1285	0.284435342	-1.451974598	2.769173584

174070	2/17/2019 0:22	B	178936763.2	61903741.81	-73.661	-44.273	1543	-0.695687738	-1.063876539	2.392354521
174070	2/17/2019 0:49	B	147964956.6	39046429.86	-73.671	-44.279	1625	-0.692368879	-1.024566064	2.389781528
174070	2/17/2019 1:31	1	8530981.235	534555.765	-73.571	-44.245	2548	-1.121177916	-1.935676491	2.454390324
174070	2/17/2019 2:27	1	72109591.6	555201.4031	-73.608	-44.248	3381	-1.066452373	-1.57161144	2.468674807
174070	2/17/2019 3:14	1	642699.6495	959525.3505	-73.576	-44.254	2762	-1.090706993	-1.776943488	2.448371129
174070	2/17/2019 3:38	B	409488513.4	8113807.606	-73.577	-44.249	1482	-1.112807319	-1.869106235	2.453226194
174070	2/17/2019 4:11	2	107562.3421	158946.6579	-73.567	-44.242	1960	-1.127588561	-2.009363321	2.450257585
174070	2/17/2019 4:45	B	10321897.91	2967846.087	-73.557	-44.249	2021	-1.137705111	-1.991094027	2.43893949
174070	2/17/2019 7:26	2	34927.44606	295601.0539	-73.604	-44.232	9684	-0.985494002	-1.598801023	2.465387205
174070	2/17/2019 9:04	A	2473754.27	37830.73018	-73.588	-44.23	5879	-1.077382523	-1.882818194	2.462175634
174070	2/17/2019 9:58	B	35292126.44	2903473.556	-73.575	-44.237	3260	-1.137833676	-1.997872385	2.456845771
174070	2/17/2019 10:47	A	31061954.44	16303548.06	-73.517	-44.153	2949	-0.491176174	-1.241126349	2.494475892
174070	2/17/2019 11:49	0	26309752.04	11134986.46	-73.553	-44.196	3700	-1.057024095	-1.518500939	2.436604751
174070	2/17/2019 12:15	B	38552779.07	9078523.432	-73.549	-44.206	1558	-1.150149413	-1.706872041	2.44437809
174070	2/17/2019 12:59	0	1177718.596	17998586.4	-73.553	-44.136	2657	-0.599800303	-1.002957462	2.388131886
174070	2/17/2019 13:25	A	929862.4299	1879410.07	-73.516	-44.101	1560	-0.240406204	-1.17409607	2.444454966

174070	2/17/2019 13:29	B	9037470.527	3559294.473	-73.515	-44.104	217	-0.241900181	-1.182017901	2.449239026
174070	2/17/2019 13:56	0	4830182.949	3291169.051	-73.507	-44.149	1625	-0.376337289	-1.282521873	2.520397468
174070	2/17/2019 14:46	B	13568129.71	4002932.787	-73.468	-44.105	3019	0.25718962	-1.547104948	2.61624456
174070	2/17/2019 15:01	B	10635846.31	3600566.192	-73.47	-44.105	870	0.242951836	-1.531472647	2.613158517
174070	2/17/2019 15:37	B	11039732.79	3564969.714	-73.449	-44.091	2198	0.596553181	-1.791141277	2.640659526
174070	2/17/2019 21:23	A	529837716.3	4064696.161	-73.412	-44.16	20727	0.135752433	-2.383919067	2.820855471
174070	2/17/2019 21:33	B	20930993.93	173312.0717	-73.424	-44.16	589	0.186446666	-2.363563102	2.803713404
174070	2/17/2019 23:08	B	2246013.712	4656466.288	-73.542	-44.234	5700	-1.184486139	-2.293606854	2.437594171
174070	2/17/2019 23:41	B	144861004.7	51145307.34	-73.592	-44.276	1982	-1.017825871	-1.479508785	2.440160331
174070	2/18/2019 0:28	B	1853015.51	533946.9902	-73.605	-44.285	2848	-0.402089327	-1.246918828	1.208666442
174070	2/18/2019 1:35	A	2855220.325	11222181.68	-73.51	-44.244	4008	-0.294376699	-2.480503942	1.140003922
174070	2/18/2019 2:06	1	11897442	17672	-73.442	-44.249	1859	-0.388507903	-2.822702813	1.108622715
174070	2/18/2019 2:42	B	961844944.7	101551220.3	-73.427	-44.223	2134	-0.574954758	-2.866475189	1.044979447
174070	2/18/2019 3:01	B	18182265.89	2114078.611	-73.434	-44.223	1160	-0.635427354	-2.866475189	1.032839263
174070	2/18/2019 3:17	B	2778752.497	382768.0034	-73.416	-44.216	974	-0.821505482	-2.866475189	1.008501833
174070	2/18/2019 3:51	B	9916638.659	260667473.8	-73.489	-44.221	2026	-0.369260493	-2.50008006	1.033750865

174070	2/18/2019 4:30	B	10263446.94	398565.5566	-73.382	-44.195	2363	-0.815702093	-2.866475189	0.997947897
174070	2/18/2019 7:11	B	4563020.109	3025222.391	-73.495	-44.208	9631	-0.355967793	-2.3175069	1.018834314
174070	2/18/2019 8:59	A	6122082859	160831703.8	-73.564	-44.195	6475	-0.363627888	-1.868420724	0.989389305
174070	2/18/2019 9:34	B	42932591.7	35278398.8	-73.511	-44.221	2139	-0.31495475	-2.440299265	1.056654994
174070	2/18/2019 10:34	B	3488650.529	16300862.47	-73.47	-44.209	3602	-0.423149985	-2.558710755	1.014087318
174070	2/18/2019 11:13	B	14693054.67	2671214.329	-73.471	-44.217	2297	-0.402921371	-2.66092426	1.033746333
174070	2/18/2019 11:55	0	20607672.81	3509577.192	-73.594	-44.245	2527	-0.341418087	-1.747029173	1.102606497
174070	2/18/2019 12:32	B	49970004.5	12655480.5	-73.619	-44.247	2213	-0.353649126	-1.418317798	1.079619145
174070	2/18/2019 12:53	B	138537998.9	11514635.58	-73.619	-44.242	1251	-0.351541689	-1.532394943	1.076368746
174070	2/18/2019 13:30	B	513461040.3	17617334.18	-73.545	-44.259	2251	-0.346526881	-1.680559245	1.16608293
174070	2/18/2019 14:10	B	364805415.6	14536738.9	-73.589	-44.283	2405	-0.397136882	-1.414841097	1.230225166
174070	2/18/2019 20:59	B	1164272753	475340151.2	-73.609	-44.289	24533	-0.395988781	-1.29749417	1.201682737
174070	2/18/2019 22:34	B	8010065.218	554835.2823	-73.584	-44.242	5685	-0.334616515	-1.893409718	1.10487206
174070	2/19/2019 0:16	B	4241853.607	957770.3933	-73.444	-44.271	6130	-0.567157593	-2.745580056	0.585414437
174070	2/19/2019 2:28	A	760727876	150834329	-73.355	-44.289	7908	-0.3502537	-2.866475189	0.748782855
174070	2/19/2019 2:44	B	3206745.746	325892.7544	-73.362	-44.3	996	-0.272723191	-2.866475189	0.781940466

174070	2/19/2019 2:50	B	23047644.01	2762355.988	-73.352	-44.29	330	-0.224417108	-2.866475189	0.812243465
174070	2/19/2019 3:04	B	37967432.57	1470669.931	-73.332	-44.272	823	-0.289915569	-2.866475189	0.873065908
174070	2/19/2019 3:28	1	1461041.208	313683.2919	-73.303	-44.29	1449	0.326282249	-2.811882457	0.993968466
174070	2/19/2019 4:08	A	137403859.4	16358877.05	-73.255	-44.262	2405	0.852433962	-2.800233578	1.203039356
174070	2/19/2019 5:09	B	251505537.2	49491629.33	-73.277	-44.286	3663	0.882151456	-2.702696995	1.146258812
174070	2/19/2019 7:01	2	165682.298	71698.20197	-73.287	-44.314	6722	0.509121441	-2.529206274	1.105547826
174070	2/19/2019 8:46	0	8441720.963	2488869.537	-73.303	-44.337	6297	0.496552147	-2.724900599	1.055237418
174070	2/19/2019 9:13	B	15844939.74	3766902.259	-73.305	-44.347	1617	0.526312803	-2.727251653	1.06345477
174070	2/19/2019 10:21	B	473790041.7	412007696.8	-73.312	-44.351	4077	0.349268081	-2.866475189	0.9328276
174070	2/19/2019 10:47	0	1226340.374	9600282.126	-73.357	-44.333	1551	-0.149854992	-2.866475189	0.805851373
174070	2/19/2019 10:54	B	33155861.34	11806588.66	-73.357	-44.333	468	-0.149854992	-2.866475189	0.805851373
174070	2/19/2019 11:03	B	52448330.19	18107988.31	-73.356	-44.333	531	-0.149854992	-2.866475189	0.805851373
174070	2/19/2019 12:07	B	2130142.854	3012983.646	-73.356	-44.342	3847	-0.081930242	-2.866475189	0.822055903
174070	2/19/2019 12:27	B	301102903.4	50026533.58	-73.352	-44.311	1197	-0.086571725	-2.866475189	0.825710955
174070	2/19/2019 13:44	0	7457887102	635797673.7	-73.258	-44.264	4581	0.262098393	-2.866475189	0.962494945
174070	2/19/2019 14:25	B	2051298506	185996787	-73.269	-44.284	2478	0.72737116	-2.75324582	1.074190817

174070	2/19/2019 14:49	B	3616976979	168784091.1	-73.256	-44.306	1444	0.617110762	-2.586232502	1.107944294
174070	2/19/2019 15:29	B	1980933.845	3649267.155	-73.289	-44.313	2377	0.509803085	-2.617227803	1.108421532
174070	2/19/2019 20:26	A	74074.60906	2222797.891	-73.458	-44.238	17818	-0.326546059	-2.821139514	0.48561747
174070	2/19/2019 21:02	B	1588513.881	566736.1188	-73.468	-44.241	2155	-0.398765042	-2.793521229	0.474401369
174070	2/19/2019 22:07	B	70959229.26	5839827.241	-73.456	-44.208	3909	-0.429628835	-2.763137764	0.425325002
174070	2/19/2019 22:48	A	5347021.651	1165770.849	-73.339	-44.255	2470	-0.042810209	-2.866475189	0.823619071
174070	2/19/2019 23:04	B	5435152.066	8886964.434	-73.346	-44.254	974	-0.008159594	-2.866475189	0.80709512
174070	2/19/2019 23:44	B	2347641.392	6240250.608	-73.343	-44.248	2419	0.09323545	-2.866475189	0.799296681
174070	2/20/2019 0:20	B	12233851	7490838.995	-73.322	-44.257	2118	0.614258036	-2.866475189	0.270846108
174070	2/20/2019 0:39	1	2332162.967	40885.53298	-73.359	-44.279	1176	-0.031254505	-2.866475189	0.349334648
174070	2/20/2019 0:59	B	7266018.352	3296421.648	-73.349	-44.282	1180	0.065710691	-2.866475189	0.370961001
174070	2/20/2019 1:32	B	20851507.46	9882587.038	-73.338	-44.27	1980	0.408911576	-2.866475189	0.328104109
174070	2/20/2019 1:59	A	54765.26916	37433.23084	-73.374	-44.251	1635	0.639042618	-2.866475189	0.199971088
174070	2/20/2019 2:24	1	70188.40442	695232.0956	-73.329	-44.258	1479	0.47670772	-2.866475189	0.2610984
174070	2/20/2019 2:37	B	5564492.511	19642151.49	-73.352	-44.272	795	0.263721017	-2.866475189	0.32349759
174070	2/20/2019 3:08	1	1208554.166	247078.8341	-73.315	-44.242	1882	0.69385733	-2.854078241	0.230637203

174070	2/20/2019 3:44	B	748346.0914	20338960.41	-73.331	-44.242	2146	0.291740642	-2.866475189	0.195461005
174070	2/20/2019 6:54	A	14484601.17	10210538.83	-73.405	-44.32	11393	0.215462672	-2.866475189	0.209433964
174070	2/20/2019 8:34	A	83559.5	176149.5	-73.449	-44.317	5981	0.10322654	-2.682223459	0.006776853
174070	2/20/2019 8:44	B	200236.3233	77036.6767	-73.449	-44.317	602	0.10322654	-2.682223459	0.006776853
174070	2/20/2019 10:16	1	1816706.398	1480010.602	-73.526	-44.247	5549	0.434308863	-2.251952203	-0.359166868
174070	2/20/2019 10:23	2	778518.2293	11483.77074	-73.522	-44.233	427	0.537208924	-2.431330882	-0.395448154
174070	2/20/2019 10:53	B	8162158.709	1901857.791	-73.511	-44.223	1746	0.703254917	-2.486406163	-0.403903917
174070	2/20/2019 11:52	B	21478151.76	5063320.739	-73.532	-44.209	3588	-0.159668183	-1.907888679	-0.528592775
174070	2/20/2019 11:53	B	34937980.67	5823149.326	-73.532	-44.207	46	-0.251635947	-1.902311249	-0.53840389
174070	2/20/2019 12:03	B	22279856.32	2079028.681	-73.537	-44.198	589	-0.500177887	-1.670415443	-0.558509943
174070	2/20/2019 12:23	B	25996542.03	139218.4744	-73.538	-44.227	1231	0.153146287	-2.285678986	-0.474417147
174070	2/20/2019 12:54	B	480142849.1	1361263.412	-73.534	-44.186	1809	-0.664255077	-1.598498752	-0.563102425
174070	2/20/2019 13:34	A	980614.9586	2508229.541	-73.476	-44.19	2427	0.266306257	-2.258729291	-0.437686938
174070	2/20/2019 14:04	B	4148766.164	3938180.336	-73.466	-44.177	1818	-0.019853108	-2.19941472	-0.439166906
174070	2/20/2019 14:31	B	13792078.1	450630.898	-73.493	-44.209	1579	0.749330876	-2.33811194	-0.406358166
174070	2/20/2019 15:05	A	202797834.9	880122.1054	-73.507	-44.22	2050	0.81462471	-2.523963331	-0.388895972

174070	2/20/2019 21:33	B	4143299.524	189310533	-73.332	-44.243	23318	0.312300016	-2.866475189	0.218481909
174070	2/20/2019 22:27	A	1123113.884	94791.11588	-73.373	-44.29	3234	-0.203837793	-2.866475189	0.339046347
174070	2/20/2019 22:33	B	502666.1306	51470.36945	-73.373	-44.291	333	-0.203837793	-2.866475189	0.339046347
174070	2/20/2019 23:14	B	1899703.98	878834.5201	-73.354	-44.284	2479	-0.044922071	-2.866475189	0.366531477
174070	2/21/2019 0:08	B	2258317.51	675744.9896	-73.352	-44.288	3224	0.554455106	-2.866475189	-0.585234885
174070	2/21/2019 0:17	B	10879375.06	70410.93938	-73.352	-44.302	548	0.759774165	-2.866475189	-0.65283226
174070	2/21/2019 0:46	B	3314000.869	1146903.631	-73.352	-44.291	1715	0.555588691	-2.866475189	-0.597716932
174070	2/21/2019 1:07	1	460915.9871	325998.0129	-73.359	-44.296	1275	0.771681576	-2.866475189	-0.621169923
174070	2/21/2019 1:39	B	10614467.44	2856025.057	-73.357	-44.299	1946	0.941351976	-2.866475189	-0.661878958
174070	2/21/2019 2:20	A	7233081.661	110088.3387	-73.359	-44.314	2413	1.221303697	-2.866475189	-0.739934666
174070	2/21/2019 2:31	2	995096.3443	109350.1557	-73.376	-44.322	704	1.839852767	-2.866475189	-0.873468244
174070	2/21/2019 2:42	A	6510507.285	457032.7153	-73.376	-44.32	644	1.866575982	-2.866475189	-0.864406537
174070	2/21/2019 3:24	A	1692717.015	224742.9847	-73.423	-44.334	2488	1.315693873	-2.866475189	-1.104418946
174070	2/21/2019 3:55	B	1408408.91	345935.0899	-73.431	-44.34	1882	0.729428827	-2.847042389	-1.159356038
174070	2/21/2019 4:08	B	2002981.905	474992.5951	-73.434	-44.343	803	0.496090261	-2.845032099	-1.18390862
174070	2/21/2019 4:26	2	148050.9061	101278.0939	-73.425	-44.349	1058	0.244845457	-2.866475189	-1.189550409

174070	2/21/2019 5:05	A	5396547.461	2298717.539	-73.422	-44.351	2319	0.107675347	-2.866475189	-1.196862001
174070	2/21/2019 6:40	A	6314634.14	1539238.36	-73.394	-44.362	5713	0.273819002	-2.866475189	-1.161010298
174070	2/21/2019 8:20	3	127677.8798	55934.62018	-73.406	-44.387	6013	0.018588404	-2.866475189	-1.194893594
174070	2/21/2019 9:39	B	6635279.738	301877.2617	-73.412	-44.404	4755	0.539519325	-2.866475189	-1.152550193
174070	2/21/2019 10:05	B	10259852.05	3134566.454	-73.377	-44.392	1524	1.036519622	-2.866475189	-1.089033346
174070	2/21/2019 10:36	A	25463716.64	15567235.86	-73.416	-44.399	1892	0.433988288	-2.866475189	-1.162541566
174070	2/21/2019 11:25	B	203306013.8	7701676.163	-73.403	-44.417	2936	0.749015225	-2.866475189	-1.150817686
174070	2/21/2019 11:32	B	24786041.42	1032208.575	-73.403	-44.417	431	0.704264982	-2.866475189	-1.154618839
174070	2/21/2019 12:20	B	325869757.2	26794374.76	-73.396	-44.445	2851	2.307119865	-2.866475189	-0.999143133
174070	2/21/2019 12:33	B	26594321.98	2038770.516	-73.426	-44.441	792	2.408993112	-2.866475189	-0.929655216
174070	2/21/2019 13:10	B	4159305.747	489315.2527	-73.418	-44.454	2230	3.502179992	-2.866475189	-0.90733223
174070	2/21/2019 13:39	B	4517171.885	371660.6153	-73.417	-44.458	1751	4.035776768	-2.866475189	-0.839805353
174070	2/21/2019 14:18	0	5059241.235	15363117.26	-73.423	-44.446	2304	2.54500183	-2.866475189	-0.933200725
174070	2/21/2019 14:47	B	83975700.56	19579851.94	-73.422	-44.442	1756	2.408993112	-2.866475189	-0.929655216
174070	2/21/2019 21:06	1	48280948.83	2586111.667	-73.486	-44.454	22716	1.252490901	-2.660869188	-0.45261491
174070	2/21/2019 22:13	1	95905859.13	4338831.37	-73.406	-44.424	4061	1.362252577	-2.866475189	-1.048916817

174070	2/21/2019 22:39	A	63668865.9	9607654.1	-73.468	-44.478	1532	3.189652398	-2.708732105	-0.364249521
174070	2/21/2019 23:50	B	230035630.7	22864406.35	-73.43	-44.478	4293	4.980907174	-2.830798551	-0.415027875
174070	2/22/2019 0:01	B	202240347.5	26482633.52	-73.435	-44.478	608	3.11221435	-2.835895213	-0.341825015
174070	2/22/2019 0:22	B	139436845.7	9624095.253	-73.434	-44.465	1291	2.801354691	-2.830798551	-0.400954
174070	2/22/2019 0:31	B	32945158.74	674445.7638	-73.447	-44.486	564	2.882877636	-2.815508563	-0.202383461
174070	2/22/2019 1:26	B	16141485.21	4493588.785	-73.497	-44.475	3257	1.361469743	-2.151017431	-0.29078791
174070	2/22/2019 2:13	1	3746527.597	34440.40305	-73.524	-44.464	2861	0.988281354	-1.873428295	-0.392118348
174070	2/22/2019 2:25	1	9405006.949	30918.05115	-73.496	-44.458	669	1.192620399	-2.432008862	-0.407419323
174070	2/22/2019 3:06	1	483336564.5	15029378.03	-73.483	-44.461	2505	1.622017116	-2.617372362	-0.404423344
174070	2/22/2019 4:08	2	583256.858	36539.64198	-73.454	-44.455	3702	1.904595675	-2.828420108	-0.519379993
174070	2/22/2019 6:26	B	20135614.38	4448680.123	-73.409	-44.457	8309	2.830959335	-2.866475189	-0.57635514
174070	2/22/2019 8:04	1	870321.1615	71763.83849	-73.521	-44.415	5872	0.774428963	-2.227875042	-0.713474552
174070	2/22/2019 9:31	1	992874.6093	510126.3907	-73.548	-44.43	5201	0.779034068	-1.881814851	-0.652410347
174070	2/22/2019 9:49	0	30010301.51	19757220.99	-73.532	-44.422	1070	0.811831151	-2.055270785	-0.675508359
174070	2/22/2019 10:51	0	85269933.63	52157500.87	-73.552	-44.421	3723	0.687798282	-1.742605249	-0.706090725
174070	2/22/2019 11:11	B	66963226.61	16777106.39	-73.552	-44.421	1228	0.675152092	-1.71964058	-0.706811619

174070	2/22/2019 12:03	B	162090661	29868069.05	-73.563	-44.418	3089	0.687652271	-1.880203575	-0.726206258
174070	2/22/2019 12:32	B	266359.7694	1787544.231	-73.49	-44.419	1757	0.886516661	-2.711012682	-0.6842504
174070	2/22/2019 12:45	B	3529453.305	1968410.695	-73.515	-44.439	797	0.974047669	-2.427919656	-0.555893944
174070	2/22/2019 13:19	B	1795707.559	489694.9413	-73.493	-44.42	2038	0.892246745	-2.711012682	-0.671229796
174070	2/22/2019 13:52	0	29865125.67	4092876.829	-73.536	-44.437	1950	0.911646835	-2.191031476	-0.589465478
174070	2/22/2019 14:22	A	1045103278	171178334.9	-73.574	-44.446	1831	0.889006278	-2.073247085	-0.574825165
174070	2/22/2019 14:57	B	2423302.857	747602.143	-73.515	-44.417	2068	0.802775424	-2.300684802	-0.701393954
174070	2/22/2019 20:23	A	70659.2438	1182447.256	-73.533	-44.393	19590	0.42281911	-1.761096428	-0.85018272
174070	2/22/2019 20:31	B	109812.2235	309740.2765	-73.532	-44.393	428	0.464910183	-1.761096428	-0.838605857
174070	2/22/2019 22:11	0	171047404.5	16158893.49	-73.48	-44.404	6008	0.787836035	-2.691579868	-0.779181269
174070	2/22/2019 23:30	B	347724827	33928731.53	-73.464	-44.427	4777	0.986988221	-2.782986064	-0.654643319
174070	2/23/2019 0:20	1	498289.2486	883751.2514	-73.547	-44.389	3000	-0.160652948	-1.574858015	0.246987416
174070	2/23/2019 0:25	B	483629.2683	527687.2317	-73.545	-44.389	279	-0.228332832	-1.599963725	0.2550841
174070	2/23/2019 1:05	1	835568.9679	78059.53214	-73.521	-44.411	2432	-0.51908803	-2.179519458	0.297865328
174070	2/23/2019 1:40	B	19054240.85	4928225.145	-73.521	-44.411	2083	-0.489860944	-2.094789074	0.292707822
174070	2/23/2019 2:01	1	20971480.14	1749248.856	-73.552	-44.42	1243	-0.210514317	-1.883566459	0.237005763

174070	2/23/2019 2:07	2	237416.5916	60507.90843	-73.53	-44.419	375	-0.353478516	-2.082102188	0.267413626
174070	2/23/2019 2:40	A	192943952.7	294383.3069	-73.555	-44.403	1948	-0.157376639	-1.642591882	0.234063179
174070	2/23/2019 3:13	B	218586029.4	8259482.614	-73.562	-44.427	1993	-0.113937122	-1.764501666	0.18540556
174070	2/23/2019 4:25	1	639652.5789	36989.92114	-73.486	-44.418	4310	-0.728549696	-2.720369221	0.303682193
174070	2/23/2019 5:25	B	7983686.867	2060555.133	-73.491	-44.42	3592	-0.664823272	-2.725587292	0.301178369
174070	2/23/2019 6:16	B	15475548.18	8240916.319	-73.485	-44.424	3112	-0.6440348	-2.765840976	0.294661765
174070	2/23/2019 7:59	B	3010109.898	1053799.102	-73.481	-44.427	6178	-0.65654746	-2.808176748	0.288156634
174070	2/23/2019 9:12	B	75553666.08	747991.9161	-73.463	-44.339	4341	-0.206179908	-2.595695602	0.245318374
174070	2/23/2019 9:31	B	42741918.36	5232146.138	-73.487	-44.343	1129	-0.318989311	-2.458618971	0.276443215
174070	2/23/2019 10:21	0	3131849.387	623632730.6	-73.414	-44.396	3024	-0.389905818	-2.866475189	0.166686626
174070	2/23/2019 10:46	A	48186341.92	6724724.581	-73.459	-44.421	1524	-0.764871754	-2.866475189	0.265405496
174070	2/23/2019 11:56	A	649238.5822	278841.4178	-73.501	-44.373	4206	-0.829340899	-2.245202567	0.334739939
174070	2/23/2019 12:25	B	56170633.71	1262039.288	-73.515	-44.392	1701	-0.747090395	-2.081047652	0.325487596
174070	2/23/2019 12:26	B	42101623.84	1107714.164	-73.516	-44.392	47	-0.747090395	-2.081047652	0.325487596
174070	2/23/2019 13:00	B	49620901.99	4570476.514	-73.519	-44.379	2052	-0.63082202	-1.944925457	0.318696617
174070	2/23/2019 13:29	A	56538906873	2139029783	-73.561	-44.371	1763	-0.292961873	-1.658323825	0.28165877

174070	2/23/2019 14:05	B	2019560155	76375877.04	-73.584	-44.356	2179	0.026733738	-1.480496914	0.231214935
174070	2/23/2019 14:43	B	1128124321	92486324.26	-73.541	-44.385	2264	-0.418976319	-1.712812033	0.294758731
174070	2/23/2019 15:08	B	22848481.57	10112898.43	-73.521	-44.37	1492	-0.522505475	-1.858152147	0.309379554
174070	2/23/2019 20:12	B	20886121.92	975887.0845	-73.477	-44.359	18222	-0.52477499	-2.644364647	0.300974355
174070	2/23/2019 21:40	B	3014830.972	79617526.03	-73.429	-44.358	5265	-0.113802677	-2.866475189	0.154794041
174070	2/23/2019 23:19	1	207334.4567	97274.04335	-73.411	-44.428	5982	-0.742227055	-2.866475189	0.197687274
174070	2/23/2019 23:33	2	167384.9273	106808.0727	-73.413	-44.431	842	-0.829329551	-2.866475189	0.208085099
174070	2/24/2019 0:11	B	5737530.272	1413048.228	-73.402	-44.421	2285	1.327047111	-2.866475189	0.456735474
174070	2/24/2019 0:39	B	1737070739	162369073.5	-73.397	-44.414	1642	1.21760558	-2.866475189	0.421387895
174070	2/24/2019 0:41	B	26155350.93	2450978.071	-73.403	-44.416	133	1.178867757	-2.866475189	0.413900308
174070	2/24/2019 1:49	1	526790.6393	85558.36072	-73.424	-44.421	4100	0.795354133	-2.866475189	0.453324496
174070	2/24/2019 1:51	B	1478797.386	157763.1145	-73.423	-44.421	96	0.783140322	-2.866475189	0.458397065
174070	2/24/2019 2:20	B	6541754.852	1187423.648	-73.427	-44.427	1726	0.928163427	-2.866475189	0.447736906
174070	2/24/2019 3:00	B	14346543.49	7378309.01	-73.421	-44.45	2405	1.716507966	-2.866475189	0.469379522
174070	2/24/2019 3:33	B	24588967.72	631305.2801	-73.418	-44.434	1989	1.19133468	-2.866475189	0.482408454
174070	2/24/2019 4:05	3	19552.09192	23751.90808	-73.417	-44.43	1923	1.104941087	-2.866475189	0.4772441

174070	2/24/2019 5:03	B	4176993.983	319522.017	-73.411	-44.412	3465	0.961363528	-2.866475189	0.405007453
174070	2/24/2019 9:26	2	246668.9124	101204.0876	-73.368	-44.412	15799	1.830476351	-2.866475189	0.38738629
174070	2/24/2019 10:01	B	7062372.655	1798151.845	-73.365	-44.408	2111	1.729242881	-2.866475189	0.366216536
174070	2/24/2019 10:21	A	387592.1658	12825.83418	-73.356	-44.413	1185	1.788837396	-2.795159	0.385094149
174070	2/24/2019 11:31	A	13701635.41	34736.58701	-73.33	-44.444	4192	3.094809705	-2.71278401	0.722060373
174070	2/24/2019 12:06	0	3950781.39	2189243.61	-73.376	-44.41	2098	1.745670092	-2.866475189	0.381412916
174070	2/24/2019 13:07	0	24610005.93	9631979.065	-73.406	-44.351	3704	0.815650319	-2.866475189	0.071943649
174070	2/24/2019 13:27	1	3593765.769	214876.7311	-73.353	-44.382	1194	0.459494007	-2.785351936	0.185669957
174070	2/24/2019 13:42	A	1095858492	41422237.94	-73.375	-44.385	856	0.449627668	-2.754581048	0.215054983
174070	2/24/2019 14:16	B	100290332.8	15853021.23	-73.361	-44.391	2037	0.572224786	-2.750584828	0.248306561
174070	2/24/2019 14:47	B	63972649.38	531407.1238	-73.276	-44.437	1908	1.471448915	-2.047025211	0.640862636
174070	2/24/2019 15:23	B	23099687.4	1068139.102	-73.239	-44.457	2109	-0.090124095	-1.648297864	0.811539819
174070	2/24/2019 20:01	B	106811949.4	12489129.07	-73.272	-44.497	16713	0.262309509	-2.187705777	0.95955149
174070	2/24/2019 21:10	B	2399119243	1812939257	-73.427	-44.467	4127	2.735607739	-2.866475189	0.677908785
174070	2/24/2019 21:40	1	1373704.569	202320.4313	-73.389	-44.404	1802	1.3294384	-2.866475189	0.317864757
174070	2/24/2019 22:33	B	141472219.9	5166441.128	-73.389	-44.377	3188	1.094008196	-2.866475189	0.15745991

174070	2/24/2019 22:51	B	8219919.449	139005.5514	-73.389	-44.378	1108	0.942705214	-2.866475189	0.148106793
174070	2/24/2019 23:21	3	83446.18898	11542.31102	-73.326	-44.409	1763	1.134231198	-2.481920712	0.382772254
174070	2/24/2019 23:58	B	6958787.711	691512.7886	-73.306	-44.418	2220	1.264213465	-2.263750001	0.458261054
174070	2/25/2019 0:17	0	1354336094	51190887.23	-73.303	-44.415	1149	1.443636757	-2.263750001	0.347746935
174070	2/25/2019 0:27	B	3740506.092	405023.9083	-73.307	-44.419	626	1.739075123	-2.271196691	0.371465503
174070	2/25/2019 1:26	A	6170.147657	1115679.852	-73.313	-44.421	3487	2.049448254	-2.41767954	0.383394974
174070	2/25/2019 1:40	A	3150460.505	196188.4951	-73.315	-44.404	867	0.749978856	-2.326187112	0.24600342
174070	2/25/2019 1:58	B	606869.2316	82687.26844	-73.312	-44.398	1095	0.592302757	-2.288870665	0.236828919
174070	2/25/2019 2:32	B	16033294.66	5509733.34	-73.293	-44.407	1994	0.719647959	-1.972673564	0.319366932
174070	2/25/2019 3:07	1	723437.6275	645850.3725	-73.325	-44.409	2123	1.541323931	-2.481920712	0.300530554
174070	2/25/2019 3:20	B	2072091.284	15581907.22	-73.306	-44.406	815	0.946441167	-2.21004253	0.293864295
174070	2/25/2019 3:46	A	269155.5712	18534.42878	-73.343	-44.438	1509	3.745410632	-2.774260482	0.659528857
174070	2/25/2019 5:20	B	6871138.264	1945032.236	-73.332	-44.4	5634	1.211975221	-2.531858683	0.237061336
174070	2/25/2019 7:33	2	116162	47124.5	-73.351	-44.387	7991	0.857428829	-2.779557418	0.162214428
174070	2/25/2019 9:10	A	4010631.584	31414.91558	-73.385	-44.388	5849	1.444412445	-2.866475189	0.229861042
174070	2/25/2019 10:02	B	5277786.262	1264416.238	-73.383	-44.37	3118	0.948462873	-2.866475189	0.116011948

174070	2/25/2019 10:52	B	2817753.08	489343.9205	-73.384	-44.362	2970	0.811455346	-2.866475189	0.080490301
174070	2/25/2019 11:02	B	3155649.182	494016.8181	-73.395	-44.372	600	1.416857134	-2.866475189	0.178693298
174070	2/25/2019 11:32	A	370388299.3	11585405.73	-73.37	-44.341	1825	-0.186315726	-2.866475189	-0.019208864
174070	2/25/2019 11:44	B	92791162.25	2687410.245	-73.368	-44.335	738	-0.38178398	-2.866475189	-0.033911508
174070	2/25/2019 12:36	0	324334980.5	163592	-73.406	-44.347	3069	0.991312113	-2.866475189	0.054237206
174070	2/25/2019 12:49	B	53783305.55	6476703.45	-73.408	-44.349	835	1.379943386	-2.866475189	0.095941917
174070	2/25/2019 13:07	B	463119431.2	43523977.34	-73.428	-44.377	1072	1.463800158	-2.866475189	0.178642091
174070	2/25/2019 13:25	2	2126724.402	156620.0982	-73.386	-44.352	1056	0.5054076	-2.866475189	0.031750795
174070	2/25/2019 14:00	B	139678991.6	30297536.42	-73.417	-44.333	2135	0.767067142	-2.866475189	0.009423372
174070	2/25/2019 14:27	2	180835.5892	274252.9108	-73.405	-44.359	1593	1.504252056	-2.866475189	0.137976745
174070	2/25/2019 15:01	0	17987229.52	81073619.48	-73.346	-44.355	2043	-0.252021803	-2.866475189	0.030271938
174070	2/25/2019 19:51	B	469248733	67530725.55	-73.226	-44.314	17377	0.793500515	-1.736423697	0.673851307
174070	2/25/2019 20:39	B	570670088.5	46654794	-73.227	-44.336	2869	1.212748137	-1.894499458	0.463725699
174070	2/25/2019 21:29	2	1252152.749	156972.2513	-73.361	-44.318	3040	0.098103648	-2.866475189	-0.014177021
174070	2/25/2019 22:19	0	9095677527	282799490.7	-73.322	-44.327	2968	-0.258648133	-2.866475189	-0.044084027
174070	2/25/2019 23:10	1	417628.2209	53621.77914	-73.385	-44.332	3073	-0.066485809	-2.866475189	-0.040079013

174070	2/26/2019 0:00	B	12972948.77	3187049.734	-73.409	-44.335	3034	0.363592825	-2.866475189	0.23924089
174070	2/26/2019 1:06	1	1674599.426	1133596.574	-73.34	-44.332	3916	-0.204383177	-2.866475189	0.267798448
174070	2/26/2019 1:26	2	694044.9249	40493.57509	-73.331	-44.332	1202	-0.168336613	-2.866475189	0.286564968
174070	2/26/2019 1:31	A	73295.32053	15079.17947	-73.329	-44.332	338	-0.110545262	-2.866475189	0.296473901
174070	2/26/2019 1:35	B	126925.1881	33347.3119	-73.328	-44.332	246	-0.110545262	-2.866475189	0.296473901
174070	2/26/2019 2:16	B	837979.449	209334.551	-73.329	-44.332	2420	-0.110545262	-2.866475189	0.296473901
174070	2/26/2019 2:40	B	1271773.893	315722.1075	-73.331	-44.331	1430	-0.151620034	-2.866475189	0.28759465
174070	2/26/2019 3:15	B	20790759.34	796521.6559	-73.329	-44.347	2126	-0.035516532	-2.866475189	0.299210192
174070	2/26/2019 3:21	1	569958.2742	56838.72583	-73.334	-44.341	385	-0.128917251	-2.866475189	0.277459276
174070	2/26/2019 4:26	1	360009.4605	3124533.04	-73.32	-44.348	3851	-0.00126079	-2.866475189	0.330227985
174070	2/26/2019 5:02	B	9377550.751	2952515.749	-73.317	-44.353	2179	0.021031025	-2.808530008	0.346416039
174070	2/26/2019 7:23	0	46036223.4	52860626.6	-73.354	-44.373	8479	0.603458479	-2.810528118	0.396875831
174070	2/26/2019 9:31	A	1492250.721	410878.2791	-73.296	-44.35	7689	0.091709848	-2.559223247	0.419080915
174070	2/26/2019 10:38	A	2129031.076	7614633.924	-73.338	-44.363	4019	0.231620411	-2.808530008	0.339150533
174070	2/26/2019 11:13	B	75042473.14	17490506.86	-73.379	-44.314	2070	-0.211124739	-2.866475189	0.223815696
174070	2/26/2019 12:07	B	2613606.742	47550065.76	-73.303	-44.282	3268	0.123130232	-2.840120077	0.520256685

174070	2/26/2019 13:05	1	9747486.277	20853.72269	-73.378	-44.283	3481	0.301432143	-2.866475189	0.305465842
174070	2/26/2019 14:08	2	985311.3622	12001.13785	-73.399	-44.311	3734	-0.298113358	-2.866475189	0.185717166
174070	2/26/2019 14:40	1	575092.2041	59004.79585	-73.413	-44.322	1952	-0.042181531	-2.866475189	0.197563391
174070	2/26/2019 21:18	1	1537196.298	32821.70152	-73.387	-44.209	23850	0.407696803	-2.866475189	0.508761523
174070	2/26/2019 21:40	B	8046233.499	2013856.501	-73.382	-44.199	1367	0.354012694	-2.866475189	0.561352317
174070	2/26/2019 23:05	B	25516726.53	6265182.473	-73.368	-44.185	5054	-0.081538953	-2.866475189	0.639935737
174070	2/26/2019 23:29	0	9516494.771	1566149.729	-73.36	-44.257	1482	0.226450285	-2.866475189	0.459931942
174070	2/27/2019 0:43	B	295486995.3	52718049.71	-73.349	-44.232	4413	-1.179193435	-2.866475189	0.633243504
174070	2/27/2019 1:04	1	876759.7002	53786.29982	-73.34	-44.25	1238	-1.133945399	-2.866475189	0.624372713
174070	2/27/2019 1:18	A	628719.8622	2266954.138	-73.366	-44.269	872	-0.78278771	-2.866475189	0.604800121
174070	2/27/2019 2:23	1	42669446.77	1091075.727	-73.341	-44.27	3894	-0.629074075	-2.866475189	0.602215154
174070	2/27/2019 2:58	A	1938830.06	301242.4397	-73.329	-44.288	2114	0.185560248	-2.866475189	0.530900864
174070	2/27/2019 3:36	B	27722802.14	6570696.356	-73.315	-44.286	2237	0.113942863	-2.866475189	0.527148724
174070	2/27/2019 4:02	B	27785125.65	7014840.854	-73.325	-44.287	1573	0.192240415	-2.866475189	0.529264357
174070	2/27/2019 4:40	B	167530152.6	43539929.85	-73.354	-44.321	2293	0.392874168	-2.866475189	0.395209239
174070	2/27/2019 7:10	1	2600581.669	294272.8314	-73.345	-44.289	8994	0.137797742	-2.866475189	0.541833569

174070	2/27/2019 9:11	A	50648214.8	606109.6982	-73.358	-44.286	7261	-0.160945027	-2.866475189	0.566961669
174070	2/27/2019 10:00	B	15302116.21	772324.2919	-73.359	-44.288	2951	-0.056587862	-2.866475189	0.55447927
174070	2/27/2019 10:29	0	375634266.8	230130210.2	-73.387	-44.326	1713	0.057714081	-2.866475189	0.430629471
174070	2/27/2019 10:49	B	1163127019	358798223.9	-73.366	-44.349	1216	-0.077599169	-2.866475189	0.371652855
174070	2/27/2019 11:36	0	83813836.64	1882101.356	-73.343	-44.345	2848	-0.323955128	-2.866475189	0.298158536
174070	2/27/2019 12:09	A	42394556.99	25924524.01	-73.307	-44.301	1973	0.499861854	-2.813764965	0.435909944
174070	2/27/2019 13:14	B	18369953.74	9718511.255	-73.384	-44.328	3880	-0.230930407	-2.866475189	0.430134915
174070	2/27/2019 13:42	0	16450233.33	1244579.171	-73.378	-44.385	1711	0.338588347	-2.866475189	0.486070074
174070	2/27/2019 14:22	B	492048053	241329045.5	-73.352	-44.394	2385	0.304880626	-2.866475189	0.452191179
174070	2/27/2019 21:06	A	3370186.477	51201117.52	-73.49	-44.373	24231	0.080002156	-2.492535798	0.355461674
174070	2/27/2019 21:17	B	1737198.868	1802233.132	-73.488	-44.379	685	0.140808552	-2.515671073	0.35328522
174070	2/27/2019 22:50	1	3598270.996	491685.0036	-73.489	-44.402	5530	0.410642419	-2.633281428	0.310194801
174070	2/28/2019 0:21	B	34719821.26	18983395.74	-73.53	-44.395	5459	-0.57824364	-1.914455553	0.058603458
174070	2/28/2019 0:38	A	16281065.48	1357587.021	-73.479	-44.39	1060	-0.686502529	-2.663215125	0.077358086
174070	2/28/2019 1:03	1	64681.2012	1054968.799	-73.501	-44.393	1469	-0.717215871	-2.391190006	0.078275142
174070	2/28/2019 2:06	1	31456661.86	14683.13944	-73.486	-44.339	3777	-0.477184504	-2.284828111	0.179577035

174070	2/28/2019 2:19	B	3849150.787	624435.2134	-73.486	-44.34	824	-0.421228475	-2.354800184	0.170739562
174070	2/28/2019 2:43	A	6236069.776	201948.724	-73.422	-44.348	1418	1.27581392	-2.866475189	-0.088833123
174070	2/28/2019 3:18	B	7887958.347	516422.1533	-73.397	-44.34	2074	2.185437025	-2.866475189	-0.218331647
174070	2/28/2019 3:43	B	274392.0687	746785.9313	-73.368	-44.338	1548	2.61139843	-2.866475189	-0.254542722
174070	2/28/2019 4:26	B	129331.5144	58878.98561	-73.368	-44.309	2578	3.234533843	-2.866475189	0.111514547
174070	2/28/2019 7:00	B	27130367.7	520468.2953	-73.309	-44.246	9194	0.740406539	-2.842536256	0.540580209
174070	2/28/2019 8:35	0	246295295.8	26111664.73	-73.326	-44.276	5704	1.261846374	-2.866475189	0.552555528
174070	2/28/2019 9:26	B	220060779.9	134626714.6	-73.374	-44.286	3065	1.376032729	-2.866475189	0.409534891
174070	2/28/2019 10:19	B	737164417.4	107713839.6	-73.374	-44.264	3220	0.245182022	-2.866475189	0.5365495
174070	2/28/2019 11:03	A	43894938.59	2354101.91	-73.319	-44.265	2642	0.835103616	-2.866475189	0.592072349
174070	2/28/2019 11:46	B	32976813.31	36286612.69	-73.411	-44.275	2534	0.32963738	-2.866475189	0.40441022
174070	2/28/2019 12:24	B	69890189.76	37802222.74	-73.436	-44.245	2274	-0.781362038	-2.851363297	0.495478504
174070	2/28/2019 12:44	1	108103.8625	1000348.637	-73.41	-44.23	1202	-1.064138777	-2.866475189	0.504671489
174070	2/28/2019 15:07	B	215832139.7	32535065.31	-73.509	-44.249	8624	-0.336522569	-2.402784358	0.479057142
174070	2/28/2019 15:44	B	274899181.3	44351791.23	-73.52	-44.243	2204	-0.243119062	-2.365476258	0.465440348
174070	2/28/2019 20:57	B	44241881.08	435631.4249	-73.683	-44.201	18776	0.315428288	-0.842363387	-0.175386008

174070	2/28/2019 22:24	B	4257459485	350063048	-73.592	-44.217	5234	0.298560542	-1.479159249	0.243754765
174070	3/1/2019 0:00	A	9845186.61	18575077.39	-73.505	-44.222	5714	-0.901993772	-2.526055197	0.104545354
174070	3/1/2019 0:16	1	14735912.9	130251.1003	-73.485	-44.209	1012	-0.97396157	-2.438077605	0.138253998
174070	3/1/2019 0:36	B	3690164.505	1182975.995	-73.477	-44.206	1181	-0.986321718	-2.49783222	0.149855648
174070	3/1/2019 0:50	B	783449.0366	16637137.46	-73.497	-44.208	842	-0.973230761	-2.318568375	0.123813856
174070	3/1/2019 1:38	B	20933170.31	416372.1948	-73.486	-44.203	2845	-0.986932632	-2.355246198	0.138507365
174070	3/1/2019 1:59	B	3715333.936	127124.0635	-73.486	-44.201	1316	-0.988588283	-2.296258238	0.136888927
174070	3/1/2019 2:12	2	250632	12482	-73.481	-44.202	782	-0.990324146	-2.355246198	0.142179064
174070	3/1/2019 2:32	1	991652.7666	469987.2334	-73.468	-44.201	1180	-0.98481997	-2.49783222	0.159423565
174070	3/1/2019 3:22	1	8691047.329	422026.671	-73.573	-44.207	2968	-0.800665024	-1.5446334	-0.002624414
174070	3/1/2019 3:59	B	4969193.419	41366611.58	-73.531	-44.207	2225	-0.903048525	-1.870593633	0.067734879
174070	3/1/2019 4:14	B	6667289.141	2586075.359	-73.529	-44.197	924	-0.940205478	-1.676482131	0.07632776
174070	3/1/2019 6:48	A	3237515.771	12329802.73	-73.629	-44.146	9240	-0.857471598	-0.727637169	-0.080451592
174070	3/1/2019 8:27	1	49012710.06	8018972.44	-73.612	-44.173	5922	-0.820101124	-0.957378716	-0.063496738
174070	3/1/2019 9:55	B	1159891303	153718790.3	-73.624	-44.185	5297	-0.821943929	-1.175582783	-0.02682206
174070	3/1/2019 10:42	B	18602703.51	12726556.99	-73.554	-44.219	2813	-0.799117448	-1.930999075	0.02247165

174070	3/1/2019 11:34	B	13419516.91	5957321.586	-73.637	-44.261	3114	-0.701402002	-1.216150424	-0.175052117
174070	3/1/2019 11:59	B	14225946.09	5770306.914	-73.633	-44.261	1532	-0.697205346	-1.27313698	-0.167536927
174070	3/1/2019 12:28	B	17791517.71	30991902.79	-73.559	-44.23	1720	-0.744376335	-2.076891917	0.000180789
174070	3/1/2019 13:05	B	35038585.98	749482064	-73.569	-44.23	2216	-0.764584605	-1.871133128	-0.00339166
174070	3/1/2019 13:52	B	3378782.402	251626.0985	-73.588	-44.201	2843	-0.786527057	-1.33880285	-0.025860597
174070	3/1/2019 14:01	B	93371006	811434.4981	-73.59	-44.195	503	-0.789107821	-1.22848744	-0.038874158
174070	3/1/2019 15:19	B	66086549.31	2781817.187	-73.666	-44.156	4676	-0.795282824	-0.70193252	-0.145976577
174070	3/1/2019 20:43	A	7936319.412	1181605.588	-73.314	-44.33	19459	1.05486386	-2.866475189	0.088833797
174070	3/1/2019 21:52	B	14401956.9	1107903.104	-73.317	-44.297	4160	0.76589305	-2.866475189	0.309981069
174070	3/1/2019 22:21	B	2139952.809	2571945.691	-73.366	-44.292	1727	0.380914452	-2.866475189	0.209233346
174070	3/1/2019 23:29	0	37914724.32	103505544.7	-73.356	-44.285	4051	0.385914968	-2.866475189	0.256568031
174070	3/1/2019 23:54	0	37783196.1	4085576.904	-73.372	-44.295	1543	0.380914452	-2.866475189	0.209233346
174070	3/2/2019 0:05	B	125936013.5	5846586.51	-73.358	-44.292	629	-1.151687727	-2.866475189	0.436676034
174070	3/2/2019 0:42	B	2969154.137	9651414.863	-73.384	-44.29	2253	-1.174172719	-2.866475189	0.423200547
174070	3/2/2019 1:20	A	132781.653	15356.34698	-73.396	-44.357	2281	-1.198319451	-2.866475189	0.386217743
174070	3/2/2019 1:56	B	41978020.66	20589056.34	-73.461	-44.386	2129	-1.30703467	-2.866475189	0.345252655

174070	3/2/2019 2:22	B	8914782.713	1458037.287	-73.478	-44.376	1553	-1.320254063	-2.687405071	0.340838635
174070	3/2/2019 2:35	B	4257571.458	811493.542	-73.486	-44.38	794	-1.329887346	-2.59611424	0.336002669
174070	3/2/2019 2:58	B	7365362.359	3108486.141	-73.511	-44.404	1398	-1.358796691	-2.314063043	0.322364666
174070	3/2/2019 3:43	1	343844.3948	1321468.105	-73.513	-44.409	2680	-1.363919788	-2.252920624	0.320042153
174070	3/2/2019 4:02	B	4557917.971	1518167.029	-73.512	-44.409	1144	-1.36358707	-2.274833287	0.320632621
174070	3/2/2019 4:39	A	231132.5604	282049.9396	-73.47	-44.442	2216	-1.365523836	-2.850618905	0.3204756
174070	3/2/2019 6:36	0	238742989.6	389014690.9	-73.426	-44.449	6995	-1.353687964	-2.856281864	0.319908882
174070	3/2/2019 8:15	A	3215811871	909199210.4	-73.358	-44.573	5970	-1.45715921	-2.866475189	0.300368482
174070	3/2/2019 9:33	B	126980779.9	351166602.6	-73.52	-44.548	4703	-1.448977711	-2.319221815	0.297642249
174070	3/2/2019 10:01	A	344616.2208	221390.2792	-73.514	-44.505	1666	-1.4356986	-2.041924029	0.301034802
174070	3/2/2019 11:08	B	16019881.22	5941157.276	-73.521	-44.569	4000	-1.457550825	-2.137135265	0.294105684
174070	3/2/2019 12:41	0	1595447072	76832	-73.358	-44.591	5581	-1.459855966	-1.405369397	0.301602035
174070	3/2/2019 12:53	B	5008416.641	920549.8585	-73.363	-44.59	726	-1.45712206	-1.3612695	0.30229696
174070	3/2/2019 15:01	1	4311956.768	780701.232	-73.542	-44.444	7700	-1.399154568	-2.118456918	0.30672725
174070	3/2/2019 19:44	B	10134955.59	2720120.414	-73.38	-44.414	16975	-1.212422178	-2.866475189	0.355571504
174070	3/2/2019 22:11	0	82969313555	3134923366	-73.574	-44.393	8800	-1.3377918	-2.42465195	0.331822688

174070	3/2/2019 23:04	A	192701256	206632466.5	-73.541	-44.36	3173	-1.343843819	-1.489699534	0.318127919
174070	3/2/2019 23:24	B	5740934.646	78166.35406	-73.542	-44.359	1213	-1.341191509	-1.503394935	0.317872958
174070	3/3/2019 0:00	B	11654876.37	32215546.13	-73.497	-44.361	2147	-1.14348823	-2.354694862	0.433651603
174070	3/3/2019 0:32	A	669121.4926	1359752.507	-73.477	-44.348	1908	-1.145162196	-2.549348778	0.4401067
174070	3/3/2019 0:59	0	4809300.25	4809300.25	-73.478	-44.363	1639	-1.15903807	-2.591630198	0.449201891
174070	3/3/2019 1:35	0	97691324.49	7205207.507	-73.479	-44.373	2142	-1.168797119	-2.631164479	0.455610185
174070	3/3/2019 2:16	A	1956174816	319330926.8	-73.475	-44.375	2460	-1.168958292	-2.515671073	0.452992631
174070	3/3/2019 2:41	0	1633409898	73799830.85	-73.504	-44.39	1529	-1.173717584	-2.534854164	0.453546033
174070	3/3/2019 3:12	1	24125407.96	2000754.538	-73.501	-44.41	1866	-1.187951906	-2.531394497	0.460728256
174070	3/3/2019 3:49	B	37479674.35	9632590.649	-73.496	-44.421	2189	-1.200368298	-2.705460449	0.469206126
174070	3/3/2019 4:17	B	47258533.97	15312868.53	-73.497	-44.42	1711	-1.199509951	-2.705460449	0.468807516
174070	3/3/2019 6:23	B	10126882.57	2220429.928	-73.478	-44.445	7571	-1.231287303	-2.800345303	0.491832853
174070	3/3/2019 8:04	B	239756785.9	10836148.59	-73.506	-44.334	6035	-1.110925623	-1.893319958	0.408267291
174070	3/3/2019 9:11	B	11989426.46	583794.0411	-73.504	-44.326	4026	-1.103322478	-1.809506493	0.405489708
174070	3/3/2019 9:44	A	2754808.178	4818904.322	-73.425	-44.298	1994	-1.123277972	-2.866475189	0.44163674
174070	3/3/2019 10:44	B	478155841.6	163163.356	-73.392	-44.364	3588	-1.213263237	-2.866475189	0.504019982

174070	3/3/2019 10:46	B	93378673.65	68482.84741	-73.395	-44.364	103	-1.214453359	-2.866475189	0.505309645
174070	3/3/2019 12:03	B	88599982.62	19993403.38	-73.254	-44.41	4637	-1.325132471	-1.443918025	0.58809425
174070	3/3/2019 12:21	B	90978874.27	6310788.229	-73.243	-44.44	1085	-1.351507527	-1.388703615	0.601214224
174070	3/3/2019 12:50	B	27406473.97	4269976.031	-73.217	-44.409	1708	-1.342098862	-1.105616419	0.601097774
174070	3/3/2019 13:37	B	325504868.6	7823279.925	-73.242	-44.394	2863	-1.30777117	-1.555771564	0.579905096
174070	3/3/2019 14:43	B	164894474.9	546382.1203	-73.462	-44.338	3944	-1.152403228	-2.802145921	0.453370861
174070	3/3/2019 20:23	B	19809827.95	81225924.55	-73.54	-44.382	20391	-1.136613966	-1.575136041	0.416007559
174070	3/3/2019 22:31	1	3234165.735	480246.7653	-73.499	-44.359	7679	-1.141348643	-2.295643285	0.431376822
174070	3/3/2019 23:00	B	1324370.33	402071.6703	-73.477	-44.37	1721	-1.166044818	-2.639278641	0.454540512
174070	3/3/2019 23:46	B	46923774.66	24773675.84	-73.497	-44.357	2789	-1.145637027	-2.371754206	0.435908645
174070	3/4/2019 0:10	A	1045110.98	554963.5199	-73.474	-44.379	1434	-1.126187889	-2.715995746	0.407846079
174070	3/4/2019 0:15	A	45512.63276	741549.8672	-73.469	-44.371	313	-1.122790039	-2.773039276	0.407150272
174070	3/4/2019 0:37	B	194447708.7	59818689.8	-73.459	-44.367	1299	-1.120179133	-2.866475189	0.408616625
174070	3/4/2019 1:18	B	348518223.7	62989648.29	-73.465	-44.367	2482	-1.117017749	-2.768514286	0.404160193
174070	3/4/2019 2:04	A	14243963.21	1507726.787	-73.471	-44.359	2732	-1.108039645	-2.735860652	0.396560011
174070	3/4/2019 2:19	1	14950209.2	125009.3025	-73.457	-44.358	928	-1.114654395	-2.866475189	0.404871555

174070	3/4/2019 2:23	B	3180170.597	405473.9025	-73.458	-44.358	252	-1.115993088	-2.866475189	0.406365638
174070	3/4/2019 3:01	B	40972184.93	2541508.068	-73.461	-44.371	2255	-1.126856304	-2.866475189	0.412627937
174070	3/4/2019 3:35	B	228830224.5	6612.5	-73.457	-44.35	2045	-1.106909687	-2.82358901	0.399355741
174070	3/4/2019 4:02	B	8640071.058	2248979.442	-73.461	-44.35	1629	-1.103657676	-2.822057361	0.396077493
174070	3/4/2019 4:35	B	1597464.571	63044.42936	-73.46	-44.348	1948	-1.102988219	-2.82358901	0.396148931
174070	3/4/2019 9:35	2	185375.9415	118394.0585	-73.373	-44.392	18032	-1.197267933	-2.866475189	0.484679614
174070	3/4/2019 10:18	2	249865.625	93496.875	-73.428	-44.388	2562	-1.162800883	-2.866475189	0.447946622
174070	3/4/2019 10:39	A	6431.304842	144879.1952	-73.427	-44.365	1287	-1.139409599	-2.866475189	0.43131014
174070	3/4/2019 11:13	B	901824.5	245700.5	-73.424	-44.362	2029	-1.139721835	-2.866475189	0.432249863
174070	3/4/2019 11:52	A	899072534.7	283725.7972	-73.392	-44.378	2314	-1.169601316	-2.866475189	0.460723098
174070	3/4/2019 12:18	A	2348015.568	11738.93242	-73.407	-44.368	1581	-1.155281247	-2.866475189	0.449015552
174070	3/4/2019 12:38	1	499338.543	303231.457	-73.449	-44.36	1218	-1.122712456	-2.866475189	0.413481463
174070	3/4/2019 13:17	B	1145059.408	294416.5915	-73.4	-44.369	2310	-1.160353514	-2.866475189	0.45498609
174070	3/4/2019 13:42	1	35491947.89	2222041.114	-73.436	-44.364	1523	-1.138608345	-2.866475189	0.42959872
174070	3/4/2019 14:20	A	2139643.218	1770461.282	-73.438	-44.343	2251	-1.111159154	-2.843443722	0.408957458
174070	3/4/2019 15:27	B	2377789.662	602307.3384	-73.433	-44.334	4003	-1.10676466	-2.822057361	0.407066941

174070	3/4/2019 20:09	A	357887.5297	4674705.47	-73.458	-44.297	16929	-1.042585418	-2.592794932	0.353674838
174070	3/4/2019 20:21	A	776714.1292	832258.3708	-73.47	-44.285	754	-1.017897646	-2.435586609	0.330174158
174070	3/4/2019 21:54	B	94723477.88	11237334.62	-73.515	-44.336	5560	-1.050562592	-1.650879509	0.334944014
174070	3/4/2019 22:38	B	16945098.28	3303104.215	-73.559	-44.367	2617	-1.058158152	-1.313442883	0.327111283
174070	3/4/2019 23:35	B	7027778.957	3957247.043	-73.431	-44.413	3419	-1.180005527	-2.866475189	0.458710464
174070	3/5/2019 0:08	A	1721974.472	8644468.028	-73.422	-44.428	1998	-1.272447116	-2.866475189	0.339720311
174070	3/5/2019 0:16	A	41996623.94	1061877.061	-73.417	-44.448	470	-1.285831914	-2.866475189	0.346929381
174070	3/5/2019 2:03	A	377010246	31009484.04	-73.364	-44.475	6418	-1.321748641	-2.866475189	0.374119547
174070	3/5/2019 2:29	1	140376893	1557043.508	-73.376	-44.459	1589	-1.316707335	-2.866475189	0.372637989
174070	3/5/2019 3:06	A	1733408802	53896790.38	-73.339	-44.449	2206	-1.322661238	-2.833460047	0.37969533
174070	3/5/2019 3:32	A	12446681.07	2247679.433	-73.341	-44.454	1555	-1.326604032	-2.792191119	0.382742361
174070	3/5/2019 3:36	A	2267342.415	104914.0849	-73.344	-44.451	266	-1.326604032	-2.789629599	0.382742361
174070	3/5/2019 4:17	A	510615.838	39484.16197	-73.333	-44.456	2446	-1.334220879	-2.783937334	0.38801175
174070	3/5/2019 7:44	A	541717.4314	1898462.569	-73.287	-44.449	12440	-1.351513089	-2.328771782	0.403995714
174070	3/5/2019 9:19	A	165866.4993	17135.5007	-73.357	-44.455	5700	-1.321077937	-2.850557174	0.377670959
174070	3/5/2019 9:58	B	768704.6731	1107395.327	-73.367	-44.453	2318	-1.316040489	-2.866475189	0.373976137

174070	3/5/2019 10:07	B	2328319.5	1258509.5	-73.36	-44.446	544	-1.314233346	-2.866475189	0.373982605
174070	3/5/2019 11:01	B	4404143.794	8824873.206	-73.309	-44.458	3228	-1.345566461	-2.717907049	0.397885743
174070	3/5/2019 11:37	B	50946536.48	1292113.524	-73.292	-44.432	2146	-1.336114498	-2.268653692	0.396141397
174070	3/5/2019 12:20	A	4212118.444	3944226.556	-73.421	-44.398	2596	-1.250308017	-2.866475189	0.329267298
174070	3/5/2019 13:57	0	476329012.7	124035879.8	-73.293	-44.469	5801	-1.35320505	-2.747053869	0.401432936
174070	3/5/2019 15:43	B	246783097.2	53578741.31	-73.231	-44.528	6412	-1.402038479	-1.340898558	0.427039474
174070	3/5/2019 21:40	B	33172358.79	30936842.21	-73.401	-44.305	21392	-1.176720552	-2.866475189	0.290820044
174070	3/5/2019 23:07	B	9911803.792	158752348.7	-73.363	-44.357	5244	-1.241156476	-2.866475189	0.337552164
174070	3/5/2019 23:51	1	4880479.25	196217.2504	-73.364	-44.327	2637	-1.219235268	-2.866475189	0.324174851
174070	3/6/2019 0:34	B	2108217.534	580544.9658	-73.314	-44.306	2567	-1.016492093	-2.866475189	-0.475813663
174070	3/6/2019 1:36	B	73970582.66	7814402.343	-73.312	-44.308	3738	-1.004242034	-2.866475189	-0.479979603
174070	3/6/2019 2:13	A	184023.698	16738.80196	-73.371	-44.303	2214	-0.779061912	-2.866475189	-0.547284002
174070	3/6/2019 2:46	B	5123200.5	1256112.5	-73.377	-44.311	1946	-0.767745589	-2.866475189	-0.574028826
174070	3/6/2019 3:17	B	9350905.697	313740.8031	-73.375	-44.31	1856	-0.768606319	-2.866475189	-0.567653991
174070	3/6/2019 3:59	B	889054.3686	5976850.131	-73.389	-44.318	2550	-0.772214852	-2.866475189	-0.6004657
174070	3/6/2019 7:31	A	323131.0143	703507.4857	-73.409	-44.379	12722	-1.054809689	-2.866475189	-0.645701455

174070	3/6/2019 9:09	B	685709268.1	30980819.89	-73.42	-44.401	5843	-1.107153038	-2.866475189	-0.644790577
174070	3/6/2019 9:32	B	6442352.355	309636.1448	-73.419	-44.401	1408	-1.104419693	-2.866475189	-0.64820015
174070	3/6/2019 9:41	B	4741520.033	220748.9672	-73.428	-44.405	552	-1.099460426	-2.866475189	-0.656556746
174070	3/6/2019 11:14	B	6779141.676	300594.8236	-73.432	-44.42	5570	-1.121726993	-2.866475189	-0.651119137
174070	3/6/2019 12:52	B	975820.5	2549236.5	-73.411	-44.458	5889	-1.188129061	-2.866475189	-0.614189716
174070	3/6/2019 13:06	B	1143996.347	2017864.653	-73.411	-44.459	850	-1.188568713	-2.866475189	-0.614220296
174070	3/6/2019 14:17	B	1955422.533	1099113.467	-73.414	-44.49	4210	-1.226798141	-2.866475189	-0.598822811
174070	3/6/2019 14:44	B	2401605.472	1131335.528	-73.414	-44.478	1624	-1.210123272	-2.866475189	-0.606197952
174070	3/6/2019 21:29	A	216059.2216	19425.77843	-73.466	-44.439	24320	-1.106632991	-2.853096449	-0.665886962
174070	3/6/2019 22:35	B	262559486.2	2033280.332	-73.483	-44.385	3967	-1.03139931	-2.866475189	-0.700153764
174070	3/6/2019 23:13	A	1317068.284	4165236.216	-73.384	-44.377	2293	-1.053667033	-2.866475189	-0.615764387
174070	3/6/2019 23:23	B	20272696.83	5272129.173	-73.384	-44.365	598	-1.000736609	-2.866475189	-0.610925544
174070	3/7/2019 0:15	B	2076657.515	40177426.98	-73.322	-44.349	3100	0.198282106	-2.866475189	-0.311758485
174070	3/7/2019 1:05	2	604275.4048	10806.59517	-73.324	-44.346	3018	0.185536726	-2.866475189	-0.312664524
174070	3/7/2019 1:19	A	94345.49368	33077.00632	-73.315	-44.344	818	0.133217477	-2.866475189	-0.268728135
174070	3/7/2019 1:26	1	31183072.54	1185139.958	-73.316	-44.339	404	0.12661091	-2.866475189	-0.260536425

174070	3/7/2019 3:02	B	26953586.62	6486237.878	-73.272	-44.308	5810	-0.24650768	-2.499140648	-0.100178462
174070	3/7/2019 3:11	A	974617.041	910126.959	-73.293	-44.297	492	-0.427367496	-2.724938478	-0.144746175
174070	3/7/2019 3:38	3	121783.8768	5961.123166	-73.317	-44.325	1639	-0.066834653	-2.866475189	-0.230388602
174070	3/7/2019 4:36	A	6616359.252	1326639.248	-73.325	-44.334	3459	0.13935011	-2.866475189	-0.281844389
174070	3/7/2019 7:21	B	136259815.5	71930646.95	-73.283	-44.313	9928	-0.284157918	-2.529206274	-0.12673579
174070	3/7/2019 9:07	A	132512.3067	5474992.693	-73.355	-44.362	6368	-0.087895714	-2.866475189	-0.472016302
174070	3/7/2019 10:47	3	135679.5732	1392.92683	-73.408	-44.459	5977	0.046947048	-2.866475189	-0.405198873
174070	3/7/2019 11:02	B	395160.5	92880.5	-73.411	-44.464	924	0.076819067	-2.866475189	-0.38578258
174070	3/7/2019 11:18	0	24668874.57	21480765.43	-73.371	-44.458	946	0.427838219	-2.866475189	-0.346442705
174070	3/7/2019 11:36	B	24480557.68	7518715.319	-73.374	-44.463	1110	0.470277425	-2.866475189	-0.330850563
174070	3/7/2019 12:27	B	63757683.53	15639404.97	-73.379	-44.471	3003	0.480629136	-2.866475189	-0.305130617
174070	3/7/2019 13:49	B	48166246.23	13117881.77	-73.396	-44.499	4956	0.214194573	-2.866475189	-0.21659682
174070	3/7/2019 14:23	A	4678906.419	99744054.58	-73.393	-44.423	2031	-0.343592382	-2.866475189	-0.488067892
174070	3/7/2019 14:35	B	4392748.354	12575021.65	-73.394	-44.424	704	-0.565299526	-2.866475189	-0.513204095
174070	3/7/2019 20:27	0	69455186.48	39548615.52	-73.486	-44.33	21148	-0.735009567	-2.254326729	-0.689834457
174070	3/7/2019 21:17	B	16689168.72	2407032.276	-73.507	-44.348	3018	-0.481048597	-2.000059044	-0.672824784

174070	3/7/2019 22:04	B	612737927.5	23159514.96	-73.48	-44.331	2780	-0.811801964	-2.340393142	-0.679909478
174070	3/7/2019 22:56	0	171335992	10680550.51	-73.447	-44.358	3114	-0.758396154	-2.866475189	-0.635732597
174070	3/7/2019 23:41	B	89860448.2	1979071.796	-73.475	-44.432	2734	-0.608425828	-2.846348346	-0.468168719
174070	3/8/2019 0:39	2	229267.781	20774.71904	-73.448	-44.451	3485	-0.655892865	-2.845333477	-0.283806212
174070	3/8/2019 0:59	B	3220275.565	775509.4349	-73.45	-44.462	1184	-0.678763231	-2.822186949	-0.274833348
174070	3/8/2019 1:14	A	51939.43192	4413.068082	-73.422	-44.47	911	-0.011954265	-2.861189761	-0.246690175
174070	3/8/2019 1:28	B	309684.5	71064.5	-73.423	-44.478	854	-0.005054563	-2.855904333	-0.24314937
174070	3/8/2019 2:35	B	535523773.5	24195372.96	-73.42	-44.539	3969	-0.006703322	-2.866475189	-0.190986959
174070	3/8/2019 3:09	A	7475677.65	27126.85007	-73.459	-44.571	2066	-0.151264925	-2.510716895	-0.262432456
174070	3/8/2019 3:45	B	11279730.15	2099966.851	-73.466	-44.593	2132	-0.357265843	-1.958479525	-0.311770374
174070	3/8/2019 4:12	A	113752.4736	22344.52645	-73.529	-44.619	1658	0.380537456	-1.215304847	-0.108969376
174070	3/8/2019 4:55	B	1160593.999	1322835.001	-73.51	-44.657	2578	1.628373066	-0.833359596	-0.051888715
174070	3/8/2019 7:05	1	361034.4643	602908.0357	-73.502	-44.723	7816	0.94851133	-0.74022945	0.109709821
174070	3/8/2019 8:44	B	4237216.333	1157609.667	-73.492	-44.784	5930	2.616998005	-1.504178849	0.338317742
174070	3/8/2019 10:11	B	1761538.205	879806.295	-73.46	-44.85	5193	1.030379273	-2.760121951	0.813966155
174070	3/8/2019 11:05	B	3384646.931	5649183.569	-73.457	-44.88	3244	-0.168073444	-2.866475189	0.82562953

174070	3/8/2019 12:02	B	3153288.493	897822.0075	-73.459	-44.896	3452	-0.190841166	-2.755506719	0.777272584
174070	3/8/2019 12:20	B	3657886.769	929491.7313	-73.455	-44.894	1028	-0.1704286	-2.795047208	0.784187278
174070	3/8/2019 12:43	B	393538944.2	14871993.76	-73.462	-44.916	1429	-0.433903398	-2.399635586	0.723921576
174070	3/8/2019 13:36	B	15201193.96	628920.04	-73.45	-44.952	3159	0.677814884	-1.758922133	0.674892818
174070	3/8/2019 13:58	B	206006457.6	10989314.85	-73.45	-44.97	1339	1.766709761	-1.63937977	0.573731939
174070	3/8/2019 14:21	B	7984008	40328	-73.454	-44.969	1335	1.767497229	-1.706778303	0.577906709
174070	3/8/2019 21:35	1	409103.0753	71141.42466	-73.588	-44.976	26083	0.907117039	-2.756008989	-0.516080121
174070	3/8/2019 22:33	0	5359065.295	12958395.71	-73.587	-44.962	3453	1.486762823	-2.802149639	-0.480381899
174070	3/8/2019 22:42	B	5883148.354	3517821.646	-73.59	-44.961	526	1.424900673	-2.769641028	-0.491227029
174070	3/8/2019 23:16	2	167409.4503	12640.54969	-73.613	-44.973	2056	-0.131416509	-2.76296281	-0.635424712
174070	3/9/2019 0:15	0	694284296.5	64947403.5	-73.688	-45.007	3552	0.594040794	-1.321607515	-1.011897544
174070	3/9/2019 0:36	0	14414035.73	30884174.27	-73.68	-44.984	1263	0.323632514	-1.466015952	-1.005964311
174070	3/9/2019 1:00	A	272271255.1	123893913.9	-73.618	-44.995	1424	0.633836536	-2.122080002	-0.746108235
174070	3/9/2019 1:13	B	18563557.12	10919978.88	-73.601	-45.013	784	0.491044981	-1.737536158	-0.575717663
174070	3/9/2019 2:12	1	4250997.752	267975.2477	-73.604	-45.055	3545	0.409434289	-1.101821799	-0.512271776
174070	3/9/2019 2:48	A	273267125.3	14569999.67	-73.609	-45.065	2178	1.249209537	-0.933628171	-0.583355529

174070	3/9/2019 2:52	A	2715153.941	58952.55947	-73.613	-45.065	215	1.249209537	-0.910542404	-0.583355529
174070	3/9/2019 3:21	B	8066350.334	1056898.166	-73.616	-45.066	1753	1.249209537	-0.891507361	-0.583355529
174070	3/9/2019 3:59	B	5543034.008	1655490.992	-73.617	-45.086	2277	1.696000895	-0.742125778	-0.615621434
174070	3/9/2019 4:22	A	762911.3103	12241697.69	-73.633	-45.102	1385	1.906191166	-0.629715517	-0.821238518
174070	3/9/2019 4:36	A	15602.94361	45098.05639	-73.63	-45.104	803	1.88620571	-0.618103013	-0.794184033
174070	3/9/2019 6:52	B	1631890	1631890	-73.672	-45.152	8197	-0.405670361	-0.38529026	-0.980926421
174070	3/9/2019 8:36	B	3346448.984	4891199.516	-73.671	-45.178	6222	-0.324350932	-0.296838363	-0.917427601
174070	3/9/2019 9:45	B	190339272	81902724.96	-73.601	-45.144	4175	1.855948704	-0.452516655	-1.008872179
174070	3/9/2019 10:12	A	1122995.375	3342417.125	-73.585	-45.12	1569	3.332295787	-0.550247794	-0.69596282
174070	3/9/2019 10:54	B	4306950.207	3493014.293	-73.593	-45.128	2562	3.179564601	-0.510584741	-0.800972348
174070	3/9/2019 11:36	A	1753779.549	13870.45118	-73.596	-45.07	2498	0.430702026	-0.870681307	-0.475395719
174070	3/9/2019 12:03	B	7065321.574	266300.9264	-73.594	-45.052	1598	-0.109339863	-1.136715297	-0.476414448
174070	3/9/2019 12:32	A	218528.1461	2905.853933	-73.622	-45.073	1770	1.860216447	-0.848228047	-0.662114813
174070	3/9/2019 12:41	B	3330436.569	761781.4314	-73.6	-45.057	524	0.279387967	-1.021848179	-0.500170552
174070	3/9/2019 13:03	A	3543586.055	2191754.445	-73.568	-45.057	1306	-0.366714553	-1.029860212	-0.434446739
174070	3/9/2019 13:35	B	49736811.24	12235572.76	-73.588	-45.086	1933	0.728416464	-0.755709943	-0.424351477

174070	3/9/2019 14:13	A	471784864.9	62920232.1	-73.6	-45.078	2271	0.959835529	-0.786806764	-0.500498442
174070	3/9/2019 15:58	B	13253149.43	1503509.071	-73.617	-45.14	6314	1.374892373	-0.459438796	-0.968096647
174070	3/9/2019 20:51	1	1624490.77	86373.2304	-73.591	-45.034	17595	-0.241090212	-1.370954332	-0.489094056
174070	3/9/2019 21:05	1	17196821.8	1273170.699	-73.611	-45.025	839	0.436677927	-1.482238543	-0.555094848
174070	3/9/2019 22:32	B	36988382.29	8911822.713	-73.61	-45.033	5185	0.475614352	-1.377442368	-0.542400266
174070	3/9/2019 22:43	B	53916833.74	94913088.26	-73.607	-45.037	704	0.284918984	-1.344338497	-0.523086169
174070	3/9/2019 23:49	B	1791125.157	208638.8428	-73.605	-45.051	3949	0.368733938	-1.127120975	-0.510659364
174070	3/10/2019 0:16	B	1840848.012	421794.488	-73.606	-45.047	1613	-0.036658924	-1.176824074	-0.616060146
174070	3/10/2019 0:49	B	2111076.413	680214.087	-73.613	-45.048	1978	0.051013661	-1.167345885	-0.644374873
174070	3/10/2019 1:33	1	7079380.731	5358907.269	-73.616	-45.08	2643	0.324577076	-0.79118221	-0.678124816
174070	3/10/2019 2:29	B	12583886	37688154	-73.619	-45.096	3375	0.317928258	-0.68064211	-0.716748926
174070	3/10/2019 3:09	B	18647667.89	370657.1118	-73.618	-45.121	2368	-0.030564029	-0.552630133	-0.771779515
174070	3/10/2019 3:34	B	2048106.782	121642.2176	-73.62	-45.12	1503	-0.032657651	-0.556011464	-0.76972083
174070	3/10/2019 4:13	B	267741.7439	3025220.756	-73.603	-45.133	2361	0.16879446	-0.489634636	-0.781691868
174070	3/10/2019 6:43	B	23065893.23	37637095.77	-73.669	-45.077	8964	0.012521336	-0.75485822	-0.917011685
174070	3/10/2019 8:16	B	6115886.845	4065413.155	-73.666	-45.083	5638	-0.033078289	-0.735675996	-0.918608504

174070	3/10/2019 9:35	B	10892924.68	7085775.822	-73.639	-45.065	4682	0.336357799	-0.934953015	-0.760543167
174070	3/10/2019 10:07	A	1624104	562536	-73.557	-45.035	1948	-0.13392929	-1.393341238	-0.431193699
174070	3/10/2019 10:55	A	113168044.5	2839835.515	-73.607	-45.073	2893	0.188877379	-0.851602442	-0.613919813
174070	3/10/2019 11:07	B	5642049.167	144064.8335	-73.605	-45.073	685	0.195772439	-0.847478167	-0.618024032
174070	3/10/2019 12:29	B	52347911.99	6989728.009	-73.496	-44.993	4949	-0.081378433	-2.175476934	-0.161374512
174070	3/10/2019 12:55	B	12603461.71	1746404.793	-73.5	-44.992	1582	-0.054333402	-2.256564886	-0.168641616
174070	3/10/2019 13:59	0	18396235505	90950784.53	-73.559	-45.004	3802	0.228220382	-2.082461409	-0.365778529
174070	3/10/2019 14:32	B	5194636.562	11072835.44	-73.564	-44.993	1988	0.428388947	-2.31136457	-0.451234706
174070	3/10/2019 15:41	B	72153712.34	1457168.663	-73.546	-45.03	4152	-0.069282243	-1.401197462	-0.40008366
174070	3/10/2019 20:44	B	3175411.249	44202427.25	-73.498	-44.819	18192	-0.944424383	-2.238386438	0.07623448
174070	3/10/2019 22:17	0	76889251.39	42558933.61	-73.407	-44.741	5587	-0.712644696	-0.843775325	-0.163442739
174070	3/10/2019 23:50	B	2678016130	1885685035	-73.514	-44.593	5551	-0.202599811	-1.351573906	-0.230965875
174070	3/11/2019 0:06	B	99081946.69	47288925.81	-73.52	-44.598	949	1.409649892	-1.569213257	0.13686068
174070	3/11/2019 0:34	B	7652607.575	5354552.925	-73.52	-44.569	1673	1.515148016	-2.137135265	0.18361999
174070	3/11/2019 1:05	0	138491422.3	97662002.74	-73.493	-44.549	1851	1.492975355	-2.641738745	-0.008991219
174070	3/11/2019 1:32	B	125174990.1	1400902.354	-73.491	-44.513	1624	0.273218036	-2.495610415	0.03486948

174070	3/11/2019 2:08	B	100027605.4	10180387.08	-73.483	-44.482	2155	-0.387259012	-2.517946687	0.086060895
174070	3/11/2019 2:20	A	3213563.965	300757.0348	-73.454	-44.467	721	-0.165692132	-2.814149453	0.125075786
174070	3/11/2019 2:44	B	24578597.31	188965.1943	-73.446	-44.449	1479	-0.068745986	-2.851185201	0.146585249
174070	3/11/2019 3:12	A	38371.33196	17342.66804	-73.443	-44.451	1645	-0.070410856	-2.851712442	0.156984303
174070	3/11/2019 3:57	A	32798.01512	3942.484884	-73.441	-44.444	2750	-0.051992229	-2.861378526	0.159651851
174070	3/11/2019 4:53	B	27351868.61	3466717.89	-73.383	-44.357	3310	0.182647013	-2.866475189	0.339043765
174070	3/11/2019 6:30	B	36222589.66	5455988.338	-73.336	-44.293	5844	-0.086866664	-2.866475189	0.620673879
174070	3/11/2019 8:14	B	320890985.5	225955373	-73.405	-44.356	6219	0.234732906	-2.866475189	0.302692161
174070	3/11/2019 9:08	B	2448125	2448125	-73.395	-44.318	3277	0.725861501	-2.866475189	0.404750007
174070	3/11/2019 9:51	B	24420.5	243321800	-73.448	-44.337	2586	0.205022974	-2.759259742	0.142756614
174070	3/11/2019 10:45	A	21210519.31	245885.693	-73.473	-44.343	3232	-0.085730745	-2.598563117	0.073945884
174070	3/11/2019 12:03	A	651868.3752	418990.1248	-73.446	-44.441	4641	-0.017582512	-2.861378526	0.140681285
174070	3/11/2019 12:04	B	1883366.075	850579.925	-73.447	-44.439	88	-0.017897028	-2.860986475	0.139759885
174070	3/11/2019 13:37	A	93867556.74	2925548.264	-73.571	-44.427	5576	0.398999769	-1.60627028	-0.105783791
174070	3/11/2019 13:49	B	6778056.517	1313333.983	-73.563	-44.422	730	0.468692452	-1.535028089	-0.127087716
174070	3/11/2019 14:40	B	42056187.89	6591880.612	-73.639	-44.424	3050	0.152769542	-0.914324245	-0.541232613

174070	3/11/2019 21:45	A	10159213836	6201986186	-73.128	-44.275	25487	0.769083271	-2.532624893	0.591287164
174070	3/11/2019 22:08	B	2250409226	1281935686	-73.132	-44.271	1403	0.864934173	-2.093914855	0.500310737
174070	3/12/2019 0:39	A	2101645.116	310362.8838	-73.338	-43.981	9079	0.659140402	-1.081940403	0.053772867
174070	3/12/2019 1:11	0	2373890.327	37376632.17	-73.372	-43.986	1863	1.125295322	-1.076514995	0.233037514
174070	3/12/2019 1:47	B	31158901.82	11856843.18	-73.335	-43.987	2178	0.823914547	-1.136799411	0.093787348
174070	3/12/2019 2:05	1	156669578.9	8353030.1	-73.391	-43.978	1115	0.816617825	-1.000907062	0.186276991
174070	3/12/2019 2:18	B	17618106.75	955423.248	-73.37	-43.972	737	0.626747565	-0.958105548	0.154778826
174070	3/12/2019 2:28	B	52138078.53	3183975.971	-73.364	-43.975	644	0.653779688	-0.993929322	0.133086156
174070	3/12/2019 2:54	B	176418	4084082	-73.325	-43.97	1563	0.16175606	-1.043886972	-0.022570765
174070	3/12/2019 3:28	B	861945.0019	525291.4981	-73.309	-43.965	2020	-0.239609727	-1.043446885	-0.076425645
174070	3/12/2019 3:46	B	34850636.95	27725.55355	-73.32	-43.961	1065	-0.105715763	-0.999891716	-0.03536048
174070	3/12/2019 4:33	0	16575308.16	6708036.343	-73.444	-43.979	2834	2.913838851	-0.78269887	0.3080059
174070	3/12/2019 8:01	A	108038363.8	7775000.169	-73.417	-43.956	12481	1.820128386	-0.714286426	0.119906771
174070	3/12/2019 9:35	B	36957447.61	6865005.387	-73.469	-43.993	5659	2.734573011	-0.836919654	0.433233844
174070	3/12/2019 10:14	B	17751478.87	2966899.634	-73.473	-43.996	2339	2.704586394	-0.875257057	0.440277866
174070	3/12/2019 10:27	B	58364215.04	29659457.46	-73.433	-43.994	740	2.570142163	-0.890863091	0.49830848

174070	3/12/2019 11:30	A	1293340.587	705701.4135	-73.433	-43.98	3766	2.652465674	-0.812048423	0.329219372
174070	3/12/2019 11:58	B	3744287.248	748289.7524	-73.442	-43.988	1724	2.79211113	-0.846148597	0.413343393
174070	3/12/2019 12:32	B	1379724.728	240805.7719	-73.44	-43.987	2045	2.784578352	-0.841511551	0.405711872
174070	3/12/2019 13:13	B	19976166.48	3267390.516	-73.466	-44.025	2443	0.796802225	-1.15802664	0.671762192
174070	3/12/2019 14:19	B	3098940.017	18935466.48	-73.475	-44.022	3953	0.778193251	-1.098919306	0.635930529
174070	3/12/2019 14:53	A	16629111.85	1227250.149	-73.448	-44.075	2046	0.982969255	-1.953107004	0.813048927
174070	3/12/2019 21:09	1	766555.3612	161254.6388	-73.456	-44.167	22552	0.530118007	-2.102734954	0.777054377
174070	3/12/2019 21:56	A	2574556.11	3494048.89	-73.441	-44.207	2816	0.92269322	-2.783805249	0.779883168
174070	3/12/2019 22:48	B	45766368.04	8270138.461	-73.441	-44.234	3154	1.472454139	-2.851363297	0.713503966
174070	3/12/2019 23:44	B	7111917.004	993171.4961	-73.439	-44.267	3315	2.851315747	-2.80137781	0.543759043
174070	3/13/2019 0:15	1	952116.2842	45084.21584	-73.435	-44.269	1893	0.684043207	-2.81005746	0.431095557
174070	3/13/2019 1:26	A	185753.8749	1850418.625	-73.448	-44.261	4230	0.749451523	-2.760691948	0.424956481
174070	3/13/2019 1:58	B	5637829.067	3026245.433	-73.447	-44.268	1954	0.785384474	-2.7395353	0.412653486
174070	3/13/2019 2:00	B	81538161.45	4355538.554	-73.448	-44.264	87	0.770224104	-2.749748854	0.418149415
174070	3/13/2019 2:27	1	15722231.51	841830.9871	-73.443	-44.257	1662	0.500192749	-2.795953028	0.475294153
174070	3/13/2019 3:08	B	391415708.7	2997393.797	-73.448	-44.255	2451	0.53696013	-2.790915731	0.467068364

174070	3/13/2019 3:41	1	108240.6337	323419.8663	-73.452	-44.243	1936	0.305105173	-2.819460415	0.504737968
174070	3/13/2019 4:17	B	3759282	998284.5	-73.456	-44.243	2194	0.364755771	-2.793521229	0.494396114
174070	3/13/2019 9:16	B	23081163.22	4557961.784	-73.47	-44.237	17960	0.383995085	-2.817652154	0.479054393
174070	3/13/2019 9:26	B	1258316709	47545485.26	-73.467	-44.248	543	0.619423461	-2.733824334	0.438672731
174070	3/13/2019 10:57	B	16584774.04	994051.9586	-73.486	-44.25	5481	0.933001917	-2.53568253	0.35296351
174070	3/13/2019 11:37	B	37645.72189	17454606.78	-73.51	-44.244	2413	1.006319395	-2.485294873	0.329899173
174070	3/13/2019 12:16	B	757703.2419	218287.2581	-73.52	-44.263	2333	1.119037893	-1.971095227	0.171063125
174070	3/13/2019 12:54	1	1431441.865	355758.1352	-73.525	-44.218	2312	0.087174785	-2.232619729	0.463007079
174070	3/13/2019 13:25	B	2039893.82	331602.18	-73.533	-44.213	1807	-0.00745924	-2.021009911	0.469781542
174070	3/13/2019 13:59	B	2515305.18	863063.3198	-73.537	-44.206	2051	-0.2624575	-1.839898366	0.493070756
174070	3/13/2019 14:32	A	486839296.5	3795059.98	-73.477	-44.271	2013	1.056566635	-2.12428703	0.189802294
174070	3/13/2019 15:38	B	82199831.72	8647938.781	-73.456	-44.29	3910	1.001310715	-2.616693971	0.232220009
174070	3/13/2019 20:38	B	161571095.3	17193747.16	-73.445	-44.268	18015	0.852963048	-2.730468165	0.399199949
174070	3/13/2019 21:52	B	397737091.8	13300044.17	-73.505	-44.219	4430	-0.031778692	-2.551538836	0.513666939
174070	3/13/2019 23:25	B	262377518.6	8233443.918	-73.417	-44.093	5620	-0.64725301	-2.083273887	0.747405639
174070	3/13/2019 23:52	A	42828.25095	18924.74905	-73.392	-44.066	1582	0.366053955	-2.371930401	0.653760762

174070	3/14/2019 2:08	0	10823093.35	1762097.145	-73.434	-43.922	8196	0.827296323	-0.549788392	0.122359726
174070	3/14/2019 2:45	B	41013557.97	10461480.53	-73.457	-43.892	2201	1.877710504	-0.378090989	0.248234005
174070	3/14/2019 3:22	B	46240048.11	14228808.89	-73.478	-43.863	2242	3.359947776	-0.279310766	0.163322591
174070	3/14/2019 3:50	B	5319669.432	484683.0684	-73.471	-43.873	1641	2.784612398	-0.308579798	0.234246895
174070	3/14/2019 4:34	B	3102328.881	345609.6189	-73.498	-43.837	2648	3.733046126	-0.190592428	-0.030437545
174070	3/14/2019 7:41	B	9787445.648	4452562.352	-73.584	-43.69	11231	-0.327445525	0.097653913	-0.173503915
174070	3/14/2019 9:20	0	3937138.74	16928234.26	-73.411	-43.668	5933	0.522297966	-0.001468571	-0.50441755
174070	3/14/2019 10:33	B	44548238.55	16170423.95	-73.409	-43.606	4387	-0.055363092	0.097524016	-0.447560105
174070	3/14/2019 11:11	B	1489275691	46356451.81	-73.405	-43.611	2281	-0.269771037	0.043302741	-0.454770421
174070	3/14/2019 12:38	B	70236364.3	29989340.7	-73.167	-43.597	5231	0.071627857	-0.064583649	-0.195058817
174070	3/14/2019 12:54	B	114666533.5	28677575.48	-73.163	-43.58	969	-0.237432037	-0.043638861	-0.240147951
174070	3/14/2019 13:17	B	51010213.67	1831406.331	-73.152	-43.588	1333	-0.133157847	-0.072422056	-0.223178292
174070	3/14/2019 13:37	B	55232711.39	3967613.613	-73.136	-43.566	1203	-0.117804808	-0.026598056	-0.303096398
174070	3/14/2019 14:16	B	25837698.99	215869.5096	-73.137	-43.551	2368	-0.059827809	0.017147491	-0.36346227
174070	3/14/2019 21:45	B	1331629.358	15977999.64	-73.123	-43.06	26957	-0.313692669	0.163203223	-0.610190177
174070	3/14/2019 23:23	B	3161407.387	10509005.11	-73.044	-42.944	5875	0.080776693	-0.112234512	-0.572047217

174070	3/15/2019 1:07	B	8781736.167	1501880.333	-73.02	-42.875	6220	0.457081514	-0.345419278	-0.526274538
174070	3/15/2019 1:35	B	888949299.7	2442076.809	-73.026	-42.887	1686	0.663817636	-0.310294408	-0.522283284
174070	3/15/2019 1:51	B	113090759.2	47702344.77	-73.024	-42.878	964	0.438206653	-0.323715341	-0.536644622
174070	3/15/2019 2:27	A	217127.4243	44043.07574	-73.012	-42.885	2145	0.612839673	-0.318554669	-0.470833313
174070	3/15/2019 3:06	B	741097.9631	197228.5369	-73.004	-42.872	2344	0.381482642	-0.377487787	-0.46234045
174070	3/15/2019 3:17	B	1066421.401	649735.5986	-73.005	-42.873	689	0.445895911	-0.368140897	-0.468637722
174070	3/15/2019 3:26	1	171580.2519	137322.2481	-73.028	-42.877	535	0.385640173	-0.341094677	-0.562980467
174070	3/15/2019 4:11	B	2034702.879	784247.6208	-73.006	-42.875	2679	0.44863333	-0.363857429	-0.467430199
174070	3/15/2019 7:25	B	66649061.18	13216958.82	-73.075	-42.78	11641	-0.655193381	-0.702266202	-0.431658007
174070	3/15/2019 9:03	A	502177198.1	9921848.445	-73.162	-42.704	5901	0.260064911	-1.297709629	-0.328031545
174070	3/15/2019 9:04	B	251319256.9	6361184.134	-73.159	-42.705	64	0.27298889	-1.310925195	-0.326648482
174070	3/15/2019 9:56	B	66311045.07	57668647.43	-73.19	-42.672	3092	0.485098288	-1.236083813	-0.094803196
174070	3/15/2019 10:46	B	17665016.83	9436793.667	-73.138	-42.591	2997	1.837897836	-1.39732771	0.365485211
174070	3/15/2019 10:48	B	6233360.567	3377345.933	-73.137	-42.589	144	1.800795607	-1.422131746	0.370429845
174070	3/15/2019 11:20	B	9288528.367	16327068.13	-73.123	-42.569	1918	0.53305377	-1.640078028	0.51937971
174070	3/15/2019 11:37	0	5114561.858	1527266.142	-73.129	-42.548	979	-0.43468248	-1.957940468	0.582389366

174070	3/15/2019 12:12	B	2617885.2	20851391.8	-73.142	-42.512	2136	0.149458552	-2.140343099	0.580811742
174070	3/15/2019 12:28	B	2703320.958	35651807.54	-73.142	-42.504	969	0.274224164	-2.093206129	0.595058159
174070	3/15/2019 12:56	B	730662.6056	458863.8944	-73.15	-42.522	1644	-0.057543228	-2.021707185	0.550692799
174070	3/15/2019 13:19	B	1295481.5	750003.5	-73.151	-42.5	1391	0.513596329	-1.971408668	0.598810893
174070	3/15/2019 13:56	B	137012619.6	3470764.934	-73.148	-42.423	2225	-0.378080011	-2.588509979	0.717724557
174070	3/15/2019 14:26	B	3030146.307	541760.1926	-73.152	-42.433	1825	-0.61234362	-2.512725375	0.728573183
174070	3/15/2019 14:39	B	10344624.52	1584889.98	-73.152	-42.441	749	-0.765938311	-2.42969234	0.743758849
174070	3/15/2019 15:36	B	1452928.771	2105256.229	-73.089	-42.384	3404	0.701001507	-2.319075748	0.959879266
174070	3/15/2019 19:43	B	5597858	1181184.5	-73.08	-42.125	14847	-0.450355457	-2.866475189	1.364801219
174070	3/15/2019 21:14	B	13168250.45	526765.5548	-73.077	-42.031	5454	-0.514552642	-2.82590223	1.241876167
174070	3/15/2019 22:57	A	220173581.7	5543280.835	-73.009	-41.959	6158	0.071867202	-2.700231219	1.364103246
174070	3/15/2019 23:06	B	59105109.5	1965091.496	-73.007	-41.96	538	0.071182597	-2.710621467	1.369073744
174070	3/16/2019 0:40	A	2000733.529	17346.47118	-73.066	-41.95	5673	1.083459855	-1.73653895	1.339168852
174070	3/16/2019 1:17	A	61319.16933	4065.830669	-73.076	-41.944	2245	1.349331153	-1.479867721	1.260390276
174070	3/16/2019 1:31	A	543850.9229	1070501.577	-73.105	-41.941	826	1.60661723	-1.281664133	1.06650089
174070	3/16/2019 2:08	B	3679957.512	7501266.488	-73.103	-41.942	2207	1.660176109	-1.288776409	1.090077687

174070	3/16/2019 2:19	B	4775334.583	7219379.917	-73.084	-41.923	676	1.202186143	-1.289863383	1.096836512
174070	3/16/2019 2:41	B	7677637.809	2714886.691	-73.087	-41.93	1328	1.387110425	-1.304771079	1.111730988
174070	3/16/2019 3:10	B	8698875.5	26090385.5	-73.095	-41.929	1700	1.273874128	-1.26292091	1.056501646
174070	3/16/2019 7:14	A	1576603.957	5545784.543	-73.082	-41.98	14679	0.395287496	-1.926088935	1.381151064
174070	3/16/2019 8:51	B	366181653.3	48510471.73	-73.093	-41.954	5803	1.306454113	-1.447255943	1.26242729
174070	3/16/2019 9:26	B	7452964.007	720746.4927	-73.086	-41.957	2090	1.126458964	-1.514403828	1.301323084
174070	3/16/2019 10:20	A	66512.84089	474367.1591	-73.115	-41.949	3236	1.858711839	-1.269854151	1.10430341
174070	3/16/2019 10:32	B	3099801.108	580863.3923	-73.076	-41.954	707	1.06347428	-1.597468031	1.307154317
174070	3/16/2019 11:04	B	2406486.945	261646.0553	-73.074	-41.946	1955	1.25976396	-1.564487178	1.292418265
174070	3/16/2019 11:12	B	2751032.967	548065.5333	-73.082	-41.951	449	1.379317631	-1.511716826	1.270355282
174070	3/16/2019 12:03	A	28354838.52	1776859.981	-73.109	-41.974	3057	0.8888551	-1.557150897	1.318179583
174070	3/16/2019 12:45	B	7781938.899	487541.1011	-73.106	-41.995	2527	-0.025685494	-1.990702476	1.395579802
174070	3/16/2019 13:28	0	18253852.12	1782032.885	-73.078	-41.964	2572	0.757246488	-1.735509005	1.349671886
174070	3/16/2019 14:08	B	131979616.9	1062676.055	-73.029	-41.902	2414	1.569904323	-1.473242514	1.139769133
174070	3/16/2019 14:35	3	73869.07333	49625.42667	-73.056	-41.877	1603	0.343700503	-1.172129952	1.081192263
174070	3/16/2019 15:11	0	20808583.74	29352128.26	-73.041	-41.864	2158	0.790350404	-1.259977655	1.123966505

174070	3/16/2019 20:43	B	353510961	330074631.5	-73.176	-41.868	19931	1.974921285	-1.722907857	0.977916155
174070	3/16/2019 21:11	A	156670229.2	1295691.842	-73.12	-41.915	1699	0.249614241	-1.331467162	0.893883941
174070	3/16/2019 22:27	B	59678319.62	6391185.375	-73.127	-41.898	4550	0.445574394	-1.351134843	0.913071948
174070	3/16/2019 22:48	0	562017082.3	319389430.2	-73.117	-41.957	1266	0.984060929	-1.28126379	0.946800822
174070	3/17/2019 0:04	B	604406748	369165686.5	-73.12	-41.953	4530	0.388681031	-1.247355171	1.145045275
174070	3/17/2019 1:07	2	392447.3659	49184.63411	-73.154	-41.933	3783	-0.299494013	-1.330960866	1.035238718
174070	3/17/2019 1:45	B	790929.4993	195153.0007	-73.148	-41.914	2325	-0.598187375	-1.426145787	1.011715583
174070	3/17/2019 1:56	0	42073730.75	2323665.254	-73.126	-41.94	626	0.043473686	-1.257696262	1.082611054
174070	3/17/2019 2:24	B	13751923.69	2628765.31	-73.125	-41.939	1685	0.13727342	-1.258713269	1.099934455
174070	3/17/2019 3:25	2	1087311.1	51058.89994	-73.158	-41.927	3688	-0.444535103	-1.39057677	1.019372521
174070	3/17/2019 3:59	B	16811494.72	5296725.783	-73.166	-41.92	2001	-0.599702358	-1.474765604	0.996537137
174070	3/17/2019 4:29	B	5171883.457	100955509.5	-73.154	-41.916	1844	-0.578890158	-1.458885661	1.005161419
174070	3/17/2019 5:07	B	5210502.308	9576029.692	-73.144	-41.895	2264	-0.360892648	-1.51091204	1.036590575
174070	3/17/2019 7:02	A	1495221.809	1711692.191	-73.203	-41.879	6870	-0.288995355	-2.314592036	0.908314038
174070	3/17/2019 10:21	1	92724.75057	197935.7494	-73.187	-41.895	11972	-0.351033698	-1.868275177	0.949600059
174070	3/17/2019 11:35	1	3296309.56	7162.940285	-73.198	-41.935	4455	-0.325540961	-1.476457424	1.004918072

174070	3/17/2019 12:13	1	558761.5235	110667.4765	-73.206	-41.914	2252	-0.532907008	-1.854527002	0.937534855
174070	3/17/2019 12:37	A	231437525.5	27475630.47	-73.197	-41.918	1458	-0.550614235	-1.618126598	0.96399966
174070	3/17/2019 13:08	A	309859978.9	103304911.6	-73.185	-41.905	1842	-0.624002016	-1.626428427	0.973223374
174070	3/17/2019 13:15	B	2833631.151	938921.8486	-73.176	-41.91	443	-0.624002016	-1.628190224	0.973223374
174070	3/17/2019 13:44	B	37958791.13	8310469.367	-73.184	-41.903	1694	-0.512146563	-1.71135076	0.959773273
174070	3/17/2019 14:11	A	1560396.436	4896.564078	-73.168	-41.938	1667	-0.228297735	-1.355532192	1.041881842
174070	3/17/2019 14:15	B	276688.6345	16279.36549	-73.168	-41.937	229	-0.265984672	-1.355532192	1.037928111
174070	3/17/2019 14:50	B	2207092.433	493532.5669	-73.187	-41.894	2098	-0.329223714	-1.920401145	0.946037992
174070	3/17/2019 20:13	B	7674766.342	1997956.158	-73.2	-41.835	19351	0.447074938	-2.043579591	1.009743756
174070	3/17/2019 21:00	A	18368267.3	4778295.201	-73.192	-41.92	2841	-0.529827238	-1.584482775	0.971799674
174070	3/17/2019 21:51	1	453470.3369	1868798.663	-73.199	-41.967	3059	-0.002439198	-1.519348404	1.11585108
174070	3/17/2019 22:38	0	1394881.991	2766902.509	-73.238	-41.888	2831	-0.55047896	-2.662151854	0.844941845
174070	3/17/2019 23:47	B	10319061.31	62878.68924	-73.249	-41.953	4148	0.217541872	-2.045800878	0.934114607
174070	3/18/2019 1:03	B	4699054.339	1012165.661	-73.273	-41.951	4557	0.919149688	-2.538015635	0.42370854
174070	3/18/2019 1:30	1	345423.9654	636318.5346	-73.253	-41.963	1623	0.884427842	-2.19888753	0.525560696
174070	3/18/2019 2:00	B	1533328.308	916084.1916	-73.223	-41.961	1783	0.633982953	-1.689255382	0.582738701

174070	3/18/2019 2:26	0	18689970.63	2547235.872	-73.219	-41.937	1566	0.504190429	-1.702915678	0.465961986
174070	3/18/2019 3:09	0	116419581.1	369231.3849	-73.243	-41.959	2561	0.796732548	-1.899424044	0.541115082
174070	3/18/2019 4:09	2	404074.9216	84375.0784	-73.263	-41.923	3582	0.445038349	-2.668519654	0.273797965
174070	3/18/2019 8:25	0	3626030039	1181193.474	-73.37	-41.961	15372	1.101608822	-2.866475189	0.450024212
174070	3/18/2019 10:05	2	657745.1939	351893.3061	-73.262	-41.977	6019	1.007044518	-2.452372465	0.614276839
174070	3/18/2019 11:11	B	1023929560	20240969.98	-73.275	-41.951	3970	0.918508511	-2.560668018	0.406609924
174070	3/18/2019 11:43	0	1254924.413	1708452.587	-73.293	-41.919	1900	0.398910868	-2.866475189	0.200183193
174070	3/18/2019 12:13	A	46158545.81	23353832.19	-73.324	-41.898	1822	0.194693388	-2.773795709	0.054875847
174070	3/18/2019 12:24	B	8530830.685	3273431.815	-73.324	-41.899	632	0.196613305	-2.773795709	0.059058211
174070	3/18/2019 12:52	A	252963.0847	26369.91529	-73.285	-41.907	1666	0.213604615	-2.866475189	0.158155037
174070	3/18/2019 13:21	B	8288892.388	3234642.112	-73.291	-41.912	1751	0.27347719	-2.866475189	0.164020267
174070	3/18/2019 13:49	2	120985.9407	94710.05927	-73.28	-41.918	1724	0.382740306	-2.826884082	0.219995734
174070	3/18/2019 14:06	A	79646.96757	461638.0324	-73.254	-41.917	1019	0.390472264	-2.593835765	0.27479599
174070	3/18/2019 14:32	B	28747044.71	142668.2918	-73.249	-41.917	1562	0.397580748	-2.477789717	0.295433414
174070	3/18/2019 15:34	B	216698.1082	719414.3918	-73.21	-41.911	3673	0.307388175	-1.937538157	0.377231855
174070	3/18/2019 20:48	B	78466372.12	17078074.38	-73.208	-41.893	18880	0.220299978	-2.282606192	0.292100489

174070	3/18/2019 21:25	A	602353905.9	11906728.58	-73.319	-41.965	2217	1.061324837	-2.730489007	0.475432062
174070	3/18/2019 22:27	A	2998688.673	7845832.327	-73.33	-41.933	3712	0.74977347	-2.866475189	0.216943655
174070	3/18/2019 22:56	B	14943810.09	8449057.915	-73.33	-41.933	1714	0.74977347	-2.866475189	0.216943655
174070	3/18/2019 23:20	B	25917300.92	6360707.584	-73.337	-41.936	1434	0.798907742	-2.866475189	0.223952386
174070	3/19/2019 0:09	B	21133542.57	128623726.4	-73.342	-41.941	2975	3.62164887	-2.866475189	-0.17623462
174070	3/19/2019 0:45	B	4225211.431	39953453.57	-73.352	-41.948	2146	4.06450098	-2.866475189	-0.054230885
174070	3/19/2019 1:07	B	4260851.982	10876262.52	-73.363	-41.956	1328	4.260770399	-2.866475189	0.098818148
174070	3/19/2019 2:09	1	3998997.032	611615.4681	-73.353	-41.996	3720	2.561589521	-2.866475189	0.609073638
174070	3/19/2019 2:29	A	4471320.244	1271545.756	-73.33	-41.999	1166	3.096997254	-2.866475189	0.618137694
174070	3/19/2019 2:43	1	77095854.68	4114637.819	-73.325	-42.007	855	2.843518141	-2.866475189	0.668574565
174070	3/19/2019 3:43	B	14734885.55	4099096.949	-73.336	-42.016	3620	1.700386258	-2.866475189	0.775285485
174070	3/19/2019 4:25	B	19283872.86	5567267.145	-73.335	-42.022	2519	1.144926743	-2.866475189	0.82673019
174070	3/19/2019 6:38	B	33380892.47	5189680.533	-73.351	-42.04	7978	0.354082549	-2.866475189	0.837807421
174070	3/19/2019 8:19	3	30225.43835	8307.061646	-73.386	-42.023	6080	0.415160535	-2.866475189	0.727591857
174070	3/19/2019 9:05	B	1006093.304	846335.6955	-73.416	-42.028	2749	0.642197903	-2.653923099	0.599305519
174070	3/19/2019 9:33	A	209473.2661	1489919.234	-73.404	-42.036	1639	0.553735398	-2.705370688	0.650241938

174070	3/19/2019 10:04	B	525257.5758	440659.4242	-73.411	-42.038	1899	0.616858053	-2.576018213	0.606317969
174070	3/19/2019 10:40	B	1268105.726	614864.7742	-73.408	-42.036	2163	0.585566685	-2.648857249	0.633973495
174070	3/19/2019 11:14	A	672131.4616	808846.5384	-73.404	-42.061	2008	0.691374549	-2.425405459	0.545679225
174070	3/19/2019 11:56	B	49457375.29	6475474.706	-73.35	-41.983	2519	3.287004789	-2.866475189	0.474546978
174070	3/19/2019 12:32	B	186652210.8	1998697.748	-73.359	-42.025	2178	0.646235192	-2.866475189	0.824998591
174070	3/19/2019 13:27	0	21506195404	812712328.7	-73.361	-42.025	3284	2.134626528	-2.866475189	0.825129679
174070	3/19/2019 13:53	B	305353446	60659116.02	-73.264	-42.021	1572	3.379011708	-2.843588289	0.698312629
174070	3/19/2019 14:10	B	15446222.88	1121319.623	-73.277	-42.007	1038	3.893936119	-2.778197147	0.508475622
174070	3/19/2019 20:40	B	16559271.01	2353341.99	-73.324	-41.999	23408	3.23390833	-2.866475189	0.603971268
174070	3/19/2019 20:50	B	43752225.85	692154.6526	-73.335	-42.027	599	0.939864559	-2.866475189	0.851053681
174070	3/19/2019 22:15	A	17365597.44	10689367.06	-73.367	-41.957	5092	4.138050673	-2.866475189	0.145447831
174070	3/19/2019 22:31	0	11328404.97	30553227.53	-73.309	-41.971	947	3.686278159	-2.866475189	0.076668369
174070	3/19/2019 22:55	B	82289332.68	11859935.32	-73.315	-41.961	1463	3.544377535	-2.866475189	-0.005284064
174070	3/19/2019 23:59	B	5634008.863	15152835.64	-73.388	-41.957	3802	3.557758962	-2.866475189	0.22050385
174070	3/20/2019 0:11	A	8448858.118	13455906.88	-73.364	-41.961	743	3.985872911	-2.866475189	0.03384868
174070	3/20/2019 0:44	B	291997778	38088	-73.36	-42.013	1964	1.805425558	-2.866475189	0.732532748

174070	3/20/2019 2:20	B	71025834.99	12846427.51	-73.354	-42.018	5771	1.373474044	-2.866475189	0.76054219
174070	3/20/2019 2:28	B	276545086.8	65787655.73	-73.364	-42.018	500	1.300029643	-2.866475189	0.759449326
174070	3/20/2019 3:00	B	11614076.17	1294151.833	-73.377	-42.018	1896	1.132421288	-2.866475189	0.738969173
174070	3/20/2019 3:27	B	158070735.8	3966778.698	-73.393	-42.027	1636	0.859167639	-2.866475189	0.712081243
174070	3/20/2019 4:09	B	23967455.67	3534944.333	-73.39	-42.024	2504	0.884187327	-2.866475189	0.71993088
174070	3/20/2019 8:04	1	600075.8285	334498.6715	-73.315	-42.013	14107	3.359389479	-2.866475189	0.666157961
174070	3/20/2019 10:24	B	31870525.2	11917253.3	-73.326	-42.008	8383	3.606752927	-2.866475189	0.627101235
174070	3/20/2019 11:29	0	6851066.55	15487834.45	-73.305	-42.039	3887	0.143377463	-2.866475189	0.845185002
174070	3/20/2019 12:02	1	3514930.305	14214.69536	-73.299	-42.052	2030	-0.677747312	-2.866475189	0.874321075
174070	3/20/2019 12:07	B	928920.4982	242679.5018	-73.302	-42.053	263	-0.677747312	-2.866475189	0.874321075
174070	3/20/2019 12:20	0	3934715.152	3237956.848	-73.302	-42.033	814	0.945774496	-2.866475189	0.809730948
174070	3/20/2019 13:09	A	118990031.5	216458.4801	-73.279	-42.03	2897	1.779947507	-2.866475189	0.743016221
174070	3/20/2019 13:44	B	1953074.548	60717.95172	-73.275	-42.036	2148	1.611316749	-2.866475189	0.743026697
174070	3/20/2019 14:22	A	28596.18282	11389.81718	-73.288	-42.022	2250	2.627044386	-2.866475189	0.692190256
174070	3/20/2019 15:30	A	622520.7725	1471919.227	-73.271	-42.015	4075	3.631962837	-2.802549711	0.501126955
174070	3/20/2019 20:23	B	11914177.63	4806688.875	-73.319	-42.046	17558	-0.598909818	-2.866475189	0.870778521

174070	3/20/2019 21:59	B	28979985.26	1739127.237	-73.314	-42.025	5765	1.599492421	-2.866475189	0.782758719
174070	3/20/2019 22:11	0	19858202371	897209279.5	-73.304	-42.018	726	1.969971216	-2.866475189	0.759078332
174070	3/20/2019 22:36	B	98618050.82	4795697.681	-73.305	-42.019	1525	2.400064476	-2.866475189	0.727354076
174070	3/20/2019 23:37	1	5307403.773	1394369.227	-73.281	-42.034	3627	1.779947507	-2.866475189	0.743016221
174070	3/21/2019 0:08	B	2728513.139	724368.8611	-73.276	-42.035	1911	2.688656774	-2.866475189	0.596450949
174070	3/21/2019 0:21	A	26900.39199	346712.108	-73.269	-42.041	728	2.379808686	-2.866475189	0.632701136
174070	3/21/2019 1:25	B	55335032.95	2501604.046	-73.257	-42.045	3849	2.749339925	-2.866475189	0.554183867
174070	3/21/2019 2:02	B	27929839.62	1560660.376	-73.227	-42.014	2212	2.657033087	-2.587862843	0.011187203
174070	3/21/2019 2:43	B	6107358.642	774539.858	-73.224	-42.014	2510	2.431030376	-2.587862843	0.000454289
174070	3/21/2019 3:09	2	1474349.965	33732.53487	-73.232	-42.035	1531	3.087809616	-2.866475189	0.256778605
174070	3/21/2019 3:43	B	9097812.039	341868.9613	-73.229	-42.03	2075	3.069878433	-2.843588289	0.223795496
174070	3/21/2019 4:45	B	10856313.53	1942766.966	-73.2	-42.009	3707	0.935597381	-2.230167617	-0.057881226
174070	3/21/2019 9:40	1	829784.0252	30016.97481	-73.171	-41.998	17693	0.027461023	-1.735703058	-0.037314547
174070	3/21/2019 10:00	B	13245804.5	4027122	-73.171	-41.998	1209	0.027461023	-1.728453471	-0.037314547
174070	3/21/2019 11:13	B	391104512	10224242	-73.13	-41.988	4379	0.378976385	-1.466897717	-0.015444106
174070	3/21/2019 11:37	B	1971207494	162078898.6	-73.145	-41.989	1416	0.454944656	-1.482305492	-0.002256571

174070	3/21/2019 11:44	B	13086735.14	518349.858	-73.133	-41.988	426	0.454944656	-1.52290804	-0.002256571
174070	3/21/2019 12:44	A	577290.6777	436578.3223	-73.086	-42.005	3627	2.401119165	-2.397217439	0.245828864
174070	3/21/2019 13:26	1	1576191.406	158501.0939	-73.096	-42.014	2465	1.851913923	-2.440401265	0.266378178
174070	3/21/2019 14:01	B	2041481.674	195998.8261	-73.067	-42.031	2126	0.979591057	-2.866475189	0.524459223
174070	3/21/2019 14:28	1	3244887.549	2650657.451	-73.037	-42.065	1647	0.296474819	-2.866475189	0.679909314
174070	3/21/2019 15:08	B	11573632.71	12715167.79	-73.045	-42.066	2356	0.397495393	-2.866475189	0.64176154
174070	3/21/2019 21:51	0	2418891.594	29980682.91	-73.399	-41.921	24171	2.60233137	-2.866475189	-0.232448799
174070	3/21/2019 22:09	B	4841867.625	6629502.875	-73.398	-41.923	1105	2.603100145	-2.866475189	-0.211673192
174070	3/21/2019 23:06	B	4041167494	479134580.2	-73.388	-42.007	3403	2.21571207	-2.866475189	0.506496548
174070	3/21/2019 23:48	A	31388141.49	625477.012	-73.343	-42.029	2538	0.347098963	-2.866475189	0.757071389
174070	3/22/2019 0:08	A	62023.15643	246720.8436	-73.352	-42.033	1222	-0.429770446	-2.866475189	0.0319732
174070	3/22/2019 1:06	A	166387.9664	201194.5336	-73.284	-42.07	3474	-0.66697909	-2.866475189	0.145725041
174070	3/22/2019 1:33	3	24345.35733	9053.142674	-73.299	-42.054	1636	-0.489847034	-2.866475189	0.101719314
174070	3/22/2019 1:41	B	206660.0102	33894.4898	-73.298	-42.057	445	-0.540986252	-2.866475189	0.111728423
174070	3/22/2019 1:48	B	301541.3113	77541.18872	-73.299	-42.056	422	-0.526734876	-2.866475189	0.108143551
174070	3/22/2019 2:43	A	110821857	2198945.526	-73.396	-42.035	3326	-0.476483701	-2.866475189	-0.005618002

174070	3/22/2019 3:32	B	966692.6248	23402485.88	-73.338	-42.052	2901	-0.687615352	-2.866475189	0.088070631
174070	3/22/2019 4:26	B	3214239.247	3052737.753	-73.334	-42.06	3257	-0.77267322	-2.866475189	0.106329037
174070	3/22/2019 5:07	B	5992405.862	63341476.64	-73.301	-42.058	2446	-0.580635593	-2.866475189	0.114452106
174070	3/22/2019 9:21	A	2121739.904	10822.59646	-73.268	-42.032	15237	-0.035555895	-2.866475189	0.015929522
174070	3/22/2019 9:30	A	2935810.153	1467314.847	-73.274	-42.056	527	-0.356967976	-2.866475189	0.102195299
174070	3/22/2019 11:19	0	14764847334	459078450.7	-73.215	-42.09	6569	-0.483214236	-2.866475189	0.184381858
174070	3/22/2019 11:29	B	96698427.09	4651845.413	-73.216	-42.092	604	-0.53919047	-2.866475189	0.191625955
174070	3/22/2019 12:27	2	376981.4565	54091.04353	-73.324	-42.138	3473	-0.620519365	-2.649679885	0.117450673
174070	3/22/2019 12:47	B	11110880.7	828431.8016	-73.327	-42.123	1203	-0.73933035	-2.611429118	0.131836553
174070	3/22/2019 13:08	2	83535.0913	40818.9087	-73.318	-42.138	1241	-0.653593353	-2.731981314	0.129872079
174070	3/22/2019 14:08	3	115128.8528	2863.647224	-73.328	-42.144	3648	-0.577900485	-2.599127533	0.107150764
174070	3/22/2019 20:03	B	18847989.35	2576065.154	-73.33	-42.127	21252	-0.695409044	-2.575499061	0.122818518
174070	3/22/2019 21:41	B	15600146.41	5050961.591	-73.331	-42.115	5889	-0.785400547	-2.650178155	0.131581004
174070	3/22/2019 22:34	B	9517295288	11606326.32	-73.283	-42.153	3212	-0.837031681	-2.866475189	0.226371451
174070	3/22/2019 23:22	1	1546990.069	80069.93133	-73.178	-42.213	2876	-0.557958005	-2.216298691	0.294125305
174070	3/23/2019 0:00	B	3540460.5	1019592	-73.159	-42.229	2292	0.256412696	-1.993128366	-0.21622197

174070	3/23/2019 0:17	B	864950.4871	1303266.513	-73.125	-42.224	964	-0.013283301	-2.518009598	-0.13837838
174070	3/23/2019 1:02	A	4088154.13	384345.8704	-73.103	-42.193	2729	-0.735465479	-2.866475189	-0.02157771
174070	3/23/2019 1:24	A	41868.09488	2284838.405	-73.124	-42.214	1341	-0.22651285	-2.549439025	-0.096207511
174070	3/23/2019 1:36	1	14048150.93	1167725.07	-73.108	-42.186	682	-0.785562234	-2.791240422	-0.015626117
174070	3/23/2019 2:24	B	6206887335	385852259.5	-73.107	-42.177	2911	-0.925748585	-2.809153461	-0.000776827
174070	3/23/2019 3:06	1	29296494.82	741367.6781	-73.131	-42.181	2489	-0.78818187	-2.665849143	-0.018017956
174070	3/23/2019 4:05	A	16235074.45	880498.049	-73.135	-42.163	3558	-0.999035722	-2.809153461	-0.000274552
174070	3/23/2019 4:42	B	2732582.993	293013.5073	-73.131	-42.152	2231	-1.141346492	-2.866475189	0.01486525
174070	3/23/2019 7:33	2	10923.72761	136410.7724	-73.192	-42.094	10275	-0.710413887	-2.866475189	-0.086895302
174070	3/23/2019 9:08	0	206213380.7	37569313.76	-73.178	-42.058	5651	-0.538298157	-2.580559746	-0.10528284
174070	3/23/2019 9:14	1	4588712.28	341335.7202	-73.154	-42.049	393	-0.527108381	-2.528326487	-0.104707968
174070	3/23/2019 11:24	B	876102556.9	462509367.6	-73.262	-42.082	7811	-0.291721003	-2.866475189	-0.297979253
174070	3/23/2019 12:21	A	9553444.834	210725.166	-73.36	-42.07	3422	-0.448393619	-2.866475189	-0.543337942
174070	3/23/2019 12:28	2	11441.85549	412650.6445	-73.372	-42.066	371	-0.506933454	-2.774503804	-0.574188373
174070	3/23/2019 12:44	B	442408.952	170859.548	-73.371	-42.067	957	-0.499089161	-2.774503804	-0.570001463
174070	3/23/2019 13:05	B	42447122.92	640803.5823	-73.367	-42.095	1278	-0.428531397	-2.502622501	-0.526697851

174070	3/23/2019 13:47	A	111035964.9	852917.5542	-73.35	-42.112	2550	-0.354438438	-2.538300379	-0.447840217
174070	3/23/2019 14:25	1	6484949.983	1694171.017	-73.298	-42.117	2269	-0.389626255	-2.866475189	-0.299703151
174070	3/23/2019 14:47	B	1950083.908	622938.5922	-73.29	-42.119	1311	-0.43058174	-2.866475189	-0.272407372
174070	3/23/2019 15:29	A	3347770.026	37081466.47	-73.228	-42.086	2525	-0.499655871	-2.866475189	-0.166832661
174070	3/23/2019 21:31	A	1111838268	16784680.32	-73.073	-42.11	21695	-1.219163729	-2.866475189	0.035014394
174070	3/23/2019 22:09	A	39287287.31	6431773.188	-73.096	-42.123	2273	-1.272277598	-2.866475189	0.037303682
174070	3/23/2019 23:12	A	67693.99903	249196.501	-73.1	-42.112	3782	-1.196683887	-2.866475189	0.030688456
174070	3/23/2019 23:43	A	20272872.17	3793024.834	-73.089	-42.144	1899	-1.278232785	-2.866475189	0.038495524
174070	3/24/2019 0:25	B	50292842.88	8841805.619	-73.08	-42.157	2526	-0.784154073	-2.866475189	0.044127839
174070	3/24/2019 0:37	B	48573900.74	12895945.76	-73.083	-42.151	681	-0.78257464	-2.866475189	0.05610101
174070	3/24/2019 1:02	A	27943.28302	509477.717	-73.071	-42.154	1515	-0.704657882	-2.866475189	0.043300556
174070	3/24/2019 1:26	2	87915.49323	73583.00677	-73.066	-42.142	1440	-0.618848547	-2.866475189	0.053385459
174070	3/24/2019 2:09	1	17246547.77	652701.2337	-73.04	-42.14	2571	-0.378515688	-2.866475189	0.014283404
174070	3/24/2019 2:17	A	2272790.333	265456.1669	-73.042	-42.147	497	-0.385948642	-2.866475189	-0.005713771
174070	3/24/2019 2:40	B	13574829.36	2809287.139	-73.036	-42.149	1369	-0.373079113	-2.866475189	-0.024242274
174070	3/24/2019 3:12	2	153313.406	62095.09403	-72.999	-42.144	1934	-0.57223389	-2.866475189	-0.099633262

174070	3/24/2019 3:14	B	359257.9151	118392.0849	-72.999	-42.144	85	-0.57223389	-2.866475189	-0.099633262
174070	3/24/2019 4:25	1	2596743.411	895167.0892	-73	-42.126	4274	-0.515059296	-2.866475189	-0.072602698
174070	3/24/2019 7:21	A	71581938.15	3999046.851	-73.088	-42.063	10590	-0.326300629	-2.846188709	0.021941933
174070	3/24/2019 9:01	A	983644436.9	19481405.13	-73.172	-42.029	5967	-0.187510249	-2.204451468	0.018030224
174070	3/24/2019 10:16	2	60252.76006	72651.23994	-73.161	-42.09	4508	-0.821478066	-2.866475189	0.043468157
174070	3/24/2019 10:18	B	408598.1644	34722.83564	-73.16	-42.087	103	-0.828503891	-2.866475189	0.045195079
174070	3/24/2019 10:38	B	2413404.5	739328	-73.161	-42.089	1194	-0.821478066	-2.866475189	0.043468157
174070	3/24/2019 11:15	B	13516868.53	29674239.97	-73.18	-42.089	2273	-0.723339767	-2.866475189	0.006190236
174070	3/24/2019 11:58	B	859921.7866	408146.2134	-73.197	-42.108	2575	-0.806610272	-2.866475189	-0.033383111
174070	3/24/2019 12:26	A	55682.42325	1038818.577	-73.231	-42.152	1635	-0.753279289	-2.866475189	-0.073021003
174070	3/24/2019 12:54	A	13536847.33	665817.6735	-73.239	-42.194	1683	0.476300306	-2.851380195	-0.217313639
174070	3/24/2019 13:31	0	300620499.3	13588657.18	-73.232	-42.181	2247	0.104232954	-2.85918795	-0.180389569
174070	3/24/2019 14:06	2	86214.47795	54014.52205	-73.282	-42.17	2075	0.056326655	-2.83847401	-0.20578955
174070	3/24/2019 14:34	B	1262469.585	215988.4152	-73.301	-42.16	1709	0.061753126	-2.837473968	-0.215755807
174070	3/24/2019 14:42	B	150241613.2	440661.3284	-73.276	-42.155	476	-0.141821191	-2.866475189	-0.182766407
174070	3/24/2019 15:08	A	445424.1023	505296.3977	-73.292	-42.153	1548	-0.195811284	-2.866475189	-0.173370388

174070	3/24/2019 15:43	B	1281044.053	263388.4473	-73.301	-42.158	2123	0.034299618	-2.866475189	-0.211834062
174070	3/24/2019 23:01	A	288754.4613	503212.0387	-73.108	-42.049	26238	-0.262372855	-2.670372557	-0.002779375
174070	3/24/2019 23:13	B	471617.75	142866.25	-73.111	-42.051	739	-0.330371878	-2.661593014	0.006795614
174070	3/25/2019 0:14	B	4795653.319	1528924.681	-73.049	-42.083	3690	-0.317516387	-2.866475189	0.052263995
174070	3/25/2019 1:14	A	54925758.34	1090282.16	-73.04	-42.106	3559	0.059667128	-2.866475189	0.039625691
174070	3/25/2019 2:18	B	19500607.03	534992.9737	-73.099	-42.1	3889	-0.523890542	-2.866475189	0.109454868
174070	3/25/2019 3:00	B	41927983.29	668275.2053	-73.12	-42.078	2516	-0.554297923	-2.866475189	0.061658914
174070	3/25/2019 3:26	2	296926.7177	191107.7823	-73.125	-42.074	1542	-0.525632775	-2.844686007	0.051607315
174070	3/25/2019 3:59	A	27378901.36	93198.63934	-73.109	-42.054	1970	-0.19678815	-2.681879493	-0.006261122
174070	3/25/2019 7:12	B	43546409.15	13708094.85	-73.124	-42.01	11565	-0.12874001	-1.967125149	-0.135535805
174070	3/25/2019 9:58	B	29776107.4	42999517.6	-73.139	-41.99	9999	-0.085391408	-1.510572221	-0.16384382
174070	3/25/2019 10:29	A	488479.4764	8507657.024	-73.17	-42.043	1830	0.036060113	-2.507135186	0.049342224
174070	3/25/2019 11:07	B	2072302.423	497273.5769	-73.18	-42.038	2280	0.06943095	-2.496868329	0.030489448
174070	3/25/2019 11:27	B	1055123.783	365489.2169	-73.173	-42.036	1184	0.179523224	-2.442701806	0.035310932
174070	3/25/2019 12:07	B	1201399.854	834610.6465	-73.217	-42.026	2428	0.336569886	-2.730898903	-0.112864232
174070	3/25/2019 13:06	1	922031.3923	803668.6077	-73.287	-42.11	3523	0.137734422	-2.866475189	-0.19787723

174070	3/25/2019 13:40	0	518473387	61506281.97	-73.284	-42.15	2078	-0.226716545	-2.866475189	-0.161602895
174070	3/25/2019 14:30	B	37816639.11	6934177.393	-73.297	-42.166	2969	0.29085322	-2.806324509	-0.242107816
174070	3/25/2019 14:44	B	70375330.21	2570870.794	-73.301	-42.183	843	0.512832903	-2.726469296	-0.308743393
174070	3/25/2019 15:22	1	212193.2416	4050009.258	-73.26	-42.19	2287	0.209998945	-2.866475189	-0.266270119
174070	3/25/2019 21:04	B	101168114.9	26379731.58	-73.262	-42.139	20538	-0.693104352	-2.866475189	-0.107221056
174070	3/25/2019 22:40	B	2054596.728	666224.2718	-73.211	-42.13	5746	-0.859649758	-2.866475189	-0.053646483
174070	3/25/2019 23:49	A	3066709.638	618300.8621	-73.294	-42.088	4160	0.247894559	-2.866475189	-0.302069888
174070	3/26/2019 0:23	B	3113189.13	1257775.87	-73.214	-42.129	2013	-0.675363374	-2.866475189	0.011676811
174070	3/26/2019 1:05	B	106006.3012	81973715.7	-73.236	-42.107	2528	-0.678986131	-2.866475189	-0.040066764
174070	3/26/2019 1:24	B	204160.5	2709792	-73.238	-42.094	1146	-0.704972025	-2.866475189	-0.06444662
174070	3/26/2019 1:59	B	3779914.552	8303022.448	-73.231	-42.043	2084	-0.278408312	-2.866475189	-0.107650143
174070	3/26/2019 2:36	B	11397928.31	13105730.19	-73.214	-42.02	2201	0.220210489	-2.594484056	-0.118490524
174070	3/26/2019 2:52	A	26304.18995	1452961.81	-73.218	-42.013	984	0.434177874	-2.506676036	-0.169395805
174070	3/26/2019 3:05	1	4293773.019	3067141.481	-73.225	-42.008	789	0.614624596	-2.450414085	-0.214415529
174070	3/26/2019 3:46	B	877812.5	440860.5	-73.21	-42.002	2431	0.862176817	-2.226873078	-0.216694674
174070	3/26/2019 4:41	B	65831525.69	15805966.31	-73.208	-42	3333	0.924465999	-2.170377186	-0.215909326

174070	3/26/2019 5:21	B	2558371.386	734401.1136	-73.206	-41.998	2377	0.911784646	-2.135470733	-0.209660626
174070	3/26/2019 6:58	B	4026129.182	768553.3184	-73.199	-41.991	5853	1.164697032	-1.844035708	-0.250267191
174070	3/26/2019 8:38	A	38507.58919	17998.91081	-73.302	-41.93	5957	0.3238329	-2.866475189	-0.801364918
174070	3/26/2019 9:31	B	923743.1816	257191.3184	-73.313	-41.915	3187	0.053966185	-2.866475189	-0.863933717
174070	3/26/2019 10:13	B	1571764.909	488467.5915	-73.321	-41.902	2555	0.039552236	-2.776991553	-0.876542636
174070	3/26/2019 11:05	0	4199816107	392686990.5	-73.272	-41.956	3119	0.529000784	-2.702245412	-0.758681818
174070	3/26/2019 12:32	3	1021407.209	39401.29104	-73.251	-41.927	5180	0.178277199	-2.437392216	-0.815459141
174070	3/26/2019 12:42	1	183794.095	297695.905	-73.281	-41.917	630	-0.098680963	-2.826884082	-0.873750656
174070	3/26/2019 12:48	B	1667911.938	582960.5619	-73.281	-41.917	364	-0.098680963	-2.828249292	-0.873750656
174070	3/26/2019 13:23	1	1219500.972	144052.0285	-73.334	-41.906	2106	0.182984445	-2.818156929	-0.845046713
174070	3/26/2019 14:00	B	2978959.777	749364.2226	-73.359	-41.902	2198	0.194985901	-2.777531121	-0.755221926
174070	3/26/2019 14:27	2	20839.55451	470182.9455	-73.389	-41.923	1596	-0.334424483	-2.866475189	-0.621391728
174070	3/26/2019 15:10	B	2495855.246	408130.7537	-73.385	-41.932	2592	-0.317363856	-2.866475189	-0.606621234
174070	3/26/2019 20:56	1	438057.8394	1007611.161	-73.257	-42.079	20778	-0.591230867	-2.866475189	-0.109826065
174070	3/26/2019 22:33	2	1059645.733	2244.266518	-73.197	-42.155	5828	-0.780820026	-2.866475189	0.072068073
174070	3/26/2019 23:26	A	50747.0049	11096.9951	-73.219	-42.14	3180	-0.718755384	-2.866475189	0.035400581

174070	3/27/2019 0:20	B	289513.5	569331.5	-73.201	-42.143	3250	-0.604357049	-2.866475189	0.569766281
174070	3/27/2019 1:05	2	40077.40588	60282.59412	-73.216	-42.135	2679	-0.580820903	-2.866475189	0.544285026
174070	3/27/2019 2:29	B	432093050.9	93961077.6	-73.232	-42.133	5016	-0.594518152	-2.866475189	0.52819197
174070	3/27/2019 2:39	B	119291066.5	17579367.99	-73.227	-42.133	629	-0.594518152	-2.866475189	0.52819197
174070	3/27/2019 3:22	3	275941.1517	2463.848266	-73.217	-42.144	2563	-0.595398968	-2.866475189	0.557836097
174070	3/27/2019 4:23	B	3699928.5	969127.9997	-73.222	-42.146	3673	-0.59577145	-2.866475189	0.562738692
174070	3/27/2019 5:02	B	2392887.607	6764318.893	-73.141	-42.145	2317	-0.880506744	-2.866475189	0.568684113
174070	3/27/2019 8:28	3	233634.6071	9775.39286	-73.192	-42.13	12399	-0.612352105	-2.866475189	0.547939905
174070	3/27/2019 9:06	B	2103498.057	585053.9429	-73.18	-42.13	2243	-0.6825993	-2.866475189	0.553698648
174070	3/27/2019 10:05	2	76445.741	71816.759	-73.185	-42.12	3525	-0.717669803	-2.866475189	0.525401508
174070	3/27/2019 10:22	B	64324266.61	121850983.4	-73.179	-42.121	1042	-0.748274307	-2.866475189	0.532760807
174070	3/27/2019 12:02	1	856326.4876	667628.0124	-73.178	-42.14	6015	-0.707689972	-2.866475189	0.576223899
174070	3/27/2019 12:24	3	387357.4609	6433.039091	-73.198	-42.132	1308	-0.572765512	-2.866475189	0.542971439
174070	3/27/2019 13:05	B	120198428.8	3018373.151	-73.193	-42.154	2491	-0.741140553	-2.866475189	0.596553924
174070	3/27/2019 13:39	B	5384975.661	1448292.839	-73.197	-42.135	2032	-0.592173633	-2.866475189	0.554912073
174070	3/27/2019 14:08	B	267328258.5	75578381.48	-73.244	-42.166	1733	-0.398170185	-2.858927692	0.584558959

174070	3/27/2019 14:43	B	10141258.68	1972847.317	-73.183	-42.202	2090	1.203870172	-2.36135852	0.468612434
174070	3/27/2019 20:44	A	16357862.73	80152599.77	-73.087	-42.153	21688	-0.720084043	-2.866475189	0.482633266
174070	3/27/2019 21:41	B	154892072.6	16423945.37	-73.083	-42.147	3410	-0.721163443	-2.866475189	0.484832233
174070	3/27/2019 22:58	B	2411965.495	1721204.505	-73.095	-42.16	4610	-0.660620929	-2.866475189	0.489089517
174070	3/27/2019 23:24	1	20416050	6384.5	-73.091	-42.182	1534	-0.344443559	-2.866475189	0.444801302
174070	3/28/2019 0:05	B	1898576.891	219791.6093	-73.085	-42.185	2493	-0.554336045	-2.866475189	0.444052858
174070	3/28/2019 0:39	A	319967.8155	4061.184494	-73.107	-42.182	2011	-0.046141615	-2.811130073	0.498294532
174070	3/28/2019 0:40	B	33315211.27	527414.7254	-73.104	-42.181	61	-0.047093881	-2.836752812	0.501811067
174070	3/28/2019 1:24	B	3682249.447	267261.0529	-73.099	-42.182	2645	-0.285963122	-2.866475189	0.474924221
174070	3/28/2019 2:22	1	19925043.44	634671.0645	-73.101	-42.19	3472	-0.274993335	-2.866475189	0.452778881
174070	3/28/2019 3:02	A	1921526502	102435966.6	-73.1	-42.211	2413	0.007518806	-2.866475189	0.389197471
174070	3/28/2019 3:33	B	8392719.025	47733.47533	-73.096	-42.221	1844	0.217341156	-2.866475189	0.346083557
174070	3/28/2019 4:05	B	2212966.918	128046.0821	-73.091	-42.226	1922	0.306454158	-2.866475189	0.306180216
174070	3/28/2019 8:14	B	213066.9503	46308930.05	-73.064	-42.222	14946	-0.015919343	-2.866475189	0.338079181
174070	3/28/2019 9:59	B	3921029.328	3810734.672	-73.067	-42.227	6336	0.200401541	-2.866475189	0.307868604
174070	3/28/2019 10:17	2	320944.7986	51063.20141	-73.069	-42.199	1083	-0.676824771	-2.866475189	0.409608678

174070	3/28/2019 10:30	B	3631072.64	1063103.36	-73.069	-42.199	739	-0.676824771	-2.866475189	0.409608678
174070	3/28/2019 11:31	B	380606152.5	4585807.959	-73.047	-42.179	3685	-0.809780897	-2.866475189	0.44435952
174070	3/28/2019 12:07	B	350752297.7	2085546.336	-73.059	-42.183	2173	-0.672089254	-2.866475189	0.440851187
174070	3/28/2019 13:09	B	2542995.651	375225.3487	-73.191	-42.197	3695	1.647248234	-2.518131081	0.581136485
174070	3/28/2019 13:39	B	634815864.6	4859335.44	-73.186	-42.211	1777	1.775370451	-2.319131227	0.466485787
174070	3/28/2019 14:26	B	28627681.37	14596137.13	-73.162	-42.189	2843	1.128841691	-2.412783404	0.652771706
174070	3/28/2019 20:34	B	305477745.6	22725895.39	-73.124	-42.185	22087	0.639670846	-2.589749609	0.587600729
174070	3/28/2019 21:11	B	330726389.9	174581172.6	-73.186	-42.189	2200	1.332947009	-2.467467723	0.652673932
174070	3/28/2019 22:33	B	5517832.075	1220187.925	-73.211	-42.187	4918	1.315979463	-2.771050263	0.609500859
174070	3/28/2019 22:48	B	6245311.892	283733.1082	-73.205	-42.18	931	1.02924734	-2.778337501	0.669264139
174070	3/28/2019 23:59	B	5424011.064	378321.436	-73.224	-42.17	4239	0.471564422	-2.855153944	0.654705347
174070	3/29/2019 0:15	A	28948934.59	1800307.908	-73.236	-42.157	957	-0.44342633	-2.866475189	0.386634069
174070	3/29/2019 0:29	B	8777407.671	494016.8285	-73.222	-42.169	849	-0.388622942	-2.855544331	0.40727231
174070	3/29/2019 2:02	B	3850907.891	1452157.109	-73.338	-42.167	5594	0.28371248	-2.260442699	0.075812192
174070	3/29/2019 2:12	B	13118353.93	4498975.067	-73.345	-42.167	605	0.156790379	-2.223029835	0.05473216
174070	3/29/2019 3:14	B	2426701.031	9089059.969	-73.238	-42.143	3680	-0.358729639	-2.866475189	0.358647509

174070	3/29/2019 3:38	B	5210331.859	3353990.641	-73.238	-42.149	1486	-0.443389908	-2.866475189	0.369254548
174070	3/29/2019 4:22	A	13648530.67	25819567.33	-73.207	-42.174	2587	-0.295695458	-2.815171835	0.449449383
174070	3/29/2019 8:03	3	106809.3834	9070.61663	-73.251	-42.183	13315	-0.818718811	-2.866475189	0.333911377
174070	3/29/2019 9:38	B	2934203.729	675165.2712	-73.217	-42.17	5683	-0.30569225	-2.847606447	0.422429583
174070	3/29/2019 10:00	B	3929962.827	807571.6727	-73.217	-42.167	1312	-0.301003465	-2.851900712	0.425295049
174070	3/29/2019 11:02	1	8111720.258	209934.2419	-73.182	-42.186	3700	-0.224954332	-2.556957037	0.455994453
174070	3/29/2019 11:45	B	7054064.781	1703857.219	-73.168	-42.191	2569	-0.329436152	-2.425881485	0.462701938
174070	3/29/2019 12:00	B	24652144.83	2994372.171	-73.166	-42.184	913	-0.500252582	-2.464430219	0.478085717
174070	3/29/2019 12:27	B	2708981.281	343035.2189	-73.164	-42.185	1638	-0.511546044	-2.453901853	0.477410342
174070	3/29/2019 13:26	A	1961422.37	228806.6299	-73.259	-42.173	3511	-0.898514406	-2.866475189	0.33464706
174070	3/29/2019 13:41	B	2952345.359	556867.1411	-73.265	-42.171	918	-0.898447384	-2.866475189	0.320656265
174070	3/29/2019 15:05	A	1465672.168	13354457.83	-73.312	-42.125	5031	0.226859076	-2.866475189	0.153651264
174070	3/29/2019 15:45	B	4089215.165	3641587.335	-73.299	-42.101	2401	-0.396281958	-2.866475189	0.179665641
174070	3/29/2019 20:20	B	414467696.5	49413575.96	-73.265	-42.15	16495	-0.710952206	-2.866475189	0.327725748
174070	3/29/2019 23:46	B	80262881.97	141330636.5	-73.197	-42.143	12372	-0.330324196	-2.86270144	0.467401295
174070	3/29/2019 23:59	A	1393134.692	1463283.308	-73.222	-42.171	774	-0.357733712	-2.855544331	0.40989908

174070	3/30/2019 0:18	B	5961751.367	3015114.633	-73.236	-42.163	1147	-0.724262296	-2.86283157	0.440409682
174070	3/30/2019 1:23	B	12718230.26	4330652.241	-73.22	-42.166	3914	-0.643979284	-2.858347115	0.465742068
174070	3/30/2019 2:01	B	19470662.31	5105196.194	-73.216	-42.163	2299	-0.630727613	-2.85918795	0.473267846
174070	3/30/2019 2:13	1	2445787.313	245875.1871	-73.225	-42.19	708	-0.682834345	-2.835167054	0.412355372
174070	3/30/2019 3:23	A	1399739.39	4287117.11	-73.224	-42.211	4161	-0.54085147	-2.767531733	0.367695349
174070	3/30/2019 3:38	A	264152.1888	61220167.81	-73.196	-42.182	914	-0.522051503	-2.620564163	0.441092507
174070	3/30/2019 4:00	A	155934.5644	1008370.436	-73.201	-42.199	1338	-0.506200815	-2.63851903	0.416224231
174070	3/30/2019 9:30	2	342691.9842	7101.015811	-73.204	-42.209	19778	-0.459241276	-2.605534921	0.380479293
174070	3/30/2019 9:32	A	874103.9453	133946.0547	-73.204	-42.207	114	-0.475434379	-2.601891302	0.393467669
174070	3/30/2019 10:32	1	9645850.784	79302.21592	-73.228	-42.214	3641	-0.535335129	-2.764989049	0.363350403
174070	3/30/2019 11:10	A	7246915.755	338016.7454	-73.221	-42.205	2231	-0.570059862	-2.776434552	0.386880561
174070	3/30/2019 11:29	B	617893.6675	286924.8325	-73.218	-42.208	1145	-0.527620151	-2.74723893	0.37900234
174070	3/30/2019 11:46	B	607756.6365	648413.3635	-73.236	-42.196	1060	-0.705976933	-2.844613474	0.392412884
174070	3/30/2019 12:07	B	36347877.54	178831.4616	-73.238	-42.192	1220	-0.741646196	-2.850821121	0.402022847
174070	3/30/2019 12:48	B	2223039.105	647293.8954	-73.247	-42.186	2493	-0.804526438	-2.858927692	0.397401953
174070	3/30/2019 13:26	A	1736329.858	1853188.642	-73.255	-42.179	2261	-0.843273113	-2.866475189	0.394769694

174070	3/30/2019 13:43	B	1945380.801	618719.6991	-73.259	-42.173	1000	-0.846651418	-2.866475189	0.396992225
174070	3/30/2019 14:42	B	7327459.348	49661753.65	-73.252	-42.155	3548	-0.762287285	-2.866475189	0.415880011
174070	3/30/2019 15:24	B	695587.9101	1116489.09	-73.256	-42.146	2560	-0.744334685	-2.866475189	0.409650993
174070	3/30/2019 20:11	B	7529049.984	866643.0161	-73.25	-42.181	17175	-0.83065604	-2.866475189	0.4011503
174070	3/30/2019 21:45	B	366461294.5	9199027.451	-73.227	-42.211	5659	-0.54085147	-2.77144525	0.367695349
174070	3/30/2019 23:23	B	2597238969	1181687744	-73.199	-42.21	5868	-0.48422155	-2.507854679	0.412456933
174070	3/30/2019 23:28	2	272220.6104	151771.8896	-73.197	-42.202	344	-0.483161944	-2.499177406	0.414852772
174070	3/31/2019 0:05	B	3413486.484	946949.5159	-73.196	-42.198	2173	-0.455529994	-2.552005673	0.254191268
174070	3/31/2019 1:04	A	54240.79599	28704.20401	-73.201	-42.179	3591	-0.606866172	-2.746528863	0.306114729
174070	3/31/2019 1:18	B	1369578.696	155893.8042	-73.211	-42.164	817	-0.729139496	-2.855544331	0.336491083
174070	3/31/2019 1:42	1	47232877.93	2519002.566	-73.227	-42.158	1418	-0.758755899	-2.86283157	0.333069717
174070	3/31/2019 2:00	B	239672614.2	660134.2793	-73.201	-42.159	1069	-0.752822856	-2.85918795	0.340470675
174070	3/31/2019 2:59	2	78787.21335	22067.28665	-73.222	-42.144	3549	-0.861867963	-2.866475189	0.36385405
174070	3/31/2019 3:25	A	187680.451	90600.54902	-73.263	-42.153	1563	-0.788697642	-2.866475189	0.315710847
174070	3/31/2019 3:41	A	37219.35105	3460.648949	-73.255	-42.149	990	-0.809884368	-2.866475189	0.324948118
174070	3/31/2019 7:41	3	34775.05971	10029.44029	-73.263	-42.102	14381	-1.103020205	-2.866475189	0.385025748

174070	3/31/2019 9:25	1	8310946.242	460606.2578	-73.234	-42.159	6239	-0.761731519	-2.866475189	0.325617816
174070	3/31/2019 11:35	B	44615043.07	11603405.43	-73.257	-42.137	7791	-0.905637761	-2.866475189	0.348522755
174070	3/31/2019 11:44	B	7143557.704	1745744.796	-73.257	-42.137	548	-0.901305519	-2.866475189	0.34608501
174070	3/31/2019 12:21	B	3311584.256	22441521.74	-73.272	-42.14	2209	-0.849891289	-2.866475189	0.333332747
174070	3/31/2019 12:43	B	397243.5487	807748.9513	-73.268	-42.131	1309	-0.928623794	-2.866475189	0.348798563
174070	3/31/2019 13:13	A	19541.2664	309883.7336	-73.274	-42.137	1857	-0.880554763	-2.866475189	0.337468628
174070	3/31/2019 13:23	0	63390633.48	1161192.52	-73.286	-42.123	586	-1.000618458	-2.866475189	0.356903472
174070	3/31/2019 13:55	B	107106528.7	5710552.344	-73.272	-42.12	1924	-1.004561073	-2.866475189	0.357444681
174070	3/31/2019 14:24	2	579767.3075	360417.6925	-73.315	-42.112	1750	-0.869143953	-2.776972968	0.335313202
174070	3/31/2019 15:02	B	488458.7351	5743078.265	-73.33	-42.11	2233	-0.802452691	-2.677215284	0.318291916
174070	3/31/2019 21:20	B	12707614.25	7989630.75	-73.39	-42.091	22669	-0.858177924	-2.247464029	0.207438864
174070	3/31/2019 21:41	B	74096028	20956669	-73.33	-42.091	1292	-0.802009796	-2.776972968	0.302790018
174070	3/31/2019 22:59	B	2190179.541	409982.959	-73.34	-42.089	4658	-0.790119018	-2.776972968	0.300743728
174070	3/31/2019 23:19	1	158616.6087	195979.8913	-73.304	-42.088	1235	-1.011960876	-2.866475189	0.356826099
174070	4/1/2019 1:01	A	36227906.24	19325831.76	-73.299	-42.092	6080	-1.029798471	-2.866475189	0.121051698
174070	4/1/2019 1:30	A	154149315.3	13496128.7	-73.303	-42.117	1793	-0.814739268	-2.866475189	0.089677721

174070	4/1/2019 1:33	B	414008454.2	37017888.33	-73.301	-42.118	126	-0.807093947	-2.866475189	0.088548117
174070	4/1/2019 2:21	B	37890403.07	172657779.4	-73.313	-42.118	2883	-0.943656461	-2.866475189	0.101175461
174070	4/1/2019 2:39	A	203950085.9	4050187.076	-73.301	-42.063	1099	-0.92813776	-2.866475189	0.123130076
174070	4/1/2019 3:11	1	22956547.89	1242710.607	-73.334	-42.067	1933	-0.819952084	-2.866475189	0.089297397
174070	4/1/2019 4:21	A	1023920.783	4634765.717	-73.297	-42.079	4161	-1.016149698	-2.866475189	0.131385506
174070	4/1/2019 4:59	B	1688236.271	1045917.729	-73.3	-42.081	2309	-1.020250316	-2.866475189	0.129564197
174070	4/1/2019 7:32	1	3370654.944	827383.5555	-73.322	-42.11	9151	-0.748495075	-2.776972968	0.076054903
174070	4/1/2019 9:08	1	168034791.3	5226985.655	-73.259	-42.127	5766	-0.701810515	-2.866475189	0.093099737
174070	4/1/2019 9:28	0	978789.676	5948642.824	-73.307	-42.142	1194	-0.259917164	-2.866475189	0.023568538
174070	4/1/2019 10:21	B	130005938.4	63758050.07	-73.328	-42.122	3194	-0.510443573	-2.62022381	0.048330692
174070	4/1/2019 11:07	B	23160139.54	354286.959	-73.324	-42.113	2797	-0.67926999	-2.657636674	0.067003099
174070	4/1/2019 11:25	0	12428756.41	1290016.089	-73.345	-42.106	1074	-0.609370388	-2.578079144	0.046814172
174070	4/1/2019 12:03	B	959716165.4	322164.5718	-73.344	-42.08	2251	-0.839502488	-2.866475189	0.086008049
174070	4/1/2019 12:21	0	1809967.494	7297621.006	-73.327	-42.071	1073	-0.879772902	-2.866475189	0.098023581
174070	4/1/2019 12:50	B	45439044.5	3892050	-73.343	-42.068	1779	-0.803868308	-2.866475189	0.084364734
174070	4/1/2019 13:02	A	223327.2887	416189.7113	-73.328	-42.065	718	-0.843785334	-2.866475189	0.098507968

174070	4/1/2019 13:36	1	66199916.73	2065211.268	-73.334	-42.106	2019	-0.710745978	-2.687470747	0.067218351
174070	4/1/2019 14:04	A	30314408.39	3211400.614	-73.335	-42.115	1659	-0.582286059	-2.610123149	0.050594926
174070	4/1/2019 14:39	A	236151.6703	358608.3297	-73.346	-42.11	2132	-0.504168606	-2.526579851	0.033807413
174070	4/1/2019 14:42	B	141663.6382	115758.8618	-73.346	-42.111	197	-0.493131165	-2.538300379	0.030949162
174070	4/1/2019 15:45	B	1876711.156	1197977.844	-73.343	-42.13	3762	-0.281268918	-2.381070133	0.002310272
174070	4/1/2019 20:47	B	227434668.6	16336095.92	-73.38	-42.056	18103	-0.624505127	-2.866475189	0.030391895
174070	4/1/2019 21:23	1	6706214.288	687555.7121	-73.284	-42.125	2152	-0.761188686	-2.866475189	0.093078361
174070	4/1/2019 22:24	0	288397692.4	34271349.58	-73.309	-42.139	3664	-0.307190405	-2.866475189	0.032346418
174070	4/1/2019 22:30	B	27535817.08	3108385.422	-73.306	-42.142	382	-0.259917164	-2.866475189	0.023568538
174070	4/2/2019 0:08	B	34222976.31	7795235.692	-73.274	-42.189	5887	2.128276741	-2.808472748	-0.115391555
174070	4/2/2019 1:16	B	50869159.23	5213791.265	-73.261	-42.191	4090	1.747610407	-2.866475189	-0.094871366
174070	4/2/2019 1:56	B	16382152.71	20191520.29	-73.282	-42.166	2351	1.424193975	-2.866475189	0.112902341
174070	4/2/2019 2:14	2	3646995.681	262232.8186	-73.279	-42.158	1099	0.914376238	-2.866475189	0.174505037
174070	4/2/2019 3:00	B	6909448.652	6009013.848	-73.246	-42.162	2782	0.979636564	-2.866475189	0.151157729
174070	4/2/2019 3:58	2	314683.4946	55461.50537	-73.312	-42.122	3444	-0.646339283	-2.834431184	0.252294871
174070	4/2/2019 4:39	2	101397.4891	107471.0109	-73.328	-42.125	2510	-0.226576121	-2.62022381	0.221883513

174070	4/2/2019 7:16	B	9213838.131	2469819.869	-73.373	-42.136	9413	0.814812975	-1.762830688	-0.013973779
174070	4/2/2019 9:00	B	23759762.8	2323231.204	-73.386	-42.141	6218	0.710472289	-1.629181075	-0.099879066
174070	4/2/2019 11:10	A	118570.8846	26234.11538	-73.324	-42.111	7770	-0.552431929	-2.770343174	0.246480352
174070	4/2/2019 11:32	1	3383731.747	13985.25287	-73.309	-42.137	1376	-0.070390988	-2.866475189	0.22665838
174070	4/2/2019 12:01	B	1687403.469	3159781.031	-73.311	-42.137	1699	-0.027985035	-2.866475189	0.221398922
174070	4/2/2019 12:16	B	54712.56457	17956695.94	-73.301	-42.114	891	-0.91227573	-2.866475189	0.269061339
174070	4/2/2019 12:42	1	11800757.76	379771.2373	-73.28	-42.156	1585	0.783698461	-2.866475189	0.184485018
174070	4/2/2019 12:47	B	3994506.711	132007.7885	-73.286	-42.16	284	1.235497235	-2.866475189	0.144673749
174070	4/2/2019 13:40	1	4091953.991	176642.0095	-73.3	-42.184	3188	2.532608715	-2.663466645	-0.097665757
174070	4/2/2019 14:30	B	14768761.92	3880600.577	-73.253	-42.186	3010	1.420685005	-2.866475189	-0.036704128
174070	4/2/2019 14:50	B	51467112.42	12618578.08	-73.251	-42.187	1172	1.329055796	-2.866475189	-0.046622348
174070	4/2/2019 21:15	A	122661202.9	3083039.626	-73.178	-42.21	23110	0.264752184	-2.267057378	-0.023466466
174070	4/2/2019 21:54	A	37140.70024	20509.79976	-73.191	-42.203	2359	0.075848382	-2.455771347	-0.032395015
174070	4/2/2019 23:30	1	280026.6518	154069.8482	-73.311	-42.16	5758	1.5386566	-2.810472832	0.0919362
174070	4/2/2019 23:43	B	6446282.159	1989376.341	-73.323	-42.155	807	1.270595423	-2.70573844	0.102343472
174070	4/3/2019 1:10	1	562687.8748	285041.1252	-73.209	-42.105	5183	-0.402394189	-2.866475189	0.384268404

174070	4/3/2019 1:32	B	1967937.125	5026834.875	-73.252	-42.115	1310	-0.641229092	-2.866475189	0.402594325
174070	4/3/2019 2:38	1	7272799.467	684890.5327	-73.361	-42.121	3959	1.243703623	-2.255834857	0.147537614
174070	4/3/2019 2:47	1	4920851.765	91698.73477	-73.347	-42.115	578	1.023237613	-2.426031661	0.236098703
174070	4/3/2019 3:14	B	15198725.05	3402207.447	-73.345	-42.116	1623	0.943115464	-2.471053442	0.246722969
174070	4/3/2019 4:17	1	1487887.732	408682.2682	-73.349	-42.129	3770	1.242450073	-2.27103551	0.192186406
174070	4/3/2019 7:07	0	7407040.522	3561751.978	-73.4	-42.043	10180	0.666337844	-2.701024656	-0.158926255
174070	4/3/2019 8:39	A	102702827.2	2623766.836	-73.458	-42.01	5527	0.579076438	-2.434495957	-0.216806936
174070	4/3/2019 9:31	1	734713.3729	566632.6271	-73.415	-41.997	3093	0.041937104	-2.83506355	-0.03711078
174070	4/3/2019 10:04	2	75930.06547	161252.4345	-73.415	-41.989	2000	0.138823205	-2.866475189	-0.024566353
174070	4/3/2019 10:23	2	42324.9439	50532.0561	-73.411	-41.987	1140	0.060004798	-2.866475189	-0.010207524
174070	4/3/2019 11:06	A	459538462.5	37788309.51	-73.427	-41.991	2600	0.352250625	-2.833941706	-0.046956424
174070	4/3/2019 11:49	B	72630175.97	3071424.532	-73.428	-42.017	2545	0.293701417	-2.596567773	-0.128547344
174070	4/3/2019 12:20	B	72779484.95	96277589.55	-73.422	-42.022	1862	0.331966489	-2.606207324	-0.127895915
174070	4/3/2019 12:40	1	8118324.831	25900.16869	-73.419	-41.994	1217	0.18175096	-2.838008391	-0.03919392
174070	4/3/2019 12:50	A	430383.4491	515866.5509	-73.39	-41.991	592	-0.022282535	-2.866475189	-0.010360793
174070	4/3/2019 13:59	B	6024939.934	402080.5663	-73.427	-41.992	4149	0.492489063	-2.83506355	-0.061809499

174070	4/3/2019 14:18	B	4654869.837	1041635.163	-73.43	-42.001	1127	0.479748338	-2.740828633	-0.094656283
174070	4/3/2019 14:29	B	3783528.336	864914.164	-73.428	-42	662	0.383896904	-2.772240272	-0.078325006
174070	4/3/2019 15:39	A	7912623.916	12926402.58	-73.354	-41.995	4211	0.465834234	-2.866475189	0.045715044
174070	4/3/2019 21:22	A	892401.126	3673896.874	-73.386	-42	20564	0.086828264	-2.866475189	-0.017503783
174070	4/3/2019 22:44	B	1209676.314	509650.1859	-73.371	-42.008	4948	0.418934375	-2.866475189	0.006368779
174070	4/3/2019 23:01	B	145169866.9	44605545.05	-73.339	-42.04	982	1.170009476	-2.866475189	0.153454742
174070	4/3/2019 23:22	B	2164652.791	362549.7088	-73.338	-42.039	1316	1.051162734	-2.866475189	0.219621989
174070	4/4/2019 0:24	B	4935958.339	1798364.161	-73.351	-42.058	3681	-0.131895316	-2.866475189	-0.512001639
174070	4/4/2019 1:01	B	190461.9337	12201415.07	-73.355	-42.061	2206	-0.170171457	-2.866475189	-0.54025798
174070	4/4/2019 1:08	1	549289.2915	86615.70849	-73.363	-42.062	461	-0.221921326	-2.805160932	-0.566701819
174070	4/4/2019 2:13	A	5531493.905	1592966.595	-73.364	-42.053	3909	-0.199998278	-2.834723163	-0.562891113
174070	4/4/2019 4:00	B	1918918.813	524041.6866	-73.363	-42.057	6411	-0.19194554	-2.834723163	-0.55667841
174070	4/4/2019 8:27	B	6600659.094	1724611.406	-73.366	-42.062	15999	-0.253026822	-2.805160932	-0.578926476
174070	4/4/2019 9:06	B	5256787.519	2207640.981	-73.33	-42.038	2332	-0.485008195	-2.866475189	-0.429978028
174070	4/4/2019 9:32	A	4954404.5	14789229.5	-73.313	-42.038	1566	-0.587482484	-2.866475189	-0.41953292
174070	4/4/2019 10:11	2	71199.73097	48106.26903	-73.312	-41.993	2375	-0.074519072	-2.866475189	-0.318540561

174070	4/4/2019 11:15	A	211009287.3	6561160.677	-73.275	-41.979	3796	0.284615644	-2.760675551	-0.327108409
174070	4/4/2019 12:02	A	7580339.939	42392.56075	-73.289	-41.979	2811	0.025636062	-2.782745061	-0.309941951
174070	4/4/2019 12:29	B	576586376	1584148.509	-73.27	-41.939	1651	-0.247266104	-2.60781329	-0.326735296
174070	4/4/2019 12:49	B	8215624.066	71193.93416	-73.27	-41.939	1211	-0.32326163	-2.589337441	-0.32192628
174070	4/4/2019 13:38	A	87776.94037	20015.05963	-73.283	-41.954	2934	-0.427919195	-2.713571603	-0.299848115
174070	4/4/2019 14:11	B	11515057.43	2285309.065	-73.296	-41.97	1960	-0.386296602	-2.866475189	-0.282450627
174070	4/4/2019 15:17	1	8244859.131	7176573.369	-73.258	-42.036	3965	0.368666517	-2.866475189	-0.55864189
174070	4/4/2019 20:50	1	5356653.335	4998387.665	-73.311	-41.957	20007	-0.332913989	-2.866475189	-0.302637629
174070	4/4/2019 22:36	B	14856502.08	4632493.923	-73.33	-41.946	6343	0.326116269	-2.866475189	-0.367542592
174070	4/4/2019 23:00	1	211741.25	211741.25	-73.335	-41.913	1433	1.531407393	-2.866475189	-0.585250758
174070	4/5/2019 0:12	1	3947586.135	2603006.365	-73.336	-41.945	4298	-0.464170471	-2.866475189	-0.877223192
174070	4/5/2019 0:35	A	31046874	10439786	-73.338	-41.945	1399	-0.459413702	-2.866475189	-0.880217937
174070	4/5/2019 1:58	A	495333008.1	65801610.42	-73.351	-41.929	5004	-0.323994908	-2.866475189	-0.932610217
174070	4/5/2019 2:17	A	3469116.586	878260.4139	-73.352	-41.933	1130	-0.324888988	-2.866475189	-0.93334972
174070	4/5/2019 2:28	B	1664396.327	367415.6729	-73.353	-41.932	630	-0.324888988	-2.866475189	-0.93334972
174070	4/5/2019 3:33	1	5421823.134	33439.36612	-73.334	-41.928	3934	-0.339256266	-2.866475189	-0.887666756

174070	4/5/2019 4:11	B	2055949.002	1377317.498	-73.337	-41.923	2263	-0.293699848	-2.866475189	-0.904250219
174070	4/5/2019 5:16	B	6216491.692	2435902.308	-73.338	-41.914	3896	-0.215052252	-2.866475189	-0.916964981
174070	4/5/2019 8:24	2	145691.8374	29564.66263	-73.355	-41.902	11312	-0.091169924	-2.792485344	-0.984210831
174070	4/5/2019 10:06	1	4102830.424	899910.0763	-73.35	-41.95	6118	-0.444868893	-2.866475189	-0.908519065
174070	4/5/2019 10:18	2	58908.75985	31873.74015	-73.359	-41.936	670	-0.290336739	-2.866475189	-0.958914787
174070	4/5/2019 10:41	B	12672645.35	3326291.153	-73.363	-41.934	1411	-0.282648883	-2.866475189	-0.962220163
174070	4/5/2019 12:00	A	1507757.371	798695.6286	-73.313	-41.939	4731	-0.461526257	-2.866475189	-0.81947024
174070	4/5/2019 12:16	2	143920.3469	69150.15314	-73.327	-41.93	978	-0.376081896	-2.866475189	-0.867173385
174070	4/5/2019 12:21	1	263849.9295	297415.0705	-73.327	-41.929	296	-0.360081322	-2.866475189	-0.865684565
174070	4/5/2019 12:39	3	14060.06273	13512.43727	-73.346	-41.932	1096	-0.332901394	-2.866475189	-0.91779539
174070	4/5/2019 13:12	2	1063671.03	174333.9703	-73.363	-41.932	1954	-0.282648883	-2.866475189	-0.962220163
174070	4/5/2019 13:48	B	2523961.501	742304.9993	-73.336	-41.933	2157	-0.36805883	-2.866475189	-0.895411135
174070	4/5/2019 13:53	1	817236.4412	4901853.559	-73.352	-41.941	327	-0.380516555	-2.866475189	-0.922273095
174070	4/5/2019 14:14	2	139664.1174	313836.8826	-73.365	-41.94	1245	-0.317903316	-2.866475189	-0.959506078
174070	4/5/2019 15:00	B	3892886.121	1103436.379	-73.358	-41.942	2765	-0.348926709	-2.866475189	-0.945582367
174070	4/5/2019 20:20	B	1137202.508	19605585.49	-73.374	-41.927	19194	-0.187667847	-2.866475189	-1.012693436

174070	4/5/2019 22:01	1	58726919.63	463850.8744	-73.38	-41.898	6043	-0.056646079	-2.702138696	-1.092255809
174070	4/5/2019 22:24	A	56946987.72	6755614.78	-73.392	-41.907	1420	-0.076642576	-2.841084431	-1.112972191
174070	4/5/2019 23:38	B	13958970.16	13020301.84	-73.382	-41.902	4428	-0.06641912	-2.773057708	-1.08373598
174070	4/6/2019 0:01	B	4021966.452	36898.54795	-73.374	-41.891	1365	0.309467235	-2.630700225	-0.436798455
174070	4/6/2019 0:14	1	25136399.4	956907.1027	-73.394	-41.878	752	0.281505048	-2.392630943	-0.467783512
174070	4/6/2019 0:35	A	14415484.47	3764352.527	-73.387	-41.89	1279	0.271676717	-2.587363947	-0.488741113
174070	4/6/2019 0:59	B	9571452.975	2316633.525	-73.387	-41.89	1422	0.275011688	-2.61624482	-0.483718455
174070	4/6/2019 1:33	2	64872.697	90265.303	-73.353	-41.873	2065	0.37836325	-2.353638606	-0.290847791
174070	4/6/2019 2:09	B	17750453.69	4226267.312	-73.363	-41.888	2151	0.34516422	-2.546569876	-0.37648475
174070	4/6/2019 2:18	3	104109.611	3972.389029	-73.368	-41.893	567	0.327915647	-2.653198694	-0.40888373
174070	4/6/2019 2:33	B	33646539.1	1795553.398	-73.358	-41.892	908	0.347855733	-2.628520106	-0.37633227
174070	4/6/2019 3:13	1	3281666.358	2489196.142	-73.334	-41.892	2344	0.406417766	-2.641357821	-0.252972653
174070	4/6/2019 4:01	B	2445719.035	393583.4647	-73.348	-41.894	2935	0.373290739	-2.649886875	-0.329555861
174070	4/6/2019 8:10	2	167833.8349	95091.16509	-73.338	-41.941	14900	0.321407718	-2.866475189	-0.41007144
174070	4/6/2019 10:08	2	2026485.506	274094.4943	-73.333	-41.949	7113	0.327476658	-2.866475189	-0.395901425
174070	4/6/2019 11:51	1	9665909.336	375469.1641	-73.345	-41.862	6144	0.398983079	-1.961908484	-0.220118702

174070	4/6/2019 12:06	3	442581.0526	5981.447448	-73.343	-41.845	905	0.399712173	-1.595207784	-0.167702042
174070	4/6/2019 12:21	A	850235.8424	102445.1576	-73.346	-41.824	925	0.404316598	-1.145494568	-0.116117889
174070	4/6/2019 13:28	B	140208368.7	20661728.31	-73.37	-41.844	4026	0.344908682	-1.493857002	-0.305260052
174070	4/6/2019 13:45	A	252876.2017	220536.2983	-73.392	-41.846	988	0.300832001	-1.583247859	-0.385453306
174070	4/6/2019 13:56	B	12082194.12	303830.8849	-73.392	-41.852	685	0.299448449	-1.717138039	-0.411228805
174070	4/6/2019 14:32	B	20240612.43	3659184.57	-73.425	-41.876	2173	0.185265751	-2.406537382	-0.59325392
174070	4/6/2019 20:29	A	32227607.7	121207477.3	-73.384	-42.019	21373	-0.01815836	-2.866475189	-0.756253444
174070	4/6/2019 21:28	0	1190288.396	20651336.6	-73.325	-42.074	3535	0.043459976	-2.866475189	-0.648319901
174070	4/6/2019 22:13	A	2363556087	106790194.8	-73.306	-42.098	2737	0.123946719	-2.866475189	-0.548319539
174070	4/6/2019 23:08	2	122108.6805	19991.31945	-73.237	-42.112	3311	0.277348859	-2.866475189	-0.363556691
174070	4/6/2019 23:55	1	506633.0309	1283446.969	-73.147	-42.103	2775	0.556456054	-2.866475189	0.090151942
174070	4/7/2019 0:36	B	5163886.385	1702550.615	-73.123	-42.115	2513	-0.935900235	-2.866475189	1.765880815
174070	4/7/2019 1:12	A	6233862.362	21630574.64	-73.096	-42.079	2131	-0.913057698	-2.866475189	1.837830443
174070	4/7/2019 1:31	3	36072.75333	17129.74667	-73.092	-42.053	1135	-0.905327998	-2.768423873	1.878416059
174070	4/7/2019 2:18	2	326995.9896	67697.01043	-73.11	-42.053	2800	-0.907861751	-2.67528679	1.86454331
174070	4/7/2019 2:59	B	12100619.64	3634092.862	-73.096	-42.048	2493	-0.904897809	-2.735740101	1.88110945

174070	4/7/2019 3:27	B	33605111.41	4865701.586	-73.103	-42.046	1666	-0.905583393	-2.677376222	1.877350436
174070	4/7/2019 3:59	B	28663231.68	2312756.823	-73.096	-42.018	1925	-0.893082421	-2.462851988	1.925562469
174070	4/7/2019 4:31	0	71261688.75	867927.2533	-73.104	-42.045	1947	-0.904959776	-2.663610397	1.878390742
174070	4/7/2019 8:00	A	296601805.5	35197684.96	-73.144	-42.008	12507	-0.900013821	-1.817078332	1.891301916
174070	4/7/2019 9:29	B	652143.3507	24744345.15	-73.235	-41.973	5338	-0.906278605	-1.950592923	1.874436367
174070	4/7/2019 9:39	B	5078617.938	160840254.6	-73.225	-41.982	619	-0.904751065	-1.865181069	1.879542755
174070	4/7/2019 9:43	B	1024975.202	20654267.3	-73.225	-41.975	256	-0.90475454	-1.865181069	1.878256812
174070	4/7/2019 11:07	1	52331080.41	2365884.593	-73.241	-42.019	5026	-0.915945316	-2.70613172	1.802615584
174070	4/7/2019 11:23	0	29843052.81	1183197.193	-73.235	-42.031	951	-0.917975176	-2.822336168	1.795144801
174070	4/7/2019 11:55	A	988692.1495	1216920.851	-73.212	-42.035	1946	-0.91764508	-2.783015575	1.808584329
174070	4/7/2019 12:37	2	27540.4396	171302.0604	-73.143	-42.021	2499	-0.901568481	-2.126039869	1.885217673
174070	4/7/2019 12:46	A	543663.3984	52306.60157	-73.143	-42.019	519	-0.901568481	-2.013340097	1.885217673
174070	4/7/2019 13:00	A	2070996.385	2680686.115	-73.154	-42.019	851	-0.904054291	-2.03344752	1.877044417
174070	4/7/2019 13:10	B	15979230.13	11256072.37	-73.158	-42.011	627	-0.90069363	-1.844246106	1.888776749
174070	4/7/2019 13:37	2	206300.214	233869.786	-73.13	-41.985	1607	-0.891631191	-1.499914833	1.946693566
174070	4/7/2019 14:10	1	3368584.821	11391.17912	-73.145	-41.982	1981	-0.892820847	-1.348892718	1.942821399

174070	4/7/2019 20:57	0	360325.9396	14454976.56	-73.432	-41.929	24406	-0.903762175	-2.866475189	1.723771655
174070	4/7/2019 21:53	A	70396729.17	15320800.83	-73.494	-41.97	3359	-0.904656251	-2.040745042	1.605778894
174070	4/7/2019 22:37	B	5895438.19	2493686.31	-73.507	-41.982	2622	-0.904965032	-2.004088905	1.574000502
174070	4/7/2019 23:44	1	598080.4724	3210984.528	-73.392	-41.956	4011	-0.910037837	-2.866475189	1.74087785
174070	4/8/2019 0:11	0	1036763.254	3817769.746	-73.366	-41.963	1630	0.239875202	-2.866475189	0.789592067
174070	4/8/2019 0:19	B	9032853.576	347927.4236	-73.35	-41.973	485	0.268022073	-2.866475189	0.79447342
174070	4/8/2019 1:08	2	168787.8073	49306.69266	-73.375	-41.983	2943	0.263978296	-2.866475189	0.688087022
174070	4/8/2019 2:00	B	4022654.781	1126450.219	-73.381	-42.002	3111	0.294413116	-2.866475189	0.59196241
174070	4/8/2019 2:28	2	492018.4832	36055.51679	-73.385	-42.004	1695	0.287430104	-2.866475189	0.568682525
174070	4/8/2019 3:06	1	32791483.59	3468328.91	-73.387	-41.993	2292	0.268968582	-2.866475189	0.607547692
174070	4/8/2019 3:32	2	11930.08176	248440.4182	-73.381	-41.996	1582	0.27975416	-2.866475189	0.623493598
174070	4/8/2019 3:35	2	114308.0186	190044.4814	-73.373	-42.002	173	0.299815499	-2.866475189	0.613465817
174070	4/8/2019 4:16	1	316204.0364	501184.4636	-73.384	-41.973	2436	0.237767423	-2.866475189	0.70766525
174070	4/8/2019 9:27	2	1305481.192	14101.30813	-73.383	-41.97	18666	0.234444969	-2.866475189	0.722017383
174070	4/8/2019 10:45	B	6554059.798	1198365.202	-73.386	-41.983	4661	0.25238676	-2.866475189	0.656717053
174070	4/8/2019 11:07	B	2723139.844	386882.6558	-73.389	-41.991	1352	0.264985268	-2.866475189	0.617267412

174070	4/8/2019 11:37	1	3274401.25	3274401.25	-73.38	-42.053	1803	0.378011561	-2.763891721	0.370811523
174070	4/8/2019 11:42	A	1393720.351	877260.6494	-73.373	-42.053	271	0.38240226	-2.805160932	0.379177714
174070	4/8/2019 12:14	B	2735649.793	1047994.707	-73.366	-42.069	1900	0.41817019	-2.771219112	0.33150066
174070	4/8/2019 13:21	1	5896618.611	780222.3895	-73.251	-42.068	4029	0.574705383	-2.866475189	0.646630208
174070	4/8/2019 14:32	B	53377634.39	13461208.11	-73.153	-42.091	4257	0.738984022	-2.866475189	0.880416251
174070	4/8/2019 15:38	B	19572034.24	4848650.756	-73.055	-42.114	3955	0.890334239	-2.866475189	1.092476096
174070	4/8/2019 22:06	B	336096068.1	66655052.41	-72.889	-41.643	23307	-0.984874033	-2.866475189	2.65057753
174070	4/8/2019 23:00	B	31837514	10614746	-72.812	-41.623	3237	-1.078188014	-2.866475189	2.736226396
174070	4/9/2019 0:36	0	4556606.299	8301150.201	-72.837	-41.789	5785	-0.575505354	-2.866475189	2.138003265
174070	4/9/2019 1:34	0	74748592.14	281161712.9	-72.826	-41.805	3468	-0.619205501	-2.866475189	2.144664666
174070	4/9/2019 2:09	0	5035222.36	833757.6396	-72.759	-41.784	2099	-0.743764907	-2.846268669	2.265146346
174070	4/9/2019 2:50	B	113115840.5	34345472	-72.683	-41.772	2446	-0.819123404	-2.361430165	2.355338468
174070	4/9/2019 3:23	B	705750968.3	49917370.22	-72.633	-41.759	1964	-0.836978028	-2.293425303	2.386923082
174070	4/9/2019 3:56	B	3381657218	1426175195	-72.731	-41.789	1980	-0.80261977	-2.488287301	2.323143364
174070	4/9/2019 9:15	0	146545863.9	4569476.638	-73.103	-41.99	19151	1.845253603	-1.883399985	0.3251933
174070	4/9/2019 10:17	2	106152.1744	74328.32563	-73.189	-42.018	3731	1.622481327	-2.324036765	-0.286031411

174070	4/9/2019 10:59	B	20815260.77	5866451.734	-73.202	-42.027	2494	1.561698759	-2.587502395	-0.385884129
174070	4/9/2019 11:30	B	21709685.39	7134190.612	-73.173	-42.021	1873	1.746088719	-2.252640442	-0.236692399
174070	4/9/2019 11:56	B	44550628.43	5028890.072	-73.176	-42.014	1549	1.699841134	-2.098954559	-0.179632353
174070	4/9/2019 12:56	A	61550.1883	96336.3117	-73.118	-41.956	3637	1.500630329	-1.308483181	0.49547632
174070	4/9/2019 13:07	1	438366.834	678838.166	-73.116	-41.958	663	1.533350911	-1.321221709	0.487076065
174070	4/9/2019 13:34	A	1094066.479	596158.5208	-73.146	-41.929	1602	1.26380666	-1.334759443	0.540566365
174070	4/9/2019 14:07	A	4780592.663	9437.836908	-73.124	-41.964	1985	1.564128506	-1.319550584	0.41085831
174070	4/9/2019 14:34	B	5145642.482	186862.5176	-73.118	-41.987	1639	1.792592526	-1.652203804	0.265665037
174070	4/9/2019 14:50	B	834632	201612.5	-73.112	-41.964	929	1.583686144	-1.397371597	0.461774224
174070	4/9/2019 19:57	B	42810859.29	10575413.71	-73.097	-42.016	18426	2.166308	-2.416280081	0.131581725
174070	4/9/2019 21:39	A	16457179.82	212948.6798	-73.129	-42.189	6108	0.682831716	-2.658418549	-1.509436669
174070	4/9/2019 23:15	1	293608.774	86252.22601	-73.097	-42.19	5762	0.97982742	-2.866475189	-1.429451317
174070	4/10/2019 0:14	A	241257754.2	12874234.26	-73.054	-42.196	3550	-0.906185888	-2.866475189	-0.970801085
174070	4/10/2019 1:12	2	26899.65249	187830.3475	-73.126	-42.21	3513	-0.927518099	-2.554942889	-0.990699688
174070	4/10/2019 1:28	1	55192447.72	2103669.277	-73.148	-42.186	920	-0.773132915	-2.506731935	-0.945420359
174070	4/10/2019 2:25	B	3070409.959	27694158.54	-73.146	-42.195	3430	-0.797311266	-2.375544557	-0.960164741

174070	4/10/2019 2:59	B	8672031.715	8116940.785	-73.147	-42.199	2059	-0.812135307	-2.318735863	-0.969682244
174070	4/10/2019 3:07	2	403630.7221	39697.77786	-73.141	-42.201	476	-0.833290593	-2.357893976	-0.971560117
174070	4/10/2019 3:28	2	334182.2257	6547.774272	-73.159	-42.179	1242	-0.775050737	-2.558932061	-0.927997334
174070	4/10/2019 4:32	B	11191670.91	5502845.59	-73.161	-42.194	3824	-0.783455364	-2.333297523	-0.952803107
174070	4/10/2019 5:08	B	3651842.995	1324223.505	-73.165	-42.199	2166	-0.770354717	-2.24915926	-0.966915734
174070	4/10/2019 7:26	B	175333953.5	5457099.54	-73.167	-42.231	8310	-0.729244126	-1.944243263	-1.028847019
174070	4/10/2019 9:01	0	97042739589	3666639015	-73.114	-42.188	5678	-0.861816992	-2.587298229	-0.974086643
174070	4/10/2019 9:58	B	108858628.1	5802674.402	-73.103	-42.19	3454	-0.800710064	-2.783457515	-0.960062242
174070	4/10/2019 11:18	0	202128.8483	12370407.65	-73.169	-42.225	4769	-0.724660075	-2.011403996	-1.020379103
174070	4/10/2019 11:35	B	722117.78	808062.22	-73.131	-42.256	1017	-0.890328966	-1.971982099	-1.059727675
174070	4/10/2019 12:37	A	564386.657	528965.343	-73.222	-42.226	3712	-0.470166153	-2.689008244	-1.046466563
174070	4/10/2019 13:03	A	117398607.6	3004390.915	-73.208	-42.214	1597	-0.590275936	-2.630361837	-1.006003918
174070	4/10/2019 13:14	B	76767012.2	4346755.804	-73.207	-42.213	642	-0.580887205	-2.71177279	-1.00574843
174070	4/10/2019 14:38	A	949703.0836	1168047.416	-73.377	-42.132	5040	-0.263336432	-1.749723633	-1.326498504
174070	4/10/2019 15:00	1	522211.6197	1409608.88	-73.391	-42.096	1339	-0.238993534	-2.160448945	-1.415587774
174070	4/10/2019 21:22	B	23607536.97	90905692.03	-73.213	-41.853	22905	-0.733426914	-2.548349151	-1.338902554

174070	4/10/2019 22:45	B	4245316.985	931209.5152	-73.254	-41.893	4944	-0.768363516	-2.738950462	-1.378730078
174070	4/10/2019 23:47	B	3067764.5	515112.5	-73.217	-41.999	3738	-0.326039002	-2.235929725	-1.12032512
174070	4/11/2019 1:28	2	91252.86776	174648.1322	-73.263	-42.186	6078	0.958641545	-2.866475189	-0.626182372
174070	4/11/2019 2:28	0	14399012400	650560940.7	-73.278	-42.185	3589	0.513525736	-2.866475189	-0.577449472
174070	4/11/2019 3:03	2	199105.8658	109276.6342	-73.261	-42.155	2124	-0.429241786	-2.866475189	-0.51149773
174070	4/11/2019 4:10	0	45818318.7	3820363.297	-73.316	-42.085	4024	0.64874518	-2.866475189	-0.737538922
174070	4/11/2019 4:53	B	2138644.749	13489608.25	-73.346	-42.067	2529	1.916347764	-2.866475189	-0.981110829
174070	4/11/2019 7:10	B	1310164.604	2394457.896	-73.409	-42.041	8236	1.550390596	-2.648857249	-1.711719329
174070	4/11/2019 8:57	0	161373138.8	13294782.25	-73.234	-42.235	6425	1.74052155	-2.866475189	-0.950428177
174070	4/11/2019 9:26	1	823057.472	2807759.028	-73.265	-42.221	1754	1.827550794	-2.866475189	-0.905642801
174070	4/11/2019 10:29	B	32711329.19	9038915.311	-73.256	-42.236	3754	1.541023948	-2.866475189	-1.001915876
174070	4/11/2019 11:04	B	15842019.94	14995766.56	-73.255	-42.244	2125	1.390339566	-2.700513316	-1.045224077
174070	4/11/2019 12:20	B	2554607.724	1010692.276	-73.269	-42.241	4543	1.283669143	-2.780092449	-1.065739355
174070	4/11/2019 12:41	A	57093529.93	5248642.57	-73.287	-42.242	1242	0.93351622	-2.53365095	-1.124510553
174070	4/11/2019 12:53	B	82817942.23	8522166.27	-73.288	-42.241	770	0.934119189	-2.491617676	-1.126747679
174070	4/11/2019 13:55	A	853706.8952	141525.6048	-73.292	-42.207	3684	1.848355876	-2.590806364	-0.910442604

174070	4/11/2019 14:34	1	1303248.956	4374113.544	-73.283	-42.213	2346	1.828640336	-2.738354138	-0.91298189
174070	4/11/2019 20:34	B	49446461.24	52777939.26	-73.328	-42.173	21588	1.542763374	-2.357480377	-0.903383942
174070	4/11/2019 21:07	B	18888960.73	12133455.27	-73.316	-42.16	2013	1.294244326	-2.692225634	-0.787078259
174070	4/11/2019 22:13	B	513870143.3	12891460.65	-73.311	-42.146	3961	0.400442327	-2.866475189	-0.620739783
174070	4/11/2019 22:50	3	102706.2314	6293.768554	-73.262	-42.145	2185	-0.537365307	-2.866475189	-0.514328309
174070	4/11/2019 23:19	0	189938818	1101953.988	-73.273	-42.155	1786	-0.22620518	-2.866475189	-0.532017109
174070	4/12/2019 0:32	B	1510347.8	3653270.7	-73.268	-42.151	4326	-0.443251398	-2.866475189	-0.690367729
174070	4/12/2019 1:08	1	872512.151	84455.84902	-73.3	-42.161	2160	1.046236318	-2.83847401	-0.838492961
174070	4/12/2019 1:10	B	3262502.534	858134.4657	-73.301	-42.162	131	1.114025103	-2.83847401	-0.844612318
174070	4/12/2019 2:10	B	6515777898	1063642191	-73.324	-42.172	3594	-0.045426647	-2.866475189	-0.703198734
174070	4/12/2019 2:50	A	49359187.54	3571234.958	-73.232	-42.172	2394	-0.54212561	-2.855544331	-0.667356864
174070	4/12/2019 3:27	B	84902742.57	20246542.43	-73.209	-42.179	2246	-0.247386428	-2.809653678	-0.640924046
174070	4/12/2019 3:48	B	570414069	93252564.96	-73.231	-42.155	1241	-0.55268889	-2.858927692	-0.630280437
174070	4/12/2019 4:31	B	2577685.601	371142.8991	-73.205	-42.167	2604	-0.3487225	-2.820584535	-0.607985938
174070	4/12/2019 7:00	B	9666163.147	577173.3532	-73.125	-42.186	8931	-0.026807844	-2.672767283	-0.514043891
174070	4/12/2019 8:40	A	6261347.874	4735810.626	-73.399	-42.035	5988	2.249214694	-2.799808735	-1.822079868

174070	4/12/2019 10:22	1	152736.5442	141325.9558	-73.477	-41.933	6167	0.530931205	-2.089275345	-1.687560383
174070	4/12/2019 10:38	B	3500658	950820.5	-73.482	-41.925	905	0.175224455	-2.018983216	-1.660145042
174070	4/12/2019 12:04	1	131934.8645	1573461.636	-73.511	-41.918	5203	-0.133663763	-1.461405466	-1.744610434
174070	4/12/2019 12:22	2	61581.08526	388986.9147	-73.498	-41.924	1071	0.131876818	-1.579391789	-1.729440366
174070	4/12/2019 12:37	A	6954286.563	833649.4373	-73.501	-41.93	887	0.181650347	-1.573132033	-1.753293356
174070	4/12/2019 13:35	B	1836346614	69435108.23	-73.513	-41.907	3498	-0.268554045	-2.055963419	-1.635299798
174070	4/12/2019 14:07	0	130420981.8	40191900.75	-73.444	-41.921	1908	-0.398198194	-2.54241519	-1.577605105
174070	4/12/2019 14:16	B	119142928.8	9174083.154	-73.46	-41.902	555	-0.480762897	-2.471859306	-1.595887428
174070	4/12/2019 15:12	B	82407444.12	11989028.88	-73.453	-41.886	3356	-0.519168042	-2.321955604	-1.628188034
174070	4/12/2019 20:04	B	114455793	32251159.95	-73.445	-41.809	17484	-1.00646673	-1.023704205	-1.682589654
174070	4/12/2019 20:56	B	16515356.74	12973565.26	-73.465	-41.905	3159	-0.465931187	-2.311666983	-1.605516726
174070	4/12/2019 21:40	2	578868.3903	17292.10966	-73.439	-41.914	2629	-0.419339929	-2.782379449	-1.571258183
174070	4/12/2019 22:38	A	185362.4284	115440.0716	-73.434	-41.918	3479	-0.367177471	-2.866475189	-1.571214367
174070	4/12/2019 22:55	A	1786054.3	296075.7004	-73.42	-41.931	1028	-0.078519498	-2.866475189	-1.586165515
174070	4/12/2019 23:21	2	110359.5416	36270.95838	-73.425	-41.95	1555	0.878421311	-2.866475189	-1.660931596
174070	4/13/2019 0:19	B	32681254.3	1781932.197	-73.427	-41.933	3474	-1.047685511	-2.866475189	-1.625704696

174070	4/13/2019 0:36	A	904237.546	2074.954032	-73.414	-41.92	1022	-0.934510688	-2.866475189	-1.641706559
174070	4/13/2019 1:00	B	24812926.9	2353307.599	-73.431	-41.923	1458	-0.944919876	-2.866475189	-1.635490739
174070	4/13/2019 2:17	B	2535584.525	4065292.475	-73.417	-41.913	4594	-0.824690113	-2.866475189	-1.653637197
174070	4/13/2019 2:27	2	398787.7959	17140.70411	-73.442	-41.906	625	-0.732094195	-2.685753147	-1.672270652
174070	4/13/2019 2:32	2	664100.3105	75328.18947	-73.444	-41.897	291	-0.681554157	-2.606566686	-1.691196567
174070	4/13/2019 3:08	B	15063478.74	4701163.764	-73.446	-41.891	2131	-0.675791374	-2.531821528	-1.699443955
174070	4/13/2019 3:29	B	87118964.49	414029.5107	-73.445	-41.901	1259	-0.706421712	-2.650751849	-1.681124007
174070	4/13/2019 8:31	A	264721.4865	771912.5135	-73.455	-41.911	18117	-0.731255138	-2.541691643	-1.666018807
174070	4/13/2019 10:07	0	19272492.81	834915.6876	-73.433	-41.87	5785	-0.737994699	-2.245974306	-1.747676653
174070	4/13/2019 10:16	1	110277.6199	255915.3801	-73.429	-41.862	529	-0.767009516	-1.974789036	-1.760653209
174070	4/13/2019 11:31	A	28309458.96	313871.0438	-73.417	-41.86	4527	-0.860617926	-1.917356041	-1.76360532
174070	4/13/2019 11:58	A	874472.9763	2052299.524	-73.399	-41.845	1608	-0.975764414	-1.547072228	-1.776049271
174070	4/13/2019 12:10	B	1436096.625	900573.875	-73.399	-41.845	691	-0.97021299	-1.547072228	-1.774747996
174070	4/13/2019 12:22	A	71131.14605	204419.3539	-73.369	-41.834	764	-0.970850685	-1.313192876	-1.752644808
174070	4/13/2019 13:09	1	1105169.616	274174.3835	-73.319	-41.845	2817	-1.149450215	-1.663646951	-1.688853072
174070	4/13/2019 13:52	0	56400752.5	5318745.997	-73.333	-41.834	2571	-1.053486792	-1.388908446	-1.702508998

174070	4/13/2019 14:55	B	1197102.553	363123.4474	-73.222	-41.846	3784	-0.768156753	-2.423512081	-1.578647888
174070	4/13/2019 15:33	B	1057173.135	7758867.365	-73.156	-41.84	2285	-0.685211211	-1.532605887	-1.529426059
174070	4/13/2019 20:44	A	33753207.48	68809997.02	-73.053	-41.936	18629	-0.211357612	-1.658967903	-1.431293074
174070	4/13/2019 22:26	1	11793983.71	1746218.293	-73.106	-41.914	6151	-0.462518353	-1.229960533	-1.415969213
174070	4/13/2019 22:29	B	2112926.038	365558.9618	-73.107	-41.914	171	-0.461604753	-1.229960533	-1.41413322
174070	4/14/2019 0:08	A	36708529.84	974920.1559	-73.126	-41.906	5922	-0.886033117	-1.331429392	-2.143005946
174070	4/14/2019 0:12	0	125595430.9	11766991.61	-73.124	-41.899	241	-0.877120483	-1.360150626	-2.141152404
174070	4/14/2019 0:43	B	7354970.133	881127.8665	-73.147	-41.925	1841	-0.622824194	-1.348350104	-2.110457942
174070	4/14/2019 1:27	2	165291.2635	13185.73647	-73.195	-41.901	2694	-0.526889298	-1.833774478	-1.972499762
174070	4/14/2019 2:09	2	112070.5524	15879.94756	-73.194	-41.893	2483	-0.531255065	-1.973586382	-1.979843579
174070	4/14/2019 2:21	1	942179.7189	45132.78111	-73.202	-41.894	743	-0.548594214	-2.088749594	-1.964190628
174070	4/14/2019 3:09	3	49198.59896	6901.901042	-73.22	-41.889	2896	-0.590837098	-2.36738996	-1.929505305
174070	4/14/2019 4:02	B	11555951.19	1426575.31	-73.247	-41.903	3159	-0.772830667	-2.722258024	-1.869185282
174070	4/14/2019 8:16	3	49475.41795	7247.082046	-73.216	-41.939	15243	-0.606949011	-1.635498891	-1.922789545
174070	4/14/2019 10:01	0	170252054	225266.0436	-73.247	-41.893	6285	-0.695790287	-2.622343868	-1.888131009
174070	4/14/2019 11:02	2	375275.5055	33437.49451	-73.192	-41.892	3666	-0.532426952	-1.976421385	-1.98729726

174070	4/14/2019 11:15	B	648091.8811	182373.1189	-73.192	-41.892	754	-0.532426952	-1.976421385	-1.98729726
174070	4/14/2019 11:34	A	1027258.67	1099417.83	-73.185	-41.906	1159	-0.482716469	-1.690737473	-1.997710921
174070	4/14/2019 11:52	B	2148634.701	1729205.299	-73.174	-41.906	1070	-0.458068285	-1.62825061	-2.031233977
174070	4/14/2019 12:08	1	1791549.501	345052.9987	-73.181	-41.909	982	-0.462143175	-1.652434488	-2.00769808
174070	4/14/2019 12:40	A	334056.8765	1712975.624	-73.19	-41.905	1932	-0.507071733	-1.707134313	-1.984561597
174070	4/14/2019 12:52	2	333810.1885	11110.3115	-73.158	-41.923	701	-0.482708163	-1.403027338	-2.079388705
174070	4/14/2019 13:29	1	3889109.348	157483.1516	-73.122	-41.93	2196	-0.826471075	-1.281658865	-2.144448714
174070	4/14/2019 13:52	1	90360.21347	1474516.787	-73.108	-41.924	1422	-0.787415901	-1.245346338	-2.143317937
174070	4/14/2019 14:33	A	299284.1621	1898277.838	-73.066	-41.93	2447	0.161146961	-1.477631359	-2.017674211
174070	4/14/2019 15:12	2	192506.4223	78214.07771	-73.05	-41.919	2307	0.283706924	-1.538123601	-1.966326946
174070	4/14/2019 20:33	A	13776200	13776200	-73.249	-41.909	19261	-0.825447066	-2.645806231	-1.853289154
174070	4/14/2019 20:40	A	6405567.314	5605985.686	-73.26	-41.906	444	-0.867788253	-2.866475189	-1.848409318
174070	4/14/2019 22:14	1	343853961.7	21376860.81	-73.244	-41.903	5630	-0.772830667	-2.722258024	-1.869185282
174070	4/14/2019 23:48	B	21028033928	6440856.605	-73.245	-41.898	5647	-0.739912505	-2.642423522	-1.876153265
174070	4/14/2019 23:57	B	410640511.1	7742765.904	-73.253	-41.904	538	-0.746677494	-2.578040859	-1.87534411
174070	4/15/2019 0:33	A	260902.3822	243610.1178	-73.22	-41.908	2145	-0.719771028	-2.099822294	-1.891454888

174070	4/15/2019 1:10	2	82026.08626	188538.9137	-73.234	-41.891	2219	-0.619051242	-2.605081578	-1.879104849
174070	4/15/2019 1:28	2	68107.32699	90662.67301	-73.213	-41.894	1074	-0.52873125	-2.261753728	-1.912738856
174070	4/15/2019 2:11	1	3687103.875	277204.6249	-73.217	-41.882	2638	-0.341894147	-2.501390459	-1.931397741
174070	4/15/2019 2:20	B	590192897.6	14811046.91	-73.216	-41.882	490	-0.359263672	-2.512101547	-1.929036651
174070	4/15/2019 3:25	3	14518.5	30786.5	-73.235	-41.883	3937	-0.431263536	-2.662151854	-1.898428796
174070	4/15/2019 3:58	B	2905132.563	189655.937	-73.237	-41.88	1988	-0.431263536	-2.688245531	-1.898428796
174070	4/15/2019 4:31	1	862984.0597	424127.9403	-73.237	-41.873	1980	-0.22849214	-2.724299822	-1.922407085
174070	4/15/2019 8:04	2	122933.6198	27288.88019	-73.188	-41.942	12769	0.048189233	-1.36224176	-1.993551683
174070	4/15/2019 9:25	3	44572.16359	42407.83641	-73.197	-41.965	4865	-0.157193575	-1.468573597	-1.974337314
174070	4/15/2019 10:29	1	718714.9936	129850.0064	-73.157	-41.968	3852	0.856294268	-1.298092919	-2.202727053
174070	4/15/2019 11:05	1	219942844.1	6839142.438	-73.167	-41.976	2153	0.941559355	-1.364181905	-2.152069059
174070	4/15/2019 11:32	B	13237199.85	8766332.652	-73.178	-41.986	1610	0.635633549	-1.562479607	-2.074289054
174070	4/15/2019 12:02	B	20213815.34	7910717.156	-73.198	-41.991	1784	-0.205343696	-1.844035708	-1.970262616
174070	4/15/2019 12:08	A	2928757.831	901011.1685	-73.202	-41.993	335	-0.287374359	-1.896774279	-1.960803191
174070	4/15/2019 12:29	3	51016.47766	17867.52234	-73.201	-41.985	1299	-0.289543863	-1.702505239	-1.961902386
174070	4/15/2019 12:45	B	494906.2992	146446.7008	-73.201	-41.987	967	-0.279197146	-1.808494481	-1.962199124

174070	4/15/2019 13:07	A	2028841.788	1072950.712	-73.218	-41.973	1320	-0.840493709	-1.753667687	-1.923192896
174070	4/15/2019 13:38	2	396762.0149	41618.48508	-73.249	-41.96	1869	-0.644259244	-2.099195562	-1.876506613
174070	4/15/2019 14:12	A	91605.30774	17427.19226	-73.236	-41.939	2002	-0.67716862	-1.97748531	-1.881738562
174070	4/15/2019 20:22	0	25700348.11	16873972.89	-73.085	-41.924	22239	0.517428184	-1.289863383	-2.137027732
174070	4/15/2019 21:47	1	440761.4485	1248201.052	-73.177	-41.996	5060	0.567056154	-1.721329758	-2.074131962
174070	4/15/2019 22:01	1	154096472.2	5824368.266	-73.183	-42.005	852	0.375739556	-1.960398032	-2.03769668
174070	4/15/2019 23:20	2	1131820.658	110145.8422	-73.203	-41.97	4737	-0.362581361	-1.591761637	-1.956484162
174070	4/15/2019 23:27	2	37814.85498	30635.14502	-73.199	-41.973	425	-0.188954871	-1.567745299	-1.969816833
174070	4/16/2019 0:18	1	305211.1341	474813.8659	-73.241	-41.967	3071	-0.902198224	-1.966276929	-1.744372832
174070	4/16/2019 1:03	2	30268.24049	100685.7595	-73.211	-41.934	2698	-0.769171191	-1.642208422	-1.783157754
174070	4/16/2019 1:25	2	73933.33742	33014.66258	-73.192	-41.915	1325	-0.768953764	-1.635792	-1.822199143
174070	4/16/2019 2:28	0	409289616.8	18495865.16	-73.084	-41.922	3747	-0.810869797	-1.27896148	-1.911785944
174070	4/16/2019 2:40	B	8806745.46	742090.54	-73.08	-41.92	754	-0.739678143	-1.273745815	-1.901855296
174070	4/16/2019 3:05	1	42119891.81	1593670.193	-73.144	-41.935	1475	-1.09482513	-1.282652312	-1.912129111
174070	4/16/2019 3:43	B	4886010.695	1080125.805	-73.152	-41.934	2271	-0.96926609	-1.311176784	-1.894302504
174070	4/16/2019 4:06	B	11793533.19	218819.8056	-73.159	-41.917	1388	-0.892522887	-1.454539168	-1.881212282

174070	4/16/2019 4:49	A	130473.875	314692.625	-73.216	-41.915	2584	-0.766888517	-2.003495813	-1.778512696
174070	4/16/2019 7:58	B	11337403.62	4419373.384	-73.314	-41.91	11366	-1.05163772	-2.866475189	-1.701237791
174070	4/16/2019 9:03	B	886197.3503	5813731.65	-73.253	-41.906	3891	-0.844566187	-2.745831984	-1.727165304
174070	4/16/2019 9:33	A	755246.2267	29305.77335	-73.309	-41.889	1817	-1.046184847	-2.653297348	-1.72331101
174070	4/16/2019 10:00	A	7118672.016	1552552.984	-73.246	-41.899	1596	-0.786197262	-2.705251777	-1.742571677
174070	4/16/2019 11:38	A	38194.57447	371937.9255	-73.328	-41.91	5851	-1.010050807	-2.866475189	-1.712929081
174070	4/16/2019 11:46	B	222915.2997	236985.7003	-73.331	-41.911	520	-1.004202698	-2.866475189	-1.715470525
174070	4/16/2019 12:13	1	32416792.85	665100.1517	-73.325	-41.921	1621	-0.955831886	-2.866475189	-1.712912922
174070	4/16/2019 12:26	B	18156500.16	385308.3397	-73.334	-41.934	744	-0.798424036	-2.866475189	-1.728386828
174070	4/16/2019 12:47	B	9363272.233	591995.767	-73.332	-41.934	1302	-0.798424036	-2.866475189	-1.728386828
174070	4/16/2019 13:28	2	320845.2508	27155.2492	-73.28	-41.925	2454	-1.070693147	-2.7843603	-1.689215055
174070	4/16/2019 13:46	B	1823646.408	499404.0922	-73.281	-41.925	1058	-1.073979732	-2.787292975	-1.689282473
174070	4/16/2019 14:28	A	32652.20578	247296.7942	-73.353	-41.945	2518	-0.591875628	-2.866475189	-1.777903718
174070	4/16/2019 15:33	A	344750.8254	16803429.67	-73.382	-41.921	3881	-0.725509564	-2.866475189	-1.793983058
174070	4/16/2019 21:17	2	257462.4463	33662.55371	-73.318	-41.92	20673	-1.006499061	-2.866475189	-1.703892987
174070	4/16/2019 22:00	B	7996662.704	1555039.796	-73.32	-41.938	2550	-0.833288553	-2.866475189	-1.719087208

174070	4/16/2019 22:58	B	18769850.98	2929817.519	-73.328	-41.946	3533	-0.698157165	-2.866475189	-1.739935931
174070	4/16/2019 23:31	B	23778138.93	2461045.067	-73.321	-41.947	1942	-0.745410255	-2.866475189	-1.730947685
174070	4/17/2019 0:26	B	1306089.051	10766775.95	-73.236	-41.943	3333	-1.033730926	-1.885920588	-1.037903393
174070	4/17/2019 0:33	B	583912.3044	1380944.696	-73.235	-41.943	417	-1.031537488	-1.885920588	-1.037179613
174070	4/17/2019 1:07	1	205036.9047	2340981.095	-73.211	-41.946	1999	-0.773955644	-1.488440979	-1.004563167
174070	4/17/2019 2:07	3	346010.0372	9034.462775	-73.271	-41.92	3642	-1.220888355	-2.747701868	-1.069298988
174070	4/17/2019 3:21	B	121675651.3	121675651.3	-73.286	-41.914	4438	-1.145578692	-2.866475189	-1.080664172
174070	4/17/2019 3:30	A	133443.9542	85438.54576	-73.285	-41.912	523	-1.145578692	-2.866475189	-1.080664172
174070	4/17/2019 4:23	B	899763.3826	311190.6174	-73.295	-41.907	3197	-1.064101211	-2.866475189	-1.092744067
174070	4/17/2019 9:26	2	1320430.311	18075.6885	-73.235	-41.944	18164	-1.033730926	-1.850900781	-1.037903393
174070	4/17/2019 10:15	B	182557651.7	137954160.3	-73.252	-41.932	2917	-1.186850815	-2.328389337	-1.053354022
174070	4/17/2019 11:09	2	1464133.076	123938.9241	-73.259	-41.924	3235	-1.206543022	-2.589337441	-1.060573649
174070	4/17/2019 11:36	B	2031611.506	1857452.994	-73.285	-41.922	1661	-1.220448513	-2.828249292	-1.074508239
174070	4/17/2019 12:01	A	125098090.6	55969.85592	-73.283	-41.962	1476	-1.141290111	-2.747701868	-1.084568886
174070	4/17/2019 12:48	B	11739063.47	2568349.026	-73.279	-41.933	2839	-1.258074894	-2.668519654	-1.069231176
174070	4/17/2019 13:21	1	651300.6196	540360.3804	-73.219	-41.904	1963	-0.98493786	-2.218022182	-1.037017744

174070	4/17/2019 13:28	0	241077624.2	9117704.31	-73.218	-41.899	433	-0.970044657	-2.191158731	-1.034258905
174070	4/17/2019 14:08	3	983709.8324	8490.167636	-73.212	-41.901	2374	-0.965344099	-2.129698342	-1.033078053
174070	4/17/2019 14:45	B	31014080.47	6621478.035	-73.163	-41.86	2263	-0.838288394	-2.01218403	-1.041009759
174070	4/17/2019 15:09	B	2378211.442	918513.558	-73.16	-41.846	1423	-0.508533067	-1.648946429	-1.074111988
174070	4/17/2019 15:48	B	4361316.565	1336632.435	-73.141	-41.829	2340	-0.299665178	-1.294604808	-1.134185189
174070	4/17/2019 20:47	B	53333975.49	8770378.512	-73.213	-41.884	17927	-0.976072349	-2.410107944	-1.054031225
174070	4/17/2019 21:39	B	4407402.313	155310.1875	-73.304	-41.917	3123	-1.121294674	-2.866475189	-1.090128002
174070	4/17/2019 22:26	A	68709604.47	2596762.034	-73.4	-41.885	2842	-0.109157016	-2.533155633	-1.266559837
174070	4/17/2019 22:32	B	4587790.791	220035.2086	-73.405	-41.884	355	-0.088856804	-2.524459843	-1.273572159
174070	4/17/2019 23:29	2	135979.7622	49806.7378	-73.371	-41.918	3414	-0.793076733	-2.866475189	-1.14626785
174070	4/18/2019 0:13	1	46017309.9	1745067.098	-73.298	-41.921	2639	-0.903858333	-2.866475189	-1.301741361
174070	4/18/2019 1:40	B	331975762.2	17713854.8	-73.41	-41.973	5226	-0.986867545	-2.866475189	-1.326576435
174070	4/18/2019 2:25	2	173420.9961	87837.50389	-73.355	-41.973	2707	-1.183459752	-2.866475189	-1.323380477
174070	4/18/2019 3:01	B	33127572.23	5356630.272	-73.357	-41.986	2110	-1.130018289	-2.866475189	-1.331731281
174070	4/18/2019 3:20	A	11206901.42	18303419.08	-73.352	-42.004	1188	-1.089320896	-2.866475189	-1.335119294
174070	4/18/2019 3:29	A	5767545.24	420663.2604	-73.347	-41.997	495	-1.114494341	-2.866475189	-1.332035269

174070	4/18/2019 4:08	A	13612232.1	4926870.399	-73.318	-41.988	2382	-0.929978787	-2.866475189	-1.305496411
174070	4/18/2019 7:27	B	93631251.28	14387313.72	-73.272	-41.983	11925	-0.446910475	-2.627372783	-1.19403074
174070	4/18/2019 9:15	1	283793814.6	10726507.36	-73.327	-41.924	6495	-1.000383479	-2.866475189	-1.320046047
174070	4/18/2019 10:31	B	33912848.6	8888919.399	-73.331	-41.913	4534	-1.001712741	-2.866475189	-1.321505869
174070	4/18/2019 11:24	A	75184201.01	176240245.5	-73.351	-41.952	3187	-1.159716452	-2.866475189	-1.321390472
174070	4/18/2019 12:11	A	3774984.359	176868.6406	-73.337	-41.975	2837	-1.117253973	-2.866475189	-1.321953068
174070	4/18/2019 13:04	1	6800696.039	260190.4613	-73.323	-41.943	3169	-0.989394355	-2.866475189	-1.31674801
174070	4/18/2019 13:09	2	1390447.498	396337.0018	-73.324	-41.948	305	-0.99689667	-2.866475189	-1.317200205
174070	4/18/2019 13:48	B	55519179.9	3461870.1	-73.321	-41.944	2341	-0.989394355	-2.866475189	-1.31674801
174070	4/18/2019 14:52	B	7607756.194	873703.8063	-73.318	-41.943	3801	-0.971337283	-2.866475189	-1.313813438
174070	4/18/2019 15:30	B	2931005.183	431924.8167	-73.317	-41.944	2339	-0.942854538	-2.866475189	-1.310939707
174070	4/18/2019 21:28	B	1133133157	691673282.8	-73.314	-41.954	21436	-1.130734274	-2.866475189	-1.321813539
174070	4/18/2019 21:53	1	141286.0645	1470122.436	-73.351	-41.964	1533	-1.173789446	-2.866475189	-1.320455867
174070	4/18/2019 23:08	2	394542.9414	79946.05859	-73.306	-41.983	4472	-0.752257701	-2.866475189	-1.283605604
174070	4/18/2019 23:30	2	49783.62071	26910.87929	-73.309	-42.007	1313	-0.821101147	-2.866475189	-1.279889938
174070	4/19/2019 1:22	3	73064.02623	4255.973768	-73.384	-41.982	6753	-1.099195745	-2.866475189	-1.593217632

174070	4/19/2019 1:30	3	12674	20066	-73.395	-41.978	434	-1.135390964	-2.866475189	-1.593984384
174070	4/19/2019 2:07	A	2131355.332	24637.16849	-73.403	-41.977	2259	-1.153740896	-2.866475189	-1.596041476
174070	4/19/2019 3:03	2	550094.4393	21746.56072	-73.433	-41.962	3337	-0.948493958	-2.866475189	-1.608371764
174070	4/19/2019 3:05	3	272731.5299	3293.470141	-73.43	-41.965	147	-0.997725984	-2.866475189	-1.60542544
174070	4/19/2019 3:45	A	26138967.27	133616.7341	-73.443	-41.969	2401	-0.840281842	-2.776900908	-1.623042144
174070	4/19/2019 7:21	A	1187362.781	341469.7189	-73.434	-41.986	12970	-0.895876086	-2.866475189	-1.631779535
174070	4/19/2019 9:02	0	462075553.1	20879687.89	-73.356	-41.997	6049	-1.054771516	-2.866475189	-1.591308011
174070	4/19/2019 9:26	B	5499275.767	1934472.733	-73.353	-41.997	1399	-1.040800355	-2.866475189	-1.595536317
174070	4/19/2019 10:08	0	837622826.7	13005945.77	-73.376	-42.029	2532	-1.16670488	-2.866475189	-1.573045952
174070	4/19/2019 11:05	1	120071476.5	9881366.028	-73.368	-42.021	3461	-1.168865055	-2.866475189	-1.574084308
174070	4/19/2019 11:09	B	4450445.855	504179.1446	-73.363	-42.022	205	-1.160566381	-2.866475189	-1.574837111
174070	4/19/2019 11:15	1	6202896.683	42055.81692	-73.361	-42.018	371	-1.156519232	-2.866475189	-1.57578544
174070	4/19/2019 11:47	A	1558794.809	32734.191	-73.385	-42.057	1911	-0.846854884	-2.707715061	-1.595929938
174070	4/19/2019 12:51	2	705913.9156	202043.0844	-73.388	-42.044	3819	-0.980421784	-2.771219112	-1.590770936
174070	4/19/2019 12:54	A	1203642.866	38042.13438	-73.389	-42.044	218	-0.980421784	-2.771219112	-1.590770936
174070	4/19/2019 13:23	A	5251894.648	4633615.852	-73.392	-42.037	1748	-0.984031007	-2.810909144	-1.595369473

174070	4/19/2019 14:02	B	9304731.353	979011.147	-73.396	-42.016	2294	-1.020653383	-2.866475189	-1.595967976
174070	4/19/2019 14:28	3	87617.66932	28667.33068	-73.432	-41.984	1560	-0.932272235	-2.866475189	-1.624338557
174070	4/19/2019 15:06	2	157625.3174	323914.6826	-73.449	-41.962	2308	-0.734407988	-2.645099179	-1.632316395
174070	4/19/2019 21:19	A	1569474.675	446683.8255	-73.359	-42.041	22364	-1.104512275	-2.866475189	-1.570154066
174070	4/19/2019 21:22	2	4346.587095	391431.9129	-73.361	-42.034	212	-1.17023122	-2.866475189	-1.570886847
174070	4/19/2019 23:02	1	549178.7995	909326.2005	-73.341	-41.976	6009	-1.039045825	-2.866475189	-1.627880306
174070	4/19/2019 23:24	1	537413.6448	59807.35522	-73.33	-41.958	1270	-1.072522853	-2.866475189	-1.638245919
174070	4/20/2019 1:01	3	144716.202	3223.798006	-73.303	-41.97	5862	-0.928832162	-2.866475189	-1.717407543
174070	4/20/2019 1:04	1	288824.5505	430127.9495	-73.311	-41.969	154	-0.955056262	-2.866475189	-1.71055673
174070	4/20/2019 1:16	A	2928421.842	188796.1579	-73.328	-41.978	708	-0.939035829	-2.866475189	-1.693782086
174070	4/20/2019 1:42	B	3879998.593	144971.4074	-73.332	-41.964	1581	-1.047339942	-2.866475189	-1.690058963
174070	4/20/2019 2:41	2	4460884.568	139764.4324	-73.415	-41.964	3534	-1.106758117	-2.866475189	-1.594851453
174070	4/20/2019 3:24	1	3365991.779	16862.72126	-73.437	-41.962	2560	-0.929093832	-2.823647134	-1.599995274
174070	4/20/2019 4:25	0	20545044.3	16688047.7	-73.402	-41.975	3670	-1.052285603	-2.866475189	-1.610216346
174070	4/20/2019 7:09	B	39787152.84	11667503.66	-73.421	-41.974	9837	-1.049562639	-2.866475189	-1.594829326
174070	4/20/2019 8:45	A	9041509.617	342114.3831	-73.4	-41.956	5785	-1.061648479	-2.866475189	-1.606070007

174070	4/20/2019 9:04	B	712915.0268	93127.47322	-73.401	-41.957	1155	-1.068378525	-2.866475189	-1.605125535
174070	4/20/2019 9:35	A	611350.5123	1422474.488	-73.398	-41.961	1842	-1.05688807	-2.866475189	-1.609442534
174070	4/20/2019 10:26	2	125929.6665	22288.83355	-73.414	-41.959	3063	-1.087600712	-2.866475189	-1.595565101
174070	4/20/2019 10:41	3	179953.3825	47311.11747	-73.417	-41.971	905	-1.079119229	-2.866475189	-1.594431832
174070	4/20/2019 10:59	B	245529.2826	110153.2174	-73.415	-41.969	1079	-1.078242214	-2.866475189	-1.59631772
174070	4/20/2019 11:17	A	4765261.326	452051.1744	-73.406	-41.979	1076	-1.061896679	-2.866475189	-1.604138296
174070	4/20/2019 12:26	A	5994787.486	2168471.014	-73.372	-41.95	4150	-0.981154886	-2.866475189	-1.644878311
174070	4/20/2019 12:38	A	1996815.422	25860.57772	-73.361	-41.965	687	-1.017513038	-2.866475189	-1.660627072
174070	4/20/2019 12:49	B	4258046.935	93035.06482	-73.402	-41.965	696	-1.062559253	-2.866475189	-1.607471661
174070	4/20/2019 13:06	B	1007255.397	712937.1034	-73.394	-41.965	1030	-1.023401243	-2.866475189	-1.615234048
174070	4/20/2019 14:05	A	44908175.66	3700476.84	-73.403	-42.003	3514	-0.99860039	-2.866475189	-1.625120329
174070	4/20/2019 14:19	B	8406643.609	293628.8913	-73.401	-42.007	826	-1.025961142	-2.866475189	-1.6282449
174070	4/20/2019 14:42	A	250645.9223	1033915.078	-73.383	-42.005	1399	-1.112631572	-2.866475189	-1.634861766
174070	4/20/2019 21:09	B	420897.7776	9702134.722	-73.331	-42.074	23190	-0.619400651	-2.866475189	-1.705098284
174070	4/20/2019 22:46	2	90205.49152	41179.00848	-73.253	-42.073	5835	-0.421903522	-2.866475189	-1.548695151
174070	4/20/2019 22:57	1	531903.093	1026189.407	-73.229	-42.088	691	-0.134909051	-2.866475189	-1.534112259

174070	4/21/2019 0:12	B	72931502.53	17284653.97	-73.238	-42.082	4456	-0.687045477	-2.866475189	-1.59400377
174070	4/21/2019 0:33	B	75872483.83	16723820.17	-73.235	-42.087	1300	-0.662182414	-2.866475189	-1.595507411
174070	4/21/2019 0:57	B	94139456.83	20807019.67	-73.242	-42.092	1412	-0.717662031	-2.866475189	-1.621826004
174070	4/21/2019 1:22	A	4271739.94	3715593.06	-73.296	-42.073	1502	-0.947261692	-2.866475189	-1.69527292
174070	4/21/2019 2:24	1	23849398.61	1718235.893	-73.349	-42.048	3707	-1.144392895	-2.866475189	-1.706949025
174070	4/21/2019 2:39	2	962778.6324	14046.36757	-73.347	-42.048	912	-1.14511609	-2.866475189	-1.707591524
174070	4/21/2019 2:59	2	12066899.57	750845.4348	-73.348	-42.034	1199	-1.212554167	-2.866475189	-1.695188241
174070	4/21/2019 4:04	A	6747137.592	365144.4083	-73.361	-42.01	3886	-1.220355507	-2.866475189	-1.679198344
174070	4/21/2019 4:47	1	442144.0698	615558.4302	-73.358	-41.991	2573	-1.200260931	-2.866475189	-1.675480971
174070	4/21/2019 8:40	2	1241355.027	53155.4731	-73.383	-42.03	13987	-1.101810955	-2.866475189	-1.687793035
174070	4/21/2019 8:40	B	523916.2011	46078.29894	-73.383	-42.03	27	-1.101810955	-2.866475189	-1.687793035
174070	4/21/2019 10:16	2	79175.64141	35972.85859	-73.307	-42.108	5768	-0.951364522	-2.866475189	-1.72870333
174070	4/21/2019 10:44	3	163821.2683	3981.231653	-73.278	-42.125	1667	-0.972120199	-2.866475189	-1.698261552
174070	4/21/2019 10:47	B	148626.1738	23054.32617	-73.28	-42.126	175	-0.990269926	-2.866475189	-1.70220886
174070	4/21/2019 11:54	A	97119532.05	475432.9462	-73.256	-42.129	4018	-0.969000366	-2.866475189	-1.686231644
174070	4/21/2019 12:06	B	3146537.464	47734.53642	-73.253	-42.131	752	-0.935874758	-2.866475189	-1.680105045

174070	4/21/2019 12:52	B	380967.2943	137401.2057	-73.159	-42.135	2710	-0.073435442	-2.866475189	-1.560419959
174070	4/21/2019 13:41	0	187698565	19860239.52	-73.127	-42.104	2952	-0.331048141	-2.866475189	-1.373110588
174070	4/21/2019 14:11	B	2945474.37	3623126.63	-73.074	-42.1	1794	-0.880559849	-2.866475189	-1.326244797
174070	4/21/2019 14:28	B	42358068.84	1697869.16	-73.052	-42.064	1066	-1.298730863	-2.866475189	-1.307957687
174070	4/21/2019 15:25	1	299199.7373	7614400.763	-73.023	-42.025	3379	-1.218507195	-2.866475189	-1.33023098
174070	4/21/2019 20:59	B	46579149.26	6787575.241	-72.893	-41.903	20045	-1.045624723	-1.899384218	-1.444038933
174070	4/21/2019 23:39	A	49356013.05	30289643.45	-73.324	-41.905	9585	-1.145952327	-2.866475189	-1.70592301
174070	4/22/2019 0:12	1	44211991.2	4138337.797	-73.33	-41.938	2012	-1.23620378	-2.866475189	-1.576872187
174070	4/22/2019 0:22	B	6197198	2479398	-73.343	-41.919	620	-1.2420019	-2.866475189	-1.585111565
174070	4/22/2019 0:45	1	798918.8613	40717.63875	-73.353	-41.952	1374	-1.174727534	-2.866475189	-1.590558896
174070	4/22/2019 1:05	B	7898556.213	2135934.287	-73.353	-41.953	1162	-1.174727534	-2.866475189	-1.590558896
174070	4/22/2019 1:50	B	31300995.13	1845767.367	-73.351	-41.954	2723	-1.191664514	-2.866475189	-1.582033563
174070	4/22/2019 1:59	1	6523337.015	169647.9848	-73.319	-41.968	549	-1.181638138	-2.866475189	-1.563929112
174070	4/22/2019 2:37	B	94486179.66	76689030.34	-73.306	-41.956	2272	-1.206465004	-2.866475189	-1.554291637
174070	4/22/2019 3:43	A	24613647.71	204297.2877	-73.336	-42.015	3937	-1.052212267	-2.866475189	-1.572530956
174070	4/22/2019 4:26	2	57798	149058	-73.329	-42.016	2609	-1.053481116	-2.866475189	-1.564930597

174070	4/22/2019 8:28	3	19329.71142	6952.288578	-73.3	-41.998	14506	-1.109659809	-2.866475189	-1.536874612
174070	4/22/2019 10:04	2	211031.0167	16453.98334	-73.345	-41.98	5777	-1.127790027	-2.866475189	-1.586041432
174070	4/22/2019 10:12	2	74344.27549	88159.72451	-73.355	-41.98	482	-1.10990916	-2.866475189	-1.59643891
174070	4/22/2019 11:32	2	1410295.753	29456.74677	-73.327	-42.01	4766	-1.064990576	-2.866475189	-1.562853946
174070	4/22/2019 12:13	A	63168800	7320.5	-73.325	-42.021	2498	-1.039198156	-2.866475189	-1.558486969
174070	4/22/2019 12:57	B	2341448	155124.5	-73.324	-42.028	2639	-1.020957282	-2.866475189	-1.553886988
174070	4/22/2019 13:11	B	2419329.642	219792.3577	-73.321	-42.027	837	-1.027575082	-2.866475189	-1.550670052
174070	4/22/2019 13:24	A	2090484831	31543911.58	-73.306	-42.027	782	-1.02718563	-2.866475189	-1.526507078
174070	4/22/2019 15:05	A	2348661.5	800983.5	-73.061	-42.052	6059	-0.773429648	-2.866475189	-1.140503007
174070	4/22/2019 15:48	B	1714595.824	496172.6756	-72.998	-42.06	2547	-1.06489103	-2.866475189	-1.03891242
174070	4/22/2019 20:42	2	164979.6928	44140.80718	-72.87	-42.099	17647	-1.166054023	-2.866475189	-1.026415709
174070	4/22/2019 21:29	1	559675.4145	77345.08548	-72.94	-42.108	2853	-1.276234804	-2.866475189	-1.015191576
174070	4/22/2019 22:23	1	4526636.723	210083.277	-72.898	-42.123	3227	-1.200985832	-2.866475189	-1.033157539
174070	4/22/2019 23:07	B	400227284.2	37418628.35	-72.864	-42.156	2606	-1.141436395	-2.866475189	-1.069926571
174070	4/22/2019 23:48	2	167423.599	14904.90099	-72.877	-42.221	2486	-1.175417811	-2.866475189	-1.113102148
174070	4/23/2019 0:39	B	267373.8722	1396355.128	-72.903	-42.224	3052	-1.032190769	-2.866475189	-1.068440379

174070	4/23/2019 1:42	A	1957969.924	91992.576	-72.96	-42.355	3811	-1.088012901	-2.866475189	-1.192294773
174070	4/23/2019 2:21	B	7671444.5	1304112.5	-72.967	-42.384	2332	-0.920153323	-2.866475189	-1.15959028
174070	4/23/2019 2:24	B	184587158.5	21889164.03	-72.962	-42.384	187	-0.919565015	-2.866475189	-1.163410302
174070	4/23/2019 2:55	B	247282455.5	9373557.539	-73.028	-42.352	1839	-0.635780874	-2.795881279	-1.252165792
174070	4/23/2019 3:24	2	110127.5732	463806.9268	-73.034	-42.33	1737	-0.353726817	-2.365253254	-1.25475515
174070	4/23/2019 4:05	2	114211.0536	12671.44644	-73.033	-42.287	2443	-0.600041338	-2.153074204	-1.1590539
174070	4/23/2019 8:18	B	16480262.5	16480262.5	-73.003	-42.271	15192	-0.821037922	-2.659442796	-1.09242425
174070	4/23/2019 9:40	B	388996.178	1865860.322	-72.954	-42.267	4933	-1.116990535	-2.866475189	-1.074720087
174070	4/23/2019 9:58	A	85315762.8	7017289.698	-72.94	-42.277	1063	-0.970465526	-2.866475189	-1.086957727
174070	4/23/2019 11:05	A	98999781.98	6154980.518	-73.021	-42.327	4009	-0.671208105	-2.672410569	-1.216372416
174070	4/23/2019 11:18	B	11929965.84	373498.6606	-73.003	-42.329	824	-0.735512756	-2.733479855	-1.213594391
174070	4/23/2019 12:06	B	2573481.725	142360.2754	-73.024	-42.35	2854	-0.515561096	-2.693892532	-1.268258728
174070	4/23/2019 12:50	B	4426749.402	257878.5979	-73.031	-42.347	2665	-0.474709441	-2.639794658	-1.275695214
174070	4/23/2019 12:59	B	318056.2521	1991960.748	-72.975	-42.364	539	-0.972121417	-2.866475189	-1.190870656
